# 2-Oxabicyclo[2.1.1]hexanes as saturated bioisosteres of the *ortho*-substituted phenyl ring

**DOI:** 10.1038/s41557-023-01222-0

**Published:** 2023-06-05

**Authors:** Aleksandr Denisenko, Pavel Garbuz, Nataliya M. Voloshchuk, Yuliia Holota, Galeb Al-Maali, Petro Borysko, Pavel K. Mykhailiuk

**Affiliations:** 1 Enamine Ltd, Kyiv, Ukraine; 2 National University of Life and Environmental Science of Ukraine, Kyiv, Ukraine; 3 Bienta, Kyiv, Ukraine; 4 M.G. Kholodny Institute of Botany of the National Academy of Sciences of Ukraine, Kyiv, Ukraine

**Keywords:** Drug discovery and development, Synthetic chemistry methodology, Synthetic chemistry methodology

## Abstract

The *ortho*-substituted phenyl ring is a basic structural element in chemistry. It is found in more than three hundred drugs and agrochemicals. During the past decade, scientists have tried to replace the phenyl ring in bioactive compounds with saturated bioisosteres to obtain novel patentable structures. However, most of the research in this area has been devoted to the replacement of the *para*-substituted phenyl ring. Here we have developed saturated bioisosteres of the *ortho*-substituted phenyl ring with improved physicochemical properties: 2-oxabicyclo[2.1.1]hexanes. Crystallographic analysis revealed that these structures and the *ortho*-substituted phenyl ring indeed have similar geometric properties. Replacement of the phenyl ring in marketed agrochemicals fluxapyroxad (BASF) and boscalid (BASF) with 2-oxabicyclo[2.1.1]hexanes dramatically improved their water solubility, reduced lipophilicity and most importantly retained bioactivity. This work suggests an opportunity for chemists to replace the *ortho*-substituted phenyl ring in bioactive compounds with saturated bioisosteres in medicinal chemistry and agrochemistry.

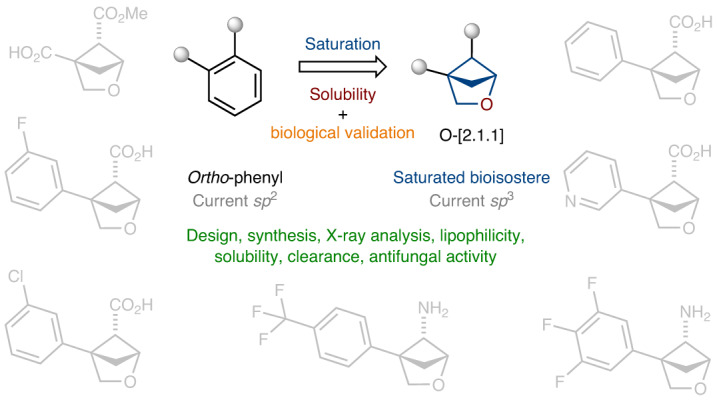

## Abstract (web summary)

The *ortho*-substituted phenyl ring is a basic structural element in chemistry. Now, 2-oxabicyclo[2.1.1]hexanes have been developed as saturated bioisosteres of the *ortho*-substituted phenyl ring with improved physicochemical properties. Replacement of the phenyl ring with 2-oxabicyclo[2.1.1]hexanes in marketed agrochemicals fluxapyroxad and boscalid improved water solubility, reduced lipophilicity and retained bioactivity.

## Main

The phenyl ring is a basic structural element in chemistry. Moreover, it is one of the most common rings in bioactive compounds^1^. However, organic compounds with more than two phenyl rings often have poor solubility and low metabolic stability—undesired effects in medicinal chemistry^2^. In this context, during the last decade, the concept ‘escape from flatland’ became popular^3,4^. Today, medicinal chemists prefer using F(*sp*^3^)-rich structures in drug discovery projects^5–10^. The replacement of the phenyl ring in bioactive compounds with saturated bioisosteres has become a popular tactic to obtain novel structures with an improved physicochemical profile^11–14^. However, most of the research in this area is devoted to the replacement of monosubstituted and *para*-disubstituted phenyl rings^12,15–49^.

**Fig. 1:**
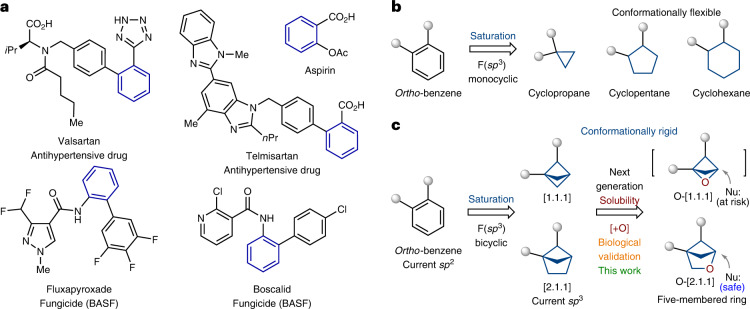
The *ortho*-substituted phenyl ring and its saturated bioisosteres. **a**, The *ortho*-substituted phenyl ring is a part of >300 drugs and agrochemicals. **b**, Previous examples of replacement of the *ortho*-substituted phenyl ring in bioactive compounds with monocyclic saturated rings by Qiao (2008) (ref. ^51^) and Shinozuka (2020) (ref. ^52^). **c**, Previous examples of replacement of the *ortho*-substituted phenyl ring in bioactive compounds with bicyclic saturated scaffolds: bicyclo[1.1.1]pentane (Ma, 2020 (ref. ^53^); Baran, 2021 (ref. ^54^)) and bicyclo[2.1.1]hexane (Mykhailiuk, 2020 (ref. ^55^); Brown, 2022 (ref. ^56^); Procter, 2023 (ref. ^57^)). The aim of this work is the replacement of the *ortho*-substituted phenyl ring in bioactive compounds with 2-oxabicyclo[2.1.1]hexane.[+O], replacement of a methylene group $(-{\mathrm{CH}}_{2^-})$ with an oxygen atom (−O−).

*Ortho*-disubstituted phenyl rings are found in more than three hundred drugs and agrochemicals (www.drugbank.ca; Fig. 1a) (ref. ^50^). For example, aspirin, which is widely known, contains an *ortho*-disubstituted phenyl ring in its structure. In 2008, the first example of mimicking an *ortho*-disubstituted phenyl ring in a bioactive compound with a saturated bioisostere, cyclopropane, appeared in the literature (Fig. 1b) (ref. ^51^). Later, similar studies were performed with 1,2-disubstituted cyclopentanes and cyclohexanes (Fig. 1b) (ref. ^52^). In the past two years, great progress has been achieved with saturated bicyclic scaffolds, which, compared to previously used monocyclic counterparts, are intrinsically conformationally rigid. In particular, 1,2-disubstituted bicyclo[1.1.1]pentanes (Fig. 1c) (refs. ^53,54^) and bicyclo[2.1.1]hexanes (Fig. 1c) (refs. ^55–61^) were used as saturated bioisosteres of the *ortho*-disubstituted phenyl ring. In this work, we report on the preparation, characterization and biological validation of the next generation of these saturated bioisosteres, 2-oxabicyclo[2.1.1]hexanes, analogues of the *ortho*-substituted phenyl ring with improved physicochemical properties (Fig. 1c).

## Design

In the design of a core with a similar structure to bicyclo[1.1.1]pentanes and bicyclo[2.1.1]hexanes, but having reduced lipophilicity and enhanced water solubility, we decided to insert an oxygen atom. Replacing the methylene group in bicyclo[1.1.1]pentane with the oxygen atom leads to a strained oxetane structure (Fig. 1c), which is interesting but could be labile due to possible ring opening with nucleophiles^62^. Analogous replacement in bicyclo[2.1.1]hexanes, however, gives the substituted tetrahydrofuran (Fig. 1c). That core should be more chemically stable, so we decided to make it. From a medicinal chemistry perspective, having the ether oxygen atom in the molecule is also useful, since it could serve as an additional binding site to a receptor.

Inspiration came from our previous work, where we synthesized bridgehead disubstituted 2-oxabicyclo[2.1.1]hexanes and believed they could mimic the *meta*-disubstituted phenyl ring in bioactive compounds^63^. This hypothesis, however, was not validated. Therefore, here we decided to prepare the disubstituted 2-oxabicyclo[2.1.1]hexanes and biologically validate them as bioisosteres of the *ortho*-disubstituted phenyl ring.

## Synthesis

**Fig. 2:**
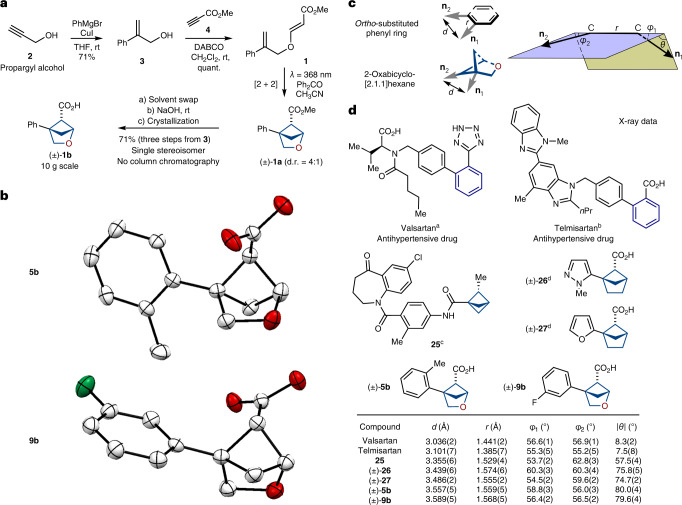
Synthesis and crystallographic analysis of 2-oxabicyclo[2.1.1]hexanes. **a**, Gram-scale synthesis of compound **1b**. THF, tetrahydrofuran; quant., quantitative. **b**, X-ray crystal structures of compounds **5b** and **9b**. Hydrogen atoms are omitted for clarity. Carbon, grey; oxygen, red; fluorine, green. **c**, Definition of vectors **n**_1_ and **n**_2_, and geometric parameters *d*, *r*, *φ*_1_, *φ*_2_ and *θ*. *Ortho*-substituted phenyl ring and 2-oxabicyclo[2.1.1]hexane are shown as examples. **d**, Geometric parameters *d*, *r*, *φ*_1_, *φ*_2_ and |*θ*| for *ortho*-substituted benzenes (valsartan, telmisartan), the saturated literature bioisosteres **25**–**27** and water-soluble saturated bioisosteres **5b** and **9b**. ^a^Data is taken from ref. ^67^. ^b^Data is taken from ref. ^68^. ^c^Data is taken from ref. ^54^. ^d^Data is taken from ref. ^55^.

The photochemical [2 + 2] cycloaddition between alkenes proved to be a powerful strategy to construct cyclobutanes^64^. In this context, we wondered if diene **1** (easily obtained from the commercially available starting materials; Fig. 2a) would undergo an intramolecular cyclization into the needed 2-oxabicyclo[2.1.1]hexane core. Direct irradiation of diene **1** in acetonitrile under different wavelengths gave only traces of product (Table 1, entries 1–4). Irradiation with a Hanovia broad wavelength mercury lamp gave the needed product along with many side products (entry 5). Next, we tried the addition of available organic ketones for the triplet sensitization of the styrene moiety. Indeed, smooth formation of the needed product **1a** (d.r. = ~4:1) was observed under irradiation at 368 nm. The best result was obtained with benzophenone (entry 7), whereas acetophenone and substituted benzophenones also worked but provided lower yields of the end product (entries 6, 8 and 9). Among all tested solvents (entries 10–13), the best outcome was obtained in acetonitrile. Without irradiation, the reaction did not take place at room temperature or with heating (entries 14 and 15).

**Table 1:** Optimization of the synthesis of compound 1a


Entry	Conditions	Yield (%)^a,b^
1	419 nm, CH_3_CN	n.d.
2	368 nm, CH_3_CN	<5
3	313 nm, CH_3_CN	<5
4	254 nm, CH_3_CN	<20
5	Hanovia mercury lamp, CH_3_CN	39
6	368 nm, CH_3_CN, PhC(O)Me	47
7	368 nm, CH_3_CN, Ph_2_CO	81 (56)^c^
8	368 nm, CH_3_CN, (p-MeOC_6_H_4_)_2_CO	61
9	368 nm, CH_3_CN, (p-NO_2_C_6_H_4_)_2_CO	38
10	368 nm, CH_2_Cl_2_, Ph_2_CO	71
11	368 nm, Me_2_CO, Ph_2_CO	68
12	368 nm, PhMe, Ph_2_CO	42
13	368 nm, EtOAc, Ph_2_CO	29
14	No light, rt	n.d.
15	No light, reflux	n.d.

^a^Reactions were performed on a 20 mmol scale. ^b^The ^1^H-NMR yield of the major isomer (CH_2_Br_2_ as an internal standard). ^c^Isolated yield of major stereoisomer of **1a**. rt, room temperature; *λ*, wavelength; n.d., not determined.

Under optimized conditions, cyclization of diene **1** led to a rather clean formation of a diastereomeric mixture of products **1a** (d.r. = 4:1); however, the pure major isomer **1a** was isolated by column chromatography in only 56% yield. The separation of isomers was problematic and led to a notable loss of yield, which needed to be solved.

## Scaled-up synthesis

The optimized synthetic protocol is shown in Fig. 2a. It was important to identify a method that employed only available and cheap starting materials. The synthesis started from propargyl alcohol (**2**). Copper-catalysed reaction with phenyl magnesium bromide gave alcohol **3** in 71% yield following the reported procedure^65^. A Michael addition of the latter with methyl propiolate (**4**) in the presence of (1,4-diazabicyclo[2.2.2]octane) (DABCO) provided the needed diene **1**. We mentioned that compound **1** partially decomposed during column chromatography and under storage at room temperature. Therefore, we decided to generate crude diene **1** in situ and use it directly in the photochemical step (Supplementary Information, page 6). A mixture of isomers **1a** was obtained. After extensive experimentation, we found a way to avoid column chromatography and not lose the yield. The crude reaction mixture after irradiation (isomers **1a** and benzophenone) was saponified with sodium hydroxide. A standard workup (removal of benzophenone) followed by crystallization from a hexane–MeO*t*Bu mixture to remove the minor isomer allowed the isolation of pure major isomer **1b** at 71% yield in three steps from alcohol **3**. Product **1b** was obtained on a ten gram scale with no column purifications.

## Scope

Next, we studied the scope of the developed method. The photocyclization method tolerated various substituents on the aromatic core (Table 2). Among them were alkyl groups (**5a**–**8a**), fluorine atoms (**9a**–**11a**) and chlorine atoms (**12a** and **13a**), methoxy groups (**14a**–**16a**) and trifluoromethyl groups (**17a**–**19a**). The reaction was also compatible with various substituted pyridines (**20a**–**24a**). In all cases, we isolated analytical quantities of intermediate esters **5a**–**24a** by column chromatography to characterize them. On a gram scale, however, we directly used crude reaction mixtures with **5a**–**24a** after photocyclization in the subsequent saponification step. In half of all cases, we could obtain the final carboxylic acids by simple crystallization of crude reaction mixtures from various solvents (Table 2). In the other half of the cases, column chromatography was still needed. The structure of carboxylic acids **5b** and **9b** was confirmed by X-ray crystallographic analysis (Fig. 2b).

**Table 2:**
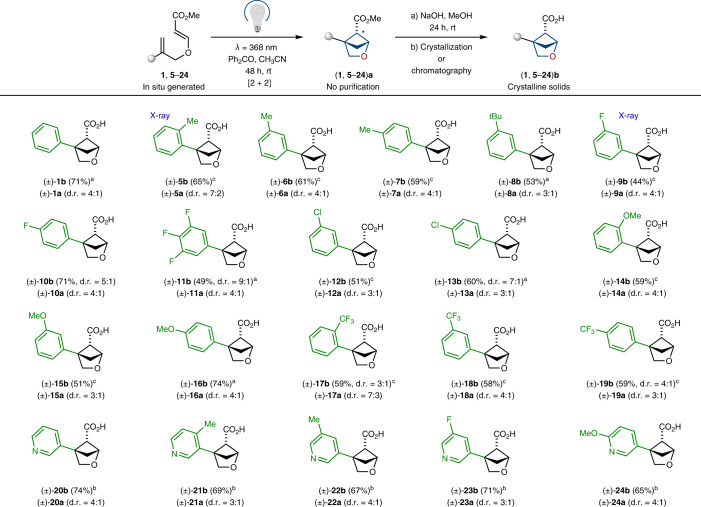
Scope of the reaction

All reactions were performed on a gram scale. Diastereomeric ratio of esters ‘a’ in crude reaction mixtures after the photochemical step is given. Isolated yields of acids ‘b’ are given in three steps from allylic alcohols. ^a^Product was isolated by crystallization from hexane–MeO*t*Bu mixture. ^b^Product was isolated by crystallization from acetone–water mixture. ^c^Product was isolated by column chromatography.

## Chemical stability

We also checked the chemical stability of three representative carboxylic acids, **1b**, **19b** and **22b** (Table 2), because we suspected that some of them could decompose via a retro-Michael-type reaction. Treatment of them with aqueous 1 M hydrochloric acid or aqueous 1 M sodium hydroxide at room temperature for one day did not lead to any decomposition. All products were crystalline solids, and we stored all of them in closed vials at room temperature on the shelf. The ^1^H-NMR, liquid chromatography–mass spectrometry (LC-MS) inspection after three months did not reveal any decomposition.

## Crystallographic analysis

Next, we compared the geometric parameters of 2-oxabicyclo[2.1.1]hexanes with those of the *ortho*-substituted phenyl ring and their previously suggested saturated bioisosteres, bicyclo[1.1.1]pentanes and bicyclo[2.1.1]hexanes. For that, we employed the exit vector plots tool^66^. In this method, substituents at the disubstituted scaffold were simulated by two exit vectors **n**_**1**_ and **n**_**2**_ (Fig. 2c). The relative spatial arrangement of vectors is described by four geometric parameters: the distance between C-C atoms *r*; the plane angles *φ*_1_ (between **n**_**1**_ and the C atom) and *φ*_2_ (between **n**_**2**_ and the C atom); and the dihedral angle *θ* defined by vectors **n**_**1**_, C-C and **n**_**2**_. An additional important parameter—the distance *d* between two carbon substituents (Fig. 2c)—was also measured.

We calculated the values of *d*, *r*, *φ*_1_, *φ*_2_ and *θ* of 2-oxabicyclo[2.1.1]hexanes from the X-ray data of compounds **5b** and **9b**. The related parameters for bicyclo[1.1.1]pentane **25** (ref. ^54^) and bicyclo[2.1.1]hexanes **26** and **27** (ref. ^55^) were calculated from their X-ray data published in the literature. The corresponding parameters for *ortho*-substituted phenyl rings were calculated from the reported crystal data of two antihypertensive drugs—valsartan^67^ and telmisartan^68^ (Fig. 2d). Analysis of this data revealed that the geometric properties of 2-oxabicyclo[2.1.1]hexanes in general were similar to those of the *ortho*-substituted phenyl ring. In particular, the distance *r* in 2-oxabicyclo[2.1.1]hexanes was ~0.2 Å longer than that in the *ortho*-phenyl ring: 1.56–1.57 Å versus 1.38–1.44 Å, respectively. The distance *d* between substituents in 2-oxabicyclo[2.1.1]hexanes was also ~0.5 Å longer than that in the *ortho*-phenyl ring: 3.6 Å versus 3.0–3.1 Å, respectively. Angles *φ*_1_ and *φ*_2_ were almost identical in both scaffolds. Moreover, *φ*_1_ and *φ*_2_ in 2-oxabicyclo[2.1.1]hexanes were much closer to those in the *ortho*-phenyl ring, than to those of the previously used saturated bioisosteres: bicyclo[1.1.1]pentanes and bicyclo[2.1.1]hexanes. The difference in planarity was notable: while *ortho*-phenyl was almost flattened (|*θ*| = 7–8°), 2-oxabicyclo[2.1.1]hexanes had a substantial three-dimensional character: |*θ*| = 80°. It must be noted, however, that non-planarity was also present in bicyclo[1.1.1]pentanes (|*θ*| = 58°) and bicyclo[2.1.1]hexanes (|*θ*| = ~75°; Fig. 2d).

In general, vector characteristics of 2-oxabicyclo[2.1.1]hexanes were very similar to those of the previously used bioisosteres of the *ortho*-substituted phenyl ring: bicyclo[1.1.1]pentanes and bicyclo[2.1.1]hexanes. Moreover, the important angles *φ*_1_ and *φ*_2_ in 2-oxabicyclo[2.1.1]hexanes were even closer to those in the *ortho*-phenyl ring than to those in bicyclo[1.1.1]pentanes and bicyclo[2.1.1]hexanes.

## Incorporation into bioactive compounds

**Fig. 3:**
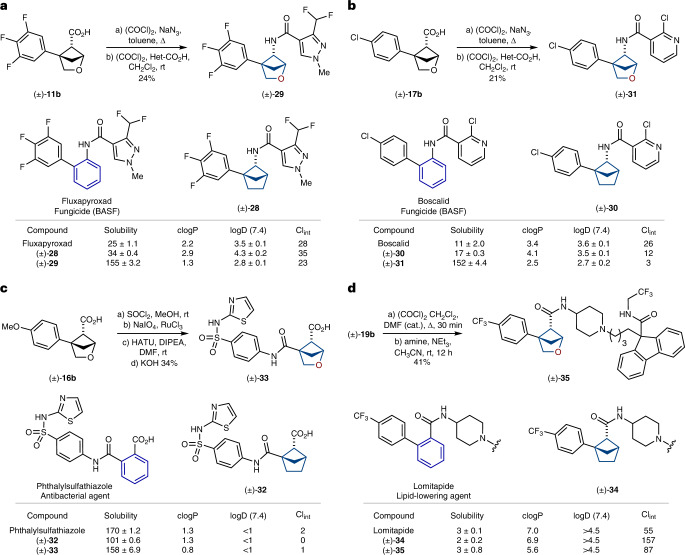
Replacement of the *ortho*-phenyl ring with saturated bioisosteres in drugs. **a**, Synthesis and properties of compounds **28** and **29**, saturated bioisosteres of fluxapyroxad. Δ, heating; Het-CO_2_H, 3-(difluoromethyl)-1-methyl-1H-pyrazole-4-carboxylic acid. **b**, Synthesis and properties of compounds **30** and **31**, saturated bioisosteres of boscalid. Het-CO_2_H, 2-chloropyridine-3-carboxylic acid. **c**, Synthesis and properties of compounds **32** and **33**, saturated bioisosteres of phthalylsulfathiazole. DIPEA, *N*,*N*-diisopropylethylamine; DMF, dimethylformamide. **d**, Synthesis and properties of compounds **34** and **35**, saturated bioisosteres of Lomitapide. Solubility (in µM) refers to the experimental kinetic solubility in phosphate-buffered saline at pH 7.4; clogP is the calculated lipophilicity; logD (7.4) refers to the experimental distribution coefficient in *n*-octanol/phosphate-buffered saline at pH 7.4. Reliable logD measures were obtained within a range 1.0–4.5. CI_int_, intrinsic clearance, experimental metabolic stability in human liver microsomes (in µl min^–1^ mg^–1^). cat., catalyst.

The incorporation of the 2-oxabicyclo[2.1.1]hexane scaffold into bioactive compounds was attempted next. We chose four bioactive products with the *ortho*-substituted phenyl ring: agrochemical fungicides fluxapyroxad and boscalid, antibacterial agent phthalylsulfathiazole and lipid-lowering agent lomitapide (Fig. 3).

Synthesis of the saturated analogue of fluxapyroxad was undertaken from carboxylic acid **11b** (Fig. 3a). The standard Curtius reaction followed by acylation of the intermediate amine with the substituted pyrazole carboxylic acid gave the needed compound **29**. Using an analogous tactic, compound **31**—a saturated analogue of boscalid—was also obtained from carboxylic acid **17b** (Fig. 3b). The saturated analogue of phthalylsulfathiazole was obtained by converting carboxylic acid **16b** first into the methyl ester followed by the oxidation of the phenyl ring. Amide coupling of the formed acid with the *para*-substituted aniline followed by saponification of the methyl ester gave the final compound **33** (Fig. 3c). Amide coupling of carboxylic acid **19b** with the correspondingly *N*-substituted 4-aminopiperidine gave compound **35**, a saturated analogue of lomitapide (Fig. 3d).

In all cases, in addition to bioactive compounds with a 2-oxabicyclo[2.1.1]hexane core (**29**, **31**, **33** and **35**), we also synthesized analogous carbocyclic analogues **28**, **30**, **32** and **34** (Fig. 3 and Supplementary Information, pages 34–42).

## Physicochemical parameters

In the next step, we studied the effect of the replacement of the *ortho*-phenyl ring by 2-oxabicyclo[2.1.1]hexanes on the physicochemical properties of bioactive compounds. For the comparison, we also used the corresponding carbocyclic core, bicyclo[2.1.1]hexane.

## Water solubility

Replacement of the *ortho*-substituted phenyl ring in fluxapyroxad by bicyclo[2.1.1]hexane (**28**) slightly increased its solubility (Fig. 3a). However, incorporation of the 2-oxabicyclo[2.1.1]hexane in fluxapyroxad (**29**) resulted in a dramatic sixfold increase in solubility: 25 µM (fluxapyroxad) versus 34 µM (**28**) versus 155 µM (**29**). An analogous trend was also seen with boscalid and its analogues **30** and **31** (Fig. 3b). Replacement of the phenyl ring in boscalid with bicyclo[2.1.1]hexane (**30**) led to the increase of solubility by ~50%. However, the corresponding replacement with 2-oxabicyclo[2.1.1]hexane (**31**) increased the solubility by more than ten times: 11 µM (boscalid) versus 17 µM (**30**) versus 152 µM (**31**). Replacement of the phenyl ring in phthalylsulfathiazole with bicyclo[2.1.1]hexane (**32**) decreased its solubility, while the incorporation of the 2-oxabicyclo[2.1.1]hexane core (**33**) restored it: 170 µM (phthalylsulfathiazole) versus 101 µM (**32**) versus 158 µM (**33**; Fig. 3c). Lomitapide had poor solubility in water, and replacement of the phenyl ring in lomitapide with saturated bioisosteres (**34** and **35**) did not have any substantial impact on the solubility (Fig. 3d).

In summary, in two (fluxapyroxad, boscalid) out of four bioactive compounds, replacement of the *ortho*-substituted phenyl ring with 2-oxabicyclo[2.1.1]hexane led to a dramatic increase in water solubility by about one order of a magnitude.

## Lipophilicity

To estimate the influence of the replacement of the *ortho*-substituted phenyl ring with saturated bioisosteres on lipophilicity, we used two parameters: calculated lipophilicity, log *P*, where *P* is the partition coefficient (clogP), and experimental lipophilicity, log *D*, where *D* is the distribution coefficient (logD).

Replacement of the phenyl ring with bicyclo[2.1.1]hexane either led to an increase of clogP (fluxapyroxad, boscalid; Fig. 3a,b) or did not affect it (phthalylsulfathiazole, lomitapide; Fig. 3c,d). However, in all four bioactive compounds, incorporation of 2-oxabicyclo[2.1.1]hexane instead of the *ortho*-substituted phenyl ring led to a decrease of the clogP index by about one unit.

The effect of the replacement of the *ortho*-substituted phenyl ring with saturated bioisosteres on the logD index was more complex. In fluxapyroxad, incorporation of the bicyclo[2.1.1]hexane core increased logD, while the incorporation of 2-oxabicyclo[2.1.1]hexane slightly decreased it: 3.5 (fluxapyroxad) versus 4.3 (**28**) versus 2.8 (**29**; Fig. 3a). In boscalid, the incorporation of the bicyclo[2.1.1]hexane core did not affect logD substantially, while the incorporation of 2-oxabicyclo[2.1.1]hexane reduced it: 3.6 (boscalid) versus 3.5 (**28**) versus 2.7 (**29**; Fig. 3b).

In summary, in all tested compounds, replacement of the *ortho*-substituted phenyl ring with 2-oxabicyclo[2.1.1]hexane decreased the lipophilicity as measured by both clogP and logD indexes by 0.5–1.4 units.

## Metabolic stability

The effect of saturated bioisosteres on the metabolic stability of bioactive compounds was complex and depended on the chemical structure. In fluxapyroxad, the incorporation of bicyclo[2.1.1]hexane (**28**) decreased the metabolic stability (Fig. 3a). However, incorporation of 2-oxabicyclo[2.1.1]hexane (**29**) unexpectedly increased it: the experimental metabolic stability in human liver microsomes, intrinsic clearance, CI_int_ (mg min^–1^ μl^–1^) = 28 (fluxapyroxad) versus 35 (**28**) versus 23 (**29**). In boscalid, incorporation of the bicyclo[2.1.1]hexane (**30**) increased the metabolic stability, but the incorporation of 2-oxabicyclo[2.1.1]hexane (**31**) increased it even more: CI_int_ (mg min^–1^ μl^–1^) = 26 (boscalid) versus 12 (**30**) versus 3 (**31**; Fig. 3b). All three compounds, phthalylsulfathiazole and its two saturated analogues **32** and **33**, were metabolically stable (Fig. 3c). In lomitapide, incorporation of the bicyclo[2.1.1]hexane core (**34**) decreased the metabolic stability, but the incorporation of the 2-oxabicyclo[2.1.1]hexane core (**35**) somewhat restored it: CI_int_ (mg min^–1^ μl^–1^) = 55 (lomitapide) versus 157 (**34**) versus 87 (**35**; Fig. 3d).

In summary, replacement of the *ortho*-substituted phenyl ring with 2-oxabicyclo[2.1.1]hexane in bioactive compounds improved metabolic stability (CI_int_) in boscalid and fluxapyroxad; slightly decreased it in lomitapide; and did not affect it in phthalylsulfathiazole.

## Bioactivity

**Fig. 4:**
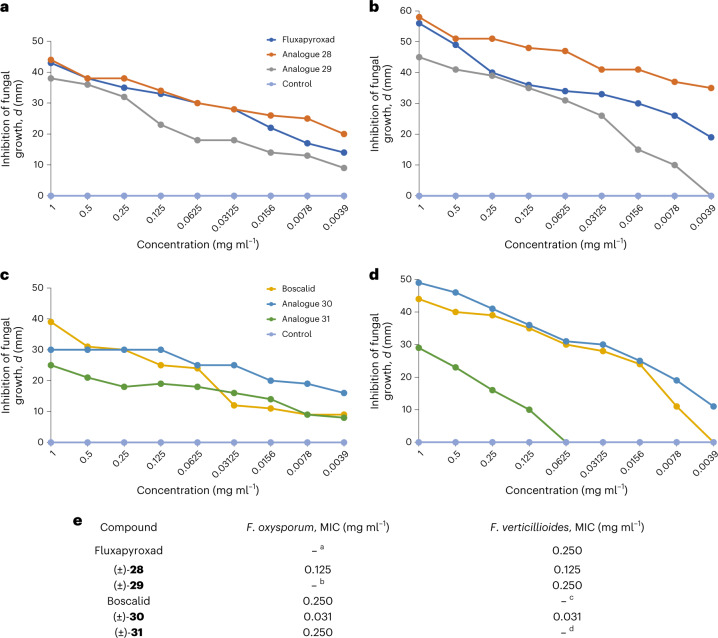
Antifungal activity of fungicides fluxapyroxad, boscalid and their saturated analogues 28–31. **a**,**b**, Inhibition of growth of *F. oxysporum* (**a**) and *F. verticillioides* (**b**; measured as a diameter *d* of the inhibition zone, in millimetres), by fluxapyroxad and its saturated analogues **28** and **29** at different concentrations after 48 h of incubation. **c**,**d**, Inhibition of growth of *F. oxysporum* (**c**) and *F. verticillioides* (**d**; measured as a diameter of the inhibition zone, in millimetres) by boscalid and its saturated analogues **30** and **31** at different concentrations after 48 h of incubation. **e**, MIC for fluxapyroxad and its analogues **28** and **29**, and for boscalid and its analogues **30** and **31**. ^a^Maximal inhibition of growth of *F. oxysporum* by 38.0 ± 1.9% at a concentration of 0.031 mg ml^–1^. ^b^Maximal inhibition of growth of *F. oxysporum* by 35.7 ± 2.4% at a concentration of 0.125 mg ml^–1^. ^c^Maximal inhibition of growth of *F. verticillioides* by 39.2 ± 2.7% at a concentration of 0.063 mg ml^–1^. ^d^Maximal inhibition of growth of *F. verticillioides* 36.3 ± 1.9% at a concentration of 0.250 mg ml^–1^.

Finally, we wanted to answer a key question: can 2-oxabicyclo[2.1.1]hexanes indeed mimic the *ortho*-substituted phenyl ring in real-world bioactive compounds? Fluxapyroxad and boscalid are marketed fungicides, developed by BASF, that have been approved for use in the United States and the European Union. Therefore, we measured their antifungal activity and compared it to that of their saturated analogues **28**–**31**. In strict contrast to medicinal chemistry, the use of racemic mixtures in agrochemistry is common^50^; therefore for the primary validation of the proof-of-concept, we directly studied the biological activity of the available racemic compounds **28**–**31** (Fig. 4).

First, we measured the antifungal activity of all compounds using the agar well diffusion method (Supplementary Information, pages 238–243). Fluxapyroxad, and its saturated analogues **28** and **29**, showed a similar trend in activity at the inhibition of fungi growth (Fig. 4a,b). The 2-oxabicyclo[2.1.1]hexane analogue **29** was active, but less potent compared to the original fungicide. Compound **29** and fluxapyroxad almost identically inhibited the growth of *Fusarium oxysporum* at high concentrations; however, at low concentrations, analogue **29** showed lower activity (Fig. 4a). Similarly, **29** and fluxapyroxad effectively inhibited the growth of *Fusarium verticillioides* at high concentrations; however, at low concentrations, only fluxapyroxad remained active, while analogue **29** did not (Fig. 4b).

Boscalid and both saturated analogues **30** and **31** also effectively inhibited the fungi growth (Fig. 4c,d). However, 2-oxabicyclo[2.1.1]hexane **31** was slightly less potent than boscalid at the inhibition of *F. oxysporum* (Fig. 4c) and notably less potent at the inhibition of *F. verticillioides*, especially at low concentrations (Fig. 4d).

Additionally, we measured a minimal inhibitory concentration (MIC) of all compounds (Fig. 4e). Interestingly, fluxapyroxad and its 2-oxabicyclo[2.1.1]hexane analogue **29** exhibited equal MIC values of 0.250 mg ml^–1^ at the inhibition of the growth of *F. verticillioides*. Carbocyclic analogue **30** was two times as potent: MIC = 0.125 mg ml^–1^. At the same time, boscalid and its 2-oxabicyclo[2.1.1]hexane analogue **31** also exhibited equal MIC values of 0.250 mg ml^–1^ at the inhibition of the growth of *F. oxysporum*. Carbocyclic analogue **30** was much more potent: MIC = 0.031 mg ml^–1^.

## Conclusion

The *ortho*-substituted phenyl ring (as well as *meta* and *para* isomers) is a basic structural element in chemistry. In this work, we synthesized, characterized and studied 2-oxabicyclo[2.1.1]hexanes as saturated bioisosteres of the *ortho*-substituted phenyl ring (Fig. 1c). These scaffolds were synthesized from readily available starting materials on a multigram scale. Crystallographic analysis revealed that these structures and the *ortho*-substituted phenyl ring indeed have similar geometric properties. Replacement of the *ortho*-substituted phenyl ring in bioactive compounds with 2-oxabicyclo[2.1.1]hexanes, in two out of four cases, dramatically improved water solubility (up to more than ten times) and metabolic stability. Moreover, in all four cases, such replacement also reduced lipophilicity by 0.5–1.4 clogP or logD units (Fig. 3b). In addition, the 2-oxabicyclo[2.1.1]hexanes **29** and **31** showed a similar antifungal activity compared to that of the original fungicides fluxapyroxad and boscalid.

Given the commonplace nature of the *ortho*-substituted phenyl ring in chemistry, we believe that its saturated bioisosteres described in this work will soon become very popular. One must always keep in mind, however, that the replacement of the phenyl ring in bioactive compounds with saturated isosteres can fail, if the phenyl ring is involved in integrations with the receptor: *π*–*π* stacking, *π*–amide stacking, *π*–Asp/Glu/Arg stacking, *π* to amide N–H, *π* to O–H, *π* to S–H, *π* to ammonium salts and so on. Therefore, the replacement of the phenyl ring in bioactive compounds with saturated isosteres must be careful and balanced^69^.

## Methods

### General procedure for the photochemical [2 + 2] cycloaddition

The solution of diene **1** (16.79 g, 0.077 mol, 1.0 equiv.) and benzophenone (1.40 g, 0.0077 mol, 0.10 equiv.) in 850 ml of dry CH_3_CN (concentration = 0.091 M) was put into a standard chemical 1 l glass flask. The reaction mixture was degassed by the bubbling of argon for 15 min. The flask was closed by a septum and irradiated with luminescent UV lamps at 368 nm (24 lamps; Sylvania 368 Blacklight F25/T8/18/BL3368; each lamp has nominal power of 25 W; total power is 600 W), under stirring at room temperature for 48 h. The reaction mixture was concentrated under reduced pressure to provide the crude product **1a** that was used in the next step (saponification) without any purification.

NMR spectra were analysed with MestreNova (11.0.3-18688).

### Reporting summary

Further information on research design is available in the Nature Portfolio Reporting Summary linked to this article.

## Online content

Any methods, additional references, Nature Portfolio reporting summaries, source data, extended data, supplementary information, acknowledgements, peer review information; details of author contributions and competing interests; and statements of data and code availability are available at 10.1038/s41557-023-01222-0.

## Supplementary information

Supplementary Information Synthetic procedures, characterization of compounds (description of NMR spectra, high resolution mass spectrometry), photos of the experimental set-up of photochemical reactions, copies of NMR spectra, X-ray crystallographic data, determination of aqueous solubility, determination of lipophilicity (logD), determination of metabolic stability and determination of antifungal activity.

Reporting Summary

Supplementary Data 1 Crystallographic data for compound **5b**; CCDC reference 2166325.

Supplementary Data 2 Crystallographic data for compound **9b**; CCDC reference 2166326.

## Data Availability

Experimental data as well as characterization data for all new compounds prepared during these studies are provided in the Supplementary Information of this manuscript. The X-ray crystallographic coordinates for compounds **5b** and **9b** have been deposited at the Cambridge Crystallographic Data Center (CCDC) with accession codes 2166325 (**5b**) and 2166326 (**9b**). These data can be obtained free of charge from the Cambridge Crystallographic Data Center via www.ccdc.cam.ac.uk/structures/.
