# Supplementary material for: 2-Oxabicyclo[2.1.1]hexanes as saturated bioisosteres of the ortho-substituted phenyl ring
Source: Nat Chem. 2023 Jun 5;15(8):1155–63. doi: 10.1038/s41557-023-01222-0 (PMC10396955; doi:10.1038/s41557-023-01222-0)
Supplement: Supplementary file 1 — Synthetic procedures, characterization of compounds (description of NMR spectra, high resolution mass spectrometry), photos of the experimental set-up of photochemical reactions, copies of NMR spectra, X-ray crystallographic data, determination of aqueous solubility, determination of lipophilicity (logD), determination of metabolic stability and determination of antifungal activity. [file 41557_2023_1222_MOESM1_ESM.pdf]

nature chemistry

Article https://doi.org/10.1038/s41557-023-01222-0

2-Oxabicyclo[2.1.1]hexanes as saturated bioisosteres of the *ortho*-substituted phenyl ring

In the format provided by the authors and unedited

Table of Contents

Experimental Section. Data description and procedures 5

Illustration of the experimental set up of a photochemical step. 8

Copies of ^{1}H, $^{13}C\{^{1}H\}$ and $^{19}F\{^{1}H\}$ spectra 43

Compound 3 43

Compound 1, (ca. 90% purity) 45

Compound (±)-1a 48

Compound (±)-1b 50

Compound (±)-5a, (d.r. = 5:1) 54

Compound (±)-5b 56

Compound (±)-6a 60

Compound (±)-6b 62

Compound (±)-7a, d.r. = 3:2 66

Compound (±)-7b 68

Compound (±)-8a 72

Compound (±)-8b 74

Compound (±)-9a, d.r. = 4:1 79

Compound (±)-9b 82

Compound (±)-10a 88

Compound (±)-10b, d.r. = 5:1 91

Compound (±)-11a, d.r. = 4:1 97

Compound (±)-11b, d.r. = 9:1 100

Compound (±)-12a, d.r. = 9:2 105

Compound (±)-12b 107

Compound (±)-13a, d.r. = 4:1 111

Compound (±)-13b (d.r. = 7:1) 113

Compound (±)-14a 117

Compound (±)-14b 119

Compound (±)-15a 123

Compound (±)-15b 125

Compound (±)-16a 129

Compound (±)-16b 131

Compound (±)-17a, d.r. = 4:1 136

Compound (±)-17b, d.r. = 3:1 139

Compound (±)-18a, d.r. = 3:1 145

Compound (±)-18b 148

Compound (±)-19a, d.r. = 4:1 154

Compound (±)-19b, d.r. = 4:1 157

Compound (±)-20a 162

Compound (±)-20b 164

Compound (±)-21a 168

Compound (±)-21b 170

Compound (±)-22a, d.r. = 4:1 174

Compound (±)-22b 176

Compound (±)-23a, d.r. = 9:2 181

Compound (±)-23b 184

Compound (±)-24a 189

Compound (±)-24b 191

Compound (±)-28 193

Compound (±)-29 196

Compound (±)-31 199

Compound (±)-30 201

(±)-5-(Methoxycarbonyl)-2-oxabicyclo[2.1.1]hexane-4-carboxylic acid, (±)-S3 203

Compound (±)-33 205

5-(Methoxycarbonyl)bicyclo[2.1.1]hexane-1-carboxylic acid, (±)-S5 207

Compound (±)-32 209

Compound (±)-34 211

Compound (±)-35 214

Crystallographic data (X-Ray) 217

Analysis of Aqueous Solubility 225

Determination of Distribution Coefficient (LogD, pH 7.4) 233

Assessment of Metabolic Stability in Human Liver Microsomes 237

Bioactivity 245

Antifungal susceptibility testing 251

Experimental Section. Data description and procedures

General Considerations. All chemicals were provided by Enamine Ltd. (www.enamine.net). All solvents were treated according to standard methods. All reactions were monitored by thin-layer chromatography (TLC) and were visualized using UV light. Product purification was performed using HPLC: AGILENT 1260 INFINITY, a column Chromatorex C18 SMB 100-5T, 100*19 mm, 5 microm; PuriFlash XS420 Plus or by distillation under a reduce pressure. ^{1}H-NMR spectra were recorded at 400, 500 or 600 MHz (Varian); ^{19}F-NMR spectra were recorded at 376 MHz (Varian) and ^{13}C NMR spectra were recorded at 100, 126 or 151 MHz (Varian). ^{1}H-NMR chemical shifts are calibrated using residual undeuterated solvents $CHCl_3$ (δ = 7.26 ppm) or DMSO (δ = 2.50 ppm). ^{13}C-NMR chemical shifts for ^{13}C-NMR are reported relative to the central $CHCl_3$ (δ = 77.16 ppm) or DMSO (δ = 39.52 ppm). Coupling constants are given in Hz. High-resolution mass spectra (HRMS) were recorded on an Agilent LC/MSD TOF mass spectrometer by electrospray ionization time of flight reflectron experiments. D.r. of compounds “a” (esters) and “b” (acids) was measured by integrating CH signals (ca. 4.7-4.8 ppm in $CDCl_3$ or DMSO-d_6) in ^{1}H NMR spectra. clogP was calculated with “Cxcalc” (ChemAxon, version 22.5.0).

General procedure A (3 as an example)

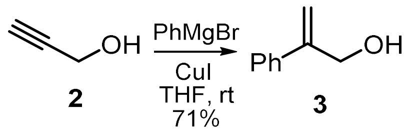

2-Phenylprop-2-en-1-ol (3)

According to a literature procedure (M. Wegmann, T. Bach, *Synthesis* **2017**, *49*, 209−217) in a flame dried three necked flask Mg turnings (8.64 g, 0.36 mol, 3.00 equiv) were covered with anhydrous THF (50 mL) under an inert atmosphere. Bromobenzene (47.10 g, 0.30 mol, 2.50 equiv) was dissolved in anhydrous THF (200 mL) and added dropwise in the presence of a small amount of iodide to help start the reaction. The rate of addition was adjusted to keep a constant reflux. After complete addition, the reaction mixture was heated at reflux for additional 1 h and then subsequently allowed to cool to a room temperature. CuI (11.43 g, 0.06 mol, 0.50 equiv) was added, the mixture stirred for 30 min, then propargyl alcohol (6.72 g, 0.12 mol, 1.00 equiv) in anhydrous THF (50 mL) was added slowly, and after complete addition the reaction mixture was stirred at a room temperature for additional 24 h. The reaction was quenched with a sat. NH_4Cl solution (100 mL) at 0 °C, allowed warming to room temperature. The layers were separated. The aqueous layer was extracted with MeO*t*Bu (3 × 100 mL), the combined organic

layers were washed with brine (1 × 100 mL), dried over $MgSO_4$, filtered and the solvent was removed under reduced pressure. The final product was purified by vacuum distillation (b.p. = 71-72 °C, 1 mmHg). Yield: 11.42 g, 0.085 mol, 71%, colorless oil. ^1H NMR (400 MHz, $CDCl_3$): δ 7.48 – 7.43 (m, 2H), 7.39 – 7.28 (m, 3H), 5.48 (s, 1H), 5.36 (s, 1H), 4.55 (s, 2H), 1.82 (s, 1H) ppm. $^{13}C\{^1H\}$ NMR (126 MHz, $CDCl_3$): δ 147.4, 138.6, 128.6, 128.1, 126.2, 112.7, 65.1 ppm. GCMS (M): 134. HRMS (ESI-TOF) *m/z*: $[M + H]^+$ calcd for $C_9H_{11}O$, 135.0810; found 135.0803.

General procedure B (compound 1 as an example)

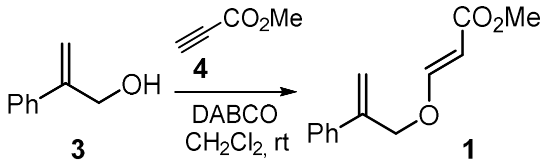

Methyl-3-((2-phenylallyl)oxy)acrylate (1)

To a solution of alcohol **3** (11.40 g, 0.085 mol, 1.00 equiv) in CH_2Cl_2 (150 mL) were added DABCO (0.95 g, 0.0085 mol, 0.10 equiv) and methyl propiolate (7.90 g, 0.094 mol, 1.10 equiv) at -15 °C. The reaction mixture was stirred at room temperature. The progress of the reaction was monitored by TLC. After completion of the reaction (~ 3 h), the solvent was removed on a rotary evaporator. The residue was dissolved in MeO*t*Bu (250 mL) and filtered through a SiO_2 pad. The solvent was removed on a rotary evaporator, and the crude product was used in a next step without further purification. Yield: 16.79 g, 0.077 mol, 90%, colorless oil, ~90% purity. ^1H NMR (500 MHz, $CDCl_3$): δ 7.63 (d, J = 12.6 Hz, 1H), 7.40 (d, J = 7.4 Hz, 2H), 7.36 (t, J = 7.3 Hz, 2H), 7.33 (t, J = 6.9 Hz, 1H), 5.61 (s, 1H), 5.39 (s, 1H), 5.32 (d, J = 12.6 Hz, 1H), 4.75 (s, 2H), 3.81 (d, J = 15.2 Hz, 1H), 3.70 (s, 3H) ppm. $^{13}C\{^1H\}$ NMR (126 MHz, $CDCl_3$): δ 168.2, 162.0, 142.1, 137.7, 128.7, 128.4, 126.1, 116.2, 97.3, 72.8, 51.3 ppm. LCMS $(M+H)^+$: 219. HRMS (ESI-TOF) *m/z*: $[M + H]^+$ calcd for $C_{13}H_{15}O_3$, 219.1021; found 219.1016.

General procedure C (1a as an example)

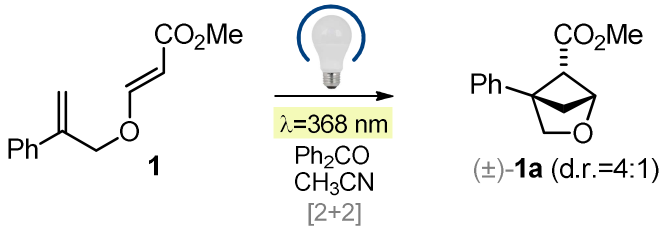

(±)-Methyl-4-phenyl-2-oxabicyclo[2.1.1]hexane-5-carboxylate (1a)

The solution of diene **1** (16.79 g, 0.077 mol, 1.0 equiv) and benzophenone (1.40 g, 0.0077 mol, 0.10 equiv) in 850 mL of dry CH_3CN (c = 0.1 M) was put into a standard chemical 1L glass

flask. The reaction mixture was degassed by the bubbling of argon for 15 min. The flask was closed by a septum and irradiated with luminescent UV lamps, 368 nm (24 lamps: Sylvania 368 Blacklight F25/T8/18/BL3368; each lamp has power 25 W; total power is 600 W), under stirring at room temperature for 48 h. The reaction mixture was concentrated under reduced pressure to provide the crude product. This crude material (mixture of diastereomers 4:1 and benzophenone) was directly used in the next step without any purification. D.r. was measured by integrating CH signals (ca. 4.7-4.8 ppm in $CDCl_3$) in ^{1}H NMR spectra of the crude reaction mixtures directly after photoirradiation. The same procedure was used in all cases.

An analytically pure sample of the product was obtained by purification of the sample of the crude mixture by HPLC: Rt = 2-10 min, water/acetonitrile, 30-55%, flow 30 mL/min (loading pump 4 mL/min). Yellow oil, single stereoisomer. ^{1}H NMR (400 MHz, $CDCl_3$): δ 7.39 (d, J = 7.1 Hz, 2H), 7.33 (t, J = 7.5 Hz, 2H), 7.29 – 7.23 (m, 1H), 4.79 (s, 1H), 4.29 (d, J = 5.9 Hz, 1H), 3.80 (d, J = 5.9 Hz, 1H), 3.69 (s, 3H), 2.87 (s, 1H), 1.96 (d, J = 7.7 Hz, 1H), 1.88 (d, J = 7.6 Hz, 1H) ppm. ^{13}C{^{1}H} NMR (126 MHz, $CDCl_3$): δ 169.5, 137.2, 128.7, 127.6, 127.1, 79.4, 69.8, 57.1, 54.9, 51.7, 42.6 ppm. GCMS (M): 218. HRMS (ESI-TOF) *m/z*: $[M + H]^+$ calcd for $C_{13}H_{15}O_3$, 219.1021; found 219.1015.

Irradiation with 368 nm was performed using 24 lamps (25W each)
"Sylvania 368 Blacklight F25/T8/18/BL3368"
https://www.sylvania-lighting.com/product/en-int/products/0002166/
Irradiation was performed until the disappearance of the starting material (ca. 48h).

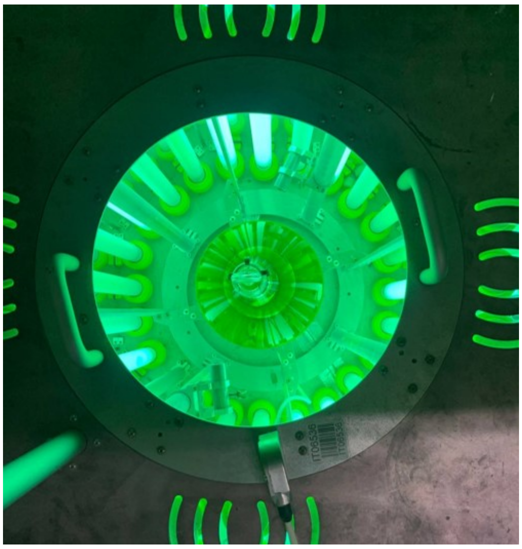
Photochemical step.

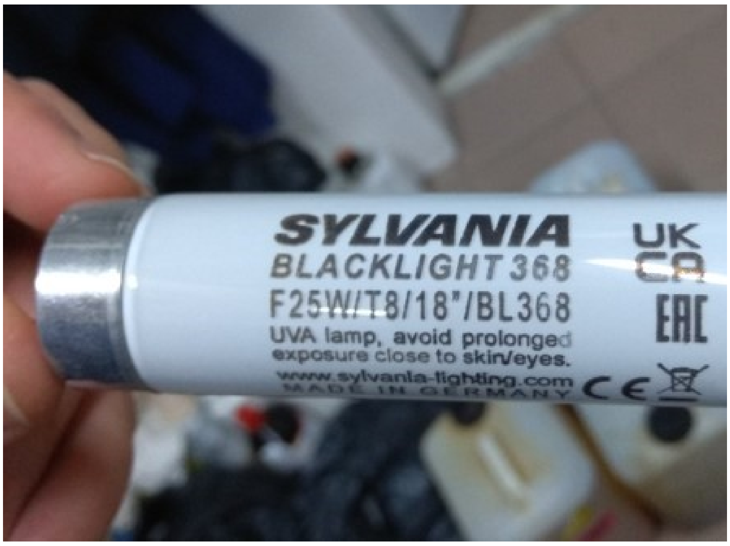

Type of lamp (368 nm)

Mark of lamp: 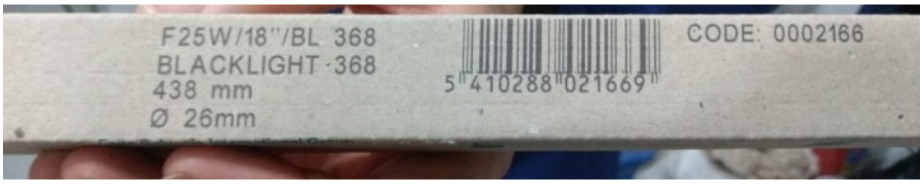

Illustration of the experimental set up of a photochemical step.

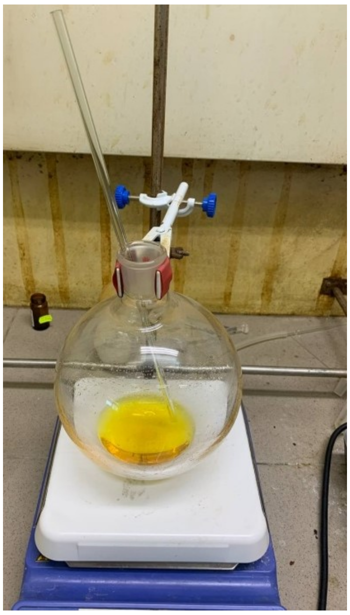
A flask with the diene

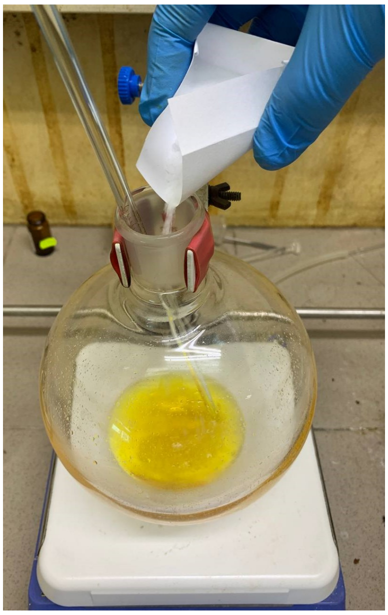
Addition of benzophenone

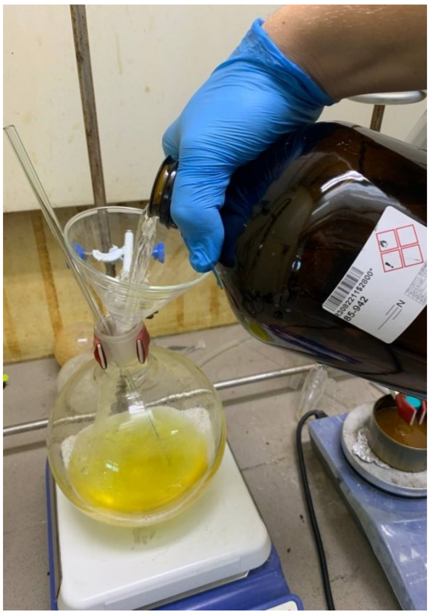
Dissolving in dry CH_3CN

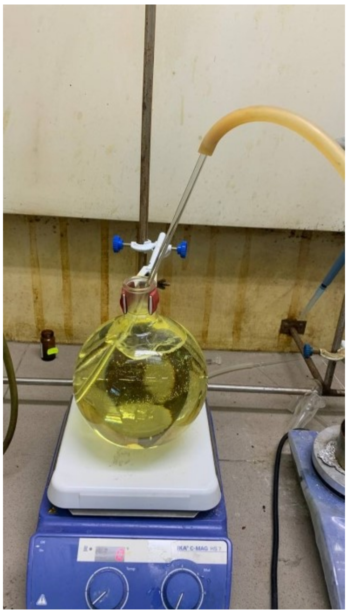
Degassing by bubbling of argon

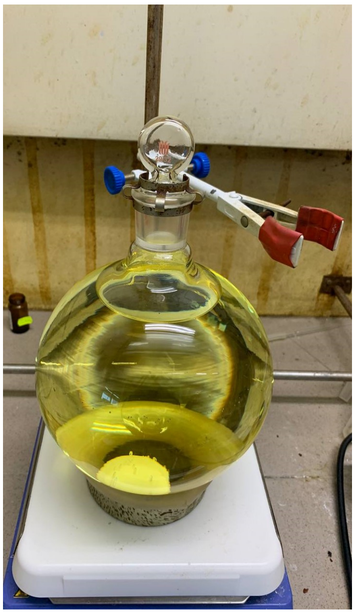
A reaction mixture

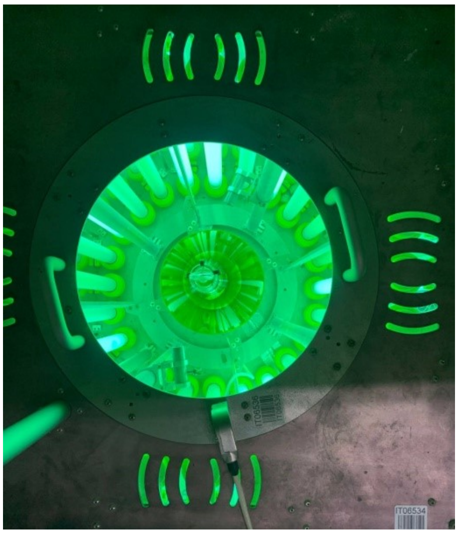
Irradiation with light

General procedure D (1b as an example)

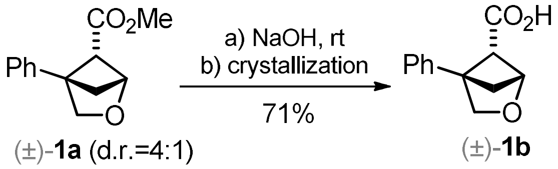

(±)-4-Phenyl-2-oxabicyclo[2.1.1]hexane-5-carboxylic acid (1b)

To a cold solution of NaOH (4.96 g, 0.124 mol, 2.00 equiv) in 100 mL of EtOH/H_2O (85/15; v/v) was added a solution of crude **1a** (13.52 g, 0.062 mol, 1.00 equiv) obtained in a previous step in EtOH (100 mL). The reaction mixture was stirred at room temperature for 12 h, and then the solvents were removed under reduced pressure. The residue was dissolved in 100 mL of water and washed with CH_2Cl_2 (2 × 50 mL). An aqueous layer was acidified with concentrated HCl to pH ~ 2 and extracted with CH_2Cl_2 (3 × 100 mL). The organic layers were combined, dried over Na_2SO_4 and evaporated to dryness. The crude product was recrystallized from a hexane-MeO*t*Bu mixture to obtain the pure product as a single stereoisomer. Yield: 8.98 g, 0.044 mol, 71%, beige solid, m.p. = 137-138 °C. ^{1}H NMR (400 MHz, $CDCl_3$): δ 9.86 (br s, 1H), 7.44 – 7.24 (m, *J* = 22.2, 16.4, 7.9 Hz, 5H), 4.88 (s, 1H), 4.32 (d, *J* = 6.1 Hz, 1H), 3.87 (d, *J* = 6.1 Hz, 1H), 2.95 (s, 1H), 2.02 (d, *J* = 7.7 Hz, 1H), 1.94 (d, *J* = 7.7 Hz, 1H) ppm. ^{13}C{^{1}H} NMR (126 MHz, $CDCl_3$): δ 136.7, 128.7, 127.8, 127.0, 79.4, 69.7, 57.3, 54.6, 42.8 ppm. LCMS $(M-H)^-$: 203. HRMS (ESI-TOF) *m/z*: $[M - H]^-$ calcd for $C_{12}H_{11}O_3$, 203.0708; found 203.0705.

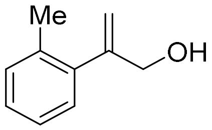

2-(*o*-Tolyl)prop-2-en-1-ol

General procedure A was used. Yield: 5.77 g, 0.039 mol, 78%, yellow oil, b.p. = 84-86 °C, 1 mmHg. ^{1}H NMR (400 MHz, $CDCl_3$): δ 7.24 – 7.11 (m, 4H), 5.51 (d, *J* = 1.4 Hz, 1H), 5.07 (s, 1H), 4.33 (s, 2H), 2.34 (s, 3H) ppm. ^{13}C{^{1}H} NMR (126 MHz, $CDCl_3$): δ 149.1, 139.8, 135.6, 130.4, 128.8, 127.6, 125.7, 113.3, 66.2, 19.9 ppm. GCMS (M): 148. HRMS (ESI-TOF) *m/z*: $[M + H]^+$ calcd for $C_{10}H_{13}O$, 149.0966; found 149.0961.

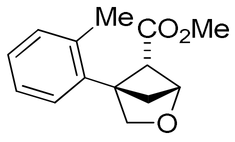

(±)

(±)-Methyl-4-(*o*-tolyl)-2-oxabicyclo[2.1.1]hexane-5-carboxylate (5a)

General procedure C was used to provide a crude mixture of products (d.r. = 7:2) with benzophenone. An analytically pure sample of the product was obtained by purification of the sample of the crude mixture by HPLC: Rt = 2-10 min, water/acetonitrile, 30-55%, flow 30 mL/min (loading pump 4 mL/min). Colorless oil, mixture of diastereomers d.r. ~ 5:1. The major one: ^{1}H NMR (500 MHz, $CDCl_3$): δ 7.49 – 7.42 (m, 1H), 7.20 – 7.12 (m, 3H), 4.80 (br s, 1H), 4.26 – 4.18 (m, 1H), 4.09 – 4.01 (m, 1H), 3.82 – 3.74 (m, 3H), 3.01 (s, 1H), 2.39 – 2.34 (m, 3H), 2.18 – 2.11 (m, 1H), 2.03 – 1.92 (m, 1H) ppm. ^{13}C{^{1}H} NMR (126 MHz, $CDCl_3$): δ 169.5, 136.0, 135.5, 131.1, 128.2, 127.4, 126.1, 78.7, 67.4, 58.6, 54.1, 51.6, 43.8, 20.3 ppm. LCMS $(M+H)^+$: 233. HRMS (ESI-TOF) *m/z*: $[M + H]^+$ calcd for $C_{14}H_{17}O_3$, 233.1178; found 233.1173.

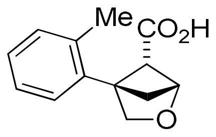

(±)

(±)-4-(*o*-Tolyl)-2-oxabicyclo[2.1.1]hexane-5-carboxylic acid (5b)

General procedure D was used. Product was isolated by column chromatography (gradient, petroleum ether/EtOAc, 9:1→ 7:3). Yield over 3 steps: 4.59 g, 0.021 mol, 65%, white solid, m.p. = 105-106 °C, single stereoisomer. ^{1}H NMR (400 MHz, $CDCl_3$): δ 10.92 (br s, 1H), 7.47 – 7.40 (m, 1H), 7.24 – 7.10 (m, 3H), 4.88 (s, 1H), 4.20 (d, *J* = 6.2 Hz, 1H), 4.09 (d, *J* = 6.2 Hz, 1H), 3.06 (s, 1H), 2.35 (s, 3H), 2.18 (d, *J* = 7.8 Hz, 1H), 2.03 (d, *J* = 7.8 Hz, 1H) ppm. ^{13}C{^{1}H} NMR (126 MHz, $CDCl_3$): δ 174.3, 136.1, 135.1, 131.3, 128.2, 127.8, 126.3, 78.9, 67.7, 59.1, 54.0, 44.0, 20.5 ppm. LCMS $(M-H)^-$: 217. HRMS (ESI-TOF) *m/z*: $[M - H]^-$ calcd for $C_{13}H_{13}O_3$, 217.0865; found 217.0864.

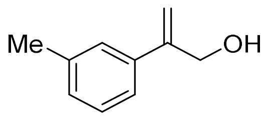

2-(*m*-Tolyl)prop-2-en-1-ol

General procedure A was used. Yield: 4.59 g, 0.031 mol, 62%, colorless oil, b.p. = 57-58 °C, 0.1 mmHg. ^{1}H NMR (400 MHz, $CDCl_3$): δ 7.32 – 7.23 (m, 3H), 7.15 (s, 1H), 5.47 (s, 1H), 5.35 (s, 1H), 4.56 (s, 2H), 2.39 (s, 3H) ppm. ^{13}C{^{1}H} NMR (151 MHz, $CDCl_3$): δ 147.6, 138.6, 138.2,

128.8, 128.6, 127.0, 123.3, 112.6, 65.2, 21.6 ppm. GCMS (M): 148. HRMS (ESI-TOF) *m/z*: [M + H]$^+$ calcd for $C_{10}H_{13}O$, 149.0966; found 149.0960.

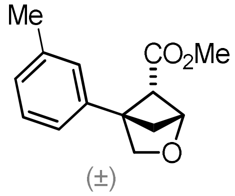

(±)-Methyl-4-(*m*-tolyl)-2-oxabicyclo[2.1.1]hexane-5-carboxylate (6a)

General procedure C was used to provide a crude mixture of products (d.r. = 4:1) with benzophenone. An analytically pure sample of the product was obtained by purification of the sample of the crude mixture by HPLC: Rt = 2-10 min, water/acetonitrile, 30-55%, flow 30 mL/min (loading pump 4 mL/min). Colorless oil, single major stereoisomer. ^{1}H NMR (400 MHz, $CDCl_3$): δ 7.26 – 7.16 (m, 3H), 7.09 (d, *J* = 7.1 Hz, 1H), 4.79 (s, 1H), 4.29 (d, *J* = 5.9 Hz, 1H), 3.79 (d, *J* = 5.9 Hz, 1H), 3.69 (s, 3H), 2.86 (s, 1H), 2.34 (s, 3H), 1.95 (d, *J* = 7.6 Hz, 1H), 1.87 (d, *J* = 8.4 Hz, 1H) ppm. $^{13}C\{^1H\}$ NMR (126 MHz, $CDCl_3$): δ 169.5, 138.3, 137.1, 128.6, 128.4, 127.7, 124.1, 79.4, 69.7, 57.1, 54.8, 51.7, 42.7, 21.6 ppm. LCMS (M+H)$^+$: 233. HRMS (ESI-TOF) *m/z*: [M + H]$^+$ calcd for $C_{14}H_{17}O_3$, 233.1178; found 233.1171.

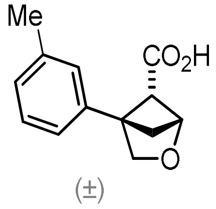

(±)-4-(*m*-Tolyl)-2-oxabicyclo[2.1.1]hexane-5-carboxylic acid (6b)

General procedure D was used. Product was isolated by column chromatography (gradient, petroleum ether/EtOAc, 9:1→ 7:3). Yield over 3 steps: 0.12 g, 0.55 mmol, 61%, yellow solid, m.p. = 99-100 °C, single stereoisomer. ^{1}H NMR (400 MHz, DMSO-d_6): δ 12.32 (s, 1H), 7.21 (br s, 3H), 7.11 – 7.04 (m, 1H), 4.69 (s, 1H), 4.05 (d, *J* = 5.3 Hz, 1H), 3.73 (d, *J* = 5.4 Hz, 1H), 3.04 (s, 1H), 2.29 (s, 3H), 1.94 (d, *J* = 7.2 Hz, 1H), 1.69 (d, *J* = 7.3 Hz, 1H) ppm. $^{13}C\{^1H\}$ NMR (126 MHz, DMSO-d_6): δ 170.5, 137.6, 137.3, 128.1, 127.7, 127.6, 124.2, 78.7, 68.3, 55.8, 54.1, 42.2, 21.1 ppm. LCMS (M-H)$^-$: 217. HRMS (ESI-TOF) *m/z*: [M - H]$^-$ calcd for $C_{13}H_{13}O_3$, 217.0865; found 217.0863.

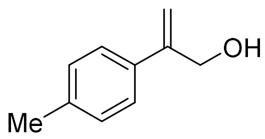

2-(*p*-Tolyl)prop-2-en-1-ol

General procedure A was used. Yield: 5.18 g, 0.035 mol, 70%, colorless oil, b.p. = 101-102 °C, 5 mmHg. ^{1}H NMR (400 MHz, $CDCl_3$): δ 7.36 (d, *J* = 8.1 Hz, 2H), 7.17 (d, *J* = 8.1 Hz, 2H), 5.44 (s, 1H), 5.31 (d, *J* = 1.0 Hz, 1H), 4.53 (s, 2H), 2.36 (s, 3H) ppm. ^{13}C{^{1}H} NMR (126 MHz, $CDCl_3$): δ 147.2, 137.9, 135.7, 129.3, 126.1, 111.9, 65.2, 21.3 ppm. LCMS $(M+H)^{+}$: 149. HRMS (ESI-TOF) *m/z*: $[M + H]^+$ calcd for $C_{10}H_{13}O$, 149.0966; found 149.0959.

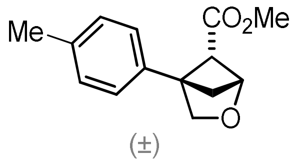

(±)-Methyl-4-(*p*-tolyl)-2-oxabicyclo[2.1.1]hexane-5-carboxylate (7a)

General procedure C was used to provide a crude mixture of products (d.r. = 4:1) with benzophenone. An analytically pure sample of the product was obtained by purification of the sample of the crude mixture by HPLC: Rt = 2-10 min, water/acetonitrile, 30-55%, flow 30 mL/min (loading pump 4 mL/min). Colorless oil, mixture of diastereomers ~ 3:2. ^{1}H NMR (400 MHz, $CDCl_3$): δ 7.35 – 7.06 (m, 4H), 4.80, 4.75 (2×s, 1H), (4.29 (d, *J* = 5.7 Hz), 4.01 (d, *J* = 6.1 Hz), 3.83 (d, *J* = 6.2 Hz), 3.80 (d, *J* = 5.9 Hz), 2H), 3.70, 3.56 (2×s, 3H), (3.20 (d, *J* = 8.0 Hz), 3.15 (d, *J* = 8.1 Hz), 1H), 2.35, 2.34 (2×s, 3H), (2.07 (t, *J* = 8.1 Hz), 1.96 (d, *J* = 7.6 Hz), 1.88 (d, *J* = 7.5 Hz), 2H) ppm. ^{13}C{^{1}H} NMR (151 MHz, $CDCl_3$): δ 171.2, 169.6, 137.4, 137.3, 134.1, 133.1, 129.3, 129.3, 127.0, 126.5, 79.7, 79.4, 74.6, 69.7, 60.1, 58.6, 56.9, 54.9, 51.8, 51.7, 42.7, 41.3, 21.3, 21.3 ppm. HRMS (ESI-TOF) *m/z*: $[M + H]^+$ calcd for $C_{14}H_{17}O_3$, 233.1178; found 233.1162.

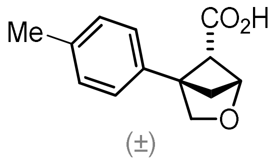

(±)-4-(*p*-Tolyl)-2-oxabicyclo[2.1.1]hexane-5-carboxylic acid (7b)

General procedure D was used. Product was isolated by column chromatography (gradient, petroleum ether/EtOAc, 9:1→ 7:3). Yield over 3 steps: 4.45 g, 0.02 mol, 59%, yellow solid, m.p. = 97-98 °C, single stereoisomer. ^{1}H NMR (400 MHz, DMSO-d_6): δ 12.31 (s, 1H), 7.29 (d, *J* = 7.9 Hz, 2H), 7.14 (d, *J* = 7.6 Hz, 2H), 4.68 (s, 1H), 4.04 (d, *J* = 5.4 Hz, 1H), 3.71 (d, *J* = 5.5 Hz,

1H), 3.01 (s, 1H), 2.28 (s, 3H), 1.92 (d, *J* = 7.0 Hz, 1H), 1.67 (d, *J* = 7.1 Hz, 1H) ppm. $^{13}C\{^1H\}$ NMR (126 MHz, DMSO-d_6): δ 170.5, 136.2, 134.6, 128.8, 127.0, 78.7, 68.3, 55.6, 54.2, 42.1, 20.7 ppm. LCMS $(M-H)^-$: 217. HRMS (ESI-TOF) *m/z*: $[M - H]^-$ calcd for $C_{13}H_{13}O_3$, 217.0865; found 217.0866.

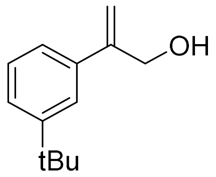

2-(3-(Tert-butyl)phenyl)prop-2-en-1-ol

General procedure A was used. Yield: 5.99 g, 0.0312 mol, 63%, colorless oil, b.p. = 90-91 °C, 0.1 mmHg. ^1H NMR (400 MHz, $CDCl_3$): δ 7.49 (s, 1H), 7.41 – 7.26 (m, 3H), 5.48 (s, 1H), 5.37 (s, 1H), 4.58 (s, 2H), 1.36 (s, 9H) ppm. $^{13}C\{^1H\}$ NMR (151 MHz, $CDCl_3$): δ 151.5, 148.1, 138.4, 128.3, 125.2, 123.4, 123.3, 112.5, 65.3, 34.9, 31.5 ppm. GCMS (M): 190. HRMS (ESI-TOF) *m/z*: $[M + H]^+$ calcd for $C_{13}H_{19}O$, 191.1436; found 191.1431.

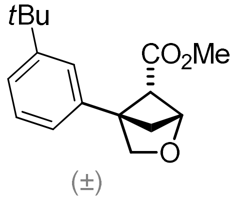

(±)-Methyl-4-(3-(tert-butyl)phenyl)-2-oxabicyclo[2.1.1]hexane-5-carboxylate (8a)

General procedure C was used to provide a crude mixture of products (d.r. = 3:1) with benzophenone. An analytically pure sample of the product was obtained by purification of the sample of the crude mixture by HPLC: Rt = 2-10 min, water/acetonitrile, 40-60%, flow 30 mL/min (loading pump 4 mL/min). Colorless oil, single major stereoisomer. ^1H NMR (400 MHz, $CDCl_3$): δ 7.46 (s, 1H), 7.36 – 7.27 (m, 2H), 7.22 (d, *J* = 7.0 Hz, 1H), 4.82 (s, 1H), 4.31 (d, *J* = 5.9 Hz, 1H), 3.82 (d, *J* = 5.9 Hz, 1H), 3.72 (s, 3H), 2.89 (s, 1H), 1.99 (d, *J* = 7.6 Hz, 1H), 1.92 (d, *J* = 7.6 Hz, 1H), 1.33 (s, 9H) ppm. $^{13}C\{^1H\}$ NMR (126 MHz, $CDCl_3$): δ 169.6, 151.5, 136.8, 128.3, 124.7, 124.1, 123.9, 79.3, 69.9, 57.5, 54.9, 51.7, 42.5, 34.9, 31.5 ppm. LCMS $(M+H)^+$: 275. HRMS (ESI-TOF) *m/z*: $[M + H]^+$ calcd for $C_{17}H_{23}O_3$, 275.1647; found 275.1644.

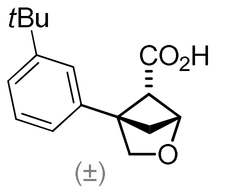

(±)-4-(3-(Tert-butyl)phenyl)-2-oxabicyclo[2.1.1]hexane-5-carboxylic acid (8b)

General procedure D was used. Product was isolated by crystallization from a hexane-MeO*t*Bu mixture. Yield over 3 steps: 6.71 g, 0.026 mol, 53%, yellow solid, m.p. = 129-130 °C, single stereoisomer. ^{1}H NMR (400 MHz, DMSO-d$_6$): δ 12.33 (s, 1H), 7.46 (s, 1H), 7.32 – 7.17 (m, 3H), 4.70 (s, 1H), 4.05 (d, *J* = 5.4 Hz, 1H), 3.73 (d, *J* = 5.4 Hz, 1H), 3.05 (s, 1H), 1.95 (d, *J* = 7.2 Hz, 1H), 1.73 (d, *J* = 7.3 Hz, 1H), 1.27 (s, 9H) ppm. ^{13}C{^{1}H} NMR (126 MHz, DMSO-d$_6$): δ 170.6, 150.5, 137.3, 127.9, 124.2, 123.9, 123.7, 78.7, 68.6, 56.1, 54.2, 42.0, 34.4, 31.2 ppm. LCMS (M-H)$^-$: 259. HRMS (ESI-TOF) *m/z*: [M - H]$^-$ calcd for $C_{16}H_{19}O_3$, 259.1334; found 259.1334.

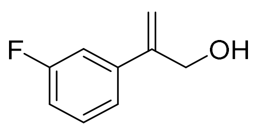

2-(3-Fluorophenyl)prop-2-en-1-ol

General procedure A was used. Yield: 5.55 g, 0.0365 mol, 73%, ca. 90% purity, colorless oil, b.p. = 88-89 °C, 1 mmHg. ^{1}H NMR (400 MHz, CDCl$_3$): δ 7.37 – 7.29 (m, 1H), 7.27 (d, *J* = 7.3 Hz, 1H), 7.18 (d, *J* = 10.4 Hz, 1H), 7.02 (t, *J* = 7.6 Hz, 1H), 5.52 (s, 1H), 5.42 (s, 1H), 4.54 (s, 2H) ppm. ^{13}C{^{1}H} NMR (151 MHz, CDCl$_3$): δ 163.1 (d, *J* = 246 Hz), 146.3, 140.9 (d, *J* = 8 Hz), 130.1 (d, *J* = 8 Hz), 121.8 (d, *J* = 3 Hz), 114.9 (d, *J* = 21 Hz), 114.0, 113.2 (d, *J* = 22 Hz), 65.0 ppm. ^{19}F{^{1}H} NMR (376 MHz, CDCl$_3$): δ -113.6 (s) ppm. GCMS (M): 152. HRMS (ESI-TOF) *m/z*: [M + H]$^+$ calcd for $C_9H_{10}FO$, 153.0716; found 153.0709.

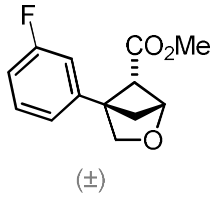

(±)-Methyl-4-(3-fluorophenyl)-2-oxabicyclo[2.1.1]hexane-5-carboxylate (9a)

General procedure C was used to provide a crude mixture of products (d.r. = 4:1) with benzophenone. An analytically pure sample of the product was obtained by HPLC: Rt = 2-10 min, water/acetonitrile, 20-45%, flow 30 mL/min (loading pump 4 mL/min). Colorless oil, mixture of diastereomers d.r. ~ 4:1. The major one: ^{1}H NMR (500 MHz, CDCl$_3$): δ 7.31 – 6.86 (m, 5H), 4.75 (s, 1H), 4.22 (d, *J* = 5.8 Hz, 1H), 3.74 (d, *J* = 5.9 Hz, 1H), 3.65 (s, 3H), 2.84 (s,

1H), 1.92 (d, *J* = 7.5 Hz, 1H), 1.84 (d, *J* = 7.6 Hz, 1H) ppm. $^{13}C\{^1H\}$ NMR (126 MHz, $CDCl_3$): δ 169.1, 162.8 (d, *J* = 246 Hz), 139.6 (d, *J* = 8 Hz), 130.0 (d, *J* = 8 Hz), 122.6 (d, *J* = 3 Hz), 114.3 (d, *J* = 21 Hz), 114.0 (d, *J* = 22 Hz), 79.1, 69.3, 59.8, 54.7, 51.4, 42.3 ppm. $^{19}F\{^1H\}$ NMR (376 MHz, $CDCl_3$): δ -113.3 (s), -113.4 (s) ppm. HRMS (ESI-TOF) *m/z*: $[M + H]^+$ calcd for $C_{13}H_{14}FO_3$, 237.0927; found 237.0920.

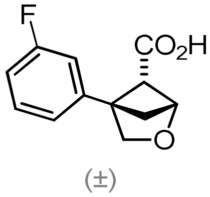

(±)-4-(3-Fluorophenyl)-2-oxabicyclo[2.1.1]hexane-5-carboxylic acid (9b)

General procedure D was used. Product was isolated by column chromatography (gradient, petroleum ether/EtOAc, 9:1→ 7:3). Yield over 3 steps: 5.26 g, 0.024 mol, 44%, yellow solid, m.p. = 121-122 °C, single stereoisomer ^1H NMR (400 MHz, $CDCl_3$): δ 10.75 (br s, 1H), 7.32 (dd, *J* = 14.0, 7.8 Hz, 1H), 7.17 (d, *J* = 7.7 Hz, 1H), 7.12 (d, *J* = 9.7 Hz, 1H), 6.99 (td, *J* = 8.4, 2.1 Hz, 1H), 4.88 (s, 1H), 4.30 (d, *J* = 6.1 Hz, 1H), 3.84 (d, *J* = 6.1 Hz, 1H), 2.95 (s, 1H), 2.01 (d, *J* = 7.7 Hz, 1H), 1.94 (d, *J* = 7.8 Hz, 1H) ppm. $^{13}C\{^1H\}$ NMR (126 MHz, $CDCl_3$): δ 174.2, 163.0 (d, *J* = 247 Hz), 139.3 (d, *J* = 7 Hz), 130.3 (d, *J* = 8 Hz), 122.7 (d, *J* = 3 Hz), 114.8 (d, *J* = 21 Hz), 114.2 (d, *J* = 22 Hz), 79.5, 69.6, 56.8, 54.7, 42.7 ppm. $^{19}F\{^1H\}$ NMR (376 MHz, $CDCl_3$): δ -113.1 (s) ppm. LCMS $(M-H)^-$: 221. HRMS (ESI-TOF) *m/z*: $[M - H]^-$ calcd for $C_{12}H_{10}FO_3$, 221.0614; found 221.0609.

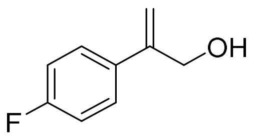

2-(4-Fluorophenyl)prop-2-en-1-ol

General procedure A was used. Yield: 5.24 g, 0.0345 mol, 69%, ca. 90% purity, yellow oil, b.p. = 90-91 °C, 1 mmHg. ^1H NMR (400 MHz, $CDCl_3$): δ 7.43 (dd, *J* = 8.7, 5.4 Hz, 2H), 7.04 (t, *J* = 8.7 Hz, 2H), 5.42 (s, 1H), 5.34 (s, 1H), 4.52 (s, 2H) ppm. $^{13}C\{^1H\}$ NMR (151 MHz, $CDCl_3$): δ 162.7 (d, *J* = 247 Hz), 146.4, 134.7 (d, *J* = 3 Hz), 127.9 (d, *J* = 8 Hz), 115.5 (d, *J* = 21 Hz), 112.9 (d, *J* = 1 Hz), 65.3 ppm. $^{19}F\{^1H\}$ NMR (376 MHz, $CDCl_3$): δ -114.8 (s) ppm. GCMS (M): 152. HRMS (ESI-TOF) *m/z*: $[M + H]^+$ calcd for $C_9H_{10}FO$, 153.0716; found 153.0707.

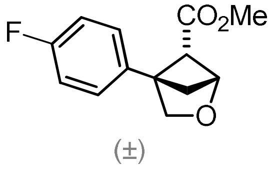

(±)-Methyl-4-(4-fluorophenyl)-2-oxabicyclo[2.1.1]hexane-5-carboxylate (10a)

General procedure C was used to provide a crude mixture of products (d.r. = 4:1) with benzophenone. An analytically pure sample of the product was obtained by HPLC: Rt = 2-10 min, water/acetonitrile, 20-45%, flow 30 mL/min (loading pump 4 mL/min). Colorless oil, single stereoisomer. ^{1}H NMR (400 MHz, $CDCl_3$): δ 7.39 (dd, *J* = 8.7, 5.4 Hz, 2H), 7.03 (t, *J* = 8.7 Hz, 2H), 4.81 (s, 1H), 4.26 (d, *J* = 6.0 Hz, 1H), 3.77 (d, *J* = 6.0 Hz, 1H), 3.71 (s, 3H), 2.85 (s, 1H), 1.96 (d, *J* = 7.6 Hz, 1H), 1.89 (dd, *J* = 7.7, 0.9 Hz, 1H) ppm. $^{13}C\{^{1}H\}$ NMR (126 MHz, $CDCl_3$): δ 169.5, 162.3 (d, *J* = 246.1 Hz), 132.9 (d, *J* = 3.1 Hz), 128.8 (d, *J* = 8.2 Hz), 115.5 (d, *J* = 21.3 Hz), 79.3, 69.8, 56.4, 55.0, 51.7, 42.5 ppm. $^{19}F\{^{1}H\}$ NMR (376 MHz, $CDCl_3$): δ -115.3 (s) ppm. HRMS (ESI-TOF) *m/z*: $[M + H]^{+}$ calcd for $C_{13}H_{14}FO_3$, 237.0927; found 237.0926.

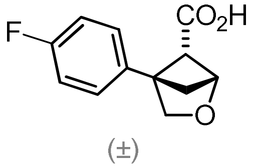

(±)-4-(4-Fluorophenyl)-2-oxabicyclo[2.1.1]hexane-5-carboxylic acid (10b)

General procedure D was used. Product was isolated by column chromatography (gradient, petroleum ether/EtOAc, 9:1→ 7:3). Yield over 3 steps: 11.05 g, 0.05 mol, 71%, white solid, mixture of diastereomers d.r. ~ 5:1. The major one: ^{1}H NMR (400 MHz, $CDCl_3$): δ 10.25 (br s, 1H), 7.36 (dd, *J* = 8.1, 5.5 Hz, 2H), 7.03 (t, *J* = 8.5 Hz, 2H), 4.86 (s, 1H), 4.27 (d, *J* = 6.1 Hz, 1H), 3.81 (d, *J* = 6.1 Hz, 1H), 2.90 (s, 1H), 1.99 (d, *J* = 7.7 Hz, 1H), 1.91 (d, *J* = 7.7 Hz, 1H) ppm. $^{13}C\{^{1}H\}$ NMR (101 MHz, $CDCl_3$): δ 174.4, 162.3 (d, *J* = 246.3 Hz), 132.5, 128.7 (d, *J* = 8.1 Hz), 115.6 (d, *J* = 21.4 Hz), 79.4, 69.7, 56.6, 54.7, 42.7 ppm. $^{19}F\{^{1}H\}$ NMR (376 MHz, $CDCl_3$): δ -114.9 (s) ppm. HRMS (ESI-TOF) *m/z*: $[M + H]^{+}$ calcd for $C_{12}H_{12}FO_3$, 223.0770; found 223.0768.

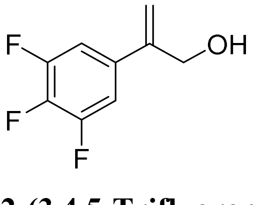

2-(3,4,5-Trifluorophenyl)prop-2-en-1-ol

General procedure A was used. Yield: 4.18 g, 0.0275 mol, 55%, white solid, m.p. = 48-49 °C, b.p. = 92-93 °C, 1 mmHg. ^{1}H NMR (400 MHz, DMSO-d$_6$): δ 7.45 (t, *J* = 8.0 Hz, 1H), 5.59 (s, 1H), 5.40 (s, 1H), 5.14 (t, *J* = 5.3 Hz, 1H), 4.29 (d, *J* = 5.1 Hz, 2H) ppm. ^{13}C{^{1}H} NMR (126 MHz, DMSO-d$_6$): δ 150.2 (dd, *J* = 245.9, 4.1 Hz), 150.1 (dd, *J* = 245.9, 4.1 Hz), 144.7, 138.1 (dt, *J* = 248.7, 15.6 Hz), 135.5 (m), 114.1, 110.5 (d, *J* = 4.6 Hz), 110.4 (d, *J* = 4.4 Hz), 62.3 ppm. ^{19}F{^{1}H} NMR (376 MHz, DMSO-d$_6$): δ -136.3(d, *J* = 21.7 Hz), -163.6(t, *J* = 21.8 Hz) ppm. HRMS (ESI-TOF) *m/z*: [M + H]$^+$ calcd for $C_9H_{10}FO$, 153.0716; found 153.0707.

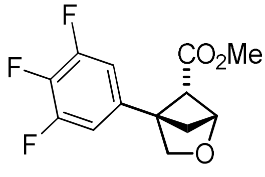

(±)-Methyl-4-(3,4,5-trifluorophenyl)-2-oxabicyclo[2.1.1]hexane-5-carboxylate (11a)

General procedure C was used to provide a crude mixture of products (d.r. = 4:1) with benzophenone. An analytically pure sample of the product was obtained by purification of the sample of the crude mixture by HPLC: Rt = 2-10 min, water/acetonitrile, 20-45%, flow 30 mL/min (loading pump 4 mL/min). Colorless oil, mixture of diastereomers d.r. ~ 4:1. The major one: ^{1}H NMR (500 MHz, $CDCl_3$): δ 7.12 – 7.02 (m, 2H), 4.81 (s, 1H), 4.19 (d, *J* = 5.9 Hz, 1H), 3.73 (s, 3H), 3.61 (s, 1H), 2.84 (s, 1H), 1.92 (dd, *J* = 17.8, 7.6 Hz, 2H) ppm. ^{13}C{^{1}H} NMR (126 MHz, $CDCl_3$): δ 169.2, 151.4 (ddd, *J* = 250.4, 9.8, 4.0 Hz), 139.2 (dt, *J* = 251.5, 15.3 Hz), 133.6 – 133.4 (m), 111.5 (dd, *J* = 16.4, 5.1 Hz), 79.3, 69.6, 59.9, 55.1, 51.9, 42.2 ppm. ^{19}F{^{1}H} NMR (376 MHz, $CDCl_3$): δ -134.3 (d, *J* = 20.5 Hz), -134.4 (d, *J* = 20.8 Hz), -161.9 (t, *J* = 21.3 Hz), -162.1 (t, *J* = 20.8 Hz) ppm. LCMS (M+H)$^+$: 273. HRMS (ESI-TOF) *m/z*: [M + H]$^+$ calcd for $C_{13}H_{12}F_3O_3$, 273.0739; found 273.0737.

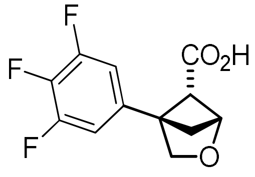

(±)-4-(3,4,5-Trifluorophenyl)-2-oxabicyclo[2.1.1]hexane-5-carboxylic acid (11b)

General procedure D was used. Product was isolated by crystallization from a hexane-MeO*t*Bu mixture. Yield over 3 steps: 5.49 g, 0.021 mol, 49%, yellow solid, m.p. = 113-114 °C, mixture of diastereomers d.r. ~ 9:1. The major one: ^{1}H NMR (400 MHz, DMSO-d$_6$): δ 12.52 (br s, 1H), 7.40 (dd, *J* = 9.0, 6.9 Hz, 2H), 4.70 (s, 1H), 3.96 (d, *J* = 5.6 Hz, 1H), 3.76 (d, *J* = 5.6 Hz, 1H), 3.15 (s,

1H), 1.96 (d, J = 7.4 Hz, 1H), 1.73 (d, J = 7.4 Hz, 1H) ppm. $^{13}C\{^1H\}$ NMR (126 MHz, DMSO-d_6): δ 170.5, 150.2 (dd, J = 246.9, 4.1 Hz), 150.1 (dd, J = 246.9, 3.7 Hz), 137.8 (dt, J = 248.2, 15.5 Hz), 135.2 – 134.9 (m), 112.2 (d, J = 4.6 Hz), 112.1 (d, J = 4.6 Hz), 78.6, 68.0, 54.9, 54.3, 42.1 ppm. $^{19}F\{^1H\}$ NMR (376 MHz, DMSO-d_6): δ -136.1 (d, J = 21.7 Hz), -164.0 (t, J = 21.7 Hz) ppm. LCMS $(M-H)^-$: 257. HRMS (ESI-TOF) *m/z*: $[M + H]^+$ calcd for $C_{12}H_{10}F_3O_3$, 259.0582; found 259.0577.

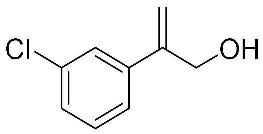

2-(3-Chlorophenyl)prop-2-en-1-ol

General procedure A was used. Yield: 5.21 g, 0.031 mol, 62%, ca. 90% purity, colorless oil, b.p. = 115-116 °C, 0.1 mmHg. ^1H NMR (400 MHz, $CDCl_3$): δ 7.45 (s, 1H), 7.39 – 7.27 (m, 3H), 5.50 (s, 1H), 5.41 (s, 1H), 4.53 (s, 2H) ppm. $^{13}C\{^1H\}$ NMR (126 MHz, $CDCl_3$): δ 146.2, 140.5, 134.6, 129.9, 128.1, 126.5, 124.4, 114.1, 65.0 ppm. GCMS (M): 168. HRMS (ESI-TOF) *m/z*: $[M + H]^+$ calcd for $C_9H_{10}ClO$, 169.0420; found 169.0414.

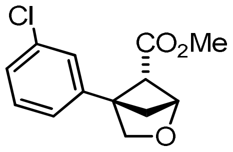

(±)

(±)-Methyl-4-(3-chlorophenyl)-2-oxabicyclo[2.1.1]hexane-5-carboxylate (12a)

General procedure C was used to provide a crude mixture of products (d.r. = 3:1) with benzophenone. An analytically pure sample of the product was obtained by purification of the sample of the crude mixture by HPLC: Rt = 2-10 min, water/acetonitrile, 30-55%, flow 30 mL/min (loading pump 4 mL/min). Colorless oil, mixture of diastereomers d.r. ~ 9:2. The major one: ^1H NMR (400 MHz, $CDCl_3$): δ 7.38 (s, 1H), 7.33 – 7.18 (m, 3H), 4.79 (s, 1H), 4.25 (d, J = 5.9 Hz, 1H), 3.77 (d, J = 6.0 Hz, 1H), 3.70 (s, 3H), 2.86 (s, 1H), 1.95 (d, J = 7.6 Hz, 1H), 1.88 (d, J = 7.7 Hz, 1H) ppm. $^{13}C\{^1H\}$ NMR (126 MHz, $CDCl_3$): δ 169.3, 139.2, 134.6, 129.9, 127.8, 127.4, 125.3, 79.4, 69.6, 56.6, 54.9, 51.8, 42.5 ppm. HRMS (ESI-TOF) *m/z*: $[M + H]^+$ calcd for $C_{13}H_{14}ClO_3$, 253.0631; found 253.0630.

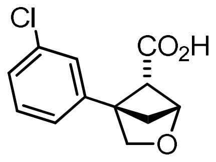

(±)

(±)-4-(3-Chlorophenyl)-2-oxabicyclo[2.1.1]hexane-5-carboxylic acid (12b)

General procedure D was used. Product was isolated by column chromatography (gradient, petroleum ether/EtOAc, 9:1→ 7:3). Yield over 3 steps: 10.08 g, 0.042 mol, 51%, yellow solid, m.p. = 109-110 °C, single stereoisomer. ^{1}H NMR (400 MHz, DMSO-d$_6$): δ 12.38 (s, 1H), 7.50 (s, 1H), 7.43 – 7.30 (m, 3H), 4.70 (s, 1H), 4.02 (d, *J* = 5.5 Hz, 1H), 3.76 (d, *J* = 5.5 Hz, 1H), 3.13 (s, 1H), 1.98 (d, *J* = 7.2 Hz, 1H), 1.74 (d, *J* = 7.3 Hz, 1H) ppm. ^{13}C{^{1}H} NMR (126 MHz, DMSO-d$_6$): δ 170.5, 140.2, 133.0, 130.1, 127.1, 127.1, 125.9, 78.7, 68.2, 55.4, 54.2, 42.0 ppm. LCMS (M-H)$^-$: 237. HRMS (ESI-TOF) *m/z*: [M + H]$^+$ calcd for $C_{12}H_{12}ClO_3$, 239.0475; found 239.0472.

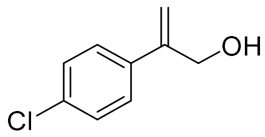

2-(4-Chlorophenyl)prop-2-en-1-ol

General procedure A was used. Yield: 5.29 g, 0.0315 mol, 63%, ca. 90% purity, colorless oil, b.p. = 114-115 °C, 0.1 mmHg. ^{1}H NMR (500 MHz, DMSO-d$_6$): δ 7.49 (d, *J* = 8.6 Hz, 2H), 7.39 (d, *J* = 8.6 Hz, 2H), 5.48 (s, 1H), 5.34 (d, *J* = 1.4 Hz, 1H), 5.08 (br s, 1H), 4.31 (s, 2H) ppm. ^{13}C{^{1}H} NMR (151 MHz, DMSO-d$_6$): δ 146.4, 137.5, 132.1, 128.2, 127.5, 112.0, 62.5 ppm. HRMS (ESI-TOF) *m/z*: [M + H]$^+$ calcd for $C_9H_{10}ClO$, 169.0420; found 169.0412.

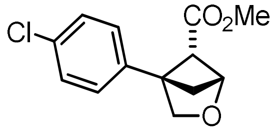

(±)

(±)-Methyl-4-(4-chlorophenyl)-2-oxabicyclo[2.1.1]hexane-5-carboxylate (13a)

General procedure C was used to provide a crude mixture of products (d.r. = 3:1) with benzophenone. An analytically pure sample of the product was obtained by purification of the sample of the crude mixture by HPLC: Rt = 2-10 min, water/acetonitrile, 40-60%, flow 30 mL/min (loading pump 4 mL/min). Colorless oil, mixture of diastereomers d.r. ~ 4:1. The major one: ^{1}H NMR (400 MHz, $CDCl_3$): δ 7.37 – 7.30 (m, 4H), 4.81 (s, 1H), 4.26 (d, *J* = 5.9 Hz, 1H), 3.78 (d, *J* = 6.0 Hz, 1H), 3.71 (s, 3H), 2.86 (s, 1H), 1.96 (d, *J* = 7.6 Hz, 1H), 1.89 (d, *J* = 7.7 Hz, 1H) ppm. ^{13}C{^{1}H} NMR (151 MHz, $CDCl_3$): δ 169.5, 135.7, 133.5, 128.8, 128.5, 79.4, 69.7,

56.5, 55.0, 51.8, 42.5 ppm. LCMS $(M+H)^+$: 253. HRMS (ESI-TOF) *m/z*: $[M + H]^+$ calcd for $C_{13}H_{14}ClO_3$, 253.0631; found 253.0625.

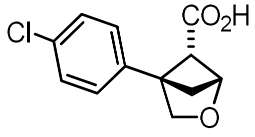

(±)

(±)-4-(4-Chlorophenyl)-2-oxabicyclo[2.1.1]hexane-5-carboxylic acid (13b)

General procedure D was used. Product was isolated by crystallization from a hexane-MeO*t*Bu mixture. Yield over 3 steps: 9.43 g, 0.0396 mol, 60%, yellow solid, m.p. = 77-78 °C. Mixture of diastereomers ~ 7:1. ^{1}H NMR (400 MHz, DMSO-d$_6$): δ 12.40 (s, 1H), 7.47 – 7.38 (m, 4H), 4.70 (s, 1H), 4.02 (d, *J* = 5.4 Hz, 1H), 3.74 (d, *J* = 5.4 Hz, 1H), 3.09 (s, 1H), 1.96 (d, *J* = 7.1 Hz, 1H), 1.71 (d, *J* = 7.2 Hz, 1H) ppm. ^{13}C{^{1}H} NMR (126 MHz, DMSO-d$_6$): δ 170.5, 136.7, 131.7, 129.1, 128.2, 78.7, 68.3, 55.2, 54.2, 42.0 ppm. LCMS $(M-H)^-$: 237. HRMS (ESI-TOF) *m/z*: $[M - H]^-$ calcd for $C_{12}H_{10}ClO_3$, 237.0318; found 237.0312.

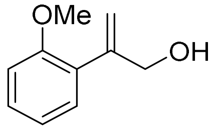

2-(2-Methoxyphenyl)prop-2-en-1-ol

General procedure A was used. Yield: 4.84 g, 0.0295 mol, 59%, yellow oil, b.p. = 50-52 °C, 0.1 mmHg. ^{1}H NMR (400 MHz, $CDCl_3$): δ 7.29 (t, *J* = 7.9 Hz, 1H), 7.05 (d, *J* = 7.7 Hz, 1H), 7.01 (s, 1H), 6.88 (dd, *J* = 8.2, 2.1 Hz, 1H), 5.49 (s, 1H), 5.37 (s, 1H), 4.54 (s, 2H), 3.84 (s, 3H) ppm. ^{13}C{^{1}H} NMR (126 MHz, $CDCl_3$): δ 159.8, 147.3, 140.2, 129.6, 118.7, 113.3, 113.0, 112.2, 65.2, 55.6, 55.4 ppm. GCMS (M): 164. HRMS (ESI-TOF) *m/z*: $[M + H]^+$ calcd for $C_{10}H_{13}O_2$, 165.0916; found 165.0907.

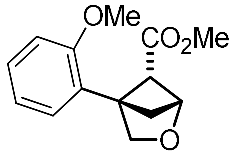

(±)

(±)-Methyl-4-(2-methoxyphenyl)-2-oxabicyclo[2.1.1]hexane-5-carboxylate (14a)

General procedure C was used to provide a crude mixture of products (d.r. = 4:1) with benzophenone. An analytically pure sample of the product was obtained by purification of the sample of the crude mixture by HPLC: Rt = 2-10 min, water/acetonitrile, 20-45%, flow 30

mL/min (loading pump 4 mL/min). Colorless oil, single major stereoisomer. ^{1}H NMR (400 MHz, $CDCl_3$): δ 7.34 – 7.24 (m, 2H), 6.99 – 6.91 (m, 1H), 6.88 (d, J = 8.2 Hz, 1H), 4.79 (s, 1H), 4.21 (d, J = 5.6 Hz, 1H), 4.06 (d, J = 5.6 Hz, 1H), 3.81 (s, 3H), 3.72 (s, 3H), 3.08 (s, 1H), 2.02 (s, 2H) ppm. ^{13}C{^{1}H} NMR (126 MHz, $CDCl_3$): δ 169.7, 158.3, 128.8, 128.7, 125.6, 120.7, 110.6, 79.4, 68.1, 55.8, 55.2, 53.3, 51.6, 43.7 ppm. LCMS $(M-H)^-$: 247. HRMS (ESI-TOF) *m/z*: $[M + H]^+$ calcd for $C_{14}H_{17}O_4$, 249.1127; found 249.1118.

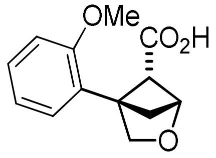

(±)

(±)-4-(2-Methoxyphenyl)-2-oxabicyclo[2.1.1]hexane-5-carboxylic acid (14b)

General procedure D was used. Product was isolated by column chromatography (gradient, petroleum ether/EtOAc, 9:1→ 7:3). Yield over 3 steps: 7.16 g, 0.031 mol, 59%, orange oil, single stereoisomer**.** ^{1}H NMR (400 MHz, DMSO-d_6): δ 12.26 (br s, 1H), 7.30 – 7.20 (m, 2H), 6.97 (d, J = 8.2 Hz, 1H), 6.90 (t, J = 7.4 Hz, 1H), 4.67 (s, 1H), 3.93 (s, 2H), 3.76 (s, 3H), 3.13 (s, 1H), 1.91 (d, J = 7.3 Hz, 1H), 1.79 (d, J = 7.3 Hz, 1H) ppm. ^{13}C{^{1}H} NMR (126 MHz, DMSO-d_6): δ 170.6, 157.9, 128.9, 128.4, 125.4, 120.1, 111.0, 78.7, 67.0, 55.3, 54.4, 52.9, 42.7 ppm. LCMS $(M-H)^-$: 233. HRMS (ESI-TOF) *m/z*: $[M - H]^-$ calcd for $C_{13}H_{13}O_4$, 233.0814; found 233.0807.

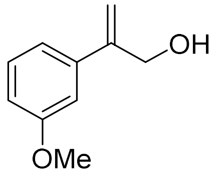

2-(3-Methoxyphenyl)prop-2-en-1-ol

General procedure A was used. Yield: 5.58 g, 0.034 mol, 68%, orange oil, b.p. = 64-65 °C, 0.1 mmHg. ^{1}H NMR (400 MHz, DMSO-d_6): δ 7.25 (t, J = 7.9 Hz, 1H), 7.02 (d, J = 7.7 Hz, 1H), 6.97 (s, 1H), 6.86 (dd, J = 8.2, 1.6 Hz, 1H), 5.44 (s, 1H), 5.31 (d, J = 1.5 Hz, 1H), 5.04 (t, J = 5.5 Hz, 1H), 4.30 (d, J = 5.5 Hz, 2H), 3.76 (s, 3H) ppm. ^{13}C{^{1}H} NMR (126 MHz, $CDCl_3$): δ 159.8, 147.3, 140.2, 129.6, 118.7, 113.3, 113.0, 112.2, 65.2, 55.4 ppm. GCMS (M): 164. HRMS (ESI-TOF) *m/z*: $[M + H]^+$ calcd for $C_{10}H_{13}O_2$, 165.0916; found 165.0905.

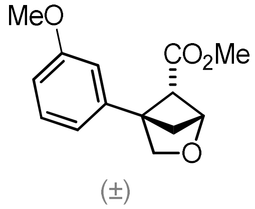

(±)-Methyl-4-(3-methoxyphenyl)-2-oxabicyclo[2.1.1]hexane-5-carboxylate (15a)

General procedure C was used to provide a crude mixture of products (d.r. = 3:1) with benzophenone. An analytically pure sample of the product was obtained by purification of the sample of the crude mixture by HPLC: Rt = 2-10 min, water/acetonitrile, 20-45%, flow 30 mL/min (loading pump 4 mL/min). Brown oil, single stereoisomer. ^{1}H NMR (400 MHz, DMSO-d_6): δ 7.26 (t, J = 7.9 Hz, 1H), 7.01 – 6.93 (m, 2H), 6.86 (dd, J = 8.2, 2.3 Hz, 1H), 4.72 (s, 1H), 4.00 (d, J = 5.6 Hz, 1H), 3.77 (d, J = 5.9 Hz, 1H), 3.75 (s, 3H), 3.59 (s, 3H), 3.31 (s, 1H), 3.18 (s, 1H), 2.00 (d, J = 7.3 Hz, 1H), 1.74 (d, J = 7.3 Hz, 1H) ppm. ^{13}C{^{1}H} NMR (126 MHz, DMSO-d_6): δ 169.2, 159.2, 138.8, 129.3, 119.2, 113.0, 112.5, 78.6, 68.2, 56.1, 55.0, 53.7, 51.1, 42.3 ppm. LCMS $(M+H)^+$: 249. HRMS (ESI-TOF) *m/z*: $[M + H]^+$ calcd for $C_{14}H_{17}O_4$, 249.1127; found 249.1118.

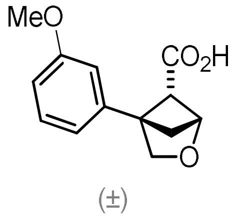

(±)-4-(3-Methoxyphenyl)-2-oxabicyclo[2.1.1]hexane-5-carboxylic acid (15b)

General procedure D was used. Product was isolated by column chromatography (gradient, petroleum ether/EtOAc, 9:1 → 7:3). Yield over 3 steps: 0.10 g, 0.43 mmol, 51%, colorless oil, single stereoisomer. ^{1}H NMR (400 MHz, DMSO-d_6): δ 12.35 (br s, 1H), 7.25 (t, J = 7.9 Hz, 1H), 7.01 – 6.94 (m, 2H), 6.84 (dd, J = 8.2, 1.8 Hz, 1H), 4.69 (s, 1H), 4.03 (d, J = 5.4 Hz, 1H), 3.74 (s, 3H), 3.73 (d, J = 5.9 Hz, 1H), 3.06 (s, 1H), 1.94 (d, J = 7.2 Hz, 1H), 1.71 (d, J = 7.3 Hz, 1H) ppm. ^{13}C{^{1}H} NMR (126 MHz, DMSO-d_6): δ 170.4, 159.2, 139.2, 129.3, 119.3, 113.1, 112.3, 78.6, 68.3, 55.8, 55.0, 54.1, 42.1 ppm. LCMS $(M-H)^-$: 233. HRMS (ESI-TOF) *m/z*: $[M - H]^-$ calcd for $C_{13}H_{13}O_4$, 233.0814; found 233.0814.

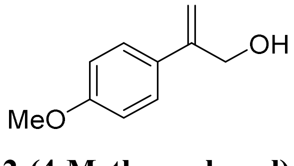

2-(4-Methoxyphenyl)prop-2-en-1-ol

General procedure A was used. Yield: 5.99 g, 0.0365 mol, 73%, yellow oil, b.p. = 77-78 °C, 0.1 mmHg. ^{1}H NMR (400 MHz, $CDCl_3$): δ 7.41 (d, *J* = 8.7 Hz, 2H), 6.89 (d, *J* = 8.7 Hz, 2H), 5.39 (s, 1H), 5.26 (s, 1H), 4.52 (s, 2H), 3.82 (s, 3H) ppm. $^{13}C\{^{1}H\}$ NMR (126 MHz, $CDCl_3$): δ 159.6, 146.7, 131.0, 127.3, 114.0, 111.2, 65.3, 55.4 ppm. LCMS $(M+H)^+$: 165. HRMS (ESI-TOF) *m/z*: $[M + H]^+$ calcd for $C_{10}H_{13}O_2$, 165.0916; found 165.0903.

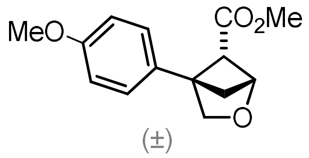

(±)-Methyl-4-(4-methoxyphenyl)-2-oxabicyclo[2.1.1]hexane-5-carboxylate (16a)

General procedure C was used to provide a crude mixture of products (d.r. = 4:1) with benzophenone. An analytically pure sample of the product was obtained by purification of the sample of the crude mixture by HPLC: Rt = 2-10 min, water/acetonitrile, 20-45%, flow 30 mL/min (loading pump 4 mL/min). Yellow oil, single stereoisomer. ^{1}H NMR (400 MHz, DMSO-d_6): δ 7.32 (d, *J* = 8.4 Hz, 2H), 6.91 (d, *J* = 8.4 Hz, 2H), 4.71 (s, 1H), 3.99 (d, *J* = 5.6 Hz, 1H), 3.74 (s, 3H), 3.72 (d, *J* = 5.9 Hz, 1H), 3.58 (s, 3H), 3.11 (s, 1H), 1.98 (d, *J* = 7.3 Hz, 1H), 1.68 (d, *J* = 7.4 Hz, 1H) ppm. $^{13}C\{^{1}H\}$ NMR (126 MHz, DMSO-d_6): δ 169.2, 158.4, 129.2, 128.1, 113.7, 78.5, 68.2, 55.6, 55.1, 53.8, 51.1, 42.3 ppm. LCMS $(M+H)^+$: 249. HRMS (ESI-TOF) *m/z*: $[M + H]^+$ calcd for $C_{14}H_{17}O_4$, 249.1127; found 249.1108.

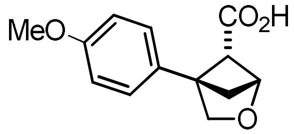

(±)-4-(4-Methoxyphenyl)-2-oxabicyclo[2.1.1]hexane-5-carboxylic acid (16b)

General procedure D was used. Product was isolated by crystallization from a hexane-MeO*t*Bu mixture. Yield over 3 steps: 4.53 g, 0.019 mol, 74%, yellow solid, m.p. = 113-114 °C, single stereoisomer. ^{1}H NMR (400 MHz, DMSO-d_6): δ 12.31 (s, 1H), 7.33 (d, *J* = 8.5 Hz, 2H), 6.90 (d, *J* = 8.5 Hz, 2H), 4.67 (s, 1H), 4.02 (d, *J* = 5.4 Hz, 1H), 3.73 (s, 3H), 3.69 (d, *J* = 5.4 Hz, 1H), 2.99 (s, 1H), 1.92 (d, *J* = 7.1 Hz, 1H), 1.66 (d, *J* = 7.3 Hz, 1H) ppm. $^{13}C\{^{1}H\}$ NMR (126 MHz, DMSO-d_6): δ 170.6, 158.4, 129.6, 128.2, 113.7, 78.6, 68.4, 55.3, 55.1, 54.3, 42.1 ppm. LCMS $(M-H)^-$: 233. HRMS (ESI-TOF) *m/z*: $[M + H]^+$ calcd for $C_{13}H_{15}O_4$, 235.0970; found 235.0961.

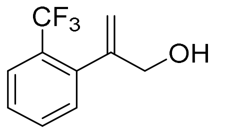

2-(2-(Trifluoromethyl)phenyl)prop-2-en-1-ol

General procedure A was used. Yield: 7.07 g, 0.035 mol, 70%, colorless oil, b.p. = 88-89 °C, 1 mmHg. ^{1}H NMR (400 MHz, $CDCl_3$): δ 7.68 (d, *J* = 7.8 Hz, 1H), 7.51 (t, *J* = 7.5 Hz, 1H), 7.41 (t, *J* = 7.6 Hz, 1H), 7.28 (d, *J* = 7.7 Hz, 1H), 5.54 (d, *J* = 1.3 Hz, 1H), 5.12 (s, 1H), 4.34 (s, 2H) ppm. $^{13}C\{^1H\}$ NMR (126 MHz, $CDCl_3$): δ 146.9, 139.2, 131.5, 131.0, 128.7 (q, *J* = 29.7 Hz), 127.7, 126.3 (q, *J* = 5.2 Hz), 124.3 (q, *J* = 273.5 Hz), 115.0, 66.7 ppm. $^{19}F\{^1H\}$ NMR (376 MHz, $CDCl_3$): δ -58.5 (s) ppm. HRMS (ESI-TOF) *m/z*: $[M + H]^+$ calcd for $C_{10}H_{10}F_3O$, 203.0684; found 203.0687.

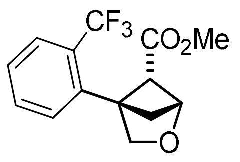

(±)

(±)-Methyl-4-(2-(trifluoromethyl)phenyl)-2-oxabicyclo[2.1.1]hexane-5-carboxylate (17a)

General procedure C was used to provide a crude mixture of products (d.r. = 7:3) with benzophenone. An analytically pure sample of the product was obtained by purification of the sample of the crude mixture by HPLC: Rt = 2-10 min, water/acetonitrile, 40-60%, flow 30 mL/min (loading pump 4 mL/min). Yellow solid, m.p. = 65-67 °C, mixture of diastereomers ~ 4:1. The major one: ^{1}H NMR (400 MHz, DMSO-d_6): δ 7.83 (d, *J* = 7.9 Hz, 1H), 7.76 (d, *J* = 7.8 Hz, 1H), 7.67 (t, *J* = 7.5 Hz, 1H), 7.53 (t, *J* = 7.9 Hz, 1H), 4.72 (s, 1H), 4.00 (d, *J* = 5.6 Hz, 1H), 3.82 (d, *J* = 5.5 Hz, 1H), 3.67 (s, 3H), 3.39 (s, 1H), 2.11 (d, *J* = 7.5 Hz, 1H), 1.92 (d, *J* = 6.9 Hz, 1H) ppm. $^{13}C\{^1H\}$ NMR (151 MHz, DMSO-d_6): δ 169.4, 135.9, 132.6, 130.9, 127.9, 126.6 (m), 124.2 (q, *J* = 273.1 Hz), 78.0, 68.4 (q, *J* = 3.2 Hz), 56.5, 54.4, 51.4, 43.5 (q, *J* = 4.4 Hz) ppm. $^{19}F\{^1H\}$ NMR (376 MHz, DMSO-d_6): δ -57.9 (s) ppm. HRMS (ESI-TOF) *m/z*: $[M + H]^+$ calcd for $C_{14}H_{14}F_3O_3$, 287.0895; found 287.0895.

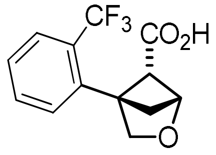

(±)

(±)-4-(2-(Trifluoromethyl)phenyl)-2-oxabicyclo[2.1.1]hexane-5-carboxylic acid (17b)

General procedure D was used. Product was isolated by column chromatography (gradient, petroleum ether/EtOAc, 9:1 → 7:3). Yield over 3 steps: 5.29 g, 0.019 mol, 59%, yellow solid, m.p. = 112-113 °C, mixture of diastereomers ~ 3:1. The major one: ^{1}H NMR (400 MHz, DMSO-d$_6$): δ 12.58 (br s, 1H), 7.93 (d, *J* = 7.8 Hz, 1H), 7.75 (d, *J* = 8.1 Hz, 1H), 7.67 (t, *J* = 7.4 Hz, 1H), 7.53 – 7.49 (m, 1H), 4.69 (s, 1H), 4.03 (d, *J* = 5.4 Hz, 1H), 3.79 (d, *J* = 5.5 Hz, 1H), 3.26 (s, 1H), 2.06 (d, *J* = 7.7 Hz, 1H), 1.89 (d, *J* = 6.6 Hz, 1H) ppm. ^{19}F{^{1}H} NMR (376 MHz, DMSO-d$_6$): δ -57.9 (s) ppm. LCMS (M-H)$^-$: 271. HRMS (ESI-TOF) *m/z*: [M - H]$^-$ calcd for $C_{13}H_{10}F_3O_3$, 271.0582; found 271.0577.

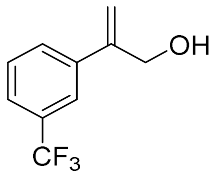

2-(3-(Trifluoromethyl)phenyl)prop-2-en-1-ol

General procedure A was used. Yield: 7.58 g, 0.0375 mol, 75%, colorless oil, b.p. = 64-65 °C, 1 mmHg. ^{1}H NMR (400 MHz, $CDCl_3$): δ 7.70 (s, 1H), 7.63 (d, *J* = 7.8 Hz, 1H), 7.56 (d, *J* = 7.7 Hz, 1H), 7.47 (t, *J* = 7.8 Hz, 1H), 5.54 (s, 1H), 5.45 (s, 1H), 4.56 (s, 2H) ppm. ^{13}C{^{1}H} NMR (151 MHz, $CDCl_3$): δ 146.2, 139.5, 131.1 (q, *J* = 32 Hz), 129.5, 129.1, 124.7 (q, *J* = 4 Hz), 124.2 (q, *J* = 272 Hz), 123.1 (q, *J* = 4 Hz), 114.6, 65.0 ppm. ^{19}F{^{1}H} NMR (376 MHz, $CDCl_3$): δ -63.2 (s) ppm. GCMS (M): 202. HRMS (ESI-TOF) *m/z*: [M + H]$^+$ calcd for $C_{10}H_{10}F_3O$, 203.0684; found 203.0680.

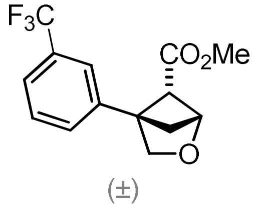

(±)-Methyl-4-(3-(trifluoromethyl)phenyl)-2-oxabicyclo[2.1.1]hexane-5-carboxylate (18a)

General procedure C was used to provide a crude mixture of products (d.r. = 4:1) with benzophenone. An analytically pure sample of the product was obtained by purification of the sample of the crude mixture by HPLC: Rt = 2-10 min, water/acetonitrile, 40-60%, flow 30 mL/min (loading pump 4 mL/min). Colorless oil, mixture of diastereomers ~ 3:1. The major one: ^{1}H NMR (400 MHz, DMSO-d$_6$): δ 7.83 (d, *J* = 7.9 Hz, 1H), 7.76 (d, *J* = 7.8 Hz, 1H), 7.67 (t, *J* = 7.5 Hz, 1H), 7.53 (t, *J* = 7.9 Hz, 1H), 4.72 (s, 1H), 4.00 (d, *J* = 5.6 Hz, 1H), 3.82 (d, *J* = 5.5 Hz, 1H), 3.67 (s, 3H), 3.39 (s, 1H), 2.11 (d, *J* = 7.5 Hz, 1H), 1.92 (d, *J* = 6.9 Hz, 1H) ppm. ^{13}C{^{1}H} NMR (126 MHz, $CDCl_3$): δ 169.4, 138.2, 131.1 (q, *J* = 32.2 Hz), 130.6, 129.2, 124.5 (q, *J* = 3.7

Hz), 123.9 (q, J = 3.8 Hz), 79.4, 69.7, 56.7, 55.0, 51.8, 42.4 ppm. $^{19}F\{^1H\}$ NMR (376 MHz, DMSO-d$_6$): δ -57.9 (s), -58.6 (s) ppm. HRMS (ESI-TOF) *m/z*: $[M + H]^+$ calcd for $C_{14}H_{14}F_3O_3$, 287.0895; found 287.0905.

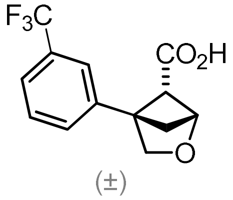

(±)-4-(3-(Trifluoromethyl)phenyl)-2-oxabicyclo[2.1.1]hexane-5-carboxylic acid (18b)

General procedure D was used Product was isolated by column chromatography (gradient, petroleum ether/EtOAc, 9:1→ 7:3). Yield over 3 steps: 6.76 g, 0.025 mol, 58%, white solid, m.p. = 100-101 °C, single stereoisomer. ^1H NMR (400 MHz, DMSO-d$_6$): δ 12.46 (s, 1H), 7.79 (s, 1H), 7.74 (d, J = 7.4 Hz, 1H), 7.65 (d, J = 7.7 Hz, 1H), 7.59 (t, J = 7.6 Hz, 1H), 4.73 (s, 1H), 4.04 (d, J = 5.5 Hz, 1H), 3.80 (d, J = 5.5 Hz, 1H), 3.20 (s, 1H), 2.03 (d, J = 7.2 Hz, 1H), 1.79 (d, J = 7.3 Hz, 1H) ppm. $^{13}C\{^1H\}$ NMR (126 MHz, DMSO-d$_6$): δ 170.6, 139.1, 131.4, 129.3, 129.1 (q, J = 32 Hz), 124.2 (q, J = 272 Hz), 123.8 (q, J = 4 Hz), 123.7 (q, J = 4 Hz), 78.8, 68.3, 55.4, 54.3, 41.8 ppm. $^{19}F\{^1H\}$ NMR (376 MHz, DMSO-d$_6$): δ -61.4 (s) ppm. LCMS $(M-H)^-$: 271. HRMS (ESI-TOF) *m/z*: $[M - H]^-$ calcd for $C_{13}H_{10}F_3O_3$, 271.0582; found 271.0574.

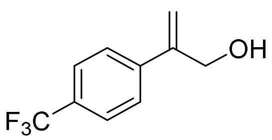

2-(4-(Trifluoromethyl)phenyl)prop-2-en-1-ol

General procedure A was used. Yield: 5.56 g, 0.0275 mol, 55%, ca. 90% purity, colorless oil, b.p. = 70-71 °C, 1 mmHg. ^1H NMR (400 MHz, $CDCl_3$): δ 7.61 (d, J = 8.5 Hz, 2H), 7.56 (d, J = 8.3 Hz, 2H), 5.55 (s, 1H), 5.46 (s, 1H), 4.55 (s, 2H) ppm. $^{13}C\{^1H\}$ NMR (126 MHz, $CDCl_3$): δ 146.3, 132.1, 130.2 (J = 30 Hz), 127.8 (J = 273Hz), 126.6, 125.6 (q, J = 3.7 Hz), 115.0, 65.0 ppm. $^{19}F\{^1H\}$ NMR (376 MHz, $CDCl_3$): δ -63.1 (s) ppm. GCMS (M): 202. HRMS (ESI-TOF) *m/z*: $[M + H]^+$ calcd for $C_{10}H_{10}F_3O$, 203.0684; found 203.0680.

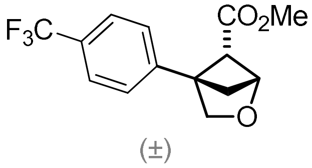

(±)-Methyl-4-(4-(trifluoromethyl)phenyl)-2-oxabicyclo[2.1.1]hexane-5-carboxylate (19a)

General procedure C was used to provide a crude mixture of products (d.r. = 3:1) with benzophenone. An analytically pure sample of the product was obtained by purification of the sample of the crude mixture by HPLC: Rt = 2-10 min, water/acetonitrile, 40-60%, flow 30 mL/min (loading pump 4 mL/min). Yellow oil, mixture of diastereomers ~ 4:1. Signals of the major stereoisomer are given. ^{1}H NMR (400 MHz, DMSO-d$_6$): δ 7.73 (d, *J* = 8.1 Hz, 2H), 7.65 (d, *J* = 8.1 Hz, 2H), 4.77 (s, 1H), 4.02 (d, *J* = 5.7 Hz, 1H), 3.84 (d, *J* = 5.7 Hz, 1H), 3.60 (s, 3H), 3.31 (s, 1H), 2.07 (d, *J* = 7.3 Hz, 1H), 1.80 (d, *J* = 7.4 Hz, 1H) ppm. ^{13}C{^{1}H} NMR (151 MHz, $CDCl_3$): δ 169.4, 141.2, 129.9 (q, *J* = 32.5 Hz), 127.6, 125.6 (q, *J* = 3.7 Hz), 124.2 (q, *J* = 271.8 Hz), 79.5, 69.7, 60.0, 55.0, 51.8, 42.5 ppm. ^{19}F{^{1}H} NMR (376 MHz, DMSO-d$_6$): δ -61.4 (s) ppm. HRMS (ESI-TOF) *m/z*: $[M + H]^+$ calcd for $C_{14}H_{14}F_3O_3$, 287.0895; found 287.0893.

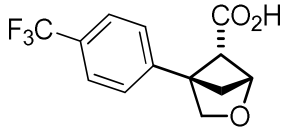

(±)

(±)-4-(4-(Trifluoromethyl)phenyl)-2-oxabicyclo[2.1.1]hexane-5-carboxylic acid (19b)

General procedure D was used. Product was isolated by column chromatography (gradient, petroleum ether/EtOAc, 9:1→ 7:3). Yield over 3 steps: 3.73 g, 0.0137 mol, 59%, yellow solid, m.p. = 64-65 °C, mixture of diastereomers d.r. ~ 4:1. The major one: ^{1}H NMR (400 MHz, DMSO-d$_6$): δ 12.48 (br s, 1H), 7.72 (d, *J* = 8.0 Hz, 2H), 7.65 (d, *J* = 8.1 Hz, 2H), 4.73 (s, 1H), 4.06 (d, *J* = 5.4 Hz, 1H), 3.80 (d, *J* = 5.5 Hz, 1H), 3.17 (s, 1H), 2.02 (d, *J* = 7.2 Hz, 1H), 1.77 (d, *J* = 7.3 Hz, 1H) ppm. ^{13}C{^{1}H} NMR (126 MHz, DMSO-d$_6$): δ 170.5, 170.5, 142.4, 128.0, 127.7 (q, *J* = 32 Hz), 127.5, 125.1 (q, *J* = 4 Hz), 124.3 (q, *J* = 272 Hz), 78.9, 68.3, 55.5, 54.3, 42.0 ppm. ^{19}F{^{1}H} NMR (376 MHz, DMSO-d$_6$): δ -61.4 (s) ppm. LCMS $(M-H)^-$: 271. HRMS (ESI-TOF) *m/z*: $[M - H]^-$ calcd for $C_{13}H_{10}F_3O_3$, 271.0582; found 271.0582.

General procedure E (2-(pyridin-3-yl)prop-2-en-1-ol as an example)

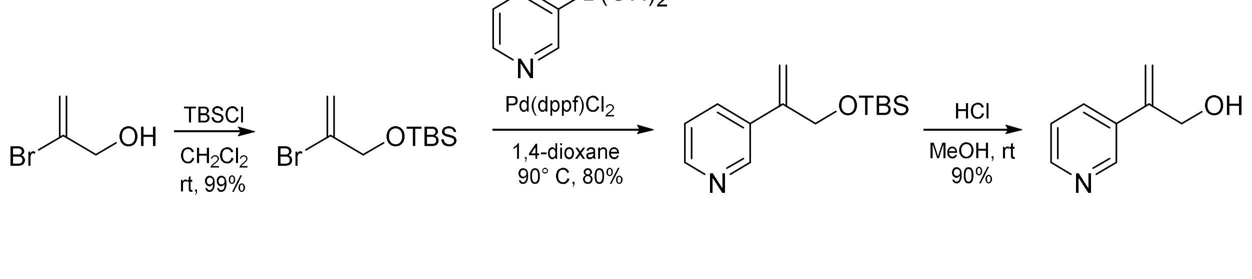

2-(Pyridin-3-yl)prop-2-en-1-ol

A) To a solution of alcohol 2-bromo-2-propen-1-ol (8.85 g, 65.0 mmol, 1.00 equiv) in dry CH_2Cl_2 (200 mL) at 0 °C was added imidazole (6.65 g, 97.6 mmol, 1.50 equiv), DMAP (0.794 g,

6.50 mmol, 0.10 equiv), and TBSCl (14.7 g, 97.6 mmol, 1.50 equiv). The transparent solution turned into a white suspension, and the reaction mixture was stirred first at 0 °C and then for 5 h at room temperature. The reaction was quenched with H_2O (100 mL), the phases were separated, and the aqueous phase was extracted with CH_2Cl_2 (3 × 100 mL). The combined organic phases were washed with brine, dried over Na_2SO_4, filtered, and the solvent was removed under reduced pressure. The residue was purified by distillation under reduced pressure. B.p. = 34-36 °C (0.3 mm). Yield: 14.7 g, 58.3 mmol, 89%, colorless oil.

B) A mixture of pyridin-3-ylboronic acid (0.72 g, 5.86 mmol, 1.00 equiv), 2-bromoallyloxy-tert-butyl-dimethyl-silane (1.47 g, 5.86 mmol, 1.00 equiv), Pd(dppf)Cl_2▪CH_2Cl_2 (0.24 g, 0.293 mmol, 5mol%) and K_2CO_3 (2.02 g, 14.65 mmol, 2.50 equiv) in 1,4-dioxane (50 mL) and water (5 mL) were heated to about 90 °C for about 12 h. Water (150 mL) was added, and the mixture was extracted with EtOAc (5*50 mL). Combined organic phase was dried over Na_2SO_4 and was concentrated under reduced pressure. The residue was purified by column chromatography (CH_2Cl_2/CH_3CN, 5:1) to afford the desire product 1.17 g, 4.69 mmol, 80% as a colorless oil.

C) To a solution of 3-(3-((tert-butyldimethylsilyl)oxy)prop-1-en-2-yl)pyridine (1.17 g, 4.69 mmol, 1.00 equiv) in MeOH (20 mL) was added 6M HCl (1.2 mL, 7.04 mmol, 1.50 equiv) at -20 °C. After being stirred at room temperature for 1 h, the reaction mixture was quenched by slowly adding a saturated aqueous $NaHCO_3$ solution (5 mL). MeOH was removed under reduced pressure, and the aqueous phase was extracted with MeO*t*Bu (3 × 10 mL). The combined organic layers were dried over $MgSO_4$ and concentrated under reduced pressure. Yield: 0.57 g, 4.22 mmol, 90%, brown oil. ^{1}H NMR (400 MHz, $CDCl_3$): δ 8.69 (s, 1H), 8.52 (d, J = 4.3 Hz, 1H), 7.77 (d, J = 7.8 Hz, 1H), 7.28 (dd, J = 7.9, 4.8 Hz, 1H), 5.53 (s, 1H), 5.46 (s, 1H), 4.55 (s, 2H) ppm. $^{13}C\{^1H\}$ NMR (126 MHz, $CDCl_3$): δ 148.7, 147.4, 144.7, 134.6, 133.9, 123.5, 114.6, 64.5 ppm. GCMS (M): 135. HRMS (ESI-TOF) *m/z*: $[M + H]^+$ calcd for $C_8H_{10}NO$, 136.0762; found 136.0757.

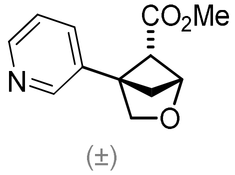

(±)-Methyl-4-(pyridin-3-yl)-2-oxabicyclo[2.1.1]hexane-5-carboxylate (20a)

General procedure C was used to provide a crude mixture of products (d.r. = 4:1) with benzophenone. An analytically pure sample of the product was obtained by purification of the sample of the crude mixture by HPLC: Rt = 2-10 min, water/acetonitrile, 20-45%, flow 30

mL/min (loading pump 4 mL/min). Yellow oil, single stereoisomer. ^{1}H NMR (400 MHz, $CDCl_3$): δ 8.60 (d, *J* = 1.8 Hz, 1H), 8.51 (dd, *J* = 4.8, 1.5 Hz, 1H), 7.77 (dt, *J* = 6.0, 1.9 Hz, 1H), 7.25 (dd, *J* = 7.8, 4.8 Hz, 1H), 4.82 (s, 1H), 4.26 (d, *J* = 5.9 Hz, 1H), 3.79 (d, *J* = 6.0 Hz, 1H), 3.68 (s, 3H), 2.89 (s, 1H), 2.00 (d, *J* = 7.7 Hz, 1H), 1.91 (d, *J* = 7.7 Hz, 1H) ppm. $^{13}C\{^{1}H\}$ NMR (126 MHz, $CDCl_3$): δ 169.3, 149.0, 148.5, 134.9, 132.8, 123.5, 79.6, 69.5, 54.9, 54.9, 51.8, 42.0 ppm. HRMS (ESI-TOF) *m/z*: $[M + H]^+$ calcd for $C_{12}H_{16}NO_3$, 220.0974; found 220.0972.

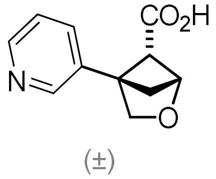

(±)-4-(Pyridin-3-yl)-2-oxabicyclo[2.1.1]hexane-5-carboxylic acid (20b)

General procedure D was used. Product was isolated by crystallization from an acetone-water mixture. Yield over 3 steps: 0.75 g, 0.0037 mol, 74%, yellow solid, m.p. = 180-181 °C, single stereoisomer. ^{1}H NMR (400 MHz, DMSO-d_6): δ 12.48 (br s, 1H), 8.63 (s, 1H), 8.53 – 8.44 (m, 1H), 7.84 (d, *J* = 7.8 Hz, 1H), 7.38 (dd, *J* = 7.6, 4.9 Hz, 1H), 4.73 (s, 1H), 4.05 (d, *J* = 5.5 Hz, 1H), 3.81 (d, *J* = 5.6 Hz, 1H), 3.18 (s, 1H), 2.03 (d, *J* = 7.2 Hz, 1H), 1.74 (d, *J* = 7.3 Hz, 1H) ppm. $^{13}C\{^{1}H\}$ NMR (126 MHz, DMSO-d_6): δ 170.5, 148.4, 148.3, 134.9, 133.2, 123.4, 78.9, 68.1, 54.2, 53.7, 41.7 ppm. LCMS $(M+H)^+$: 206. HRMS (ESI-TOF) *m/z*: $[M + H]^+$ calcd for $C_{11}H_{10}NO_3$, 204.0661; found 204.0659.

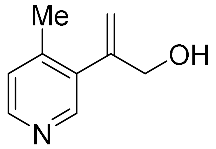

2-(4-Methylpyridin-3-yl)prop-2-en-1-ol

General procedure E was used. Yield: 1.10 g, 7.38 mmol, 63%, brown solid, m.p. = 92-93 °C, b.p. = 72-73 °C, 0.1 mmHg. ^{1}H NMR (400 MHz, $CDCl_3$): δ 8.28 (br s, 2H), 7.12 (s, 1H), 5.60 (s, 1H), 5.07 (s, 1H), 4.30 (s, 2H), 3.95 (s, 1H), 2.29 (s, 3H) ppm. $^{13}C\{^{1}H\}$ NMR (126 MHz, $CDCl_3$): δ 148.8, 147.9, 146.1, 145.5, 136.8, 125.5, 115.5, 65.7, 19.5 ppm. LCMS $(M+H)^+$: 150. HRMS (ESI-TOF) *m/z*: $[M + H]^+$ calcd for $C_9H_{12}NO$, 150.0919; found 150.0915.

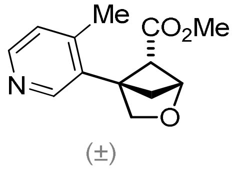

(±)-Methyl-4-(4-methylpyridin-3-yl)-2-oxabicyclo[2.1.1]hexane-5-carboxylate (21a)

General procedure C was used to provide a crude mixture of products (d.r. = 3:1) with benzophenone. An analytically pure sample of the product was obtained by purification of the sample of the crude mixture by HPLC: Rt = 2-10 min, water/acetonitrile, 20-45%, flow 30 mL/min (loading pump 4 mL/min). Yellow oil, single stereoisomer. ^{1}H NMR (400 MHz, $CDCl_3$): δ 8.40 (d, *J* = 3.6 Hz, 1H), 8.31 (s, 1H), 7.12 (d, *J* = 4.7 Hz, 1H), 6.48 (d, *J* = 7.0 Hz, 1H), 5.69 (s, 1H), 5.24 (s, 1H), 4.88 (d, *J* = 7.0 Hz, 1H), 4.66 (s, 2H), 3.69 (s, 3H), 2.32 (s, 3H) ppm. ^{13}C{^{1}H} NMR (151 MHz, $CDCl_3$): δ 169.2, 149.3, 148.9, 145.3, 131.5, 125.8, 79.2, 67.6, 56.2, 54.0, 52.0, 43.7, 20.1 ppm. LCMS $(M+H)^+$: 234. HRMS (ESI-TOF) *m/z*: $[M + H]^+$ calcd for $C_{13}H_{16}NO_3$, 234.1130; found 234.1129.

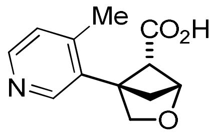

(±)-4-(4-Methylpyridin-3-yl)-2-oxabicyclo[2.1.1]hexane-5-carboxylic acid (21b)

General procedure D was used. Product was isolated by crystallization from an acetone-water mixture. Yield over 3 steps: 0.20 g, 0.91 mmol, 69%, beige solid, m.p. = 225-226 °C, single stereoisomer. ^{1}H NMR (400 MHz, DMSO-d_6): δ 12.62 (br s, 1H), 8.57 (s, 1H), 8.32 (d, *J* = 4.9 Hz, 1H), 7.16 (d, *J* = 4.9 Hz, 1H), 4.72 (s, 1H), 3.95 (d, *J* = 5.7 Hz, 1H), 3.91 (d, *J* = 5.6 Hz, 1H), 3.26 (s, 1H), 2.31 (s, 3H), 1.98 (d, *J* = 7.4 Hz, 1H), 1.95 (d, *J* = 7.5 Hz, 1H) ppm. ^{13}C{^{1}H} NMR (126 MHz, DMSO-d_6): δ 170.8, 149.2, 148.0, 145.2, 131.8, 125.5, 78.7, 66.7, 54.7, 53.8, 42.3, 19.4 ppm. LCMS $(M-H)^-$: 218. HRMS (ESI-TOF) *m/z*: $[M - H]^-$ calcd for $C_{12}H_{14}NO_3$, 220.0974; found 220.0975.

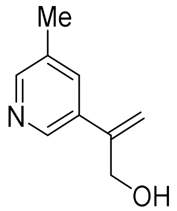

2-(5-Methylpyridin-3-yl)prop-2-en-1-ol

General procedure E was used. Yield: 0.92 g, 6.17 mmol, 60%, yellow oil, b.p. = 80-81 °C, 0.1 mmHg. ^{1}H NMR (400 MHz, DMSO-d_6): δ 8.47 (s, 1H), 8.32 (s, 1H), 7.67 (s, 1H), 5.52 (s, 1H), 5.37 (s, 1H), 4.33 (s, 2H), 2.30 (s, 3H) ppm. ^{13}C{^{1}H} NMR (126 MHz, DMSO-d_6): δ 148.9, 145.1, 144.2, 133.7, 133.6, 132.5, 112.7, 62.4, 17.8 ppm. LCMS $(M+H)^+$: 150. HRMS (ESI-TOF) *m/z*: $[M + H]^+$ calcd for $C_9H_{12}NO$, 150.0919; found 150.0915.

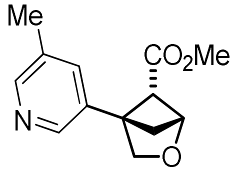

(±)

(±)-Methyl-4-(5-methylpyridin-3-yl)-2-oxabicyclo[2.1.1]hexane-5-carboxylate (22a)

General procedure C was used to provide a crude mixture of products (d.r. = 4:1) with benzophenone. An analytically pure sample of the product was obtained by purification of the sample of the crude mixture by HPLC: Rt = 2-10 min, water/acetonitrile, 5-30%, flow 30 mL/min (loading pump 4 mL/min). Colorless oil, mixture of diastereomers ~ 4:1. The major one: ^{1}H NMR (400 MHz, $CDCl_3$): δ 8.41 (d, *J* = 1.6 Hz, 1H), 8.35 (d, *J* = 1.1 Hz, 1H), 7.58 (s, 1H), 4.83 (s, 1H), 4.27 (d, *J* = 5.7 Hz, 1H), 3.80 (d, *J* = 6.0 Hz, 1H), 3.70 (s, 3H), 2.89 (s, 1H), 2.00 (d, *J* = 7.7 Hz, 1H), 1.91 (dd, *J* = 7.7, 0.8 Hz, 1H) ppm. ^{13}C{^{1}H} NMR (126 MHz, $CDCl_3$): δ 79.6, 69.5, 59.8, 54.9, 51.8, 42.1, 18.5 ppm. LCMS $(M+H)^+$: 234. HRMS (ESI-TOF) *m/z*: $[M + H]^+$ calcd for $C_{13}H_{16}NO_3$, 234.1130; found 234.1127.

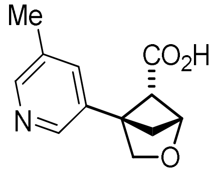

(±)

(±)-(5-Methylpyridin-3-yl)-2-oxabicyclo[2.1.1]hexane-5-carboxylic acid (22b)

General procedure D was used. Product was isolated by crystallization from an acetone-water mixture. Yield over 3 steps: 0.15 g, 0.68 mmol, 67%, yellow solid, m.p. = 204-205 °C, single stereoisomer. ^{1}H NMR (600 MHz, DMSO-d_6): 12.41 (br s, 1H), 8.43 (s, 1H), 8.32 (s, 1H), 7.65 (s, 1H), 4.73 (s, 1H), 4.05 (d, *J* = 5.5 Hz, 1H), 3.79 (d, *J* = 5.6 Hz, 1H), 3.15 (s, 1H), 2.29 (s, 3H), 2.01 (d, *J* = 7.2 Hz, 1H), 1.72 (d, *J* = 7.3 Hz, 1H) ppm. ^{13}C{^{1}H} NMR (151 MHz, DMSO-d_6): 170.4, 148.6, 145.6, 135.2, 132.6, 132.5, 78.9, 68.0, 54.1, 53.6, 41.8, 17.8 ppm. LCMS $(M+H)^+$: 220. HRMS (ESI-TOF) *m/z*: $[M - H]^-$ calcd for $C_{12}H_{14}NO_3$, 220.0974; found 220.0968.

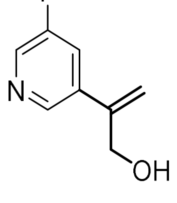

2-(5-Fluoropyridin-3-yl)prop-2-en-1-ol

General procedure E was used. Yield: 7.25 g, 47.38 mmol, 62%, brown solid, m.p. = 56-57 °C, b.p. = 62-63 °C, 0.1 mmHg. ^{1}H NMR (400 MHz, $CDCl_3$): δ 8.54 (s, 1H), 8.40 (d, *J* = 2.5 Hz, 1H), 7.54 (d, *J* = 9.6 Hz, 1H), 5.59 (s, 1H), 5.53 (s, 1H), 4.54 (s, 2H) ppm. ^{13}C{^{1}H} NMR (126 MHz, $CDCl_3$): δ 159.6 (d, *J* = 257 Hz), 143.4, 143.3 (d, *J* = 4 Hz), 137.0 (d, *J* = 24 Hz), 136.2 (d, *J* = 4 Hz), 120.7 (d, *J* = 19 Hz), 116.1, 64.6 ppm. ^{19}F{^{1}H} NMR (376 MHz, $CDCl_3$): δ -127.1 (s) ppm. GCMS (M): 153. HRMS (ESI-TOF) *m/z*: $[M + H]^+$ calcd for C_8H_9FNO, 154.0668; found 154.0661.

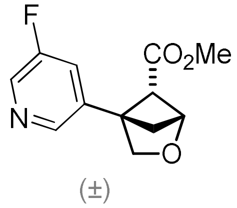

(±)-Methyl-4-(5-fluoropyridin-3-yl)-2-oxabicyclo[2.1.1]hexane-5-carboxylate (23a)

General procedure C was used to provide a crude mixture of products (d.r. = 3:1) with benzophenone. An analytically pure sample of the product was obtained by purification of the sample of the crude mixture by HPLC: Rt = 2-10 min, water/acetonitrile, 5-30%, flow 30 mL/min (loading pump 4 mL/min). Colorless oil, mixture of diastereomers ~ 9:2. The major one: ^{1}H NMR (400 MHz, $CDCl_3$): δ 8.43 (s, 1H), 8.40 (d, *J* = 2.8 Hz, 1H), 7.58 (dt, *J* = 6.9, 2.0 Hz, 1H), 4.85 (s, 1H), 4.25 (d, *J* = 5.9 Hz, 1H), 3.81 (d, *J* = 6.0 Hz, 1H), 3.72 (s, 3H), 2.92 (s, 1H), 2.03 (d, *J* = 7.7 Hz, 1H), 1.96 (dd, *J* = 7.7, 0.7 Hz, 1H) ppm. ^{13}C{^{1}H} NMR (126 MHz, $CDCl_3$): δ 169.2, 159.5 (d, *J* = 257.5 Hz), 144.3 (d, *J* = 3.9 Hz), 137.5 (d, *J* = 23.1 Hz), 134.6 (d, *J* = 3.6 Hz), 122.0 (d, *J* = 18.5 Hz), 79.6, 69.6, 59.9, 55.1, 52.0, 42.0 ppm. ^{19}F{^{1}H} NMR (376 MHz, $CDCl_3$): δ -126.94 (s), -126.96 (s) ppm. HRMS (ESI-TOF) *m/z*: $[M + H]^+$ calcd for $C_{12}H_{13}FNO_3$, 238.0879; found 238.0877.

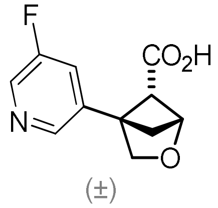

(±)-4-(5-Fluoropyridin-3-yl)-2-oxabicyclo[2.1.1]hexane-5-carboxylic acid (23b)

General procedure D was used. Product was isolated by crystallization from an acetone-water mixture. Yield over 3 steps: 6.86 g, 30.76 mmol, 71%, yellow solid, m.p. = 189-190 °C, single stereoisomer. ^{1}H NMR (400 MHz, DMSO-d_6): δ 12.52 (s, 1H), 8.62 – 8.36 (m, 2H), 8.04 – 7.68 (m, 1H), 4.74 (s, 1H), 4.03 (d, *J* = 5.6 Hz, 1H), 3.84 (d, *J* = 5.7 Hz, 1H), 3.24 (s, 1H), 2.05 (d, *J* = 7.3 Hz, 1H), 1.77 (d, *J* = 7.4 Hz, 1H) ppm. ^{13}C{^{1}H} NMR (126 MHz, DMSO-d_6): δ 170.5, 159.0

(d, J = 254 Hz), 144.8 (d, J = 4 Hz), 136.4 (d, J = 23 Hz), 135.4 (d, J = 4 Hz), 122.0 (d, J = 18 Hz), 78.9, 68.0, 54.3, 53.2, 41.9 ppm. $^{19}F\{^1H\}$ NMR (376 MHz, DMSO-d_6): δ -128.3 (s) ppm. LCMS $(M+H)^+$: 224. HRMS (ESI-TOF) *m/z*: $[M + H]^+$ calcd for $C_{11}H_{11}FNO_3$, 224.0723; found 224.0719.

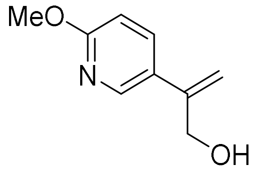

2-(6-Methoxypyridin-3-yl)prop-2-en-1-ol

General procedure E was used. Yield: 10.61 g, 0.0643 mol, 67%, yellow oil, b.p. = 90-92 °C, 0.1 mmHg. ^1H NMR (400 MHz, DMSO-d_6): δ 8.26 (s, 1H), 7.82 (dd, J = 8.6, 2.1 Hz, 1H), 6.80 (d, J = 8.6 Hz, 1H), 5.42 (s, 1H), 5.27 (s, 1H), 5.07 (t, J = 5.4 Hz, 1H), 4.31 (d, J = 5.0 Hz, 2H), 3.85 (s, 3H) ppm. $^{13}C\{^1H\}$ NMR (126 MHz, $CDCl_3$): δ 164.0, 144.4, 144.3, 136.7, 127.6, 112.4, 110.7, 65.0, 53.7 ppm. LCMS $(M+H)^+$: 166. HRMS (ESI-TOF) *m/z*: $[M + H]^+$ calcd for $C_9H_{12}NO_2$, 166.0868; found 166.0861.

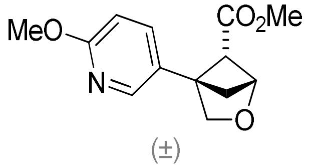

(±)-Methyl-4-(6-methoxypyridin-3-yl)-2-oxabicyclo[2.1.1]hexane-5-carboxylate (24a)

General procedure C was used to provide a crude mixture of products (d.r. = 4:1) with benzophenone. An analytically pure sample of the product was obtained by purification of the sample of the crude mixture by HPLC: Rt = 2-10 min, water/acetonitrile, 5-30%, flow 30 mL/min (loading pump 4 mL/min). Yellow oil, single stereoisomer. ^1H NMR (400 MHz, $CDCl_3$): δ 8.14 (d, J = 2.1 Hz, 1H), 7.68 (dd, J = 8.6, 2.5 Hz, 1H), 6.72 (d, J = 8.5 Hz, 1H), 4.81 (s, 1H), 4.23 (d, J = 5.4 Hz, 1H), 3.92 (s, 3H), 3.76 (d, J = 6.0 Hz, 1H), 3.69 (s, 3H), 2.83 (s, 1H), 1.96 (d, J = 7.7 Hz, 1H), 1.87 (dd, J = 7.7, 0.9 Hz, 1H) ppm. $^{13}C\{^1H\}$ NMR (126 MHz, $CDCl_3$): δ 169.4, 163.8, 145.3, 137.8, 125.5, 110.8, 79.5, 69.5, 55.0, 54.4, 53.6, 51.8, 42.0 ppm. HRMS (ESI-TOF) *m/z*: $[M + H]^+$ calcd for $C_{13}H_{16}NO_4$, 250.1079; found 250.1082. LCMS $(M+H)^+$: 250.

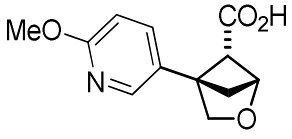

(±)-4-(6-Methoxypyridin-3-yl)-2-oxabicyclo[2.1.1]hexane-5-carboxylic acid (24b)

General procedure D was used. Product was isolated by crystallization from an acetone-water mixture. Yield over 3 steps: 6.58 g, 0.028 mol, 65%, yellow solid, m.p. = 153-154 °C, single stereoisomer. ^{1}H NMR (400 MHz, DMSO-d$_6$): δ 12.42 (br s, 1H), 8.17 (d, *J* = 2.2 Hz, 1H), 7.78 (dd, *J* = 8.5, 2.4 Hz, 1H), 6.81 (d, *J* = 8.5 Hz, 1H), 4.70 (s, 1H), 4.01 (d, *J* = 5.5 Hz, 1H), 3.83 (s, 3H), 3.74 (d, *J* = 5.6 Hz, 1H), 3.08 (s, 1H), 1.97 (d, *J* = 7.2 Hz, 1H), 1.68 (d, *J* = 7.3 Hz, 1H) ppm. ^{13}C{^{1}H} NMR (126 MHz, DMSO-d$_6$): δ 170.6, 162.8, 145.3, 138.3, 126.2, 110.1, 78.8, 68.1, 54.3, 53.2, 53.1, 41.6 ppm. LCMS (M+H)$^{+}$: 236. HRMS (ESI-TOF) *m/z*: [M + H]$^{+}$ calcd for $C_{12}H_{14}NO_4$, 236.0923; found 236.0916.

General procedure F (compound (±)-29 as an example)

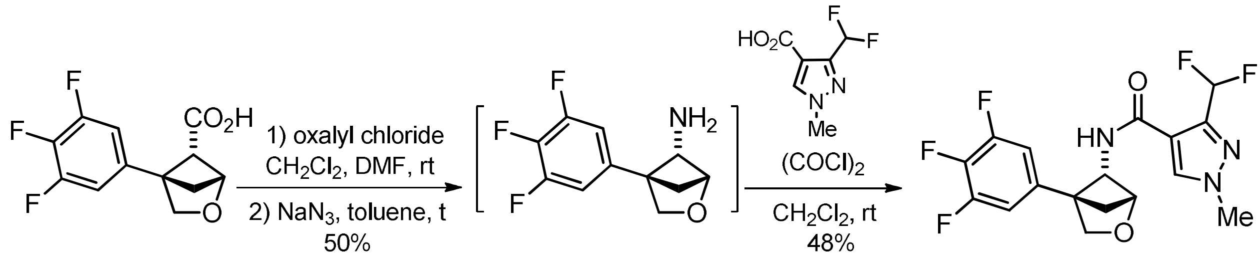

(±)-3-(Difluoromethyl)-1-methyl-*N*-(4-(3,4,5-trifluorophenyl)-2-oxabicyclo[2.1.1]hexan-5-yl)-1*H*-pyrazole-4-carboxamide (29)

A). To a solution of **11b** (1.00 g, 3.88 mmol, 1.00 equiv) in CH_2Cl_2 (10 mL) were added oxalyl chloride (0.74 g, 5.82 mmol, 1.50 equiv) and a drop of DMF. The mixture was stirred at room temperature for 2.5 h and concentrated in *vacuo* to obtain the corresponded carbonyl chloride.

B). In a three-necked flask equipped with an addition funnel and a thermometer was added a solution of NaN_3 (0.50 g, 7.76 mmol, 2.00 equiv) in a mixture of water (2 mL) and acetone (5 mL). The resulting mixture was cooled to -10 °C, and then a solution of carbonyl chloride (1.07 g, 3.88 mmol, 1.00 equiv) in acetone (5 mL) was added slowly. The mixture was stirred for 1 h. After this, a mixture of toluene (30 mL) and water (100 mL) was added. The organic phase was separated, washed with water (1 × 20 mL), dried over Na_2SO_4 and concentrated under reduced pressure to ca. 15 mL of volume of toluene solution. This solution

was added slowly to toluene (15 mL) at 90-100 °C and stirred for 1 h at the same temperature. After this, the reaction mixture was cooled to 0 °C, and *t*-BuOH (0.57 g, 7.76 mmol, 2.00 equiv) was added. The solution was stirred for 2 h at room temperature. The solvent was removed under reduce pressure. The crude product was dissolved in MeOH (15mL), and conc. HCl (1 mL) was added. The resulting mixture was stirred overnight at room temperature. The solvent was removed under reduced pressure, and crude product was dissolved in water (10 mL) and washed with MeO*t*Bu (2 × 10 mL). The aqueous layer was basified with 2M NaOH and extracted with CH_2Cl_2 (3 × 10 mL). The combined organic layers were dried over Na_2SO_4, filtered and concentrated. Yield: 0.44 g, 1.94 mmol, 50%, light yellow liquid.

C). To a stirred solution of 3-(difluoromethyl)-1-methyl-1*H*-pyrazole-4-carboxylic acid (0.31 g, 1.75 mmol, 1.00 equiv) in CH_2Cl_2 (10 mL) were added oxalyl chloride (0.23 mL, 2.63 mmol, 1.50 equiv) and a drop of DMF. The mixture was stirred at room temperature for 2.5 h and concentrated in *vacuo*. The residue was dissolved in CH_2Cl_2 (10 mL) and added to a mixture of 4-(3,4,5-trifluorophenyl)-2-oxabicyclo[2.1.1]hexan-5-amine (0.44 g, 1.94 mmol, 1.10 equiv) in CH_2Cl_2 (5 mL) and Et_3N (0.49 mL, 3.50 mmol, 2.00 equiv) dropwise at 0 °C. The mixture was stirred for 1 h at room temperature, diluted with water (100 mL) and extracted with CH_2Cl_2 (4 × 10 mL). The combined organic layers were dried over $MgSO_4$ and concentrated in *vacuo* to obtain the desired compound. Yield: 0.19 g, 0.49 mmol, 48%, beige solid, m.p. = 122-123 °C. ^1H NMR (500 MHz, DMSO-d_6): δ 8.53 (s, 1H), 7.98 (d, J = 7.7 Hz, 1H), 7.38 – 7.29 (m, 2H), 7.19 (t, J = 54.2 Hz, 1H), 4.53 (s, 1H), 4.29 (d, J = 7.4 Hz, 1H), 3.89 (s, 3H), 3.83 (d, J = 6.3 Hz, 1H), 1.93 (d, J = 7.9 Hz, 1H), 1.71 (d, J = 8.1 Hz, 1H) ppm. $^{13}C\{^1H\}$ NMR (151 MHz, DMSO-d_6): δ 161.4, 150.2 (ddd, J = 247.1, 9.6, 3.8 Hz), 144.5 (t, J = 23.5 Hz), 137.8 (dt, J = 248.2, 15.4 Hz), 134.3 (td, J = 7.8, 4.6 Hz), 133.6, 115.2, 111.9 (d, J = 4.1 Hz), 111.8 (d, J = 3.8 Hz), 109.9 (t, J = 234.2 Hz), 78.0, 66.5, 56.6, 55.2, 38.5 pmm. $^{19}F\{^1H\}$ NMR (376 MHz, DMSO-d_6): δ -114. 3 (m), -136.0 (d, J = 21.9 Hz), -163.9 (t, J = 22.0 Hz) ppm. LCMS $(M+H)^+$: 388. HRMS (ESI-TOF) *m/z*: $[M + H]^+$ calcd for $C_{17}H_{15}F_5N_3O_2$, 388.1084; found 388.1081.

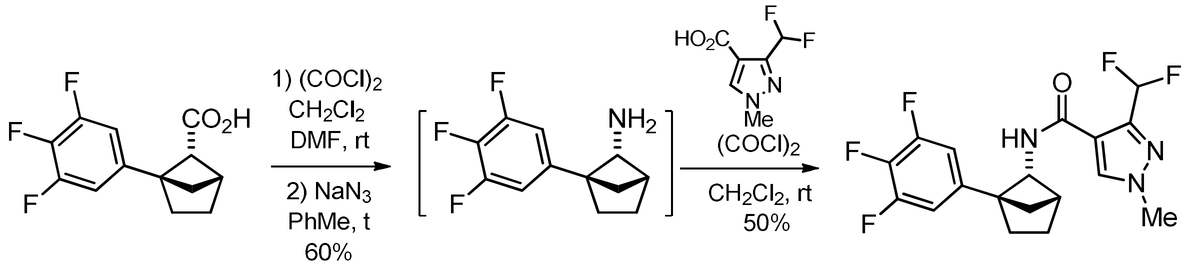

(±)-**S1** (±)-**28**

(±)-3-(Difluoromethyl)-1-methyl-*N*-(1-(3,4,5-trifluorophenyl)bicyclo[2.1.1]hexan-5-yl)-1*H*-pyrazole-4-carboxamide (28)

General procedure F was used staring from acid (±)-**S1**.[1] Yield: 0.20 g, 0.519 mmol, 30%, beige solid, m.p. = 132-133 °C. ^{1}H NMR (500 MHz, DMSO-d$_6$): δ 8.43 (s, 1H), 7.61 (d, *J* = 6.9 Hz, 1H), 7.39 – 7.08 (m, 2H), 7.24 (t, *J* = 54.2 Hz, 1H), 3.97 (d, *J* = 4.7 Hz, 1H), 3.90 (s, 3H), 2.56 (s, 1H), 2.03 – 1.95 (m, 1H), 1.88 – 1.77 (m, 2H), 1.74 – 1.63 (m, 1H), 1.61 – 1.53 (m, 1H), 1.29 (d, *J* = 6.9 Hz, 1H) ppm. ^{13}C{^{1}H} NMR (126 MHz, DMSO-d$_6$): δ 161.5, 150.0 (ddd, *J* = 246.9, 9.7, 4.1 Hz), 144.4 (t, *J* = 23.5 Hz), 139.0 – 138.8 (m), 137.3 (dt, *J* = 247.5, 15.5 Hz), 133.3, 115.6 (t, *J* = 3.1 Hz), 111.4 (d, *J* = 4.4 Hz), 111.2 (d, *J* = 4.3 Hz), 109.9 (t, *J* = 234.3 Hz), 55.8, 54.9, 38.1, 27.4, 24.1 ppm. ^{19}F{^{1}H} NMR (376 MHz, DMSO-d$_6$): δ -114.21 (s, 2F), -136.40 (d, *J* = 22.0 Hz, 2F), -165.20 (t, *J* = 22.0 Hz, 1F) ppm. LCMS (M+H)$^+$: 386. HRMS (ESI-TOF) *m/z*: [M + H]$^+$ calcd for $C_{18}H_{17}F_5N_3O$, 386.1292; found 386.1297.

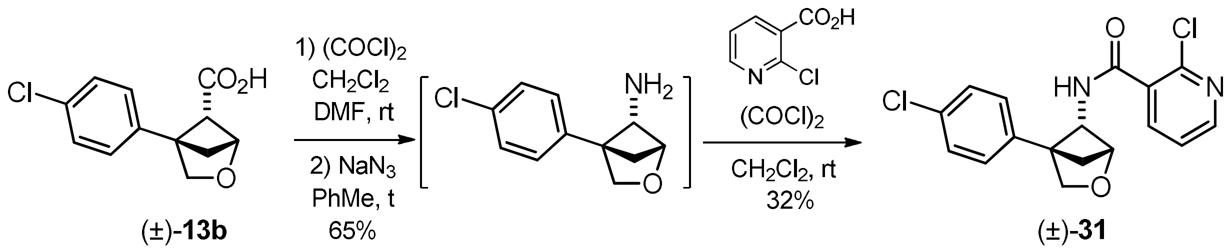

(±)-2-Chloro-*N*-(4-(4-chlorophenyl)-2-oxabicyclo[2.1.1]hexan-5-yl)nicotinamide (31)

General procedure F was used. Yield: 0.11 g, 0.315 mmol, 21%, yellow solid, m.p. = 147-148 °C. ^{1}H NMR (500 MHz, DMSO-d$_6$): δ 8.82 (d, *J* = 8.4 Hz, 1H), 8.44 (dd, *J* = 4.7, 1.6 Hz, 1H), 7.84 (dd, *J* = 7.5, 1.6 Hz, 1H), 7.46 (dd, *J* = 7.4, 4.8 Hz, 1H), 7.40 (q, *J* = 8.5 Hz, 4H), 4.55 (s, 1H), 4.28 (d, *J* = 8.4 Hz, 1H), 3.92 (d, *J* = 6.2 Hz, 1H), 3.75 (d, *J* = 6.3 Hz, 1H), 1.94 (d, *J* = 7.9 Hz, 1H), 1.72 (d, *J* = 8.0 Hz, 1H) ppm. ^{13}C{^{1}H} NMR (126 MHz, DMSO-d$_6$): δ 165.4, 150.0, 146.4, 138.1, 136.0, 133.0, 131.8, 128.7, 128.3, 122.9, 78.2, 67.1, 56.8, 55.4, 37.8 ppm. LCMS (M+H)$^+$: 350. HRMS (ESI-TOF) *m/z*: [M + H]$^+$ calcd for $C_{17}H_{15}Cl_2N_2O_2$, 349.0511; found 349.0503.

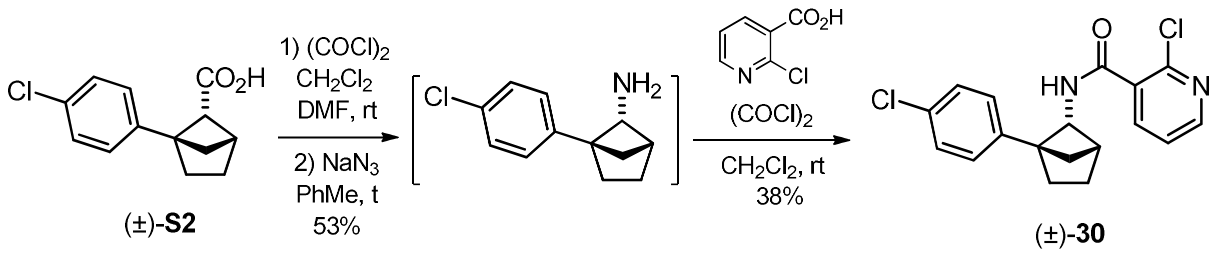

(±)-2-Chloro-*N*-(1-(4-chlorophenyl)bicyclo[2.1.1]hexan-5-yl)nicotinamide (30)

[1] Synthesis of compound (±)-**S1** is described in Ref. 55: A. Denisenko, P. Garbuz, S. V. Shishkina, N. M. Voloshchuk, P. K. Mykhailiuk. Saturated Bioisosteres of ortho-Substituted Benzenes. *Angew. Chem. Int. Ed.* **2020**, *59*, 20515-20521.

General procedure F was used staring from acid (±)-**S2**.[2] Yield: 0.21 g, 0.61 mmol, 20%, beige solid. ^{1}H NMR (500 MHz, DMSO-d$_6$): δ 8.50 (d, *J* = 7.3 Hz, 1H), 8.45 (dd, *J* = 4.3, 1.4 Hz, 1H), 7.83 (dd, *J* = 7.2, 1.4 Hz, 1H), 7.47 (dd, *J* = 7.3, 4.8 Hz, 1H), 7.42 – 7.32 (m, 4H), 3.97 (d, *J* = 5.5 Hz, 1H), 2.60 (s, 1H), 2.05 – 1.93 (m, 1H), 1.92 – 1.81 (m, 1H), 1.73 (br s, 2H), 1.57 (br s, 1H), 1.30 (d, *J* = 6.7 Hz, 1H) ppm. ^{13}C{^{1}H} NMR (126 MHz, DMSO-d$_6$): δ 165.4, 149.9, 146.4, 140.5, 138.1, 133.4, 130.9, 128.3, 128.0, 123.0, 56.1, 55.2, 37.6, 28.1, 24.3 ppm. LCMS $(M+H)^+$: 349. HRMS (ESI-TOF) *m/z*: $[M + H]^+$ calcd for $C_{18}H_{17}Cl_2N_2O$, 347.0718; found 347.0712.

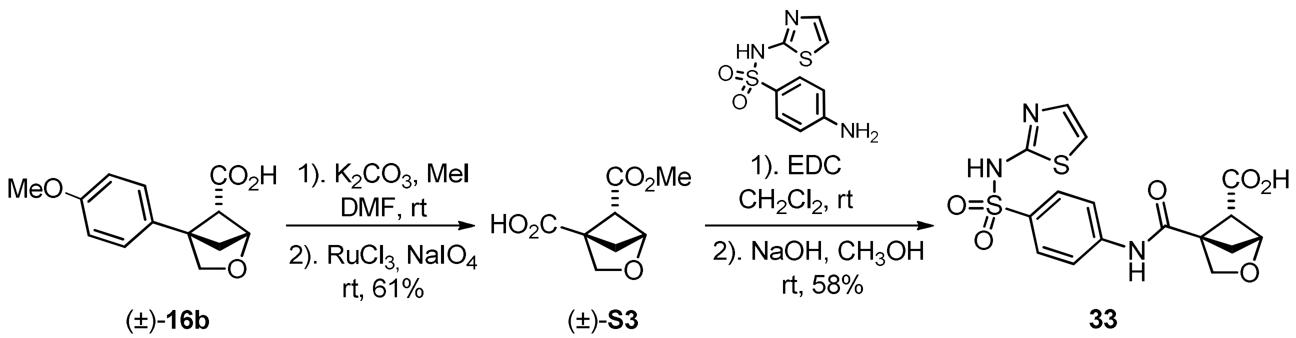

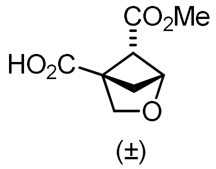

A). (±)-5-(Methoxycarbonyl)-2-oxabicyclo[2.1.1]hexane-4-carboxylic acid (S3).

To a solution of 4-(4-methoxyphenyl)-2-oxabicyclo[2.1.1]hexane-5-carboxylic acid (5.00 g, 0.0214 mol, 1.00 equiv) and K_2CO_3 (5.91 g, 0.0428 mol, 2.00 equiv) in 100 mL of DMF was added MeI (9.12 g, 0.0642 mol, 3.00 equiv) dropwise at 0 °C. The mixture was stirred overnight at room temperature. The solution was diluted with water (200 mL) and extracted with EtOAc (3 × 100 mL). The combined organic layers were washed with water (3 × 100 mL), brine (3 × 100 mL), dried over Na_2SO_4, filtered and concentrated under reduced pressure. The crude product was used without purification. The residue was dissolved in a mixture of H_2O (30 mL), CH_3CN (20 mL) and CH_2Cl_2 (20 mL). $RuCl_3 \cdot \times H_2O$ (0.14 g, 0.000642 mol, 0.03 equiv) and NaOH (3.42 g, 0.0856 mol, 4.00 equiv) were added to the mixture. $NaIO_4$ (13.74 g, 0.0642 mol, 3.00 equiv) was added in portions at 0 °C. The mixture was vigorously stirred overnight at room temperature. Then the mixture was filtered and washed with water. The layers were partitioned.

[2] Synthesis of compound (±)-**S2** is described in Ref. 55: A. Denisenko, P. Garbuz, S. V. Shishkina, N. M. Voloshchuk, P. K. Mykhailiuk. Saturated Bioisosteres of ortho-Substituted Benzenes. *Angew. Chem. Int. Ed.* **2020**, *59*, 20515-20521.

An aqueous layer was washed with MeO*t*Bu (2 × 50 mL). The aqueous layer was acidified with 5M HCl to pH = 2 and extracted with EtOAc (4 × 20 mL). The combined organic phases were dried over Na_2SO_4, filtered and concentrated under reduced pressure to give the desired product. Yield: 2.40 g, 0.013 mol, 61%, white solid. ^{1}H NMR (400 MHz, $CDCl_3$): δ 7.63 (br s, 1H), 4.73 (s, 1H), 4.13 (d, *J* = 6.3 Hz, 1H), 3.91 (d, *J* = 6.3 Hz, 1H), 3.71 (s, 3H), 3.06 (s, 1H), 2.07 (d, *J* = 7.7 Hz, 1H), 1.89 (d, *J* = 7.7 Hz, 1H) ppm. ^{13}C{^{1}H} NMR (126 MHz, $CDCl_3$): δ 173.1, 168.9, 159.2, 79.3, 66.3, 54.1, 52.7, 52.2, 42.1 ppm. LCMS (M-H)$^-$: 185. HRMS (ESI-TOF) *m/z*: [M + H]$^+$ calcd for $C_8H_{11}O_5$, 187.0606; found 187.0601.

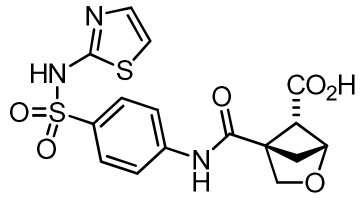

(±)

(±)-4-((4-(*N*-(Thiazol-2-yl)sulfamoyl)phenyl)carbamoyl)-2-oxabicyclo[2.1.1]hexane-5-carboxylic acid (33)

To a solution of 5-(methoxycarbonyl)-2-oxabicyclo[2.1.1]hexane-4-carboxylic acid (74 mg, 0.40 mmol, 1.00 equiv) in CH_2Cl_2 (5 mL) was added Et_3N (80 mg, 0.80 mmol, 2.00 equiv) at room temperature. The mixture was stirred for 10 min and 1-ethyl-(3-dimethylaminopropyl)carbonyldiimide hydrochloride (EDC) (115 mg, 0.60 mmol, 1.50 equiv) was added with stirring. The mixture was stirred for 30 min and 4-amino-*N*-(thiazol-2-yl)benzenesulfonamide (153 mg, 0.60 mmol, 1.50 equiv) was added. The reaction was stirred at room temperature for 12 h. CH_2Cl_2 was removed under reduced pressure. To the residue was added a mixture of CH_3OH (5 mL) and water (10 mL). The mixture was filtered and dried. To a solution of methyl 4-((4-(*N*-(thiazol-2-yl)sulfamoyl)phenyl)carbamoyl)-2-oxabicyclo[2.1.1]hexane-5-carboxylate (150 mg, 0.35 mmol, 1.00 equiv) in MeOH (10 mL) was added NaOH (40 mg, 1.05 mmol, 3.00 equiv) and left stirred for 8 h at room temperature. The solution was evaporated under reduce pressure. The residue was dissolved in water (10 mL) and acidified with 1M HCl to pH = 4. The solid residue was filtered, washed with water (2 × 10 mL) to obtain the desired product as a white solid. Yield: 96 mg, 0.23 mmol, 58% over 2 steps. ^{1}H NMR (500 MHz, DMSO-d_6): δ 12.71 (br s, 2H), 10.04 (s, 1H), 7.77 (s, 4H), 7.25 (d, *J* = 4.6 Hz, 1H), 6.82 (d, *J* = 4.6 Hz, 1H), 4.65 (s, 1H), 3.97 (d, *J* = 5.7 Hz, 1H), 3.83 (d, *J* = 5.8 Hz, 1H), 2.13 – 2.01 (m, 2H), 1.71 (d, *J* = 7.3 Hz, 1H) ppm. ^{13}C{^{1}H} NMR (126 MHz, DMSO-d_6): δ 170.4, 168.7, 167.3, 141.6, 136.8, 126.9, 124.4, 119.2, 108.1, 78.4, 66.3, 56.0, 52.8, 40.9 ppm.

LCMS $(M+H)^+$: 410. HRMS (ESI-TOF) *m/z*: $[M + H]^+$ calcd for $C_{16}H_{16}N_3O_6S_2$, 410.481; found 410.0474.

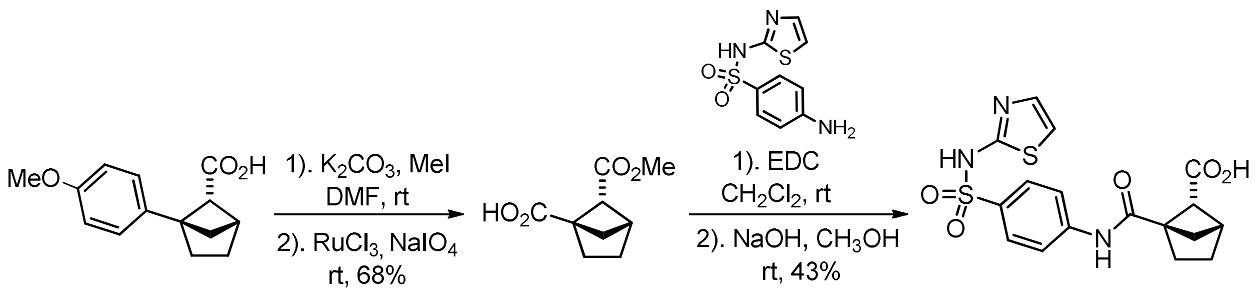

(±)-**S4** (±)-**S5** (±)-**32**

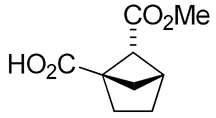

(±)

(±)-5-(Methoxycarbonyl)bicyclo[2.1.1]hexane-1-carboxylic acid (S5)

To a solution of 1-(4-methoxyphenyl)bicyclo[2.1.1]hexane-5-carboxylic acid, (±)-**S4** (5.00 g, 0.0216 mol, 1.00 equiv) [3] and K_2CO_3 (5.96 g, 0.0432 mol, 2.00 equiv) in 100 mL of DMF was added MeI (9.20 g, 0.0648 mol, 3.00 equiv) dropwise at 0 °C. The mixture was stirred overnight at room temperature. The solution was diluted with water (200 mL) and extracted with EtOAc (3 × 100 mL). The combined layers were washed with water (1 × 100 mL), brine (1 × 100 mL), dried over Na_2SO_4, filtered and concentrated under reduced pressure. The crude product was used without purification. The residue was dissolved in a mixture of H_2O (30 mL), CH_3CN (20 mL) and CH_2Cl_2 (20 mL). $RuCl_3\cdot\times H_2O$ (0.15 g, 0.000648, 0.03 equiv) and NaOH (3.46 g, 0.0864 mol, 4.00 equiv) were added to the mixture. $NaIO_4$ (13.87 g, 0.0648 mol, 3.00 equiv) was added in portions at 0 °C. The mixture was vigorously stirred overnight at room temperature. Then the mixture was filtered and washed with water. The layers were partitioned. An aqueous layer was washed with MeO*t*Bu (2 × 50 mL). The aqueous layer was acidified with 5M HCl to pH = 2 and extracted with EtOAc (4 × 50 mL). The combined organic phases were dried over Na_2SO_4, filtered and concentrated under reduced pressure to give the desired product. Yield: 2.70 g, 0.0147 mol, 68%, white solid. ^{1}H NMR (400 MHz, $CDCl_3$): δ 10.83 (br s, 1H), 3.68 (s, 3H), 2.81 (s, 1H), 2.74 (s, 1H), 2.10 – 1.96 (m, 1H), 1.95 – 1.84 (m, 1H), 1.81 – 1.69 (m, 3H), 1.33 (d, *J* = 6.7 Hz, 1H) ppm. $^{13}C\{^1H\}$ NMR (151 MHz, $CDCl_3$): δ 176.9, 171.6, 54.3, 51.9,

[3] Synthesis of compound (±)-**S4** is described in Ref. 55: A. Denisenko, P. Garbuz, S. V. Shishkina, N. M. Voloshchuk, P. K. Mykhailiuk. Saturated Bioisosteres of ortho-Substituted Benzenes. *Angew. Chem. Int. Ed.* **2020**, *59*, 20515-20521.

50.7, 40.6, 40.0, 26.5, 25.5 ppm. HRMS (ESI): calc'd for $C_9H_{11}O_4$ $[M-H]^-$ 183.657; found 183.0660.

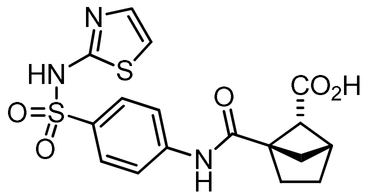

(±)

(±)-1-((4-(*N*-(thiazol-2-yl)sulfamoyl)phenyl)carbamoyl)bicyclo[2.1.1]hexane-5-carboxylic acid (32)

To a solution of 5-(methoxycarbonyl)bicyclo[2.1.1]hexane-1-carboxylic acid (94 mg, 0.51 mmol, 1.00 equiv) in CH_2Cl_2 (5 mL) was added Et_3N (103 mg, 1.02 mmol, 2.00 equiv) at room temperature. The mixture was stirred for 10 min and 1-ethyl-(3-dimethylaminopropyl)carbonyldiimide hydrochloride (EDC) (147 mg, 0.765 mmol, 1.50 equiv) was added with stirring. The mixture was stirred for 30 min and 4-amino-*N*-(thiazol-2-yl)benzenesulfonamide (195 mg, 0.765 mmol, 1.50 equiv) was added. The reaction was stirred at room temperature for 12 h. CH_2Cl_2 was removed under reduced pressure. To the residue was added a mixture of CH_3OH (5 mL) and water (10 mL). The mixture was filtered and dried. The residue was dissolved in MeOH (10 mL) and NaOH (61 mg, 1.53 mmol, 3.00 equiv) was added and left stirred for 8 h at room temperature. The solution was evaporated under reduce pressure. The residue was dissolved in water (10 mL) and acidified with 1M HCl to pH = 4. The solid residue was filtered, washed with water (2 × 5 mL) to obtain the desired product. Yield: 90 mg, 0.22 mmol, 43%, white solid, m.p. = 241-242 °C. ^1H NMR (500 MHz, DMSO-d_6): δ 12.67 (br s, 1H), 9.89 (s, 1H), 7.76 (s, 4H), 7.24 (d, J = 4.6 Hz, 1H), 6.81 (d, J = 4.6 Hz, 1H), 2.99 (s, 1H), 2.62 (s, 1H), 1.98 – 1.82 (m, 2H), 1.77 – 1.63 (m, 3H), 1.26 (d, J = 6.3 Hz, 1H) ppm. $^{13}C\{^1H\}$ NMR (126 MHz, DMSO-d_6): δ 172.7, 170.9, 168.7, 141.9, 136.5, 126.9, 124.4, 119.0, 108.1, 56.2, 50.7, 38.5, 27.0, 25.3 ppm. LCMS $(M+H)^+$: 408. HRMS (ESI-TOF) *m/z*: $[M + H]^+$ calcd for $C_{17}H_{18}N_3O_5S_2$, 408.0688; found 408.0683..

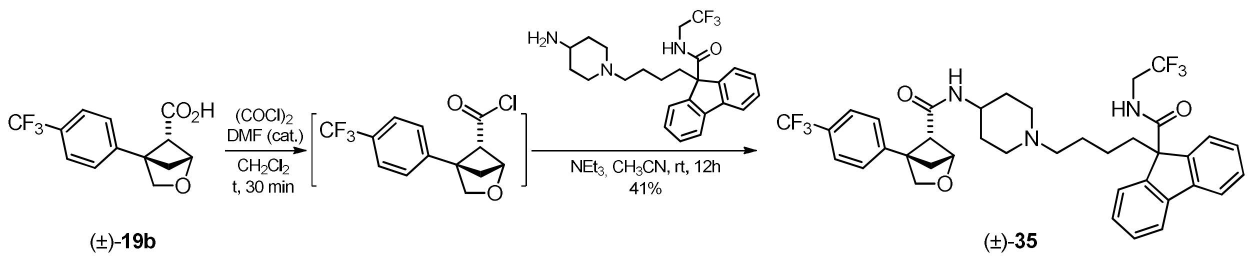

(±)-19b (±)-35

(±)-*N*-(1-(4-(9-((2,2,2-trifluoroethyl)carbamoyl)-9*H*-fluoren-9-yl)butyl)piperidin-4-yl)-4-(4-(trifluoromethyl)phenyl)-2-oxabicyclo[2.1.1]hexane-5-carboxamide (35)

Oxalyl chloride (0.20 mL, 2.31 mmol, 3.00 equiv) was slowly added to a mixture of **19b** (0.21 g, 0.77 mmol, 1.00 equiv), a drop of DMF and CH_2Cl_2 (5 mL) at 25-30 °C. The reaction mixture was stirred for 4 h at the same temperature. The mixture was concentrated under reduced pressure. The obtained compound was dissolved in CH_2Cl_2 (10 mL), and the solution was slowly added to a mixture of 9-(4-(4-aminopiperidin- 1-yl)butyl)-*N*-(2,2,2-trifluoroethyl)-9*H*-fluorene-9-carboxamide dihydrochioride (0.40 g, 0.77 mmol, 1.00 equiv) and Et_3N (3.12 g, 30.89 mmol, 40.12 equiv) in CH_2Cl_2 (5 mL) at -10 °C. The reaction mixture was stirred for 16 h at room temperature and diluted with water (10 mL). Both the organic and aqueous layers were extracted with CH_2Cl_2 (3 × 10 mL). The combined organic layers were concentrated under reduced pressure. The residue was purified by HPLC: Rt = 2-10 min, water/0.1% formic acid/acetonitrile, 30-55%, flow 30 mL/min (loading pump 4 mL/min). Yield: 0.22 g, 0.32 mmol, 41%, yellow solid. ^{1}H NMR (500 MHz, $CDCl_3$): δ 8.32 (br s, 1H), 7.76 (d, *J* = 7.4 Hz, 2H), 7.58 (d, *J* = 7.8 Hz, 2H), 7.55 – 7.48 (m, 3H), 7.45 (t, *J* = 7.3 Hz, 2H), 7.40 – 7.32 (m, 2H), 6.86 (d, *J* = 7.6 Hz, 1H), 6.28 (br s, 1H), 5.35 (s, 1H), 4.77 (s, 1H), 4.05 – 3.61 (m, 5H), 3.37 – 2.99 (m, 3H), 2.63 – 2.30 (m, 6H), 2.09 – 1.82 (m, 4H), 1.55 (br s, 2H), 0.74 (br s, 2H) ppm. $^{13}C\{^1H\}$ NMR (126 MHz, $CDCl_3$): δ 173.3, 168.4, 144.9, 141.1, 129.0, 128.5, 127.4, 127.2, 125.6 (q, *J* = 3.8 Hz), 124.3, 123.9 (q, *J* = 279.0 Hz), 120.7, 78.2, 69.7, 62.2, 57.9, 56.8, 55.3, 51.5, 44.6, 42.9, 40.9 (q, *J* = 35.2 Hz), 35.8, 29.4, 29.2, 24.2, 21.5 ppm. $^{19}F\{^1H\}$ NMR (376 MHz, $CDCl_3$): δ -63.1 (s), -73.3 (s) ppm. LCMS $(M+H)^+$: 700. HRMS (ESI-TOF) *m/z*: $[M + H]^+$ calcd for $C_{38}H_{40}F_6N_3O_3$, 700.2974; found 700.2973.

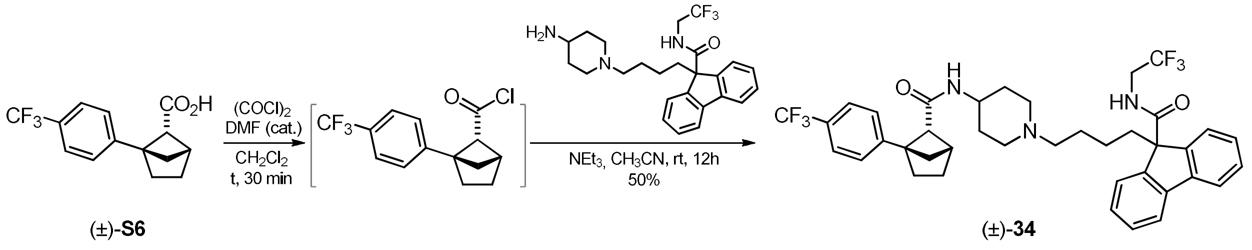

(±)-S6 (±)-34

(±)-*N*-(2,2,2-trifluoroethyl)-9-(4-(4-(1-(4-(trifluoromethyl)phenyl)bicyclo[2.1.1]hexane-5-carboxamido)piperidin-1-yl)butyl)-9*H*-fluorene-9-carboxamide (34)

The same procedure as for **35** was used staring from acid (±)-**S6**.[4] Yield: 0.27 g, 0.39 mmol, 50%, beige solid. ^{1}H NMR (500 MHz, DMSO-d_6): δ 8.18 (s, 1H), 7.88 (d, *J* = 7.5 Hz, 2H), 7.59 (dd, *J* = 20.0, 8.2 Hz, 4H), 7.51 – 7.38 (m, 5H), 7.34 (t, *J* = 7.4 Hz, 2H), 3.72 – 3.61 (m, 1H), 3.52 (br s, 1H), 2.87 – 2.73 (m, 3H), 2.34 – 2.08 (m, 7H), 1.79 – 1.55 (m, 6H), 1.50 – 1.14 (m, 6H), 0.54 (br s, 2H) ppm. ^{13}C{^{1}H} NMR (151 MHz, DMSO-d_6): δ 172.7, 169.3, 163.6, 147.7, 145.5, 140.8, 128.1, 127.7, 127.6, 126.7 (q, *J* = 31.4 Hz), 125.4 (d, *J* = 20.7 Hz), 124.7 (q, *J* = 3.7 Hz), 124.0, 123.6 (d, *J* = 12.6 Hz), 120.4, 61.7, 56.4, 55.9, 53.0, 51.2, 44.7, 40.9, 35.9, 30.4, 30.2, 30.1, 26.1, 25.4, 21.1 ppm. ^{19}F{^{1}H} NMR (376 MHz, DMSO-d_6): δ -61.2 (s), -71.0 (s) ppm. LCMS $(M+H)^+$: 698. HRMS (ESI-TOF) *m/z*: $[M + H]^+$ calcd for $C_{39}H_{42}F_6N_3O_2$, 698.3181; found 698.3186.

[4] Synthesis of compound (±)-**S6** is described in Ref. 55: A. Denisenko, P. Garbuz, S. V. Shishkina, N. M. Voloshchuk, P. K. Mykhailiuk. Saturated Bioisosteres of ortho-Substituted Benzenes. *Angew. Chem. Int. Ed.* **2020**, *59*, 20515-20521.

Copies of ^{1}H, ^{13}C{^{1}H} and ^{19}F{^{1}H} spectra

Compound 3

^{1}H NMR (400 MHz, $CDCl_3$)

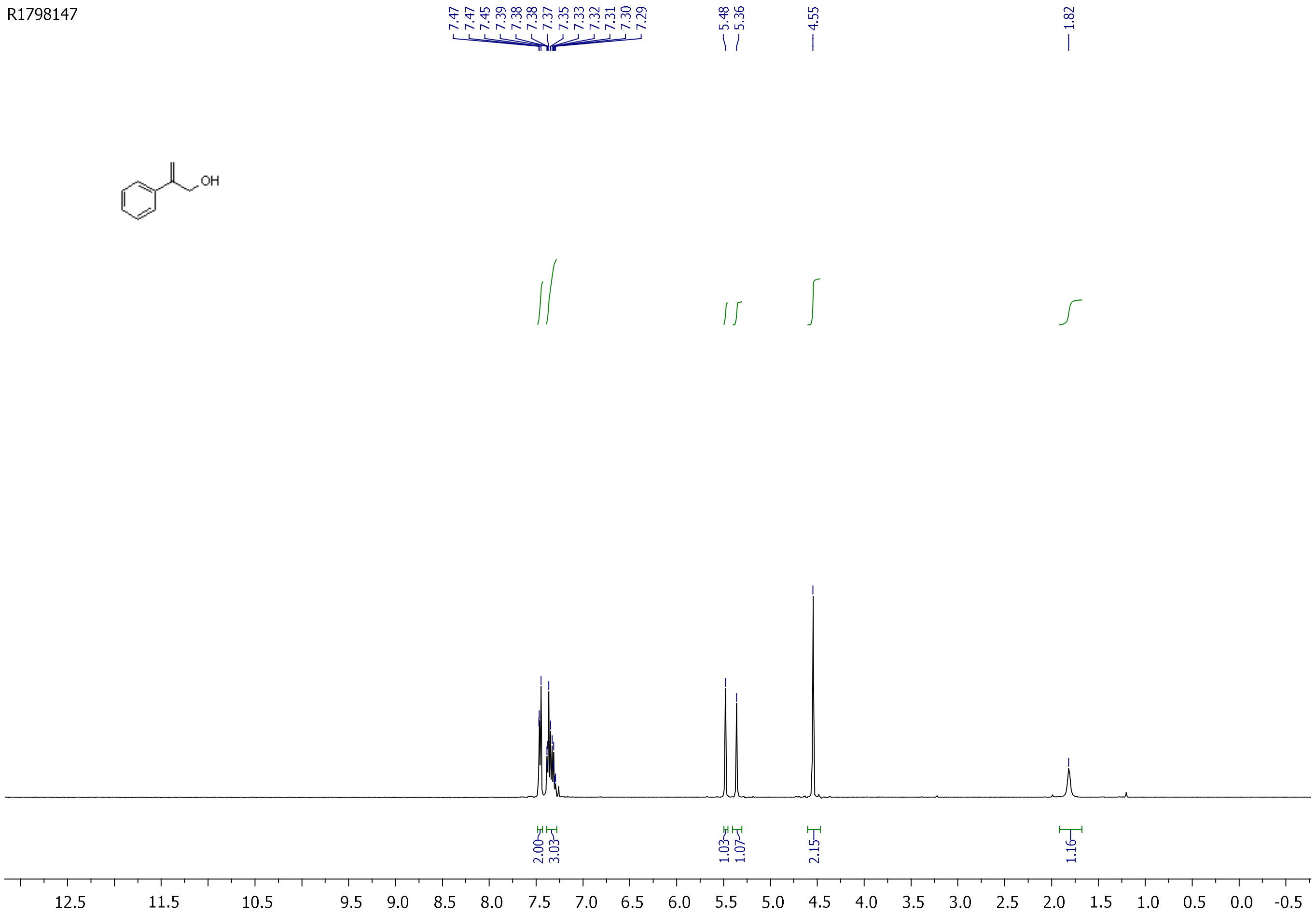

$^{13}C\{^{1}H\}$ NMR (126 MHz, $CDCl_3$)

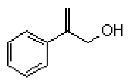

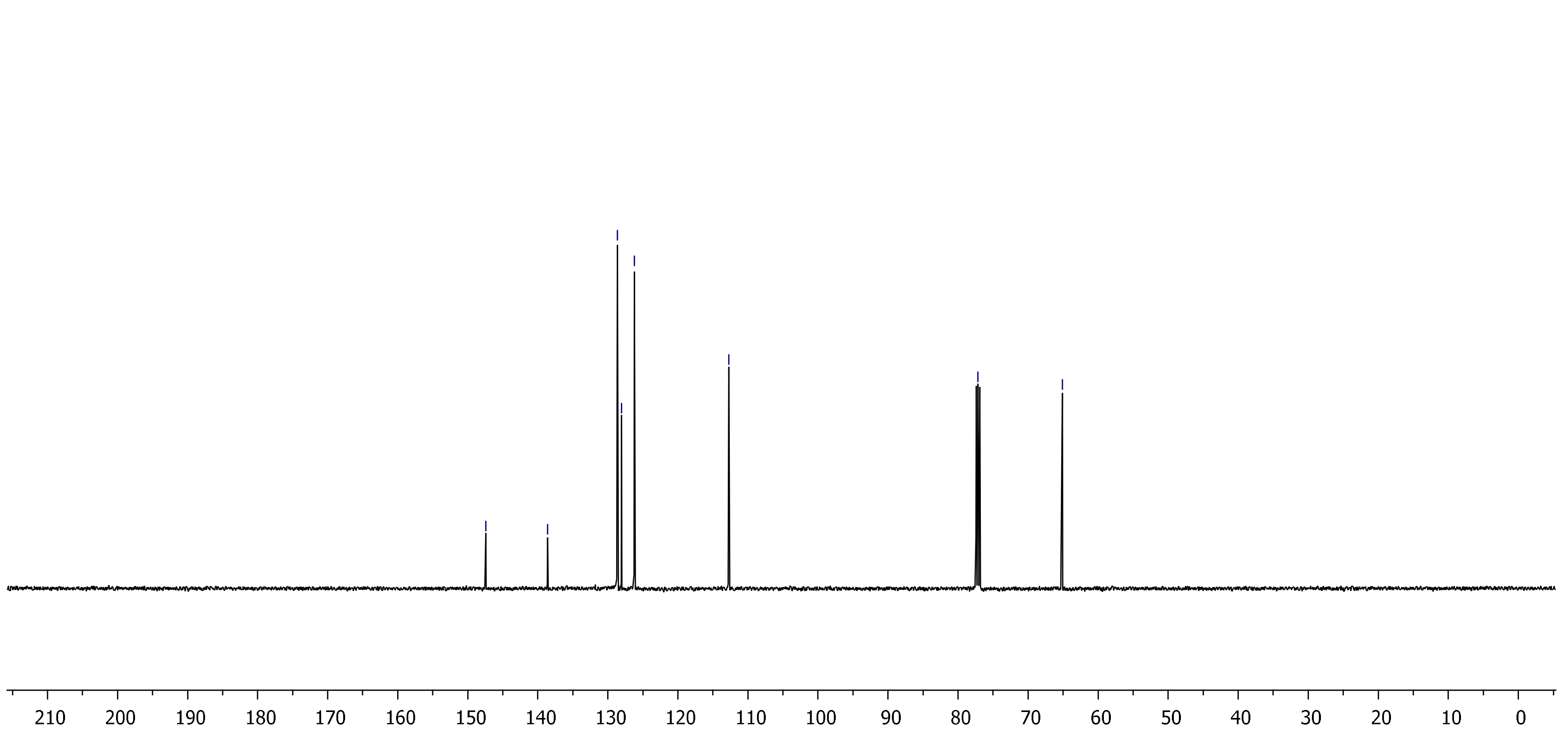

Compound 1, (ca. 90% purity)

^{1}H NMR (500 MHz, $CDCl_3$)

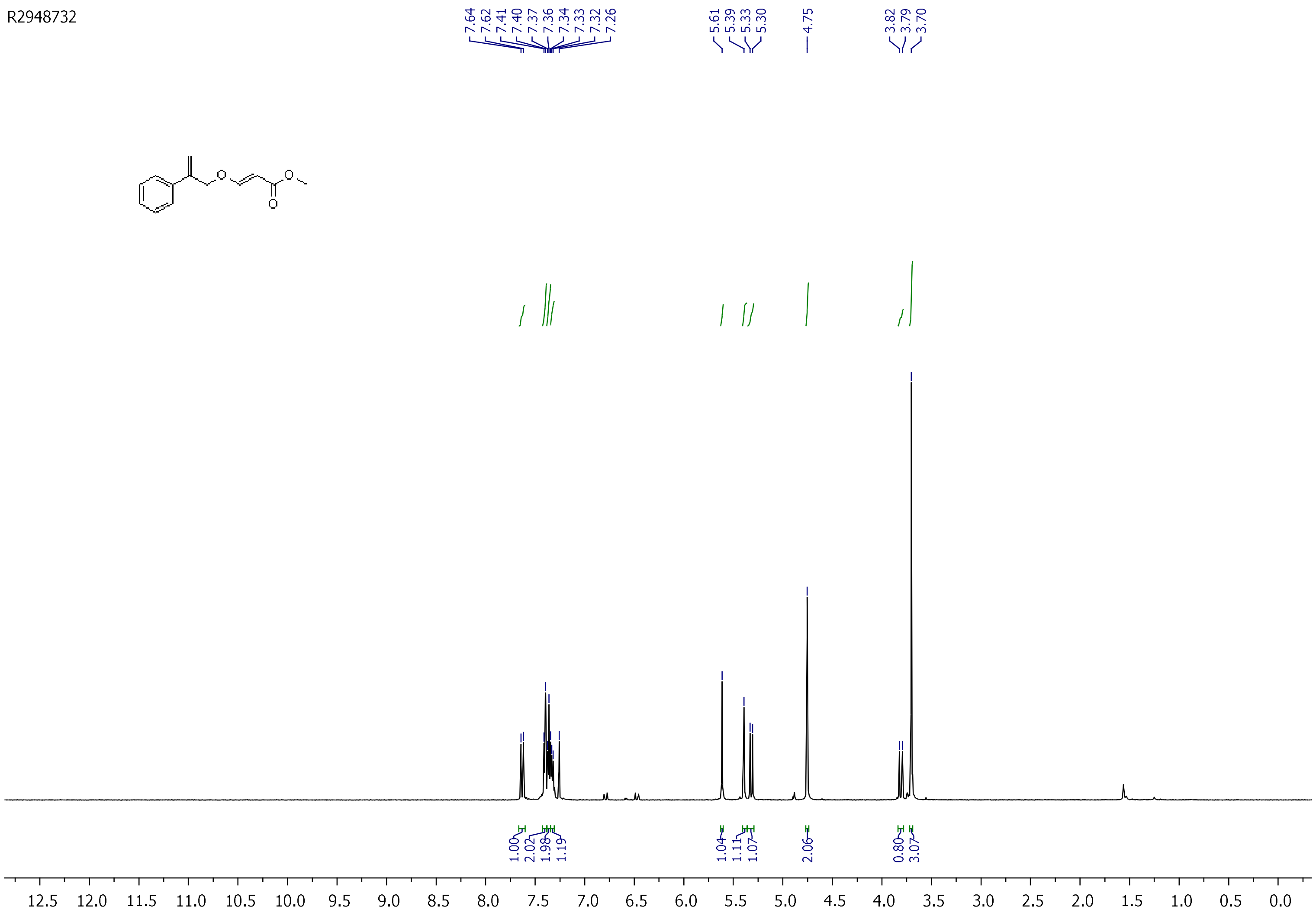

$^{13}C\{^{1}H\}$ NMR (126 MHz, $CDCl_3$)

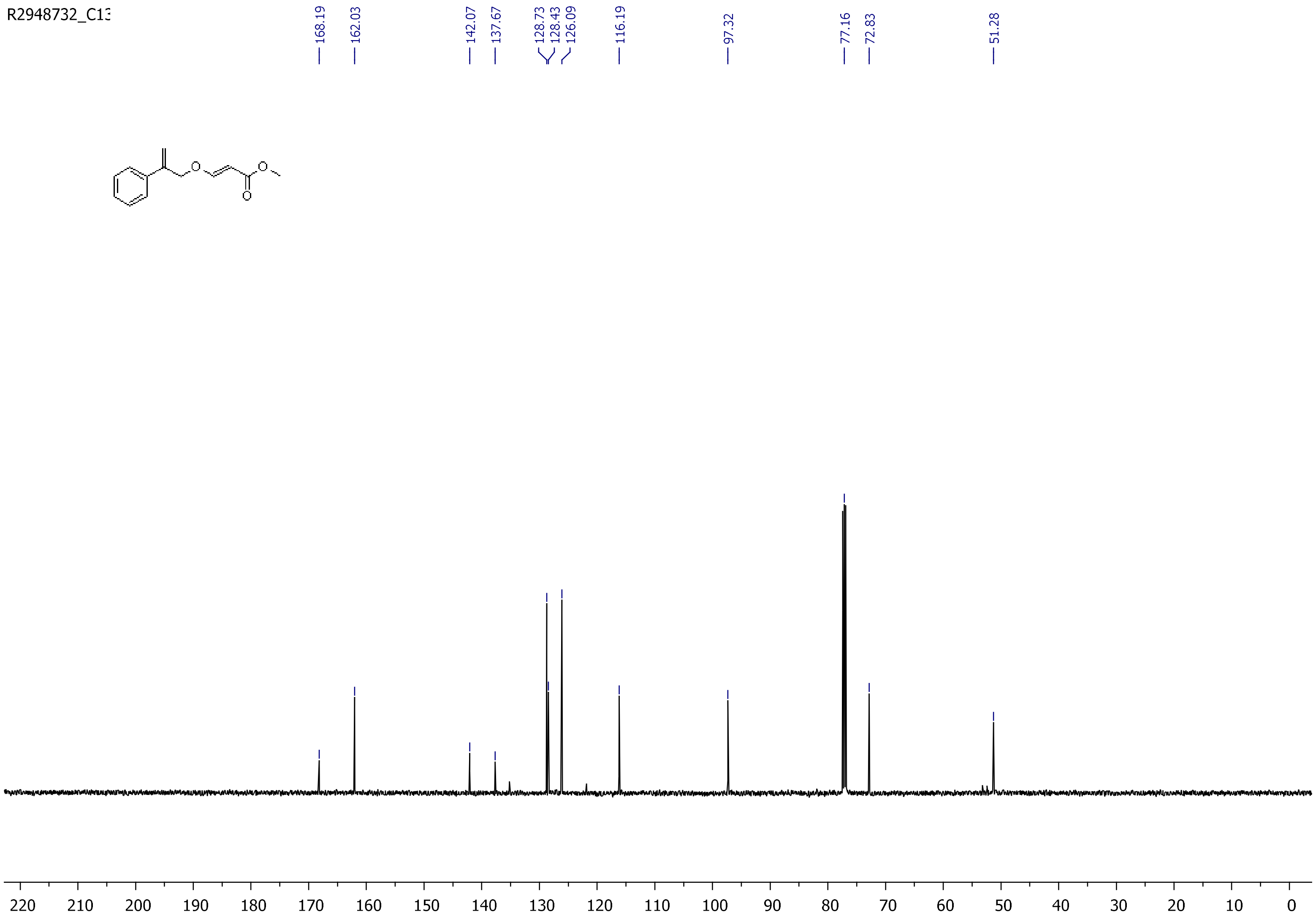

Crude reaction mixture after irradiation of 1: compound (±)-1a, d.r. = 81:19 (ca. 4:1) + benzophenone.

^{1}H NMR (400 MHz, $CDCl_3$)

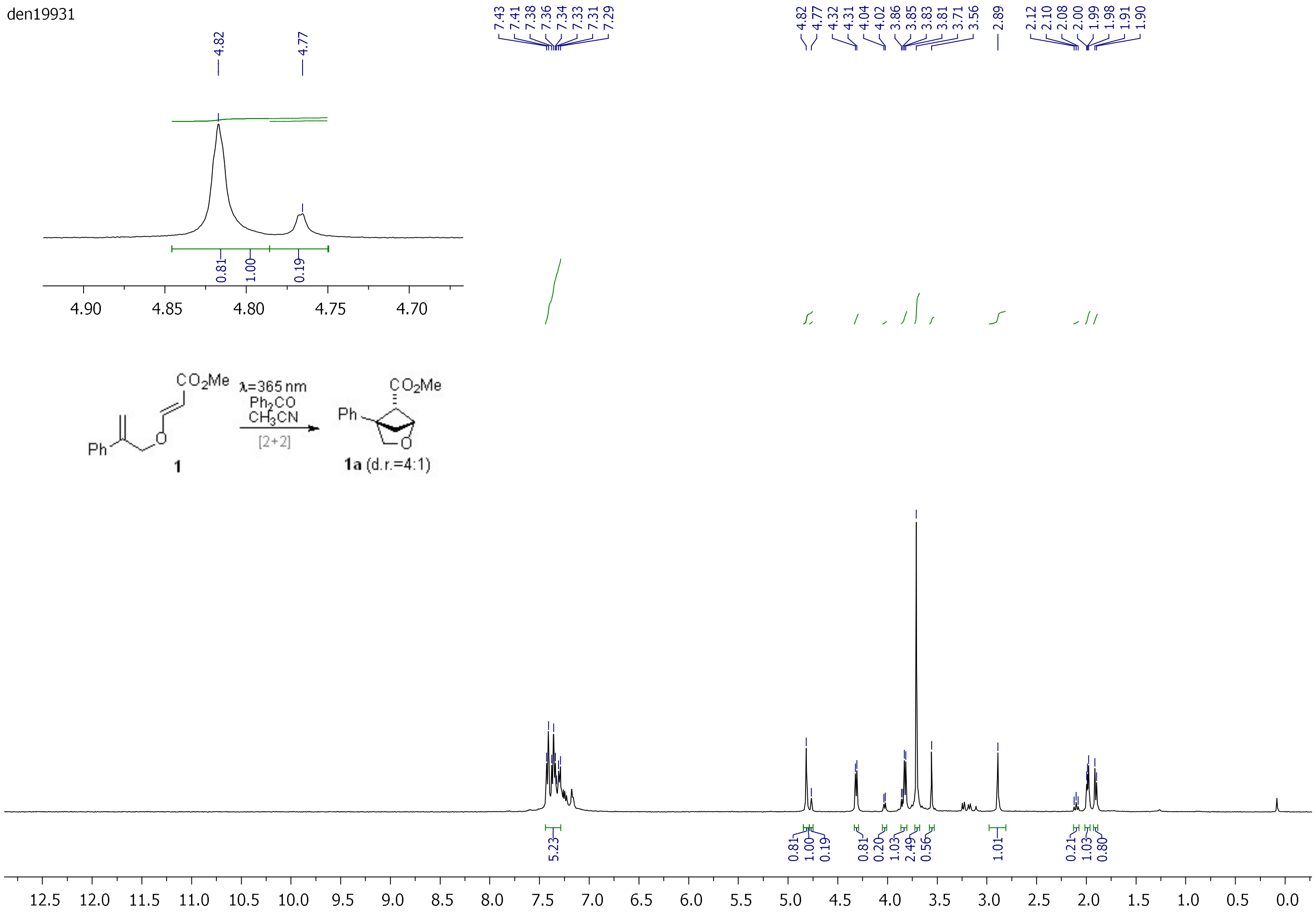

Compound (±)-1a

^{1}H NMR (400 MHz, $CDCl_3$)

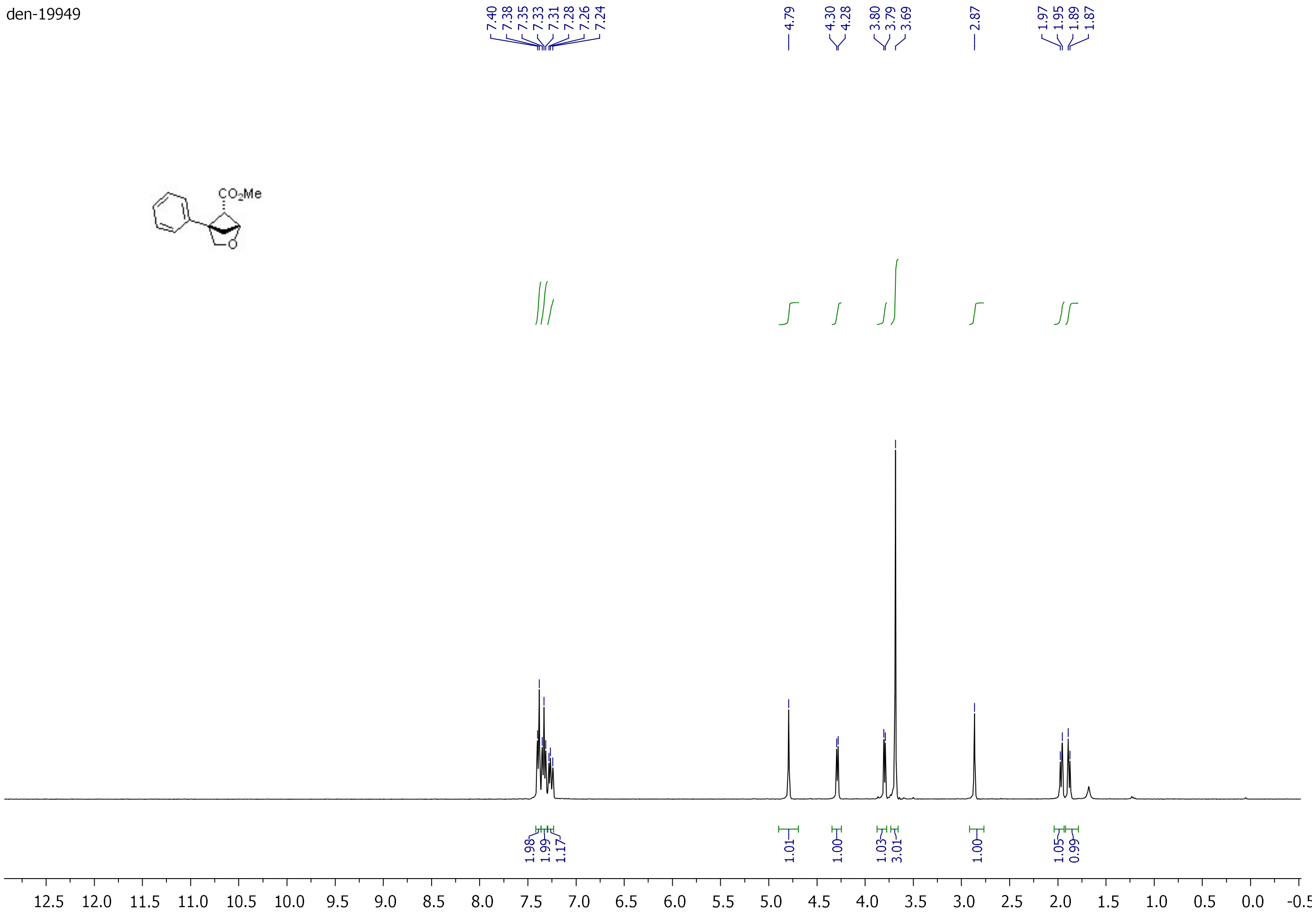

$^{13}C\{^{1}H\}$ NMR (126 MHz, $CDCl_3$)

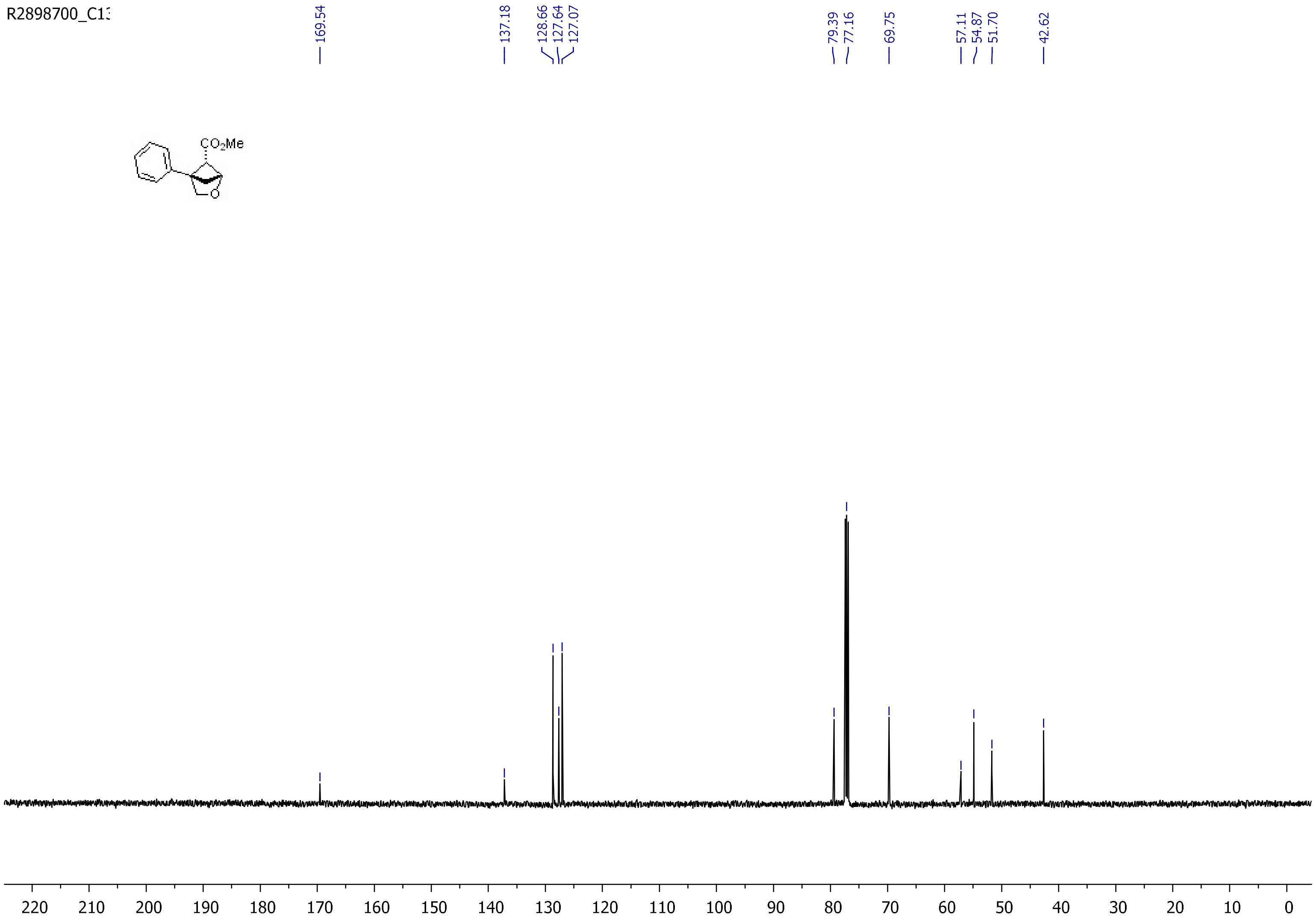

Compound (±)-1b

^{1}H NMR (400 MHz, $CDCl_3$)

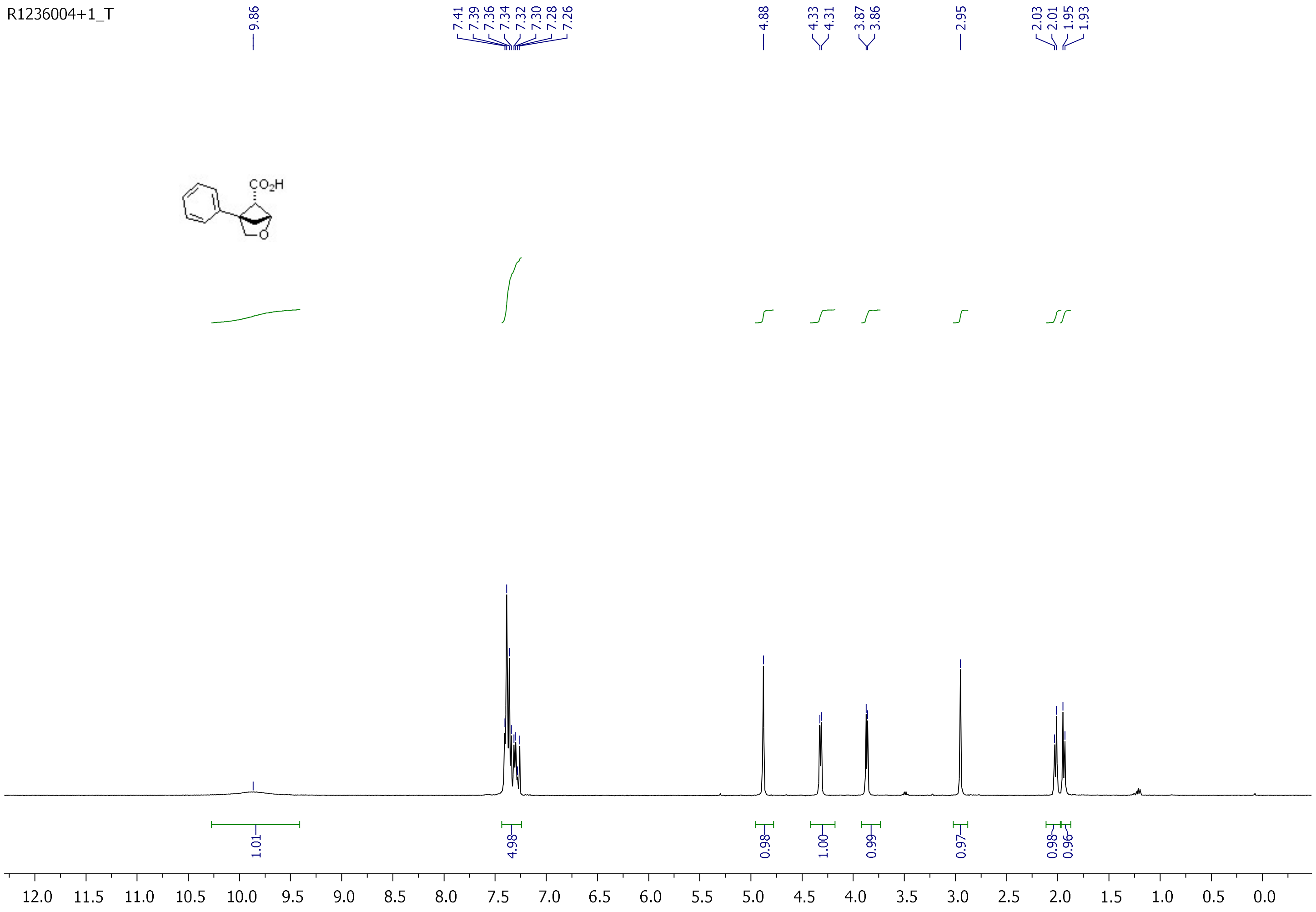

$^{13}C\{^1H\}$ NMR (126 MHz, $CDCl_3$)

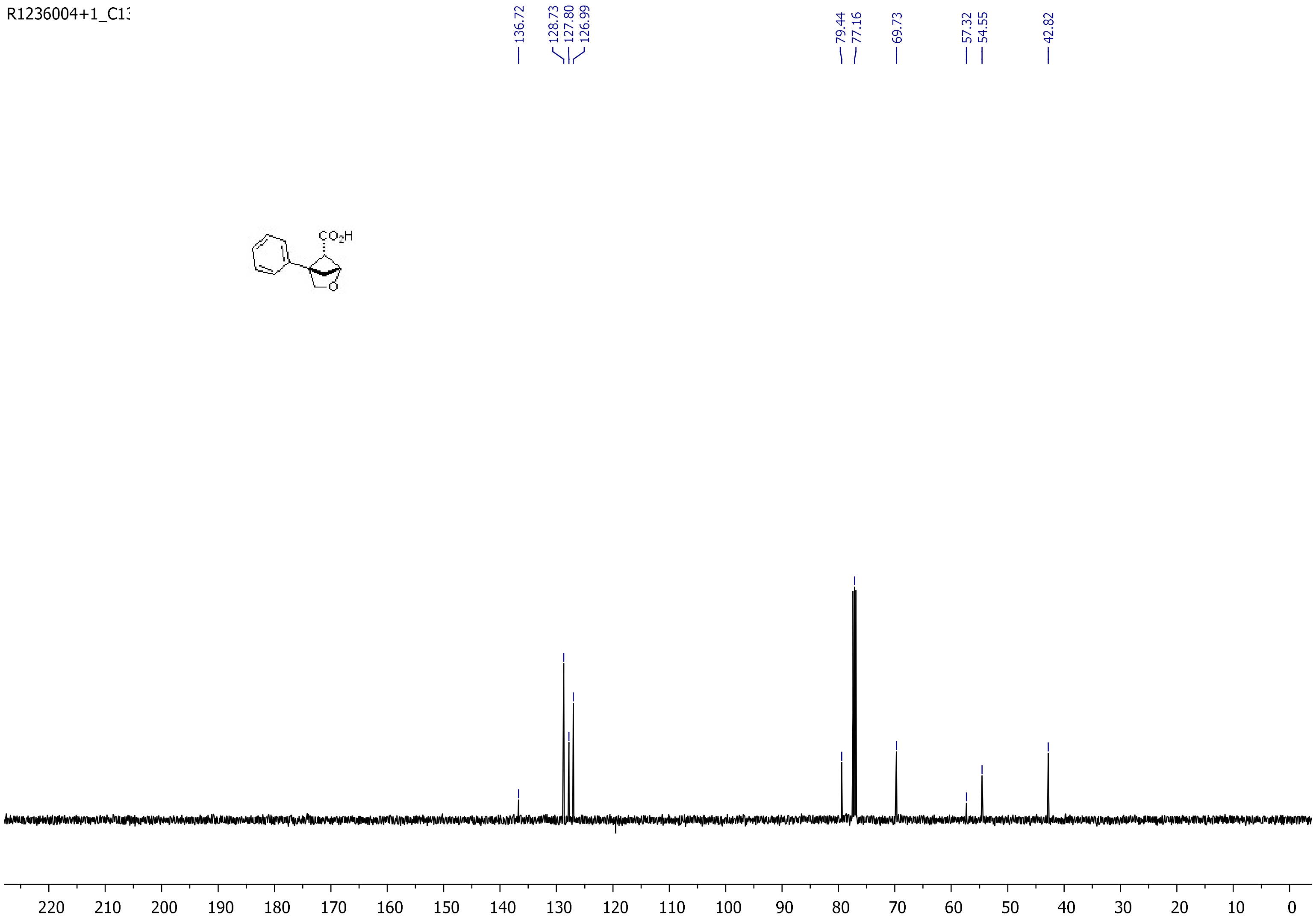

2-(*o*-Tolyl)prop-2-en-1-ol

^{1}H NMR (400 MHz, $CDCl_3$)

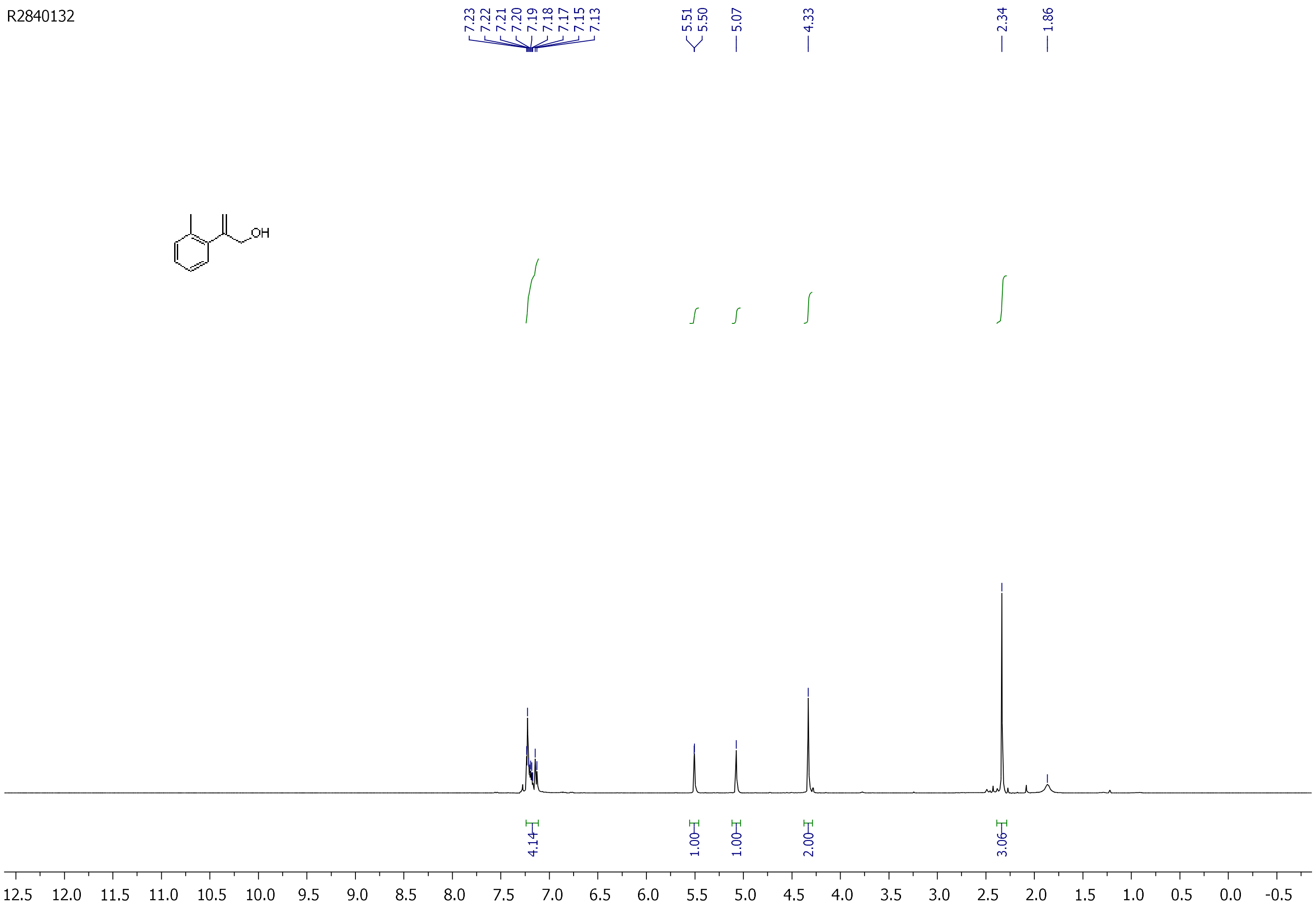

$^{13}C\{^{1}H\}$ NMR (126 MHz, $CDCl_3$)

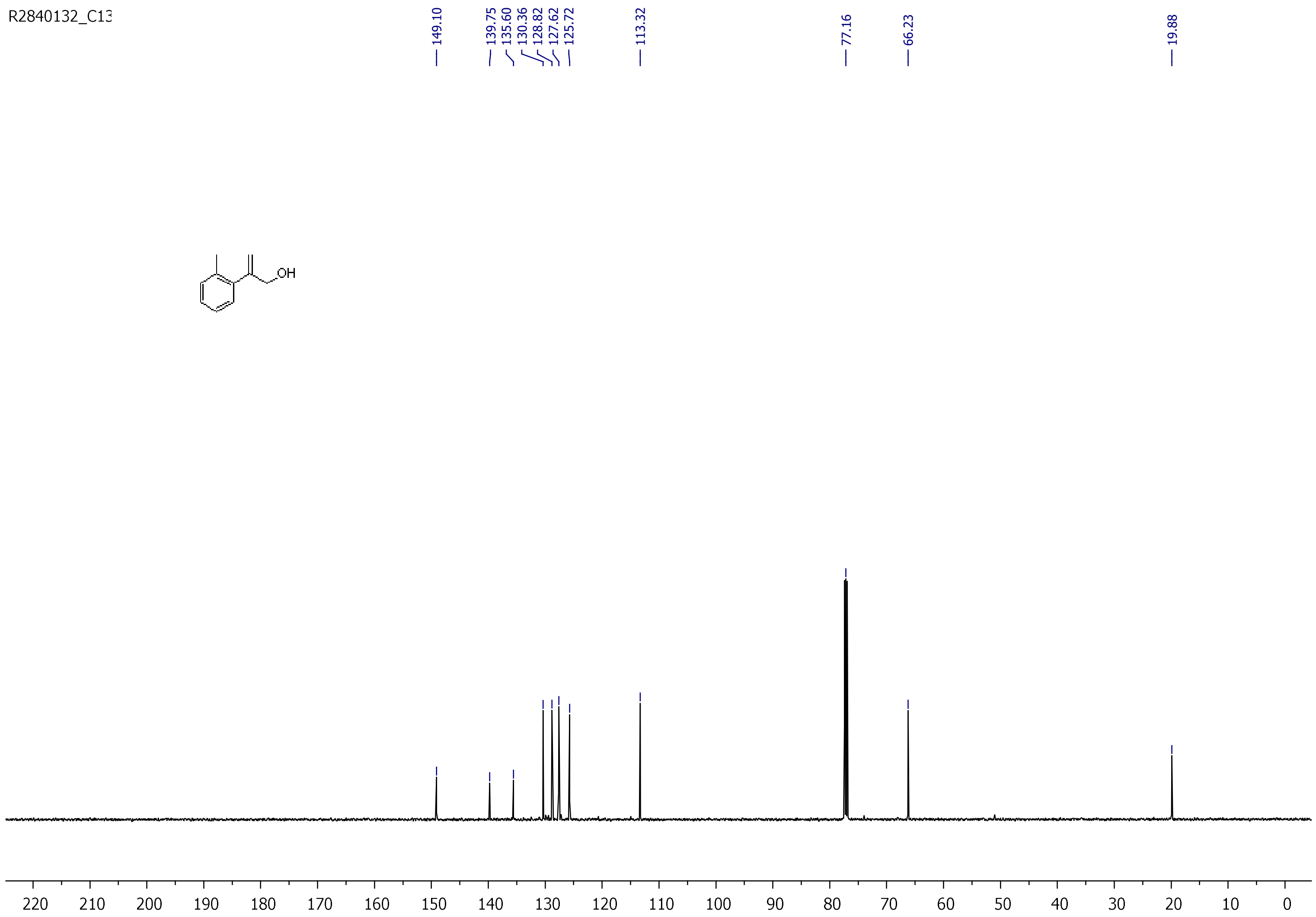

Compound (±)-5a, (d.r. = 5:1)

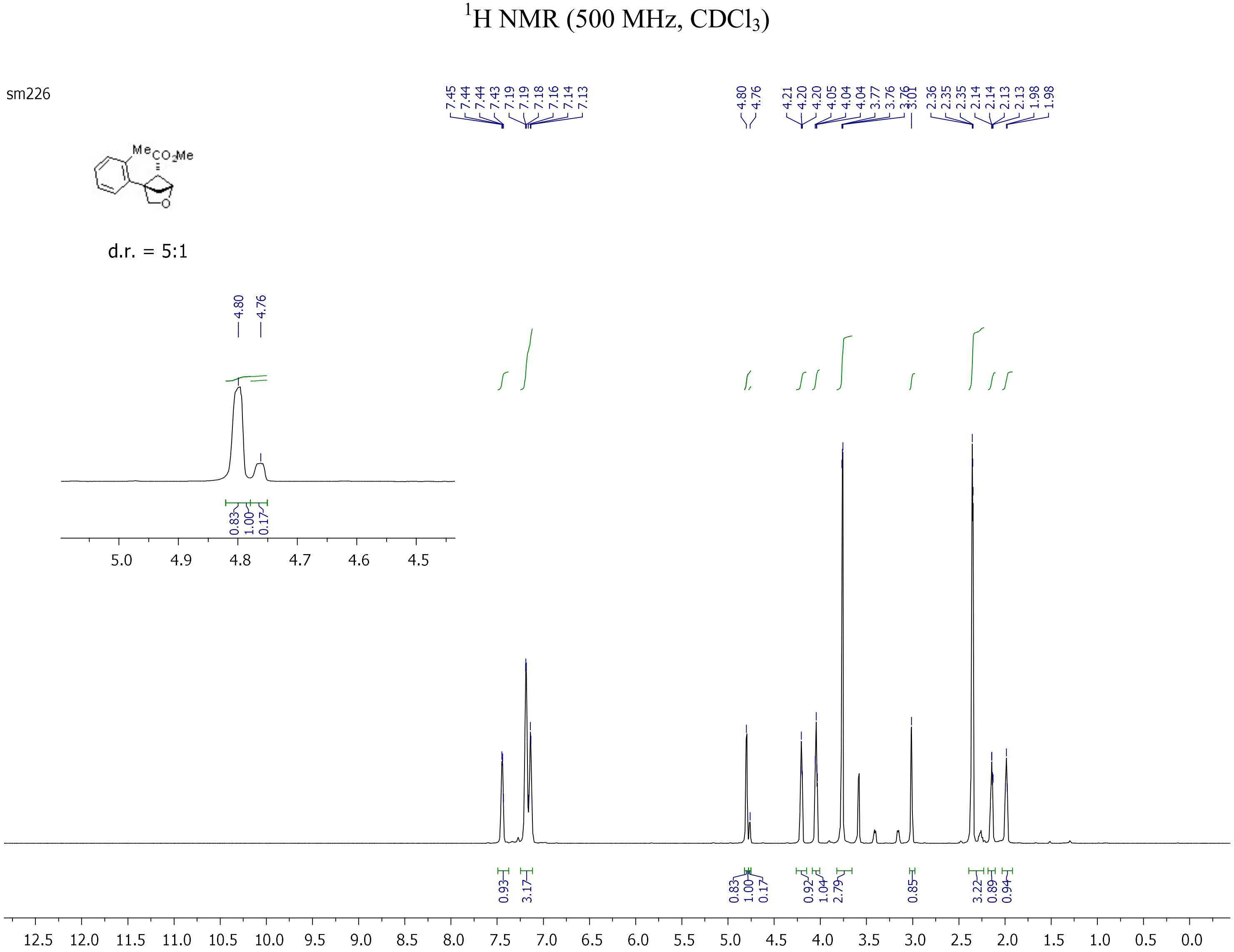

$^{13}C\{^1H\}$ NMR (126 MHz, $CDCl_3$)

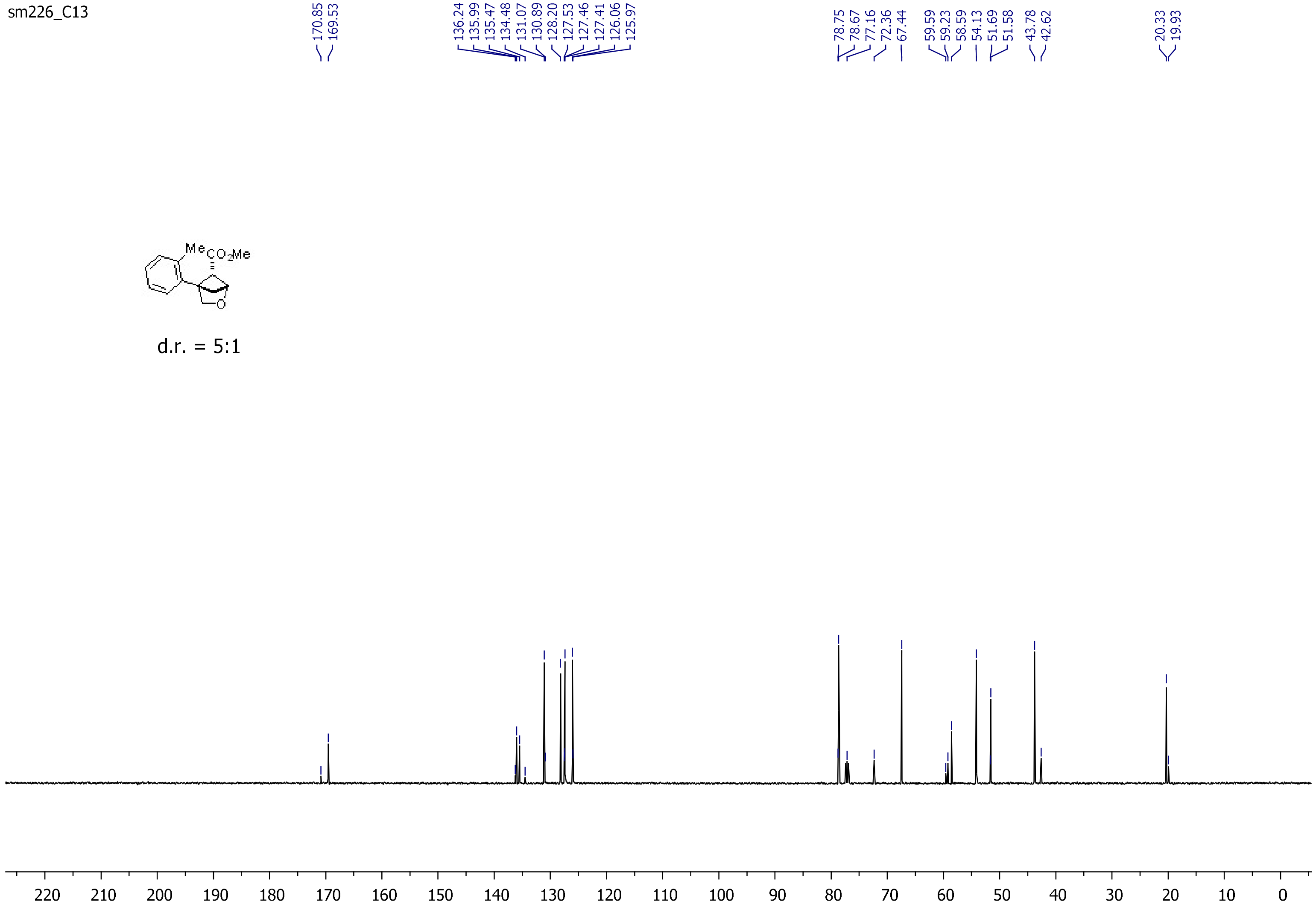

Compound (±)-5b

^{1}H NMR (400 MHz, $CDCl_3$)

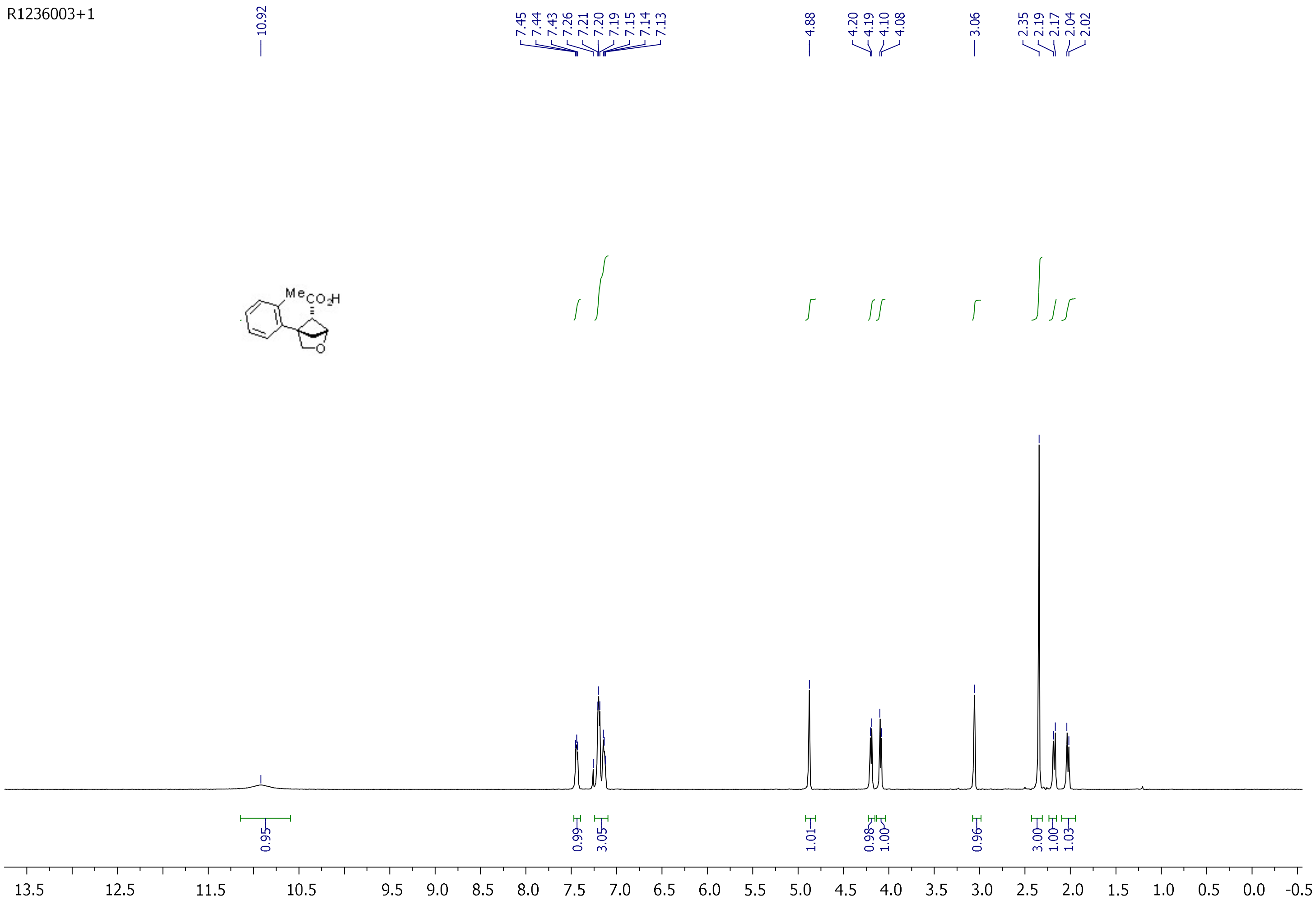

$^{13}C\{^{1}H\}$ NMR (126 MHz, $CDCl_3$)

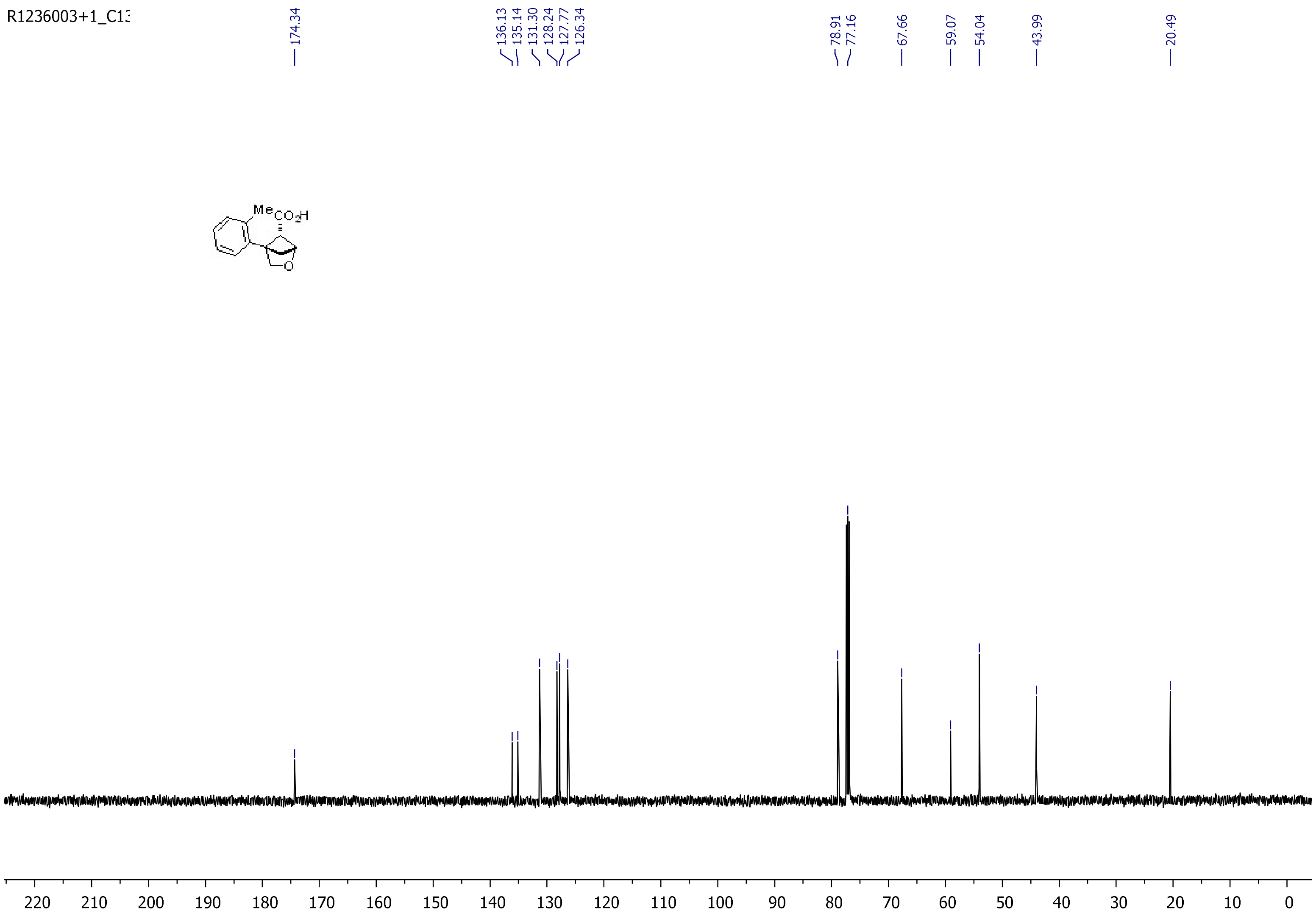

2-(*m*-Tolyl)prop-2-en-1-ol

^{1}H NMR (400 MHz, $CDCl_3$)

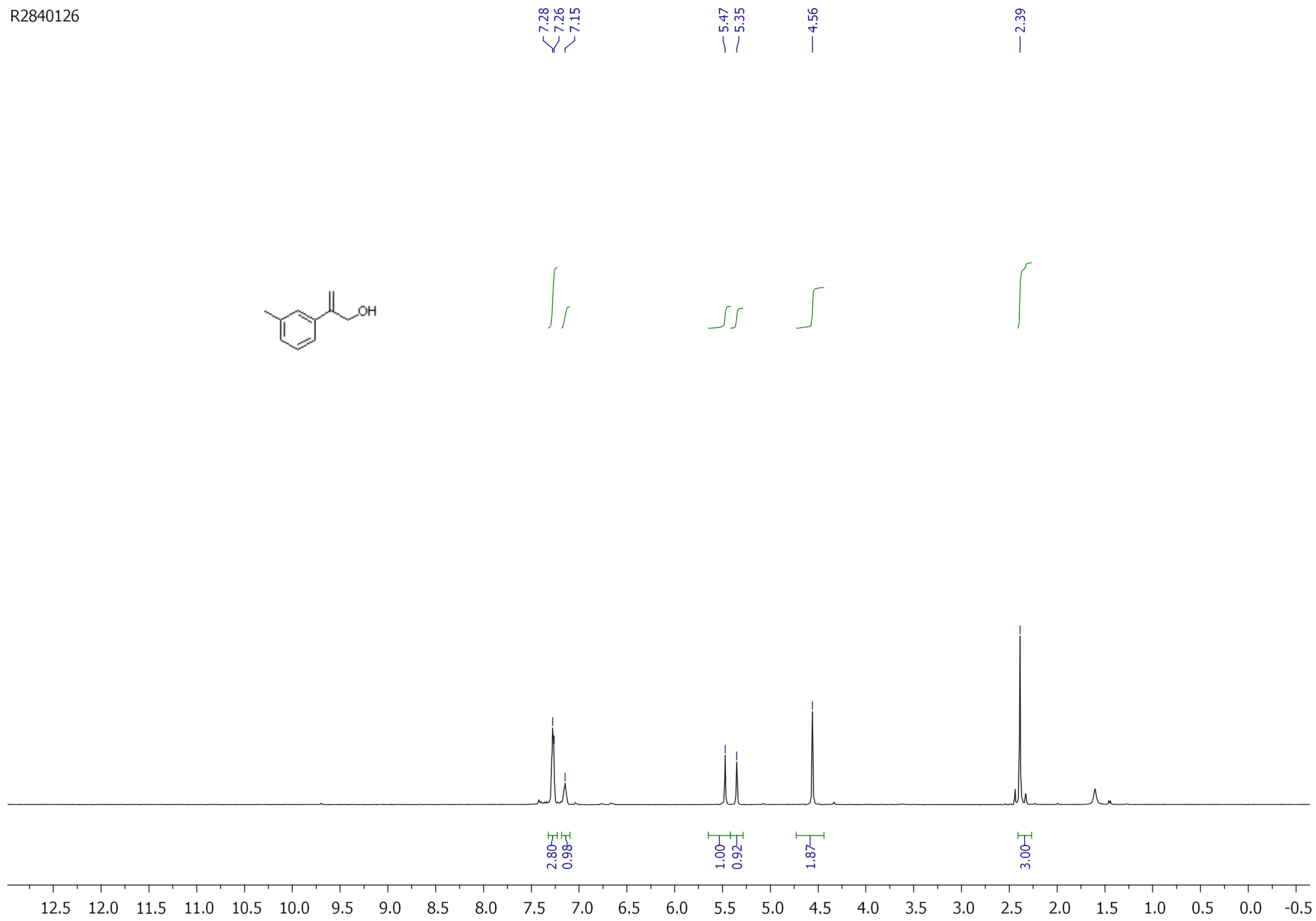

$^{13}C\{^1H\}$ NMR (151 MHz, $CDCl_3$)

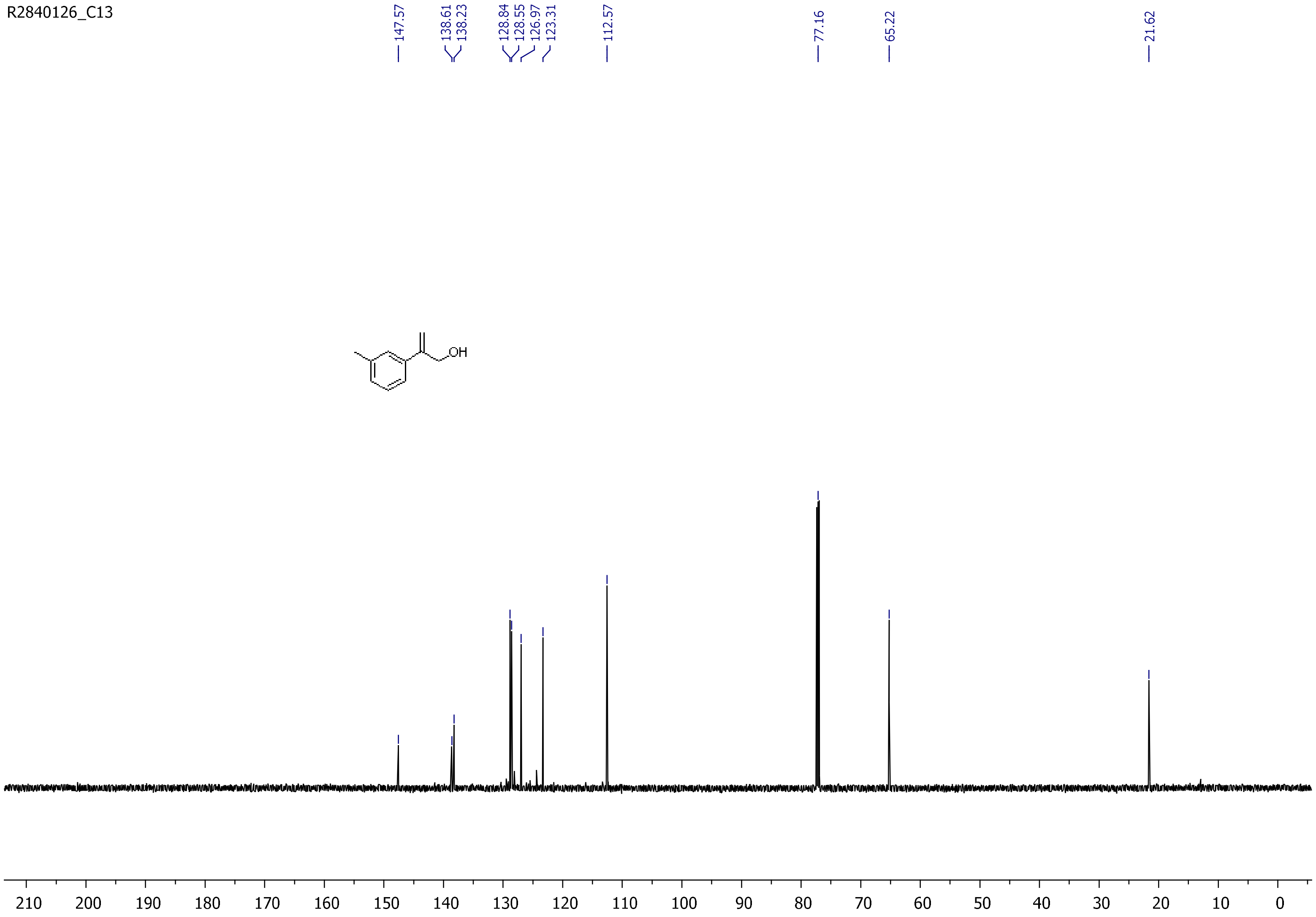

Compound (±)-6a

^{1}H NMR (400 MHz, $CDCl_3$)

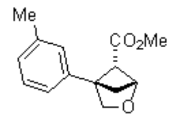

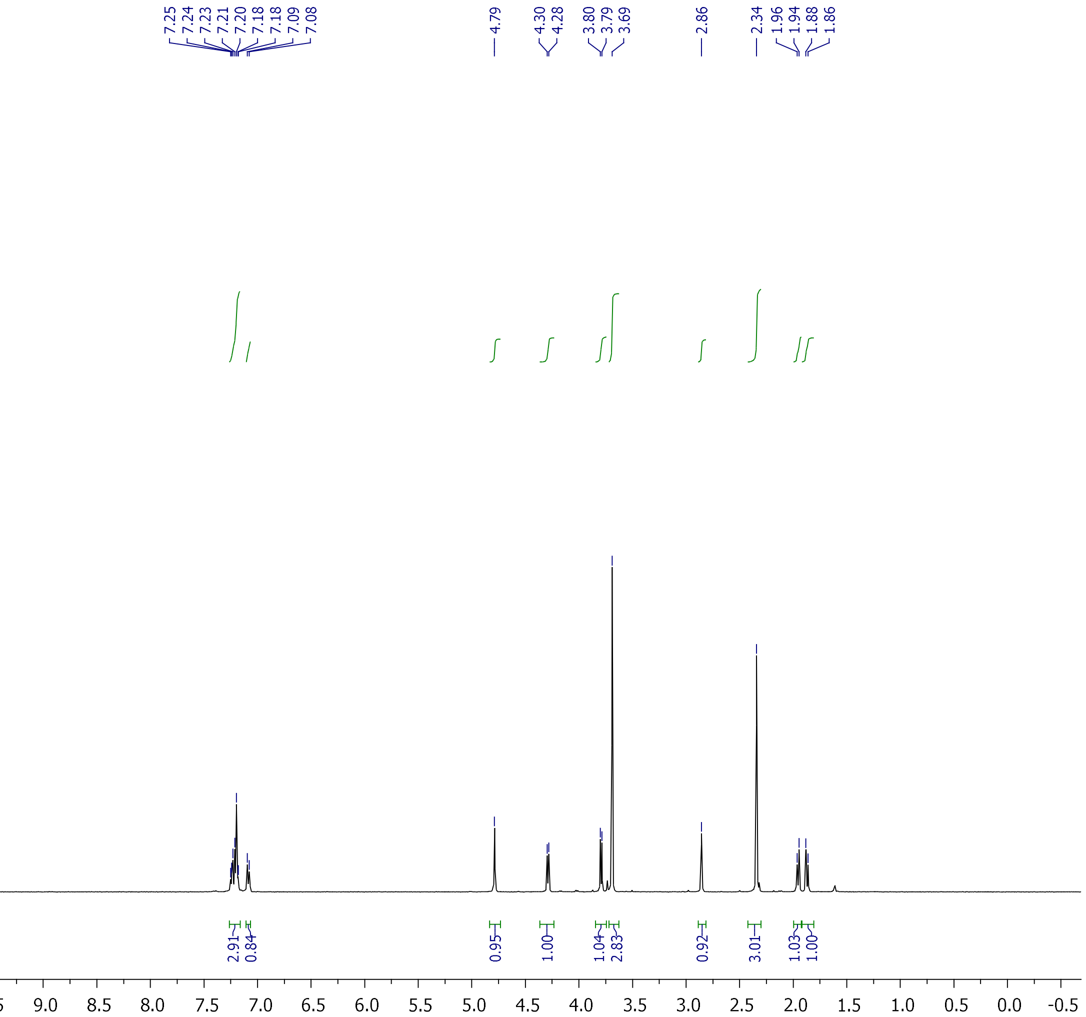

$^{13}C\{^1H\}$ NMR (126 MHz, $CDCl_3$)

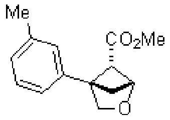

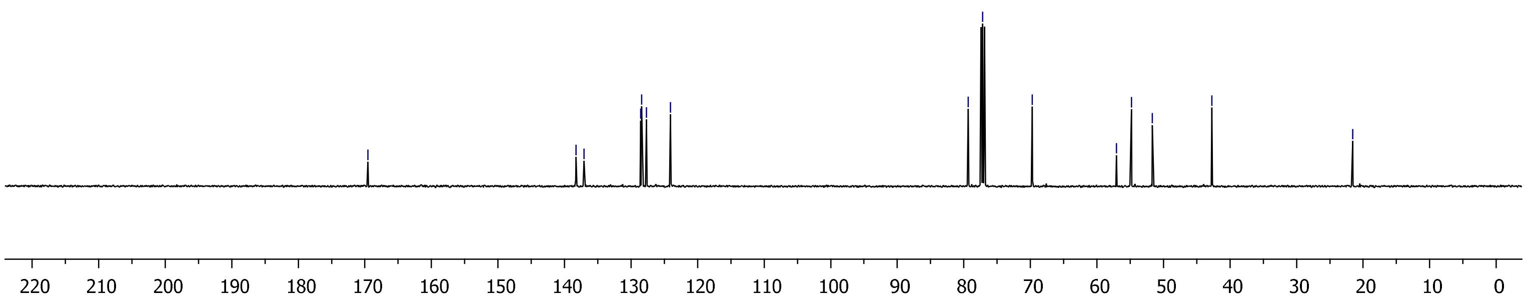

Compound (±)-6b

^{1}H NMR (400 MHz, DMSO-d$_6$)

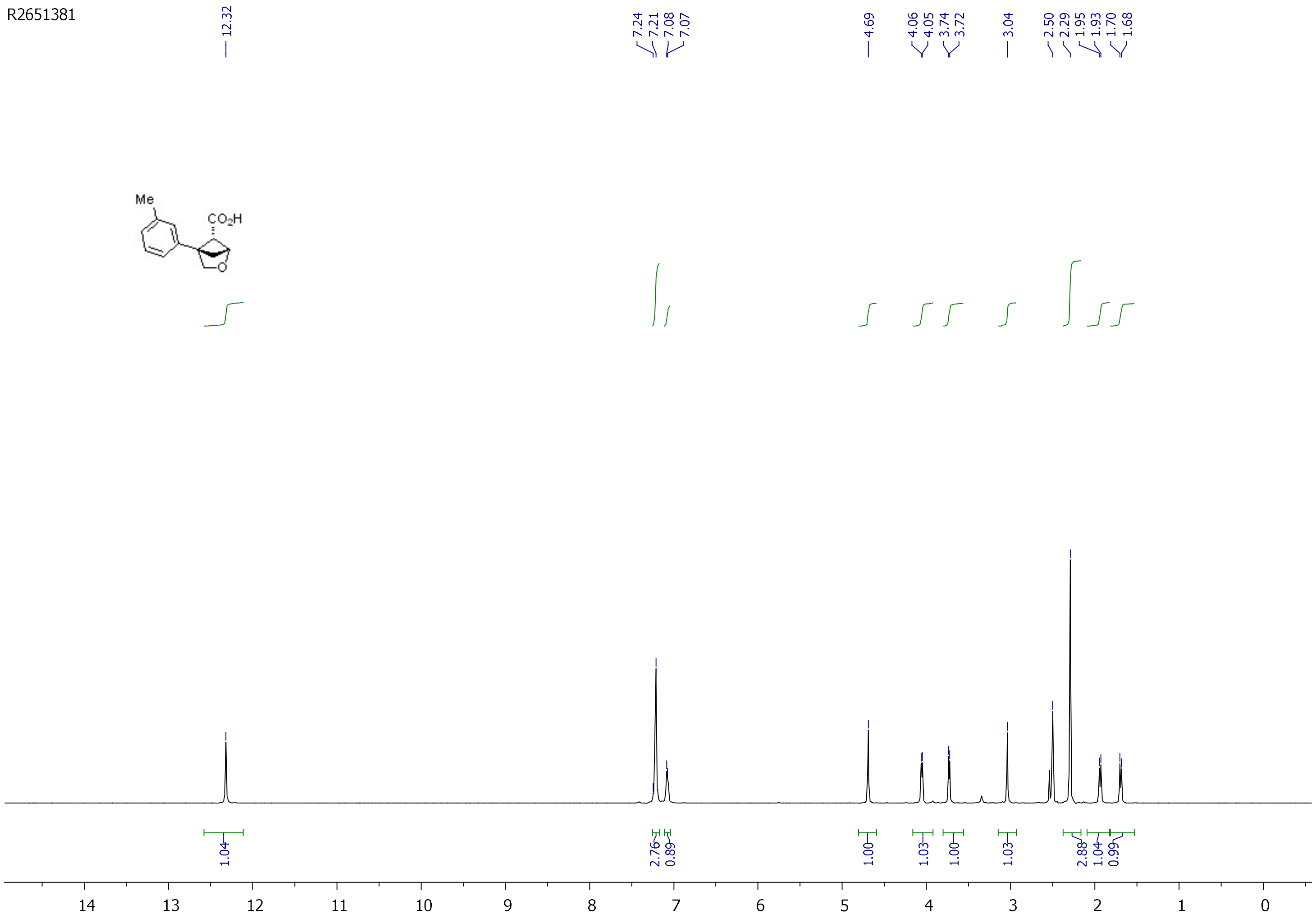

$^{13}C\{^{1}H\}$ NMR (126 MHz, DMSO-d_6)

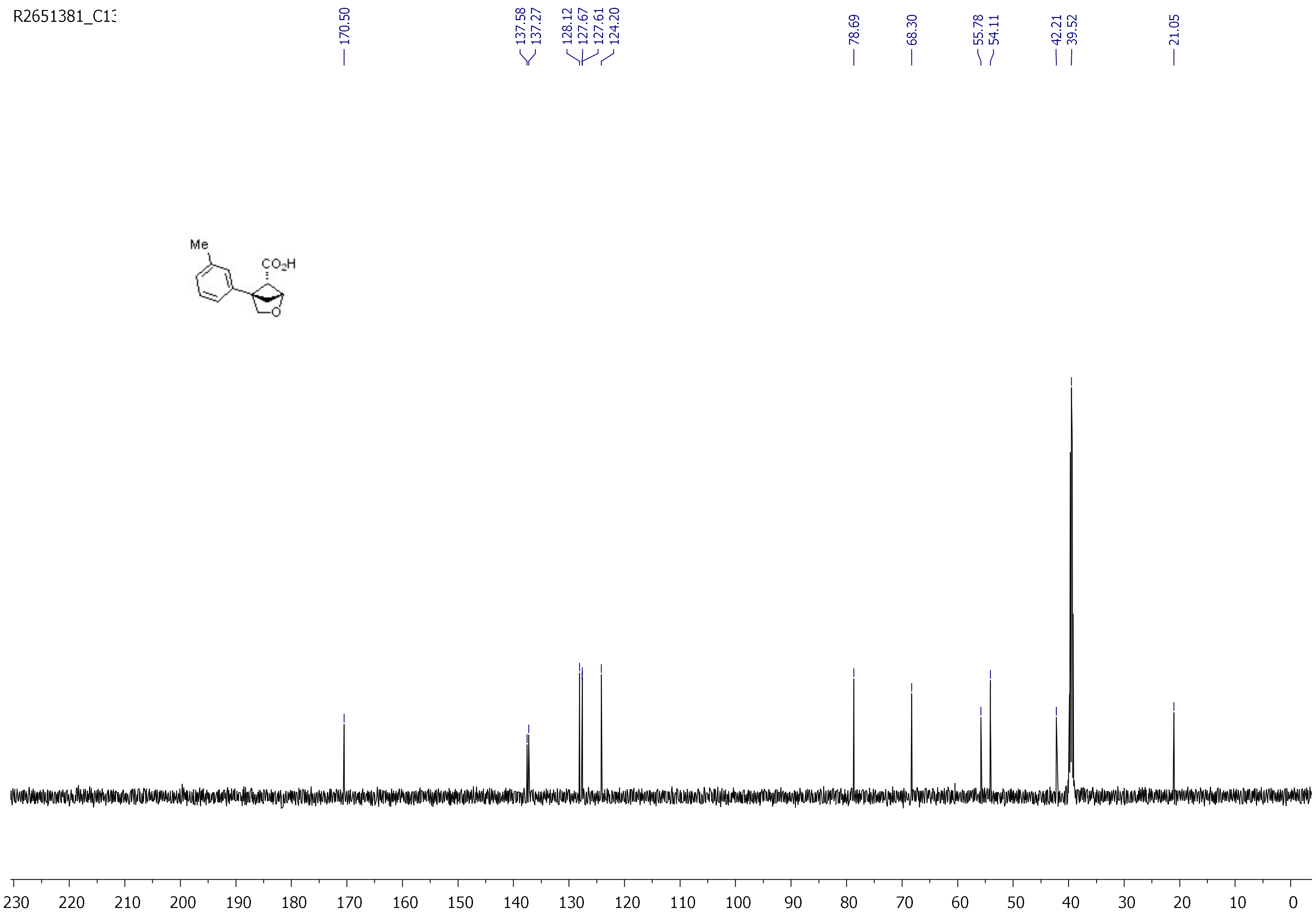

2-(*p*-Tolyl)prop-2-en-1-ol

^{1}H NMR (400 MHz, $CDCl_3$)

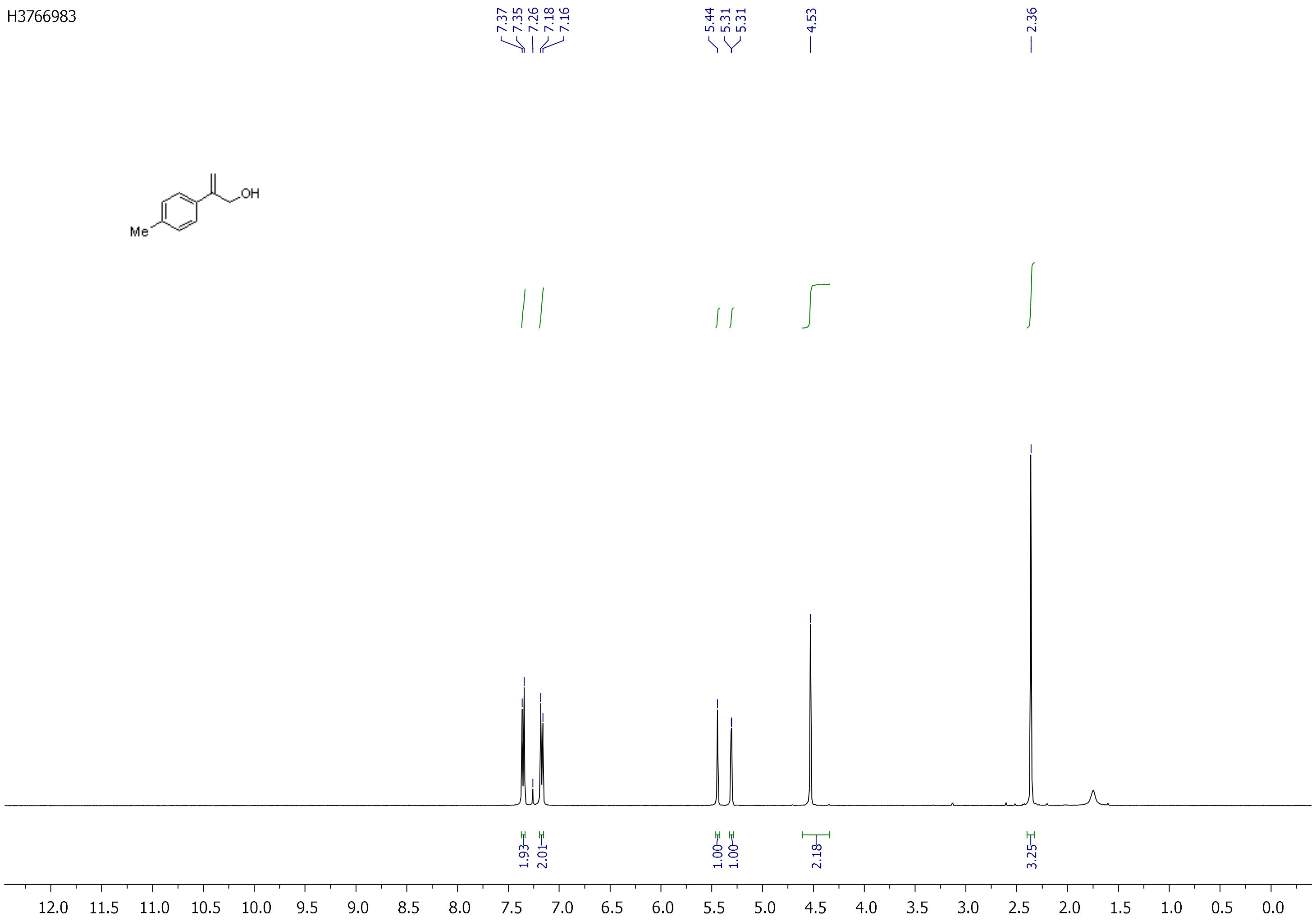

$^{13}C\{^{1}H\}$ NMR (126 MHz, $CDCl_3$)

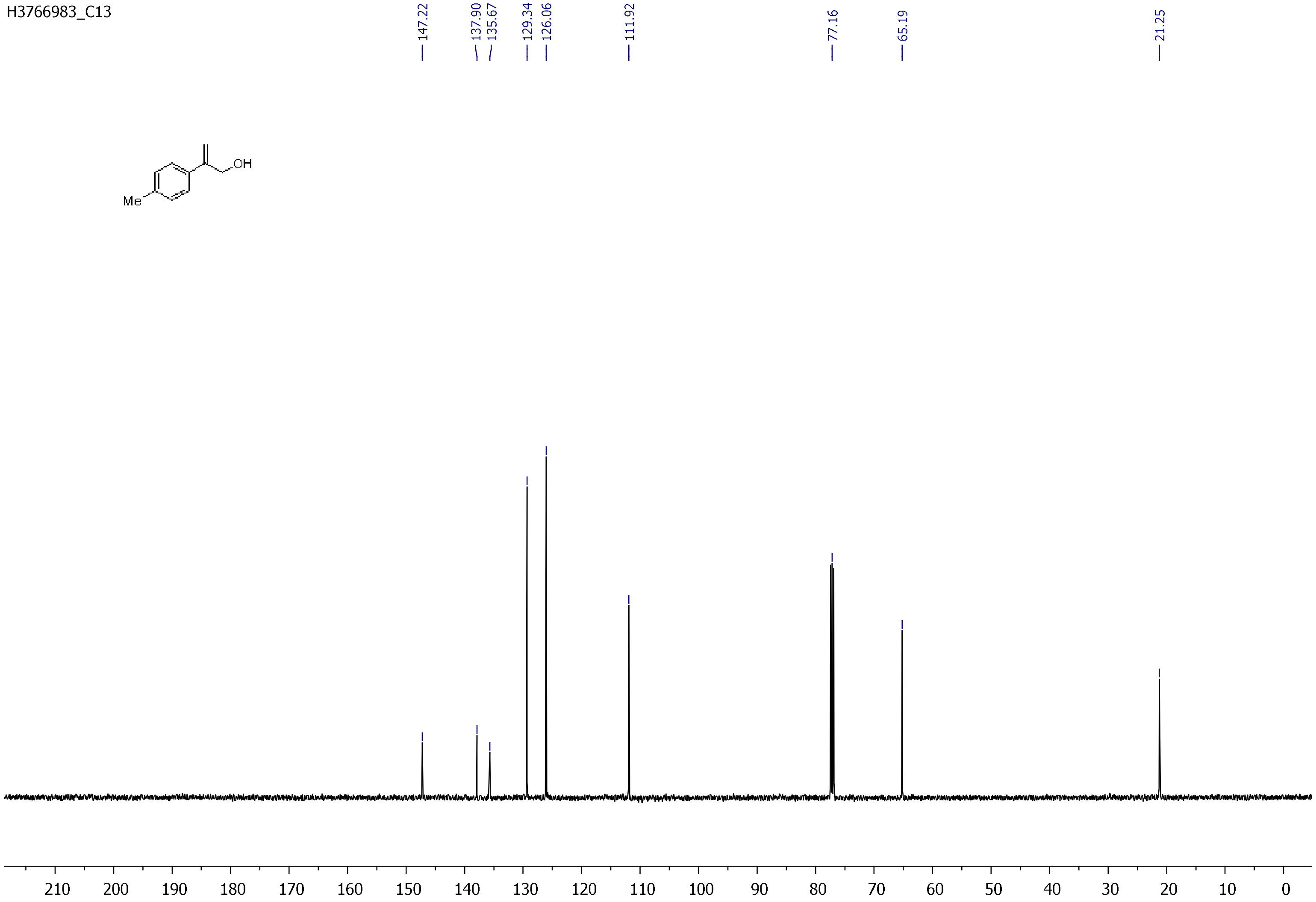

Compound (±)-7a, d.r. = 3:2

^{1}H NMR (400 MHz, $CDCl_3$)

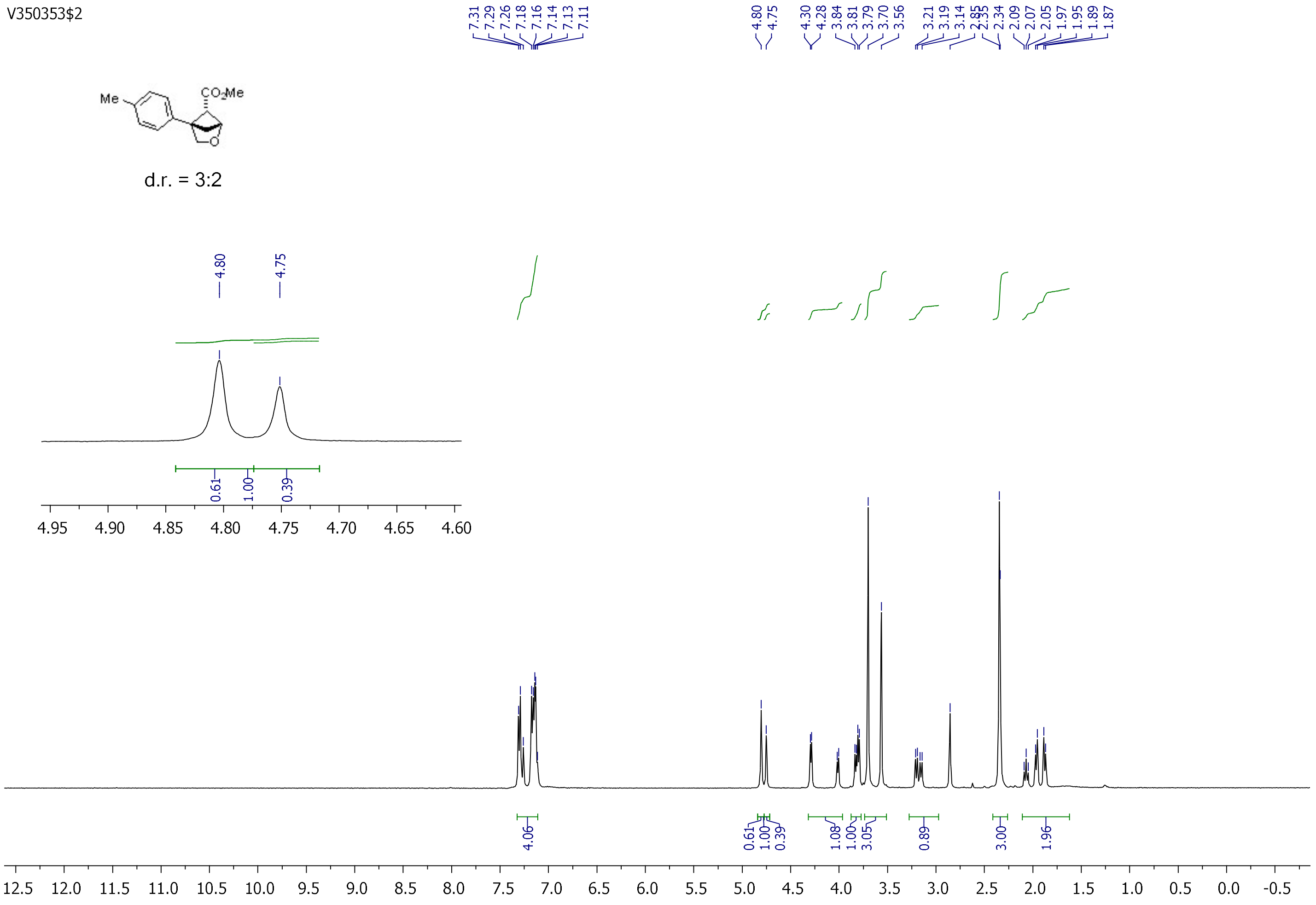

$^{13}C\{^1H\}$ NMR (151 MHz, $CDCl_3$)

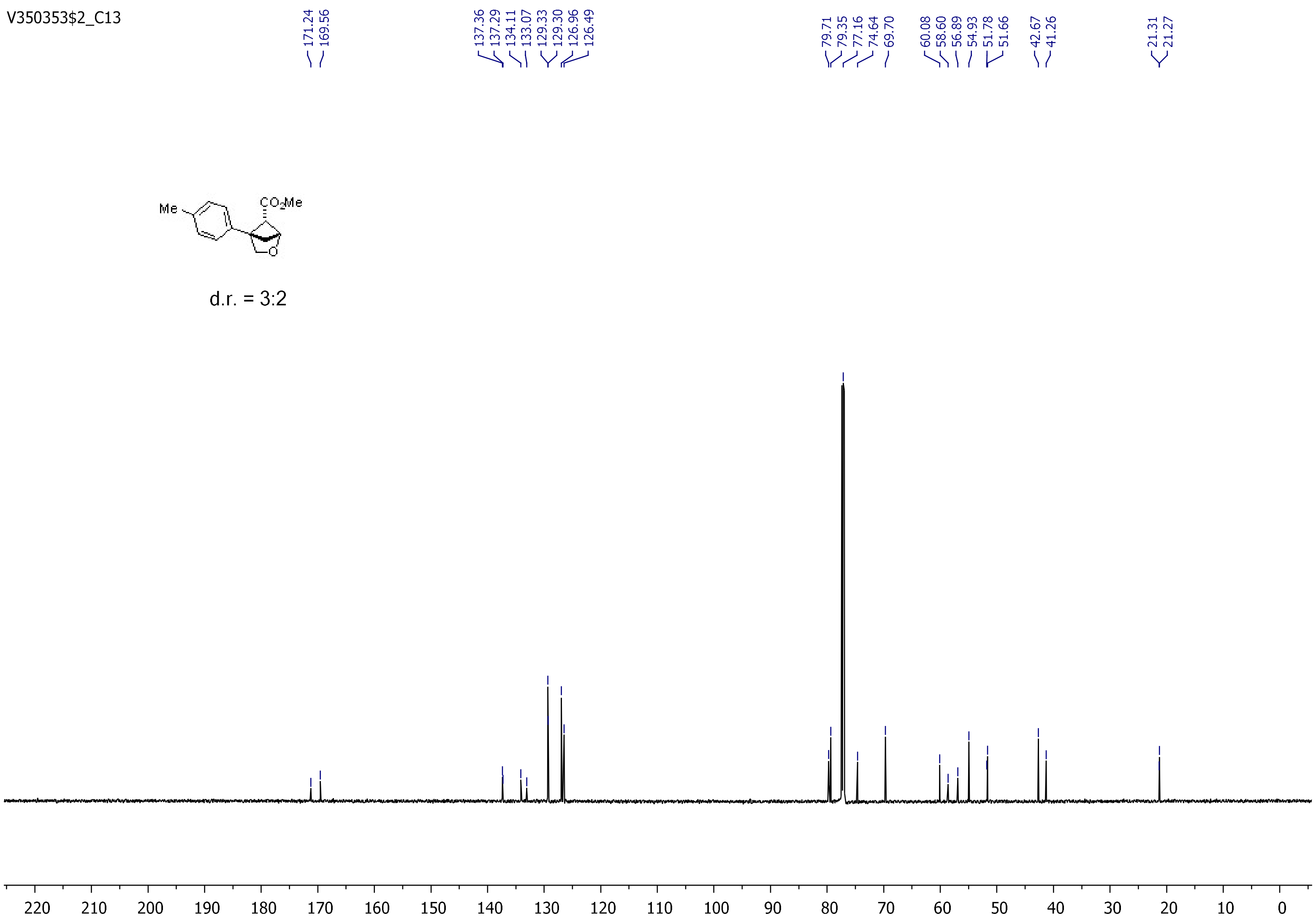
V350353$2_C13
171.24
169.56
137.36
137.29
134.11
133.07
129.33
129.30
126.96
126.49
79.71
79.35
77.16
74.64
69.70
60.08
58.60
56.89
54.93
51.78
51.66
42.67
41.26
21.31
21.27
Me
CO_2Me
O
d.r. = 3:2
220 210 200 190 180 170 160 150 140 130 120 110 100 90 80 70 60 50 40 30 20 10 0

Compound (±)-7b

^{1}H NMR (400 MHz, DMSO-d$_6$)

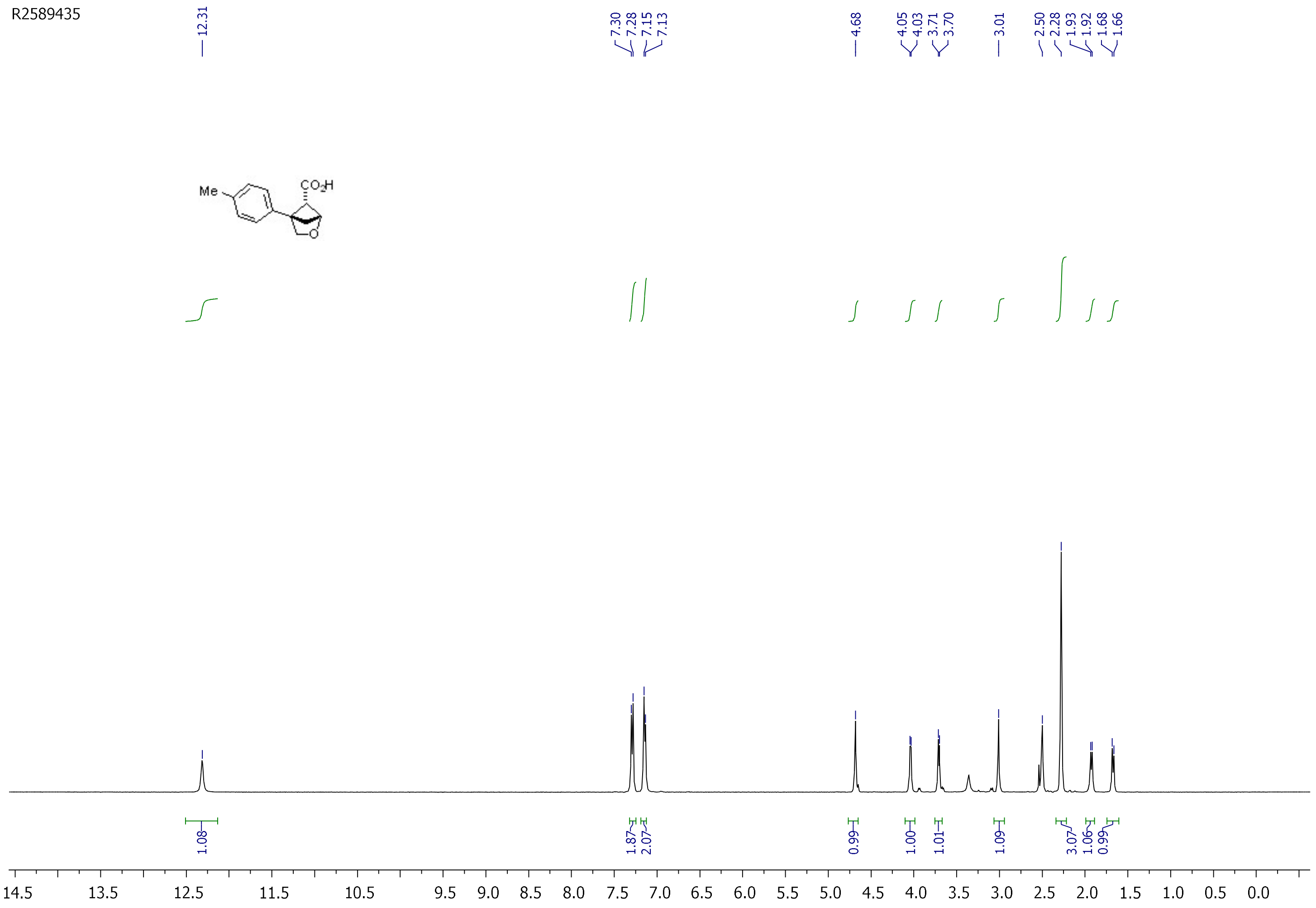

$^{13}C\{^1H\}$ NMR (126 MHz, DMSO-d$_6$)

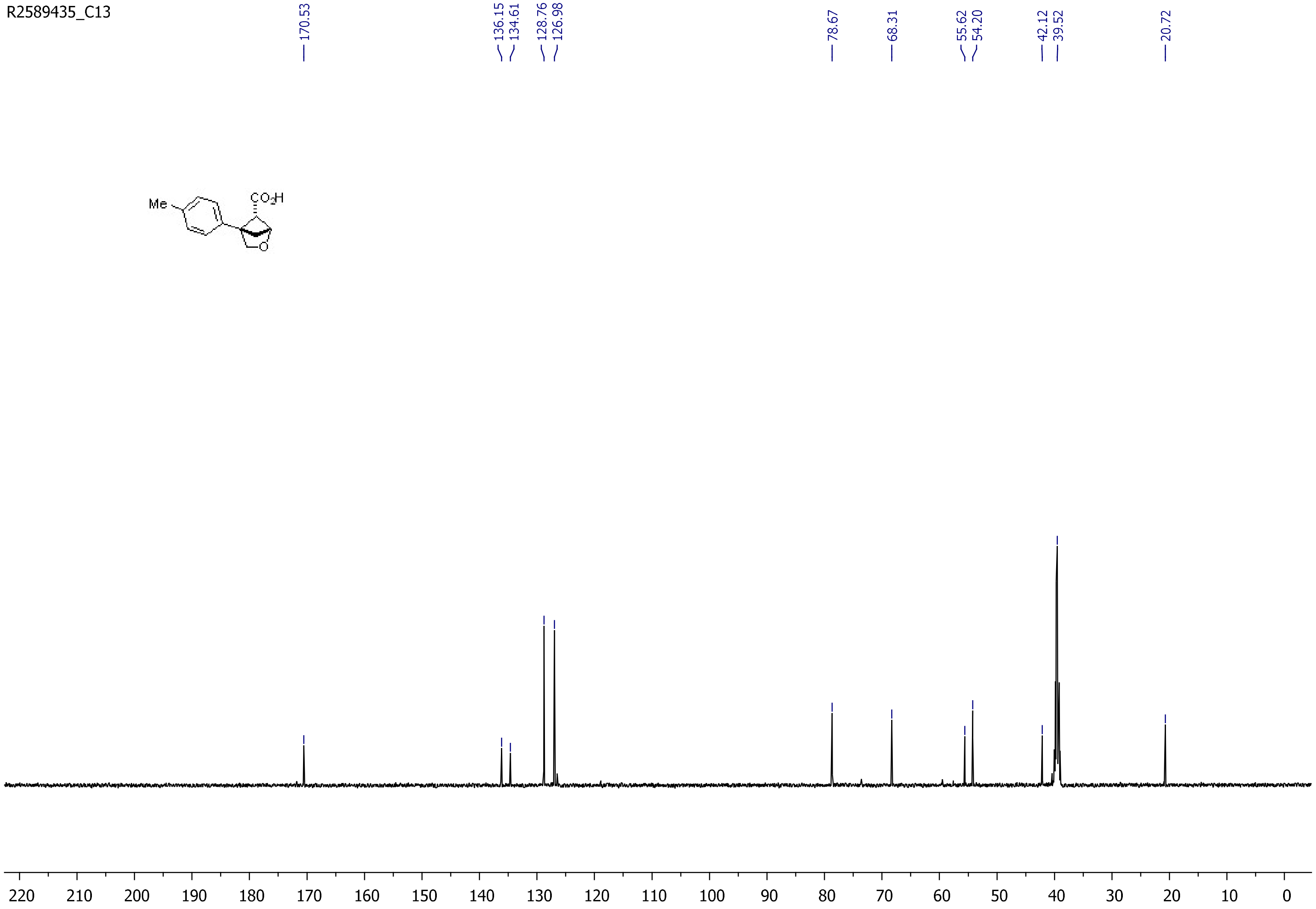

2-(3-(Tert-butyl)phenyl)prop-2-en-1-ol

^{1}H NMR (400 MHz, $CDCl_3$)

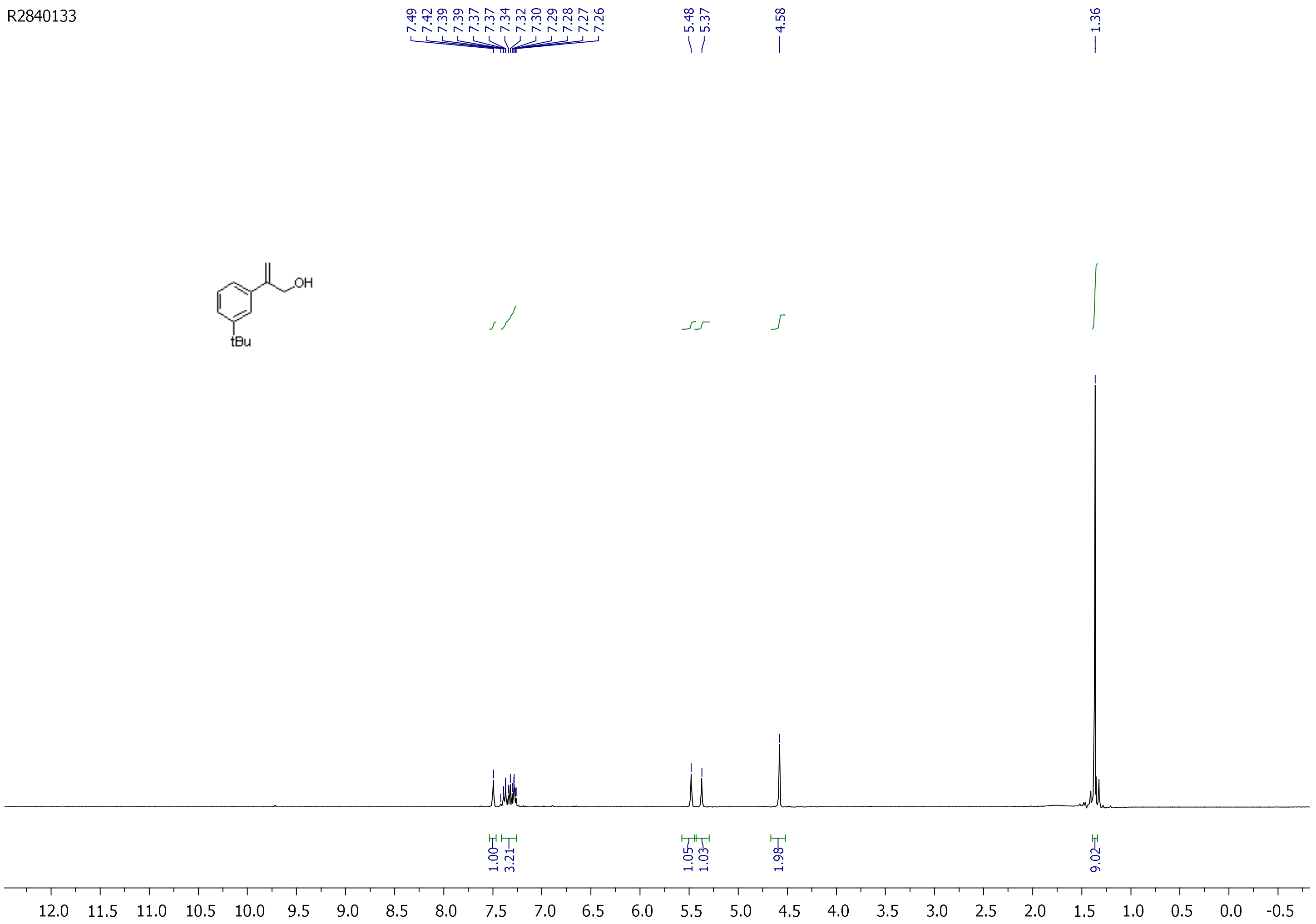

$^{13}C\{^{1}H\}$ NMR (151 MHz, $CDCl_3$)

R2840133_C13

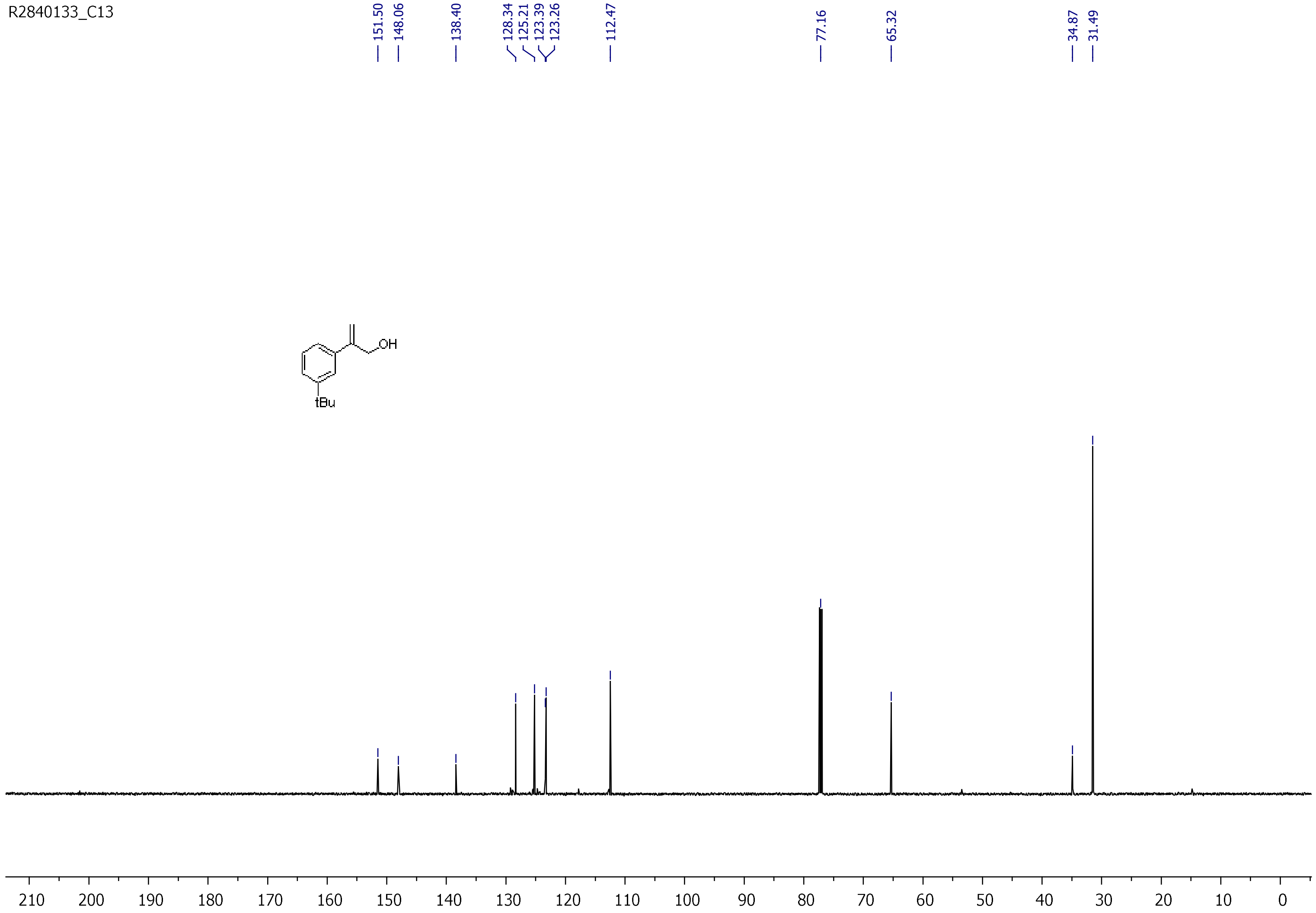

Compound (±)-8a

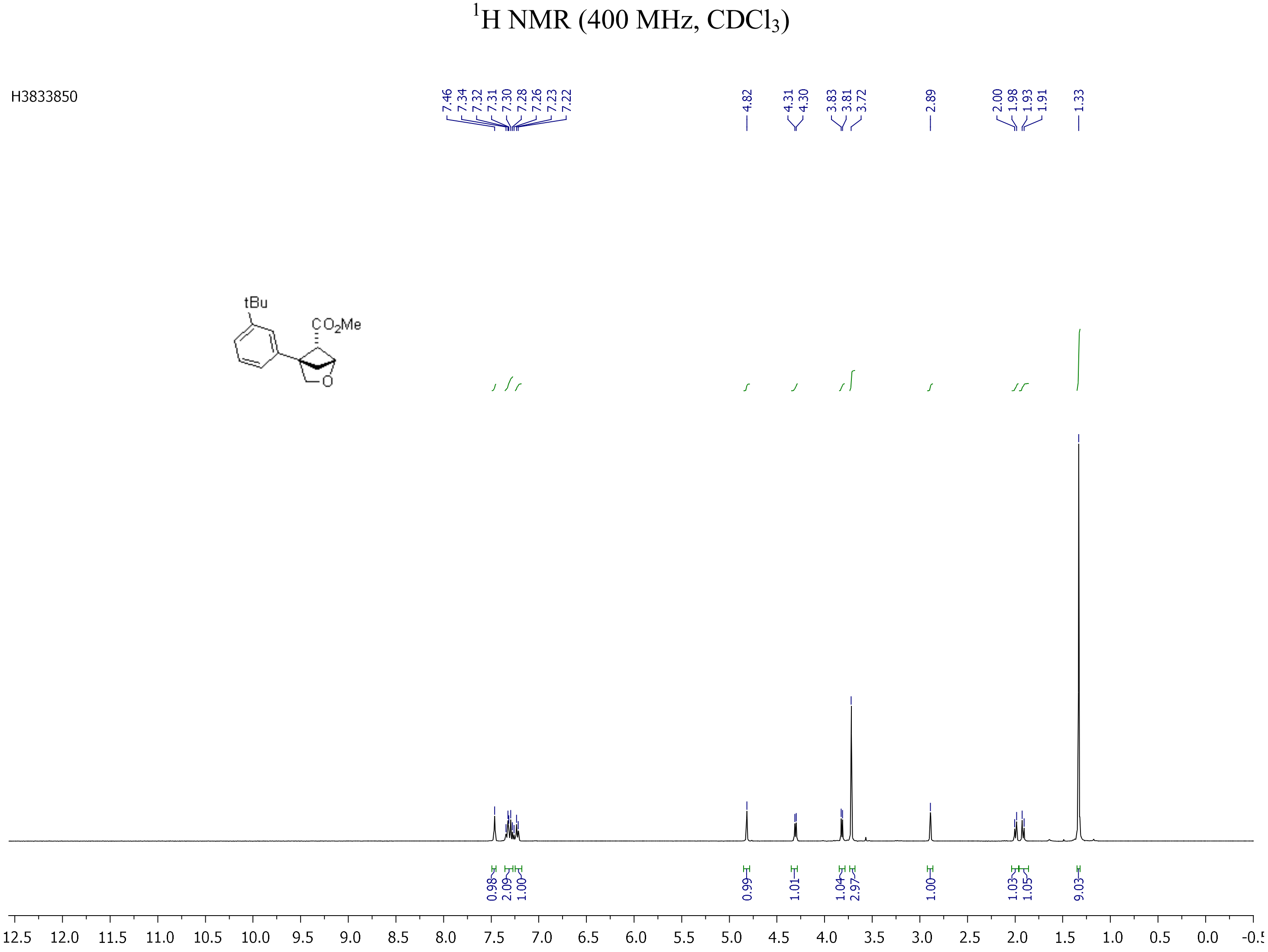

1H NMR (400 MHz, CDCl3)
H3833850
tBu
CO2Me
O
7.46
7.34
7.32
7.31
7.30
7.28
7.26
7.23
7.22
4.82
4.31
4.30
3.83
3.81
3.72
2.89
2.00
1.98
1.93
1.91
1.33
0.98
2.09
1.00
0.99
1.01
1.04
2.97
1.00
1.03
1.05
9.03
12.5 12.0 11.5 11.0 10.5 10.0 9.5 9.0 8.5 8.0 7.5 7.0 6.5 6.0 5.5 5.0 4.5 4.0 3.5 3.0 2.5 2.0 1.5 1.0 0.5 0.0 -0.5

$^{13}C\{^1H\}$ NMR (126 MHz, $CDCl_3$)

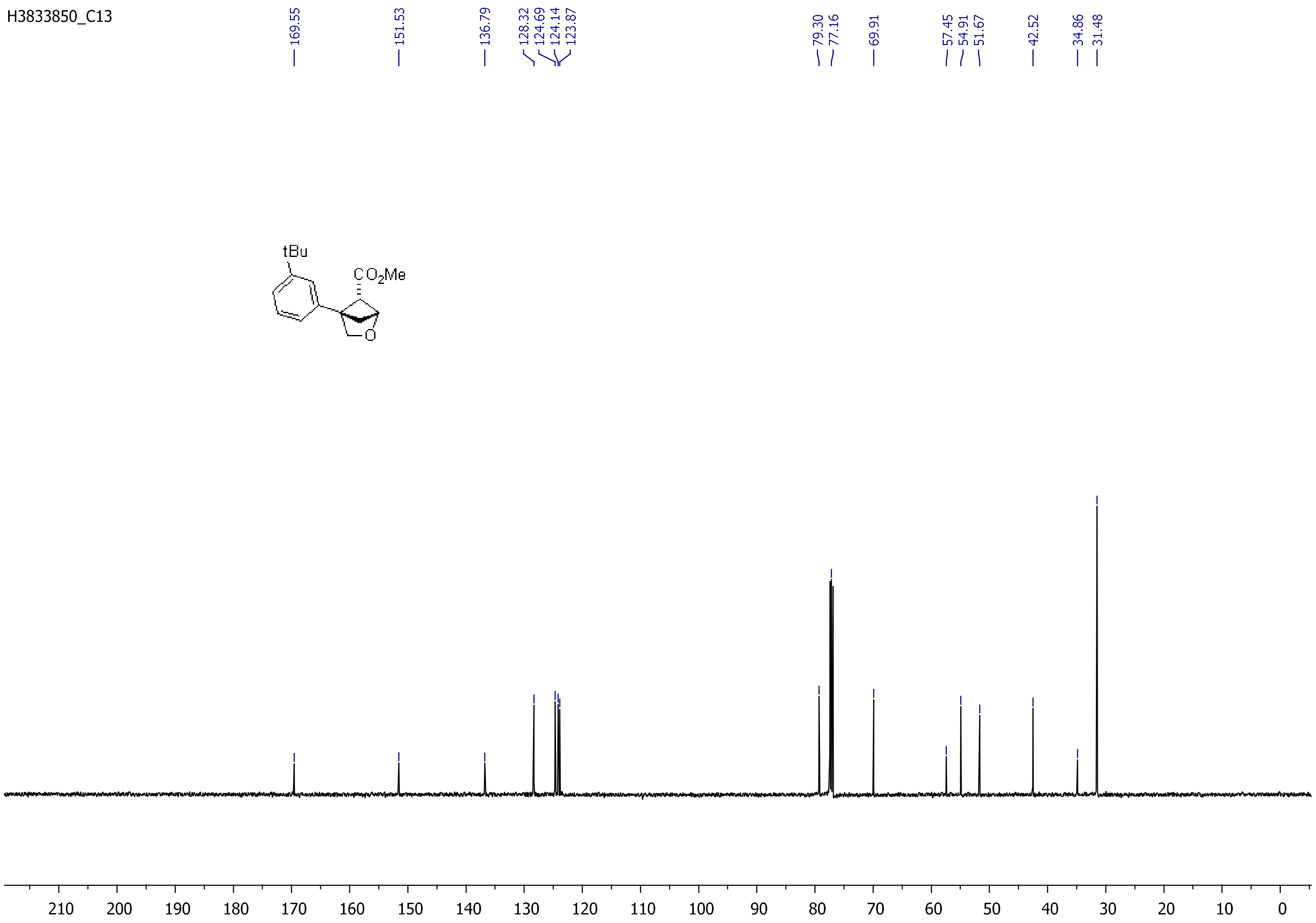

Compound (±)-8b

^{1}H NMR (400 MHz, DMSO-d$_6$)

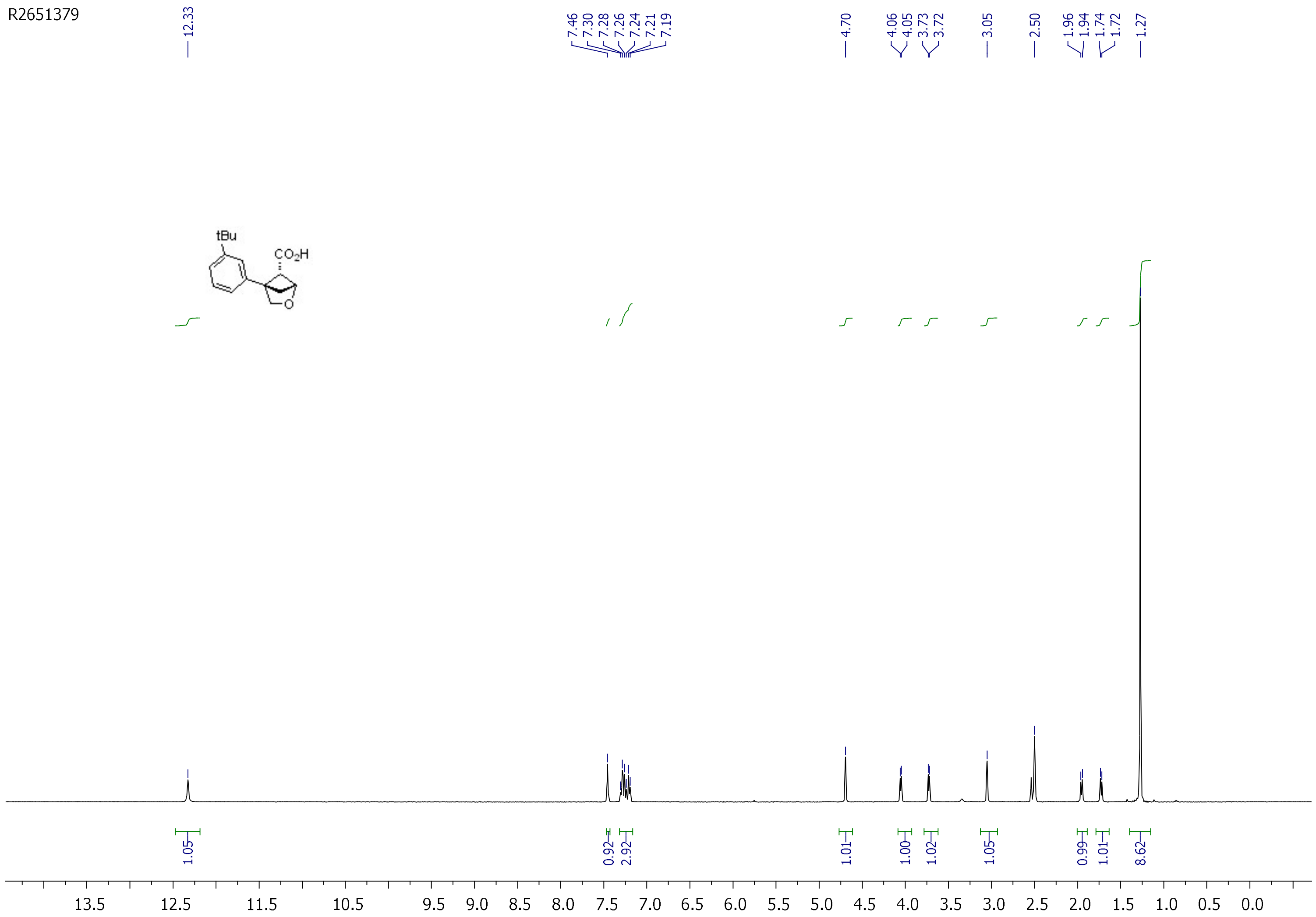

$^{13}C\{^{1}H\}$ NMR (126 MHz, DMSO-d_6)

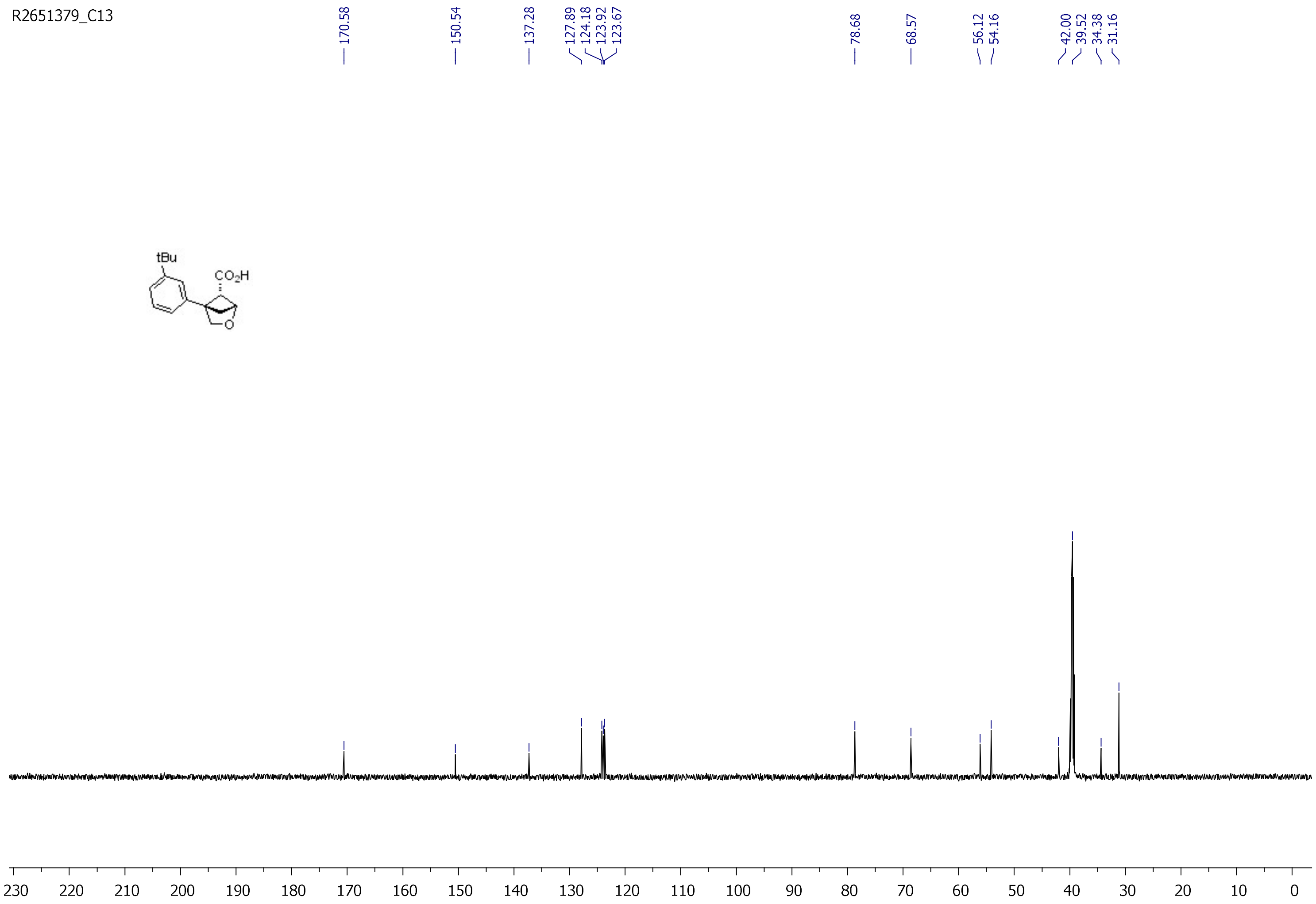

2-(3-Fluorophenyl)prop-2-en-1-ol, (ca. 90% purity)

^{1}H NMR (400 MHz, $CDCl_3$)

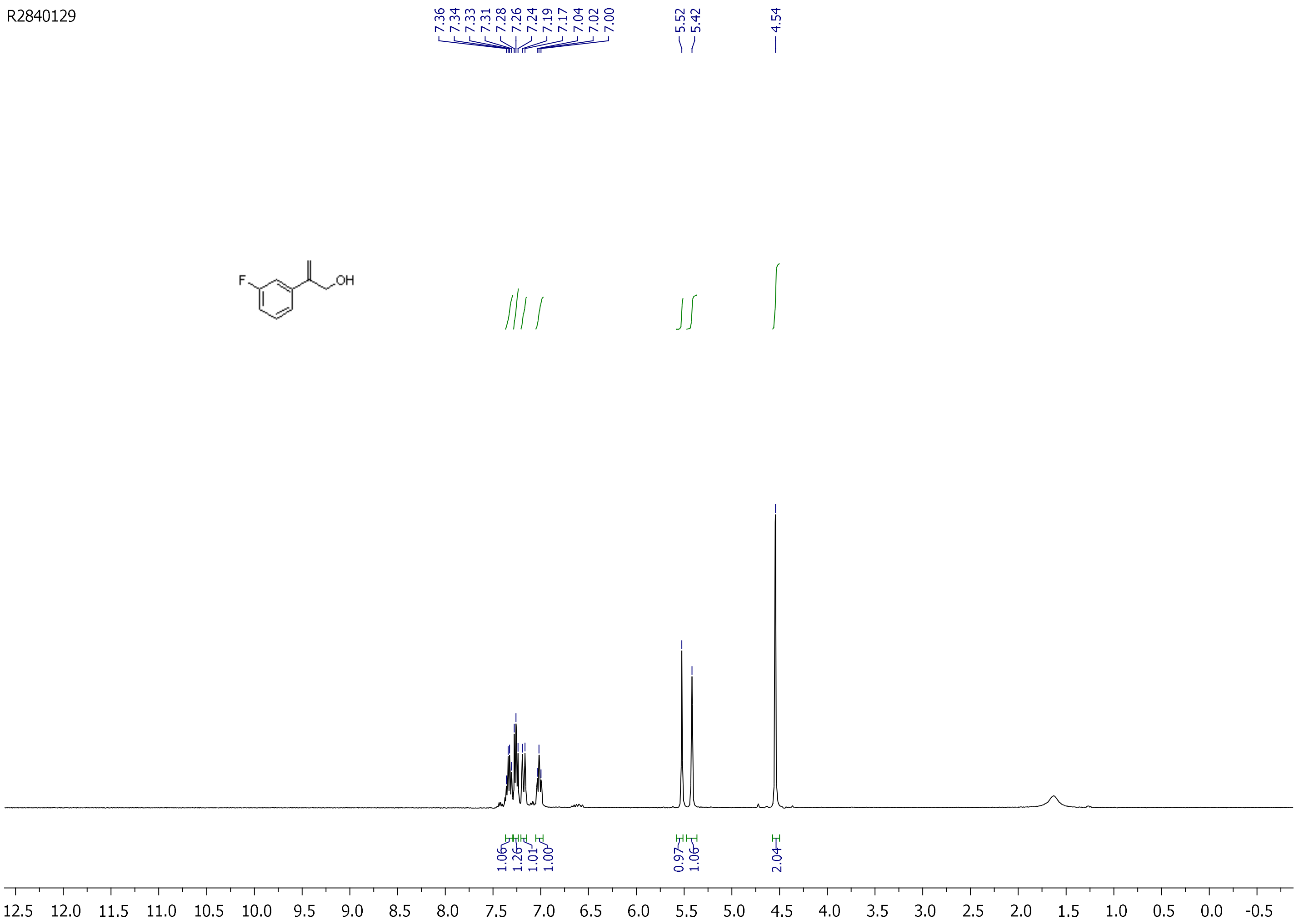

$^{13}C\{^{1}H\}$ NMR (151 MHz, $CDCl_3$)

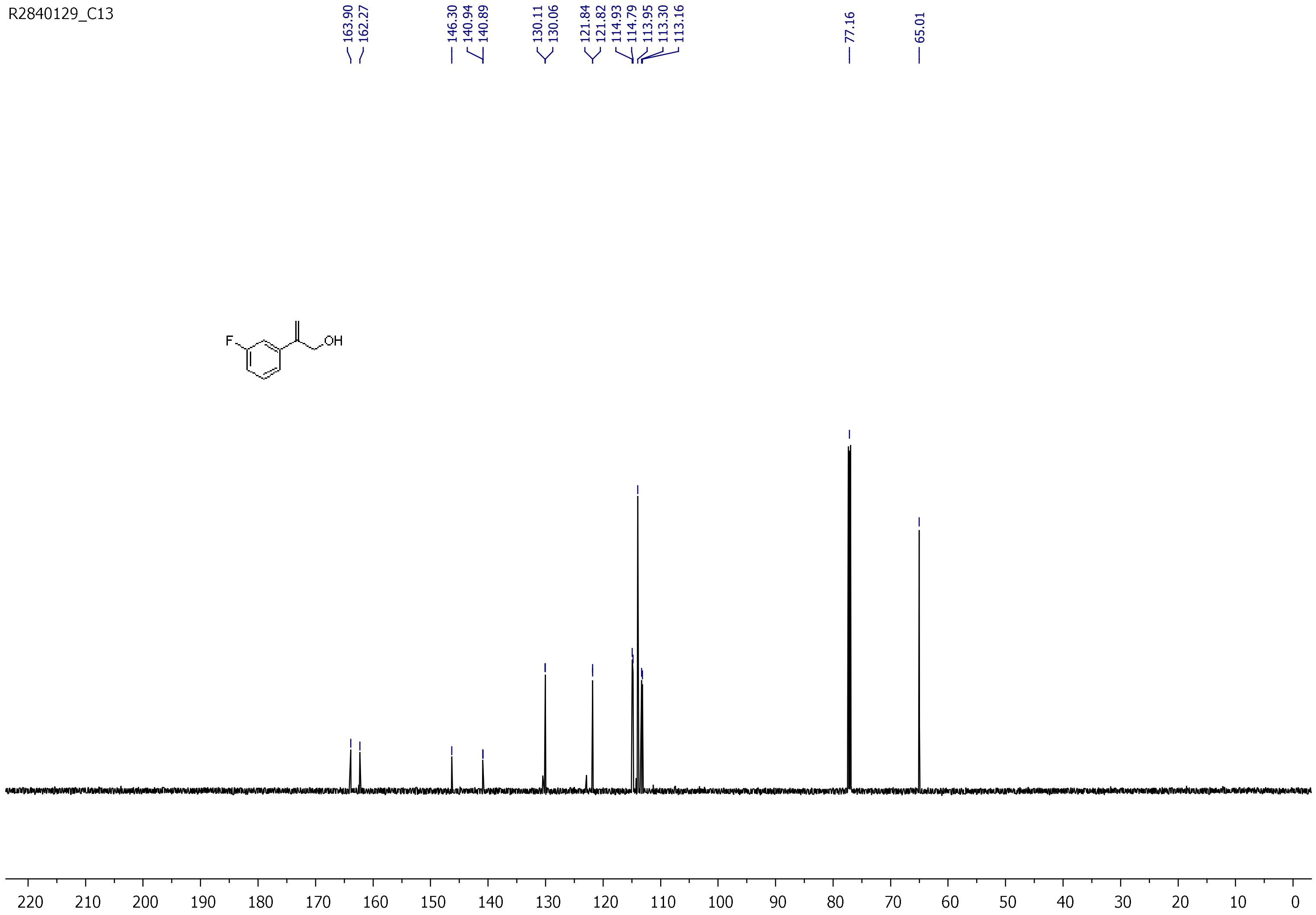

$^{19}F\{^{1}H\}$ NMR (376 MHz, $CDCl_3$)

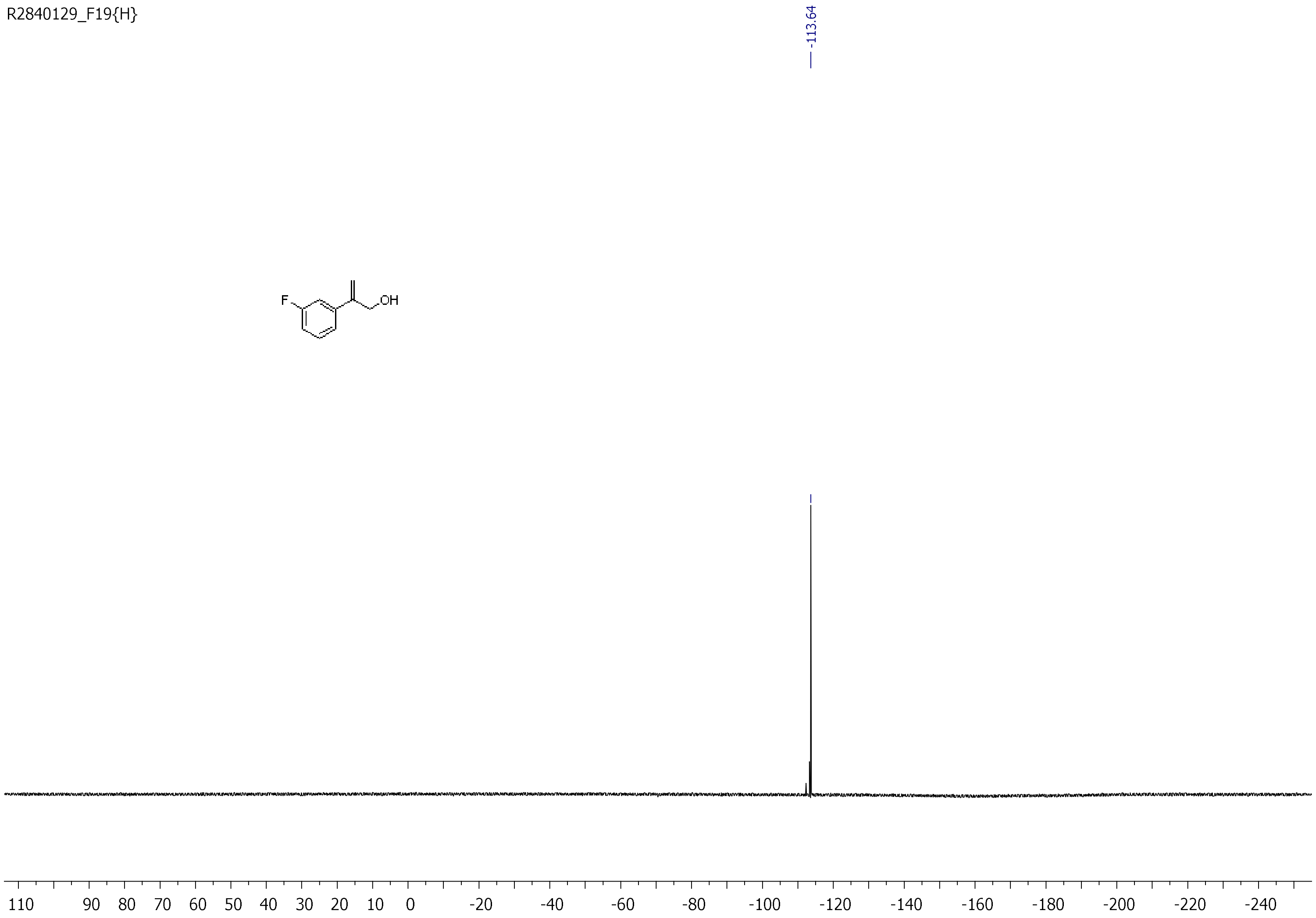

Compound (±)-9a, d.r. = 4:1

^{1}H NMR (500 MHz, $CDCl_3$)

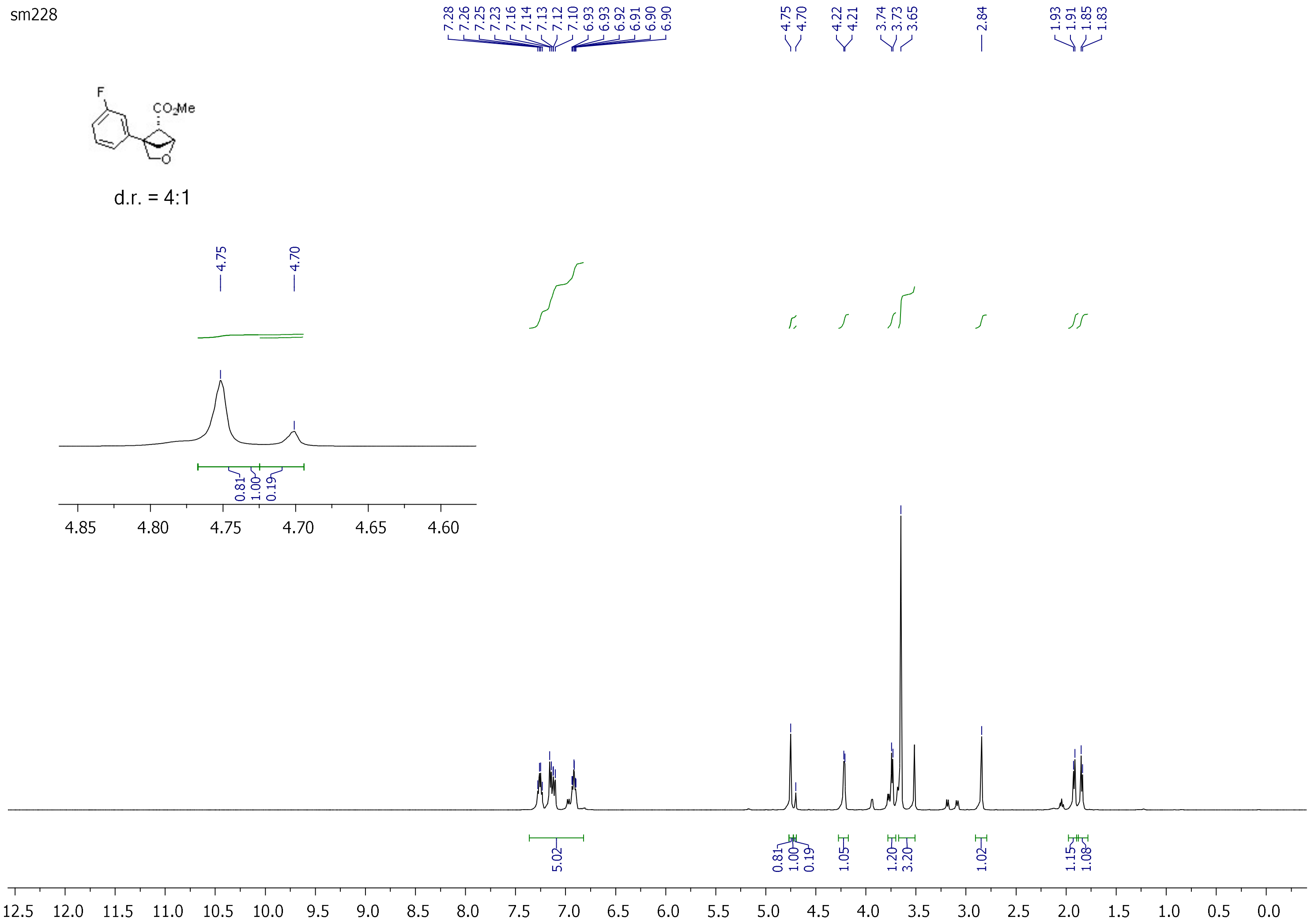

$^{13}C\{^1H\}$ NMR (126 MHz, $CDCl_3$)

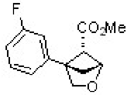

d.r. = 4:1

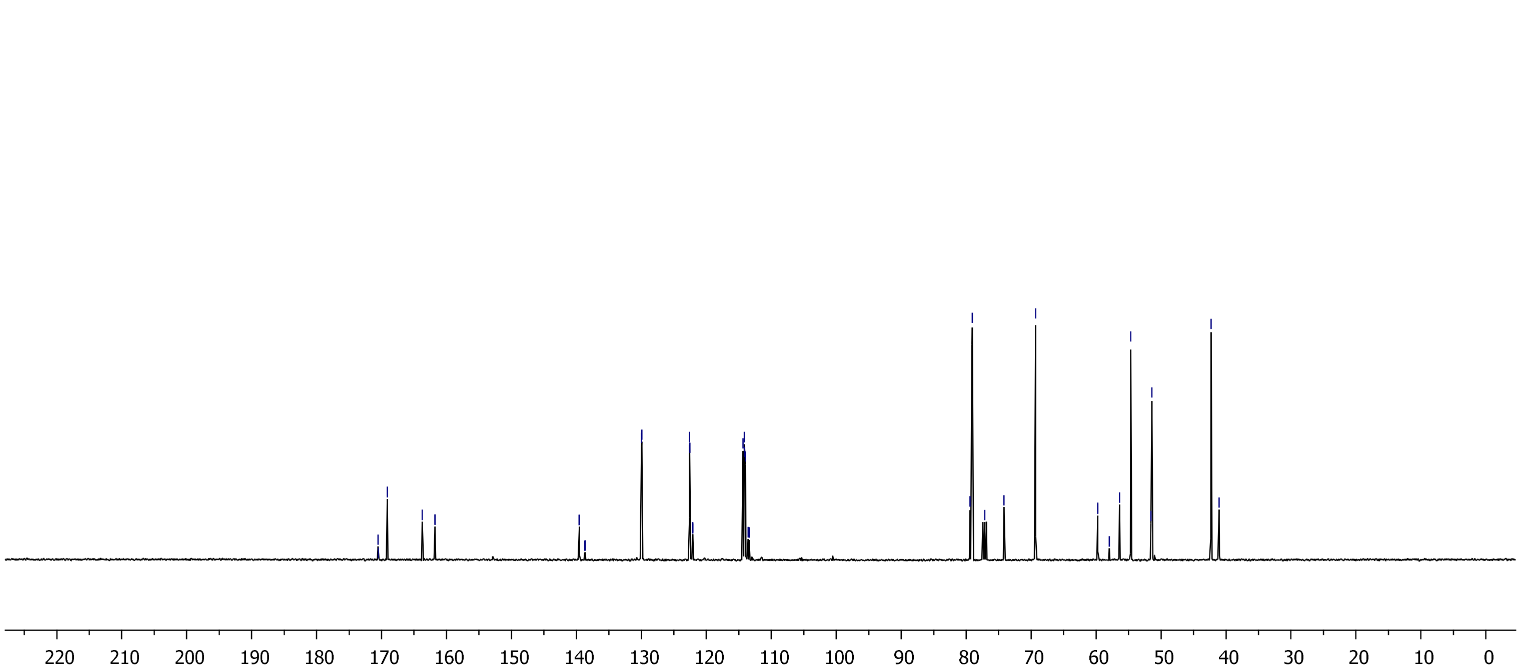

$^{19}F\{^{1}H\}$ NMR (376 MHz, $CDCl_3$)

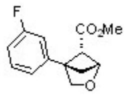

d.r. = 4:1

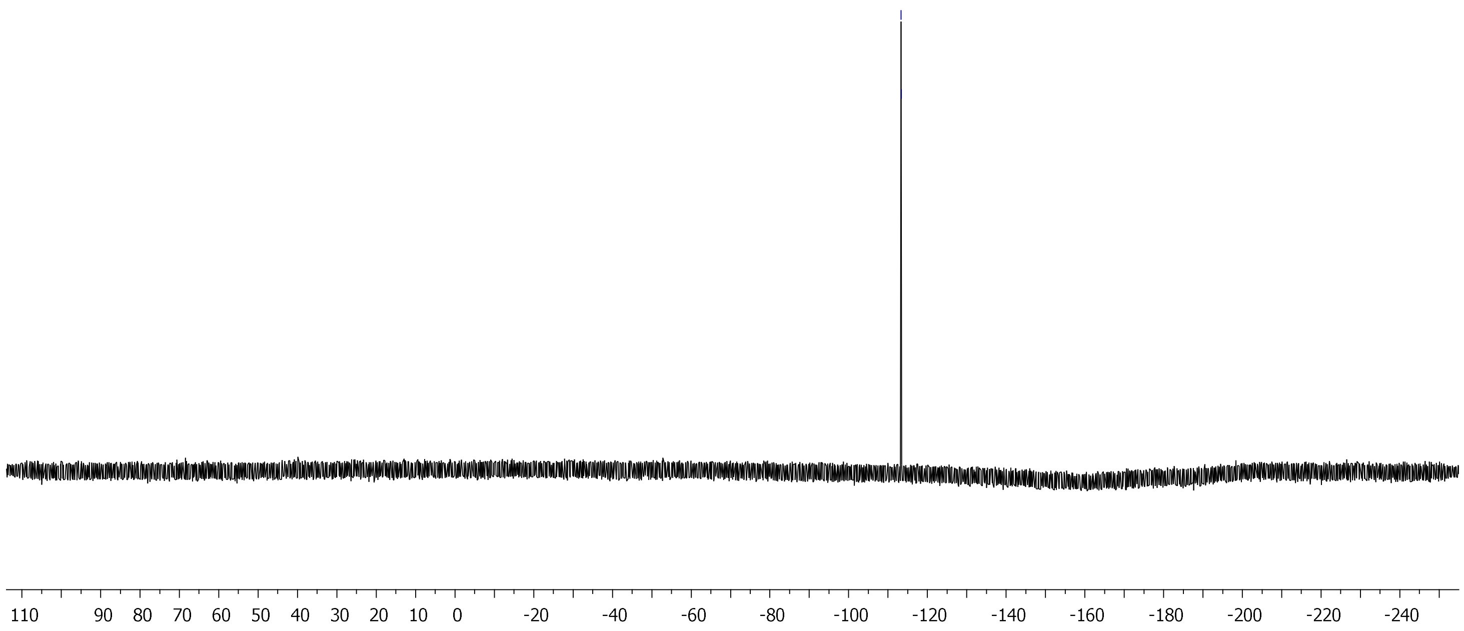

Compound (±)-9b

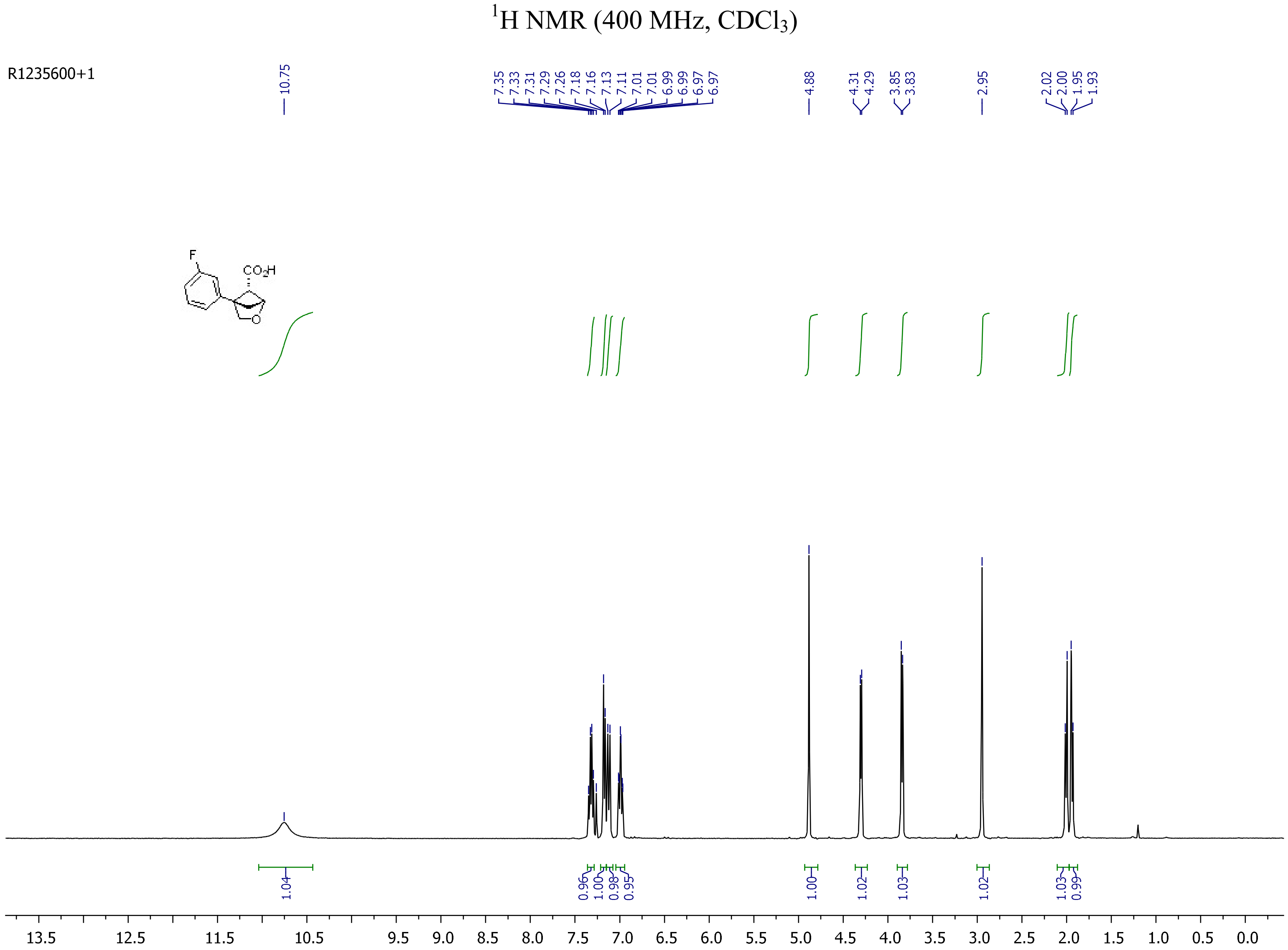

^{1}H NMR (400 MHz, CDCl$_3$)
R1235600+1
10.75
7.35 7.33 7.31 7.29 7.26 7.18 7.16 7.13 7.11 7.01 7.01 6.99 6.99 6.97 6.97
4.88
4.31 4.29
3.85 3.83
2.95
2.02 2.00 1.95 1.93
F
CO_2H
O
1.04
0.96 1.00 0.98 0.95
1.00
1.02
1.03
1.02
1.03 0.99
13.5 12.5 11.5 10.5 9.5 9.0 8.5 8.0 7.5 7.0 6.5 6.0 5.5 5.0 4.5 4.0 3.5 3.0 2.5 2.0 1.5 1.0 0.5 0.0

$^{13}C\{^{1}H\}$ NMR (126 MHz, $CDCl_3$)

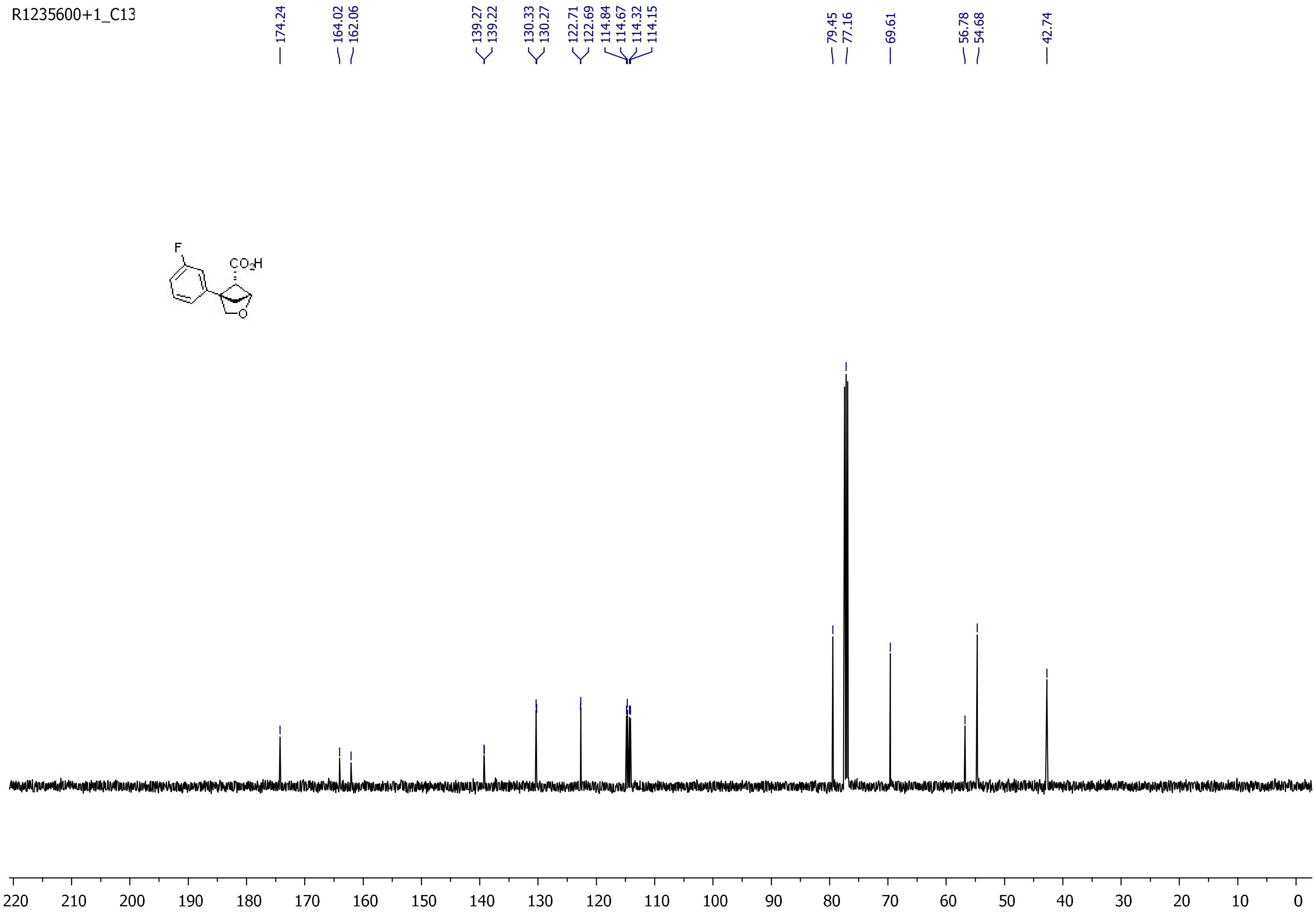

$^{19}F\{^{1}H\}$ NMR (376 MHz, $CDCl_3$)

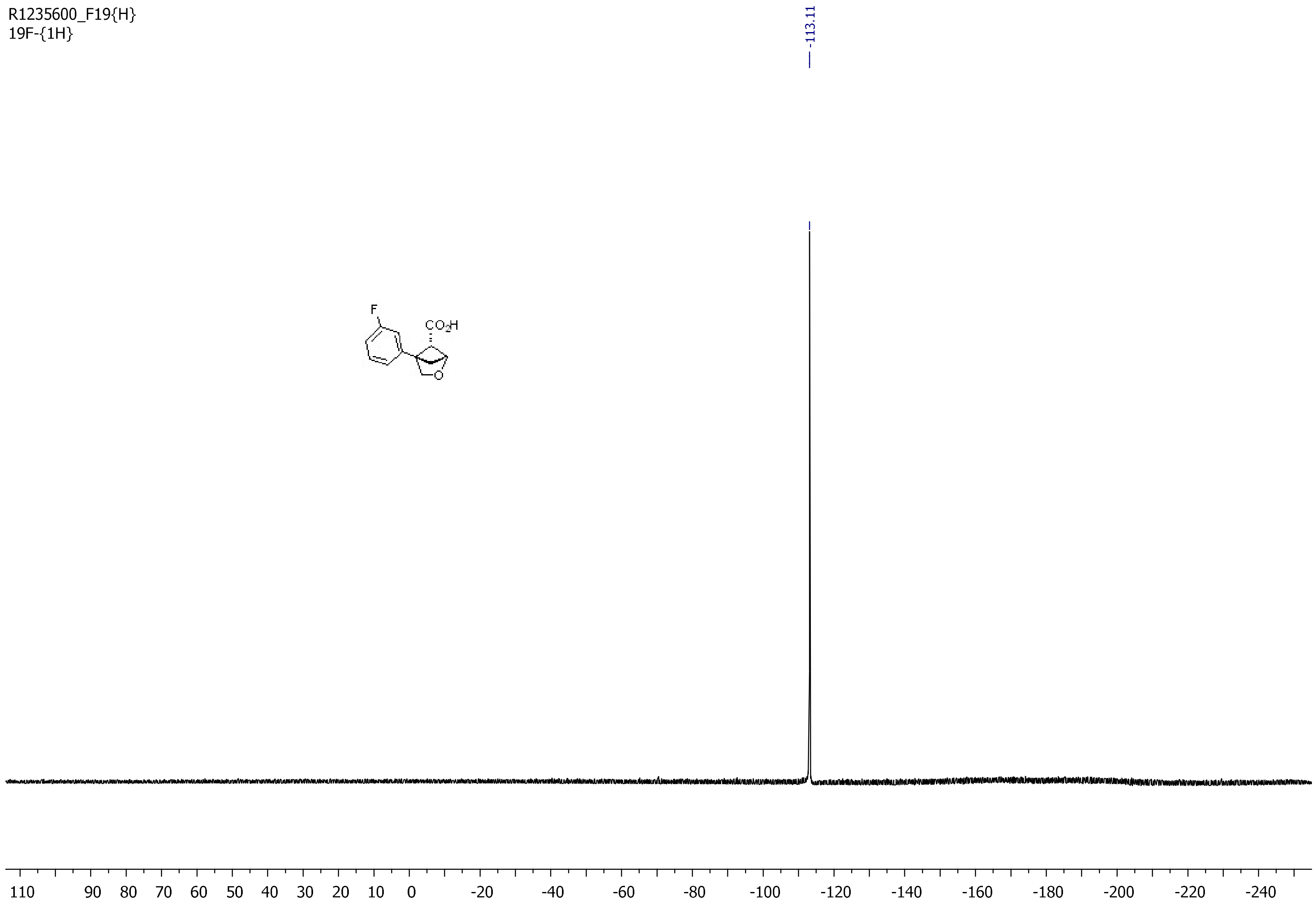

2-(4-Fluorophenyl)prop-2-en-1-ol (ca. 90% purity)

^{1}H NMR (400 MHz, $CDCl_3$)

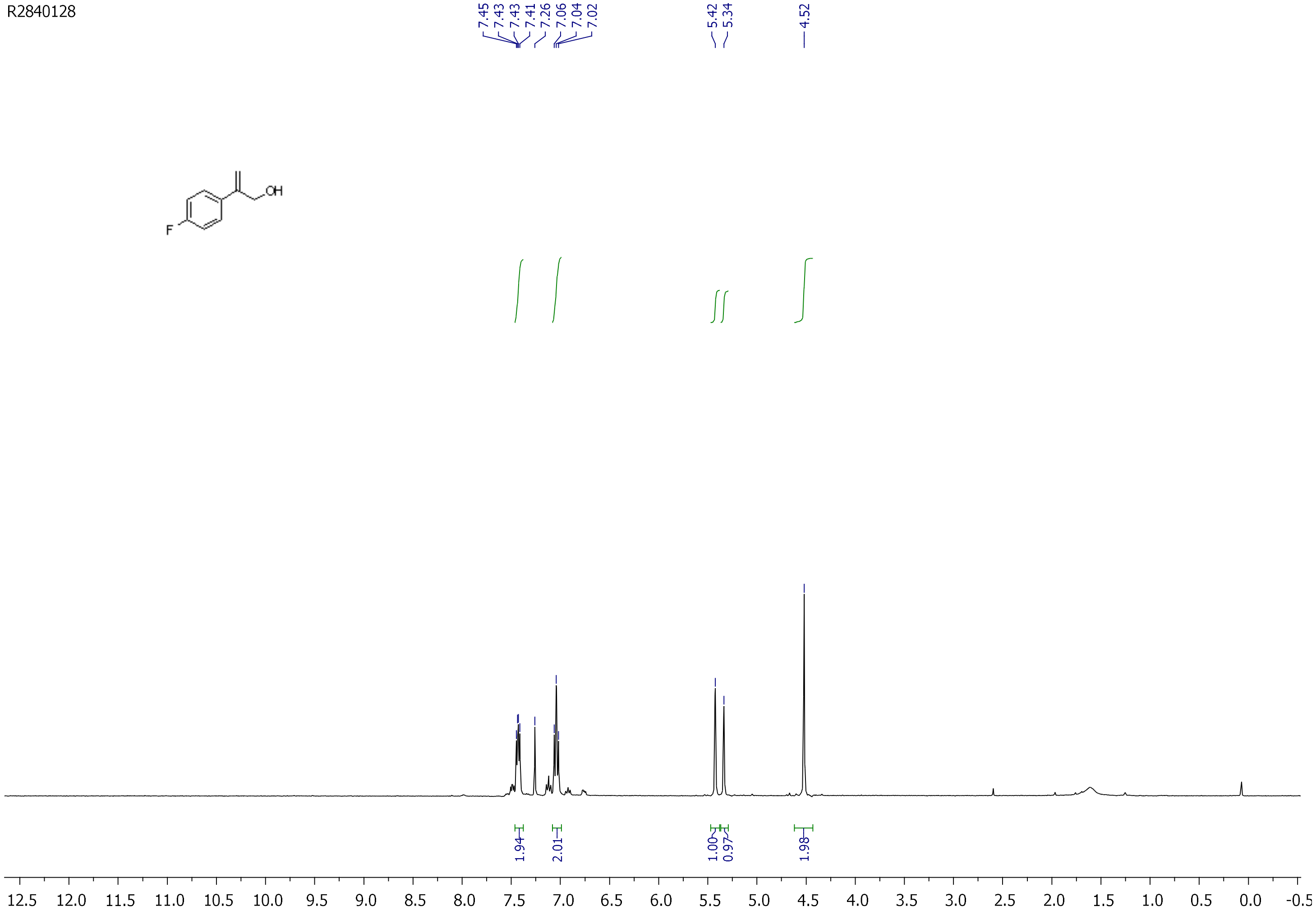

$^{13}C\{^{1}H\}$ NMR (151 MHz, $CDCl_3$)

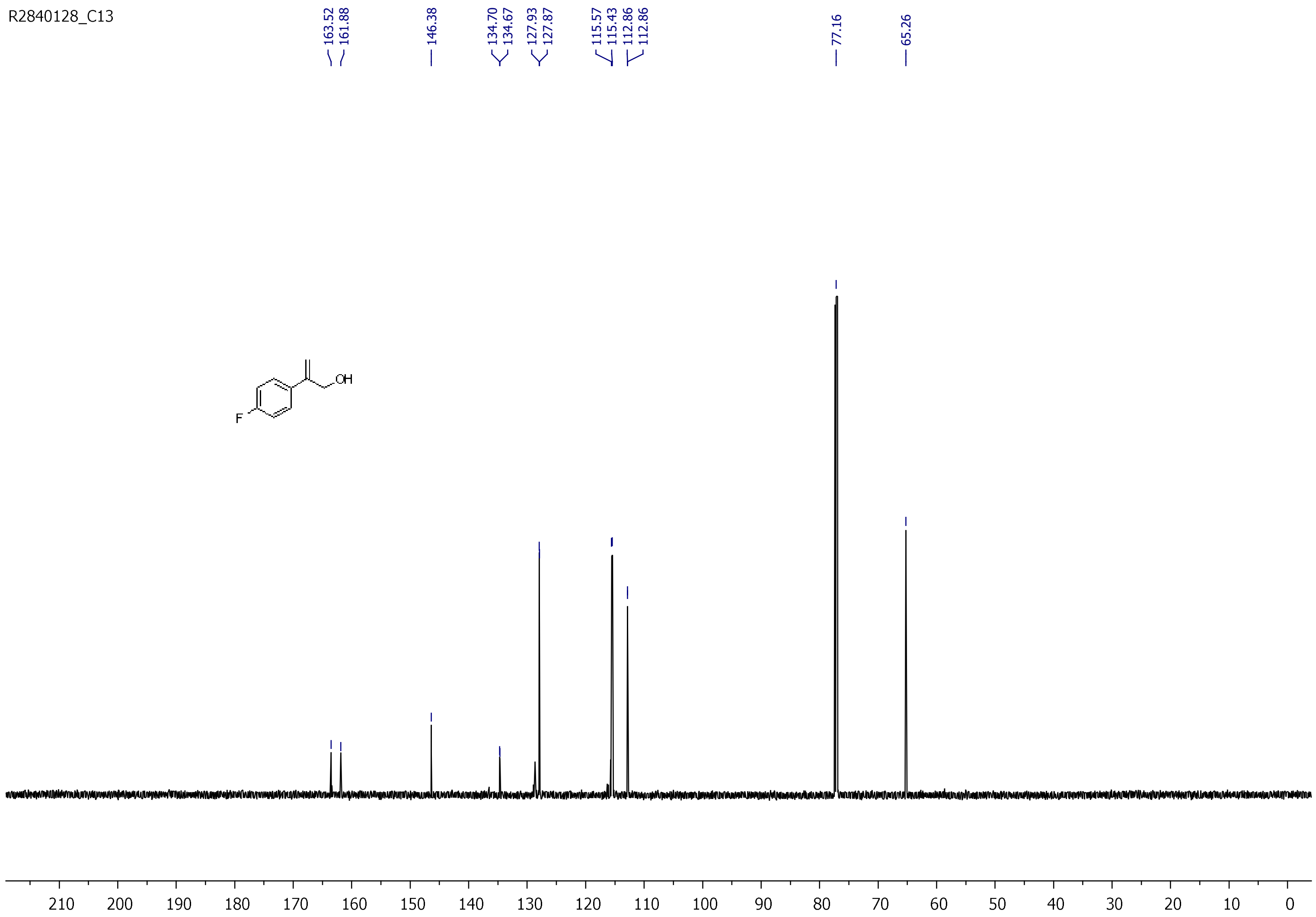

$^{19}F\{^{1}H\}$ NMR (376 MHz, $CDCl_3$)

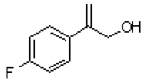

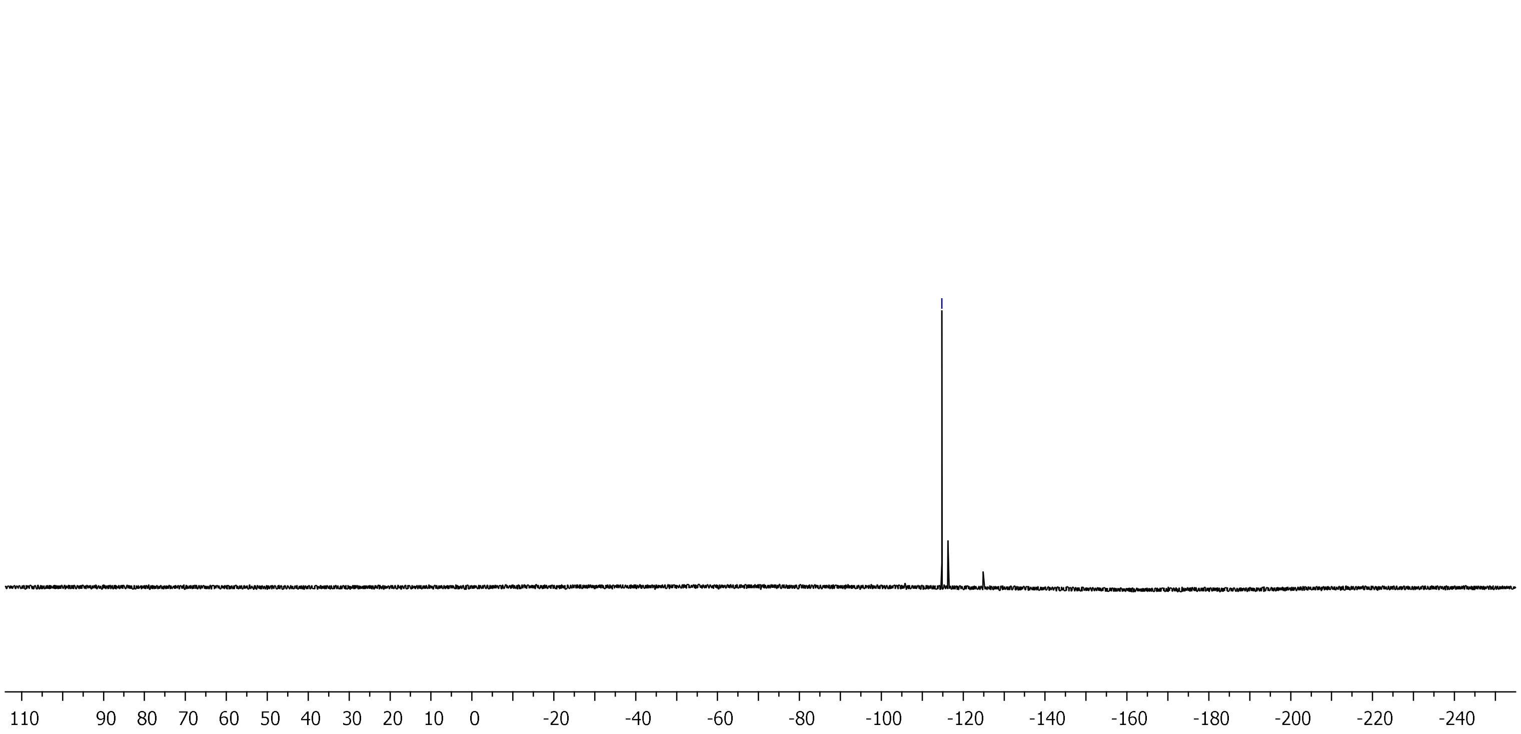

Compound (±)-10a

^{1}H NMR (400 MHz, $CDCl_3$)

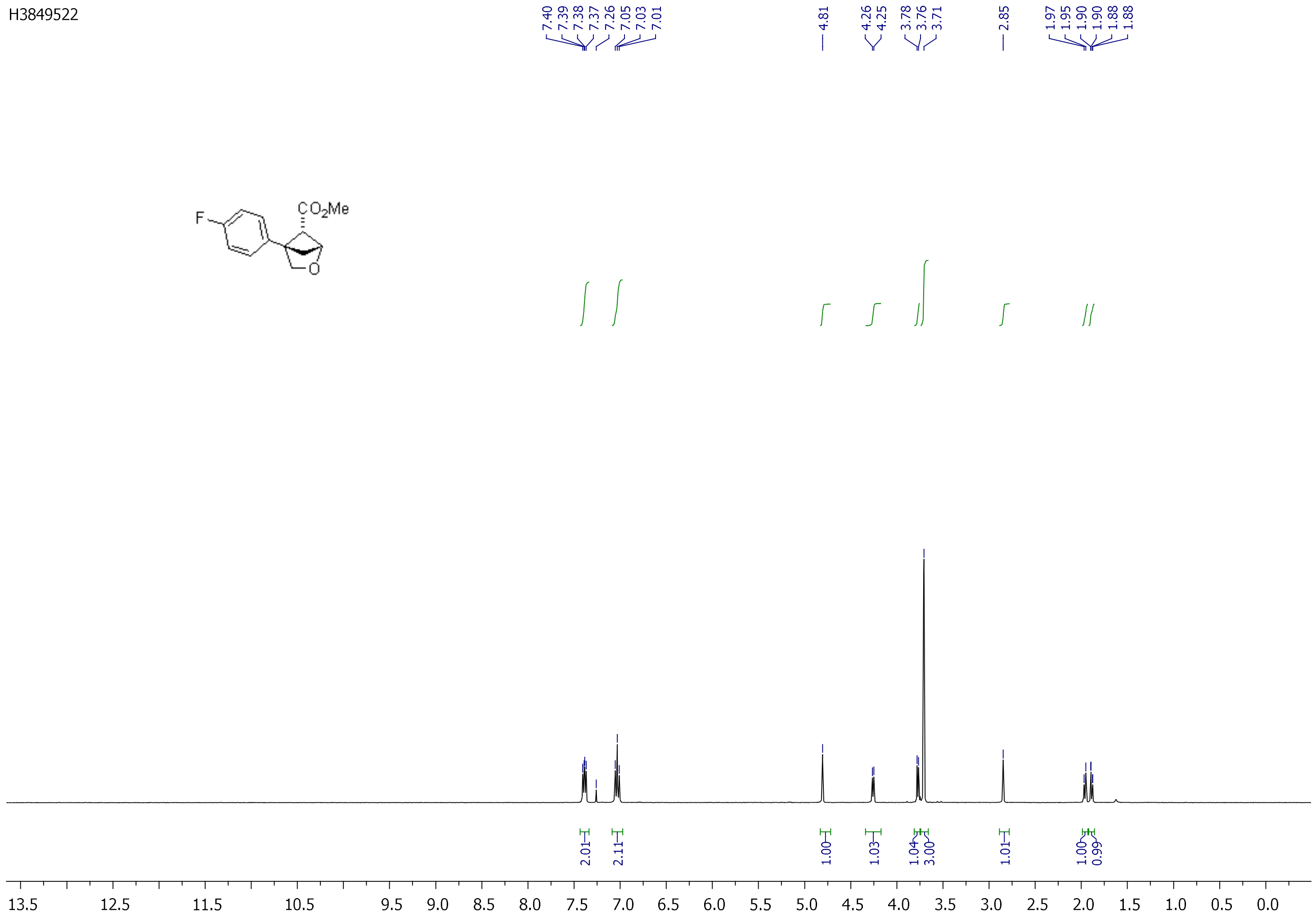

$^{13}C\{^{1}H\}$ NMR (126 MHz, $CDCl_3$)

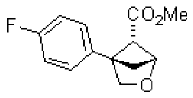

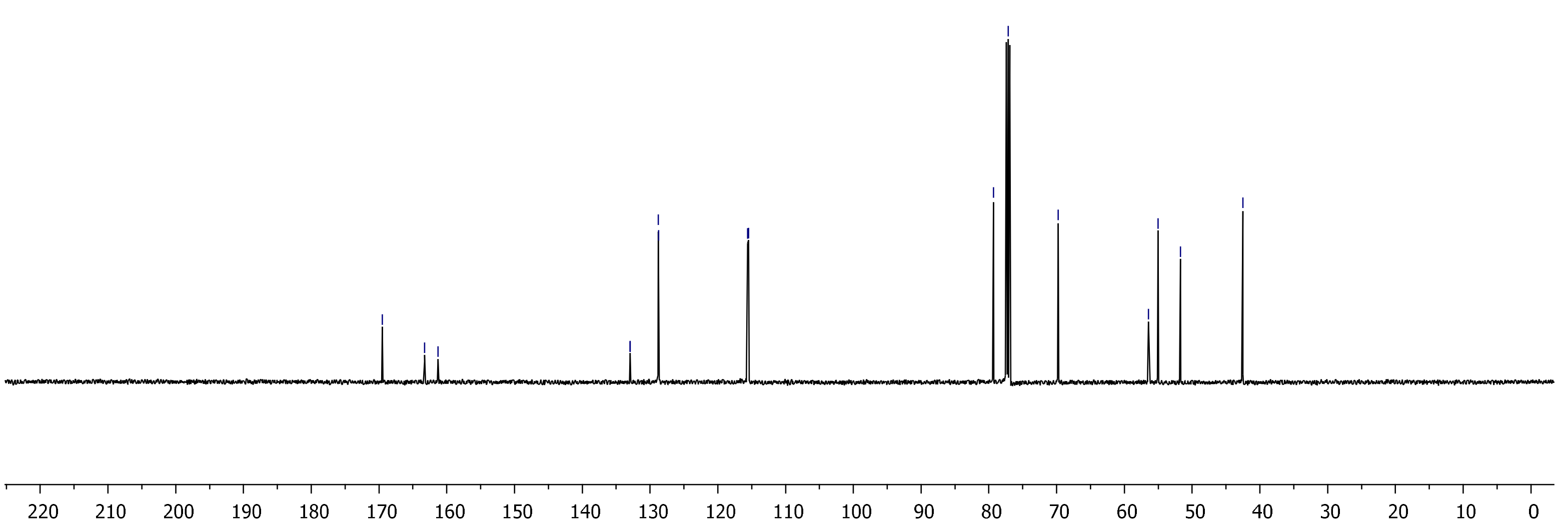

$^{19}F\{^1H\}$ NMR (376 MHz, $CDCl_3$)

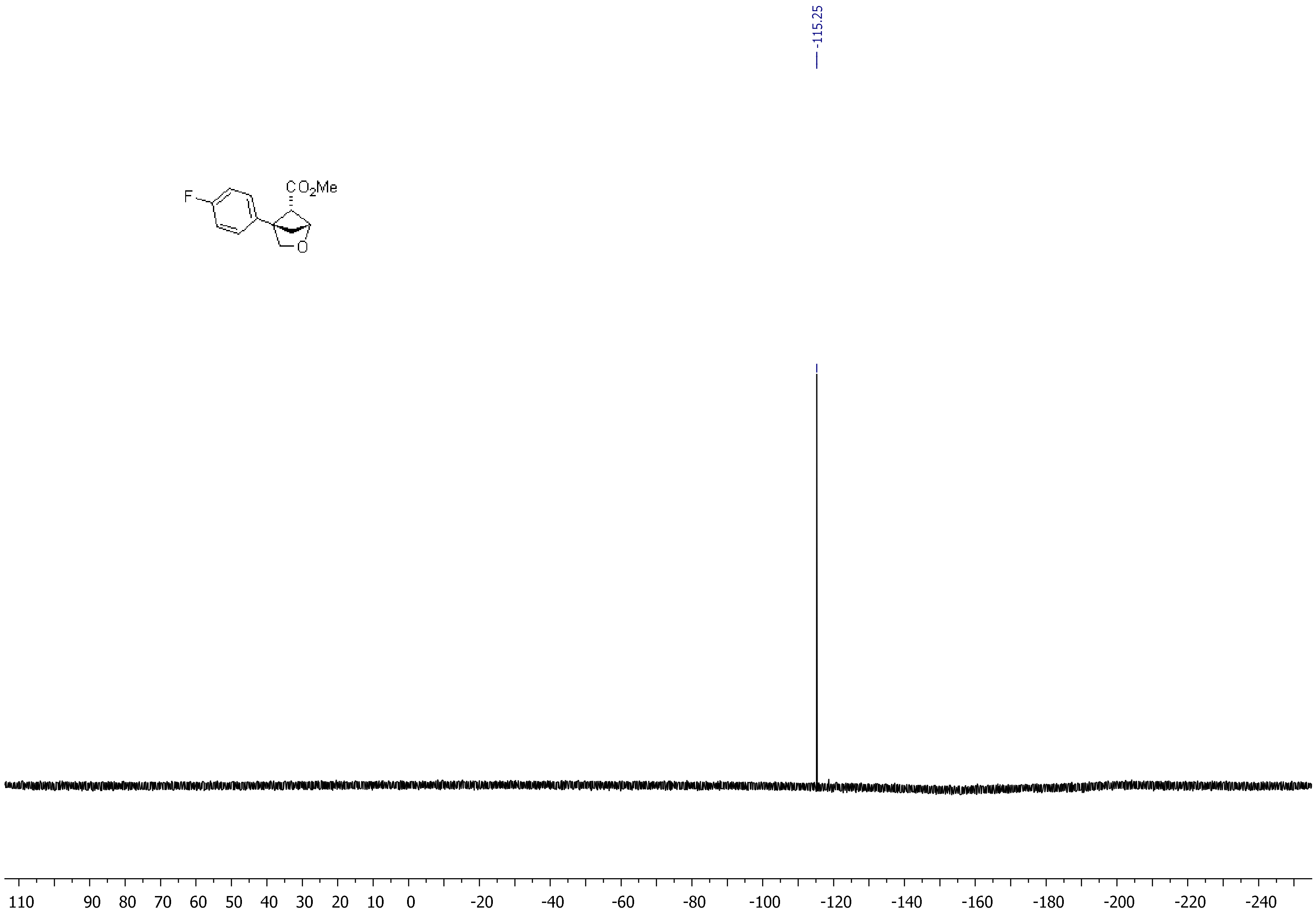

Compound (±)-10b, d.r. = 5:1

^{1}H NMR (400 MHz, $CDCl_3$)

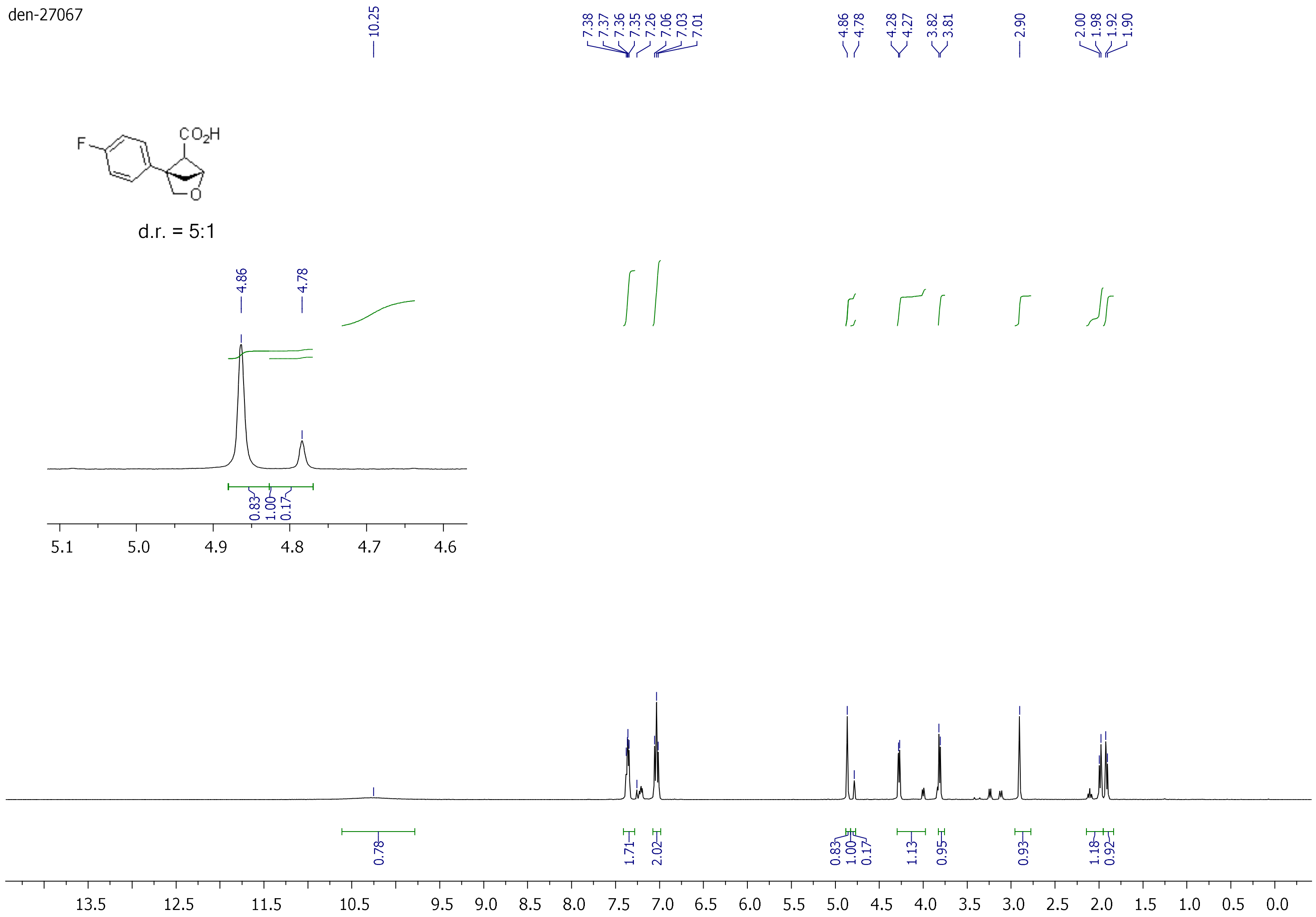

$^{13}C\{^{1}H\}$ NMR (101 MHz, $CDCl_3$)

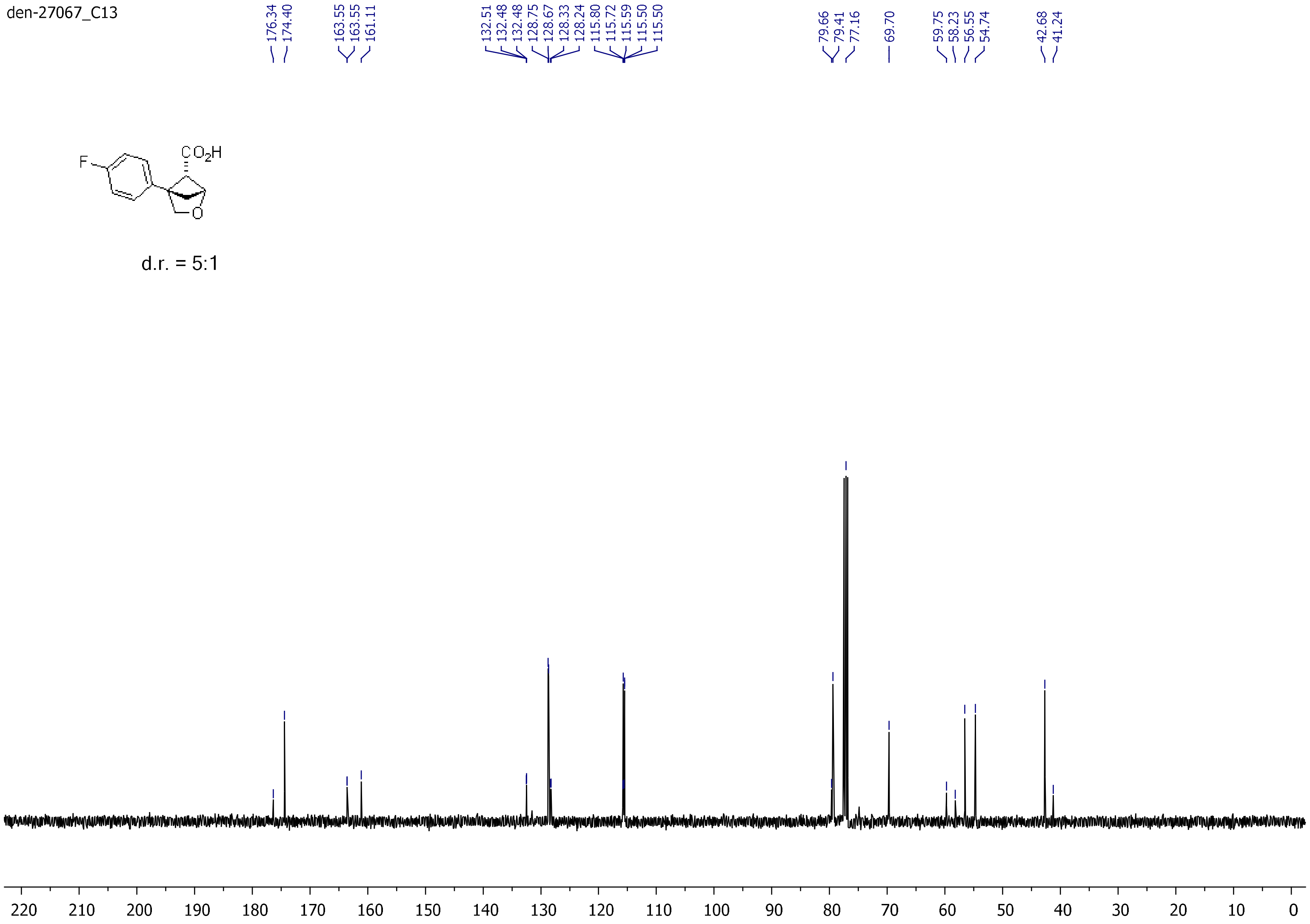

$^{19}F\{^{1}H\}$ NMR (376 MHz, $CDCl_3$)

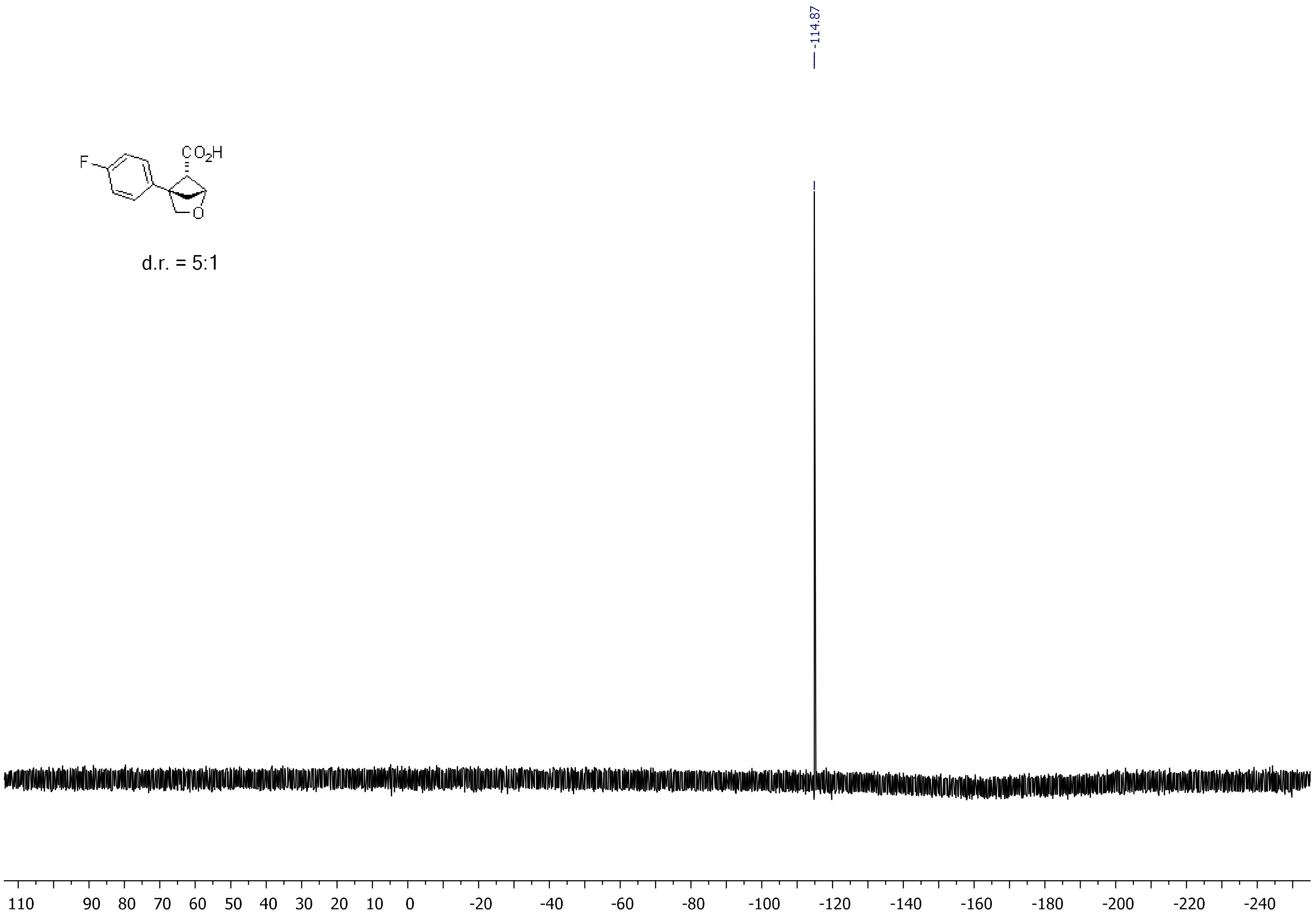

2-(3,4,5-Trifluorophenyl)prop-2-en-1-ol

^{1}H NMR (400 MHz, DMSO-d$_6$)

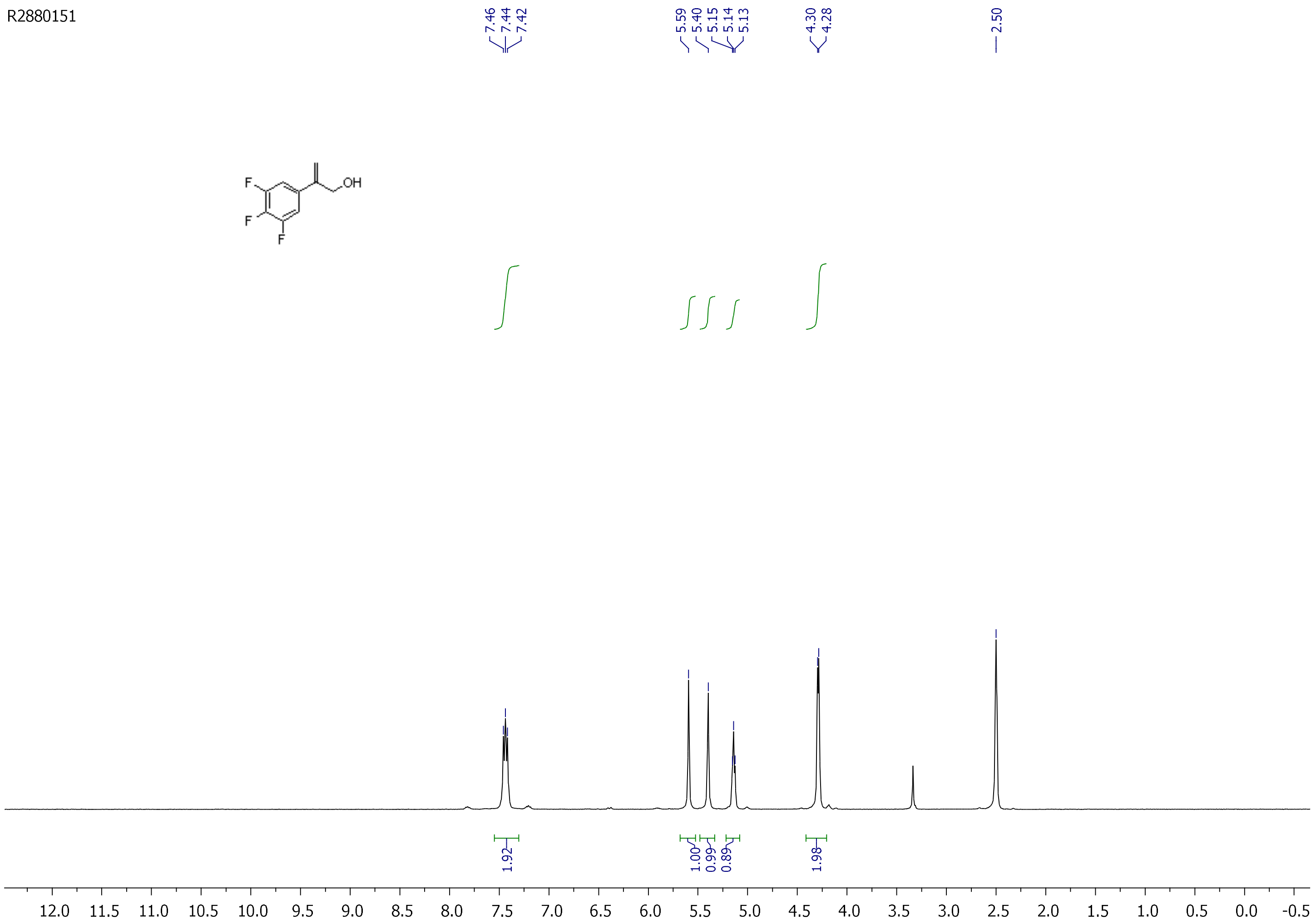

$^{13}C\{^{1}H\}$ NMR (126 MHz, DMSO-d_6)

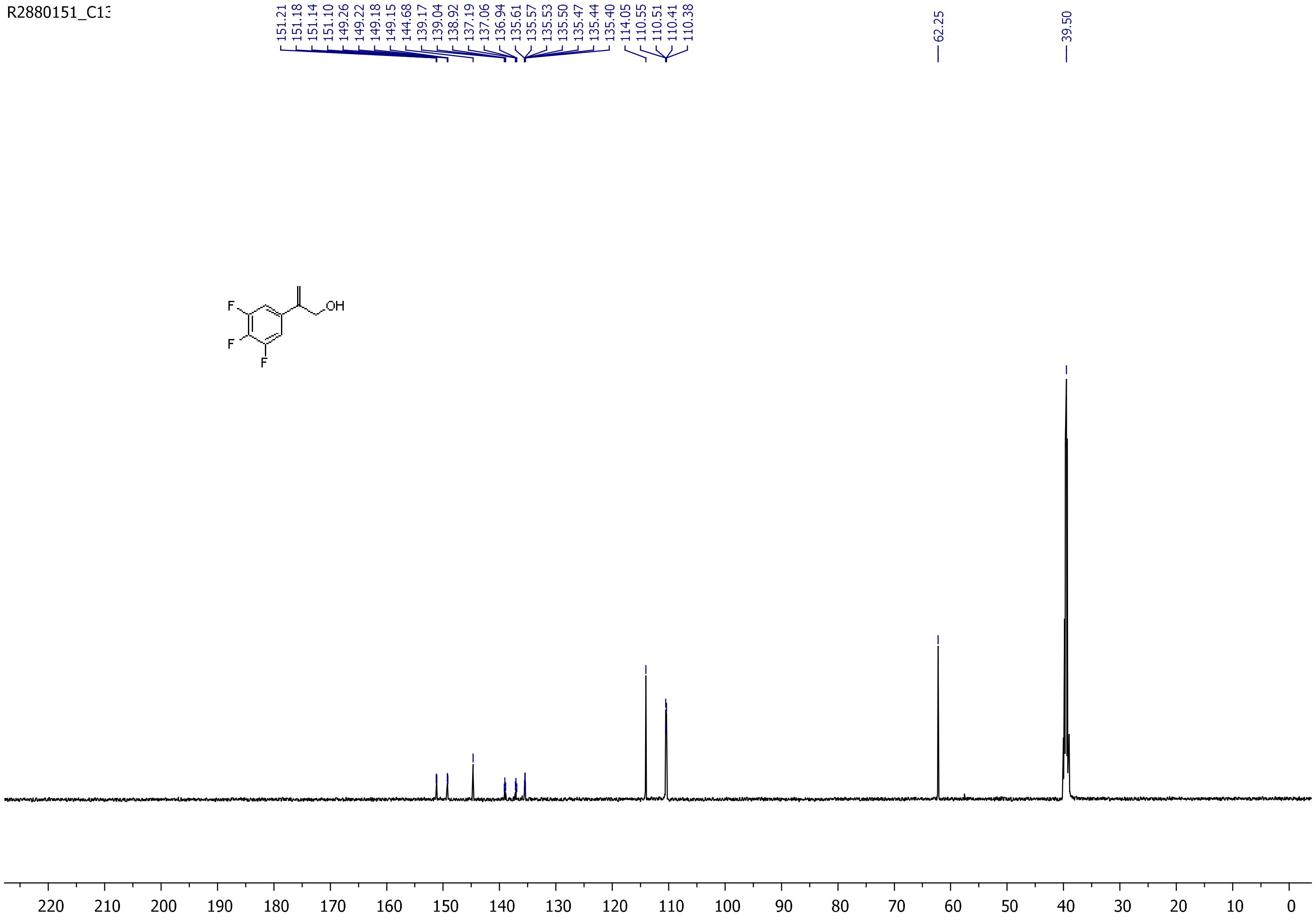

$^{19}F\{^{1}H\}$ NMR (376 MHz, DMSO-d$_6$)

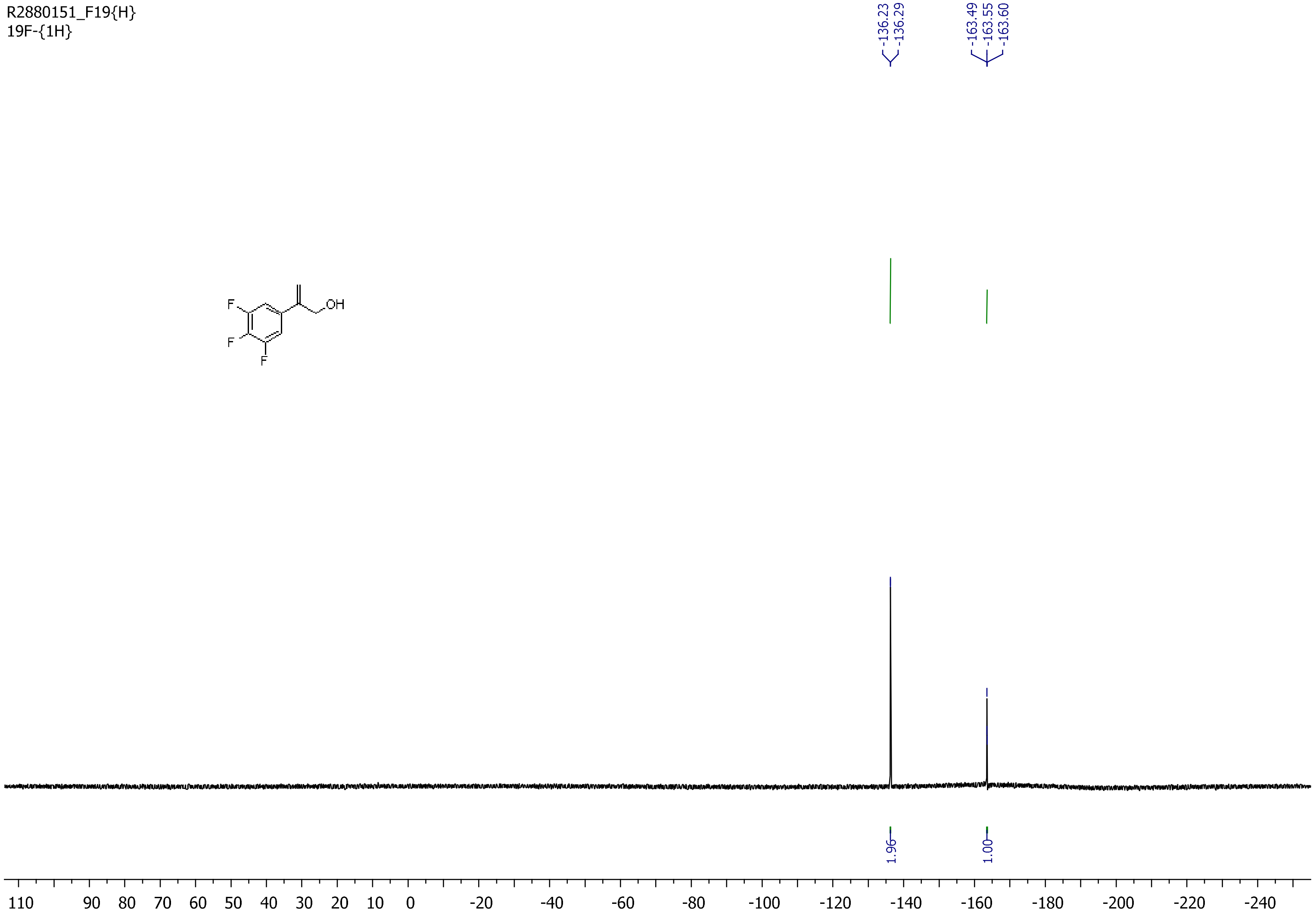

Compound (±)-11a, d.r. = 4:1

^{1}H NMR (500 MHz, $CDCl_3$)

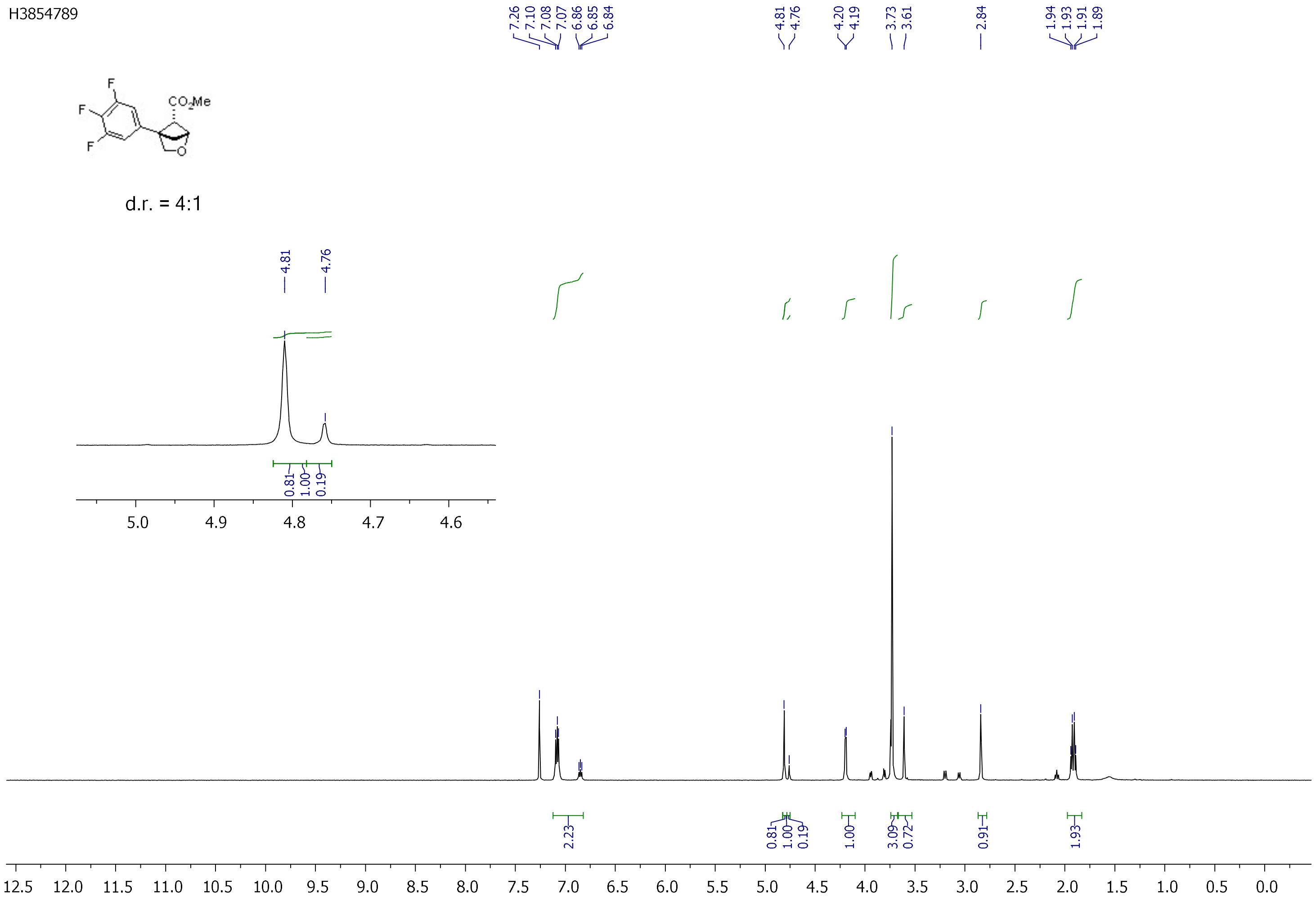

$^{13}C\{^1H\}$ NMR (126 MHz, $CDCl_3$)

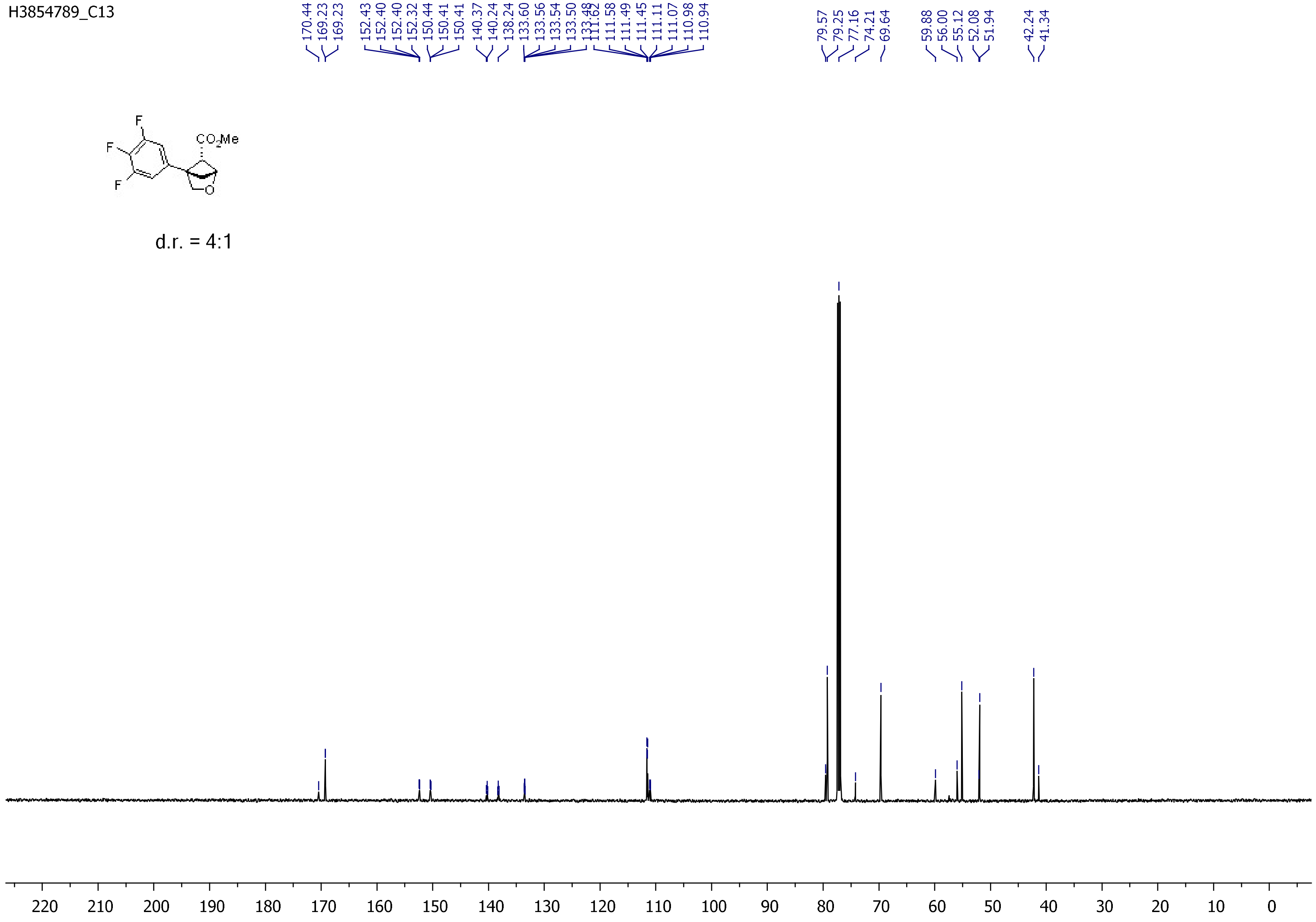

$^{19}F\{^{1}H\}$ NMR (376 MHz, $CDCl_3$)

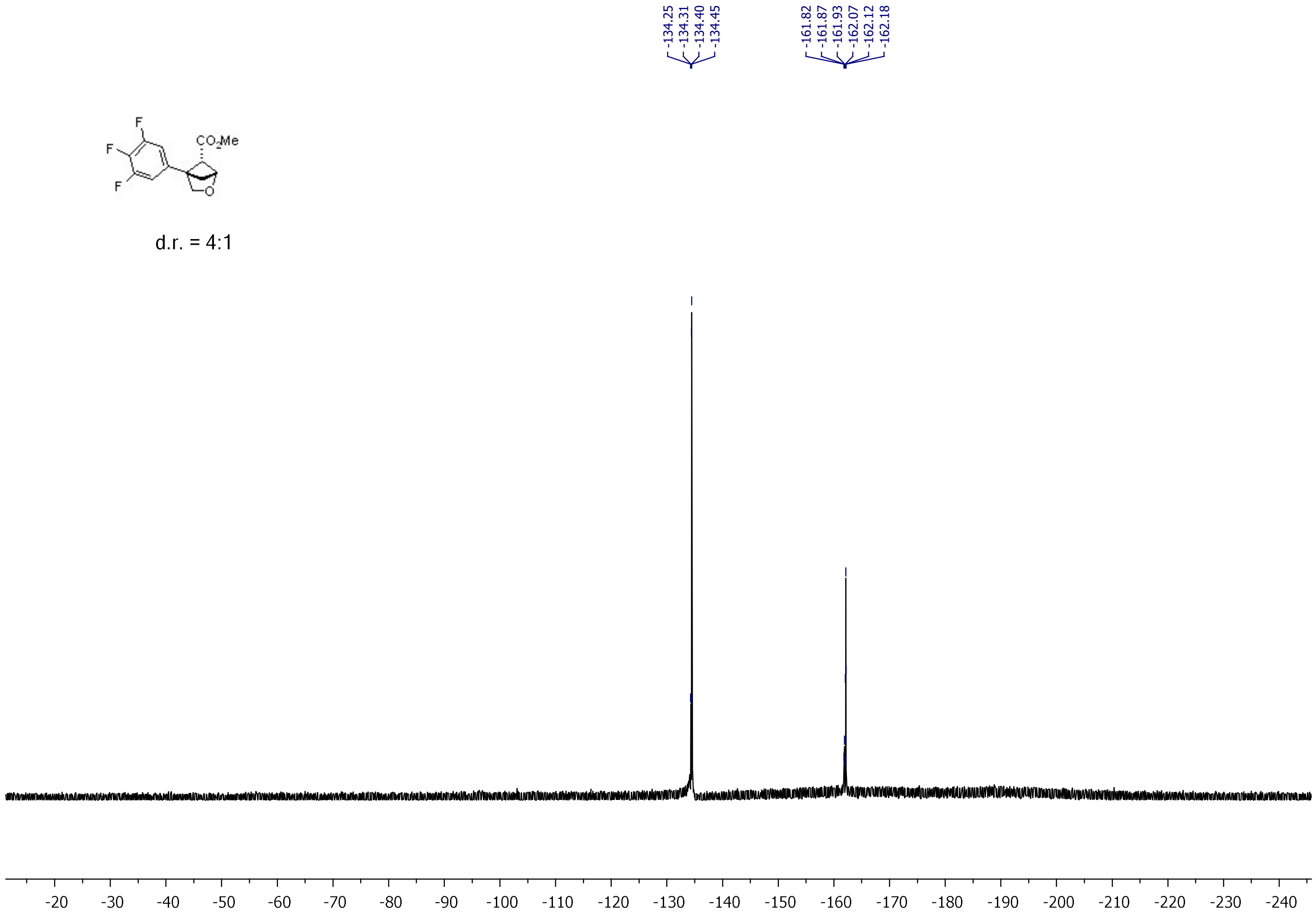

Compound (±)-11b, d.r. = 9:1

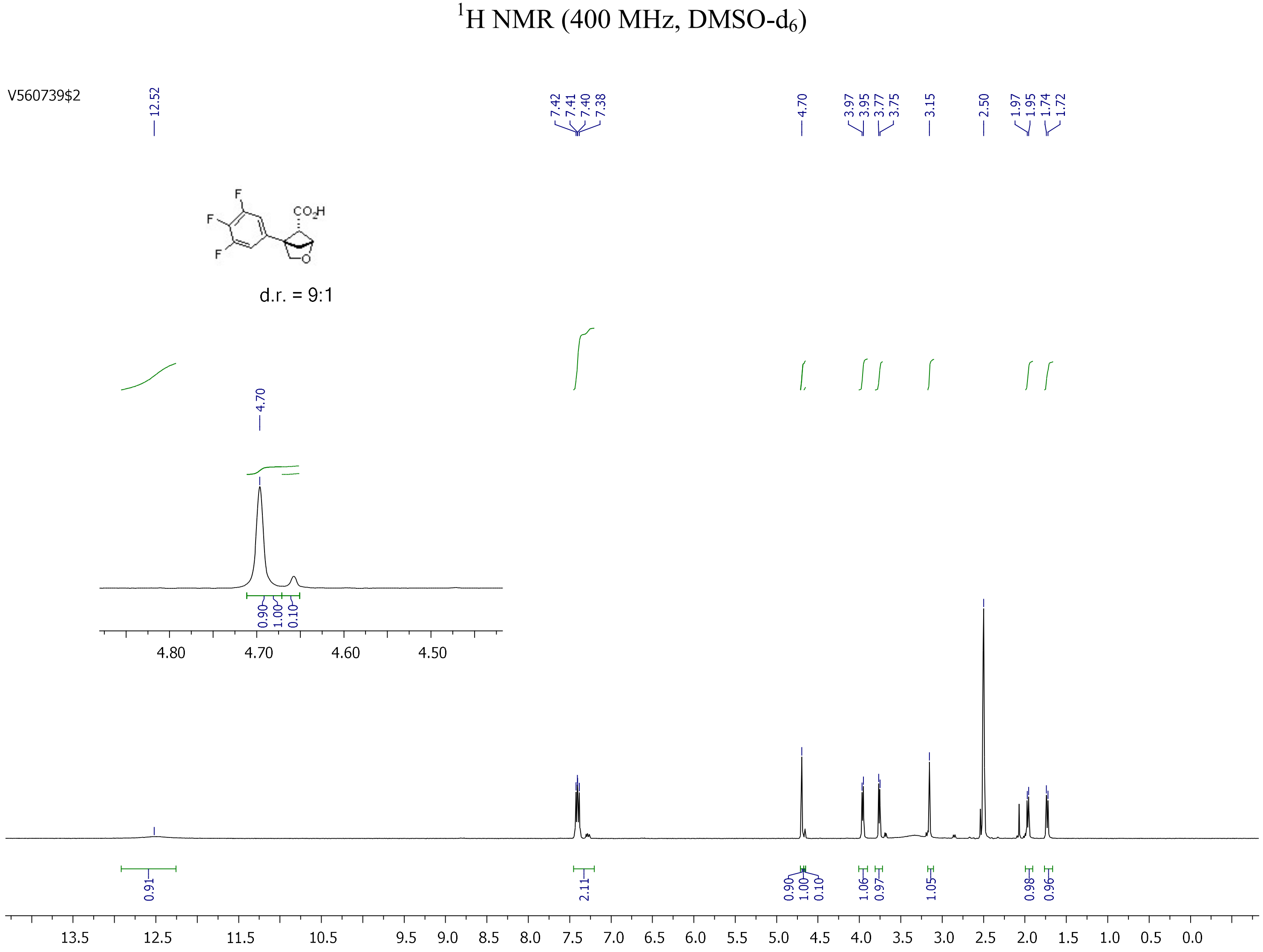

$^{13}C\{^1H\}$ NMR (126 MHz, DMSO-d_6)

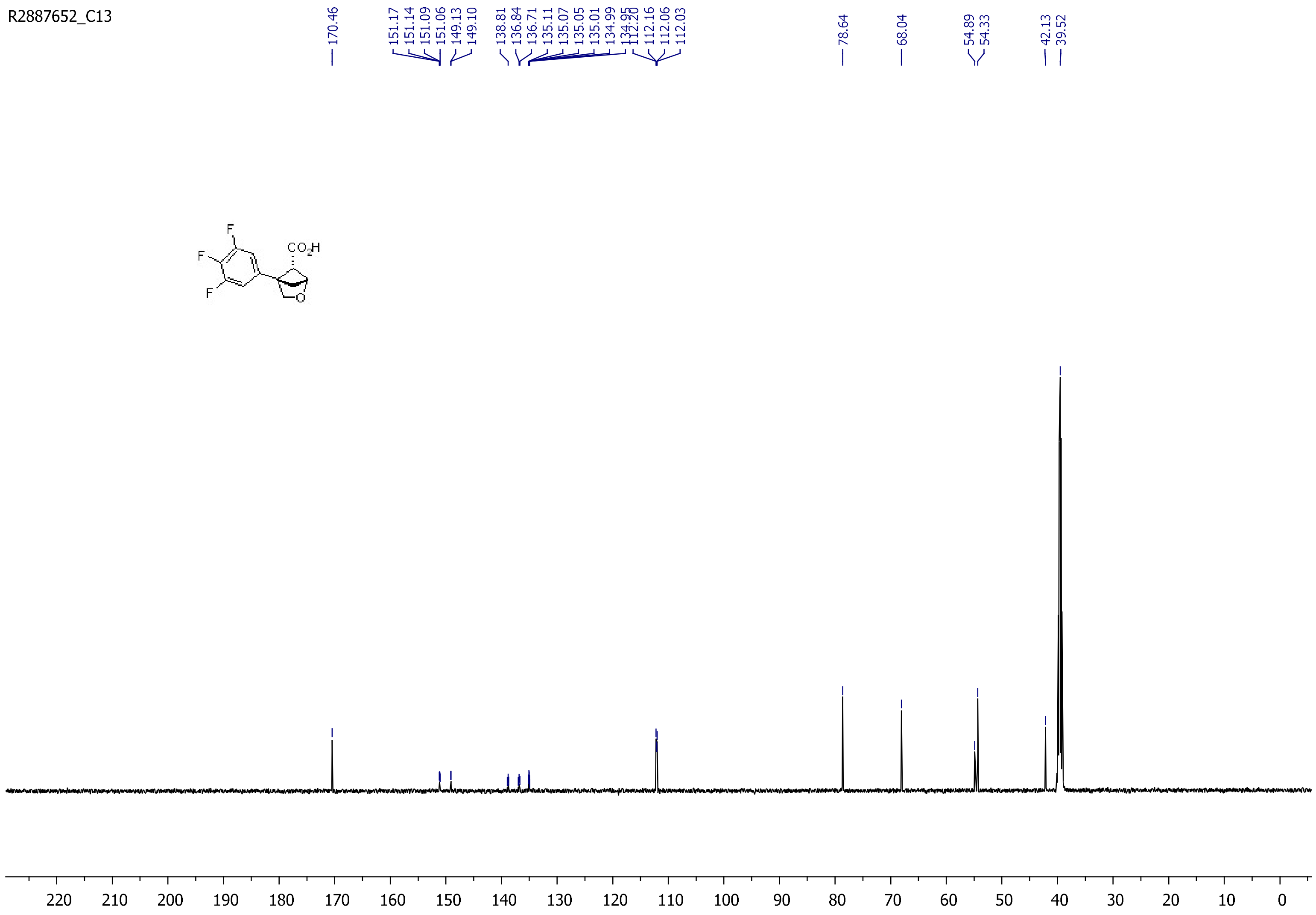

$^{19}F\{^1H\}$ NMR (376 MHz, DMSO-d_6)

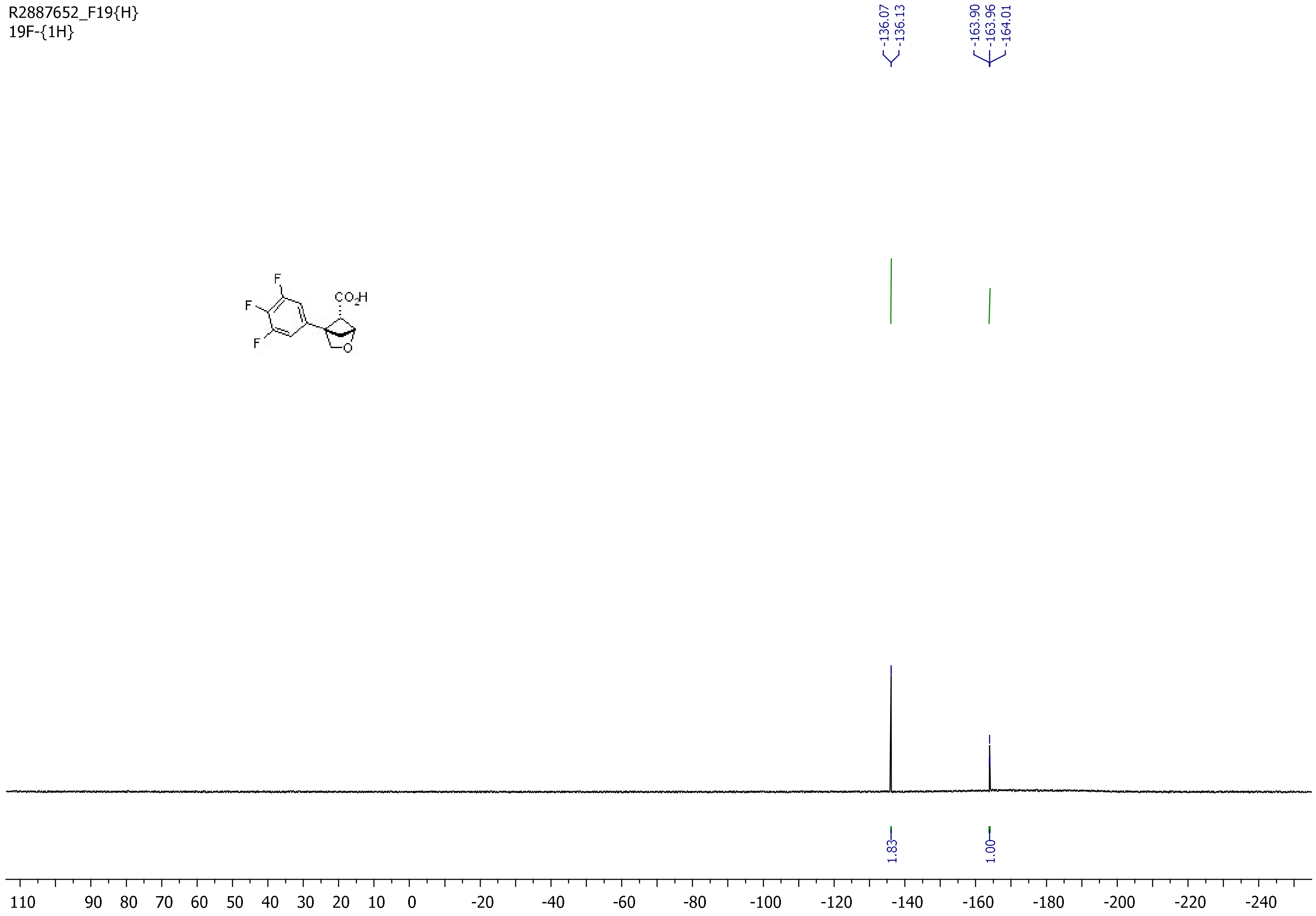

2-(3-Chlorophenyl)prop-2-en-1-ol (ca. 90% purity)

^{1}H NMR (400 MHz, $CDCl_3$)

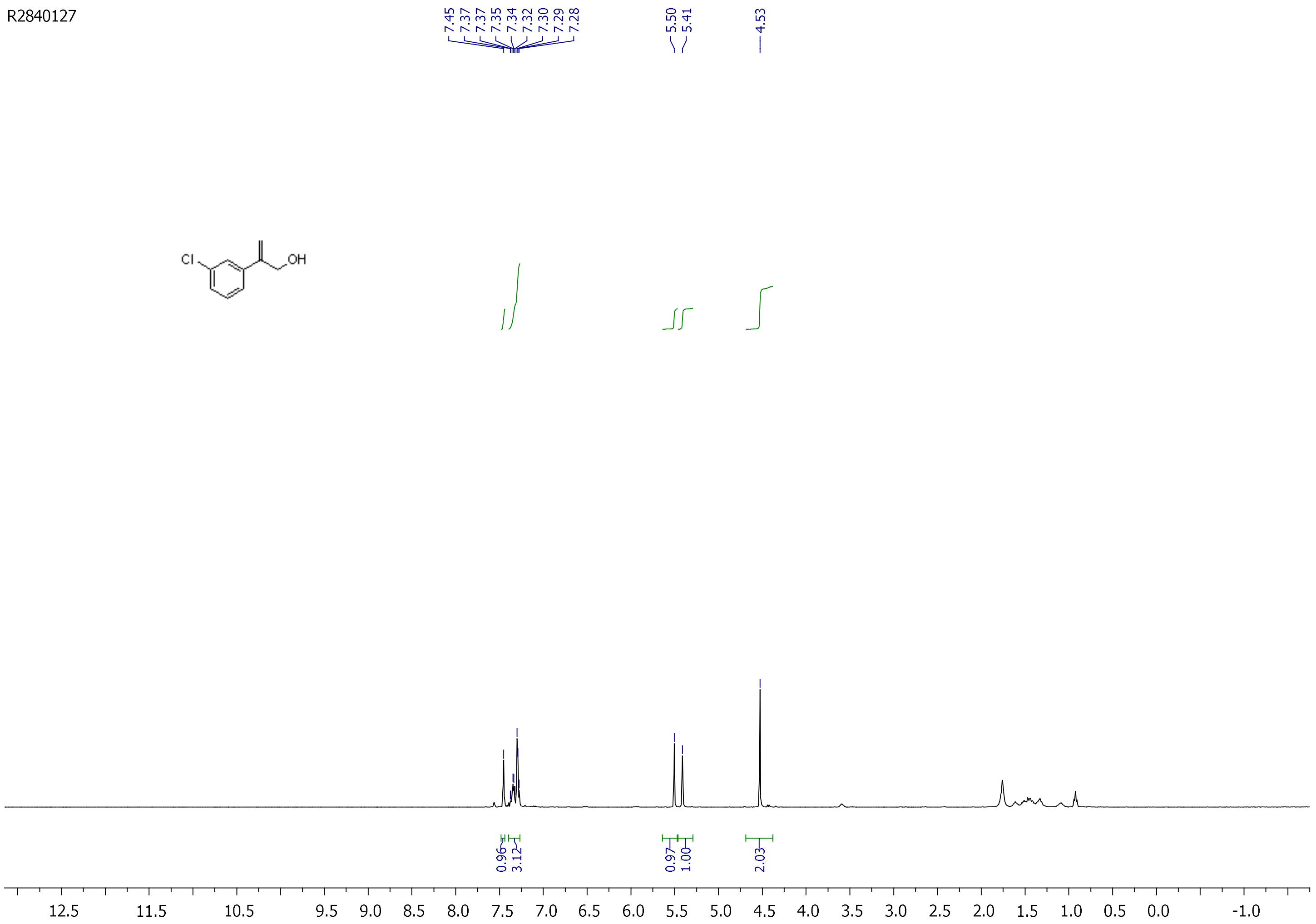

$^{13}C\{^1H\}$ NMR (126 MHz, $CDCl_3$)

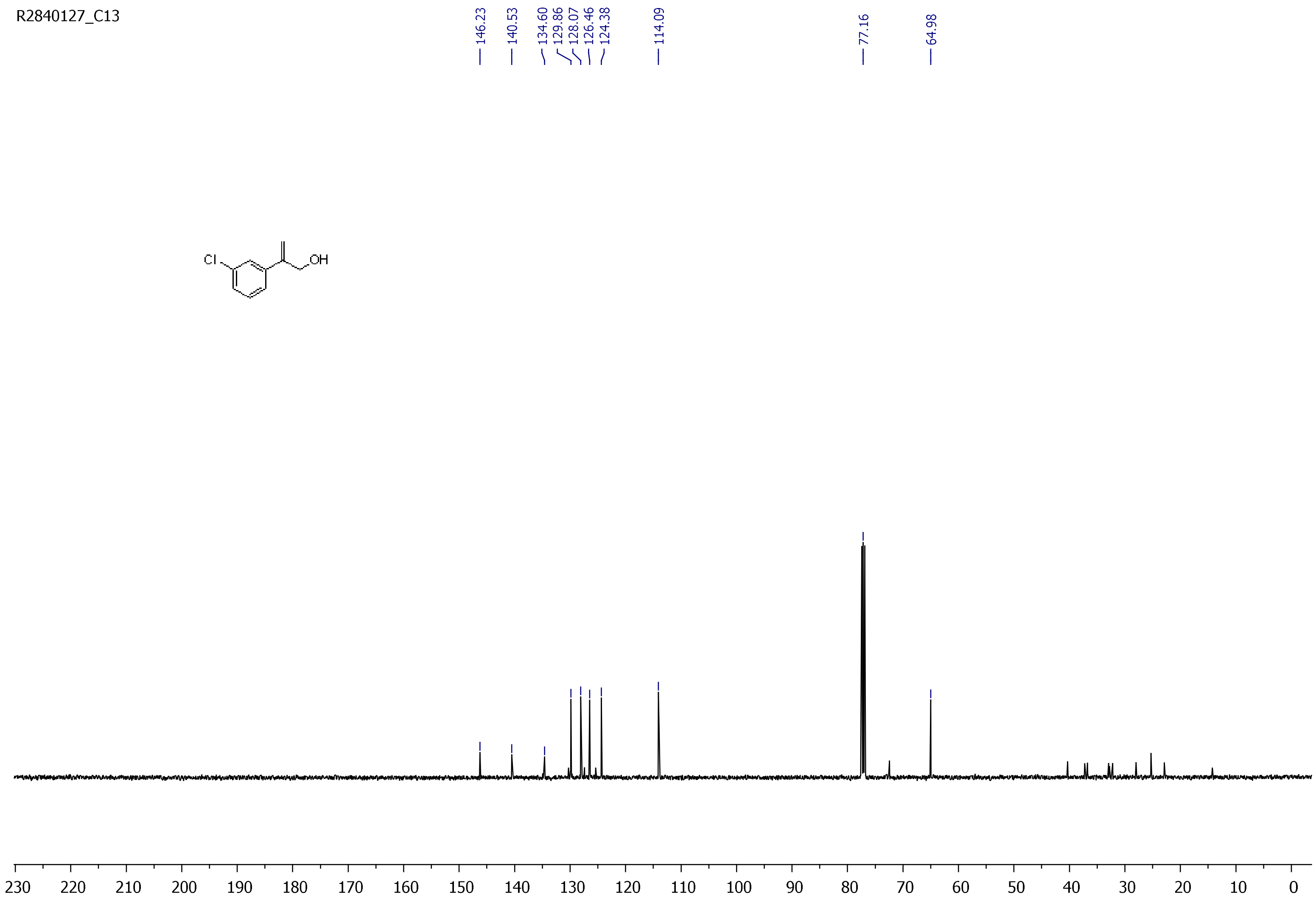

Compound (±)-12a, d.r. = 9:2

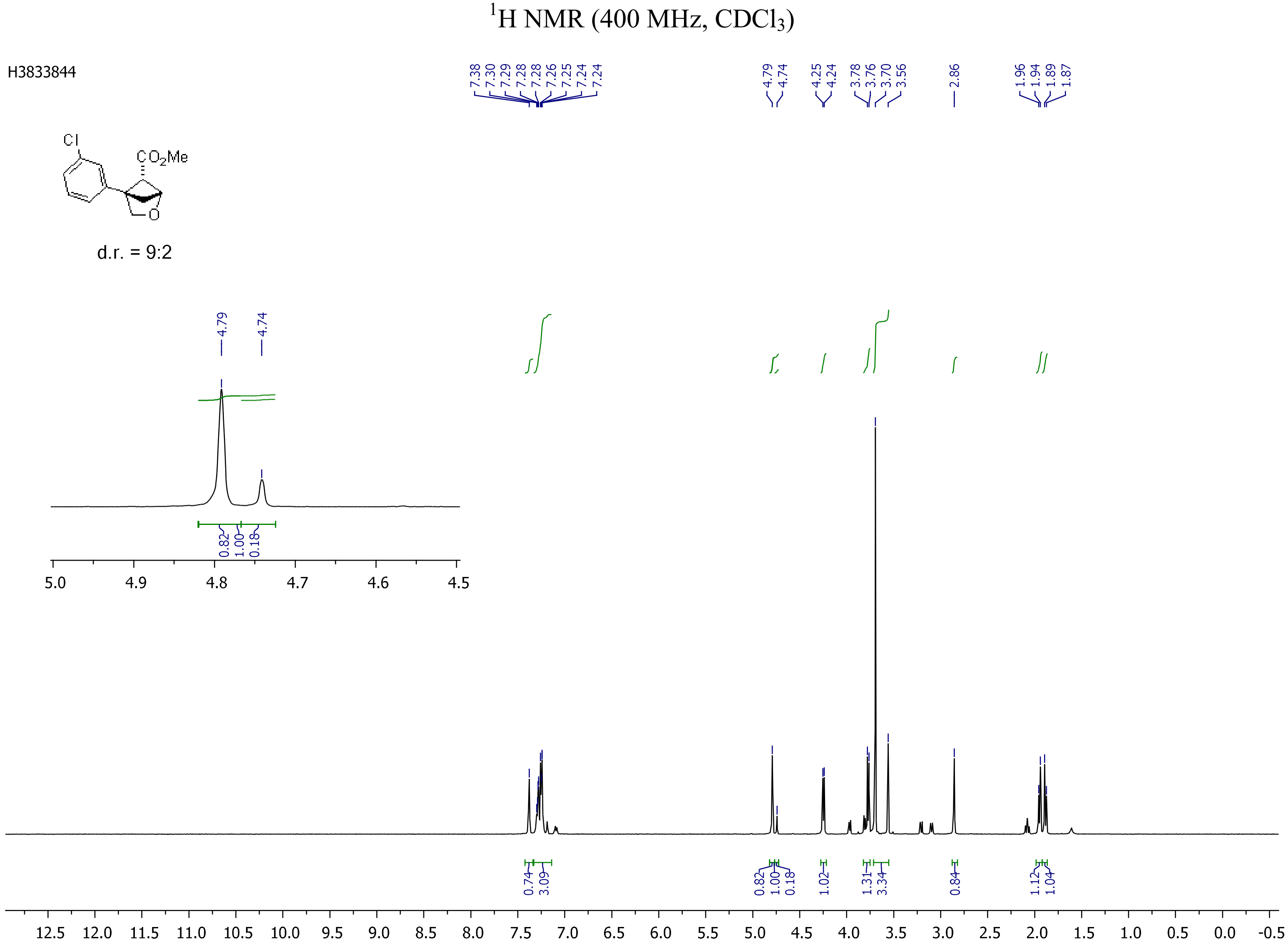

$^{13}C\{^{1}H\}$ NMR (126 MHz, $CDCl_3$)

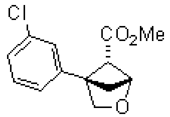

d.r. = 9:2

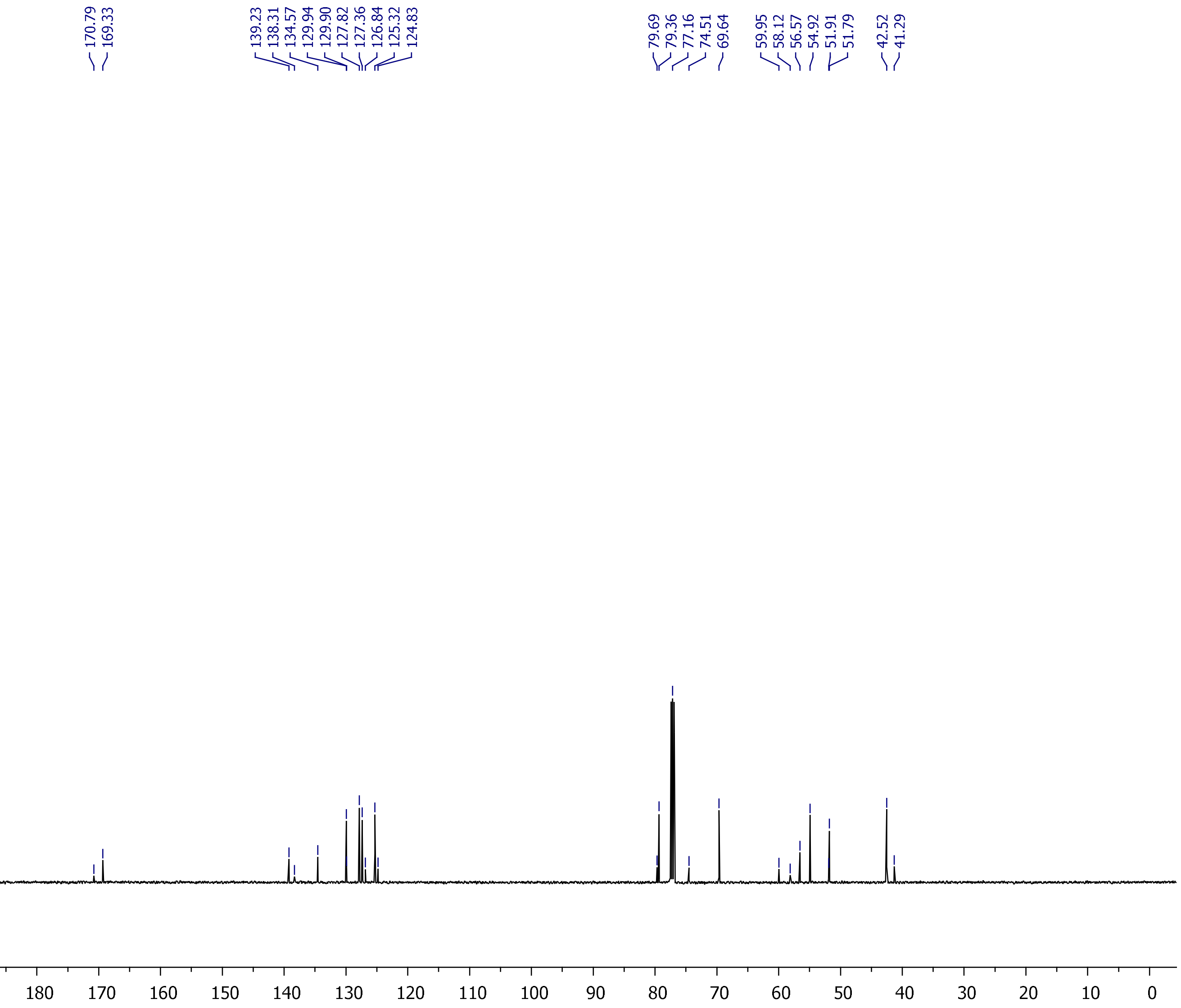

Compound (±)-12b

^{1}H NMR (400 MHz, DMSO-d_6)

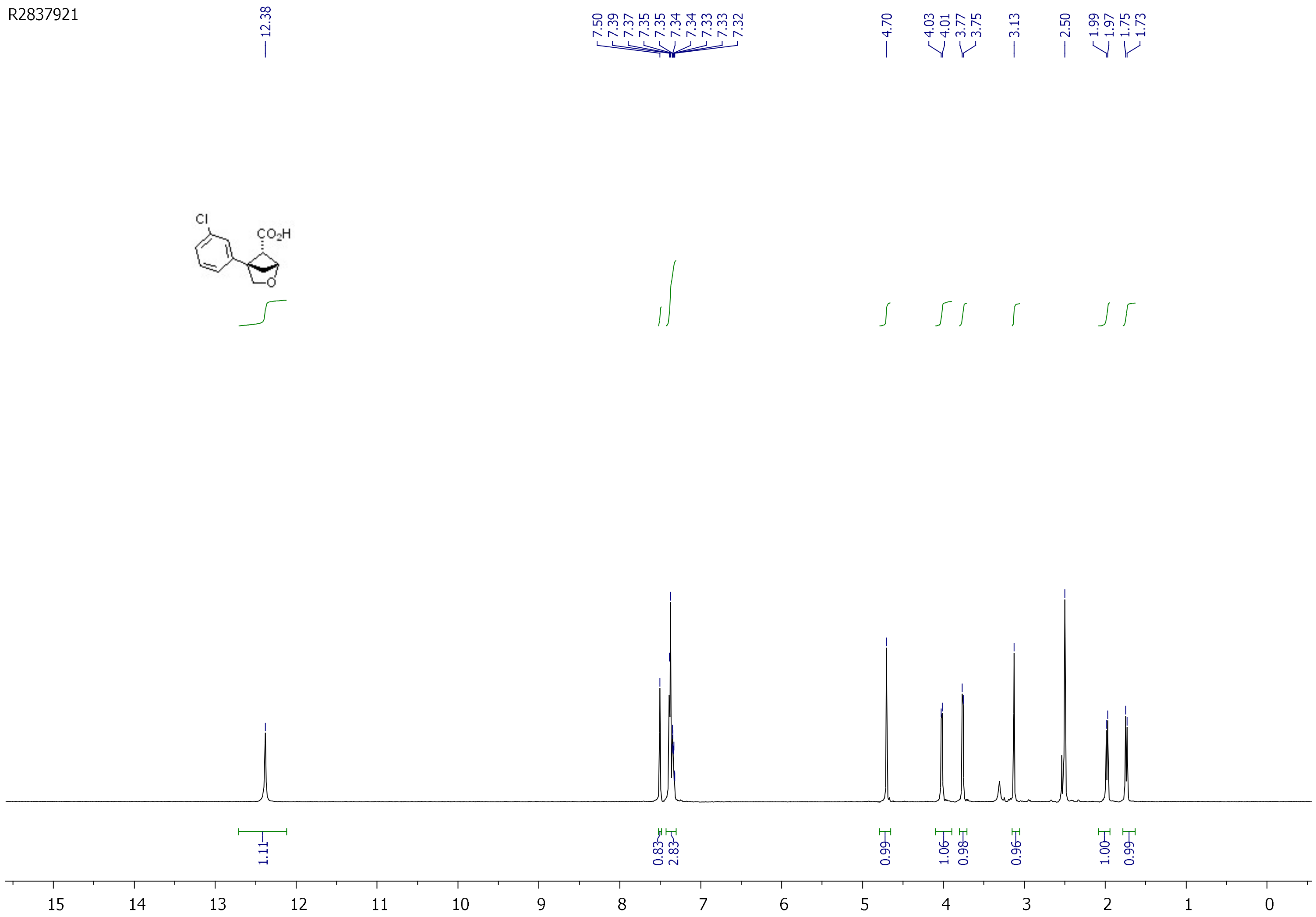

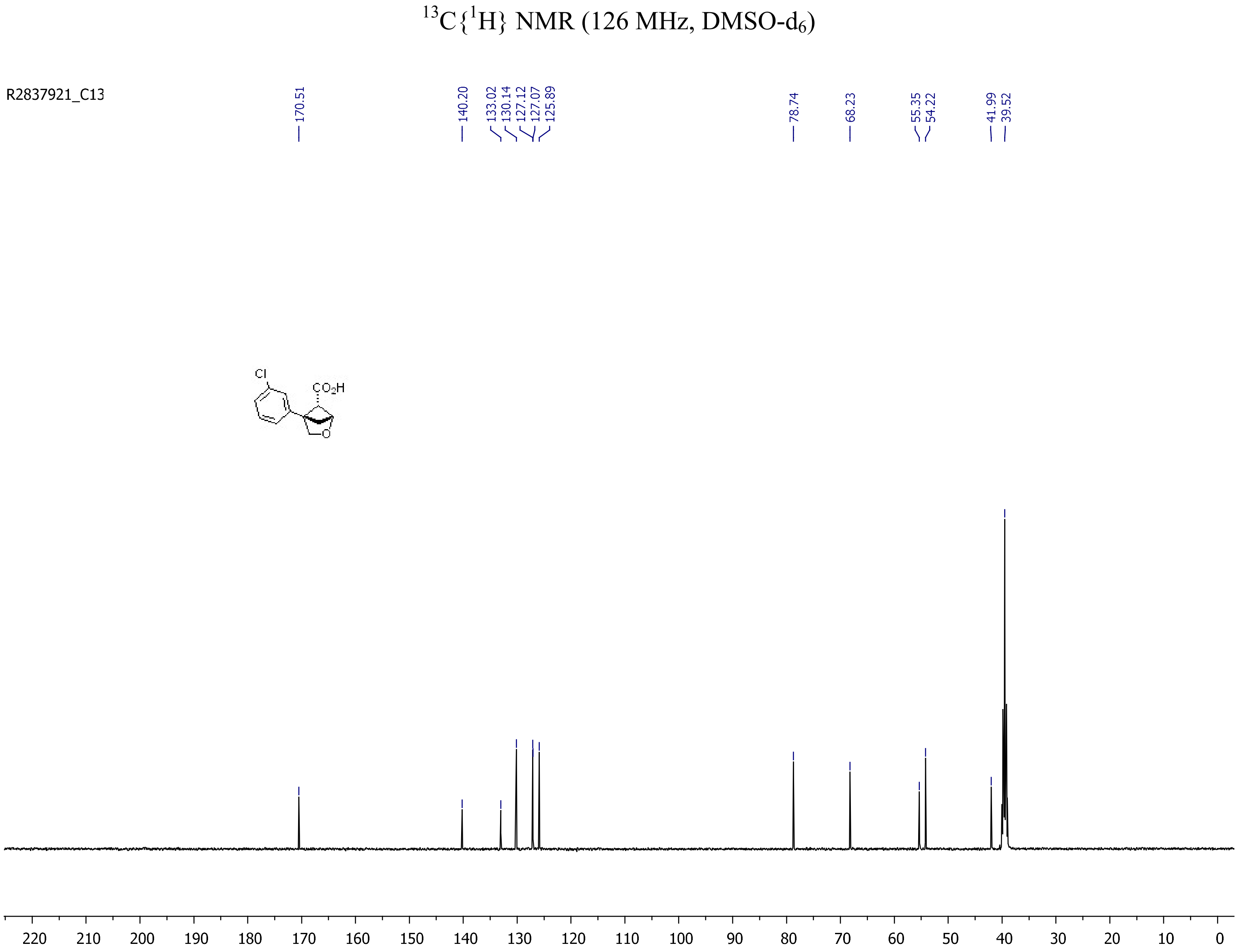

$^{13}C\{^1H\}$ NMR (126 MHz, DMSO-d_6)
R2837921_C13
170.51
140.20
133.02
130.14
127.12
127.07
125.89
78.74
68.23
55.35
54.22
41.99
39.52
Cl
CO2H
O
220 210 200 190 180 170 160 150 140 130 120 110 100 90 80 70 60 50 40 30 20 10 0

2-(4-Chlorophenyl)prop-2-en-1-ol (ca. 90% purity)

^{1}H NMR (500 MHz, DMSO-d_6)

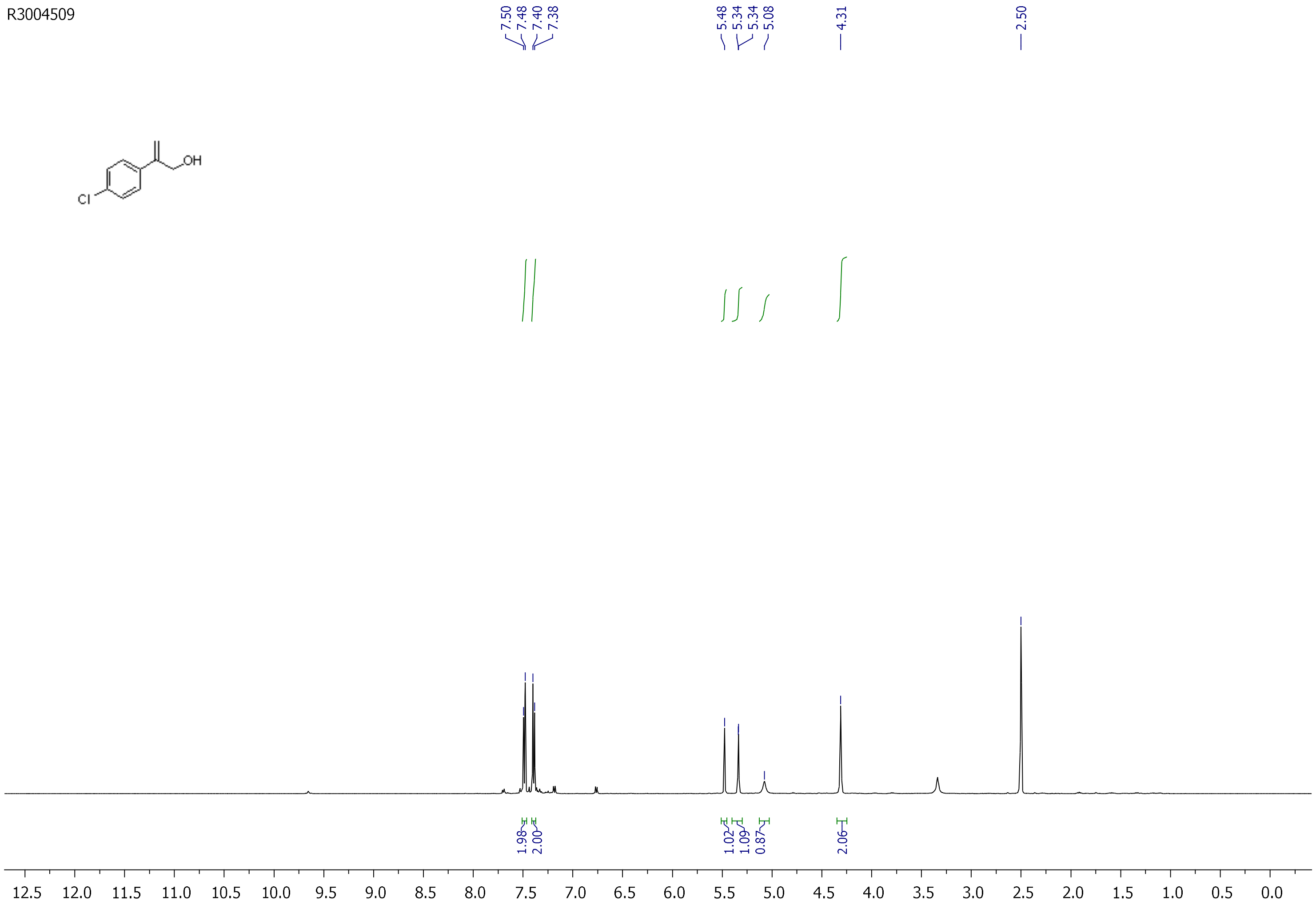

$^{13}C\{^1H\}$ NMR (151 MHz, DMSO-d_6)

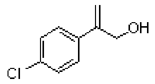

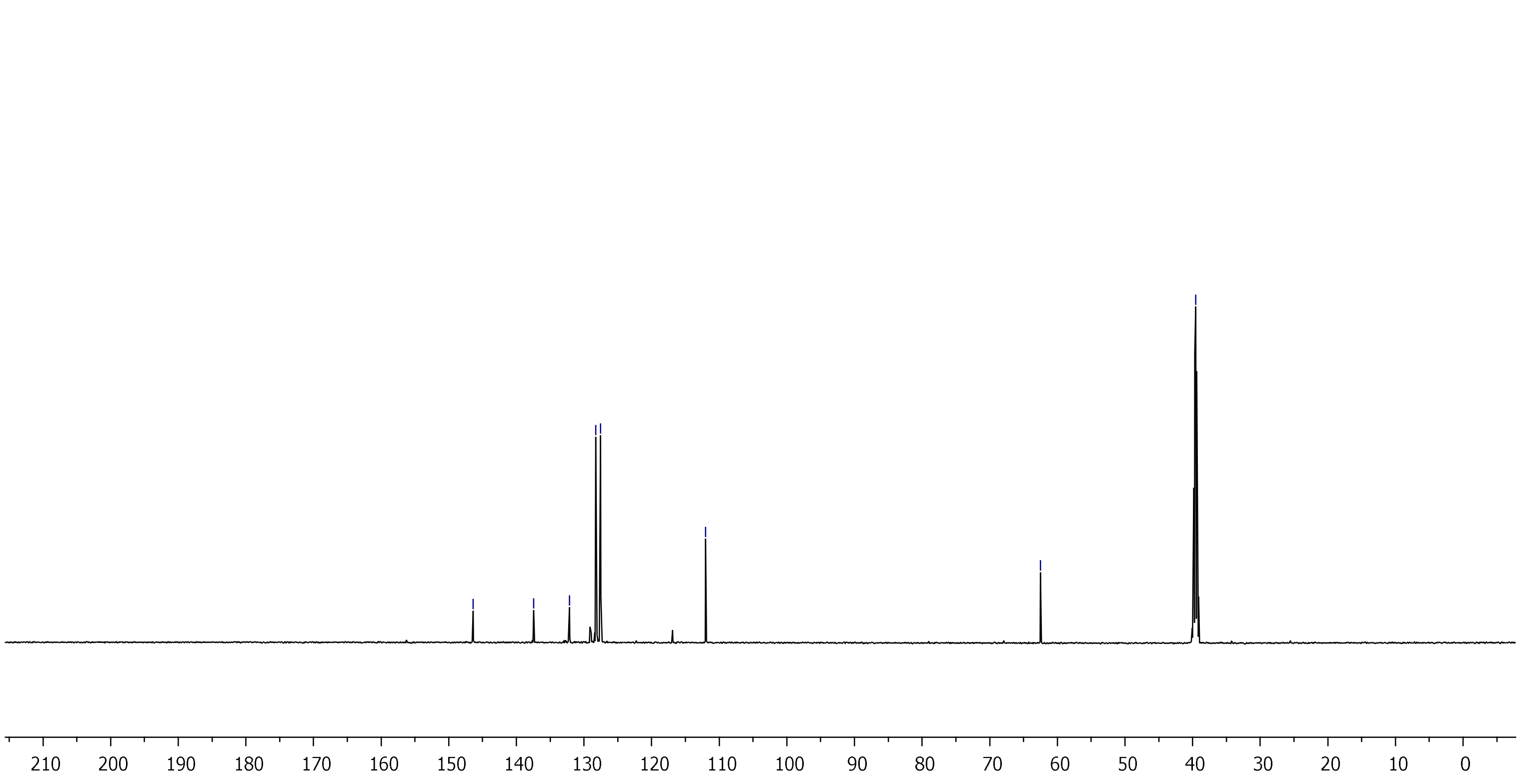

Compound (±)-13a, d.r. = 4:1

^{1}H NMR (400 MHz, $CDCl_3$)

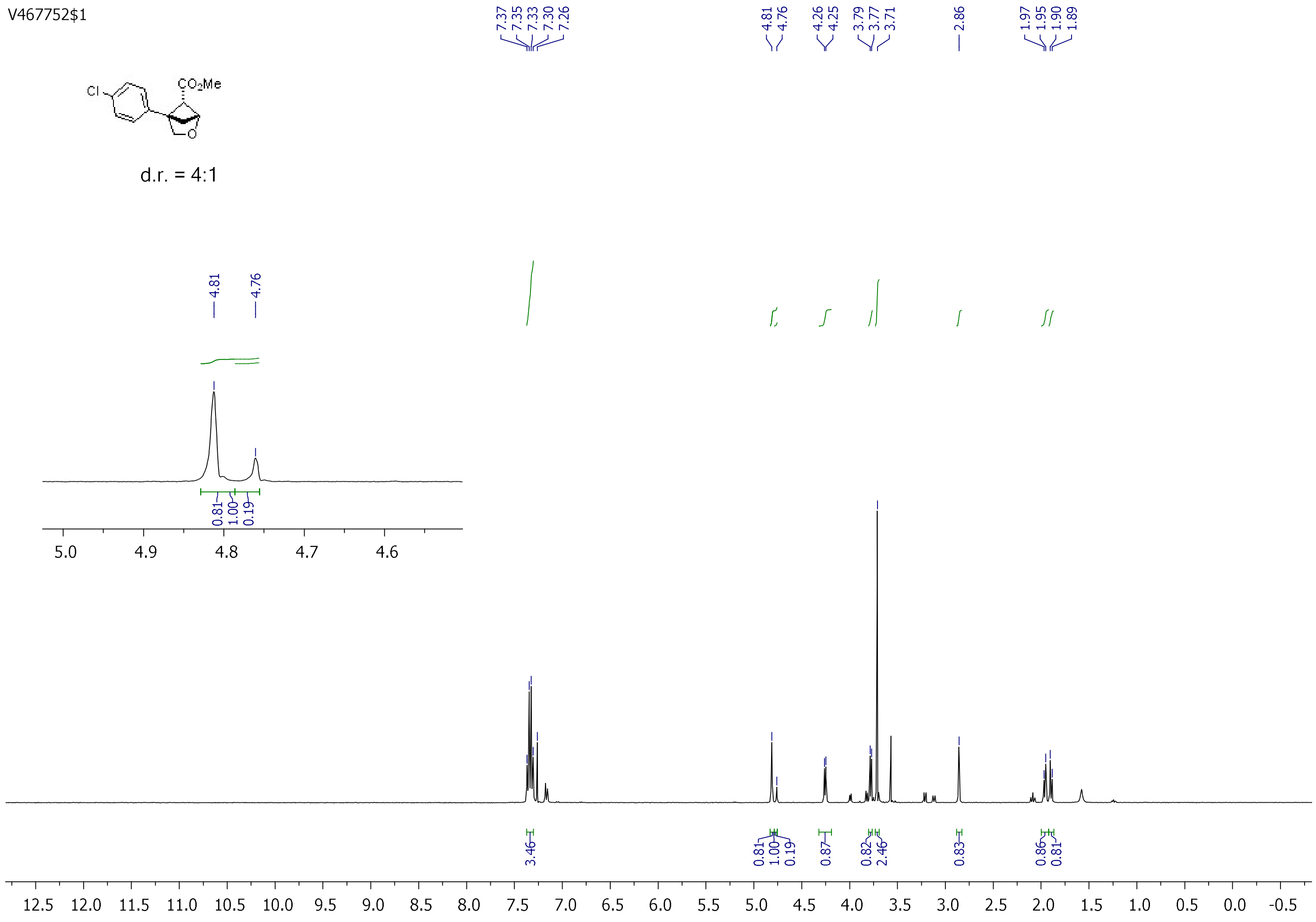

$^{13}C\{^1H\}$ NMR (151 MHz, $CDCl_3$)

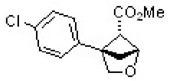

d.r. = 4:1

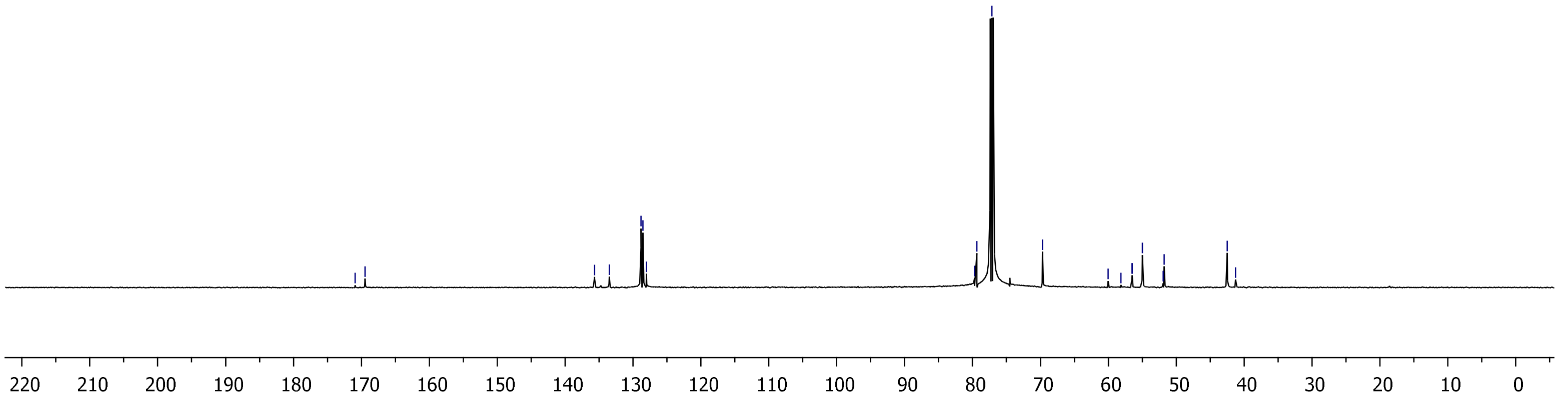

Compound (±)-13b (d.r. = 7:1)

^{1}H NMR (400 MHz, DMSO-d$_6$)

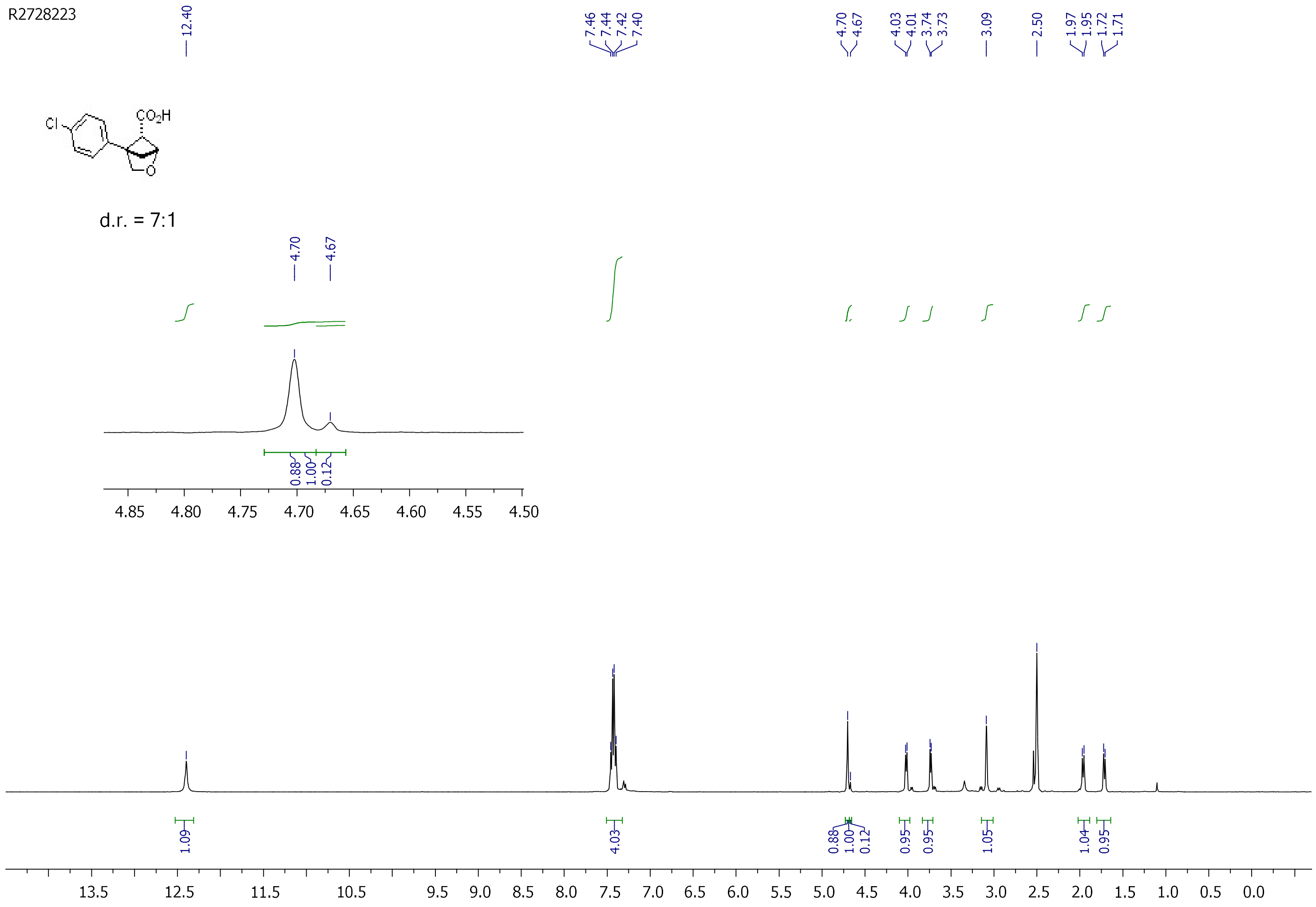

$^{13}C\{^{1}H\}$ NMR (126 MHz, DMSO-d_6)

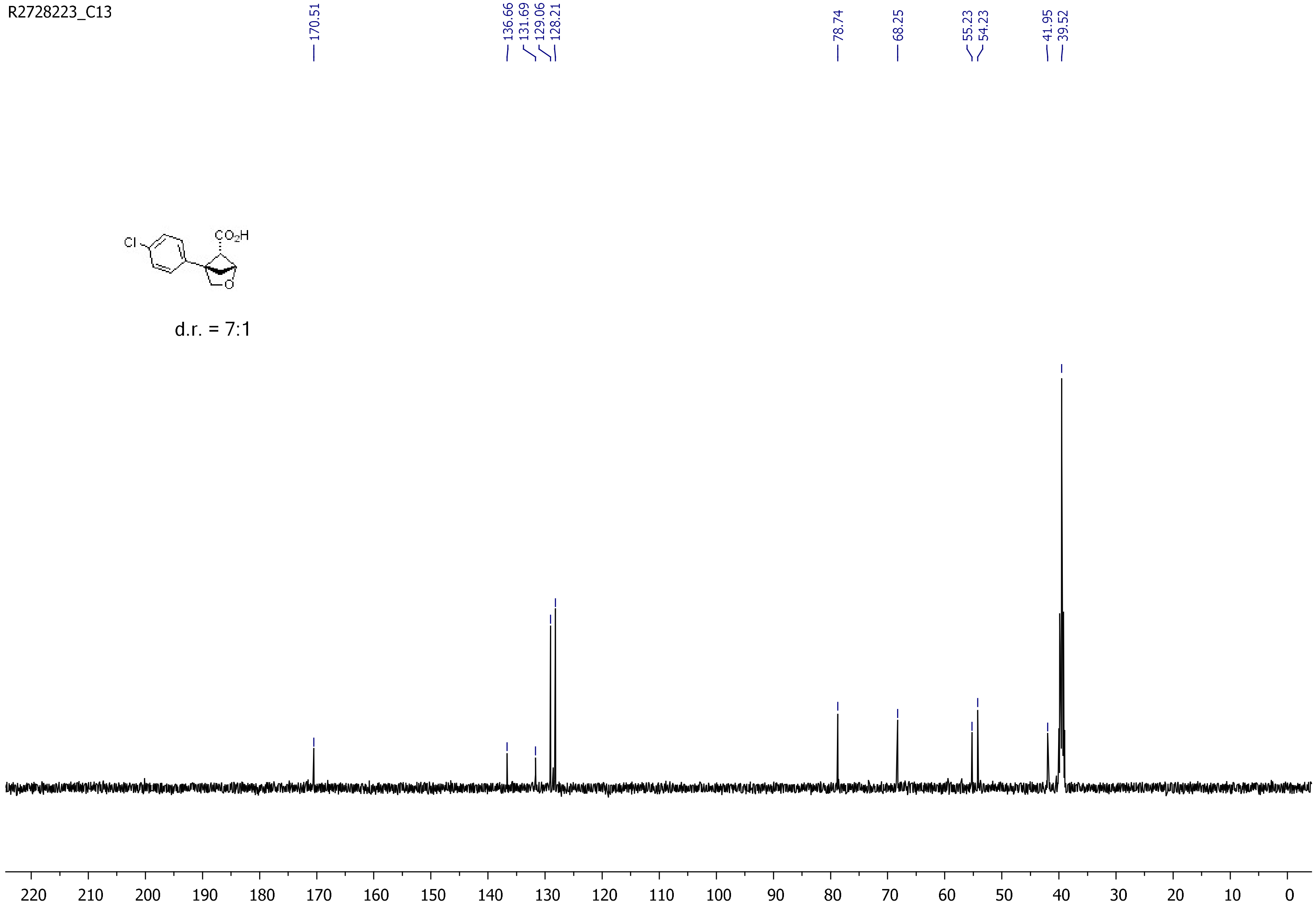

2-(2-Methoxyphenyl)prop-2-en-1-ol

^{1}H NMR (400 MHz, $CDCl_3$)

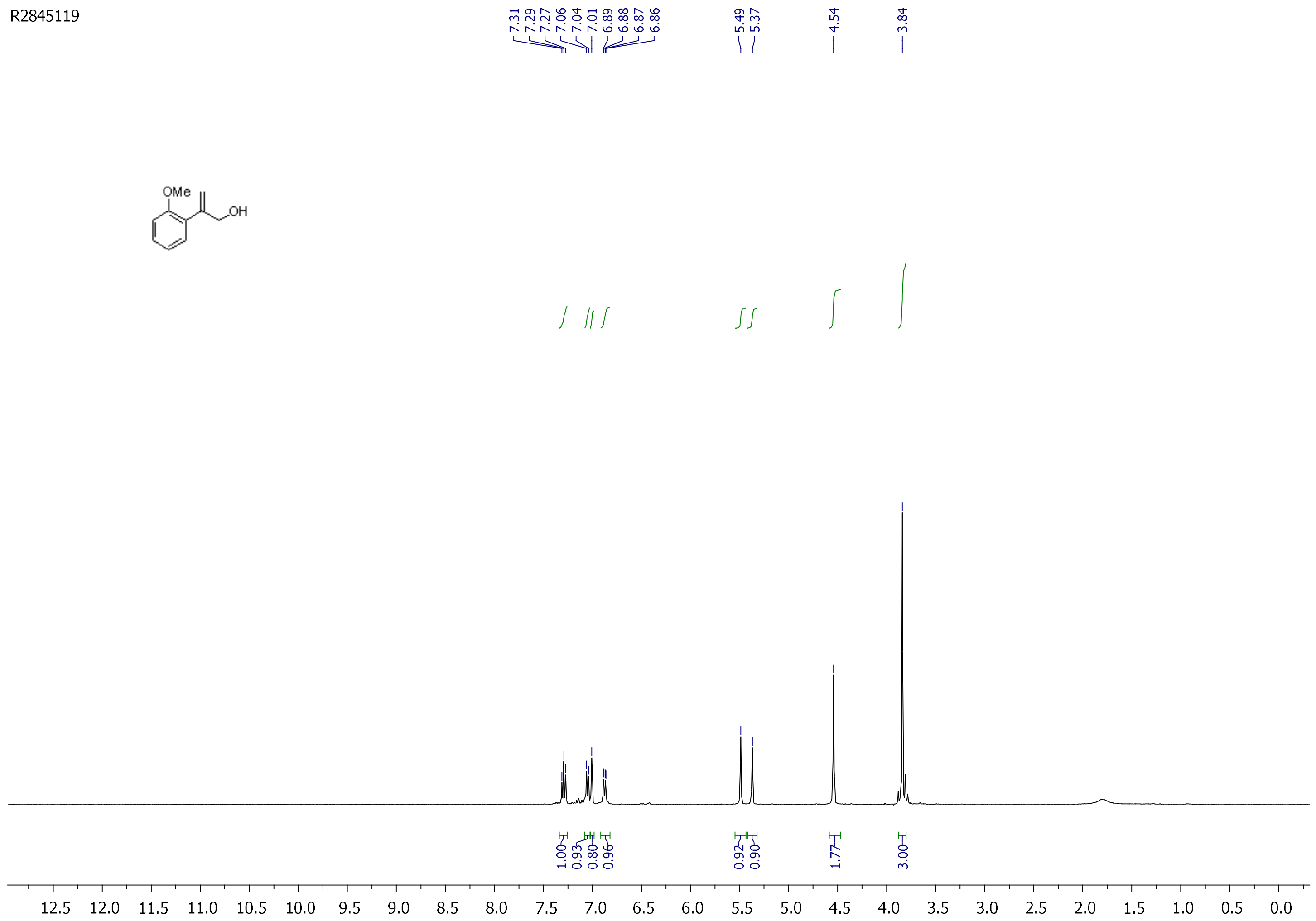

$^{13}C\{^{1}H\}$ NMR (126 MHz, $CDCl_3$)

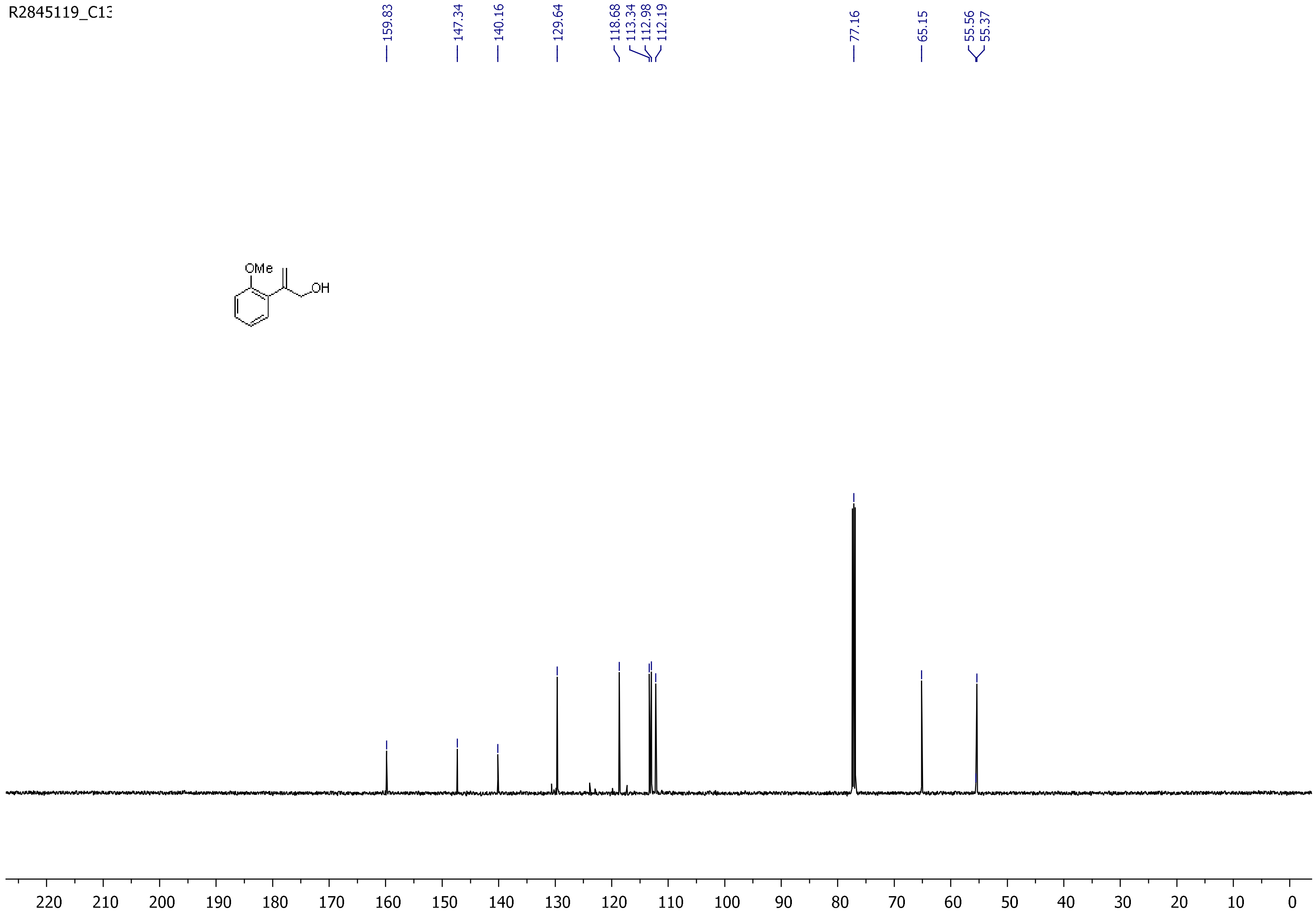

Compound (±)-14a

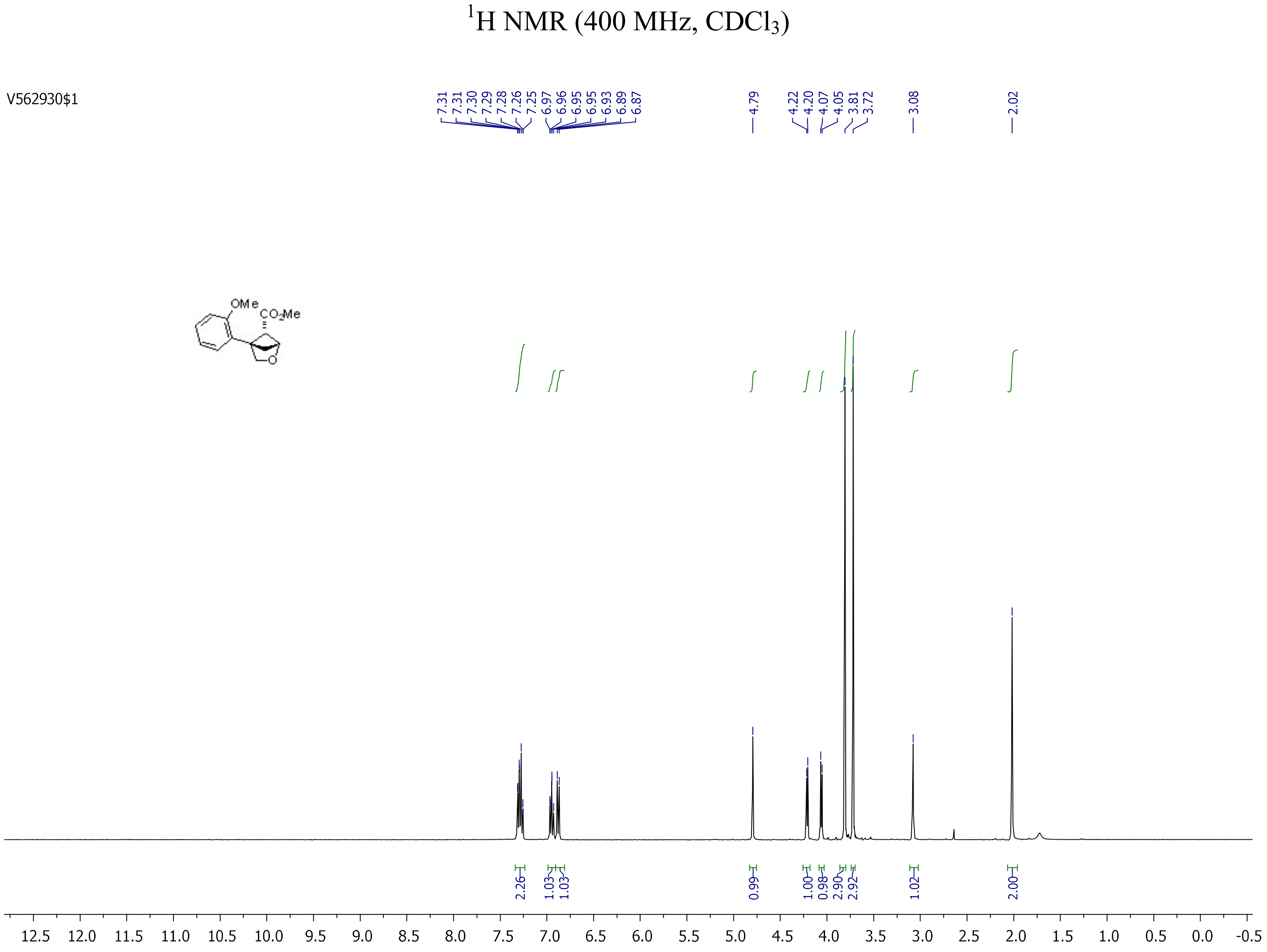

$^{13}C\{^{1}H\}$ NMR (126 MHz, $CDCl_3$)

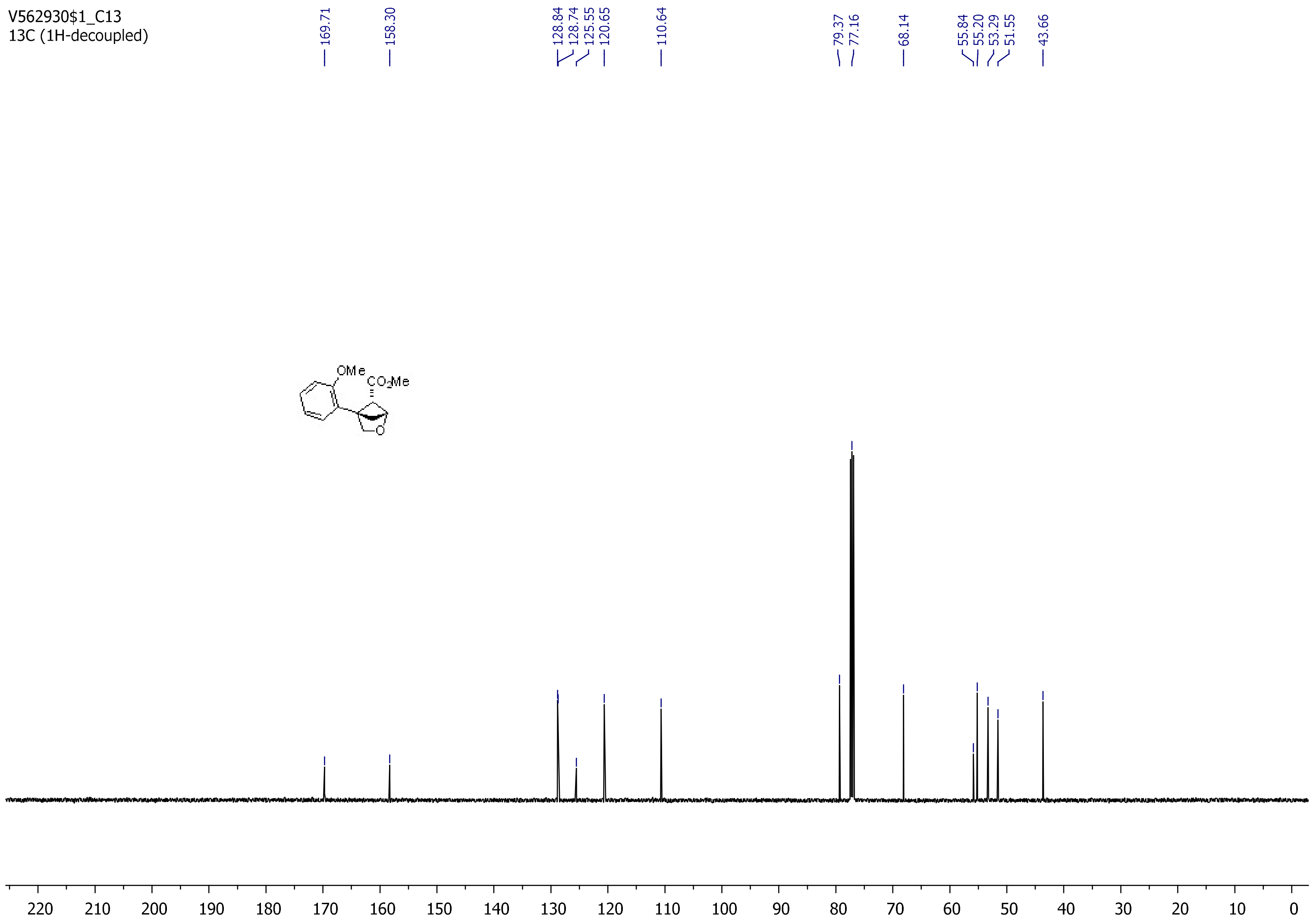

Compound (±)-14b

^{1}H NMR (400 MHz, DMSO-d$_6$)

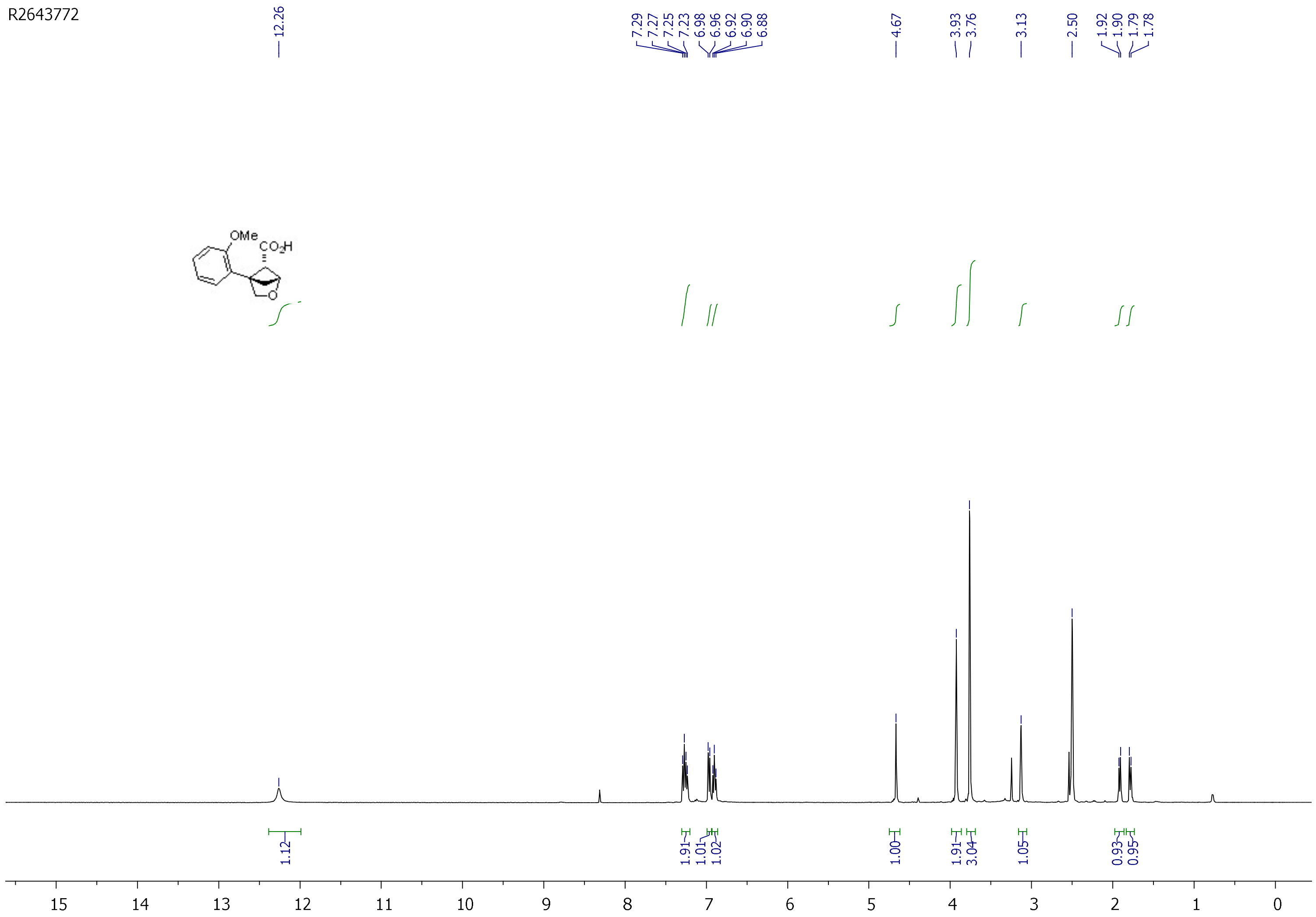

$^{13}C\{^1H\}$ NMR (126 MHz, DMSO-d_6)

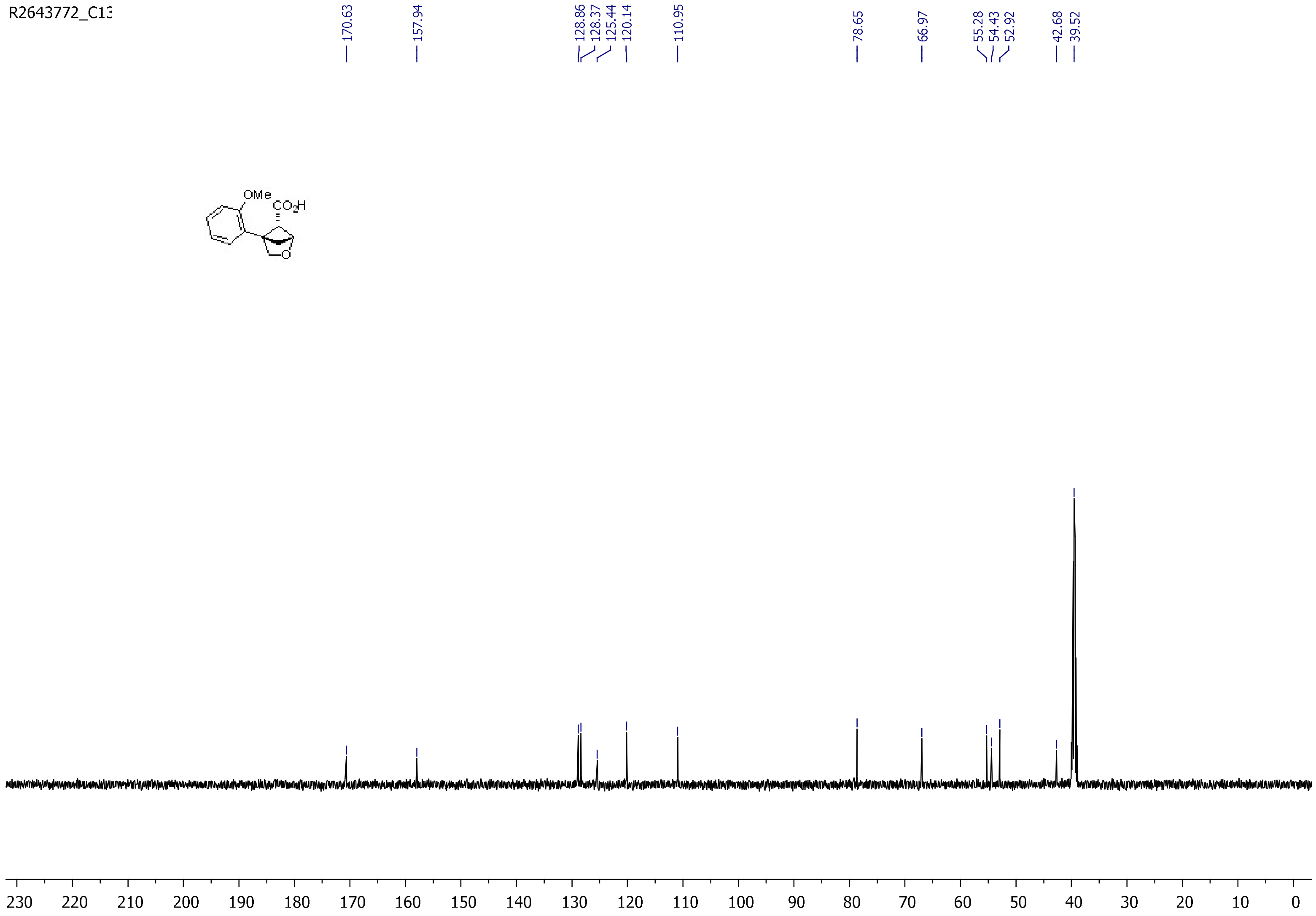

2-(3-Methoxyphenyl)prop-2-en-1-ol

^{1}H NMR (400 MHz, DMSO-d_6)

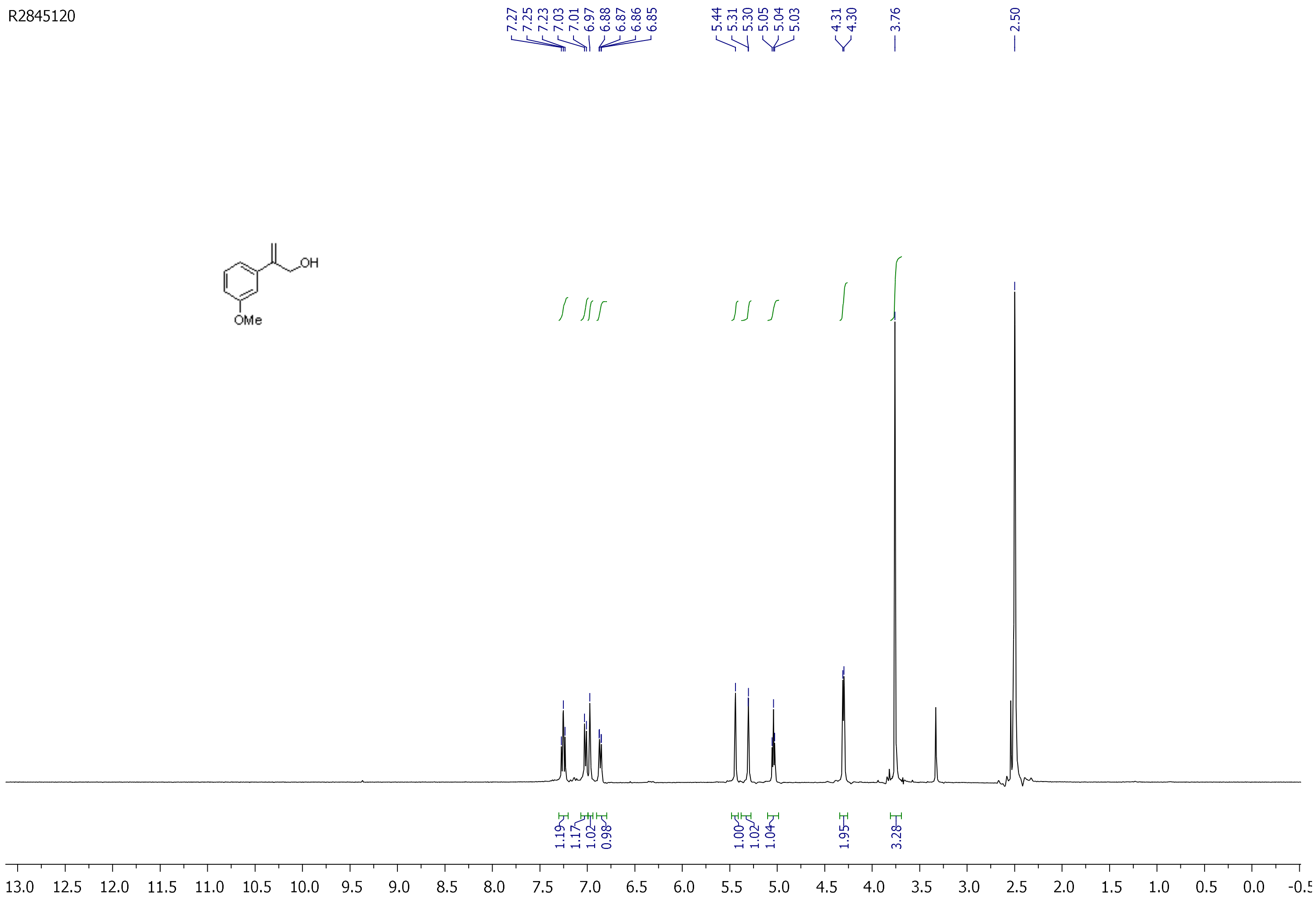

$^{13}C\{^1H\}$ NMR (126 MHz, $CDCl_3$)

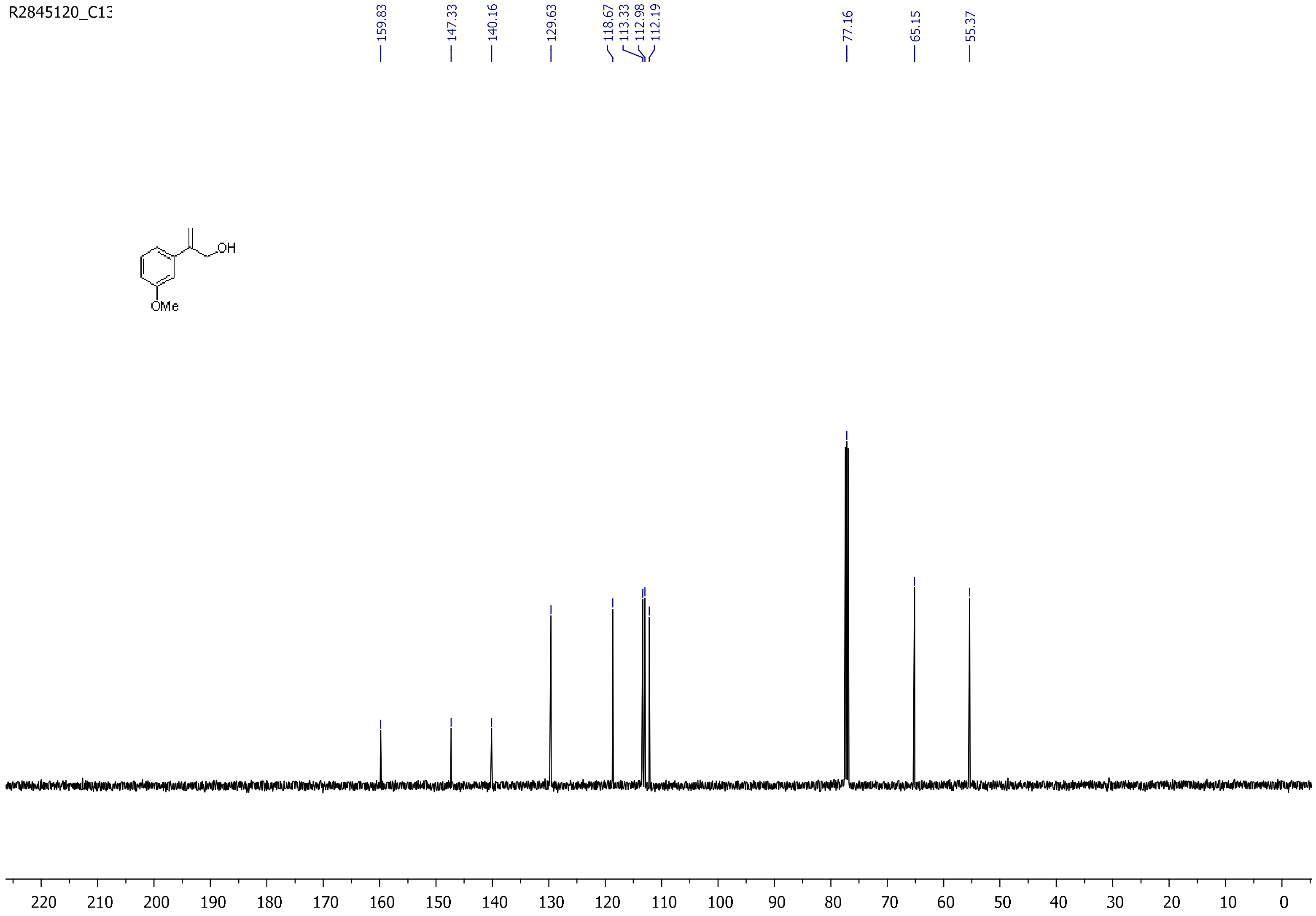

Compound (±)-15a

^{1}H NMR (400 MHz, DMSO-d$_6$)

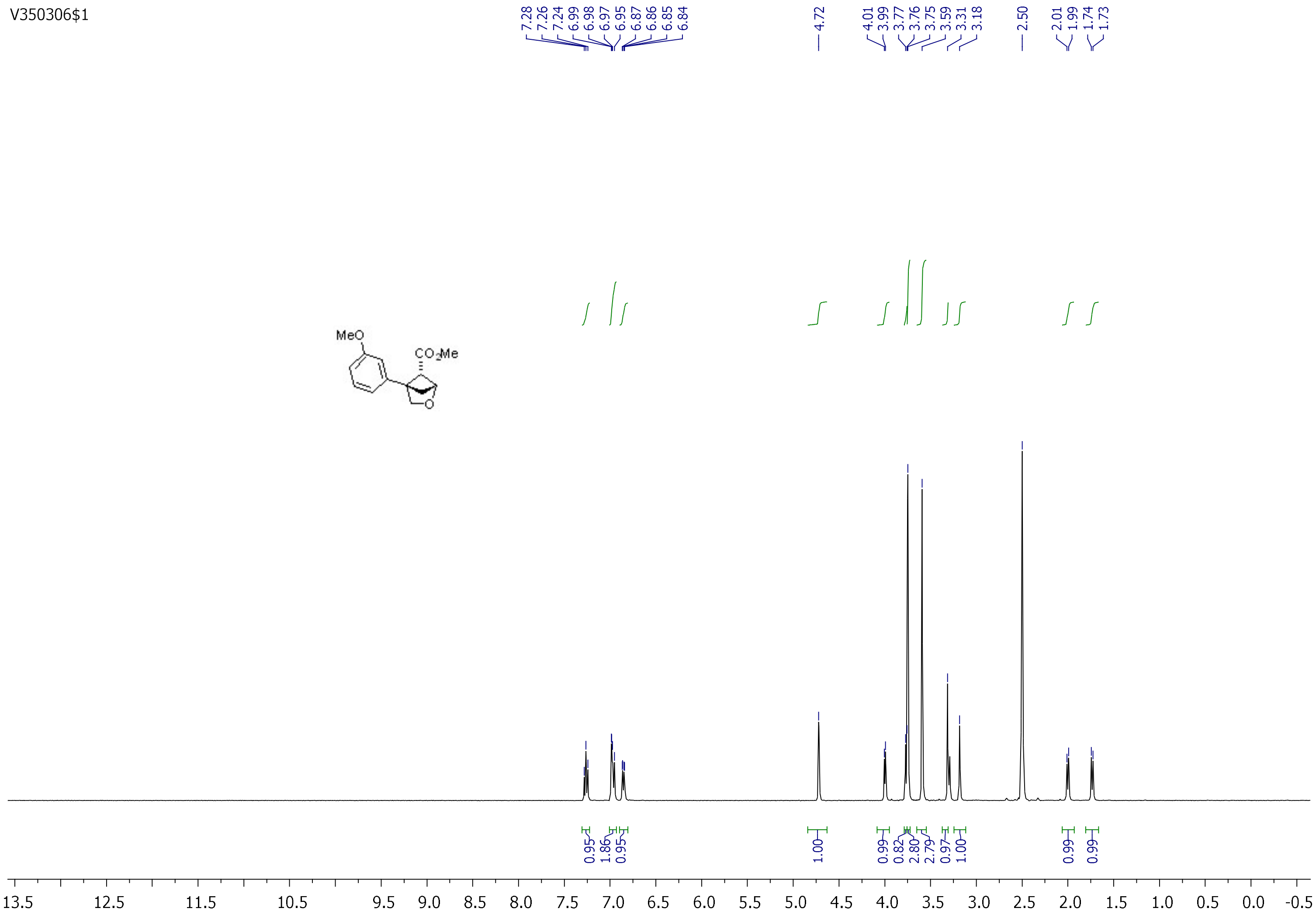

$^{13}C\{^1H\}$ NMR (126 MHz, DMSO-d_6)

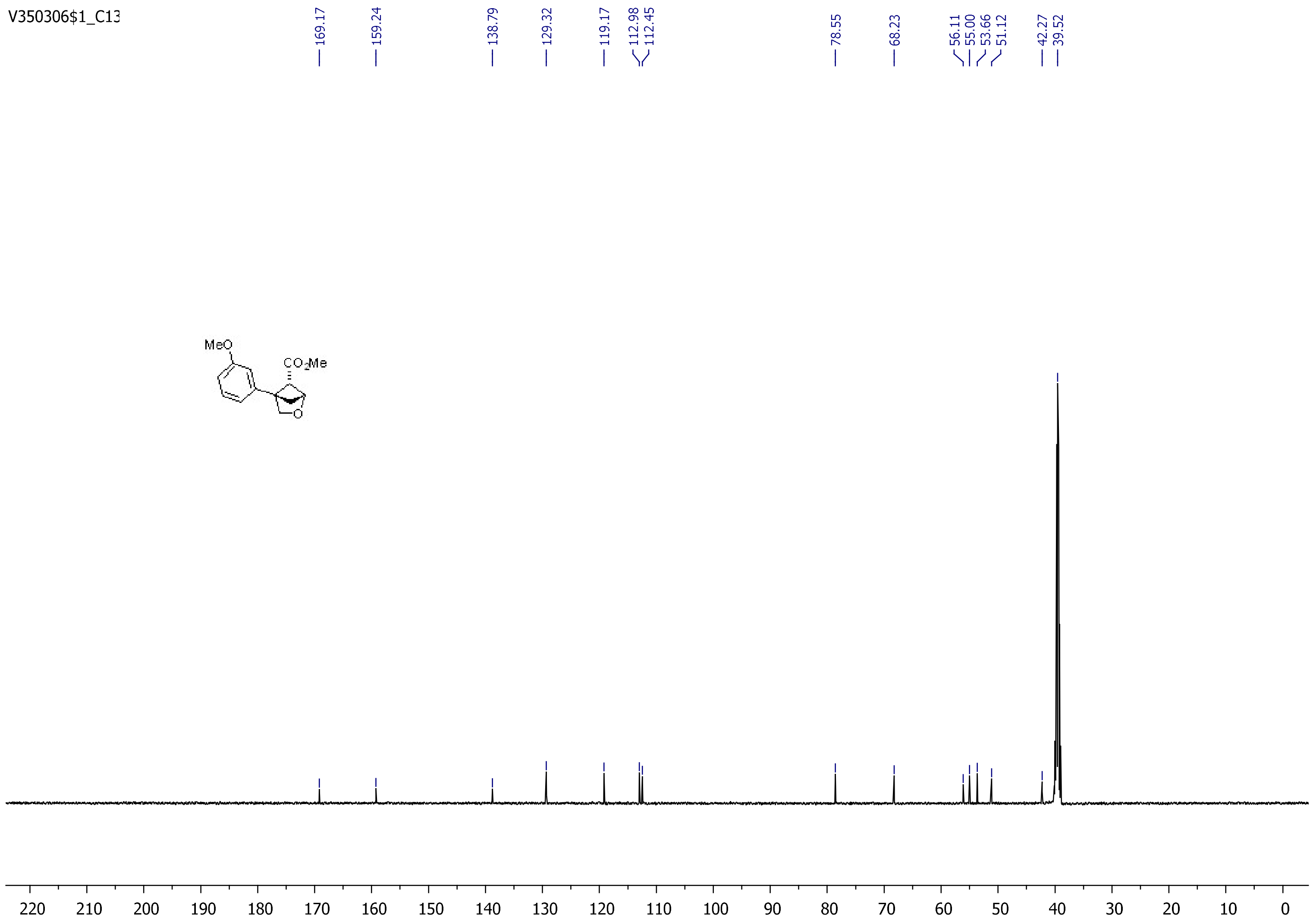

Compound (±)-15b

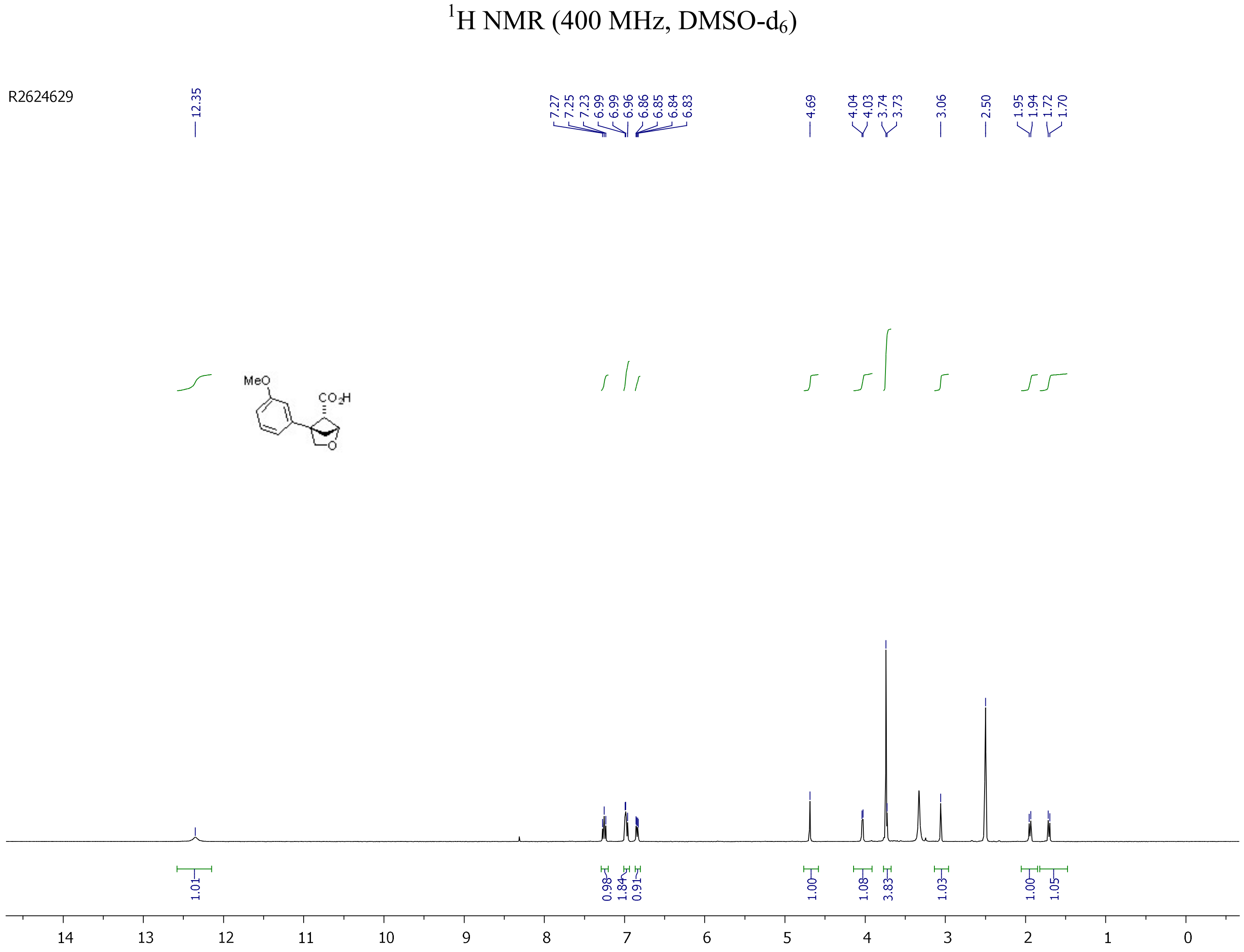

$^{13}C\{^{1}H\}$ NMR (126 MHz, DMSO-d_6)

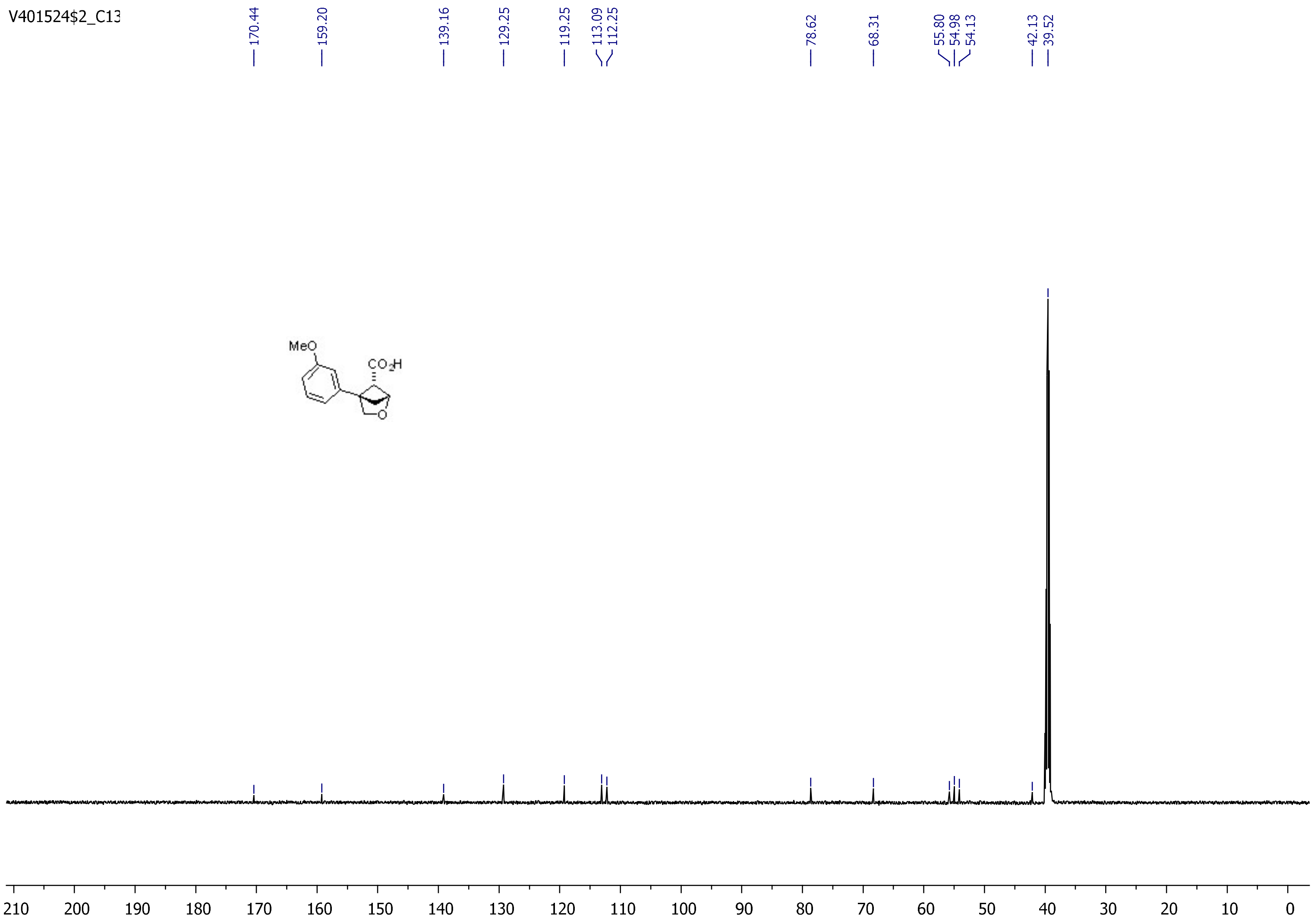

2-(4-Methoxyphenyl)prop-2-en-1-ol

^{1}H NMR (400 MHz, $CDCl_3$)

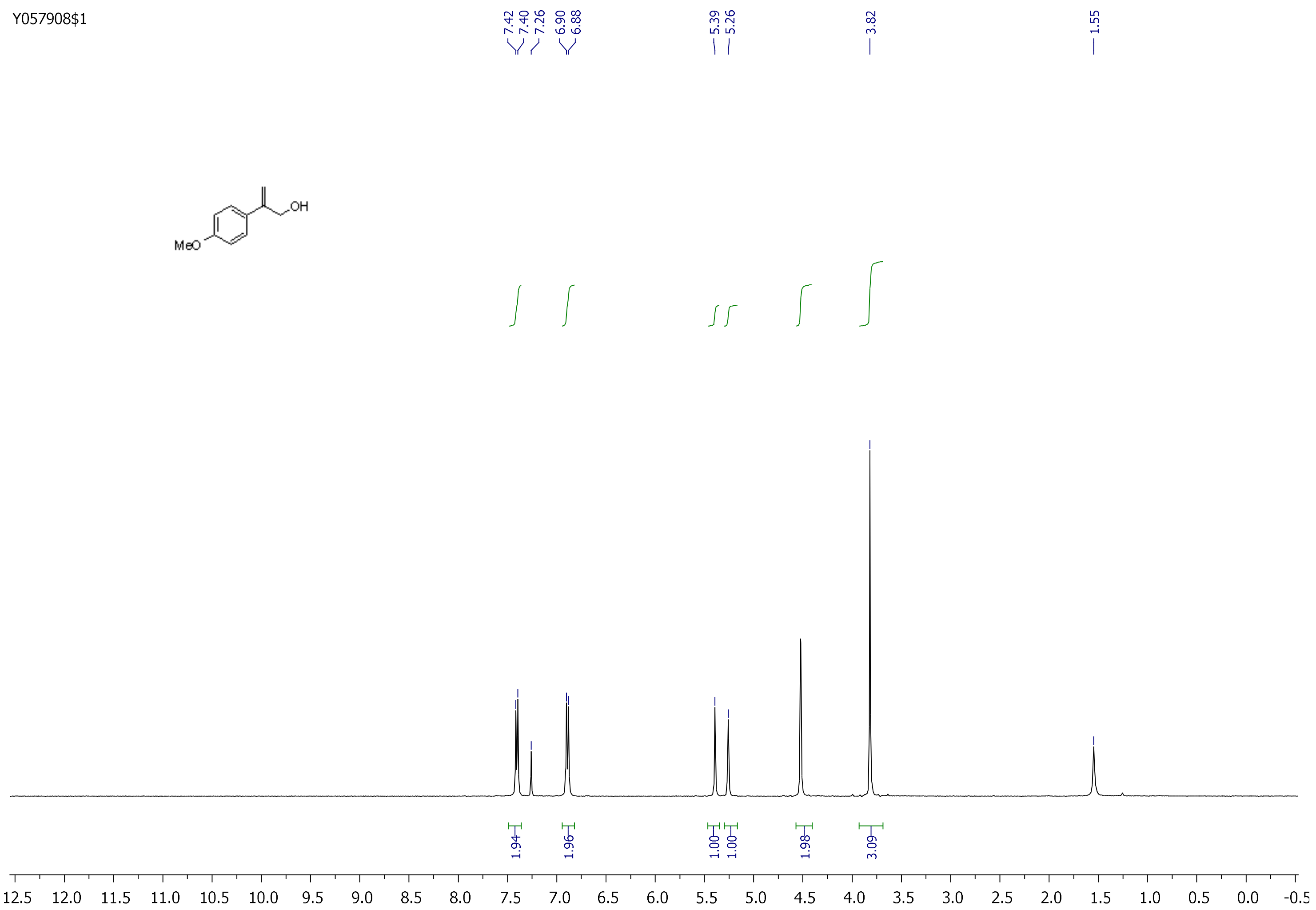

$^{13}C\{^1H\}$ NMR (126 MHz, $CDCl_3$)

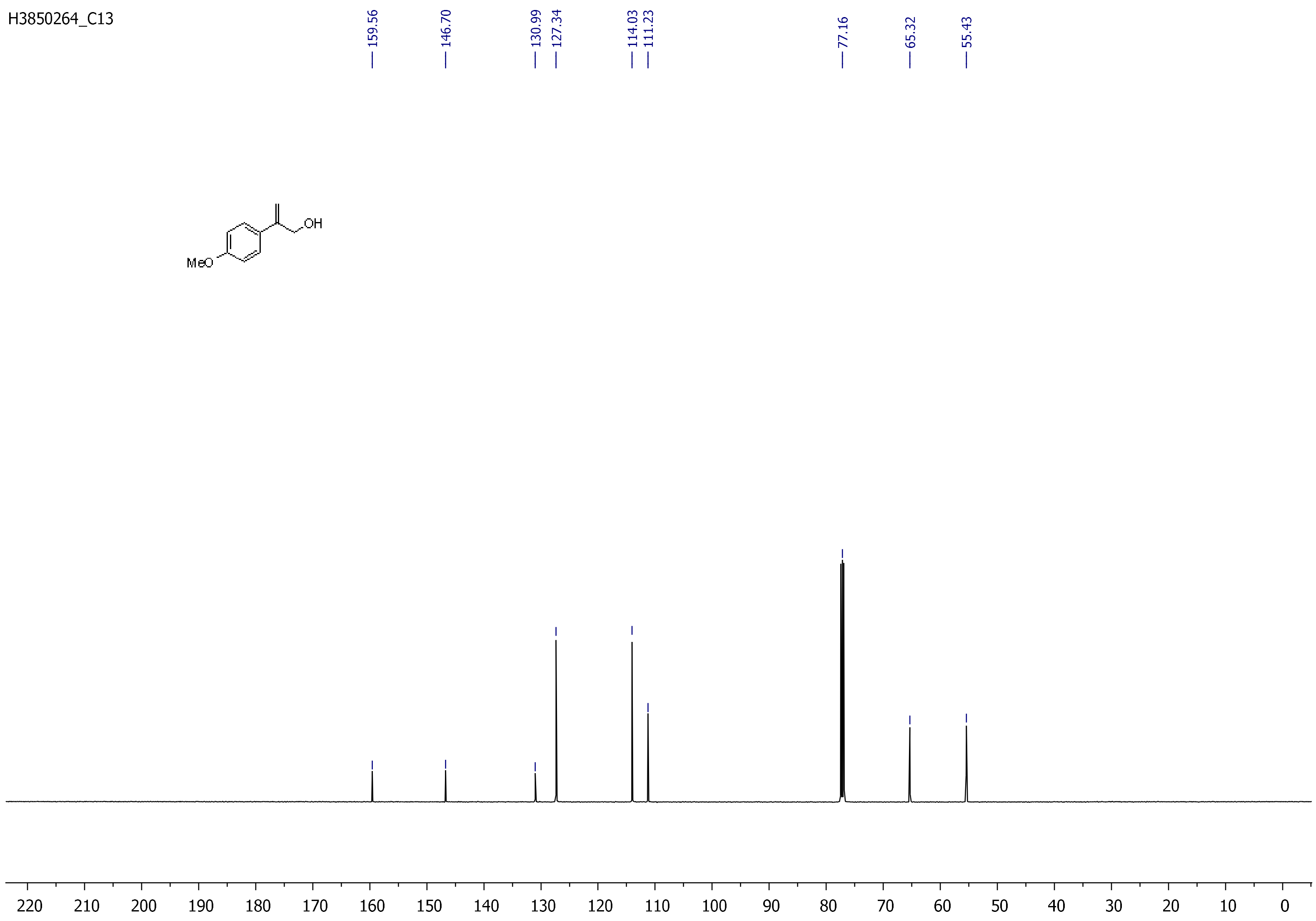

Compound (±)-16a

^{1}H NMR (400 MHz, DMSO-d$_6$)

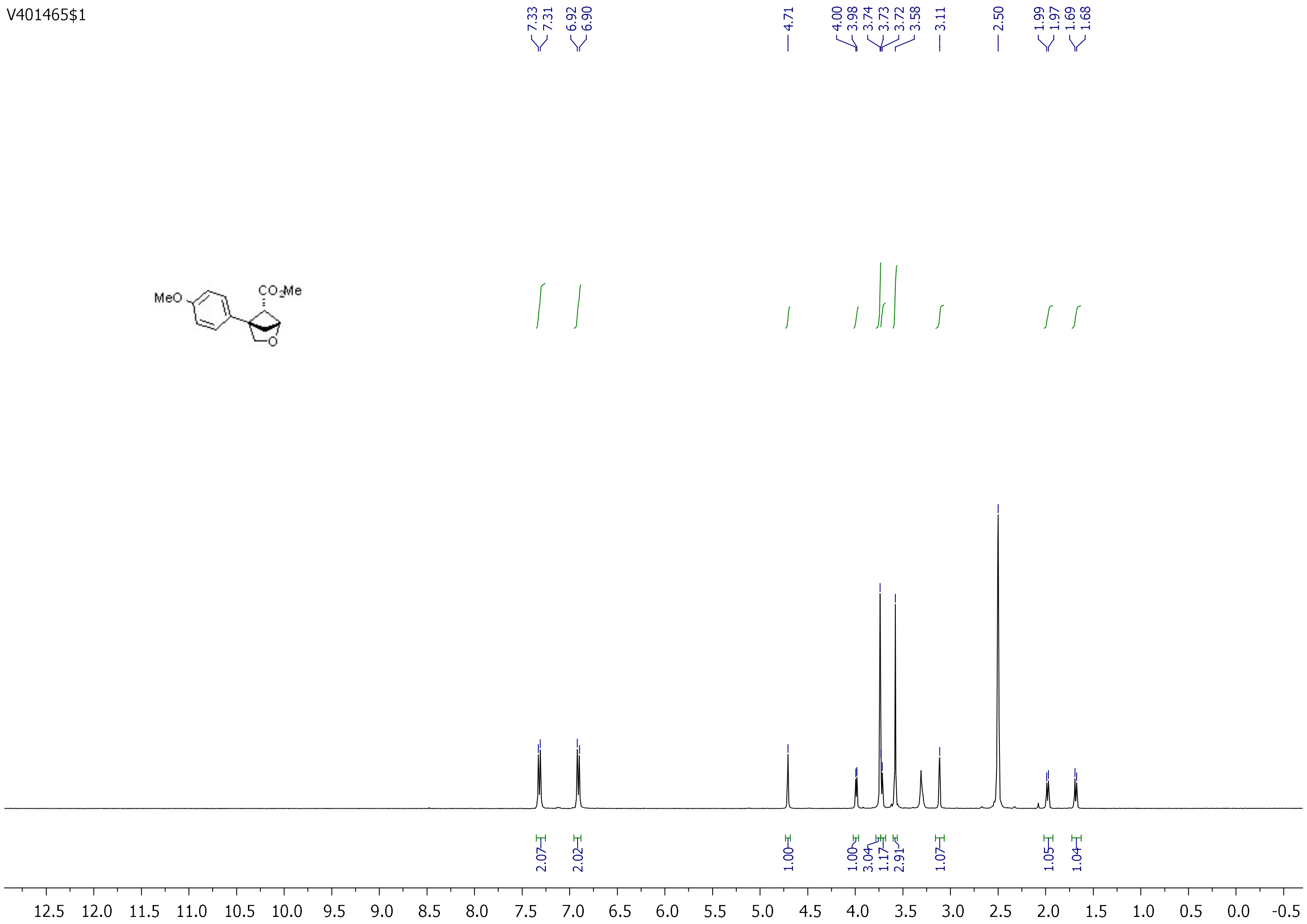

$^{13}C\{^1H\}$ NMR (126 MHz, DMSO-d_6)

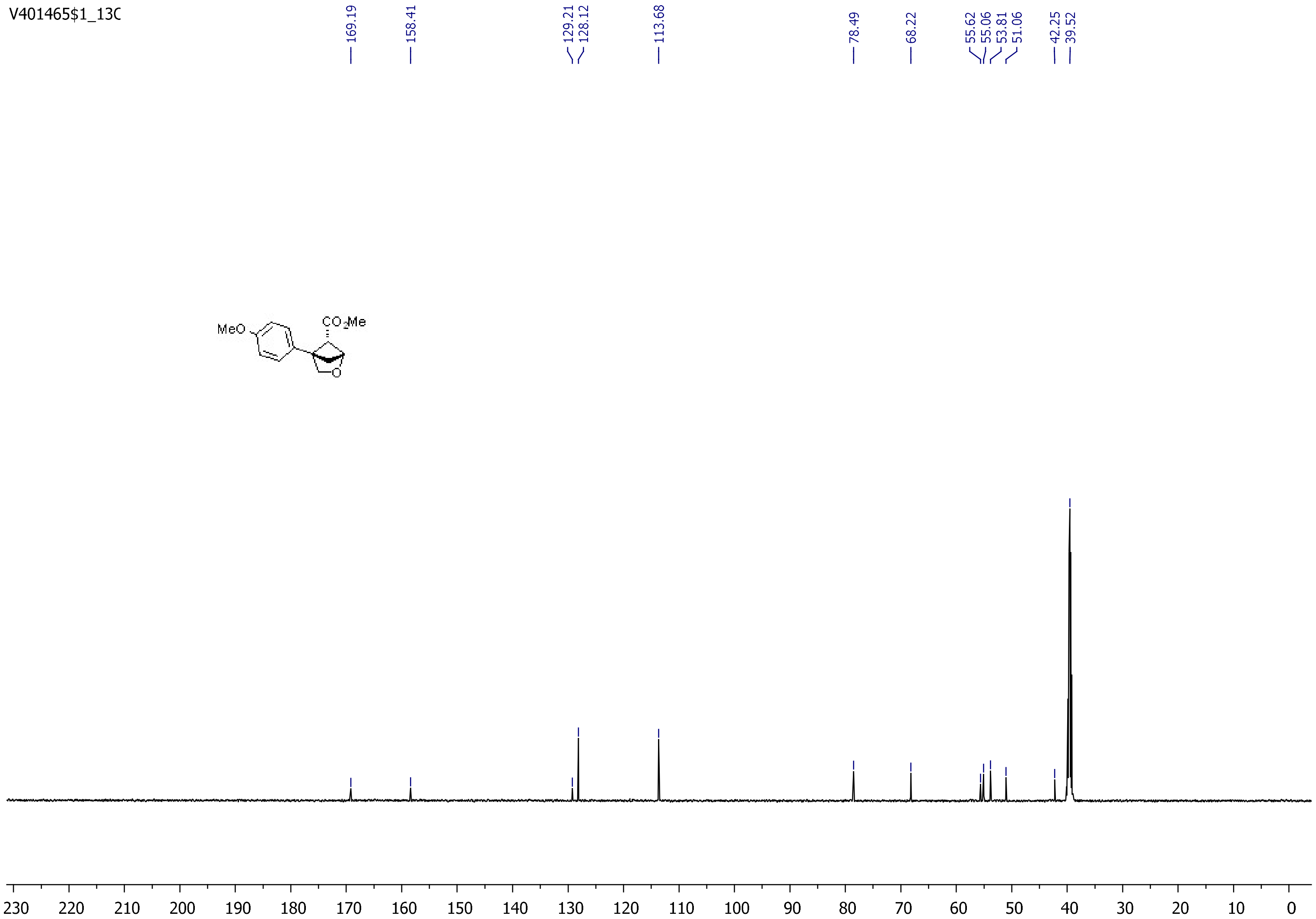

Compound (±)-16b

^{1}H NMR (400 MHz, DMSO-d_6)

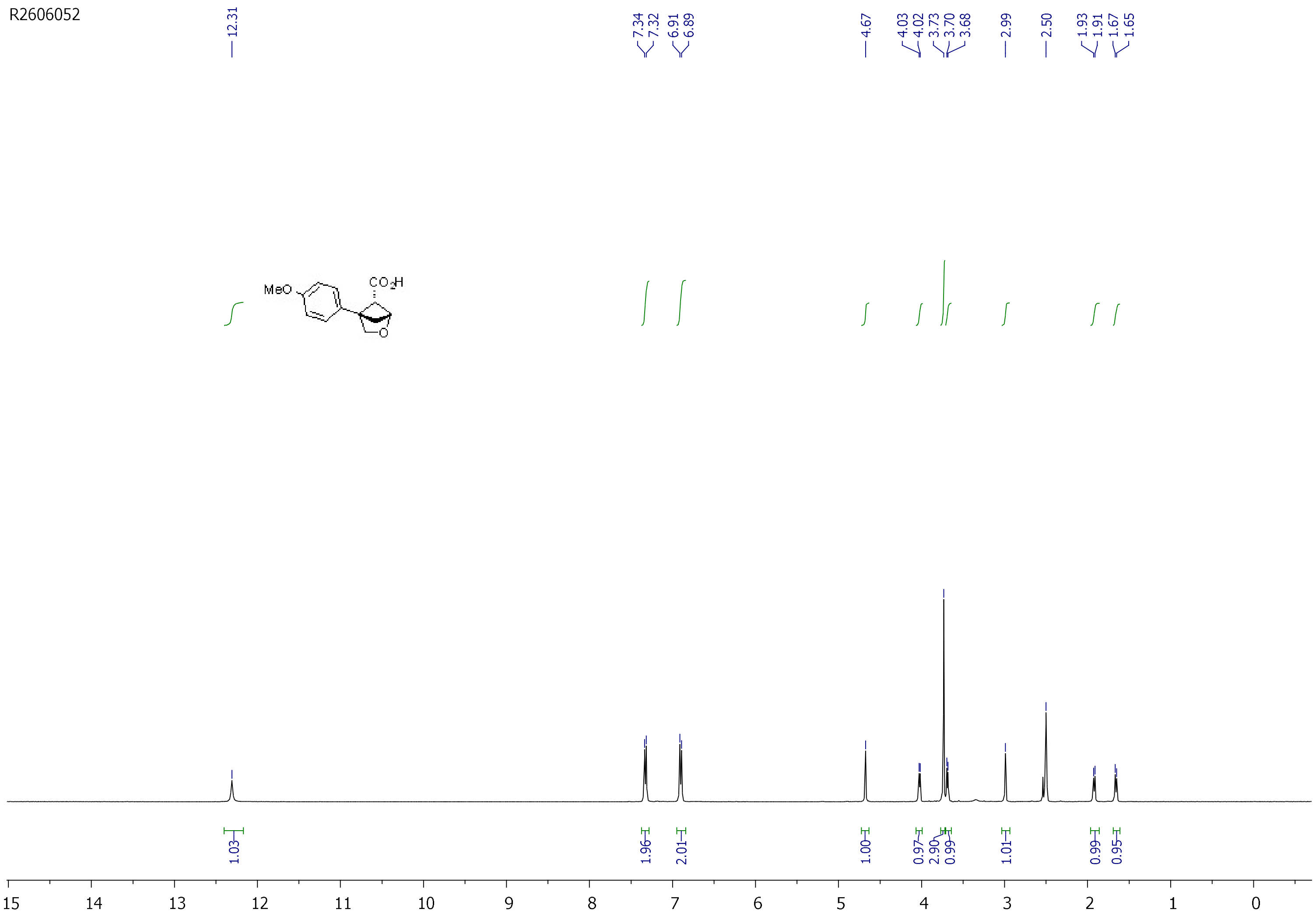

$^{13}C\{^1H\}$ NMR (126 MHz, DMSO-d$_6$)

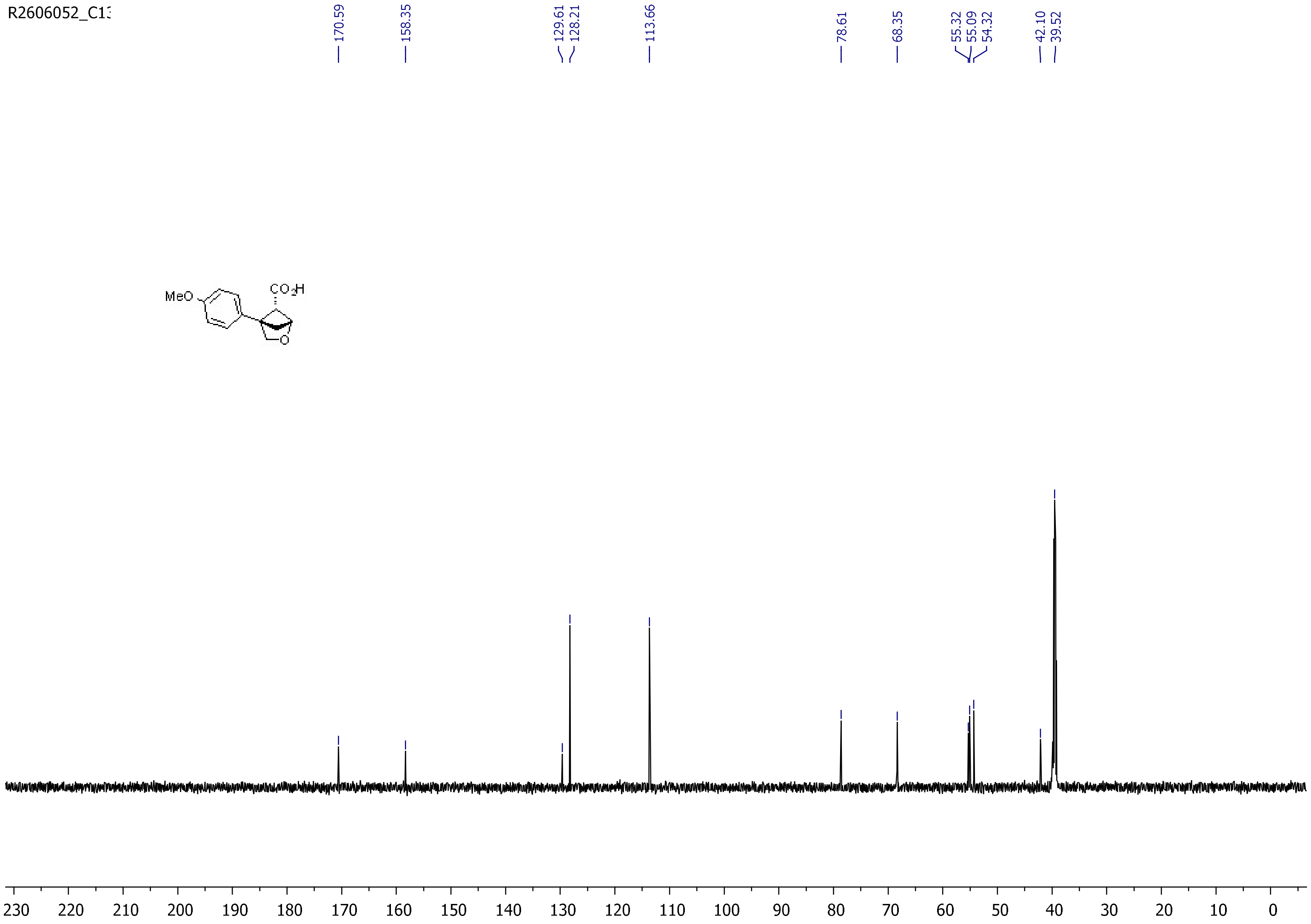

2-(2-(Trifluoromethyl)phenyl)prop-2-en-1-ol

^{1}H NMR (400 MHz, $CDCl_3$)

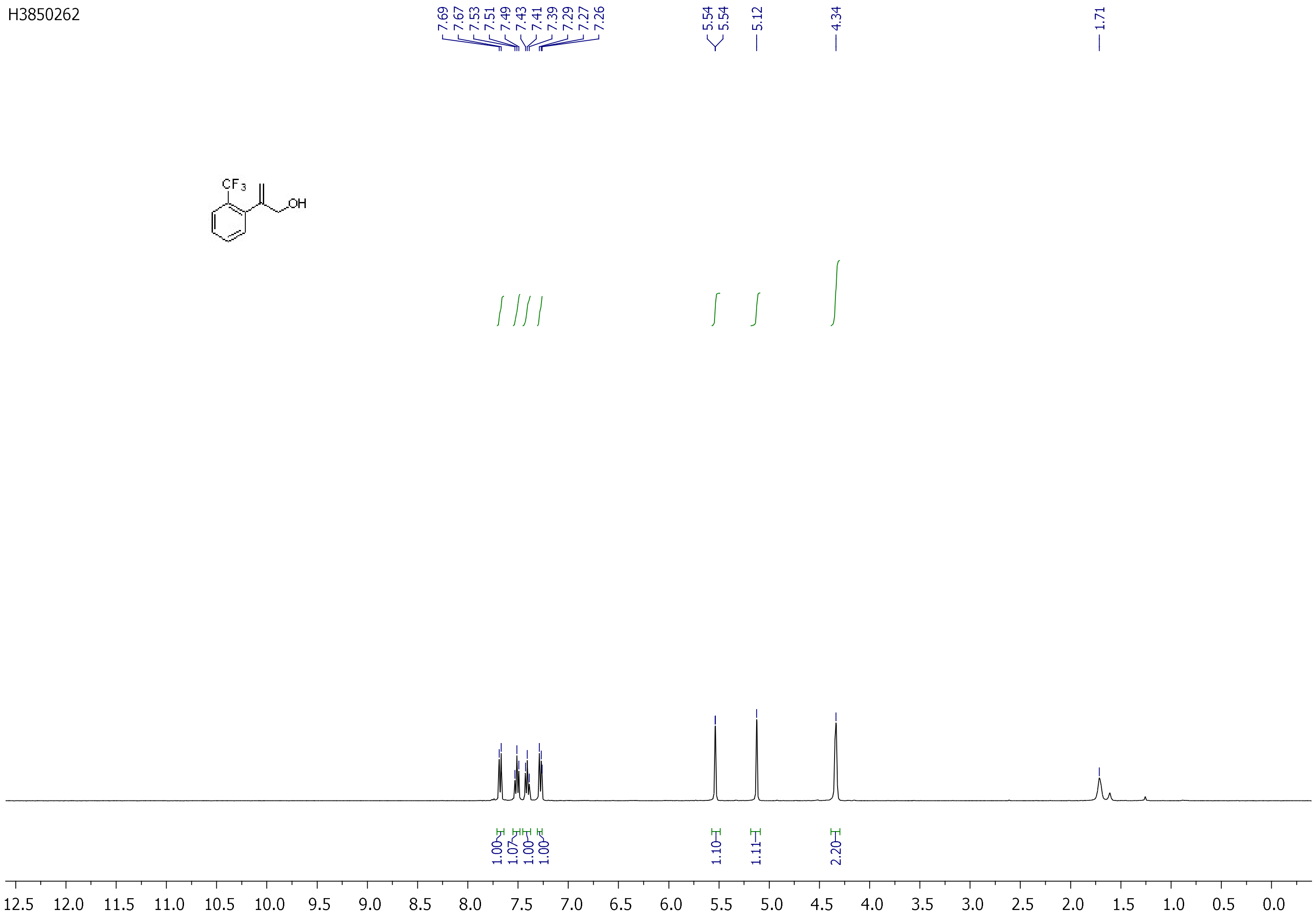

$^{13}C\{^{1}H\}$ NMR (126 MHz, $CDCl_3$)

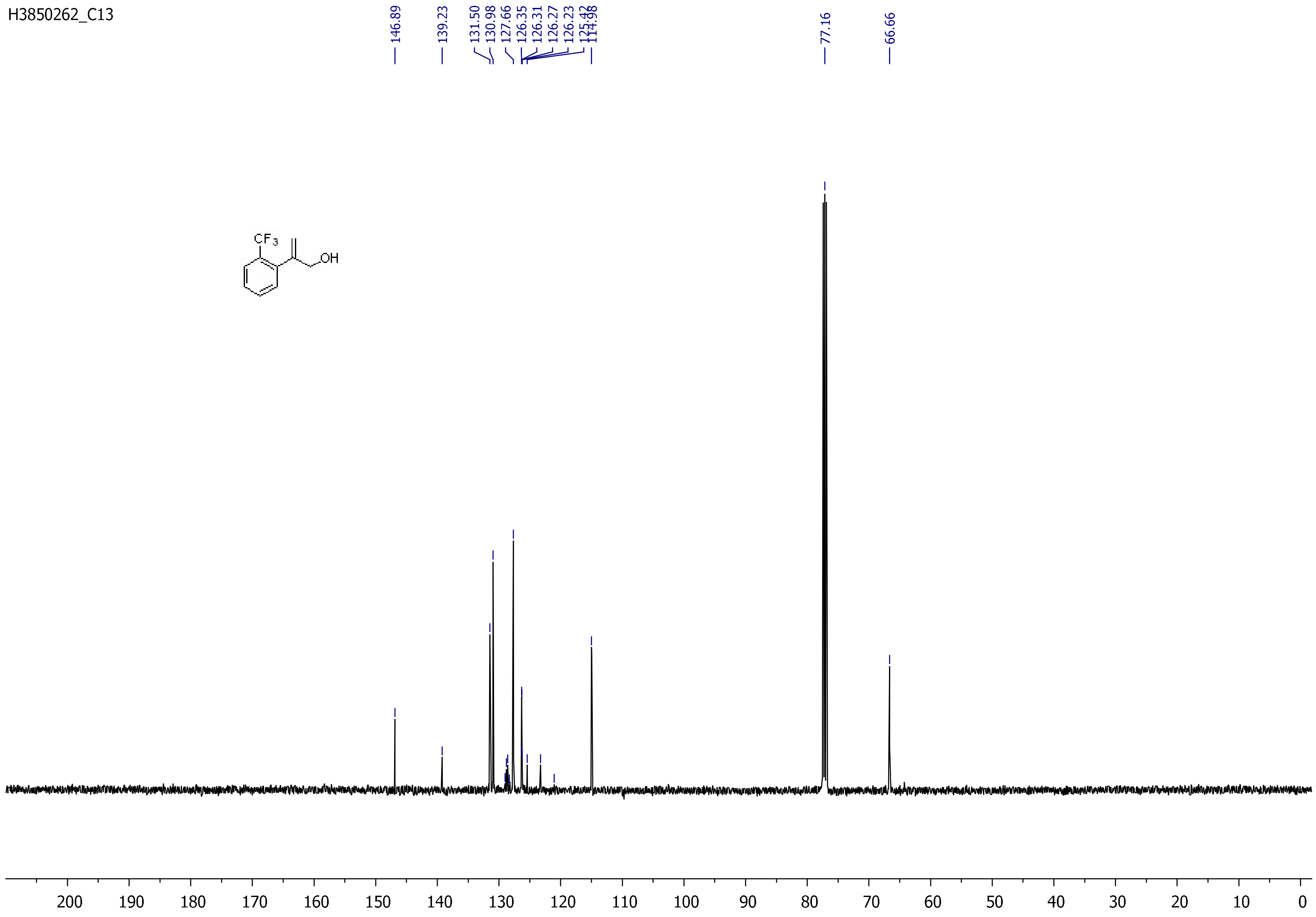

$^{19}F\{^{1}H\}$ NMR (376 MHz, $CDCl_3$)

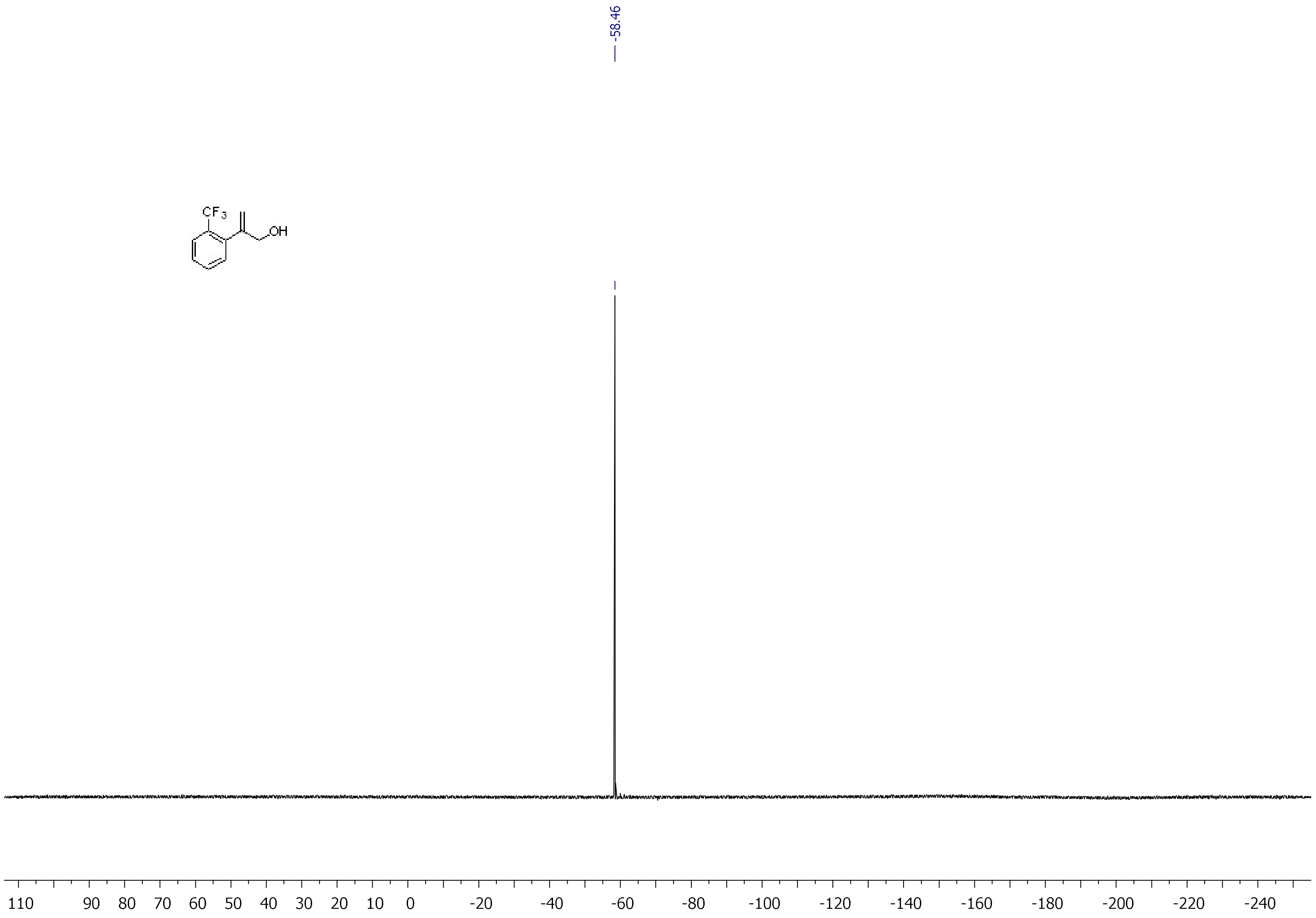

Compound (±)-17a, d.r. = 4:1

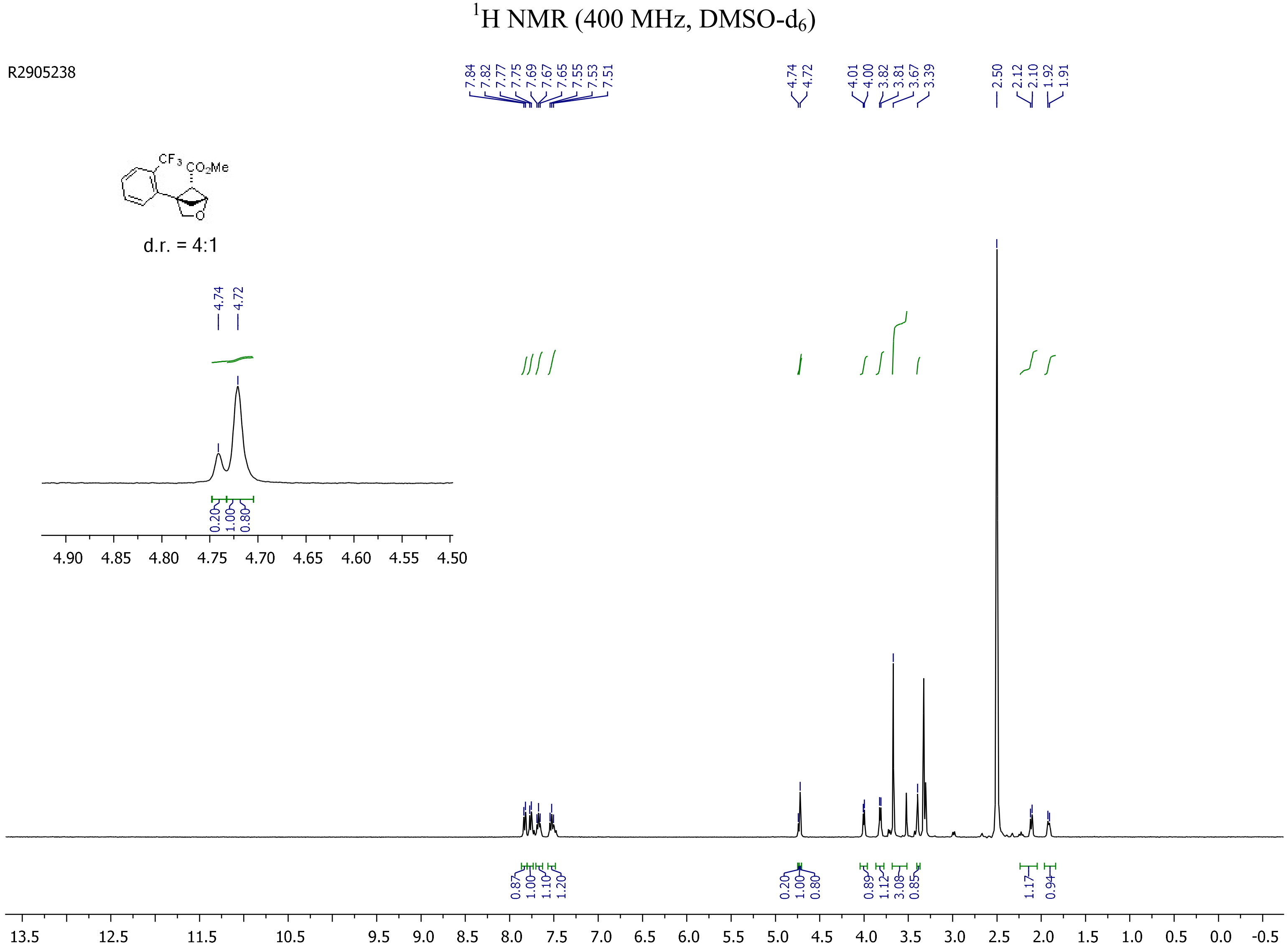

R2905238_C13

$^{13}C\{^1H\}$ NMR (126 MHz, $CDCl_3$)

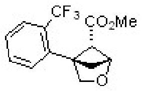

d.r. = 4:1

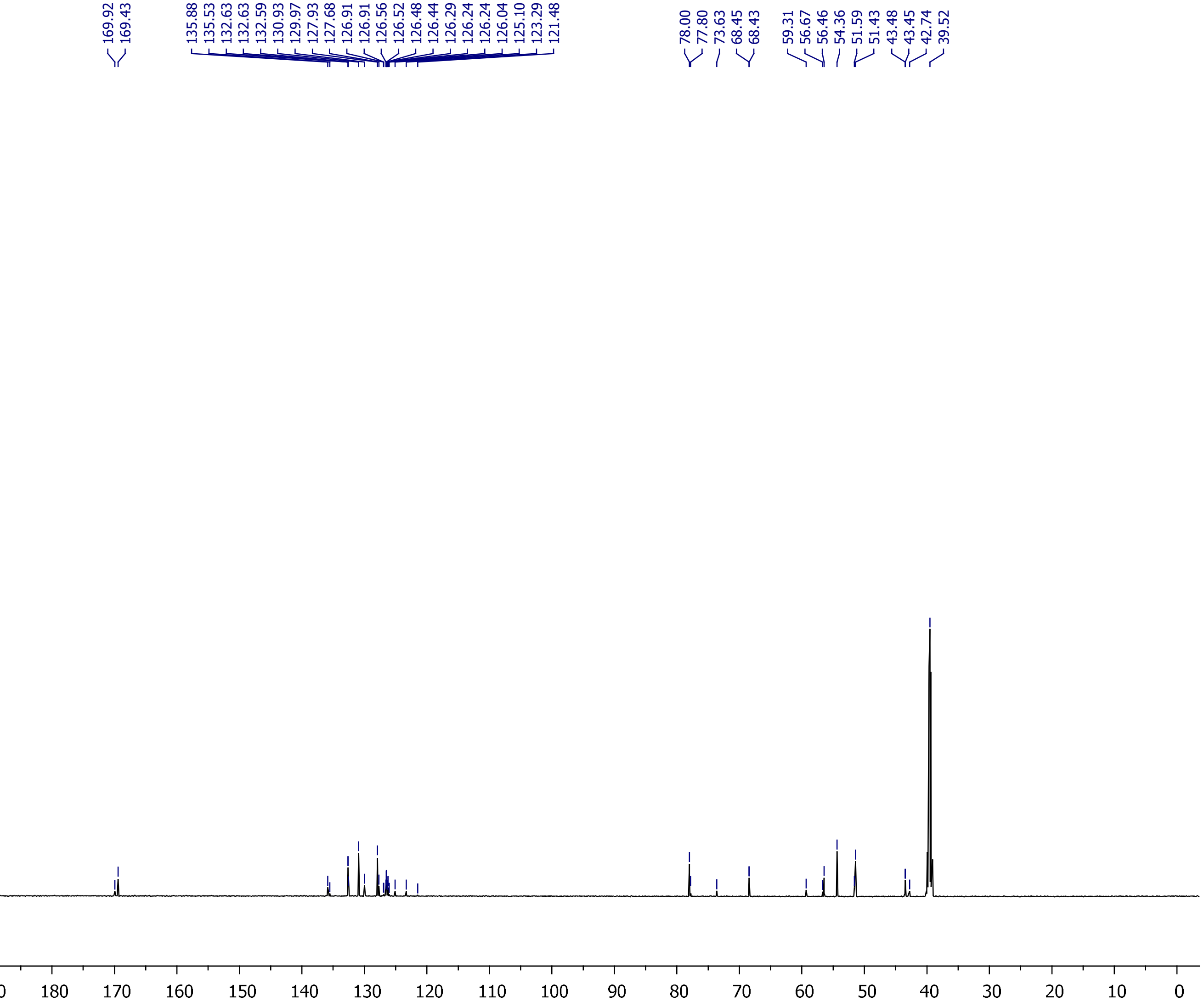

$^{19}F\{^1H\}$ NMR (376 MHz, DMSO-d_6)

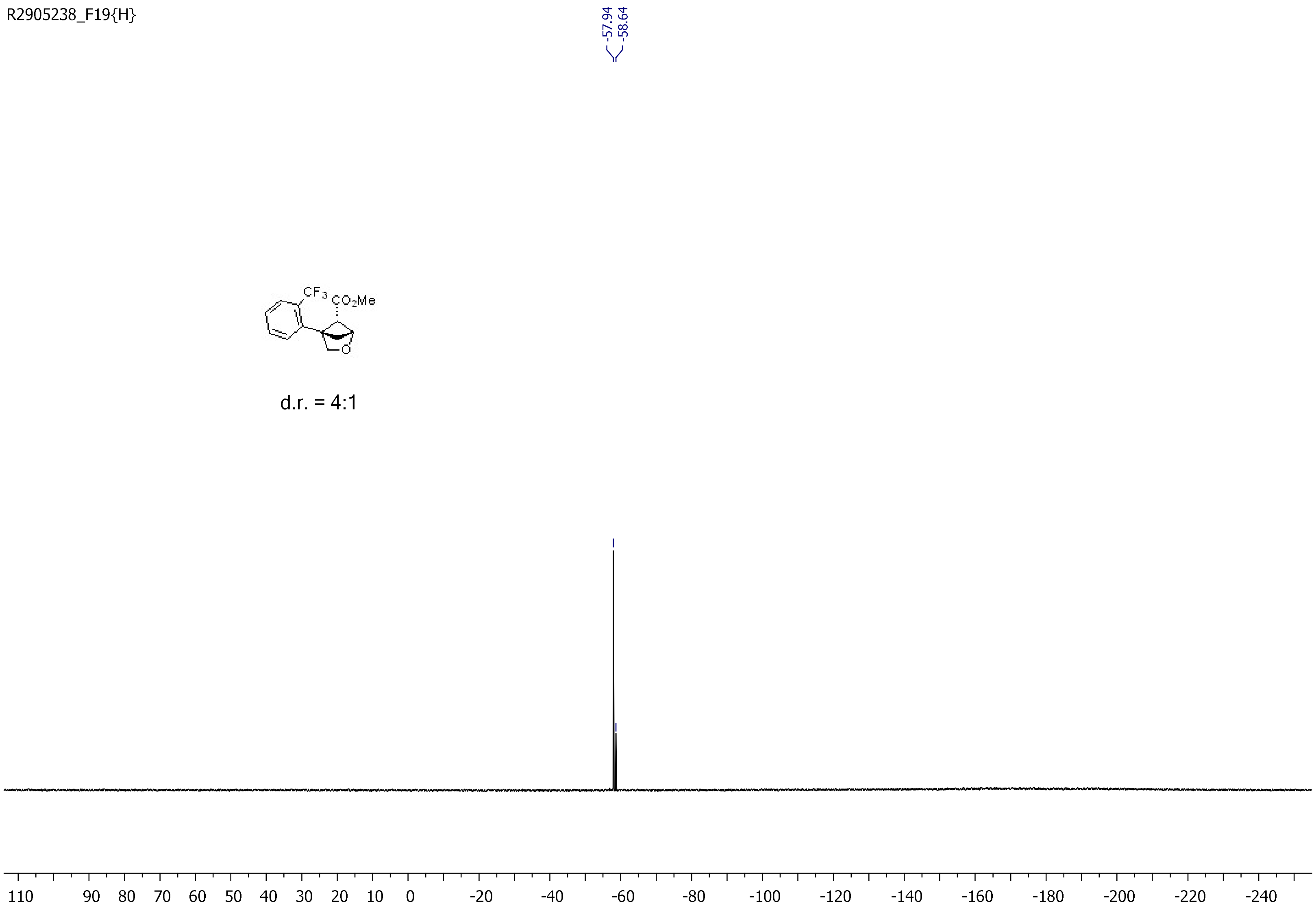

Compound (±)-17b, d.r. = 3:1

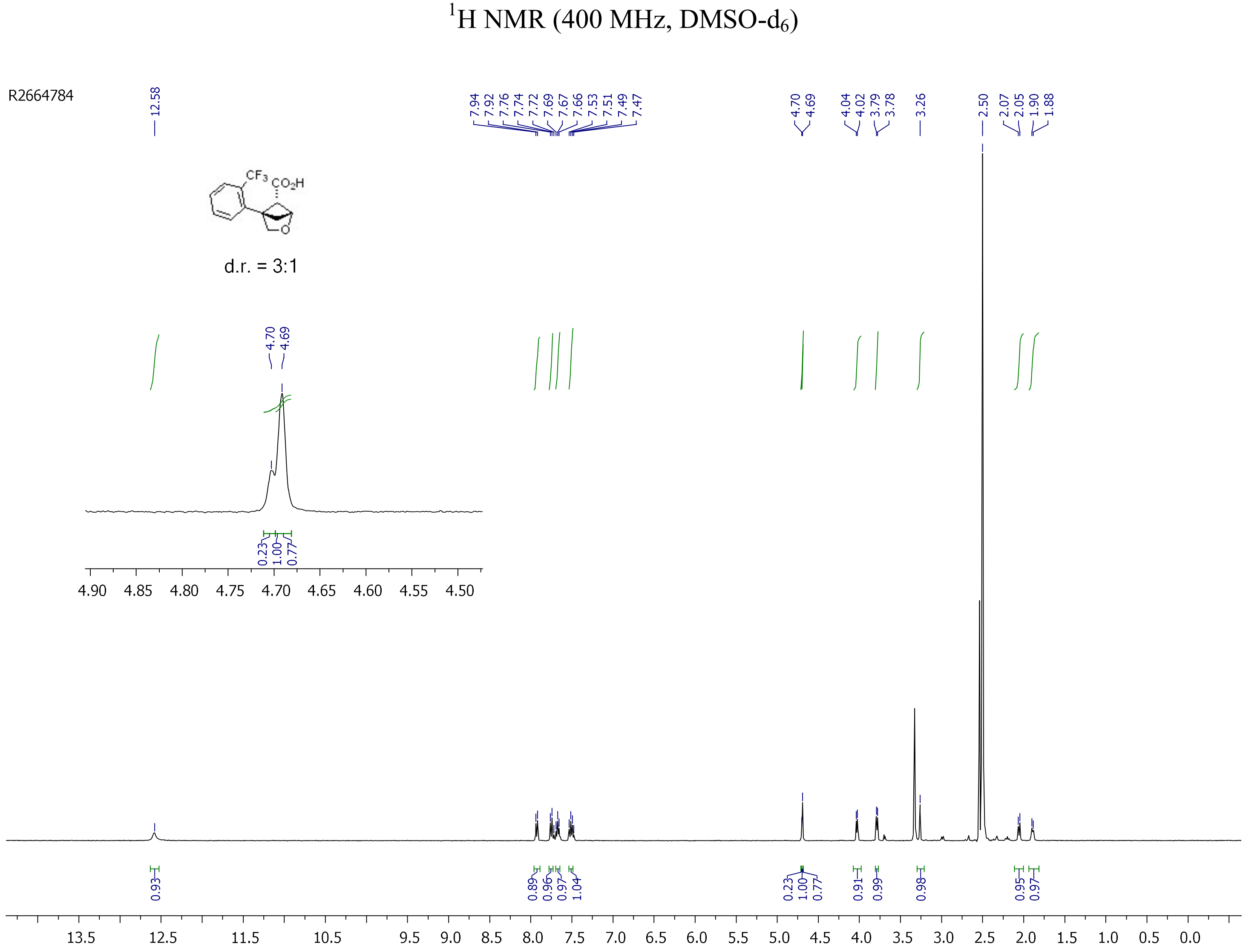

$^{13}C\{^{1}H\}$ NMR (126 MHz, $CDCl_3$)

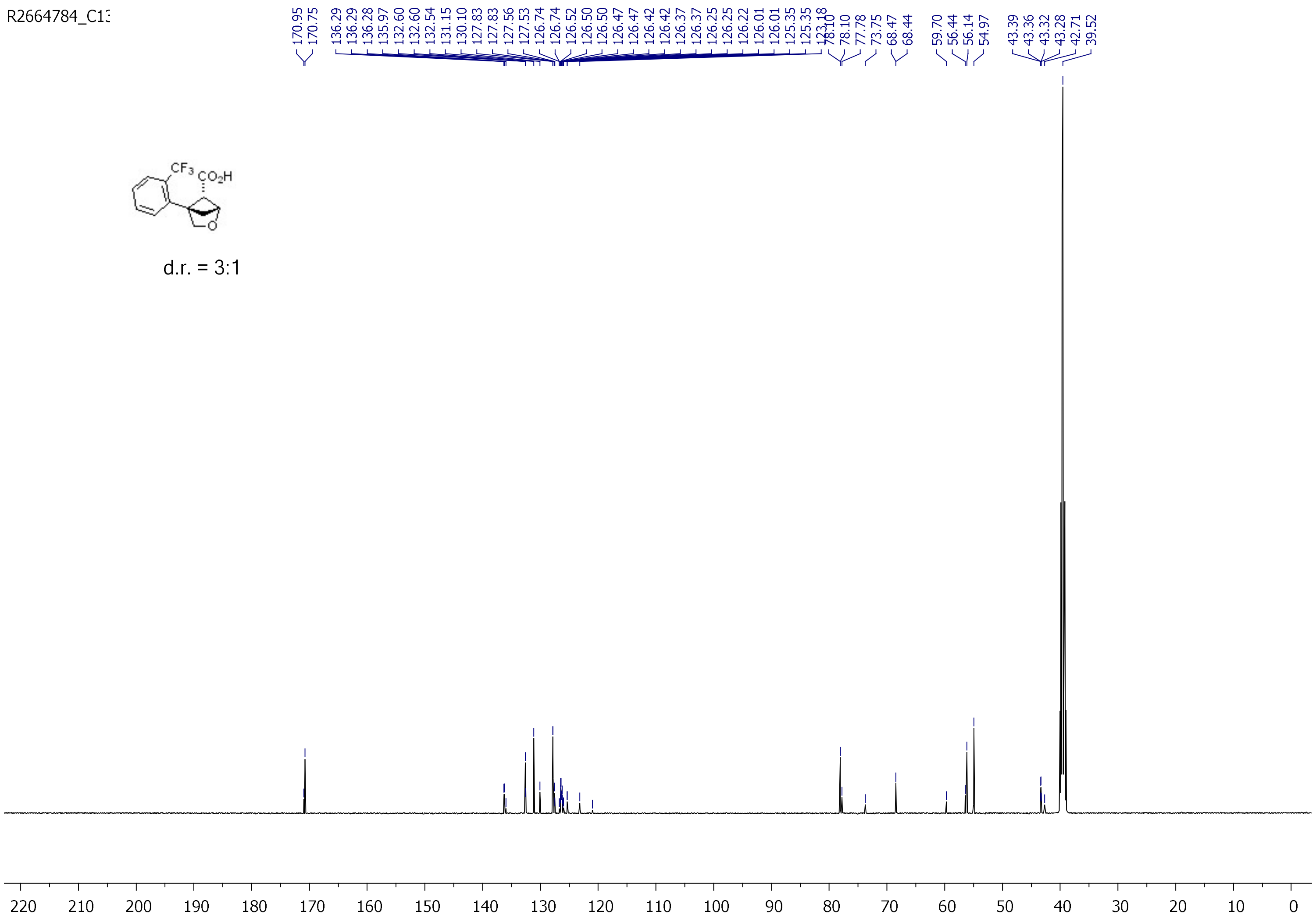

$^{19}F\{^{1}H\}$ NMR (376 MHz, DMSO-d_6)

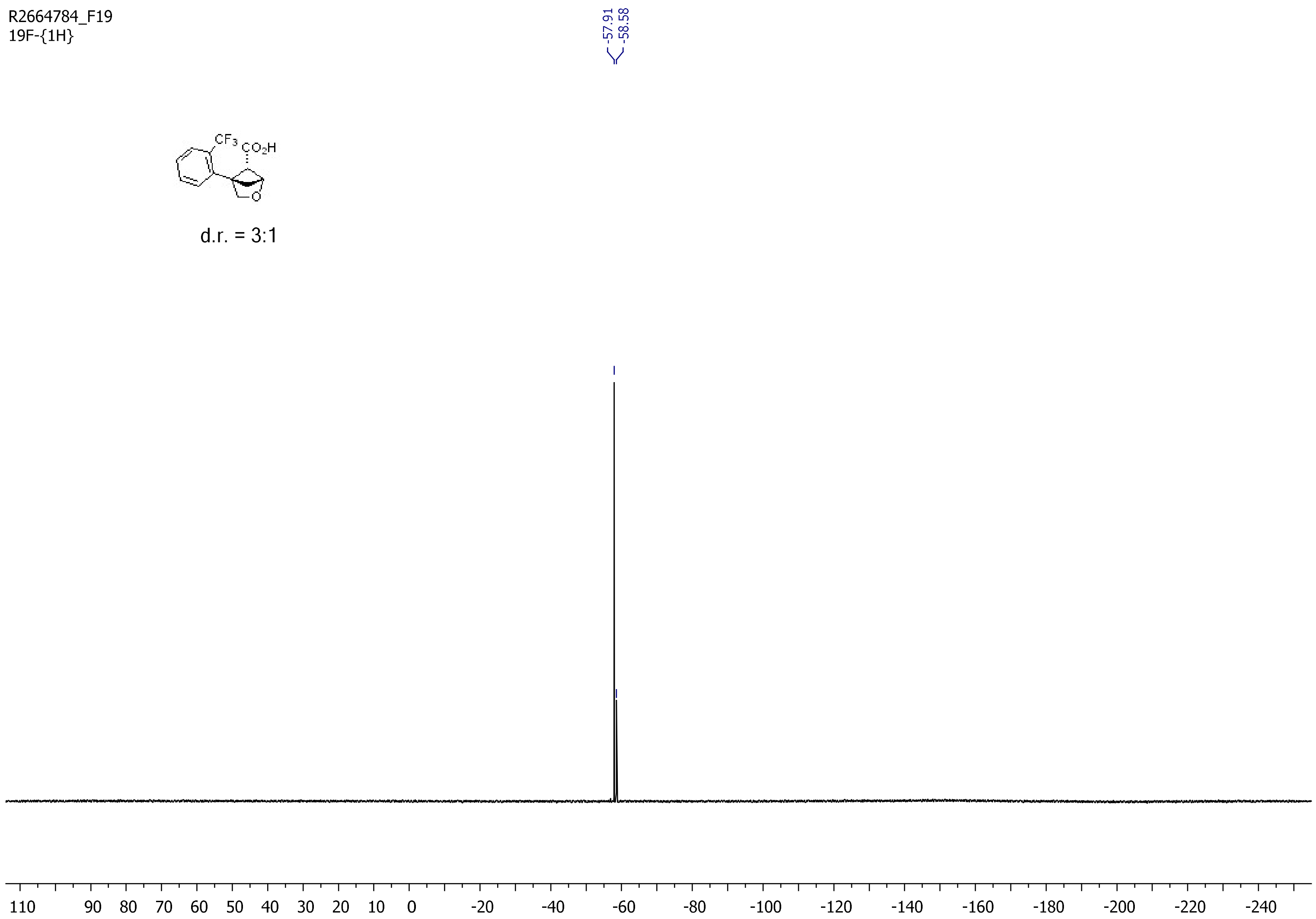

2-(3-(Trifluoromethyl)phenyl)prop-2-en-1-ol

^{1}H NMR (400 MHz, $CDCl_3$)

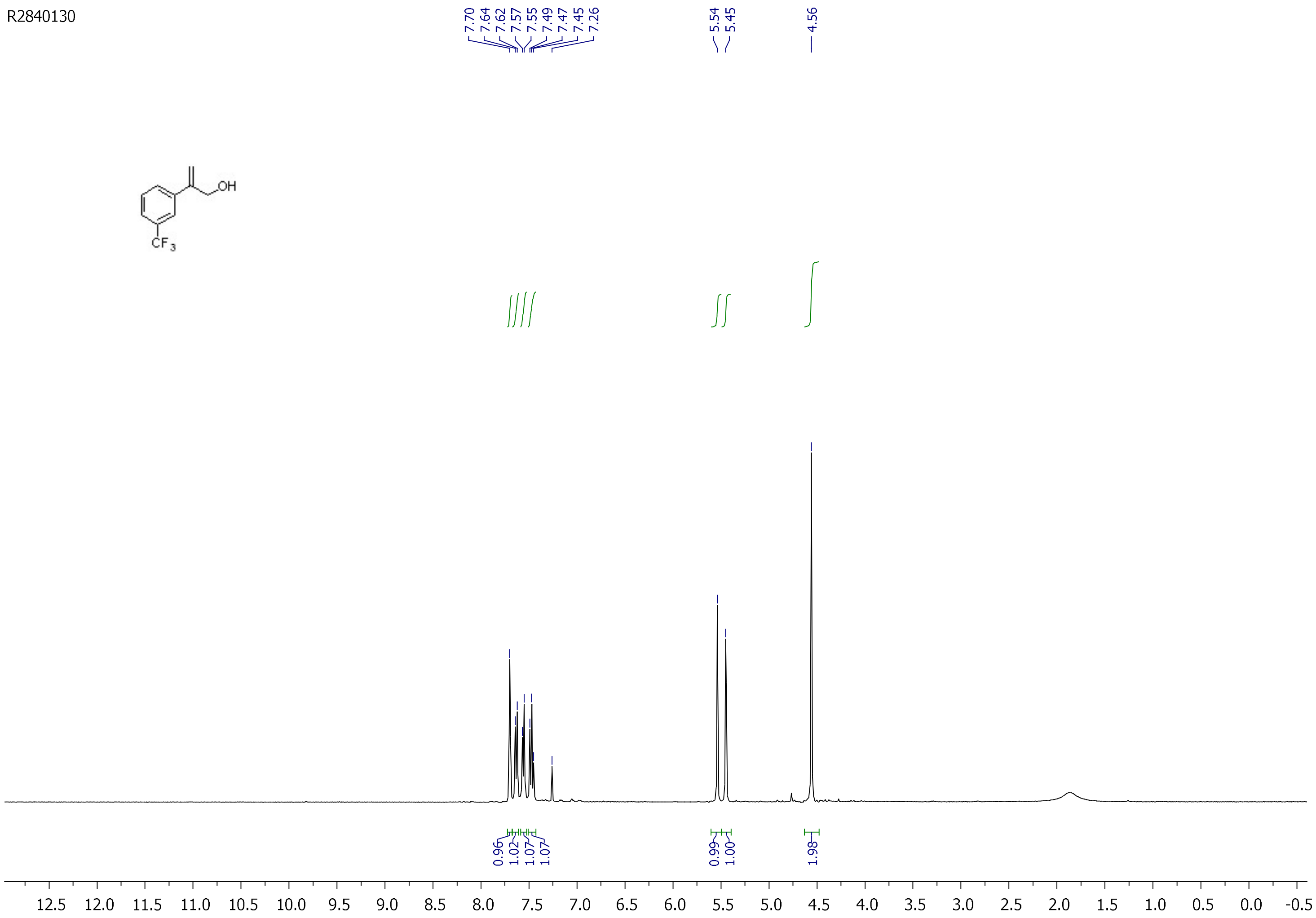

$^{13}C\{^{1}H\}$ NMR (151 MHz, $CDCl_3$)

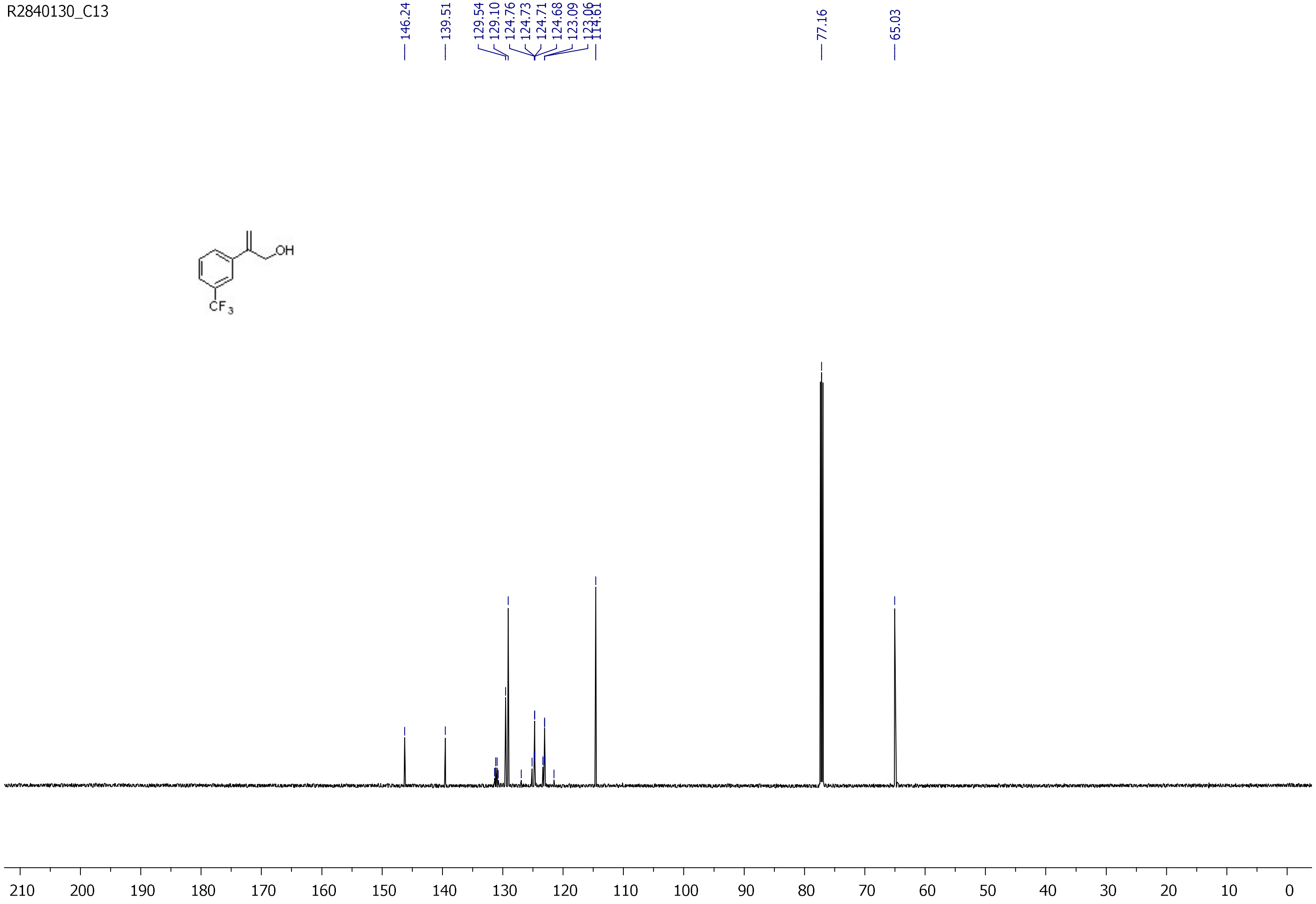

$^{19}F\{^1H\}$ NMR (376 MHz, $CDCl_3$)

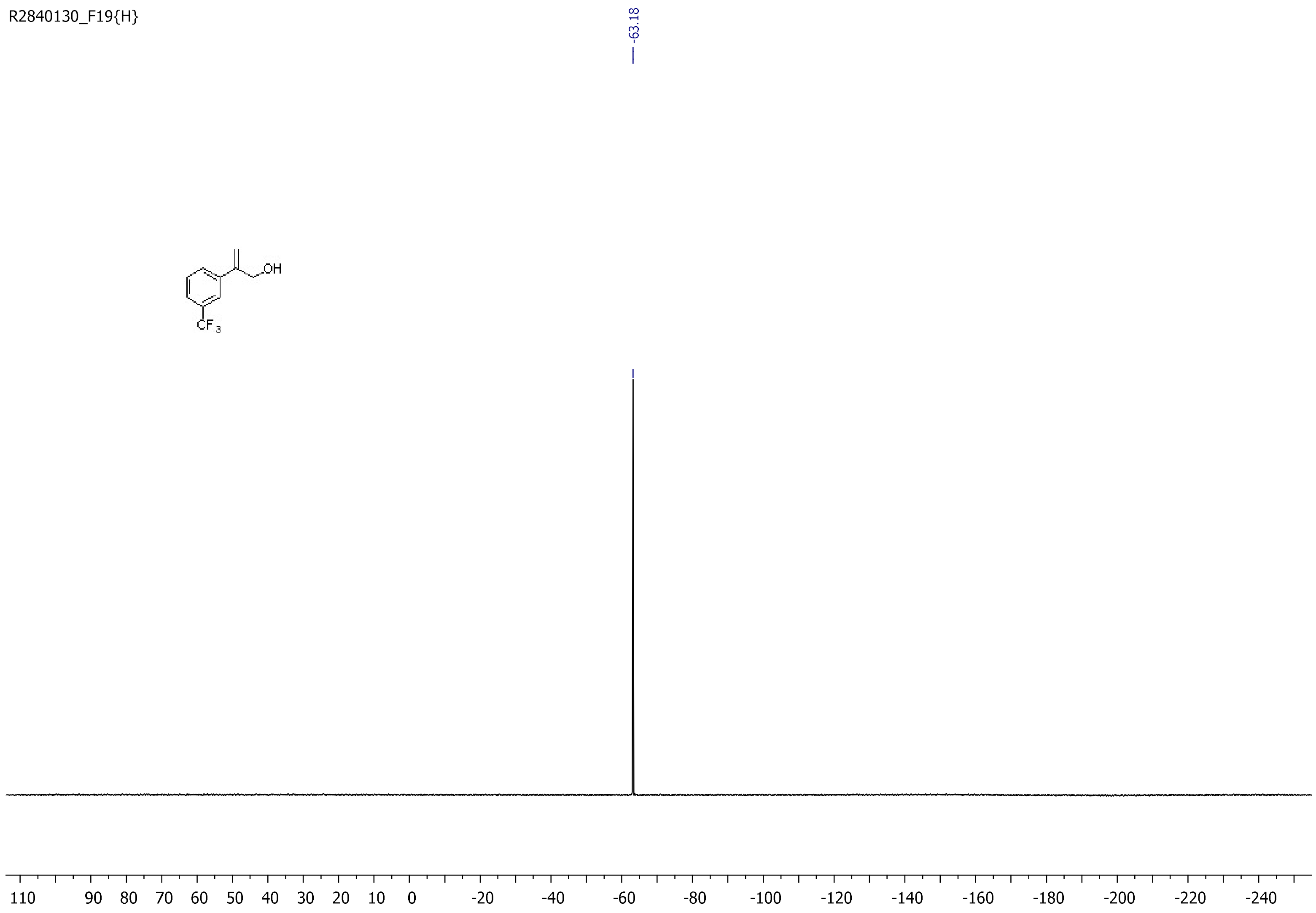

Compound (±)-18a, d.r. = 3:1

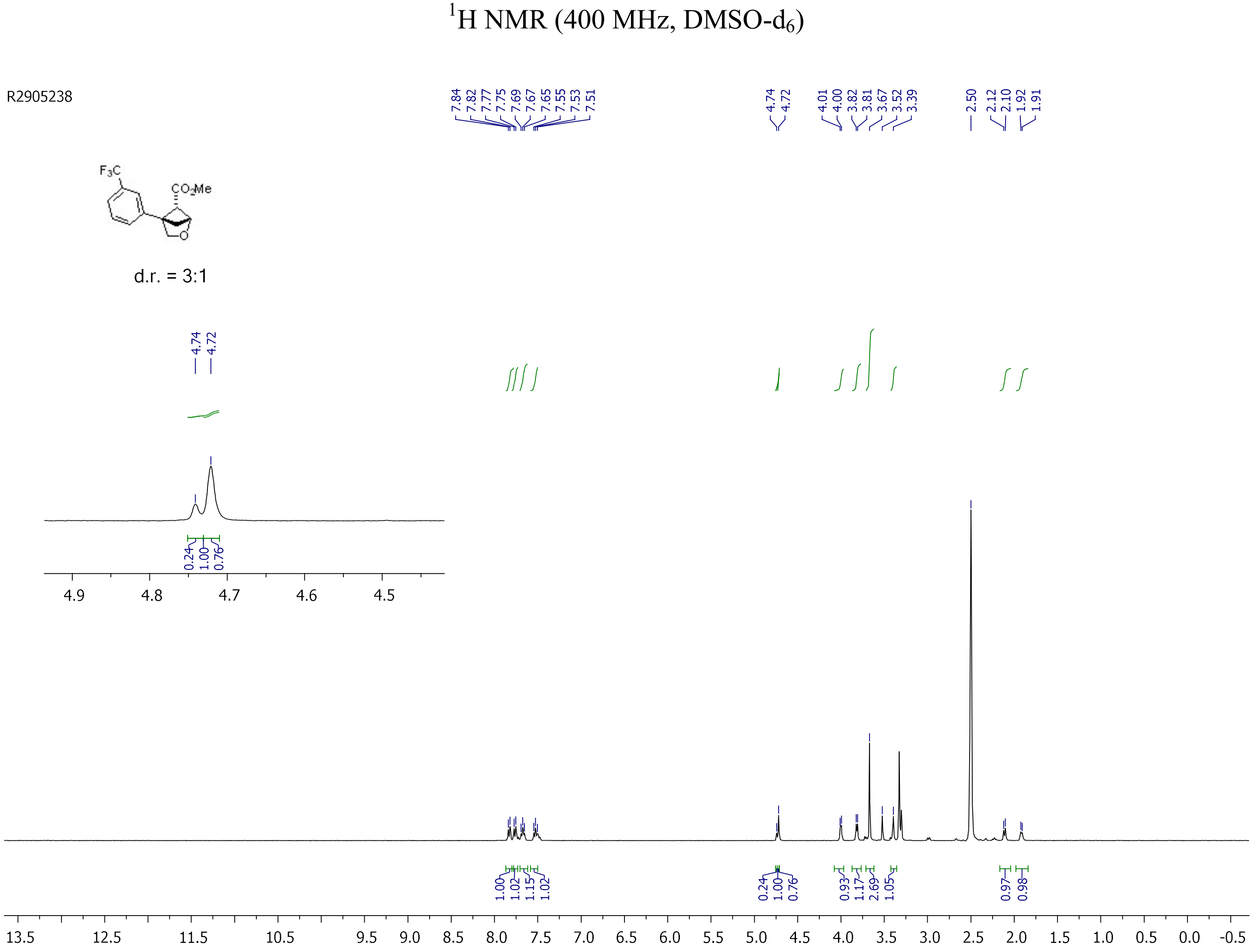

$^{13}C\{^1H\}$ NMR (126 MHz, $CDCl_3$)

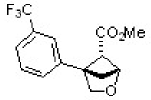

d.r. = 3:1

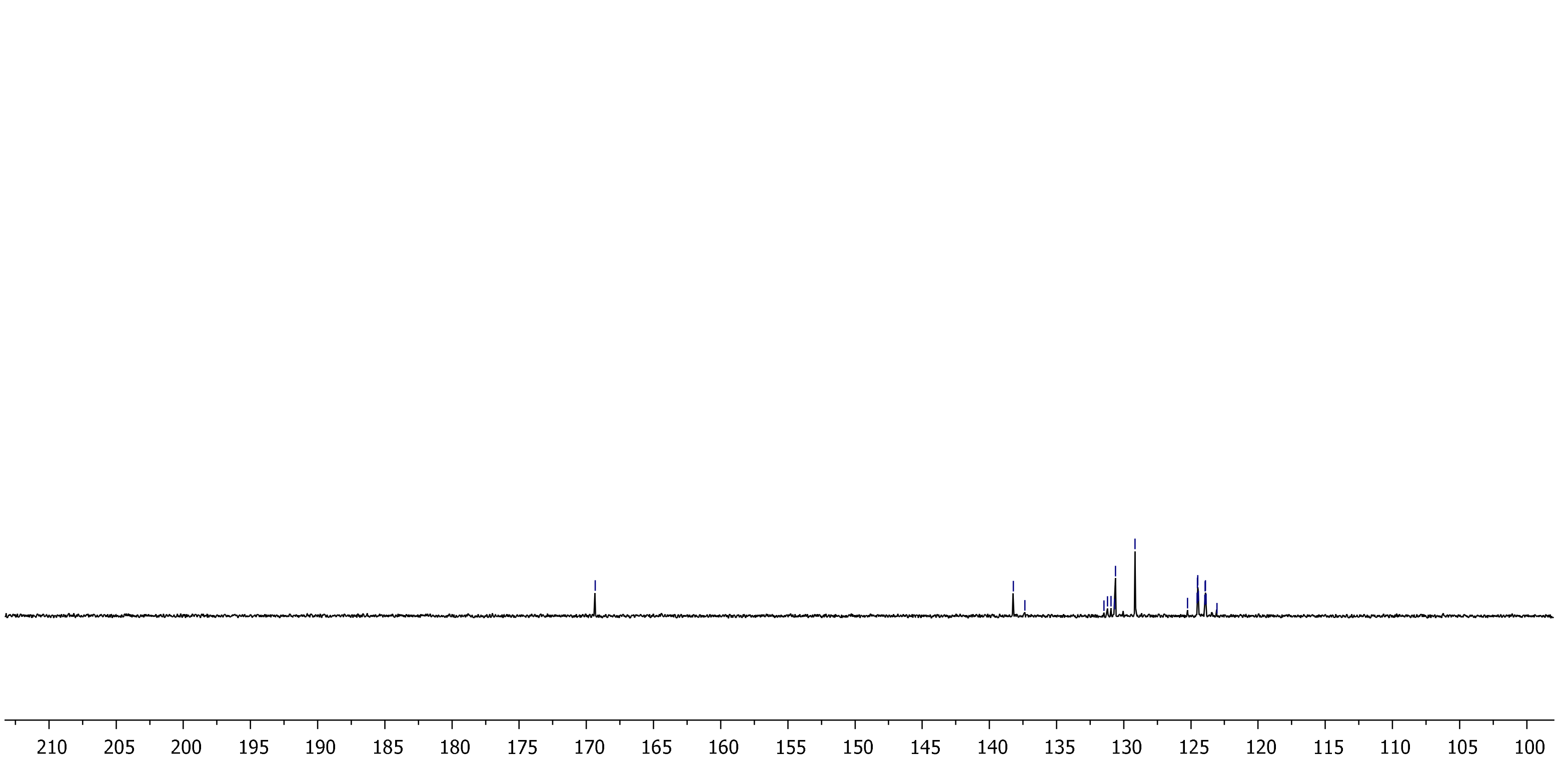

$^{19}F\{^{1}H\}$ NMR (376 MHz, DMSO-d_6)

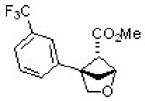

d.r. = 3:1

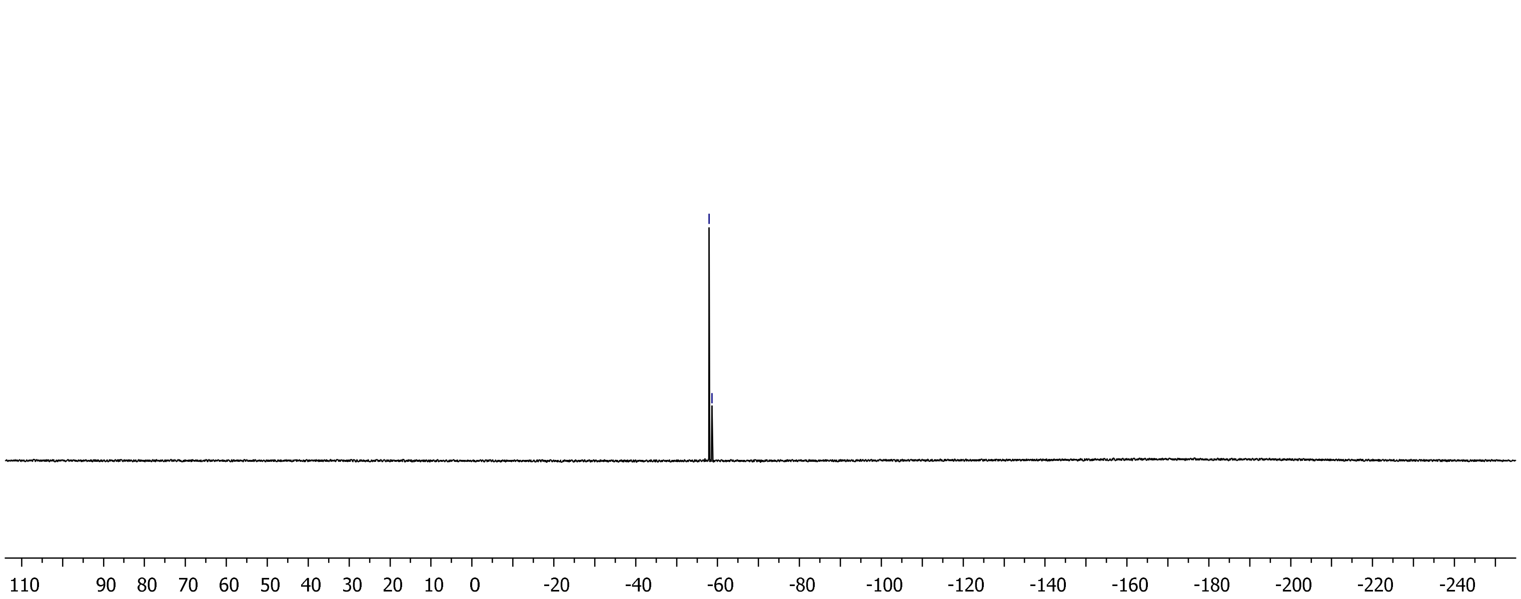

Compound (±)-18b

^{1}H NMR (400 MHz, DMSO-d$_6$)

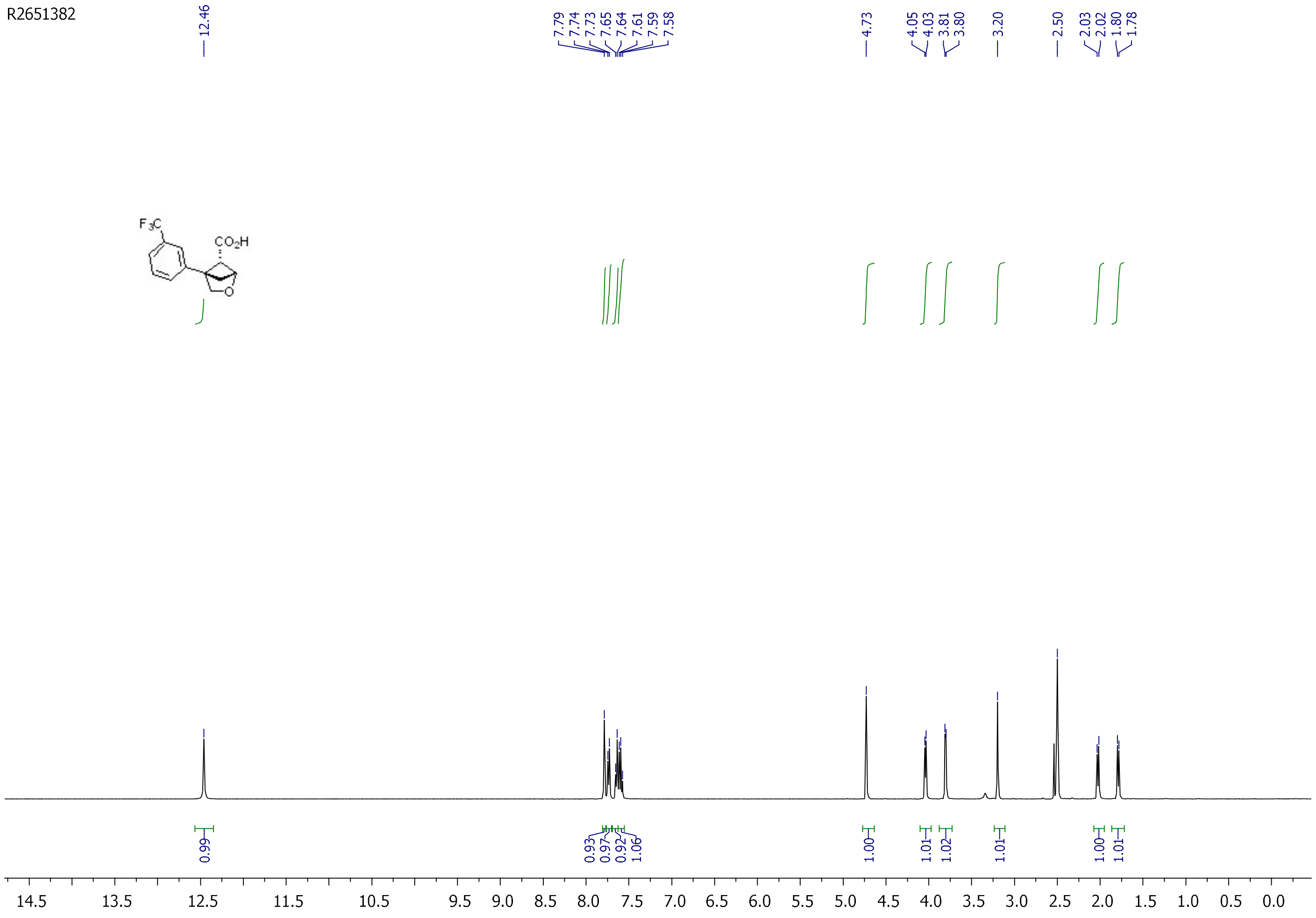

$^{13}C\{^{1}H\}$ NMR (126 MHz, DMSO-d_6)

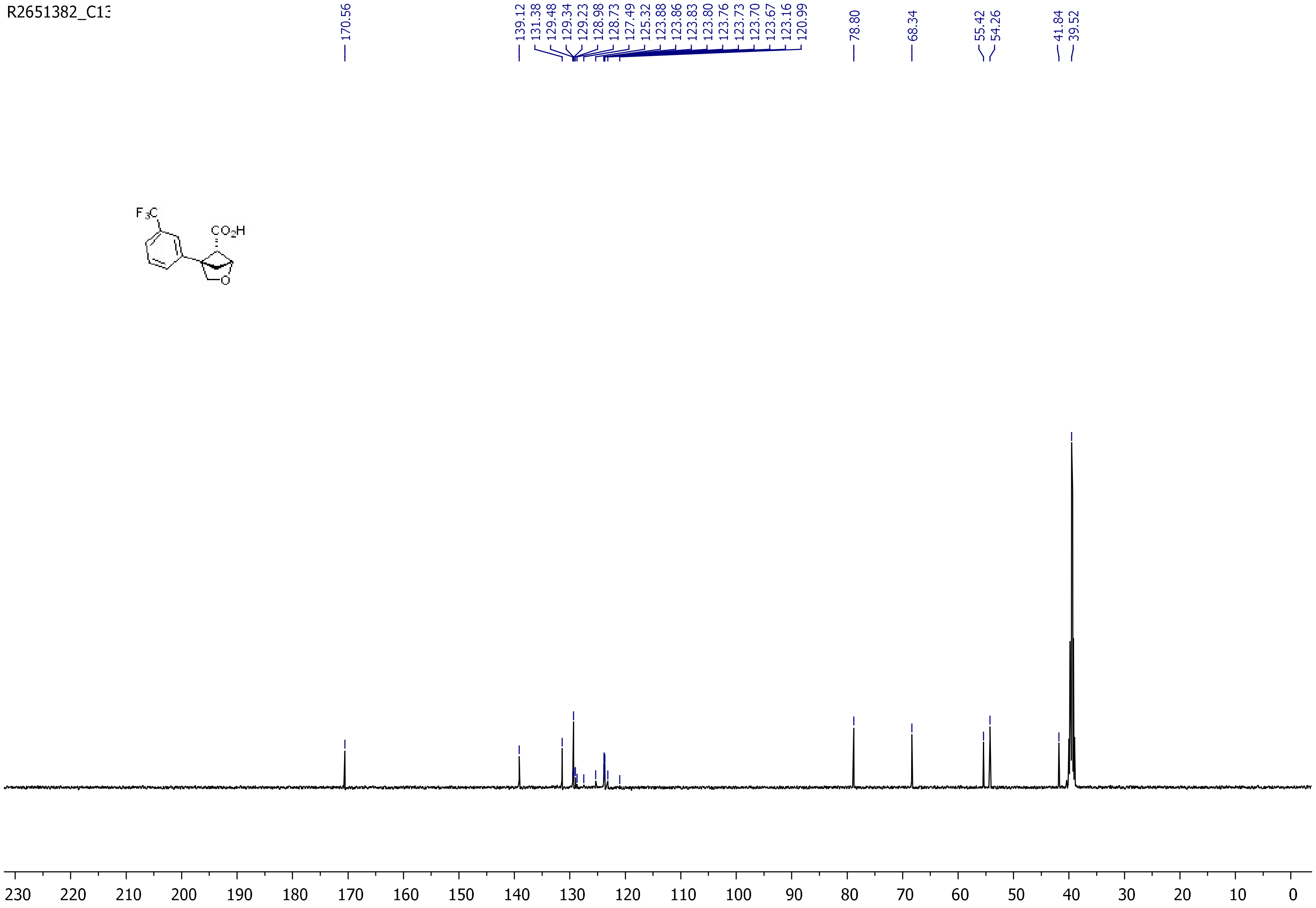

$^{19}F\{^1H\}$ NMR (376 MHz, DMSO-d$_6$)

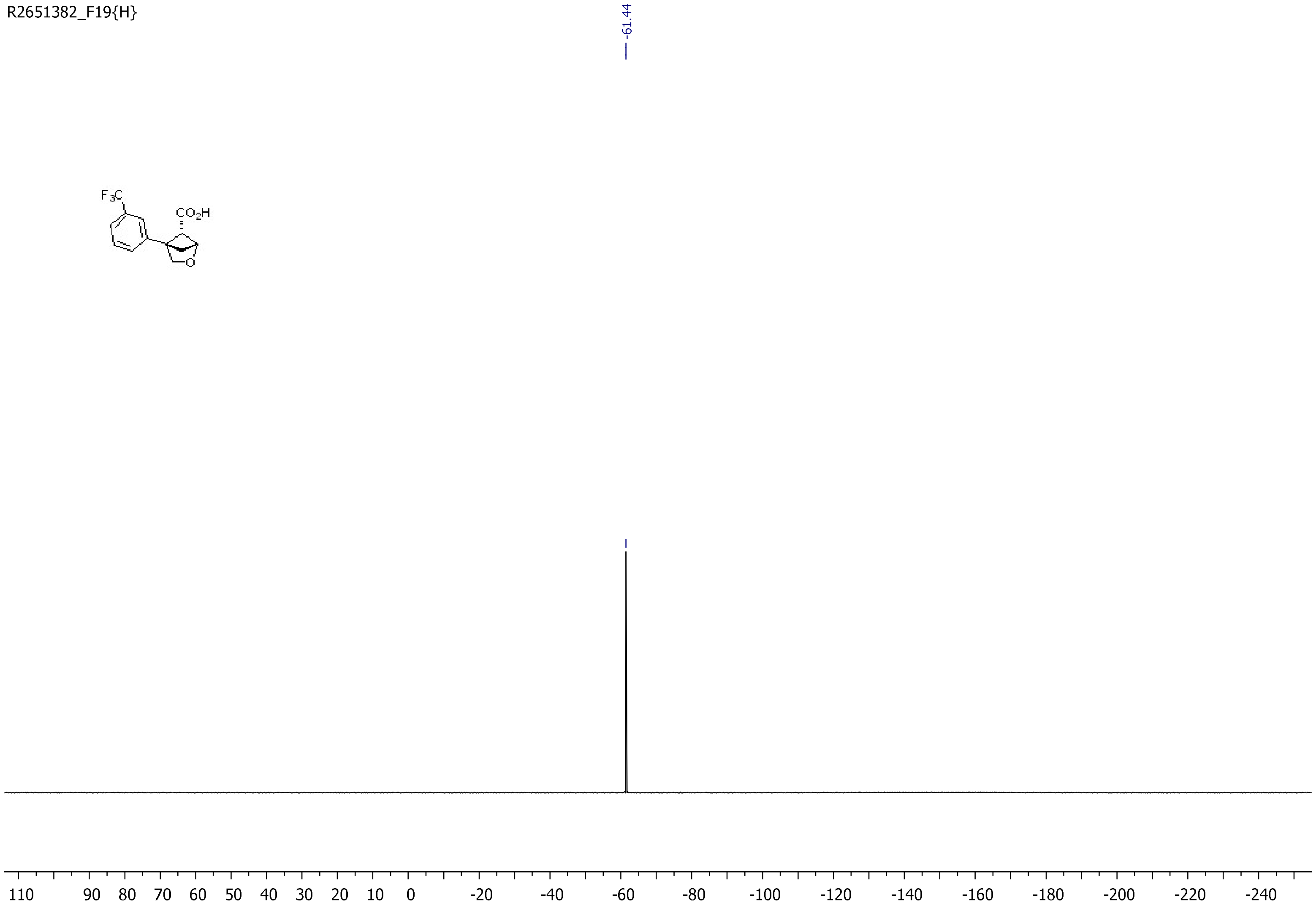

2-(4-(Trifluoromethyl)phenyl)prop-2-en-1-ol (ca. 90% purity)

^{1}H NMR (400 MHz, $CDCl_3$)

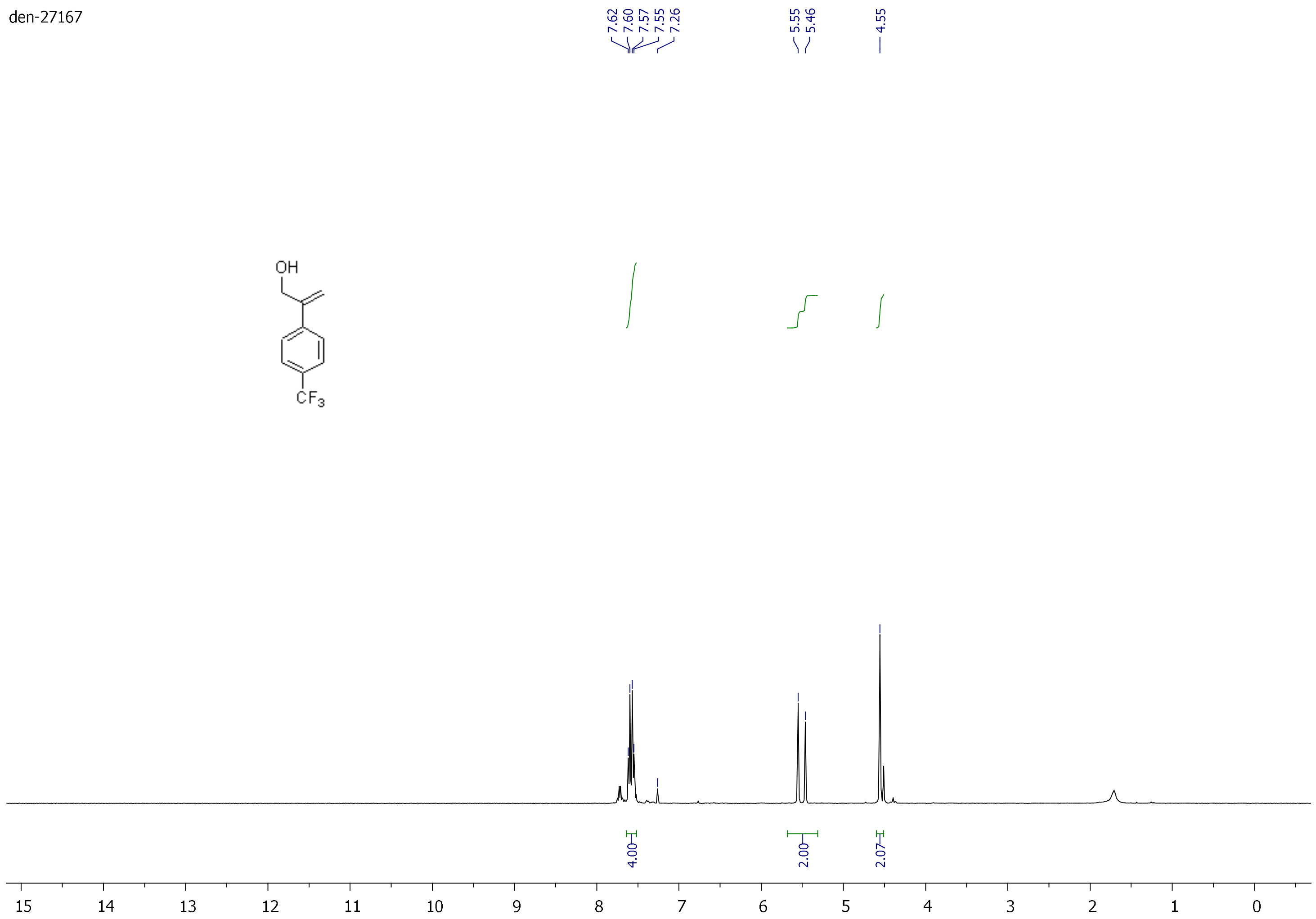

$^{13}C\{^1H\}$ NMR (126 MHz, $CDCl_3$)

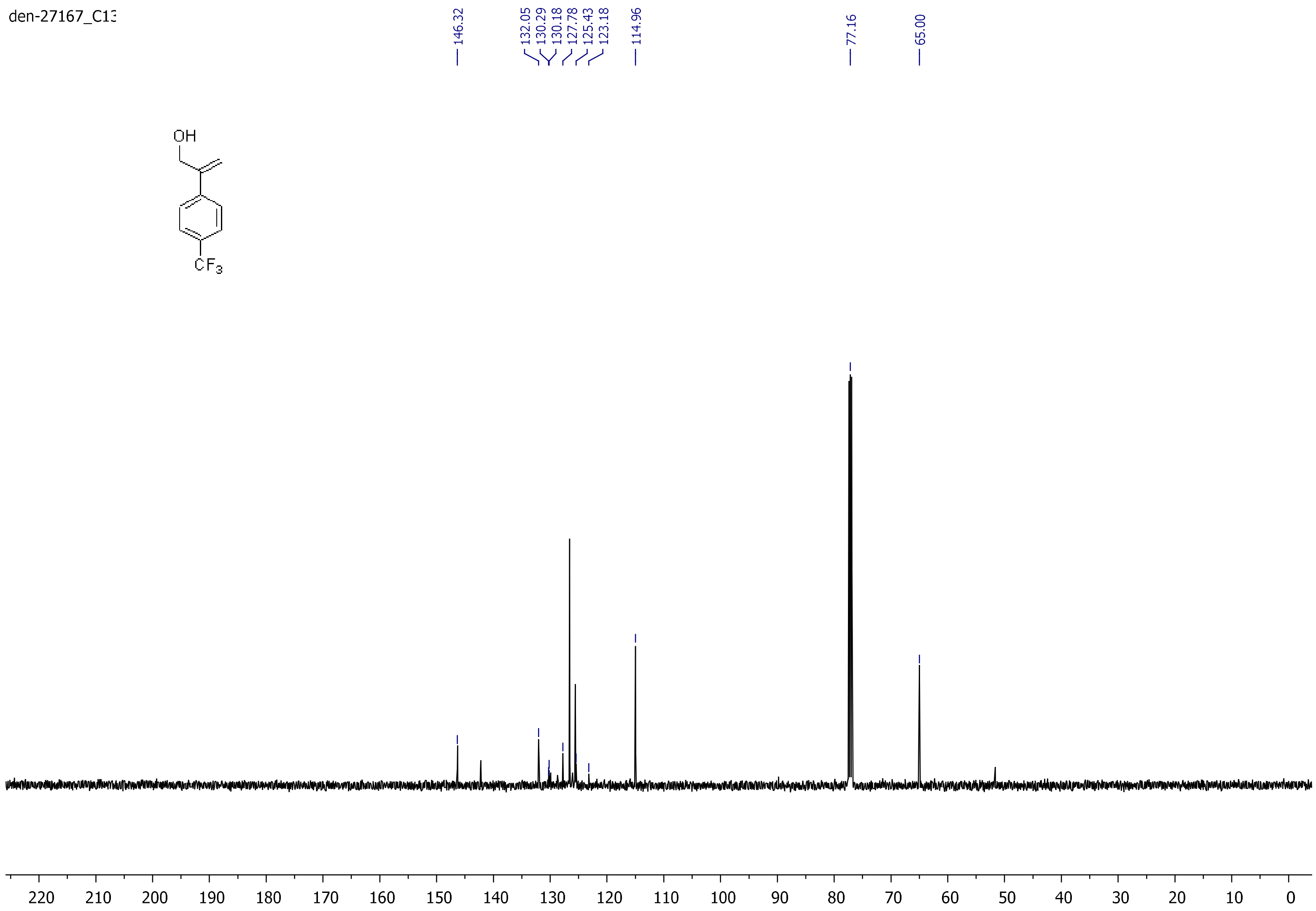

$^{19}F\{^{1}H\}$ NMR (376 MHz, $CDCl_3$)

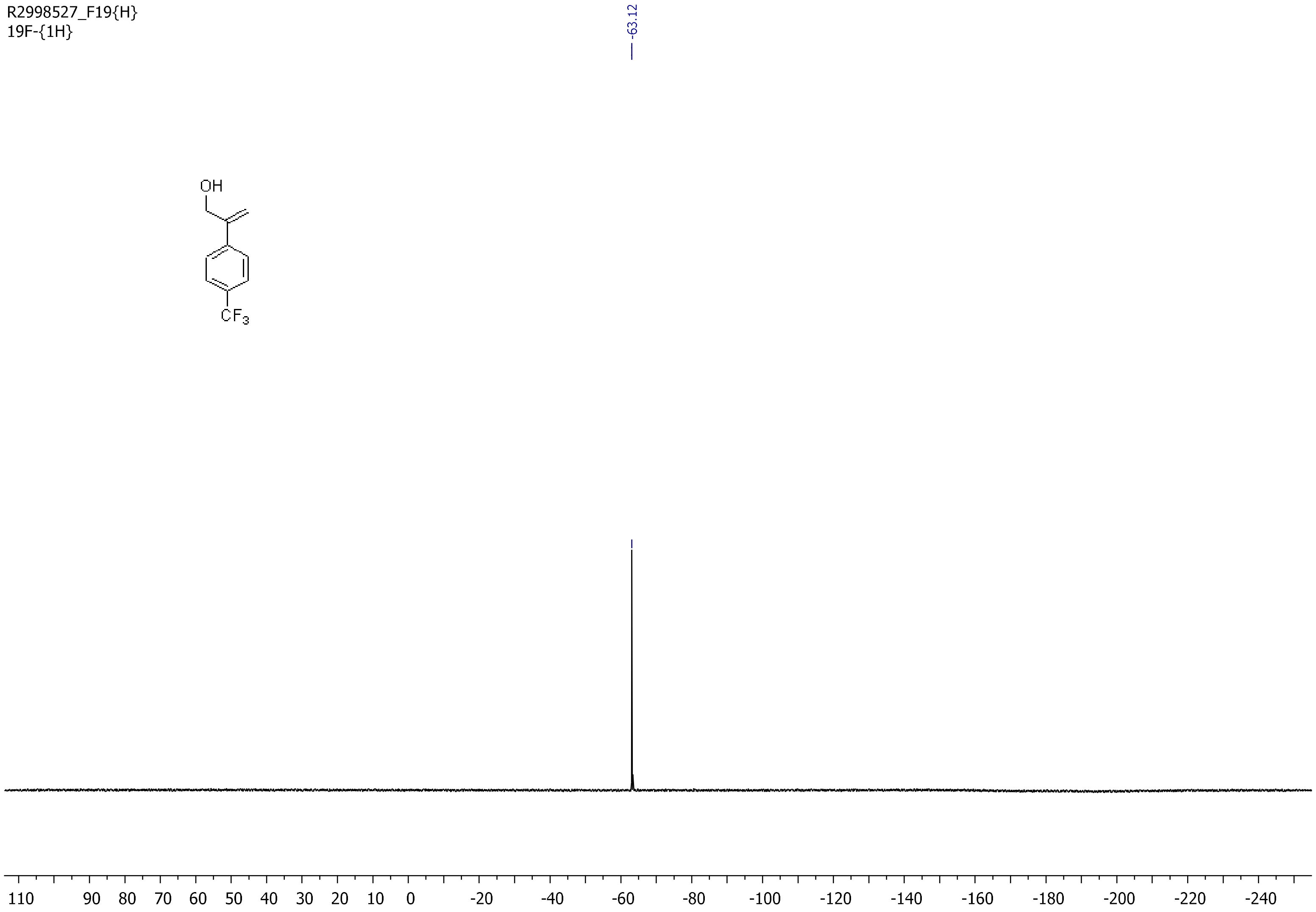

Compound (±)-19a, d.r. = 4:1

^{1}H NMR (400 MHz, DMSO-d_6)

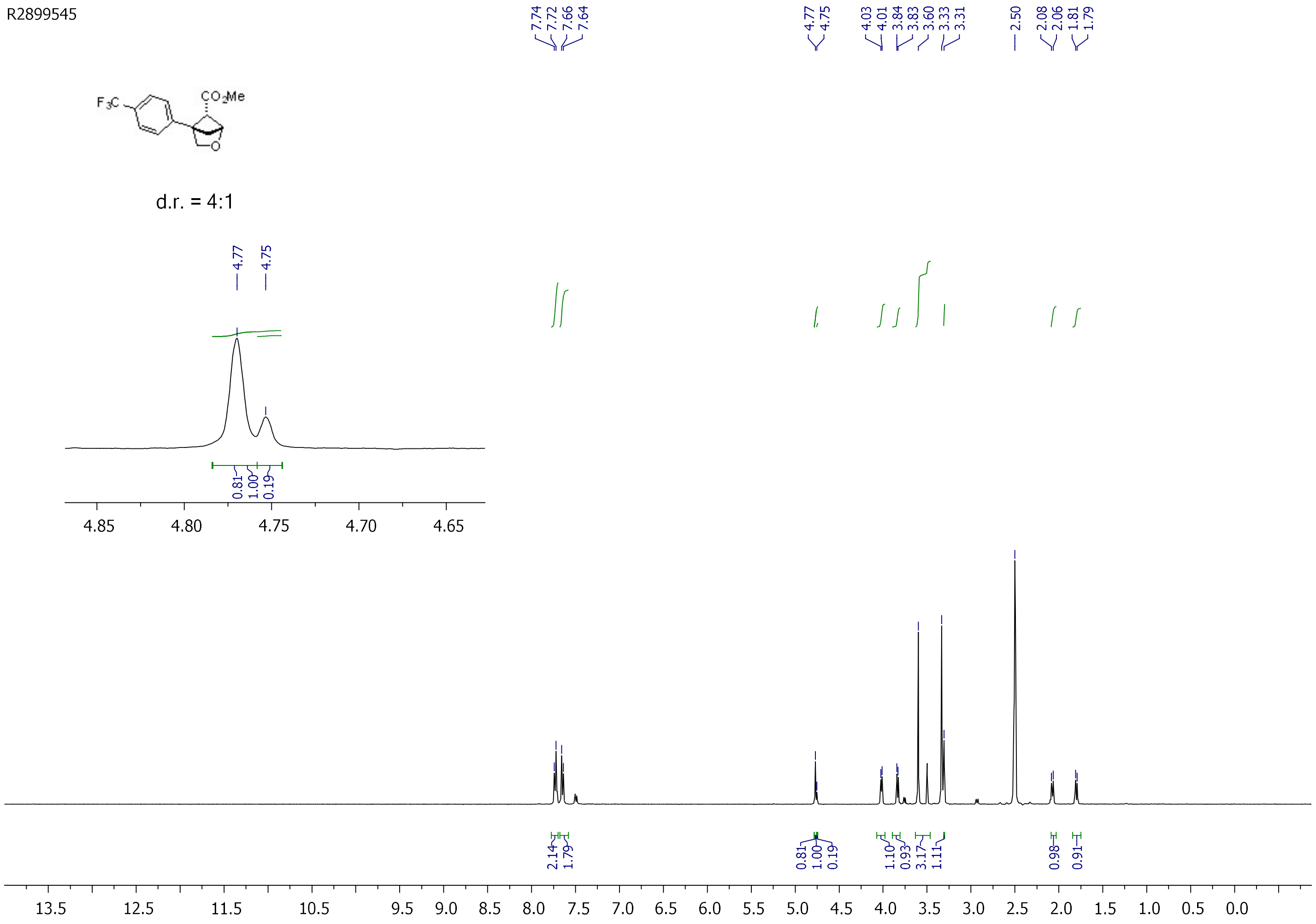

$^{13}C\{^1H\}$ NMR (151 MHz, $CDCl_3$)

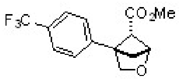

d.r. = 4:1

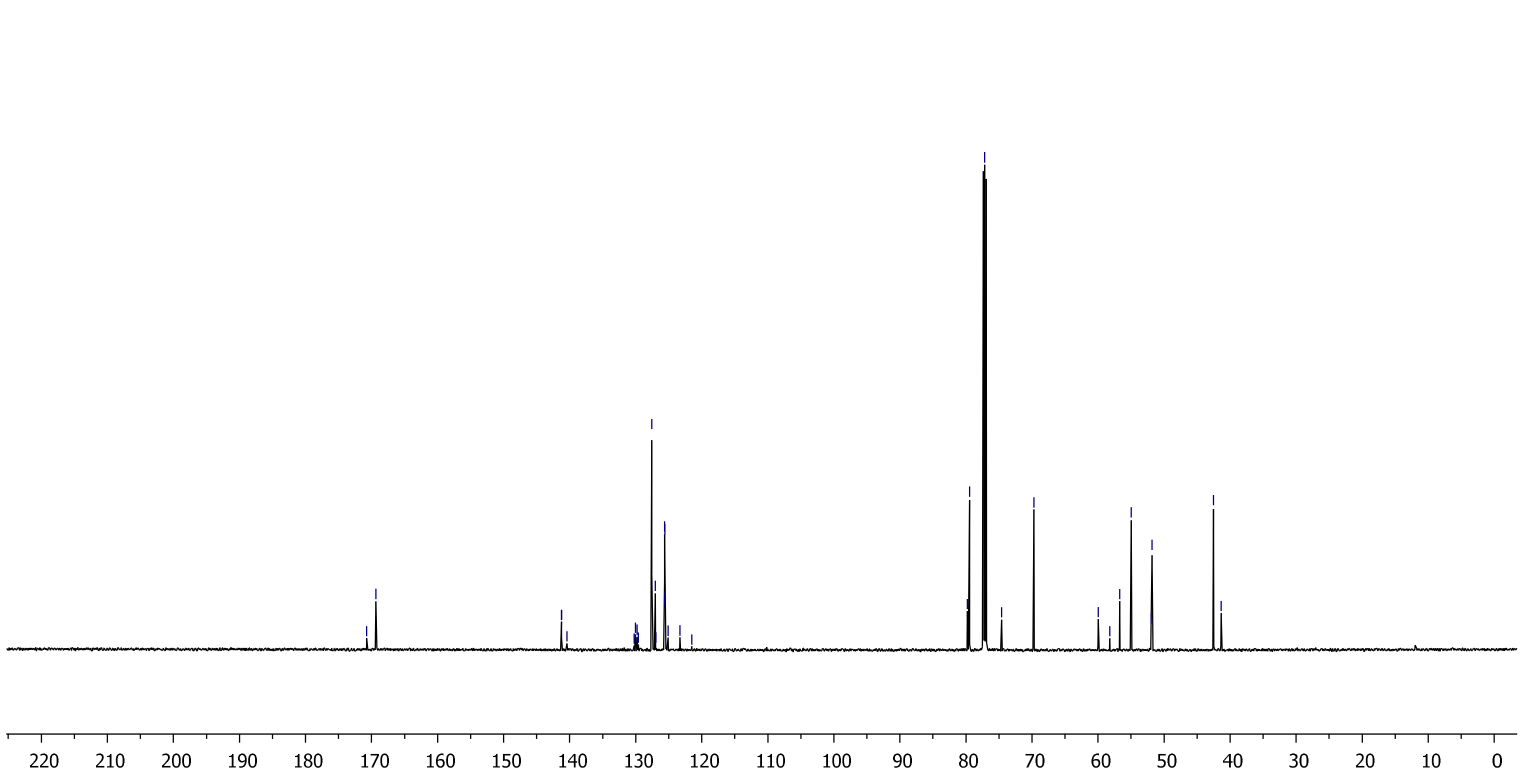

$^{19}F\{^1H\}$ NMR (376 MHz, DMSO-d_6)

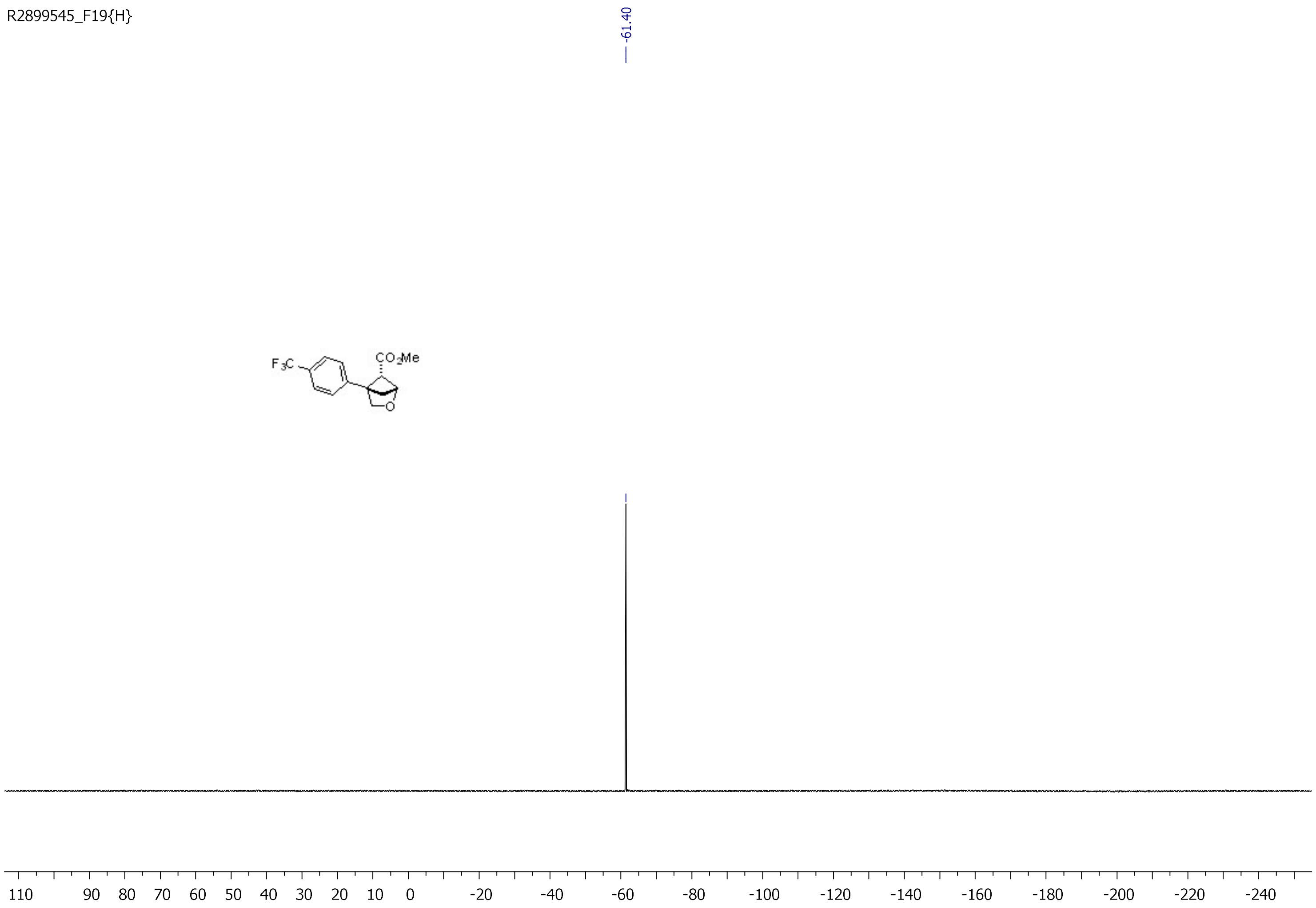

Compound (±)-19b, d.r. = 4:1

^{1}H NMR (400 MHz, DMSO-d_6)

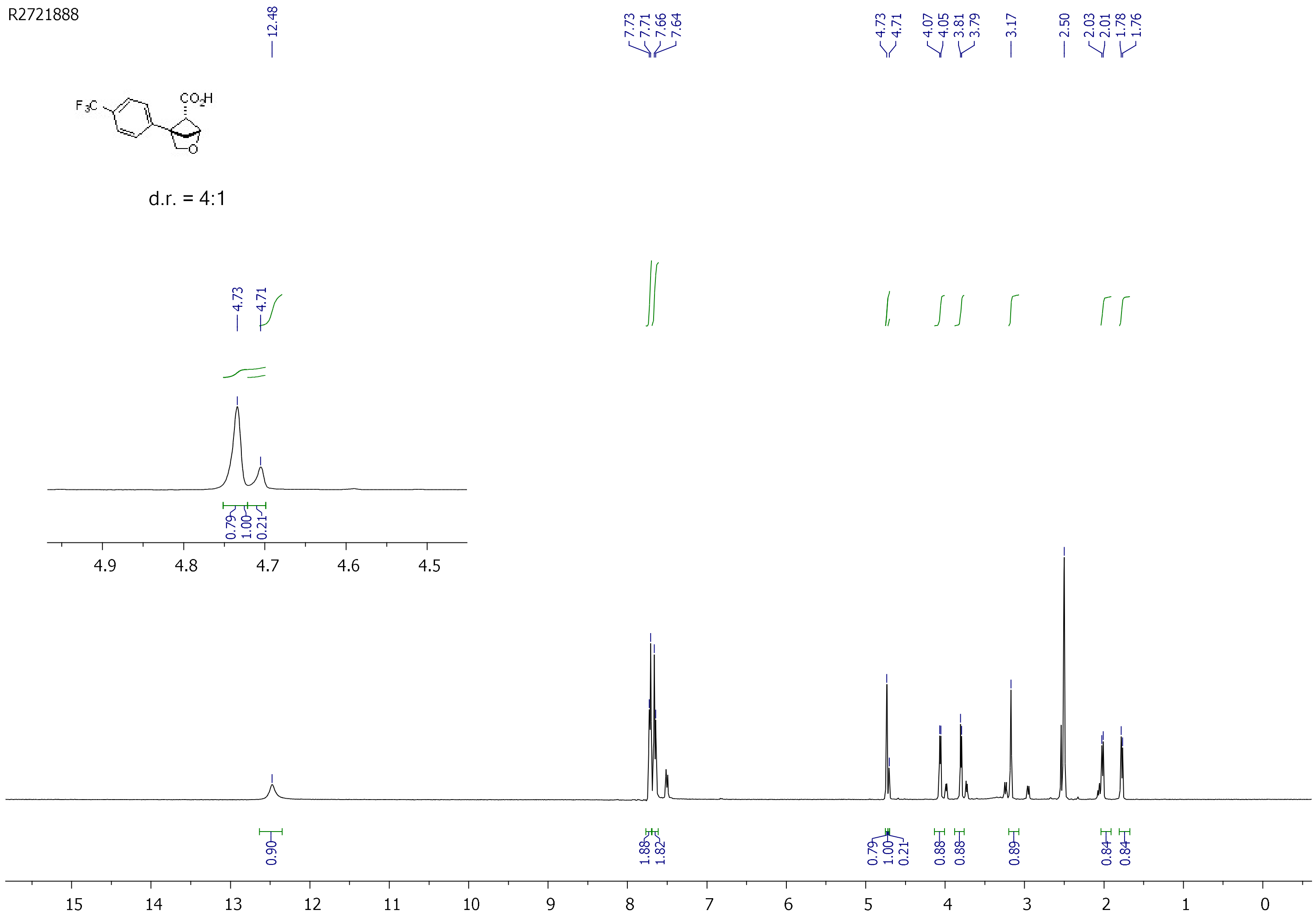

$^{13}C\{^1H\}$ NMR (126 MHz, DMSO-d$_6$)

R2721888_C13

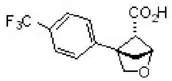

d.r. = 4:1

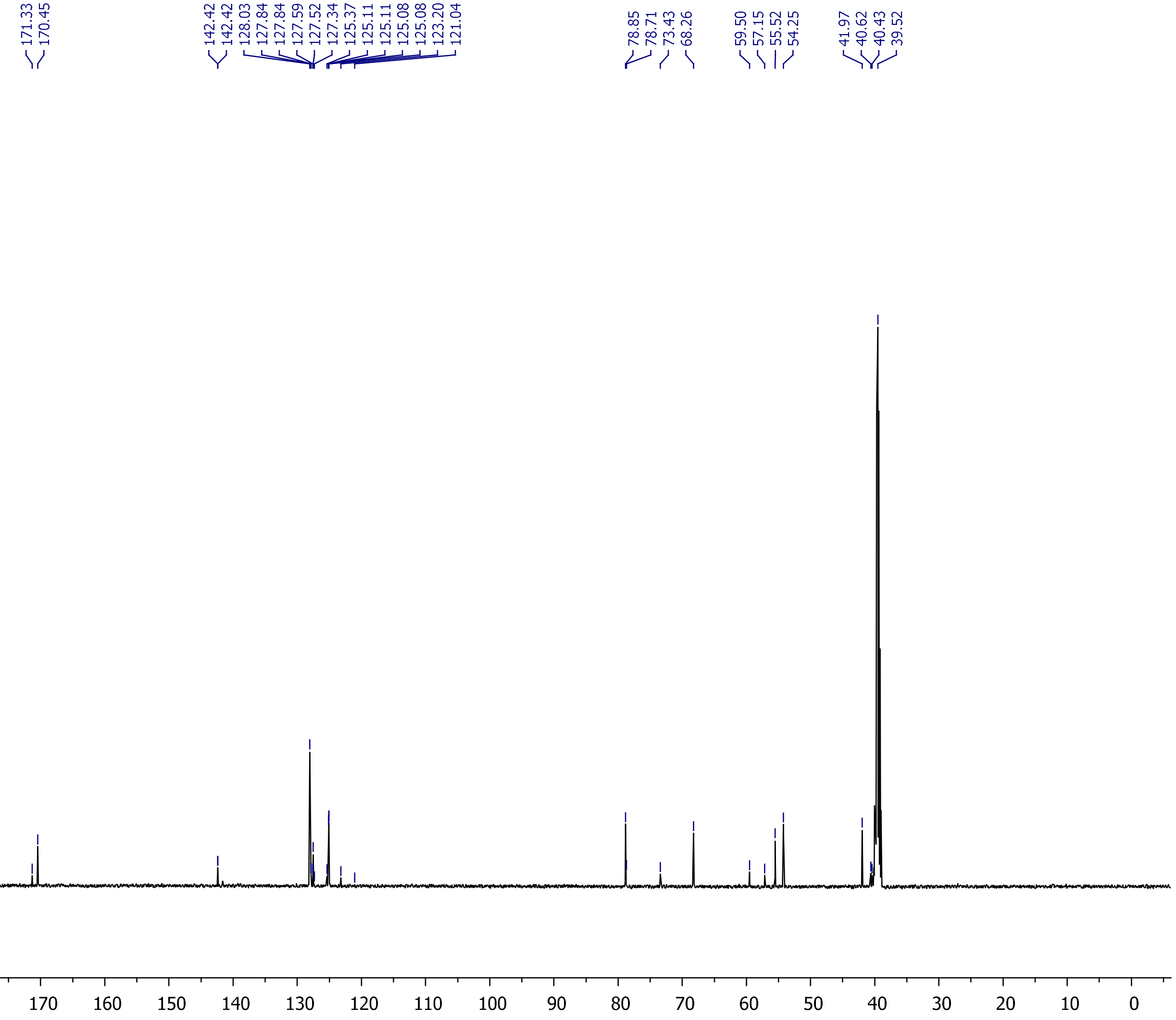

$^{19}F\{^{1}H\}$ NMR (376 MHz, DMSO-d_6)

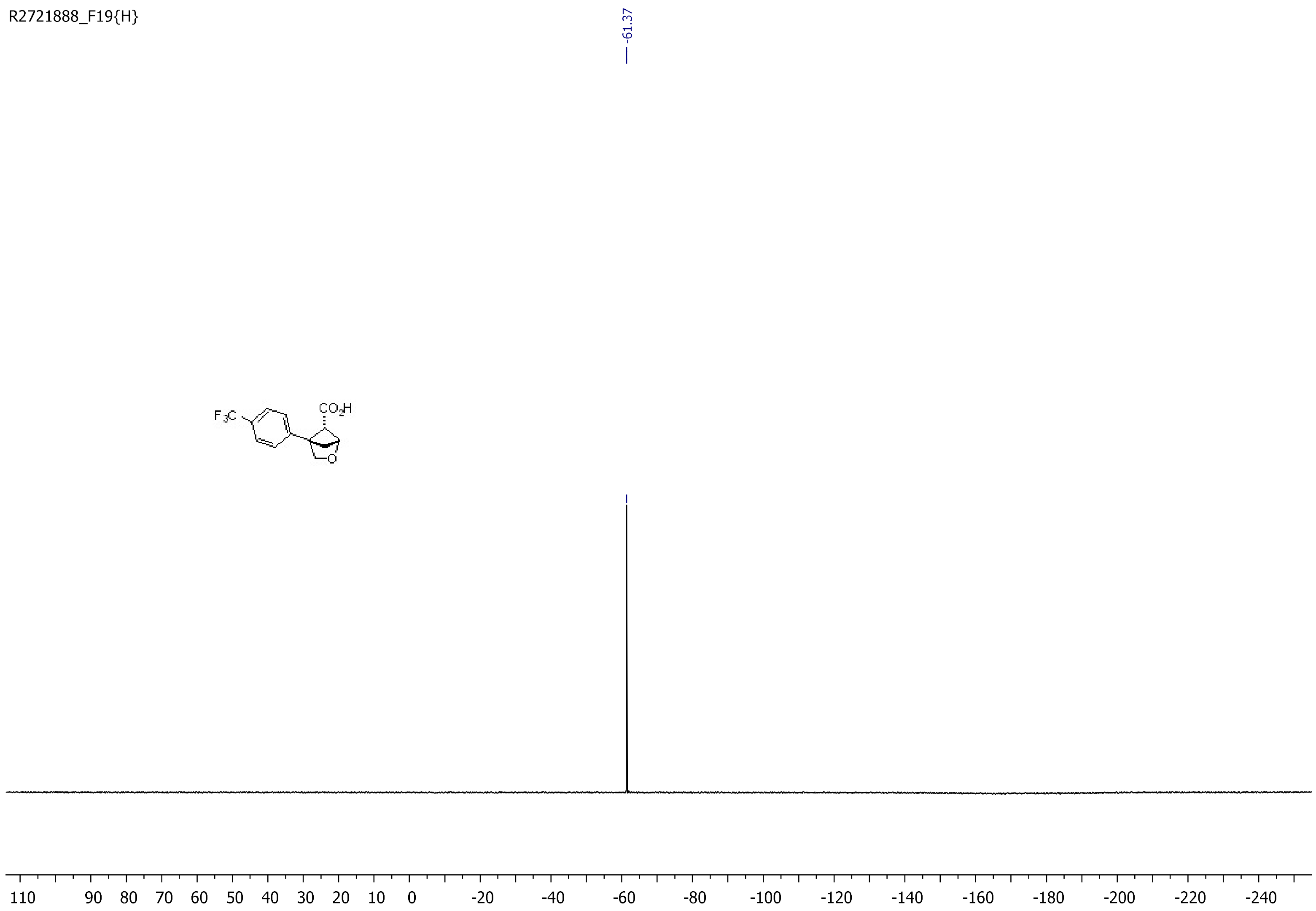

2-(Pyridin-3-yl)prop-2-en-1-ol

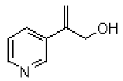

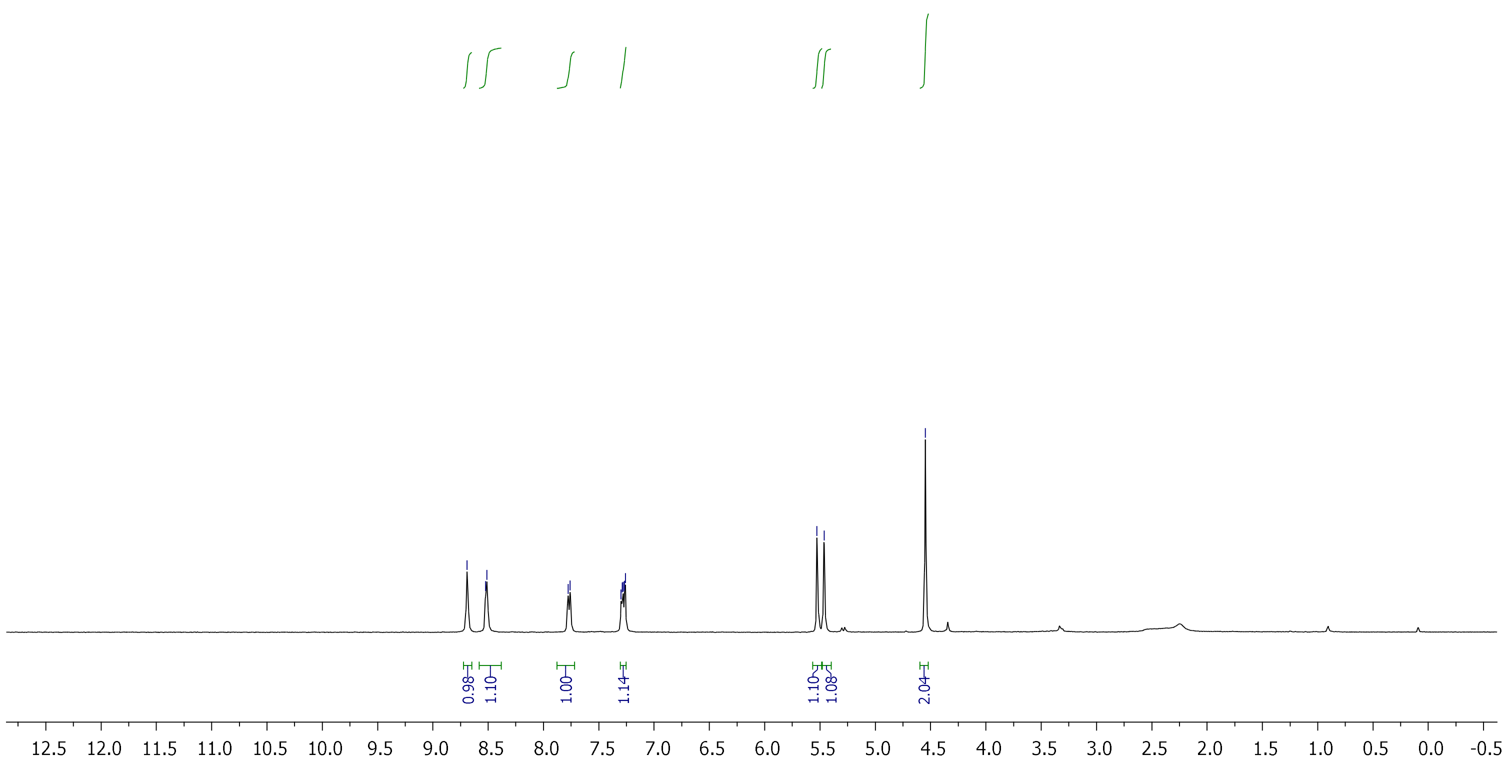

$^{13}C\{^{1}H\}$ NMR (126 MHz, $CDCl_3$)

R2904240_C13

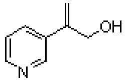

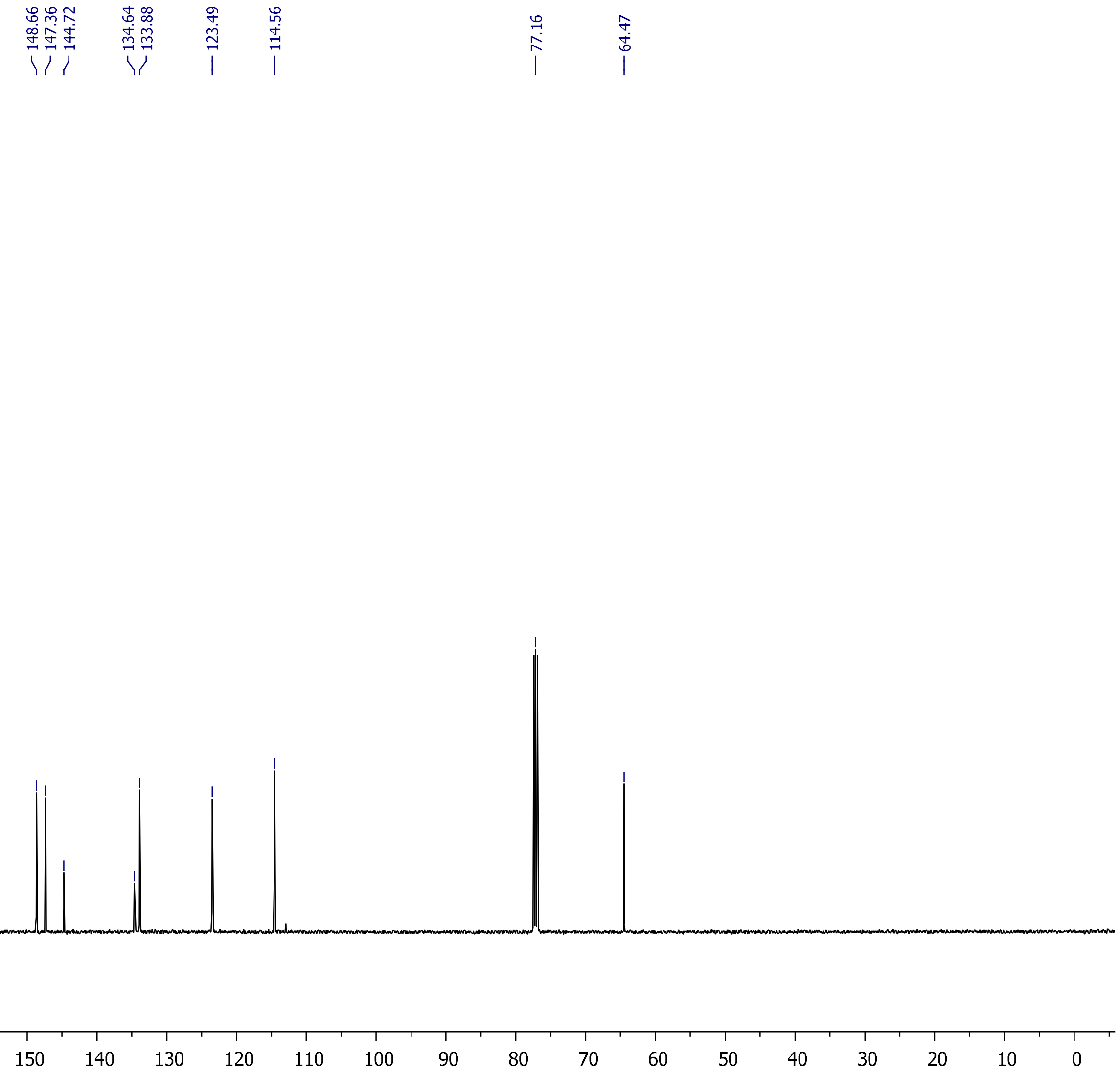

Compound (±)-20a

^{1}H NMR (400 MHz, $CDCl_3$)

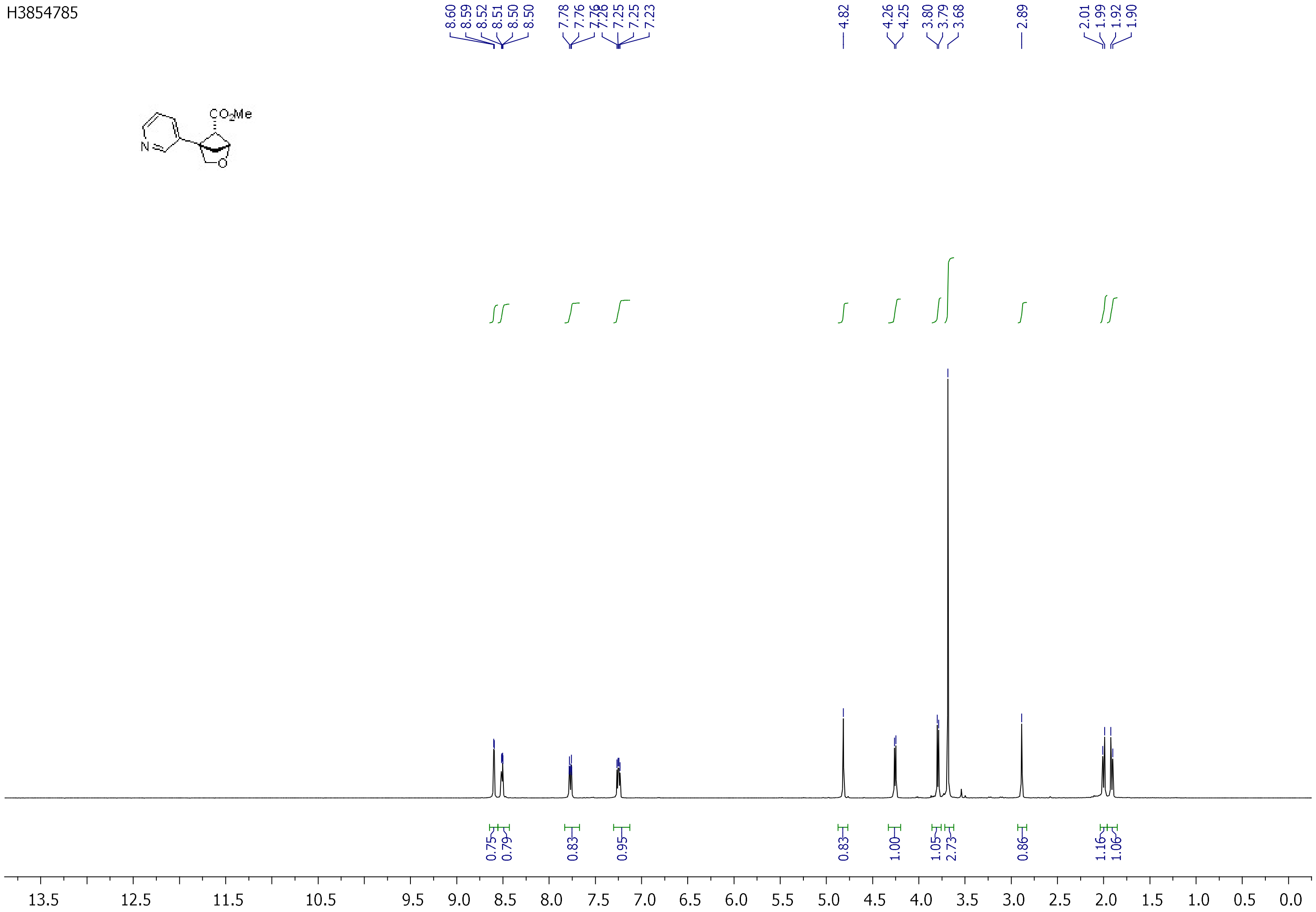

$^{13}C\{^1H\}$ NMR (126 MHz, $CDCl_3$)

H3854785_C13

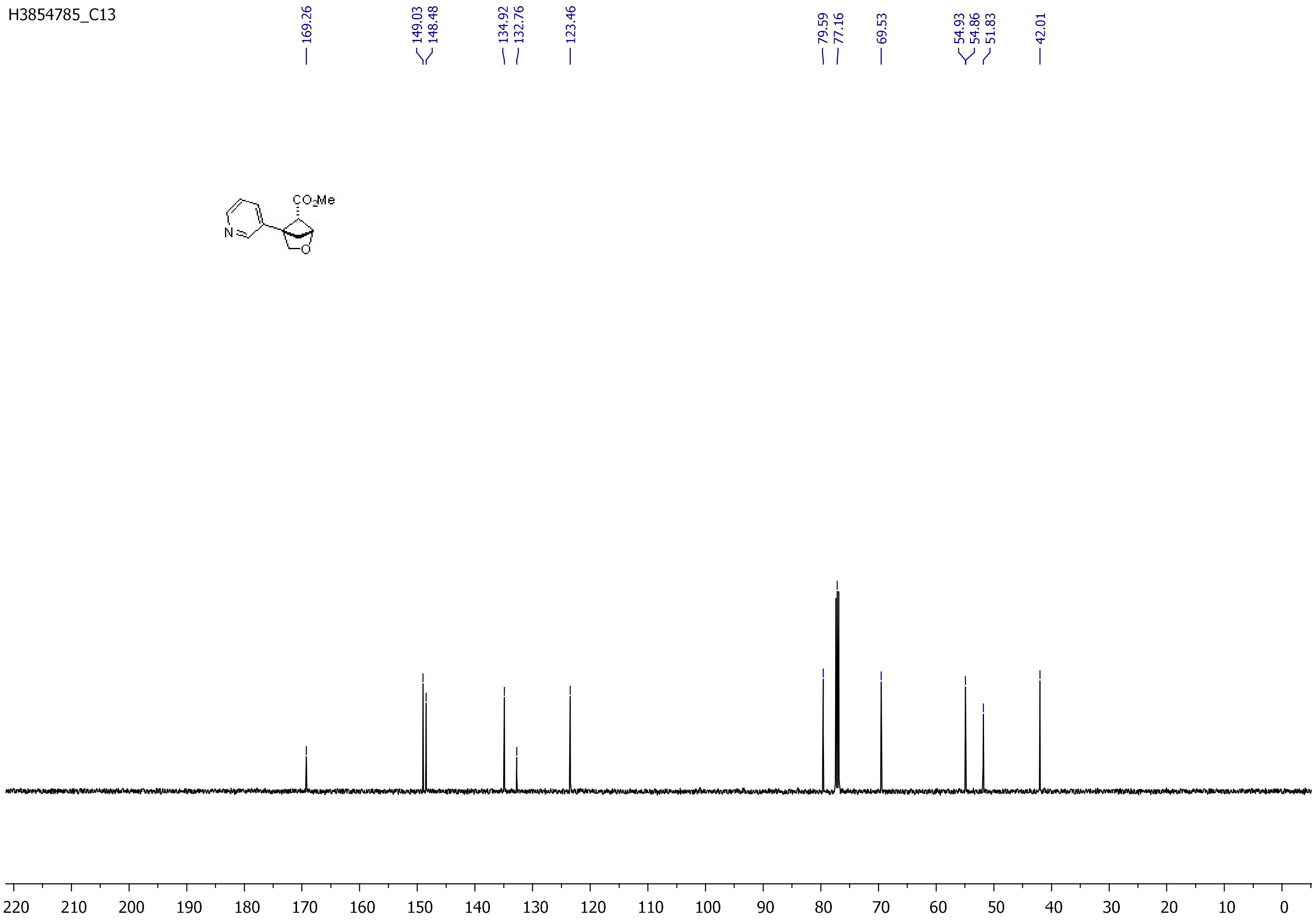

Compound (±)-20b

^1H NMR (400 MHz, DMSO-d_6)

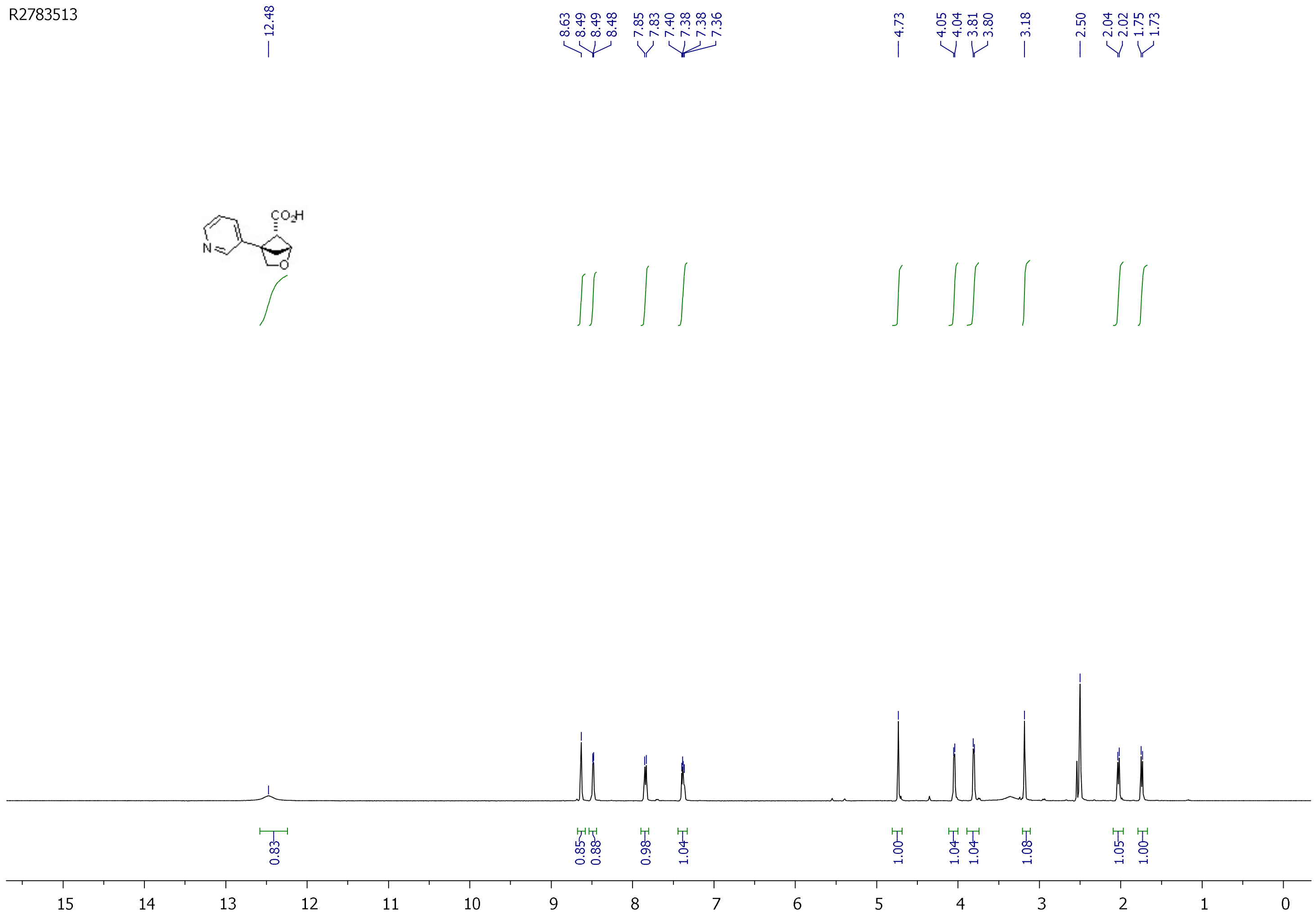

$^{13}C\{^1H\}$ NMR (126 MHz, DMSO-d$_6$)

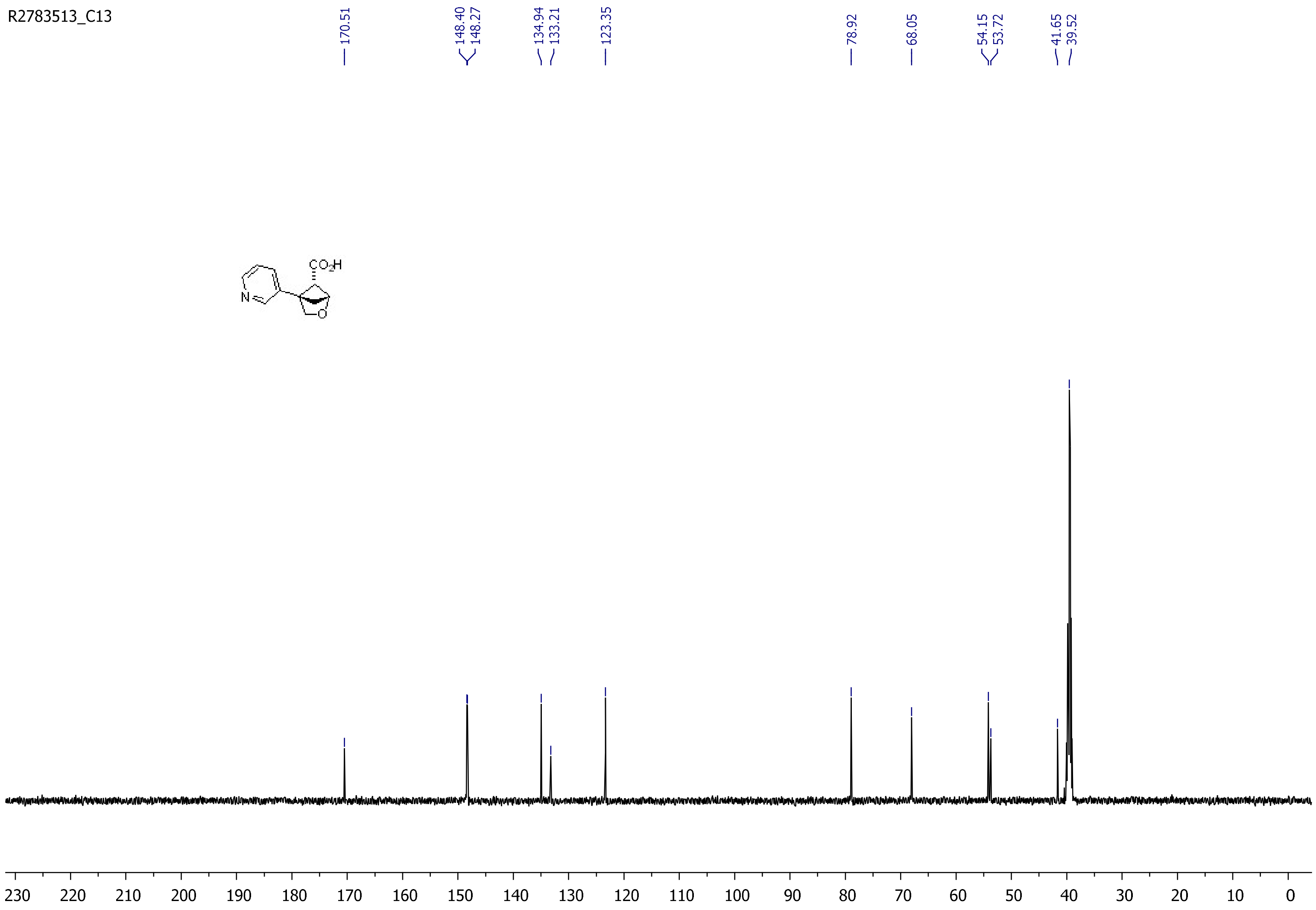

2-(4-Methylpyridin-3-yl)prop-2-en-1-ol

^{1}H NMR (400 MHz, $CDCl_3$)

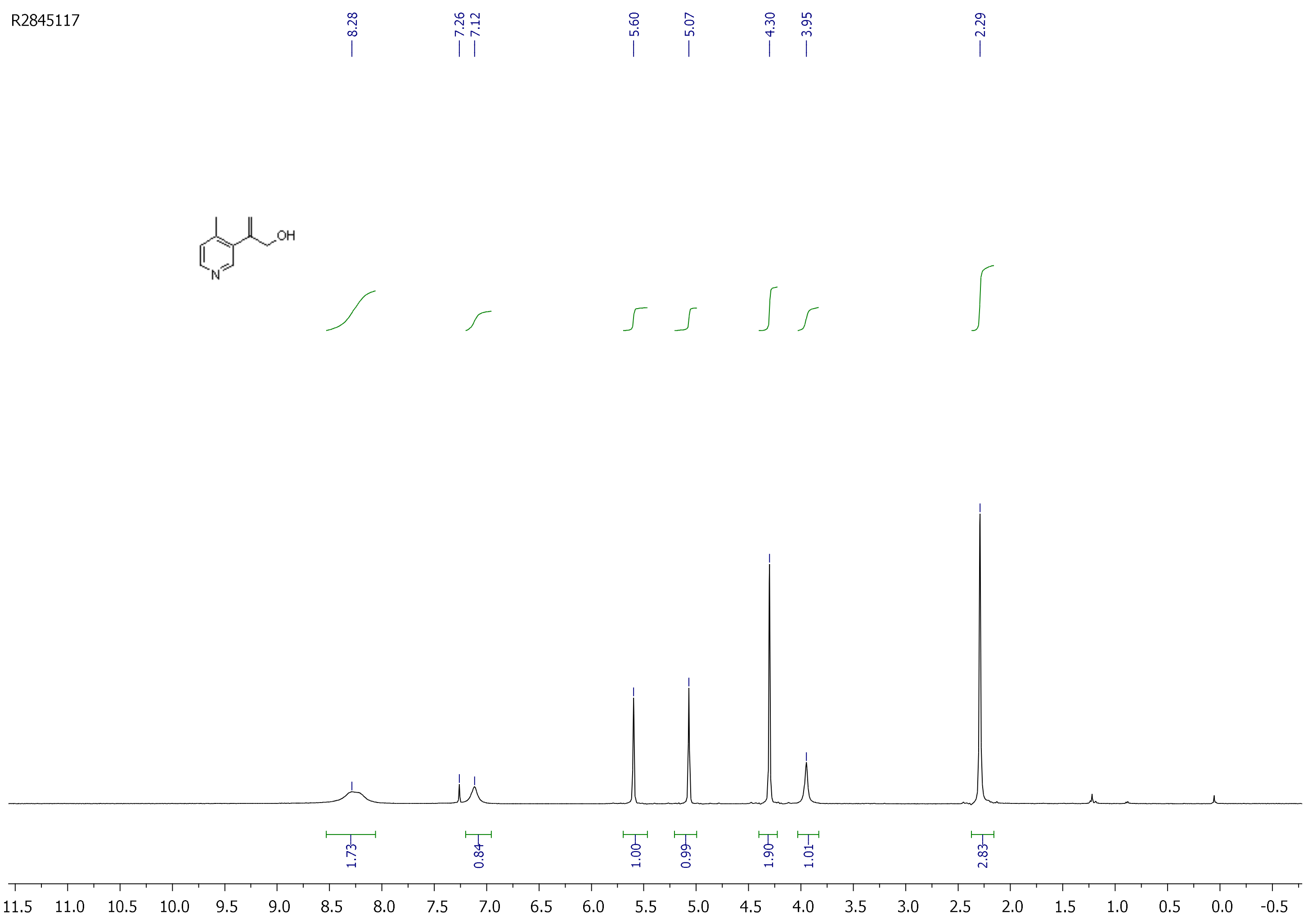

$^{13}C\{^1H\}$ NMR (126 MHz, $CDCl_3$)

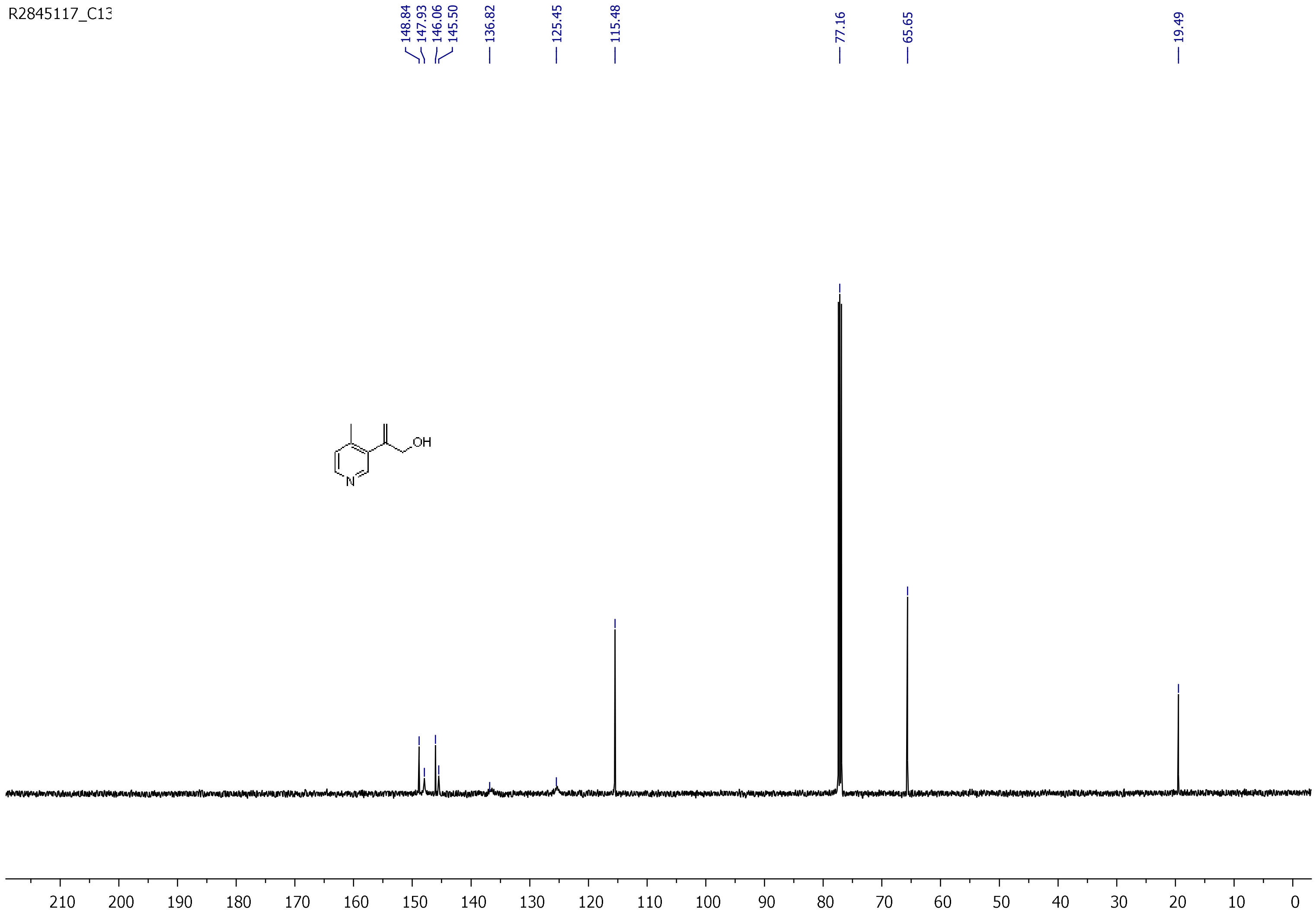

Compound (±)-21a

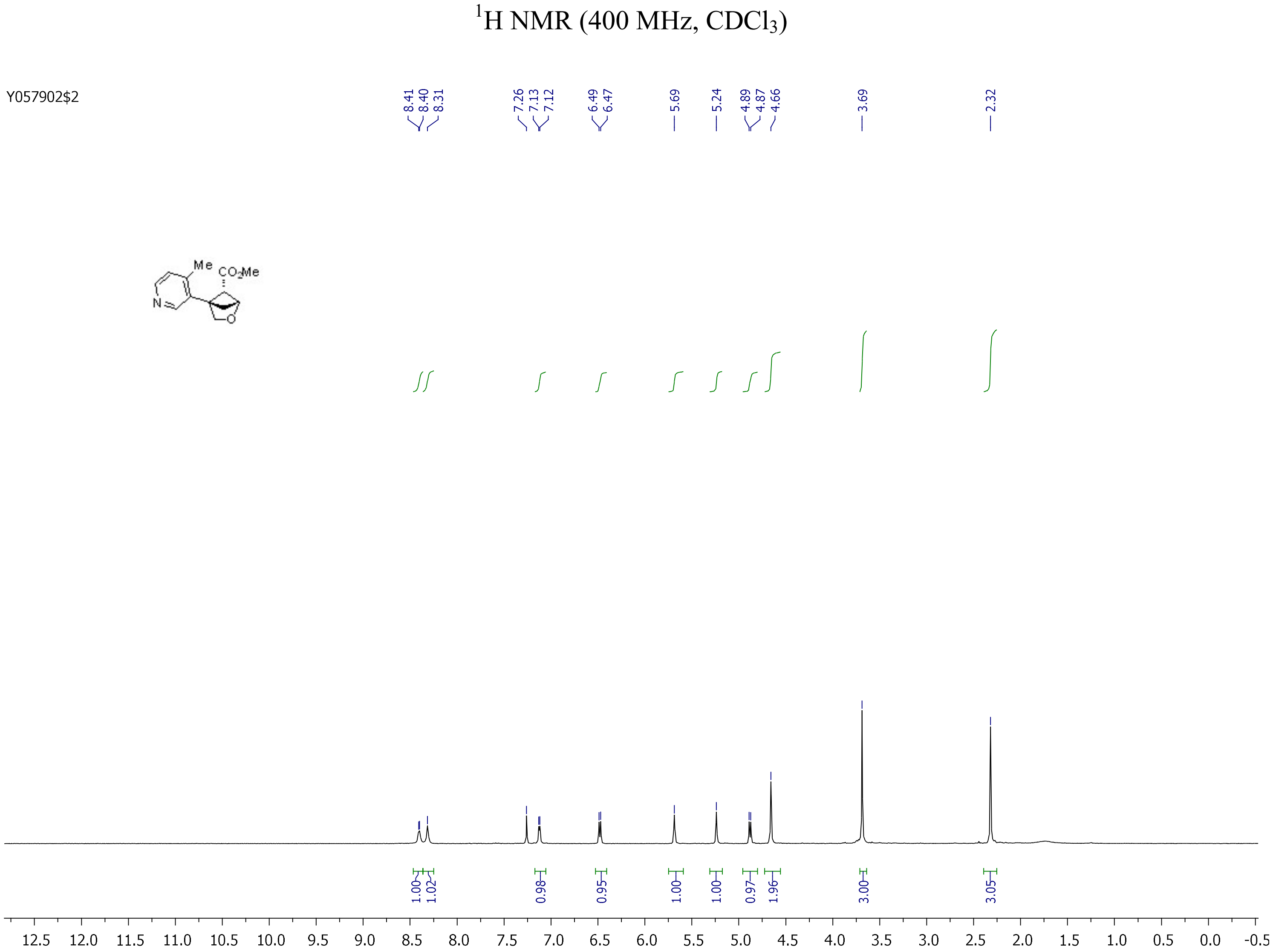

$^{13}C\{^1H\}$ NMR (151 MHz, $CDCl_3$)

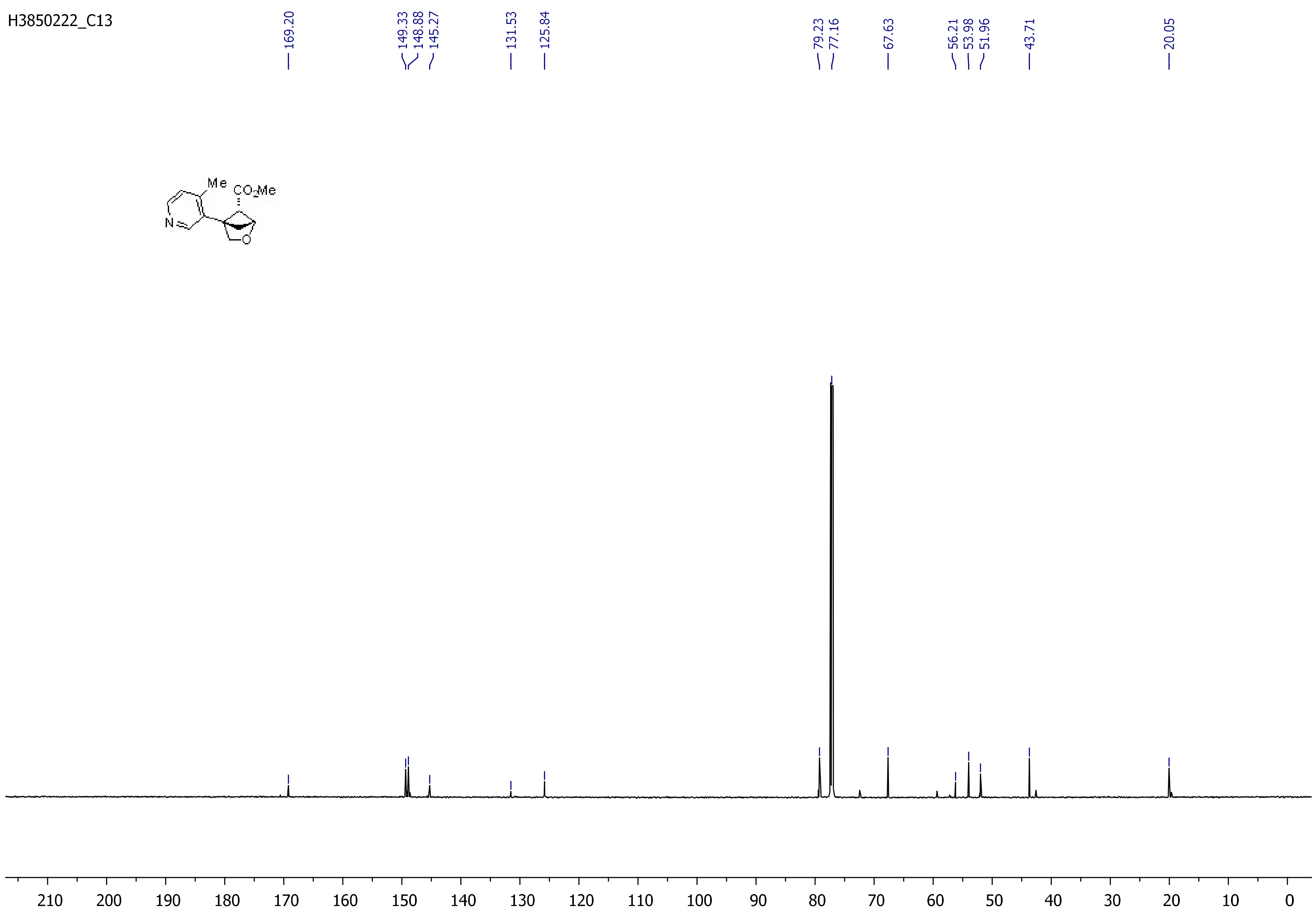

Compound (±)-21b

^{1}H NMR (400 MHz, DMSO-d$_6$)

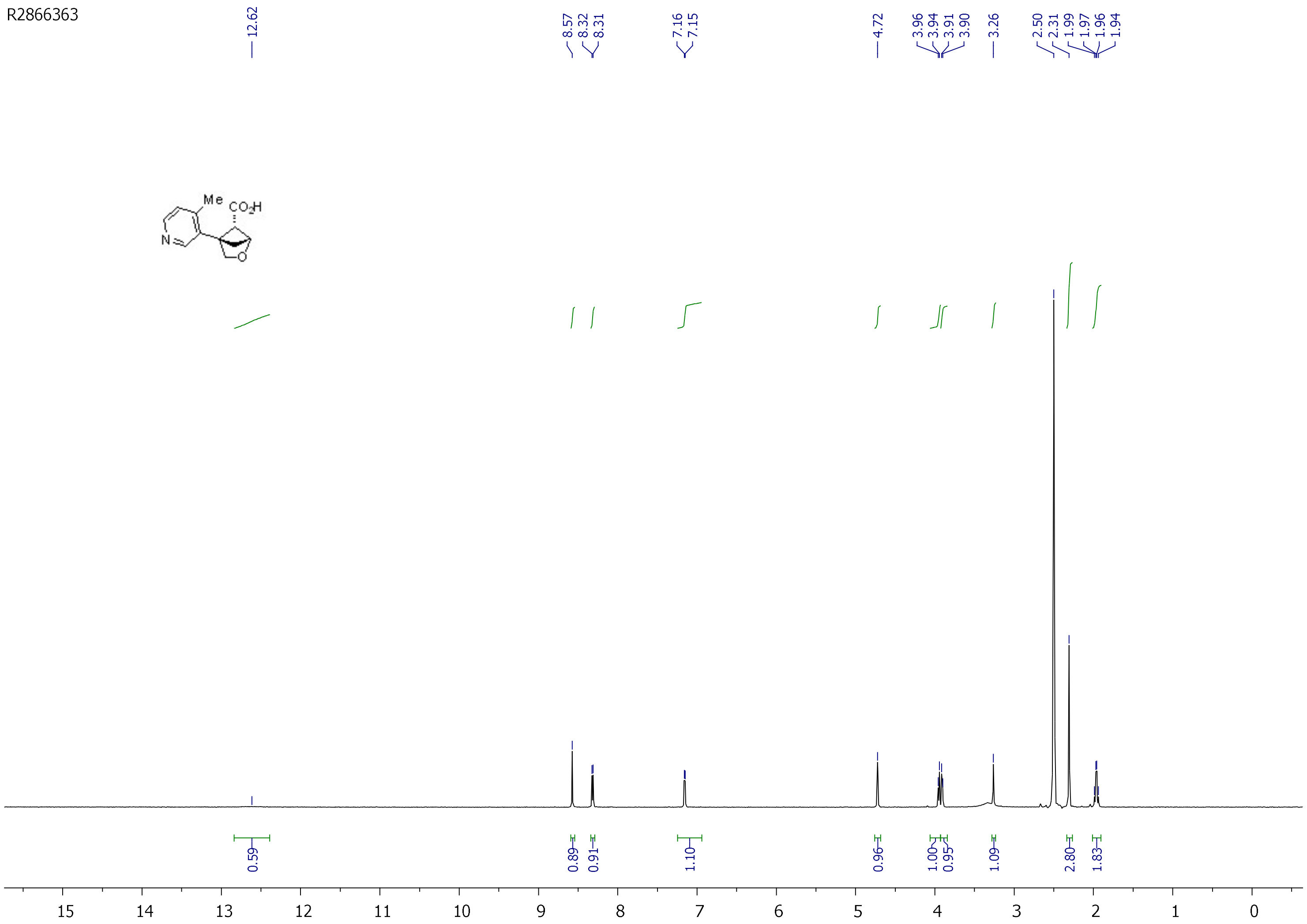

$^{13}C\{^{1}H\}$ NMR (126 MHz, DMSO-d_6)

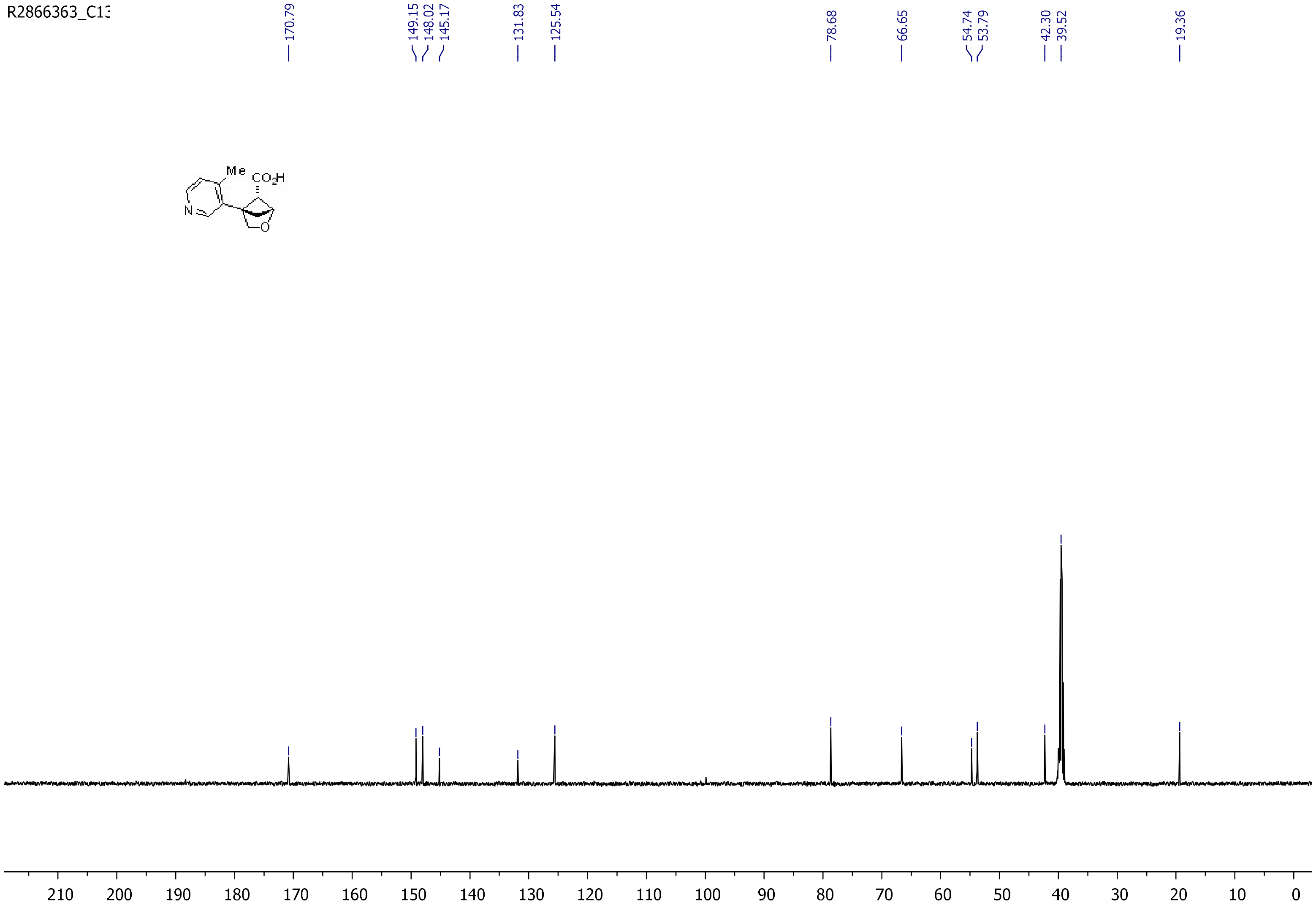

2-(5-Methylpyridin-3-yl)prop-2-en-1-ol

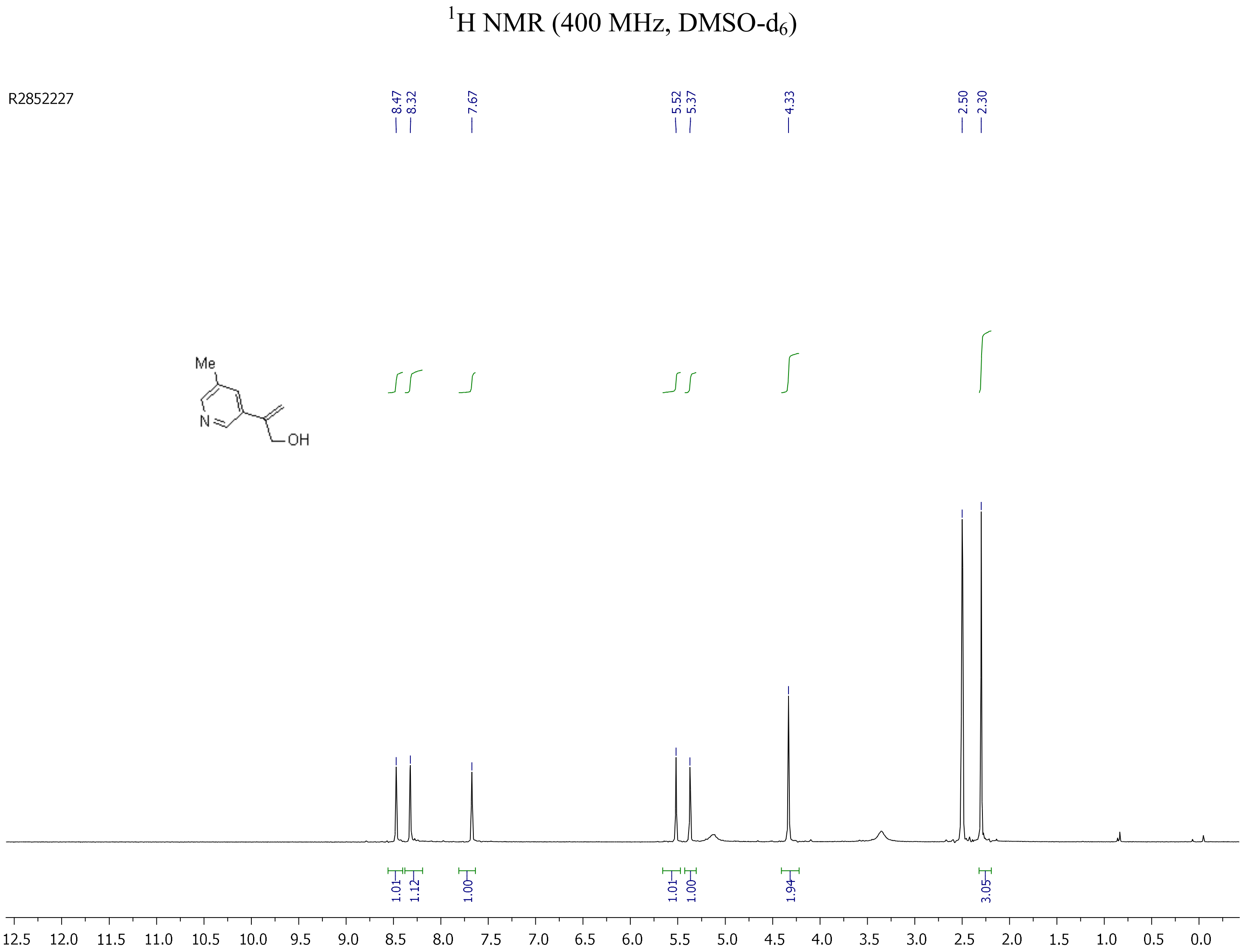

$^{13}C\{^{1}H\}$ NMR (126 MHz, DMSO-d_6)

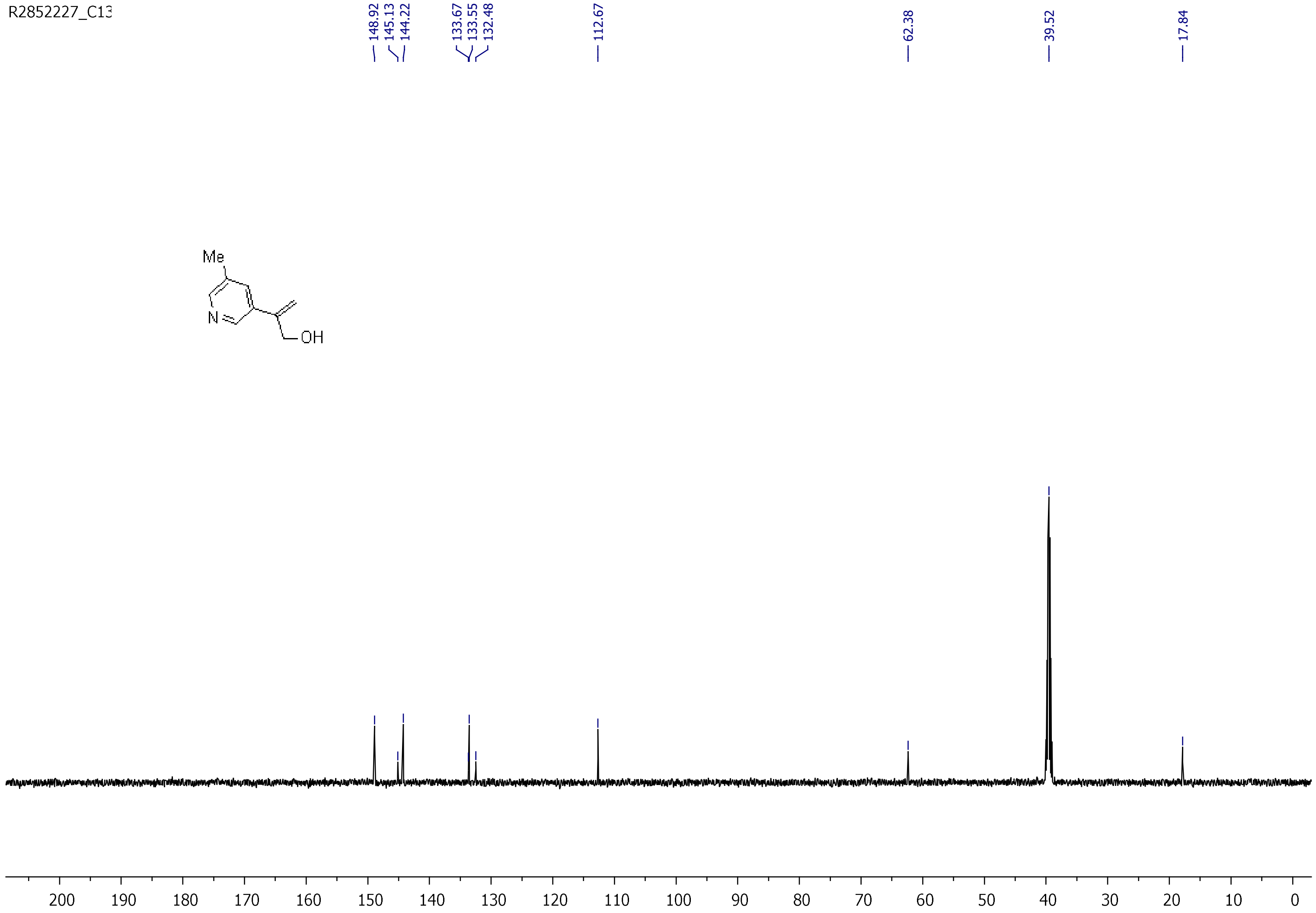

Compound (±)-22a, d.r. = 4:1

^{1}H NMR (400 MHz, $CDCl_3$)

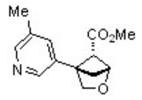

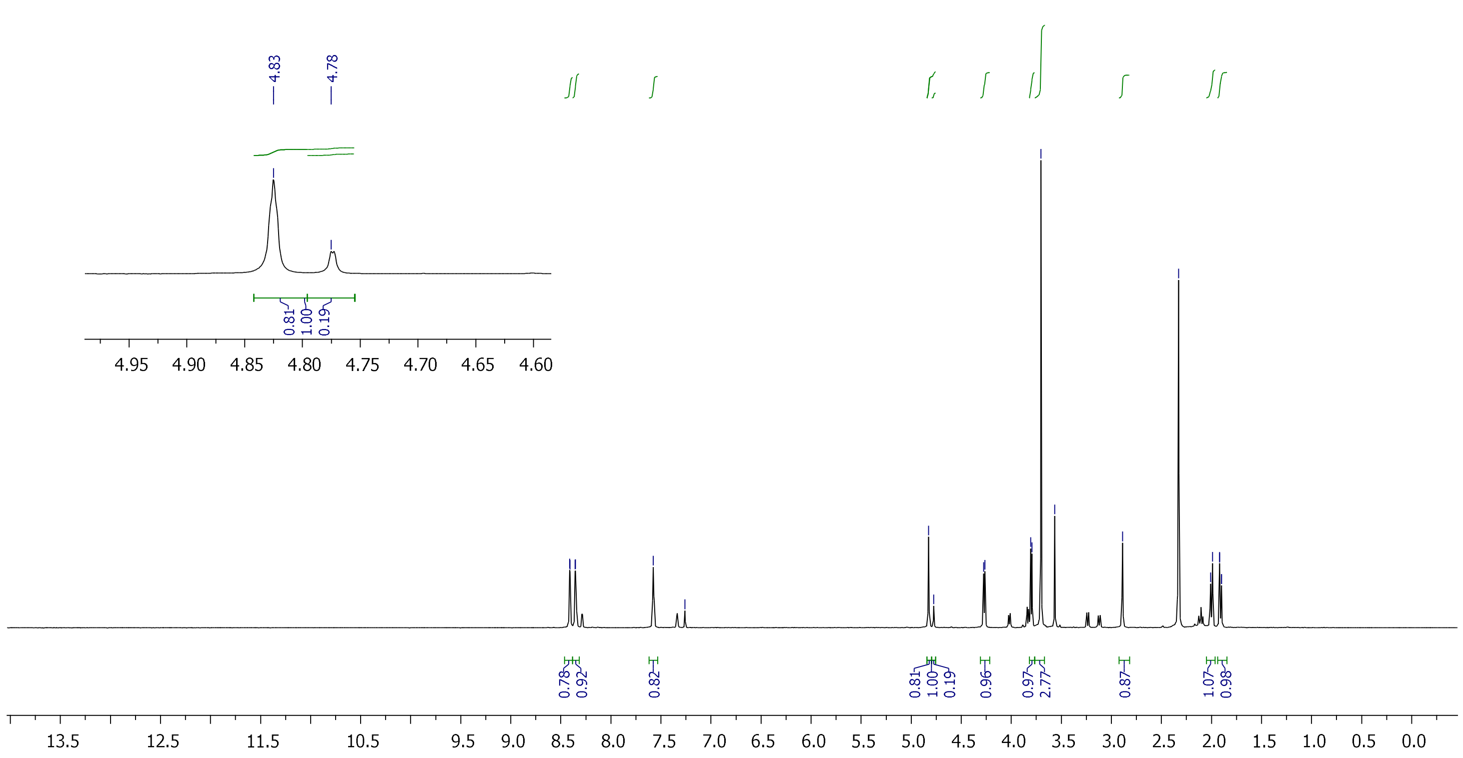

$^{13}C\{^1H\}$ NMR (126 MHz, $CDCl_3$)

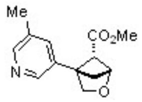

d.r. = 4:1

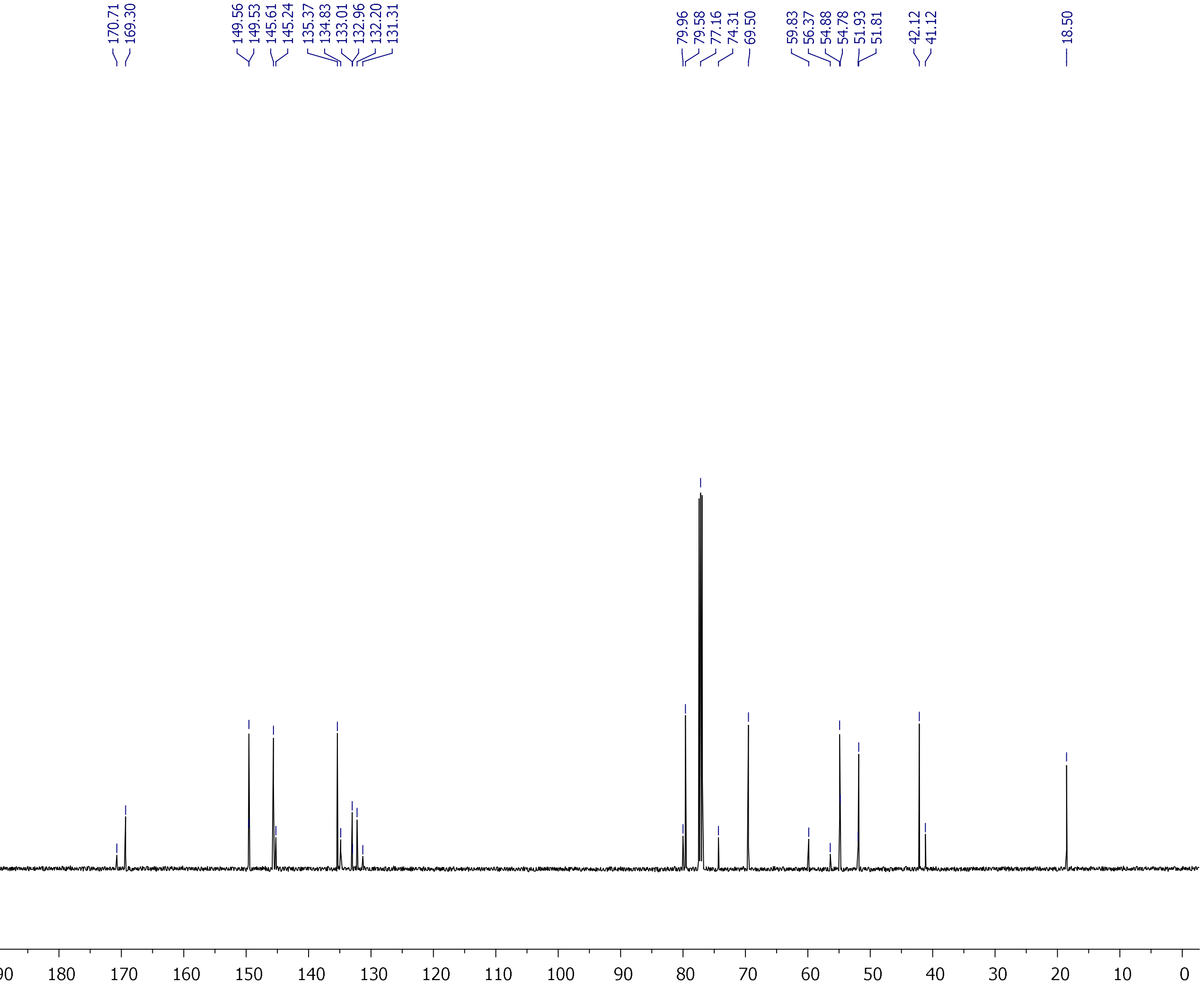

Compound (±)-22b

^{1}H NMR (600 MHz, DMSO-d$_6$)

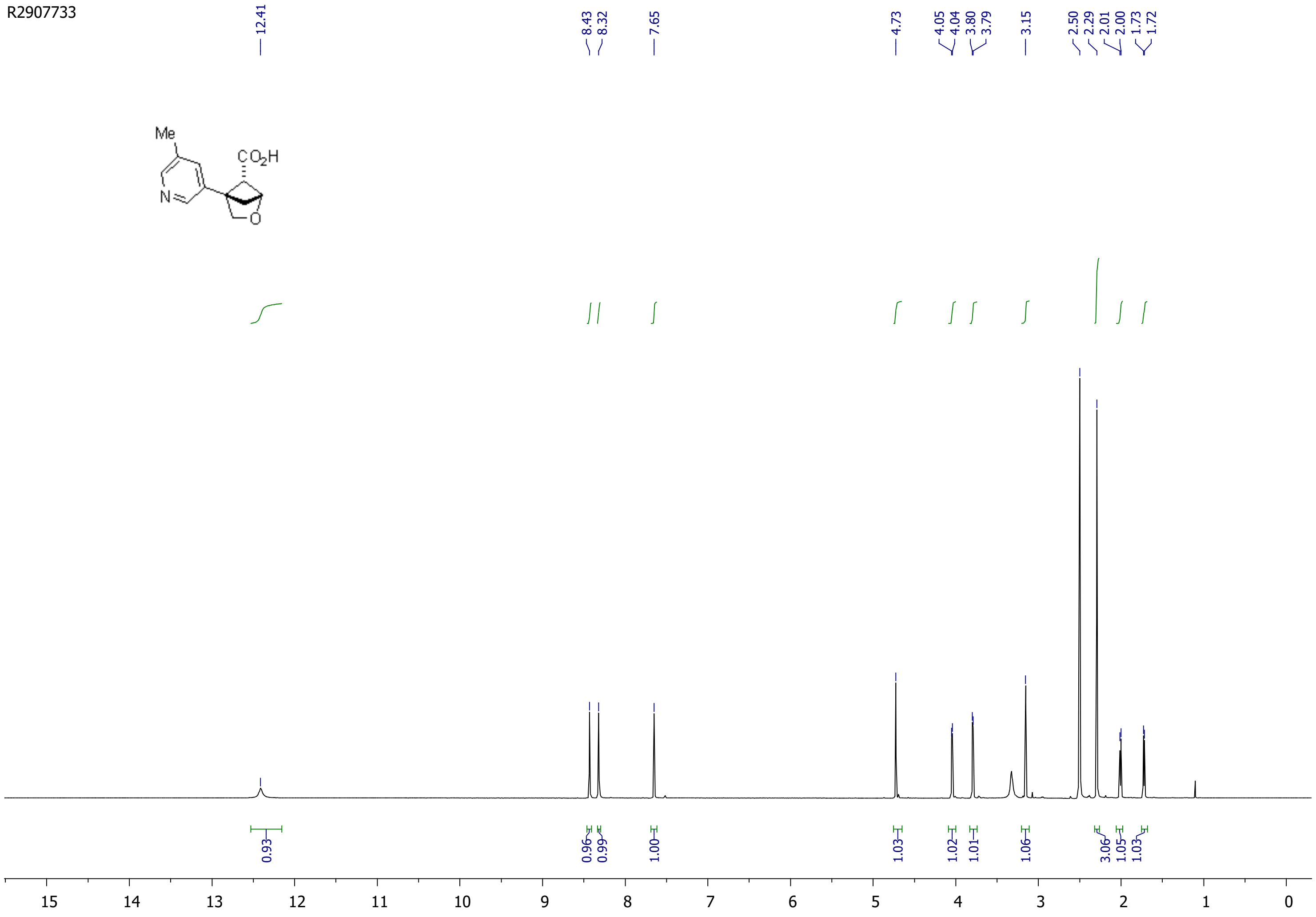

$^{13}C\{^1H\}$ NMR (151 MHz, DMSO-d_6)

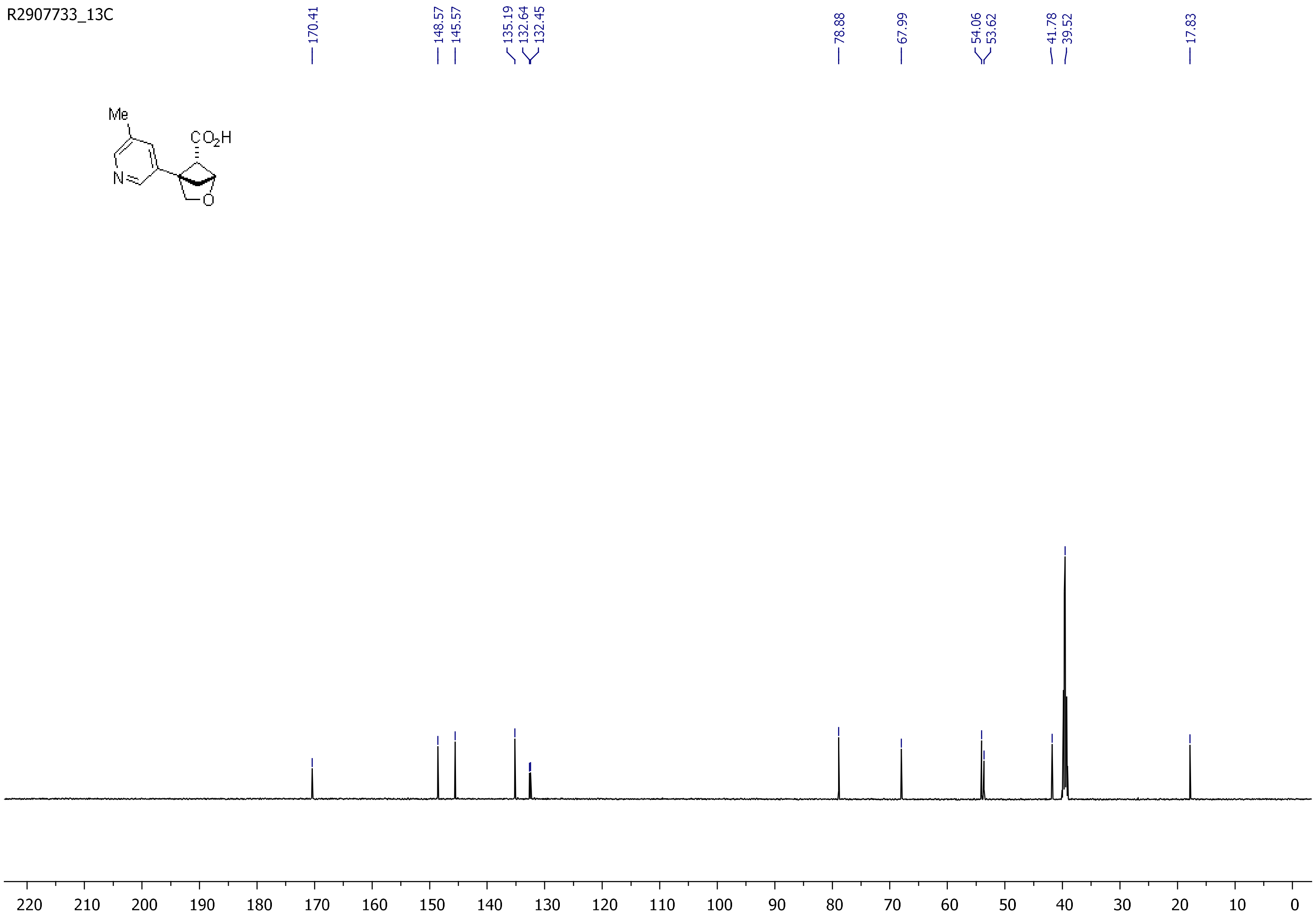

2-(5-Fluoropyridin-3-yl)prop-2-en-1-ol

^{1}H NMR (400 MHz, $CDCl_3$)

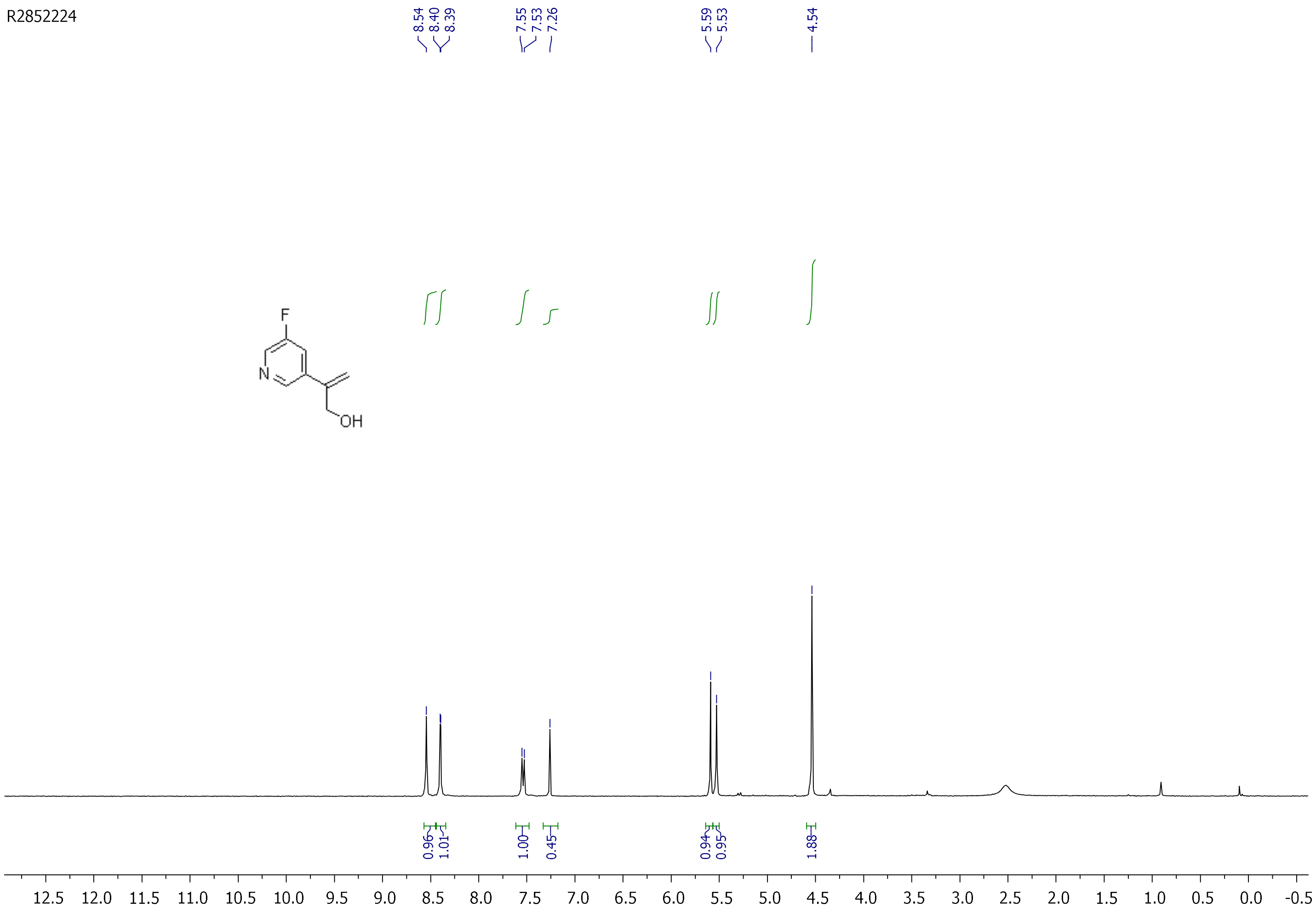

$^{13}C\{^{1}H\}$ NMR (126 MHz, $CDCl_3$)

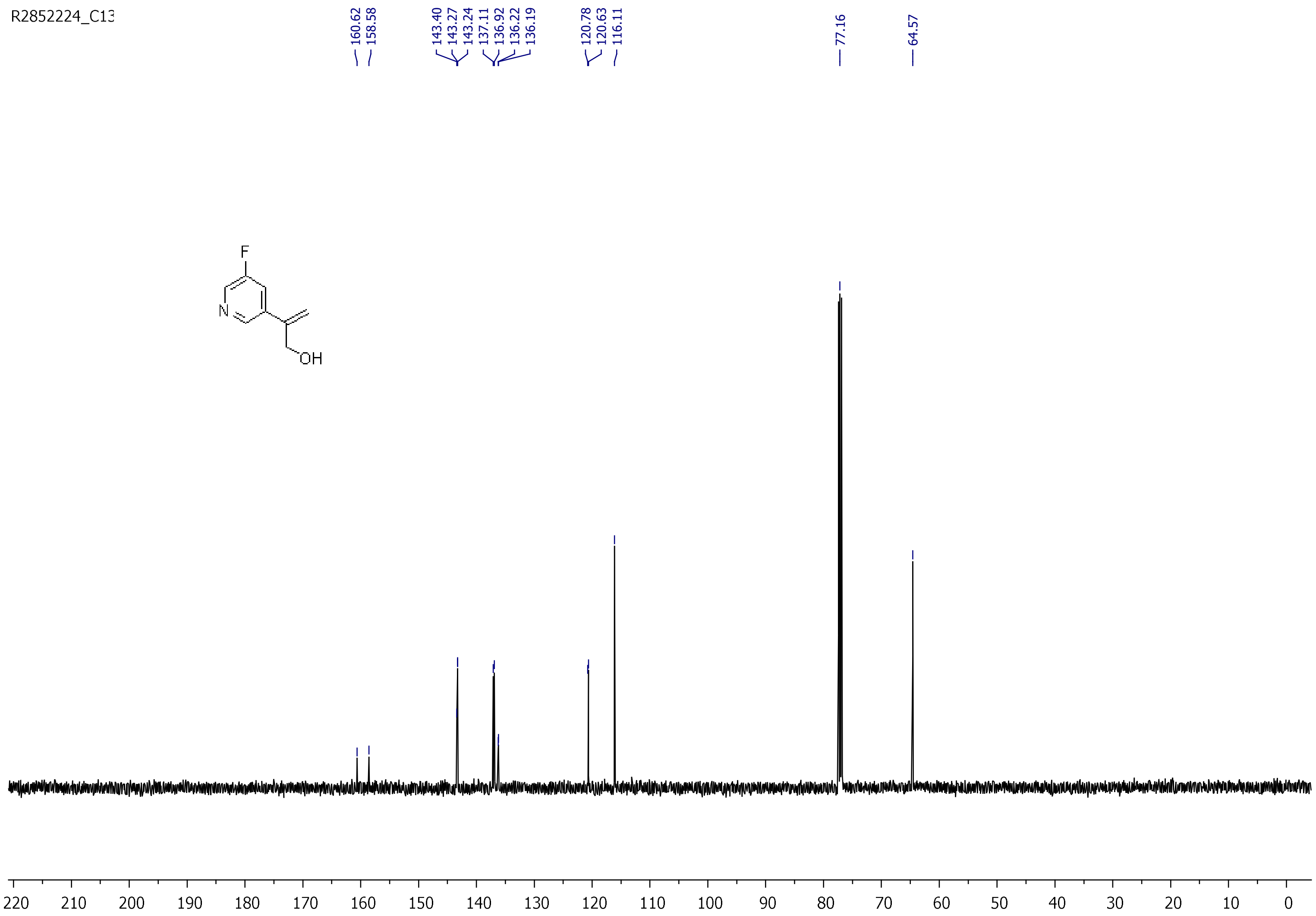

$^{19}F\{^1H\}$ NMR (376 MHz, $CDCl_3$)

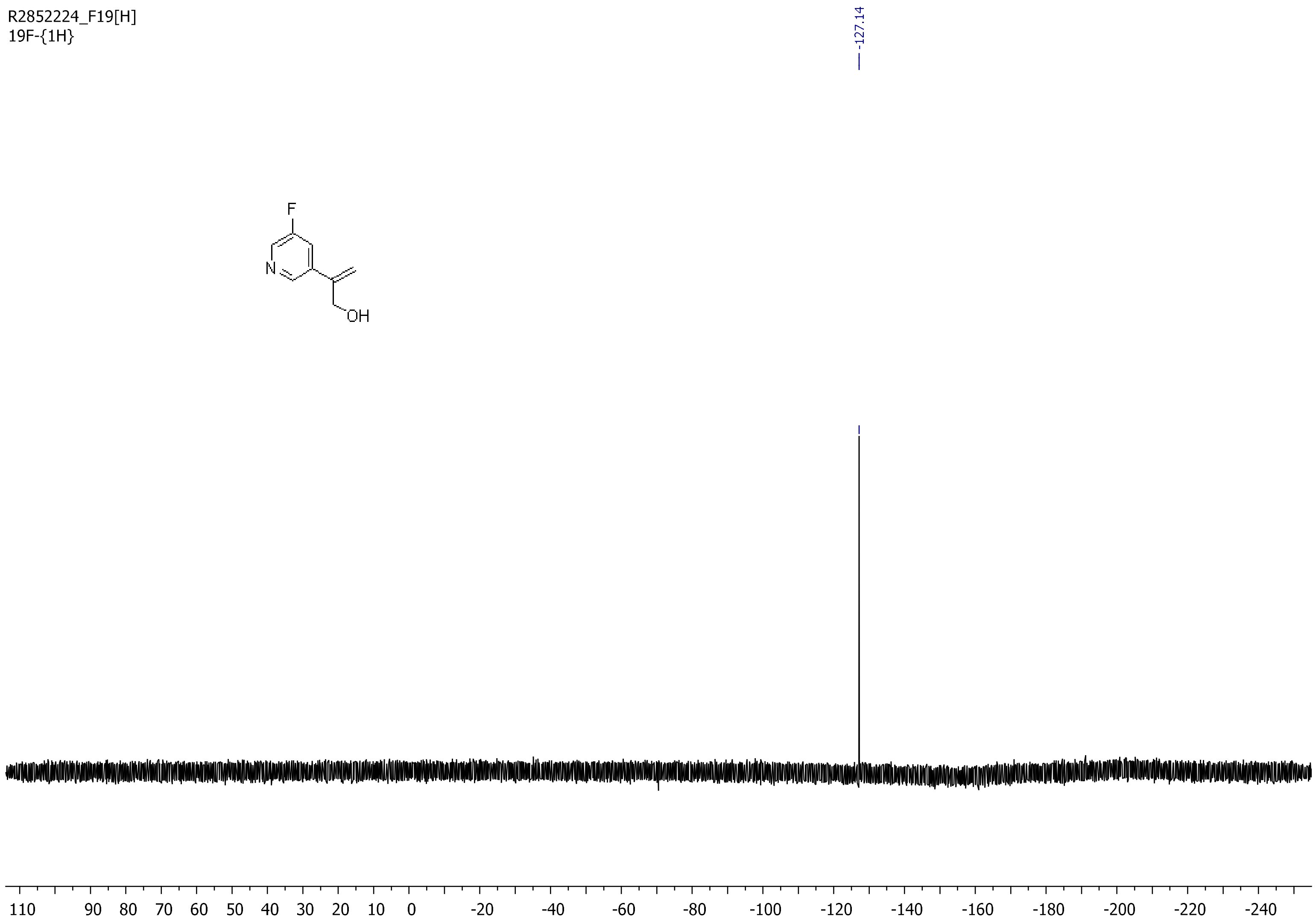

Compound (±)-23a, d.r. = 9:2

^{1}H NMR (400 MHz, $CDCl_3$)

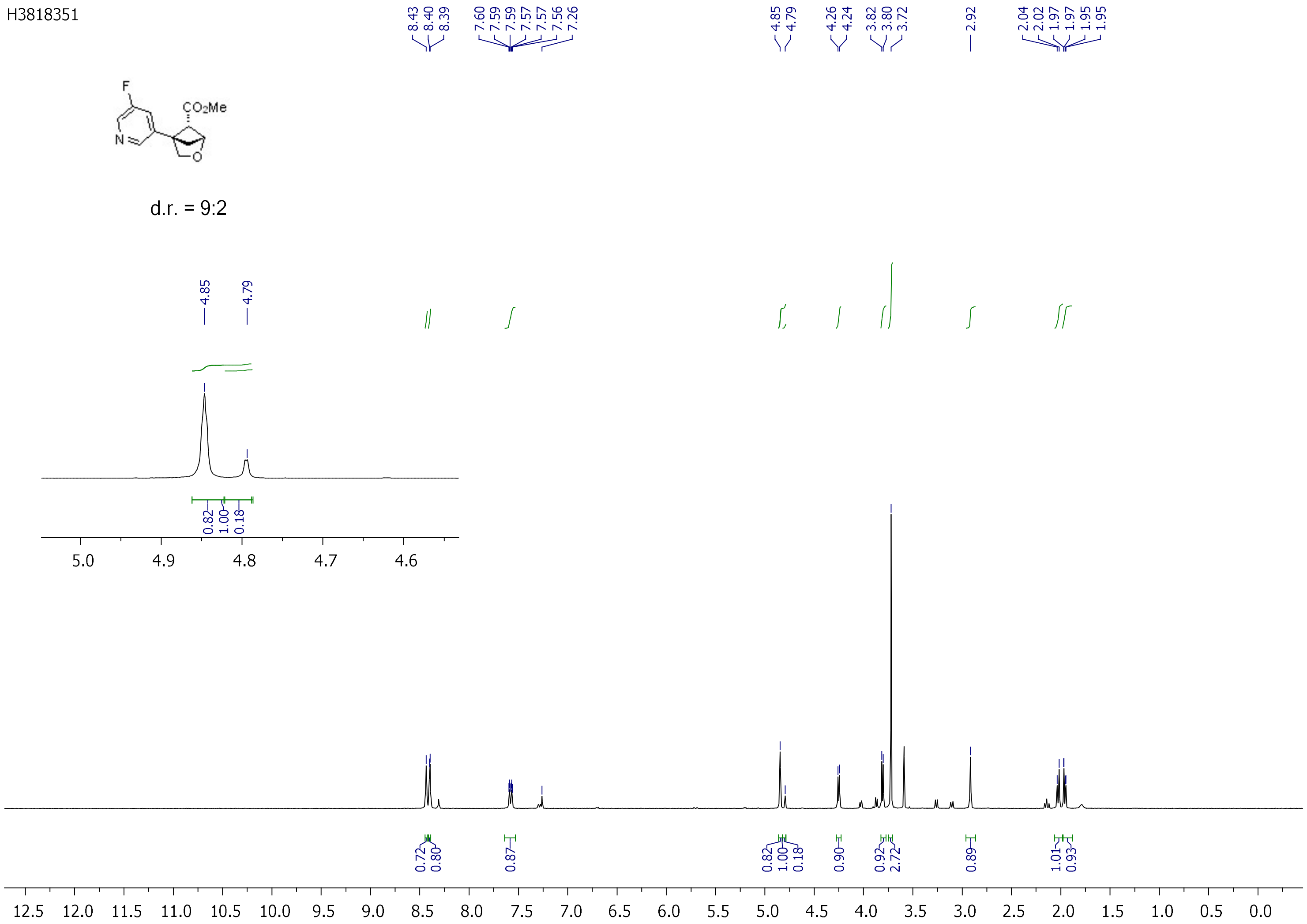

$^{13}C\{^{1}H\}$ NMR (126 MHz, $CDCl_3$)

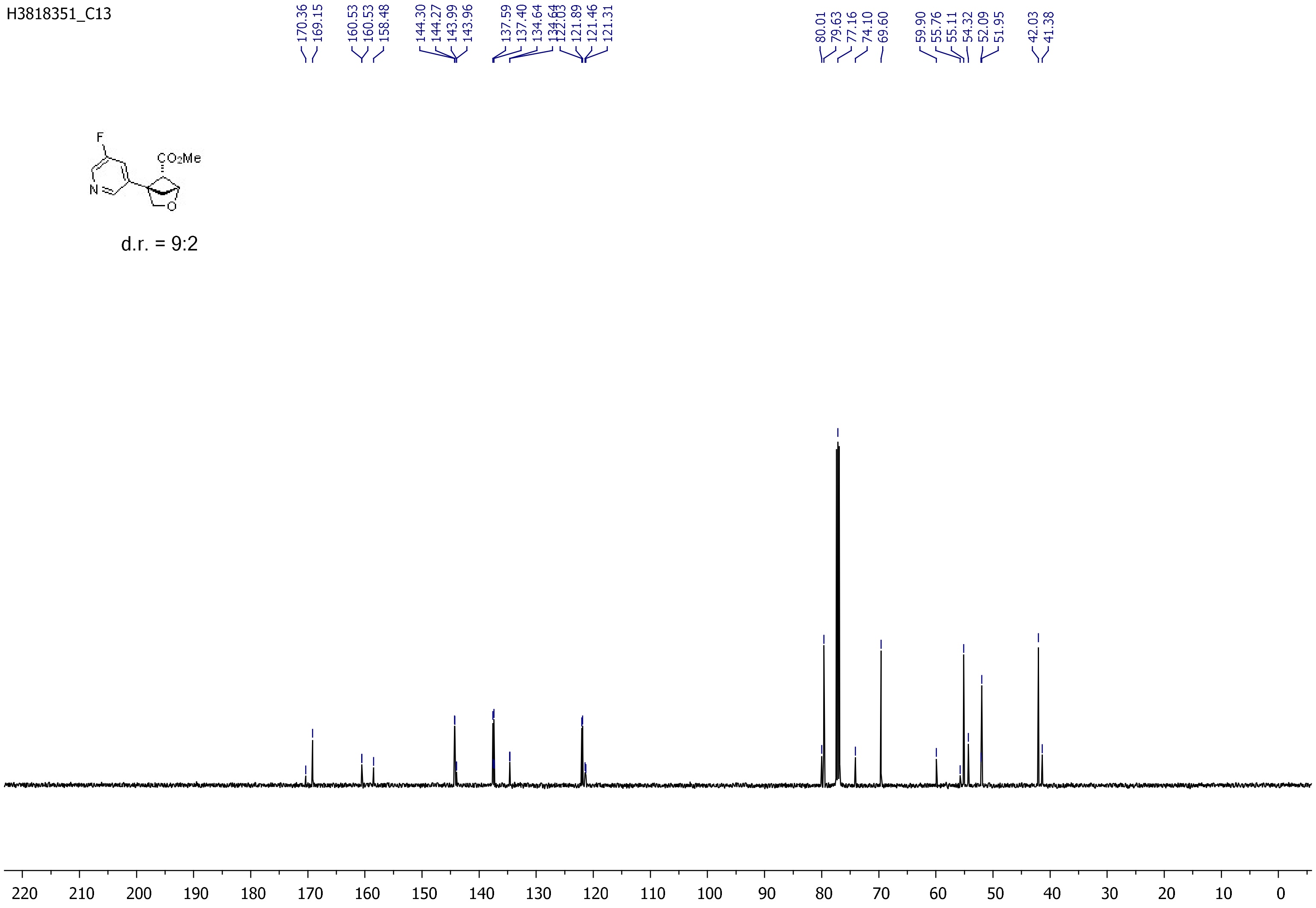

$^{19}F\{^{1}H\}$ NMR (376 MHz, $CDCl_3$)

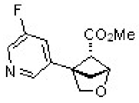

d.r. = 9:2

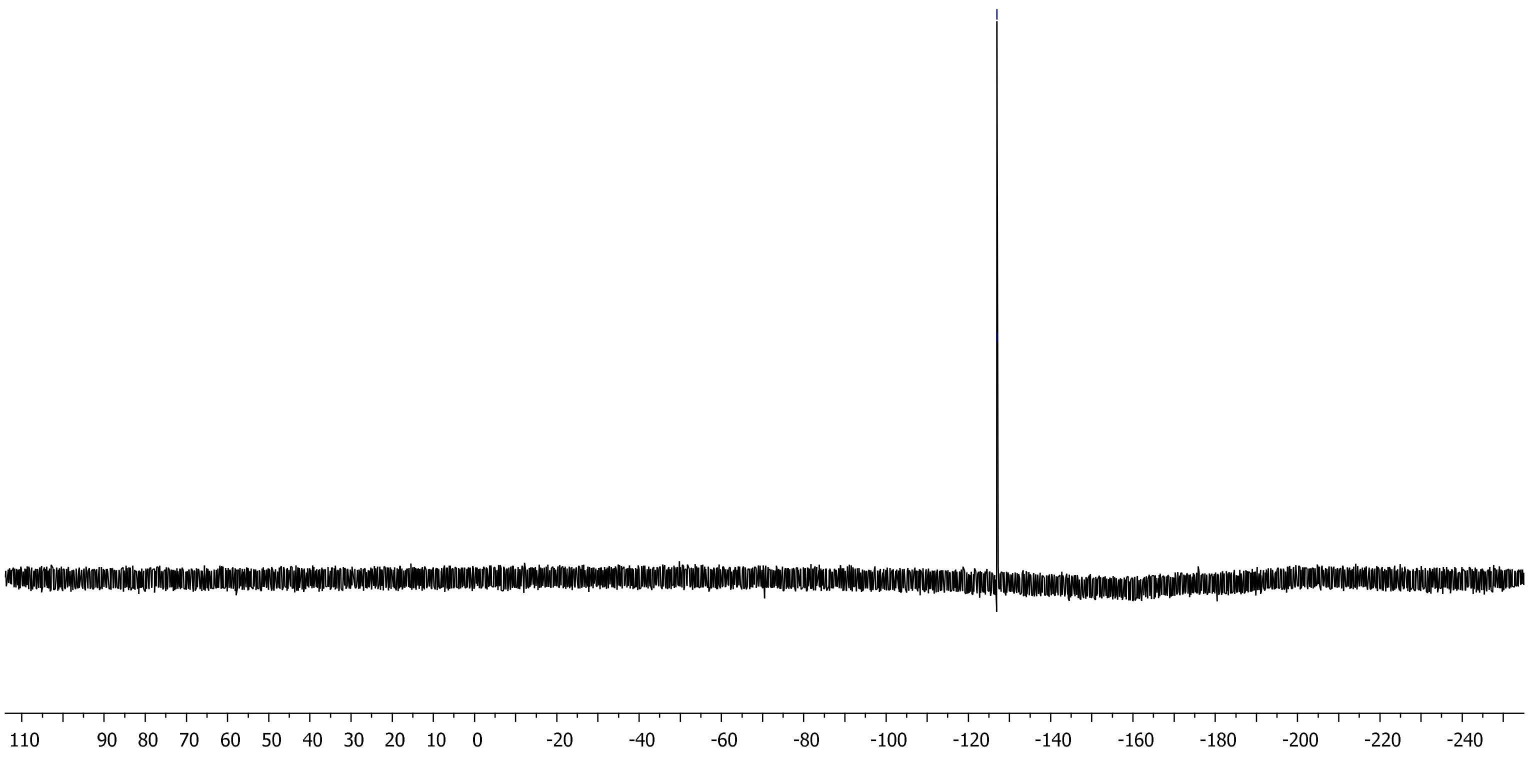

Compound (±)-23b

^{1}H NMR (400 MHz, DMSO-d_6)

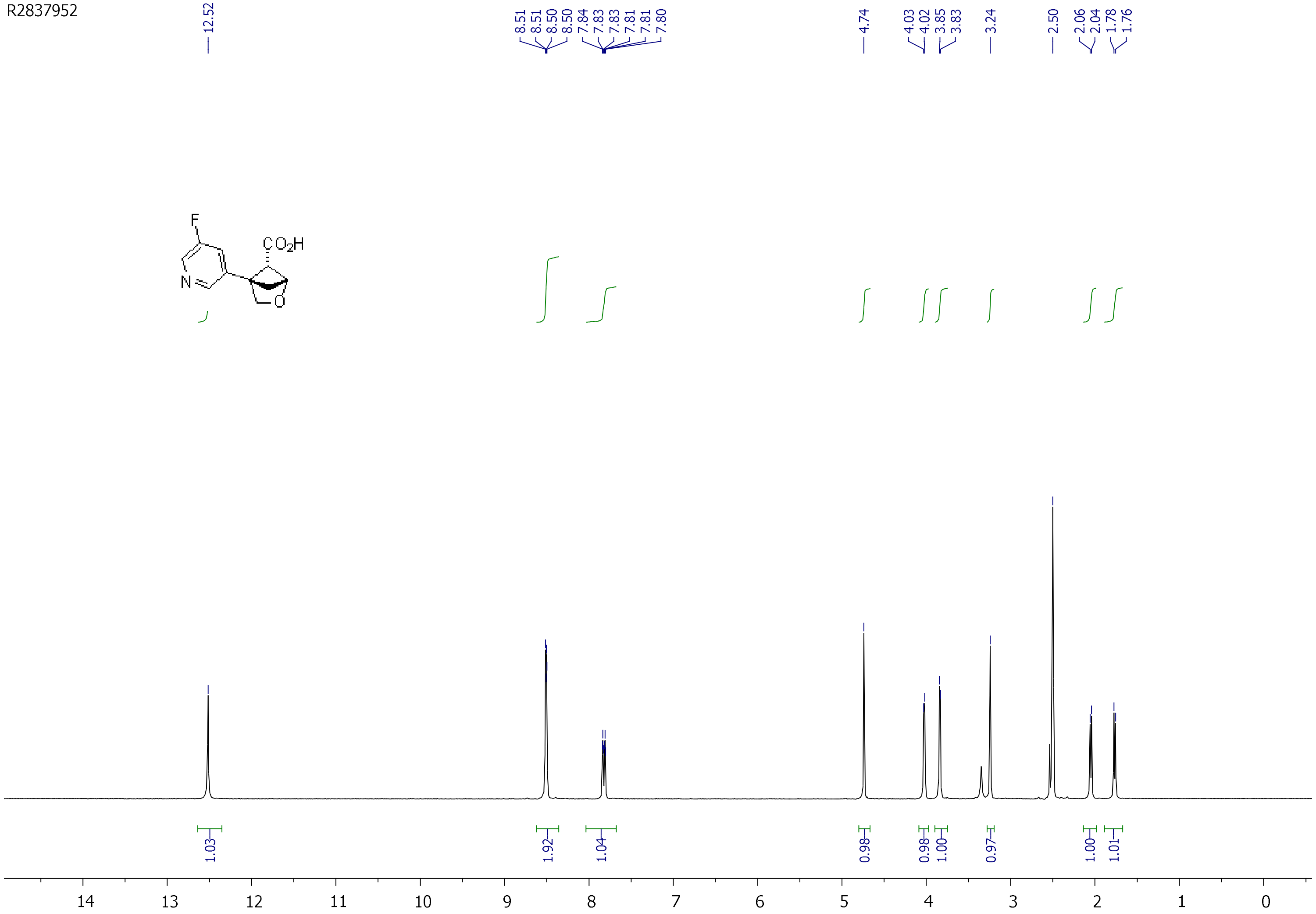

$^{13}C\{^{1}H\}$ NMR (126 MHz, DMSO-d_6)

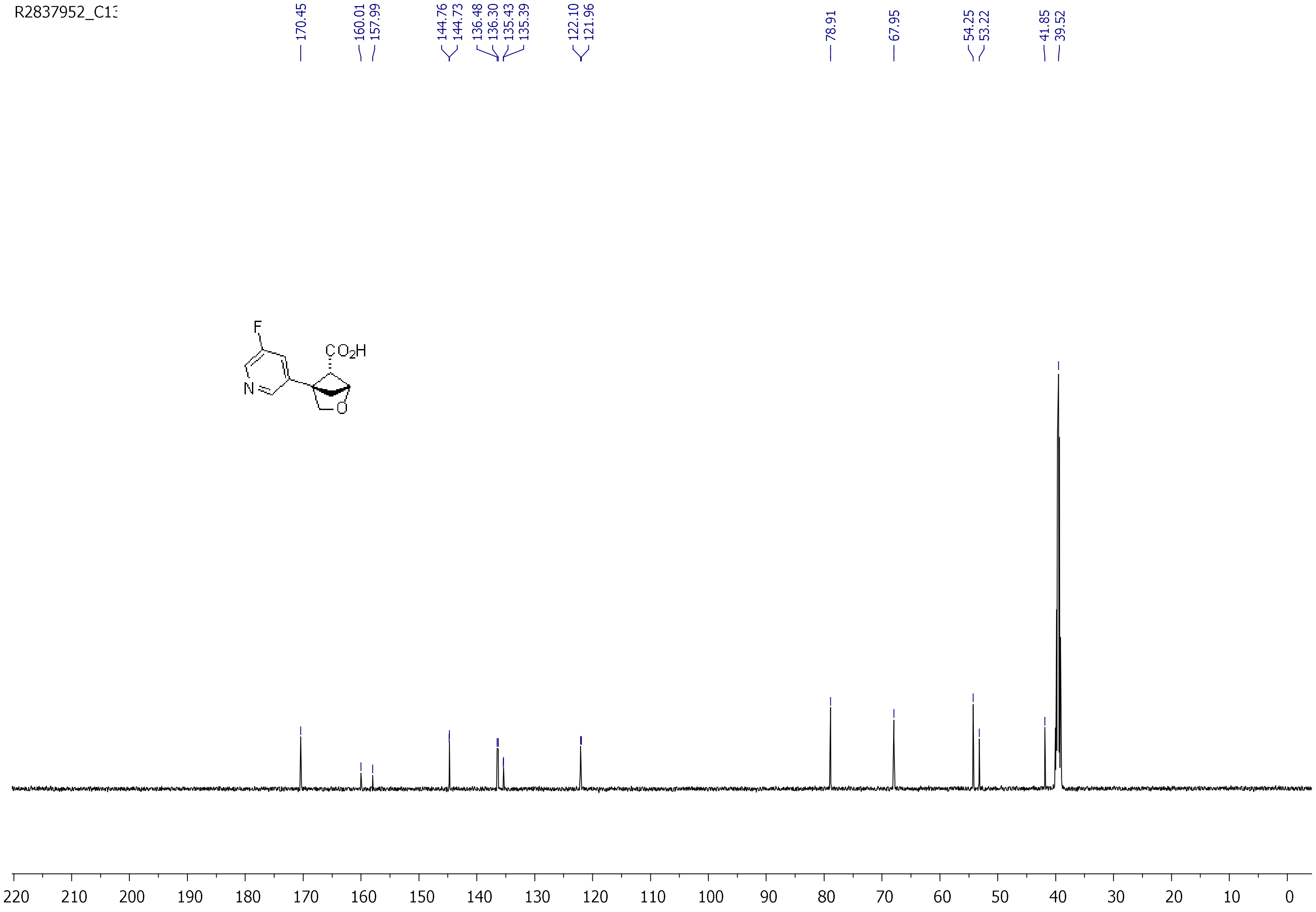

$^{19}F\{^{1}H\}$ NMR (376 MHz, DMSO-d_6)

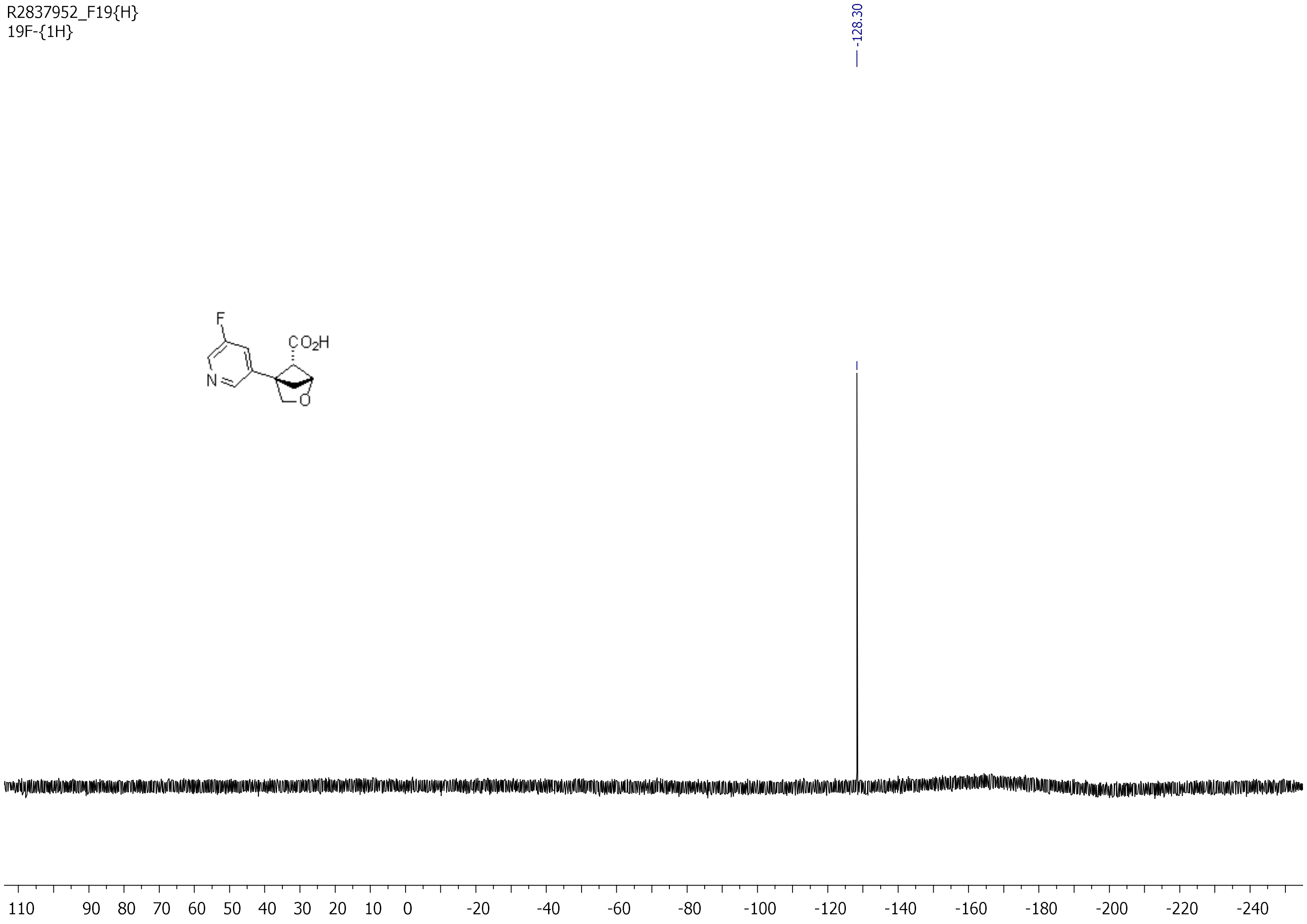

2-(6-Methoxypyridin-3-yl)prop-2-en-1-ol

^{1}H NMR (400 MHz, DMSO-d_6)

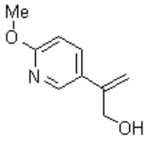

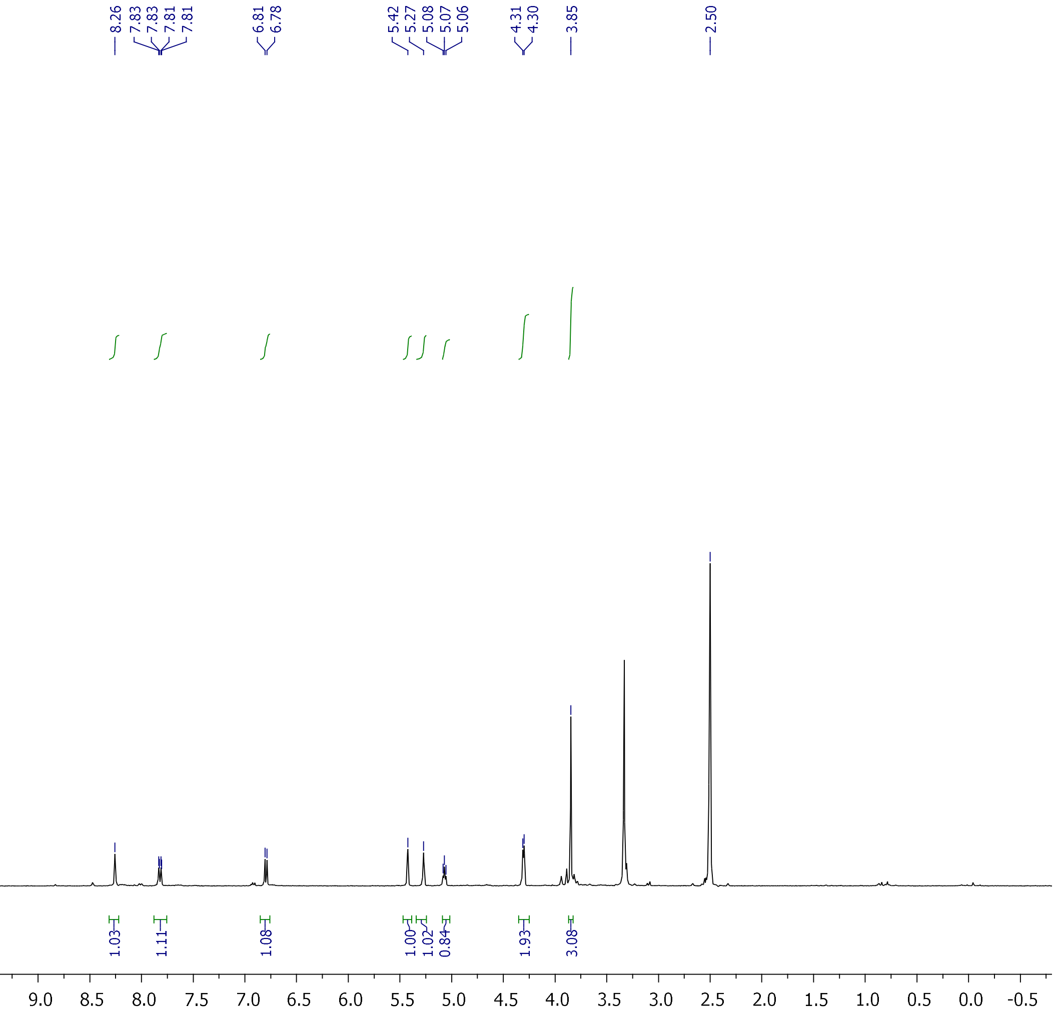

$^{13}C\{^1H\}$ NMR (126 MHz, $CDCl_3$)

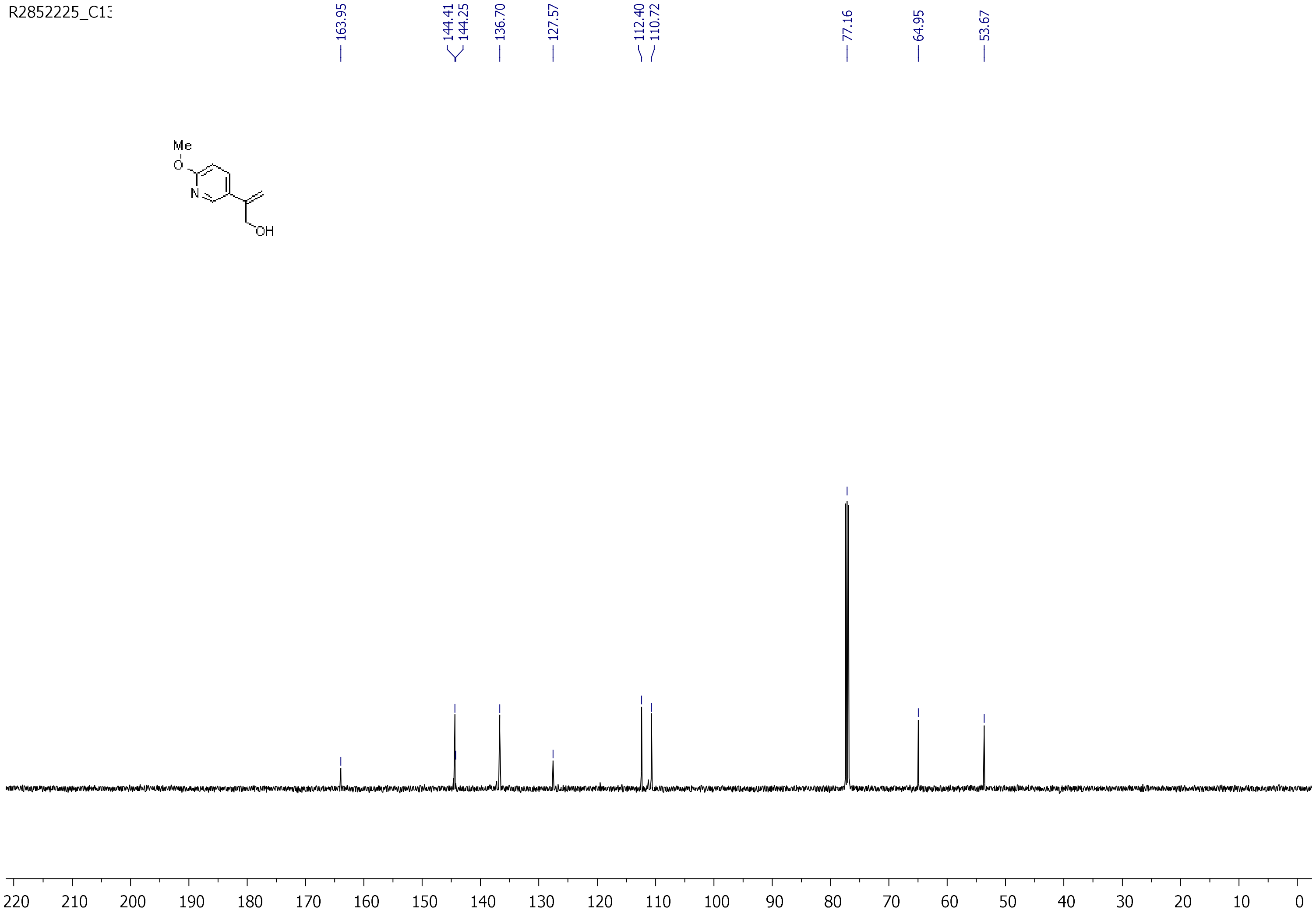

Compound (±)-24a

^{1}H NMR (400 MHz, $CDCl_3$)

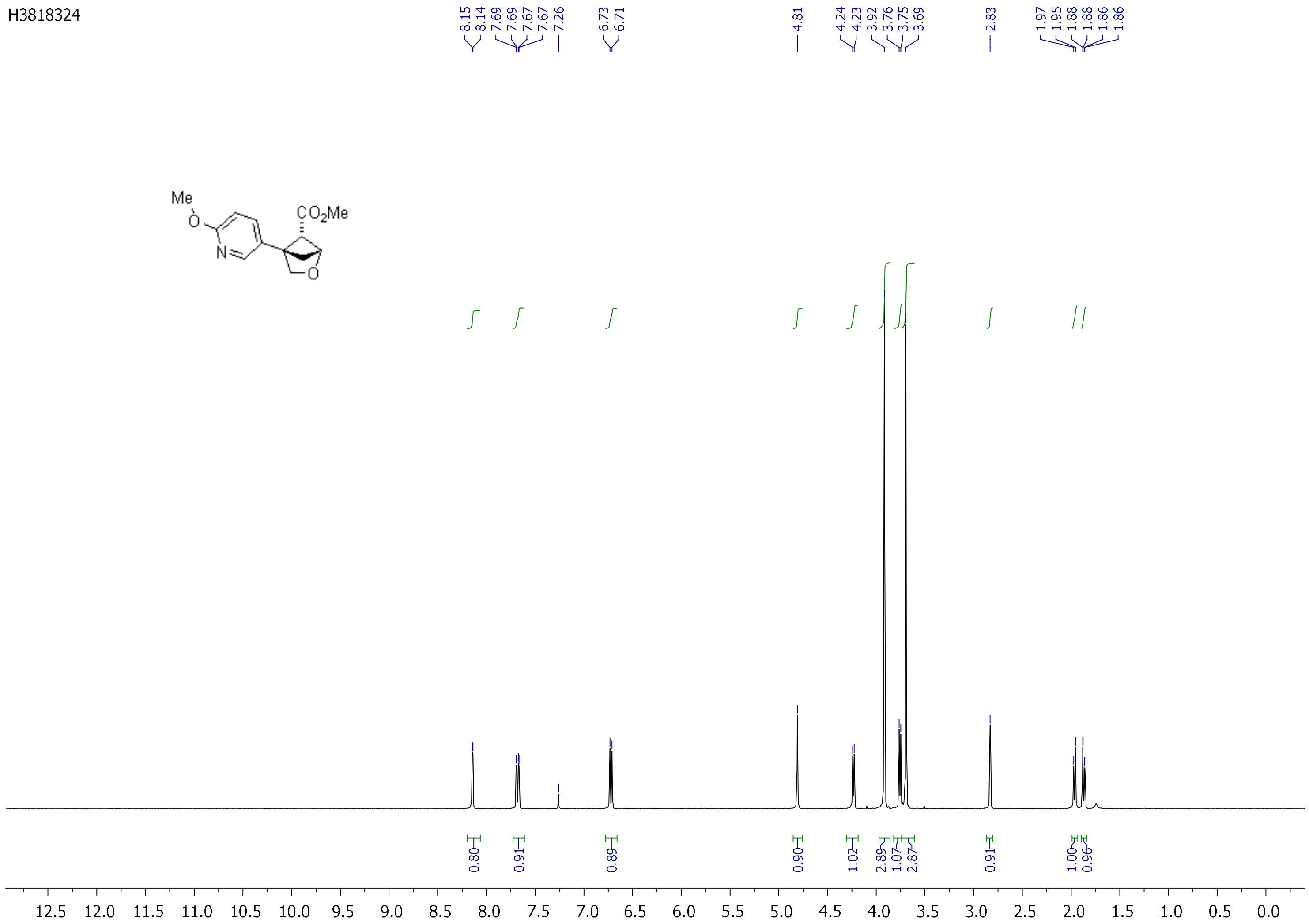

$^{13}C\{^{1}H\}$ NMR (126 MHz, $CDCl_3$)

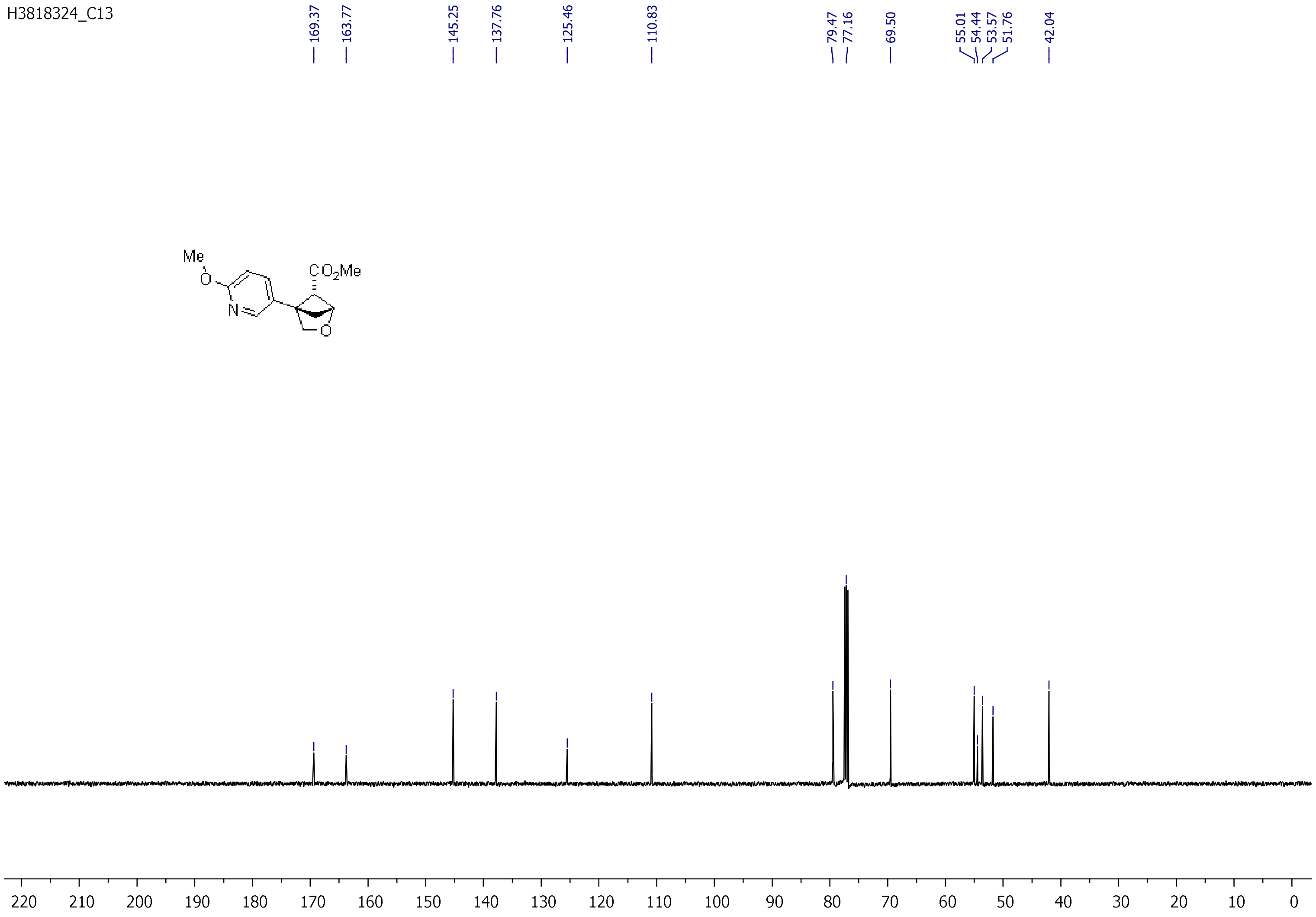

Compound (±)-24b

^{1}H NMR (400 MHz, DMSO-d_6)

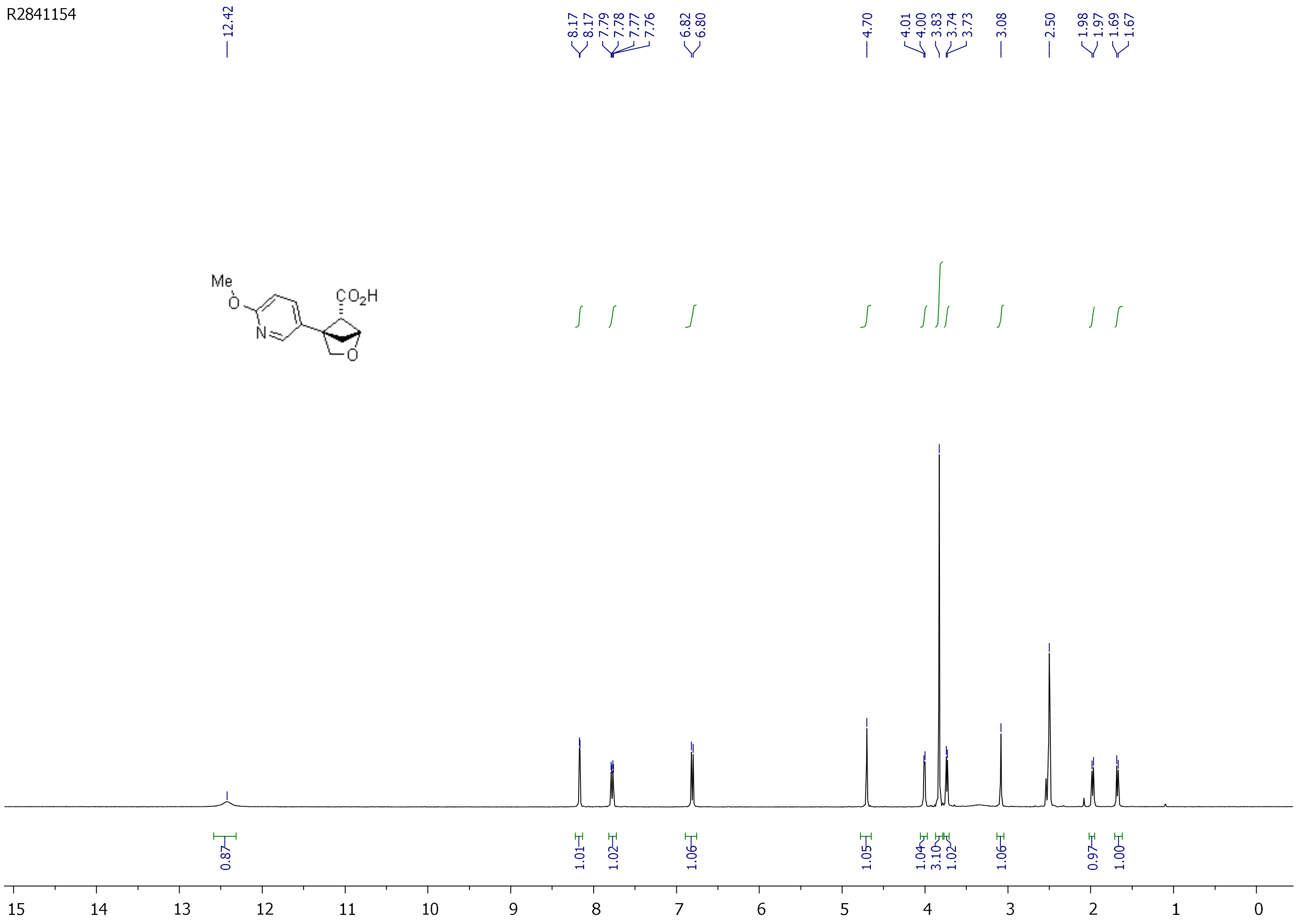

$^{13}C\{^1H\}$ NMR (126 MHz, DMSO-d_6)

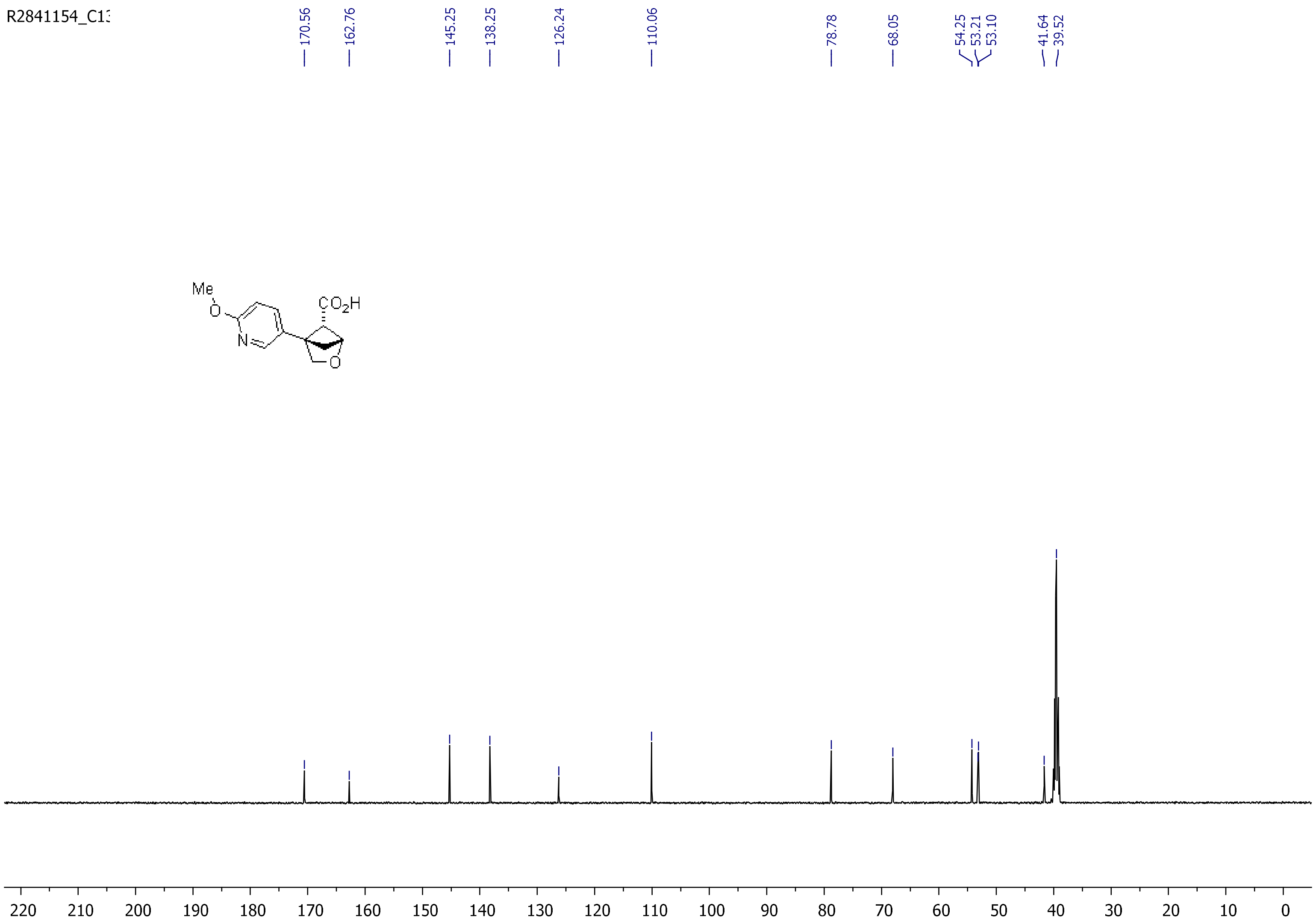

Compound (±)-28

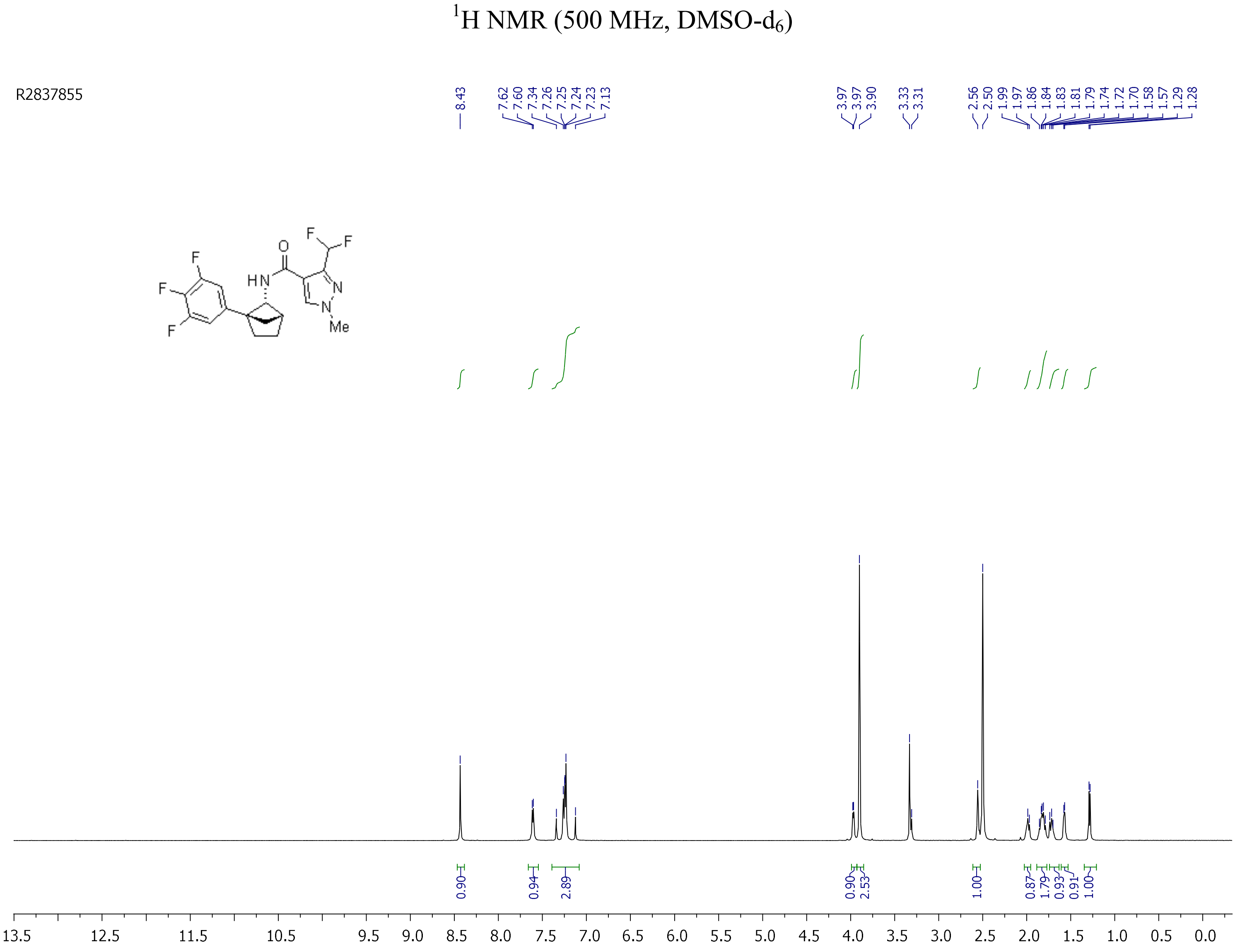

^{1}H NMR (500 MHz, DMSO-d$_6$)
R2837855
8.43
7.62
7.60
7.34
7.26
7.25
7.24
7.23
7.13
3.97
3.97
3.90
3.33
3.31
2.56
2.50
1.99
1.97
1.86
1.84
1.83
1.81
1.79
1.74
1.72
1.70
1.58
1.57
1.29
1.28
0.90
0.94
2.89
0.90
2.53
1.00
0.87
1.79
0.93
0.91
1.00
13.5
12.5
11.5
10.5
9.5
9.0
8.5
8.0
7.5
7.0
6.5
6.0
5.5
5.0
4.5
4.0
3.5
3.0
2.5
2.0
1.5
1.0
0.5
0.0

$^{13}C\{^{1}H\}$ NMR (126 MHz, DMSO-d_6)

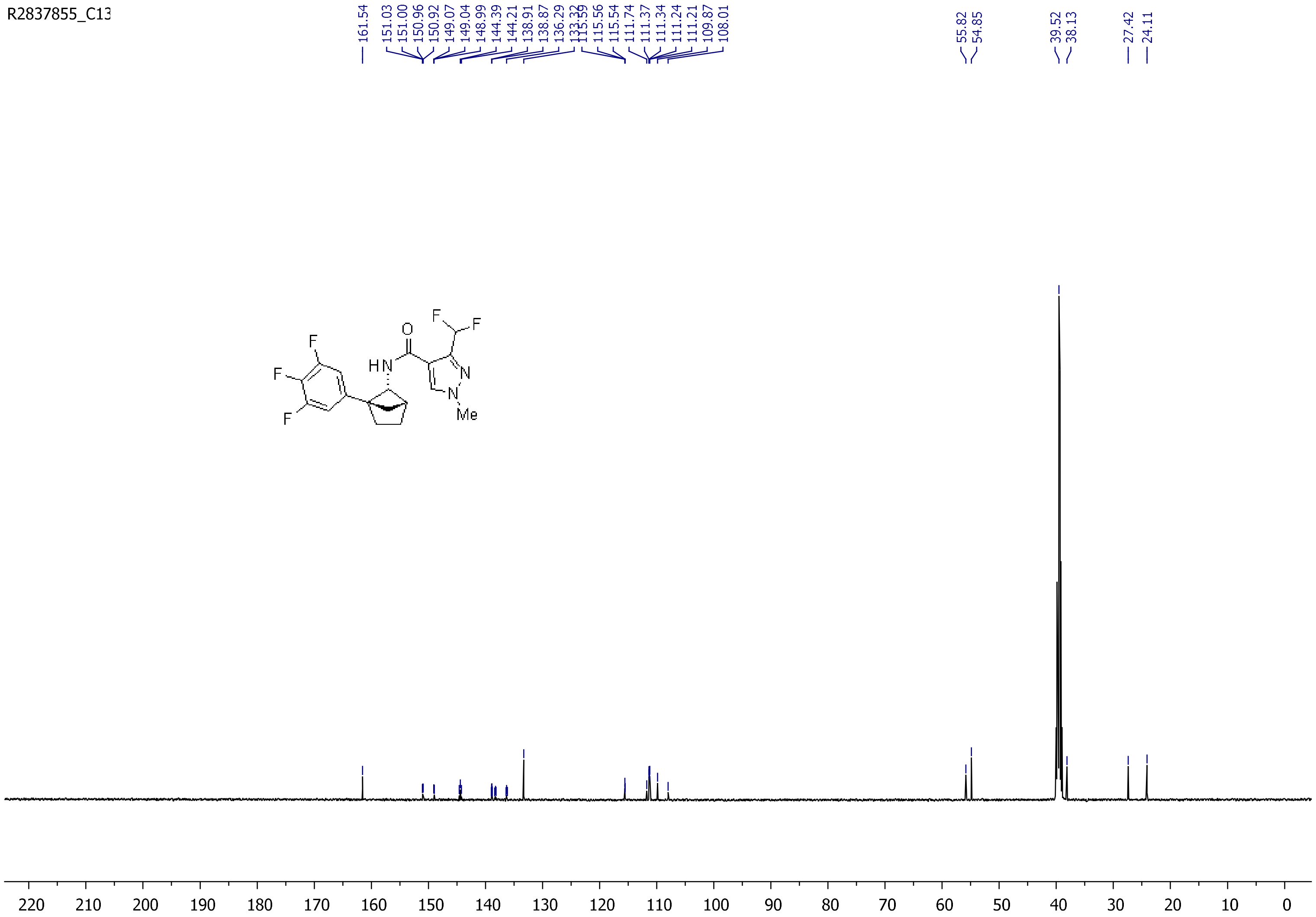

$^{19}F\{^1H\}$ NMR (376 MHz, DMSO-d$_6$)

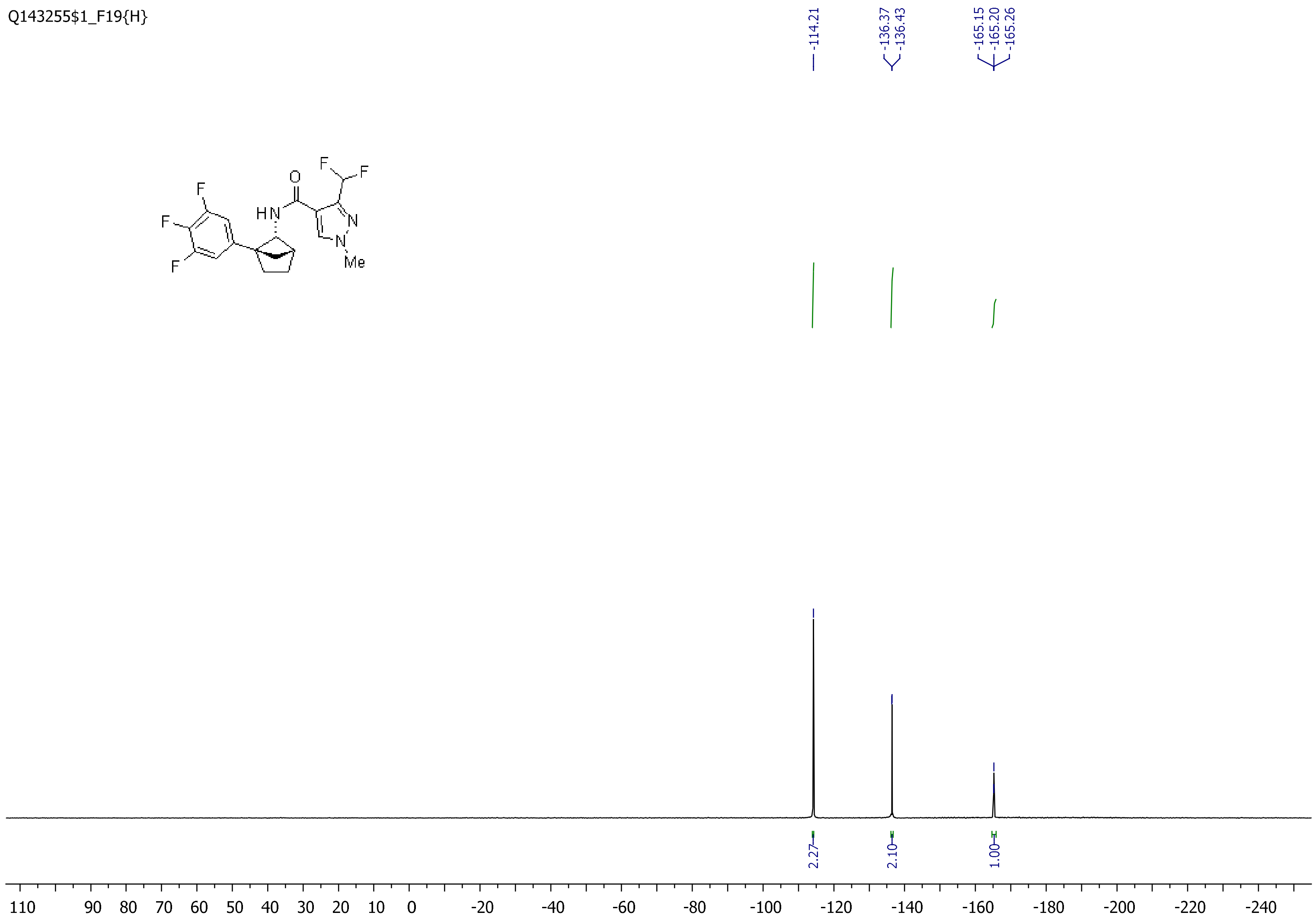

Compound (±)-29

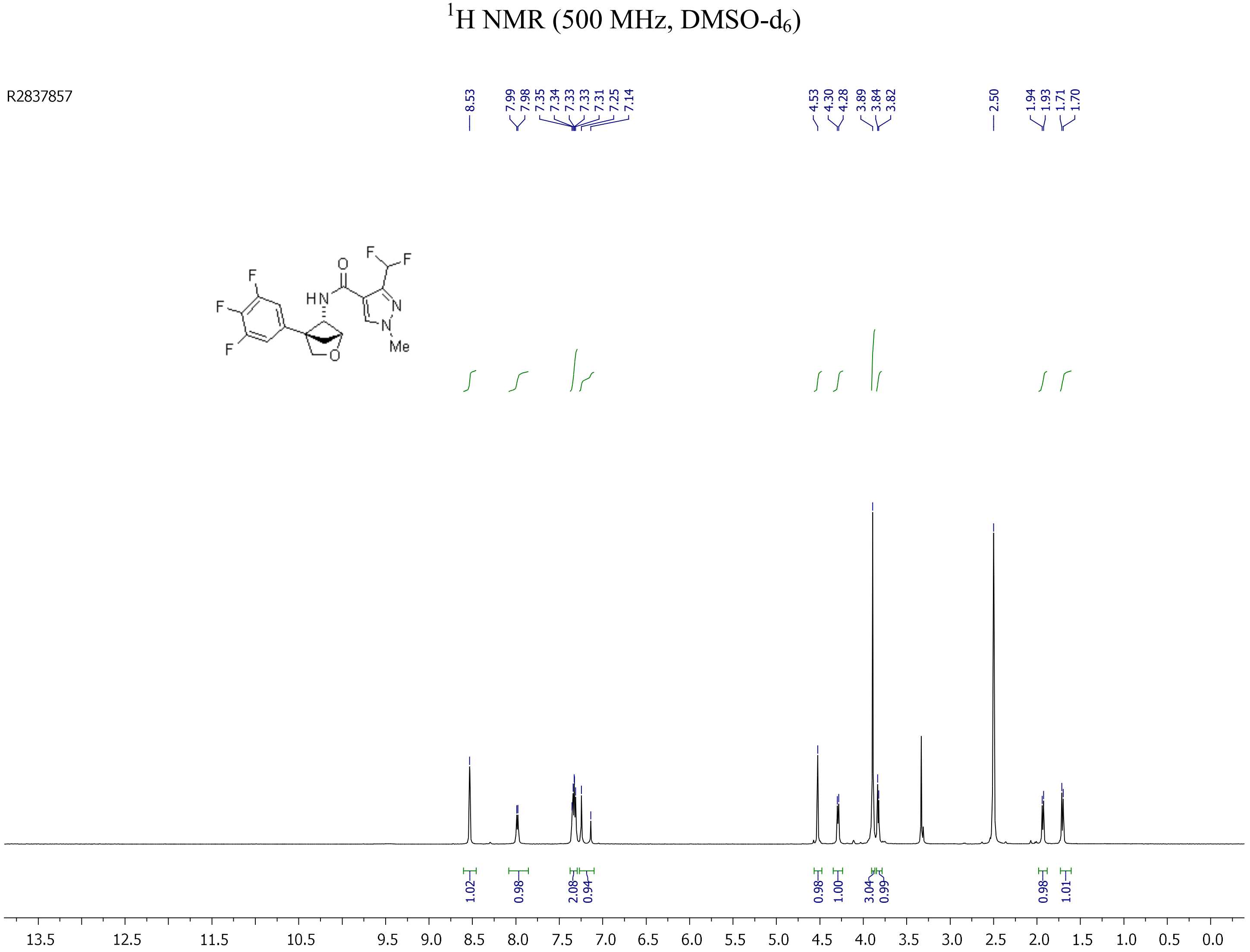

1H NMR (500 MHz, DMSO-d6)
R2837857
8.53
7.99
7.98
7.35
7.34
7.33
7.33
7.31
7.25
7.14
4.53
4.30
4.28
3.89
3.84
3.82
2.50
1.94
1.93
1.71
1.70
1.02
0.98
2.08
0.94
0.98
1.00
3.04
0.99
0.98
1.01
13.5
12.5
11.5
10.5
9.5
9.0
8.5
8.0
7.5
7.0
6.5
6.0
5.5
5.0
4.5
4.0
3.5
3.0
2.5
2.0
1.5
1.0
0.5
0.0

$^{13}C\{^{1}H\}$ NMR (151 MHz, DMSO-d_6)

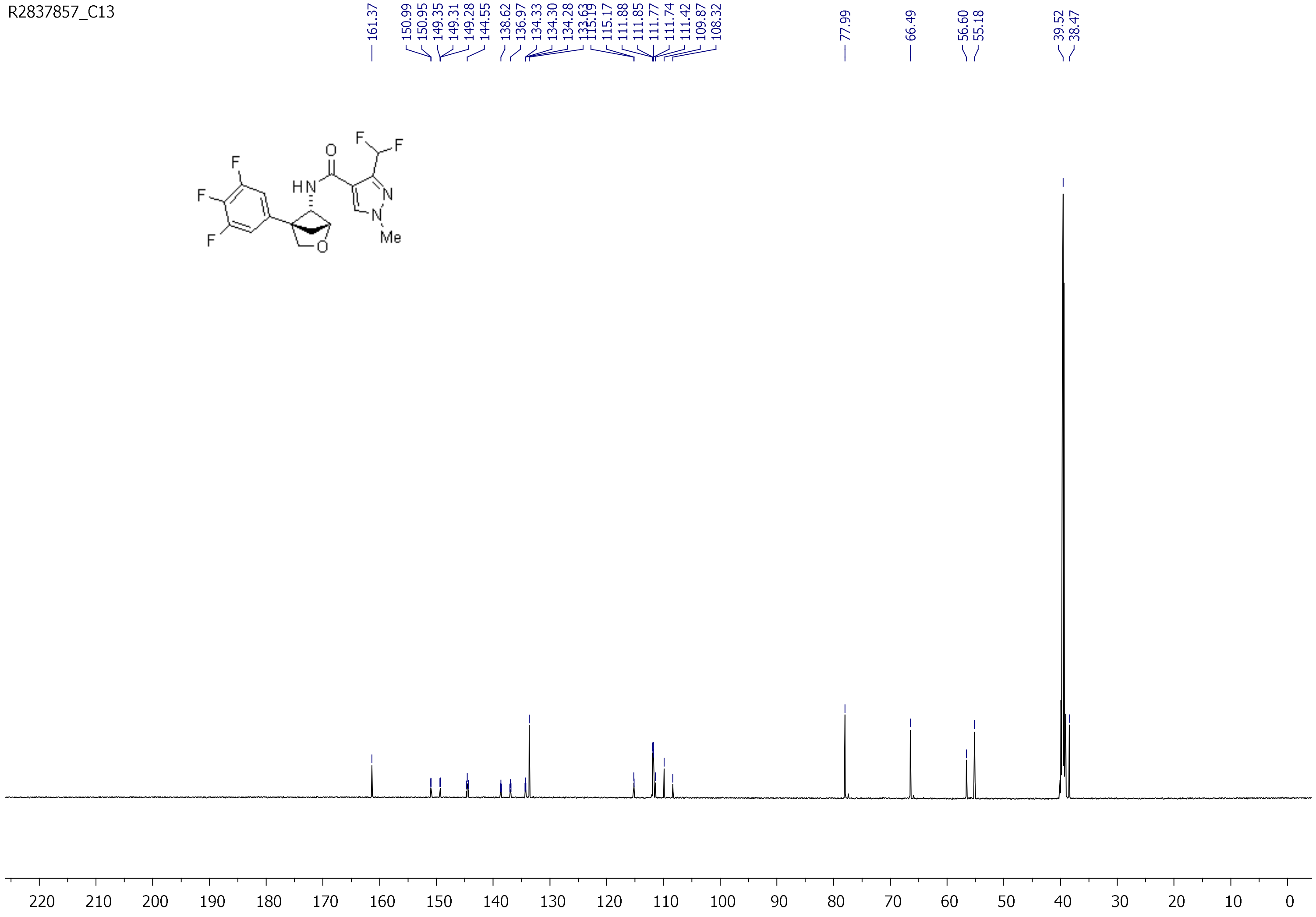

$^{19}F\{^{1}H\}$ NMR (376 MHz, DMSO-d_6)

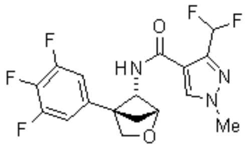

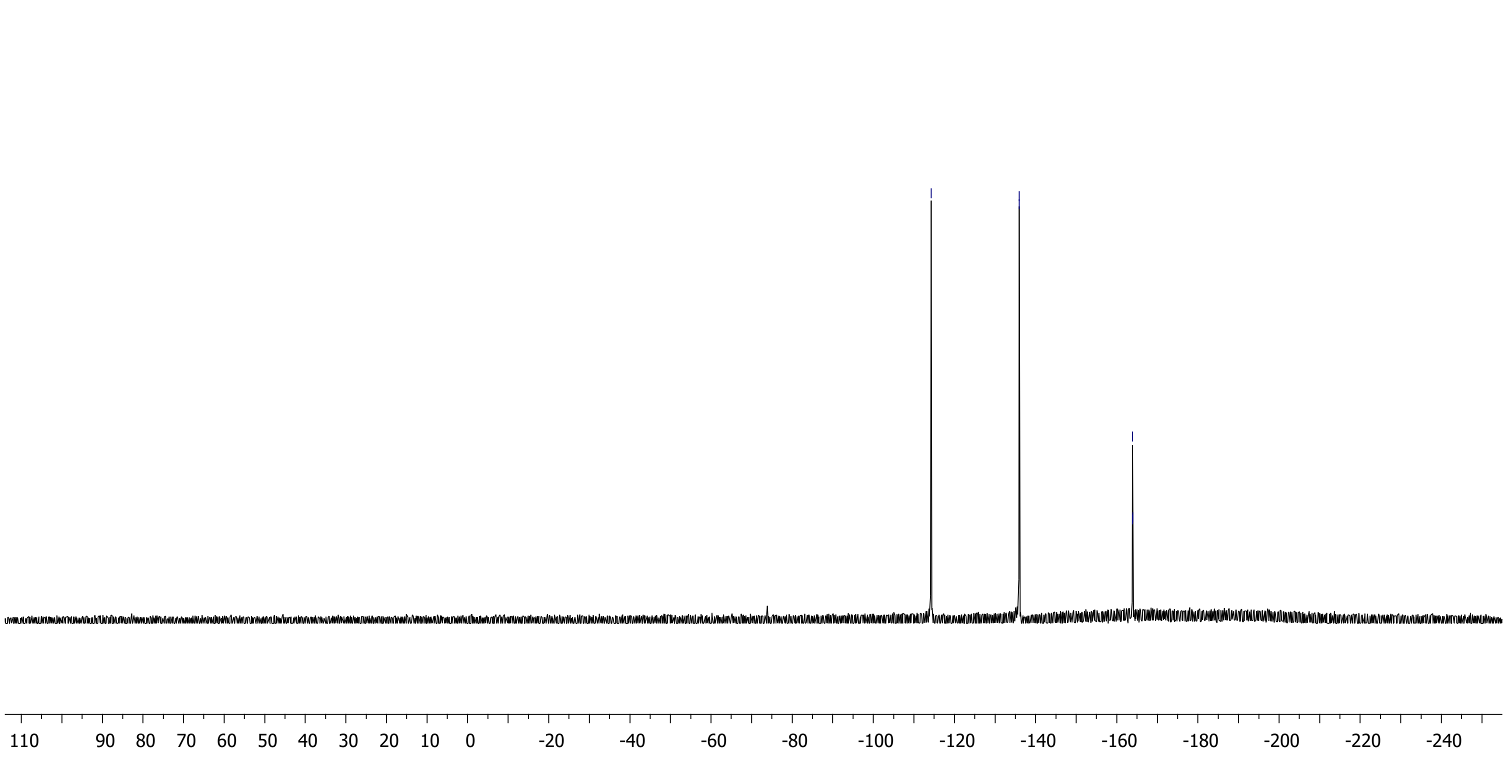

Compound (±)-31

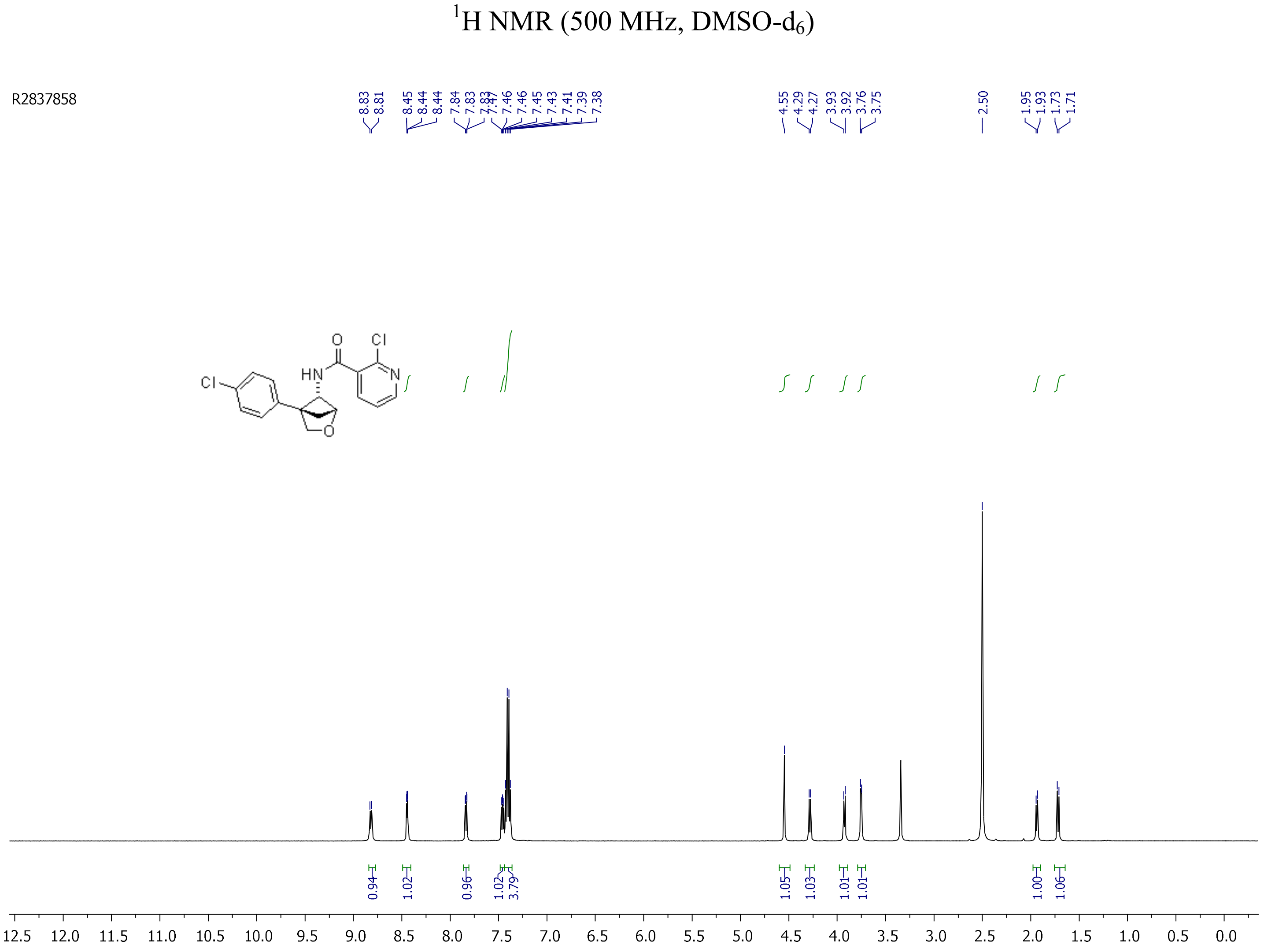

¹H NMR (500 MHz, DMSO-d6)
R2837858
8.83
8.81
8.45
8.44
8.44
7.84
7.83
7.83
7.47
7.46
7.46
7.45
7.43
7.41
7.39
7.38
4.55
4.29
4.27
3.93
3.92
3.76
3.75
2.50
1.95
1.93
1.73
1.71
0.94
1.02
0.96
1.02
3.79
1.05
1.03
1.01
1.01
1.00
1.06
12.5 12.0 11.5 11.0 10.5 10.0 9.5 9.0 8.5 8.0 7.5 7.0 6.5 6.0 5.5 5.0 4.5 4.0 3.5 3.0 2.5 2.0 1.5 1.0 0.5 0.0

$^{13}C\{^1H\}$ NMR (126 MHz, DMSO-d_6)

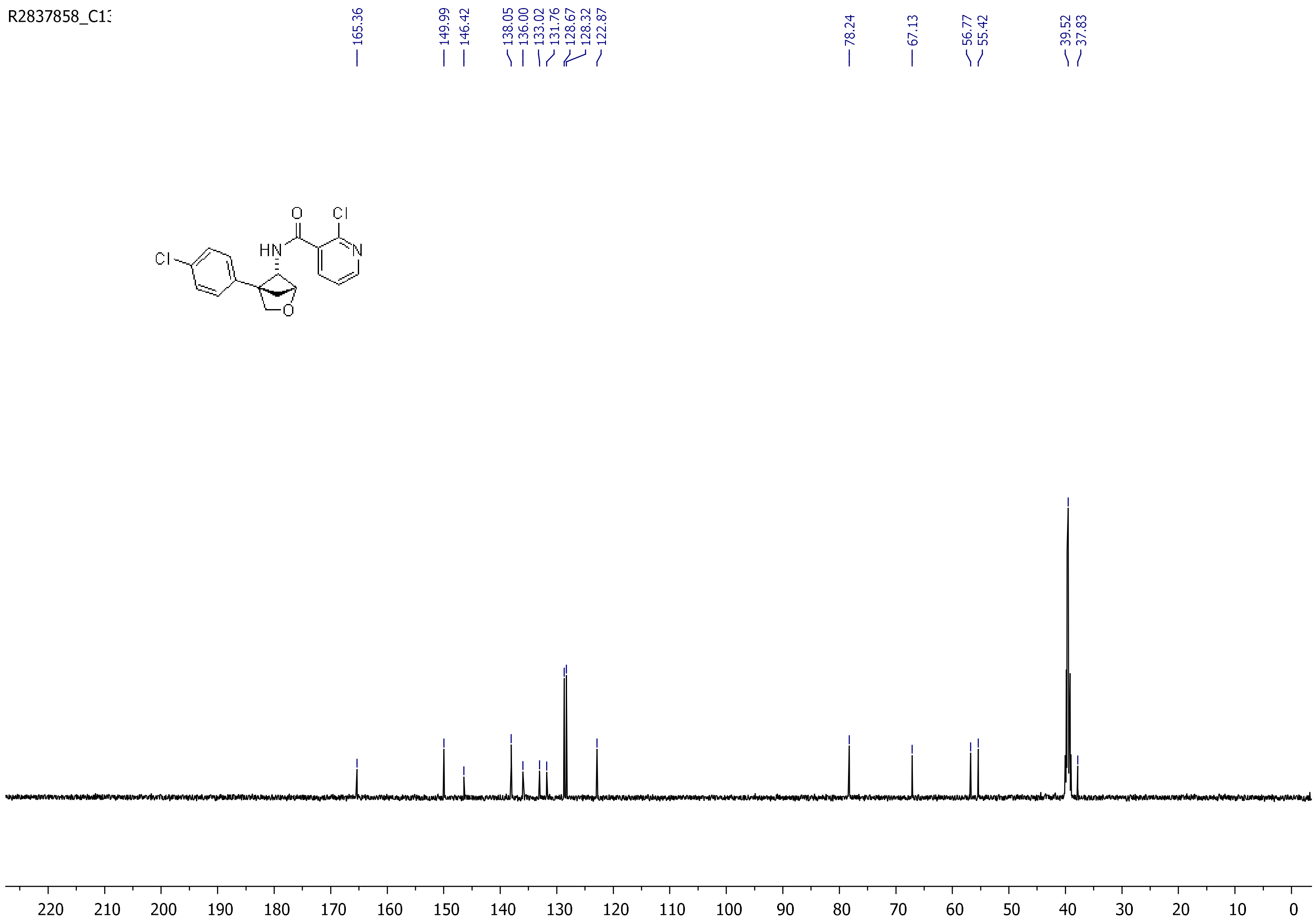

Compound (±)-30

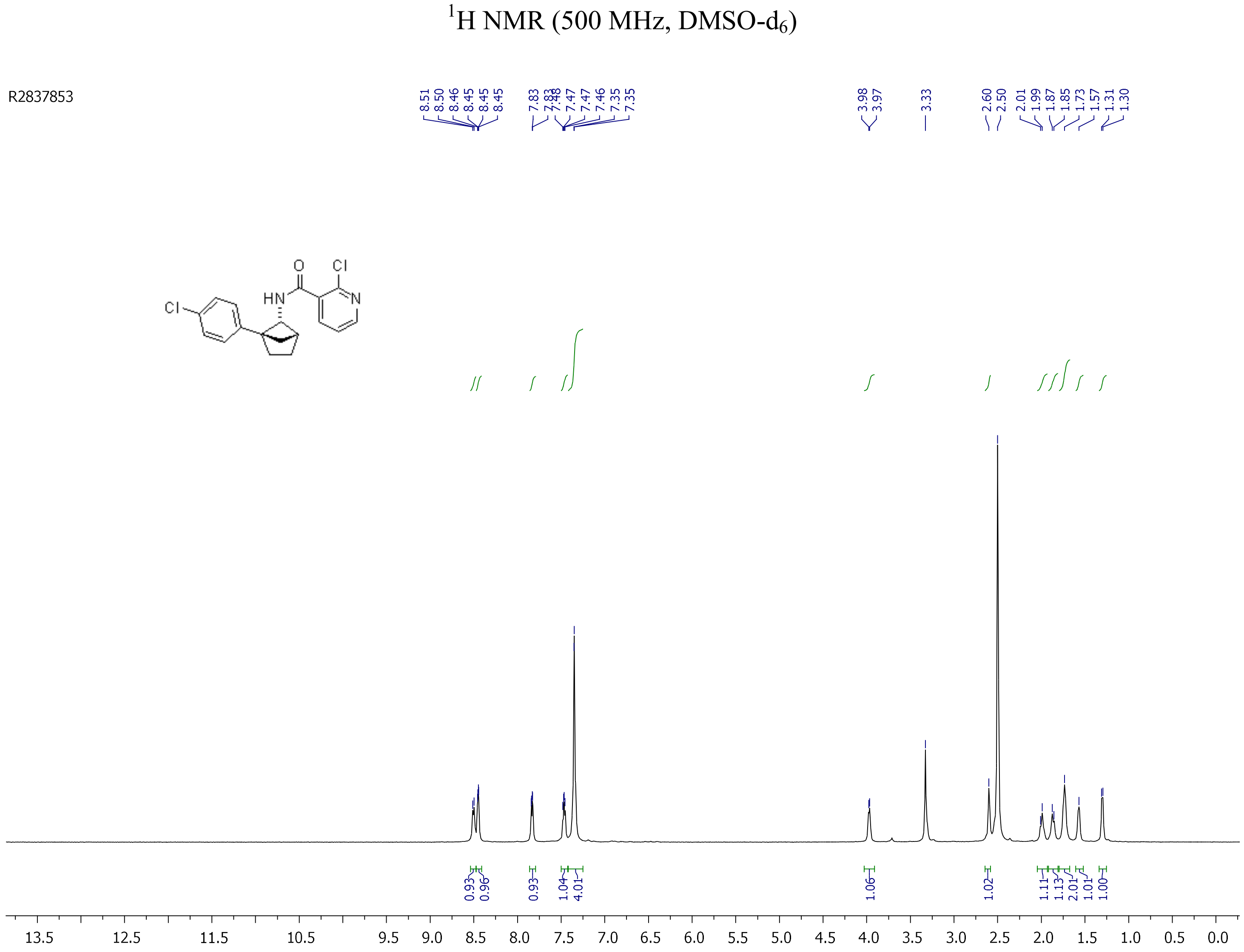

¹H NMR (500 MHz, DMSO-d6)
R2837853
8.51 8.50 8.46 8.45 8.45 8.45
7.83 7.83 7.48 7.47 7.47 7.46 7.35 7.35
3.98 3.97
3.33
2.60 2.50
2.01 1.99 1.87 1.85 1.73 1.57 1.31 1.30
O
Cl
HN
N
Cl
0.93 0.96 0.93 1.04 4.01 1.06 1.02 1.11 1.13 2.01 1.01 1.00
13.5 12.5 11.5 10.5 9.5 9.0 8.5 8.0 7.5 7.0 6.5 6.0 5.5 5.0 4.5 4.0 3.5 3.0 2.5 2.0 1.5 1.0 0.5 0.0

$^{13}C\{^{1}H\}$ NMR (126 MHz, DMSO-d_6)

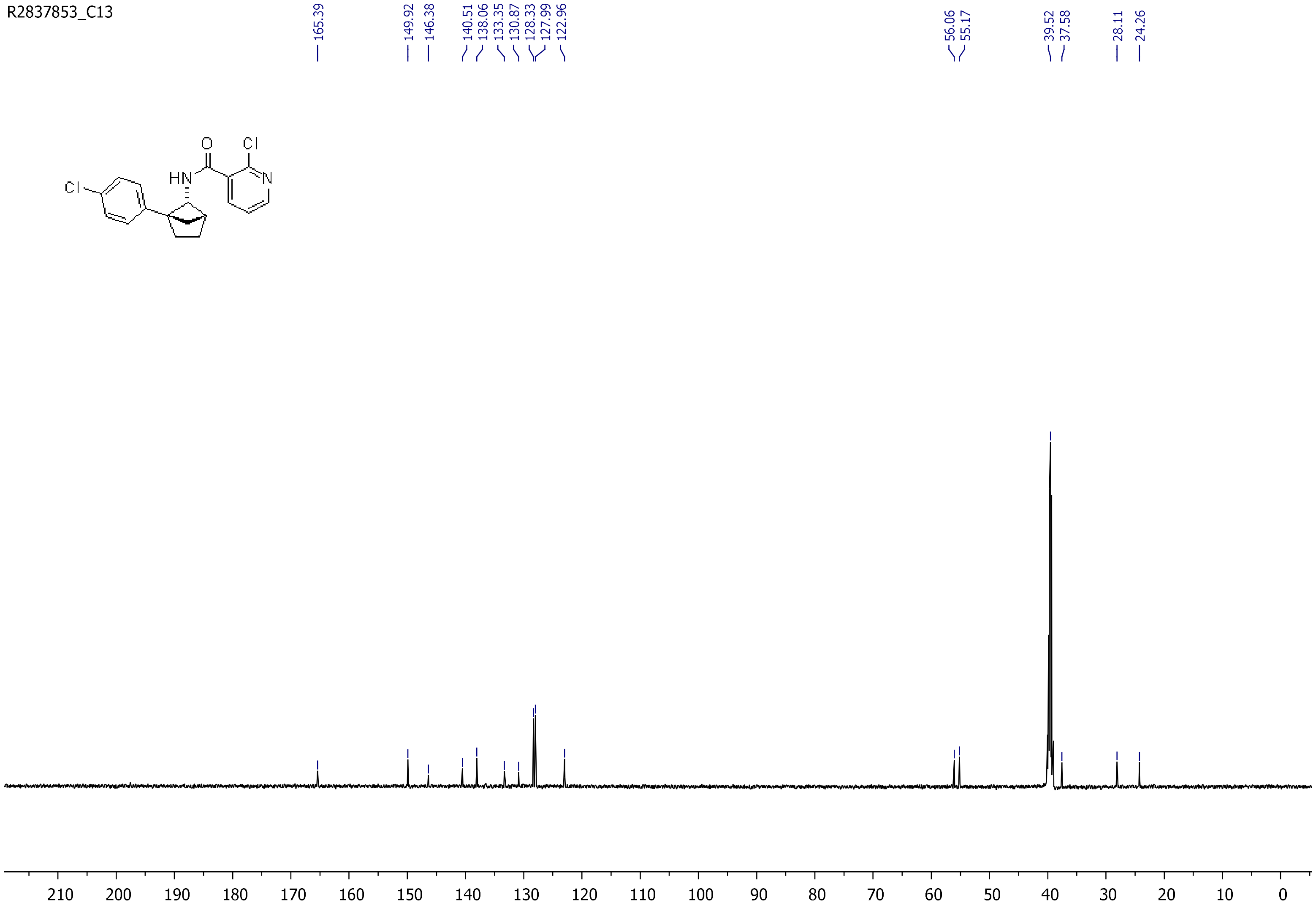

(±)-5-(Methoxycarbonyl)-2-oxabicyclo[2.1.1]hexane-4-carboxylic acid, (±)-S3

^{1}H NMR (400 MHz, $CDCl_3$)

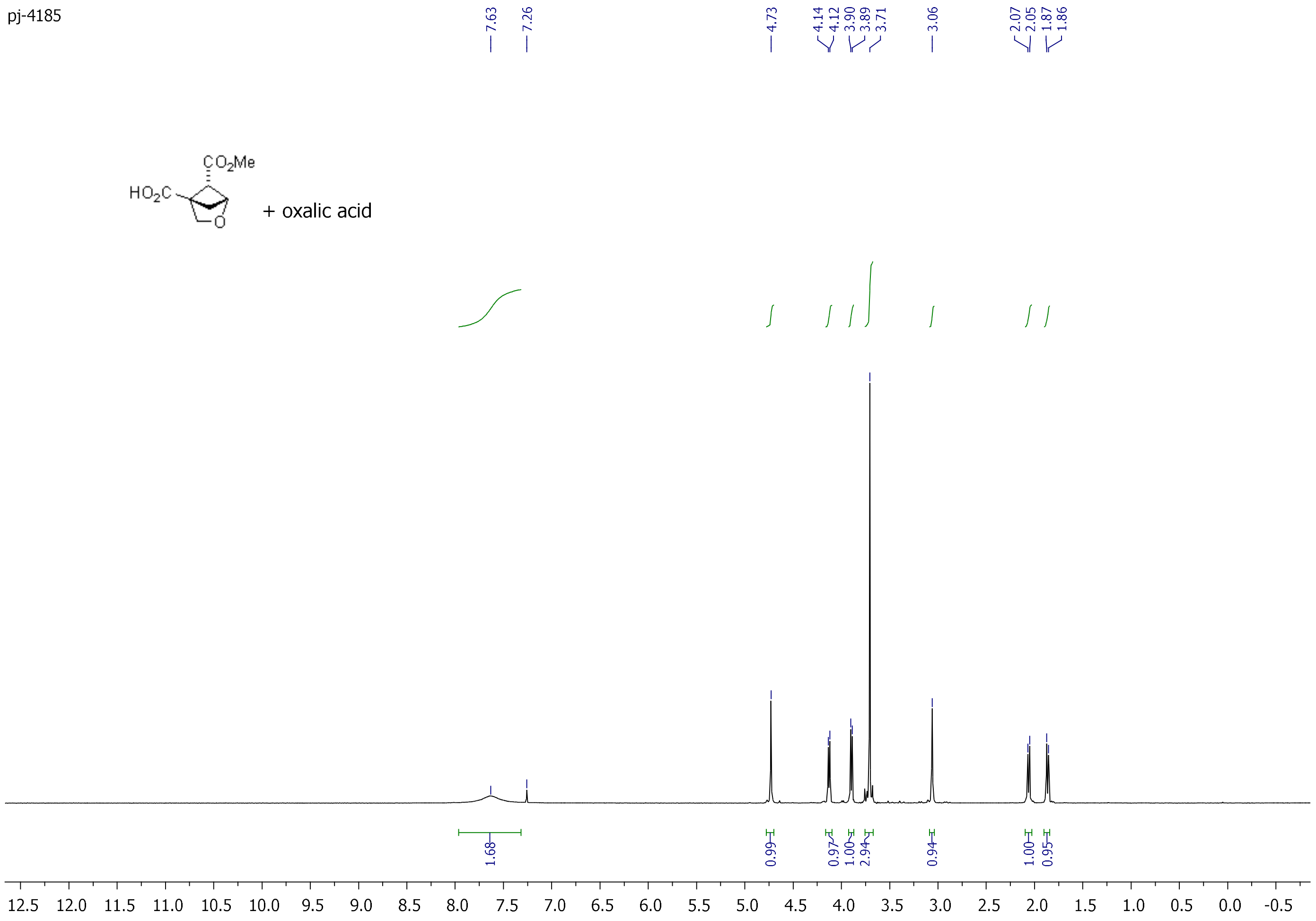

$^{13}C\{^{1}H\}$ NMR (126 MHz, $CDCl_3$)

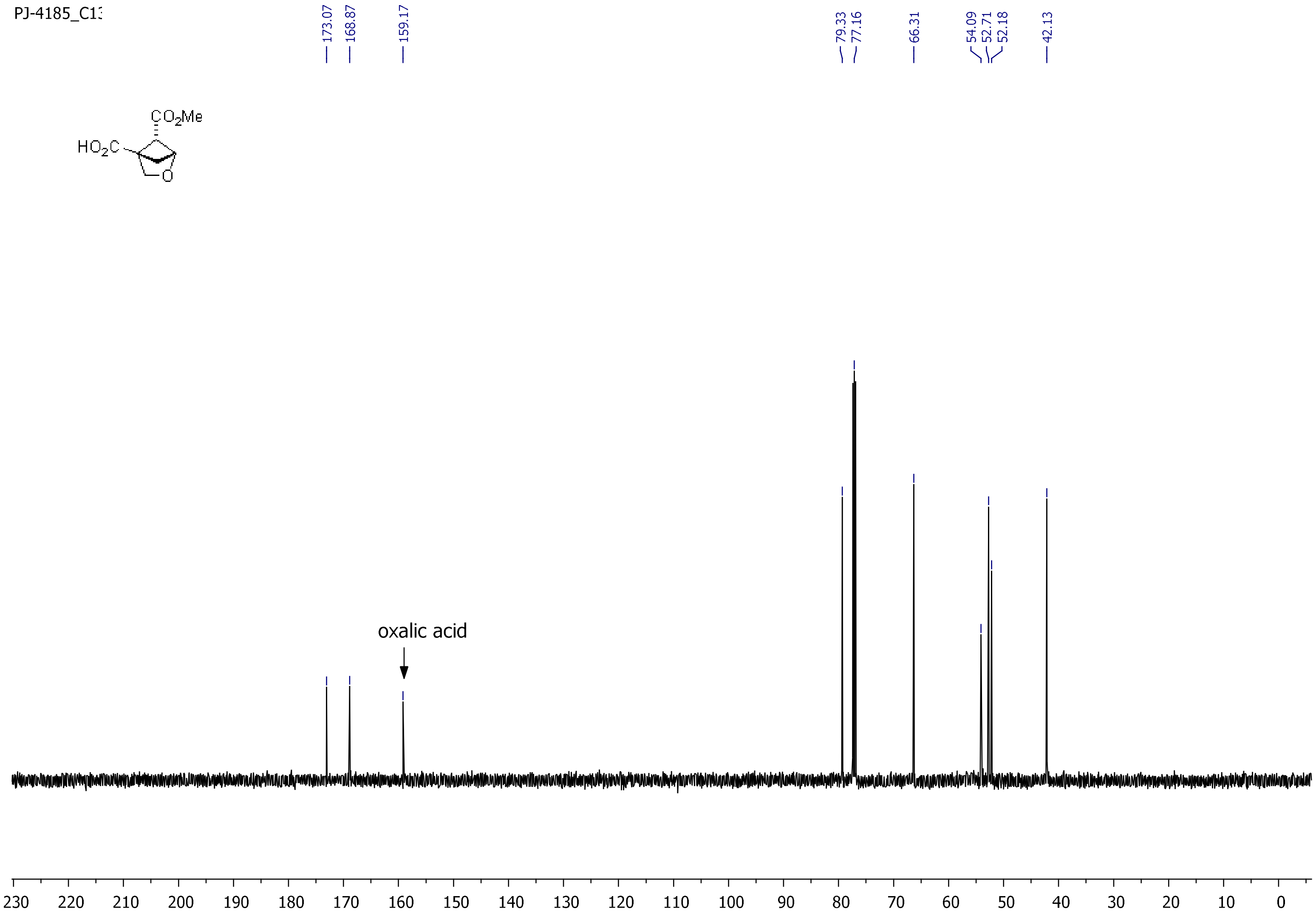

Compound (±)-33

^{1}H NMR (500 MHz, DMSO-d$_6$)

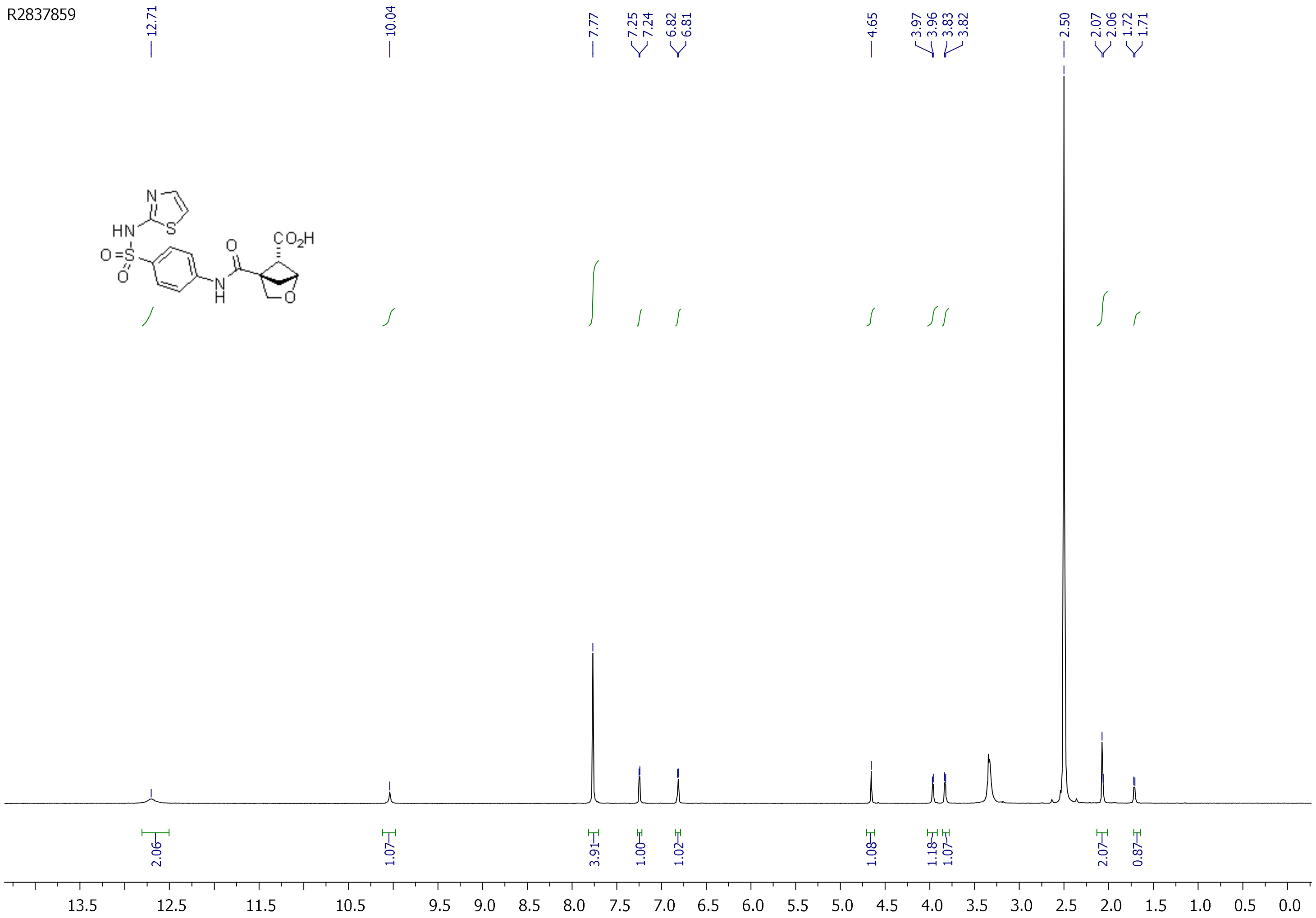

$^{13}C\{^1H\}$ NMR (126 MHz, DMSO-d$_6$)

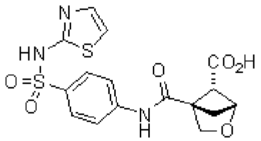

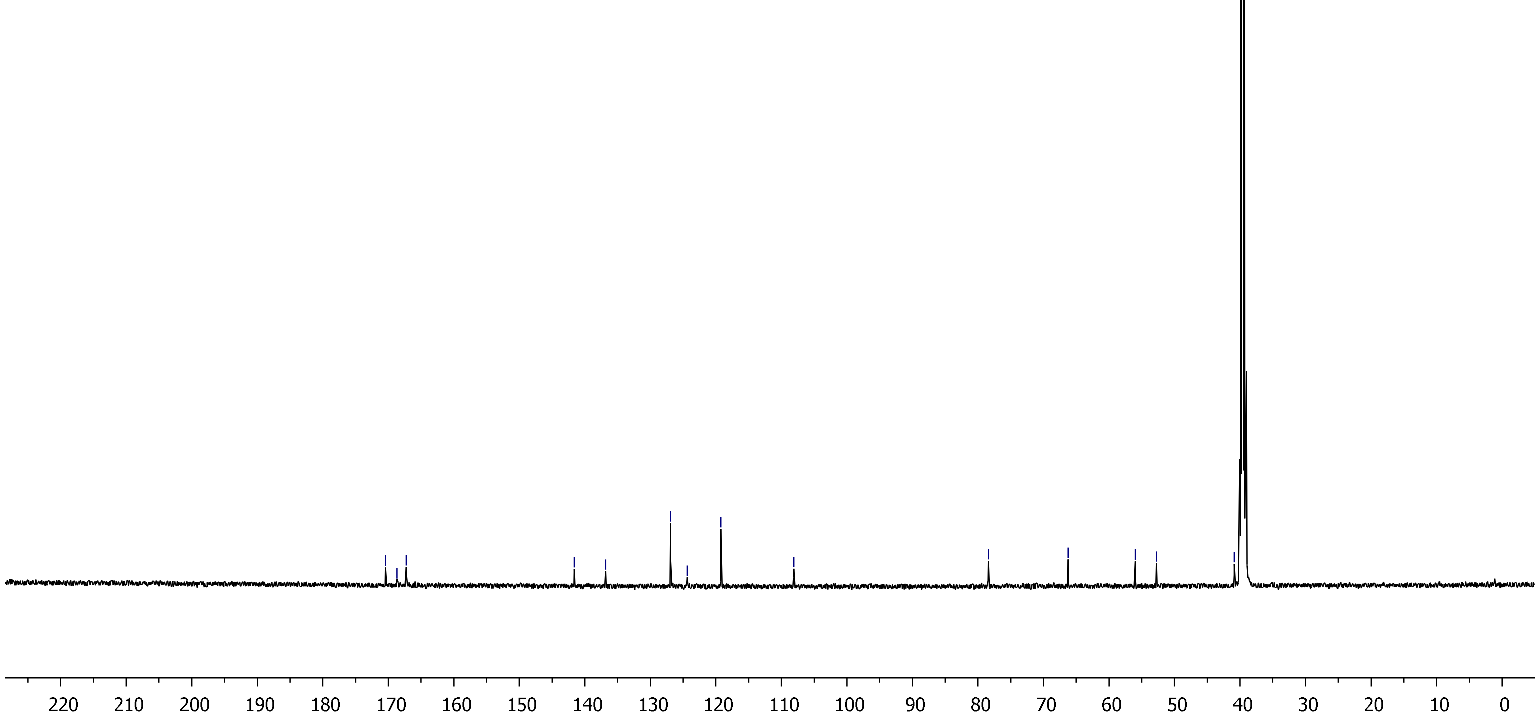

5-(Methoxycarbonyl)bicyclo[2.1.1]hexane-1-carboxylic acid, (±)-S5

^{1}H NMR (400 MHz, $CDCl_3$)

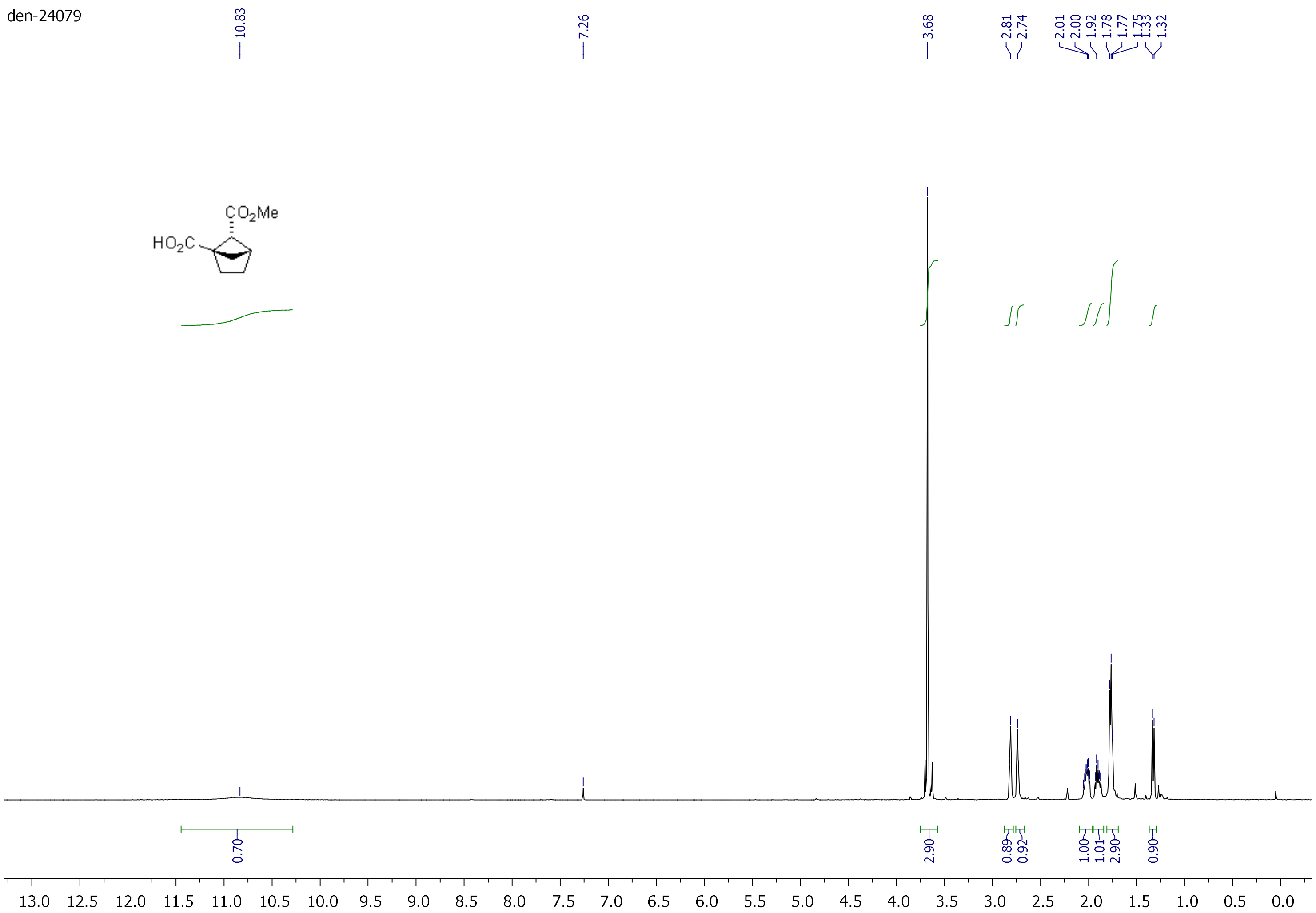

$^{13}C\{^1H\}$ NMR (151 MHz, $CDCl_3$)

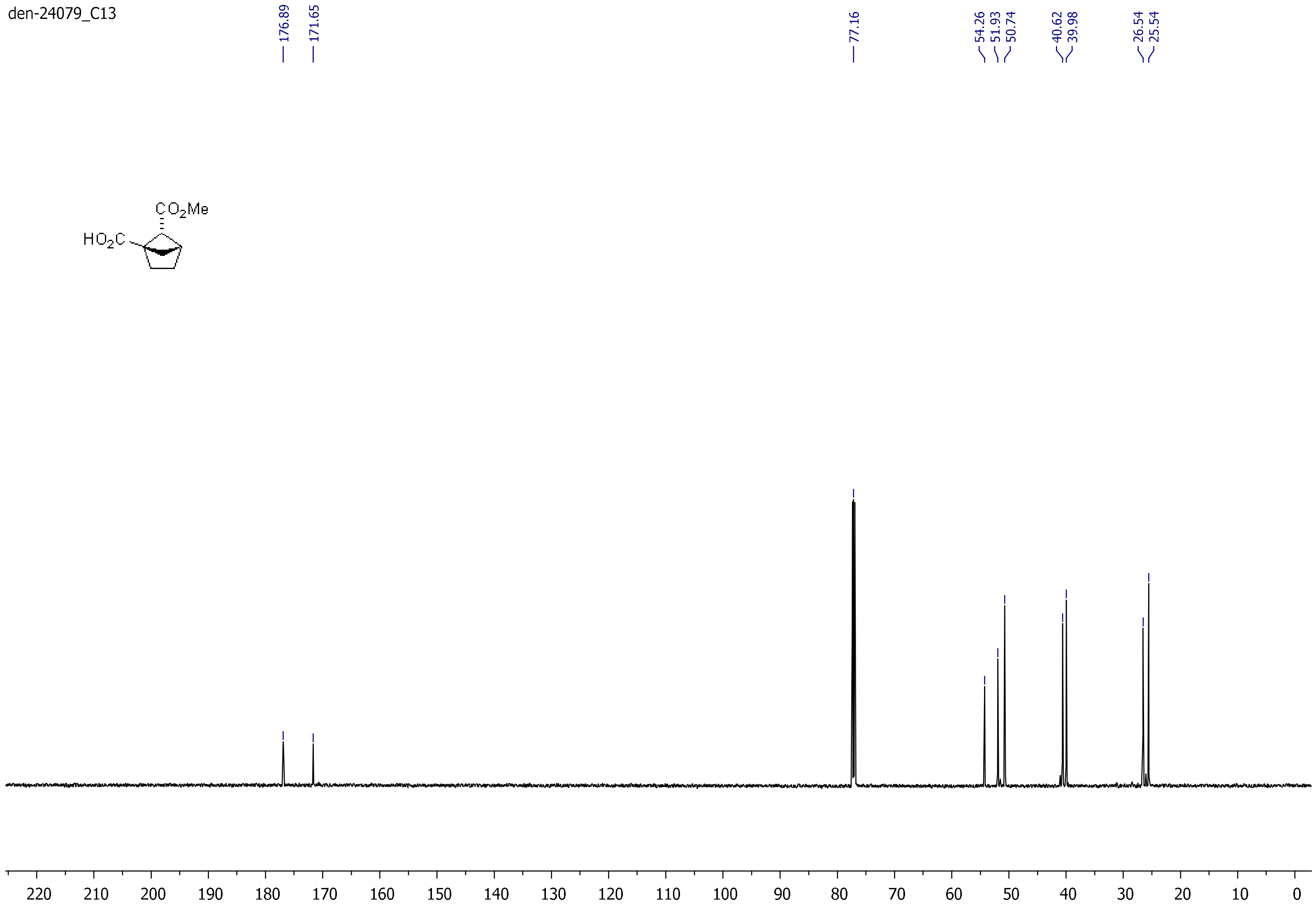

Compound (±)-32

^1H NMR (500 MHz, DMSO-d_6)

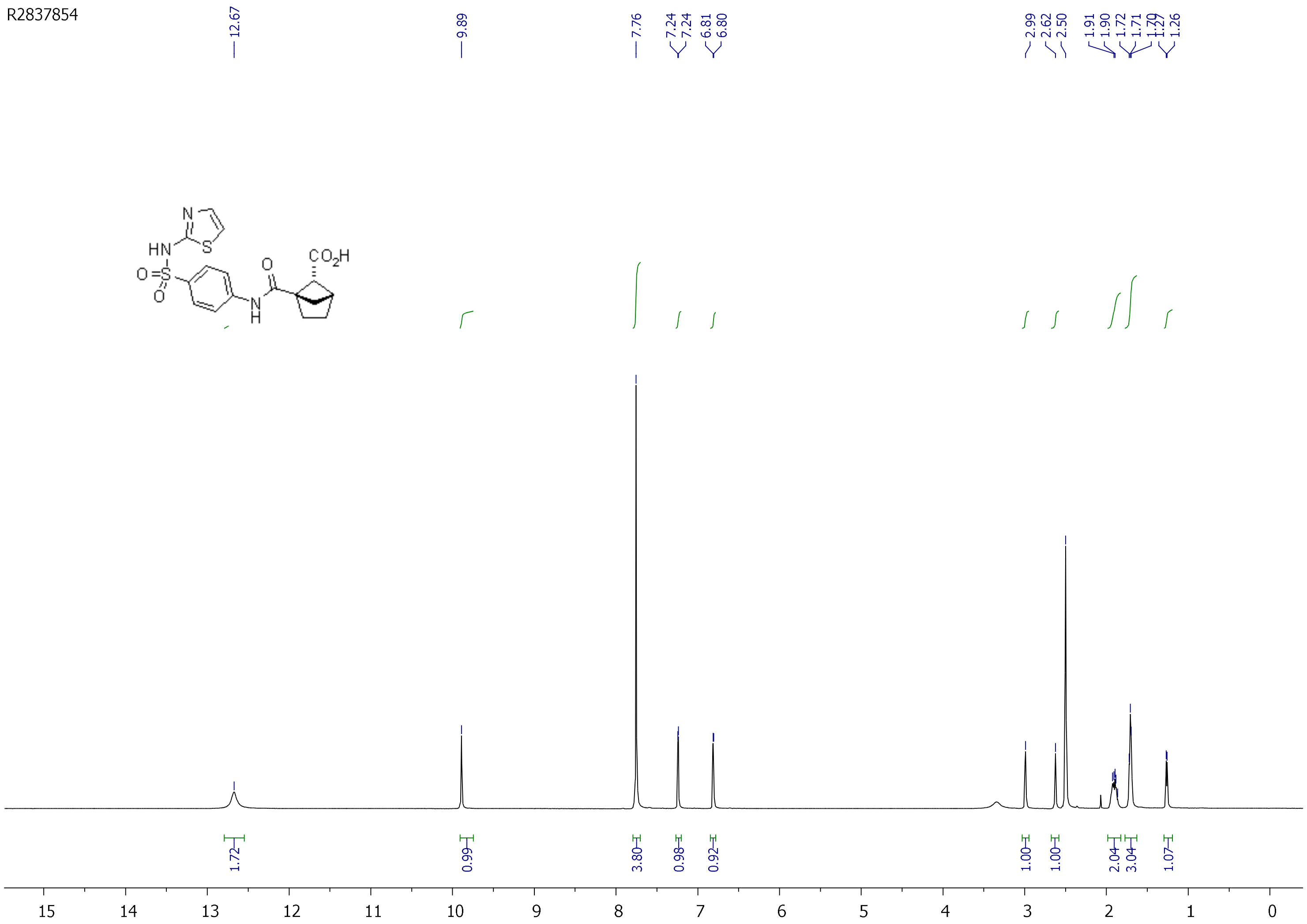

$^{13}C\{^{1}H\}$ NMR (126 MHz, DMSO-d_6)

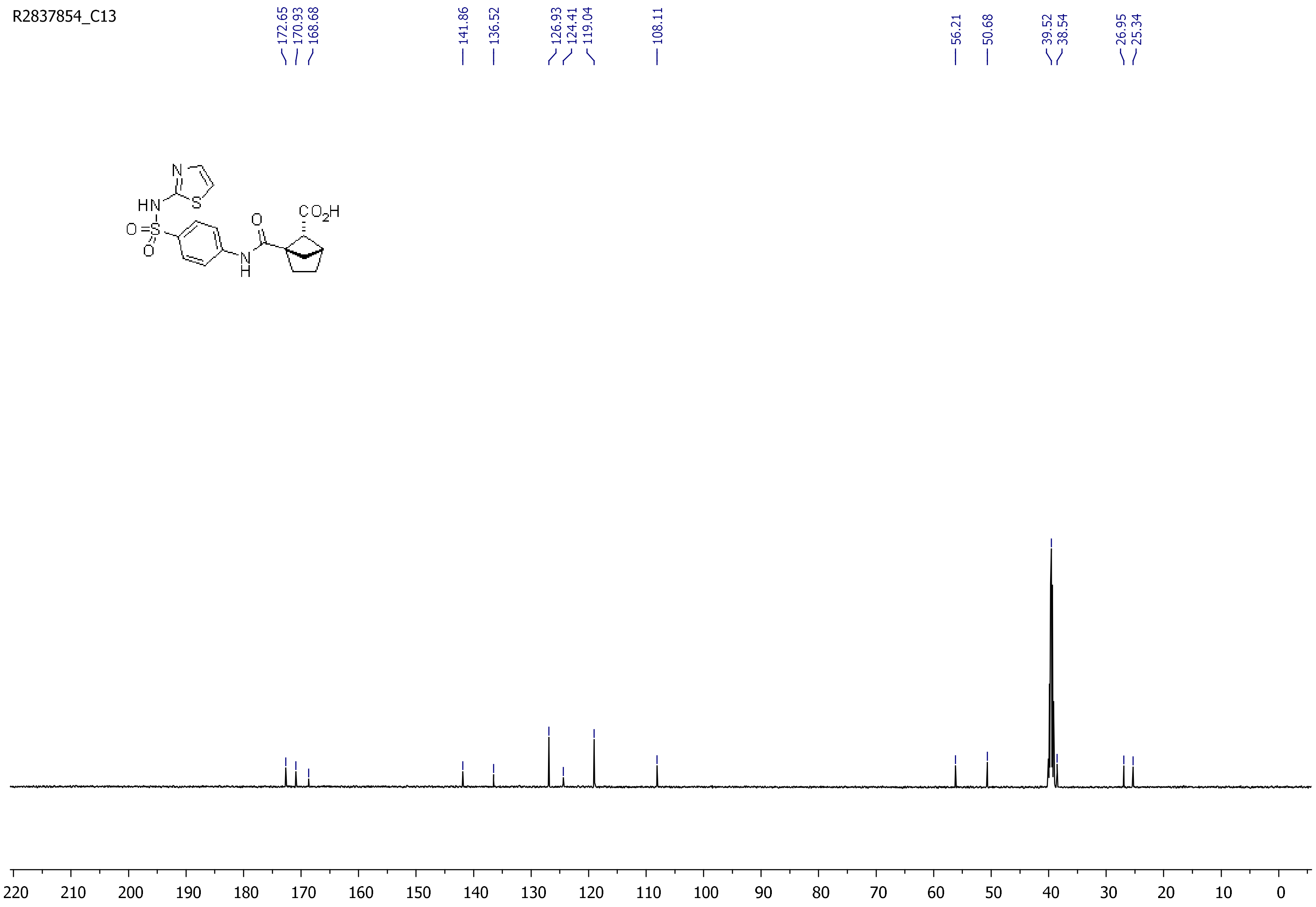

Compound (±)-34

^{1}H NMR (500 MHz, DMSO-d$_6$)

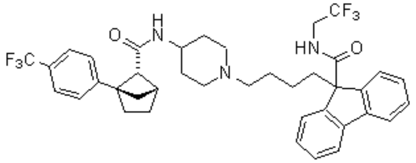

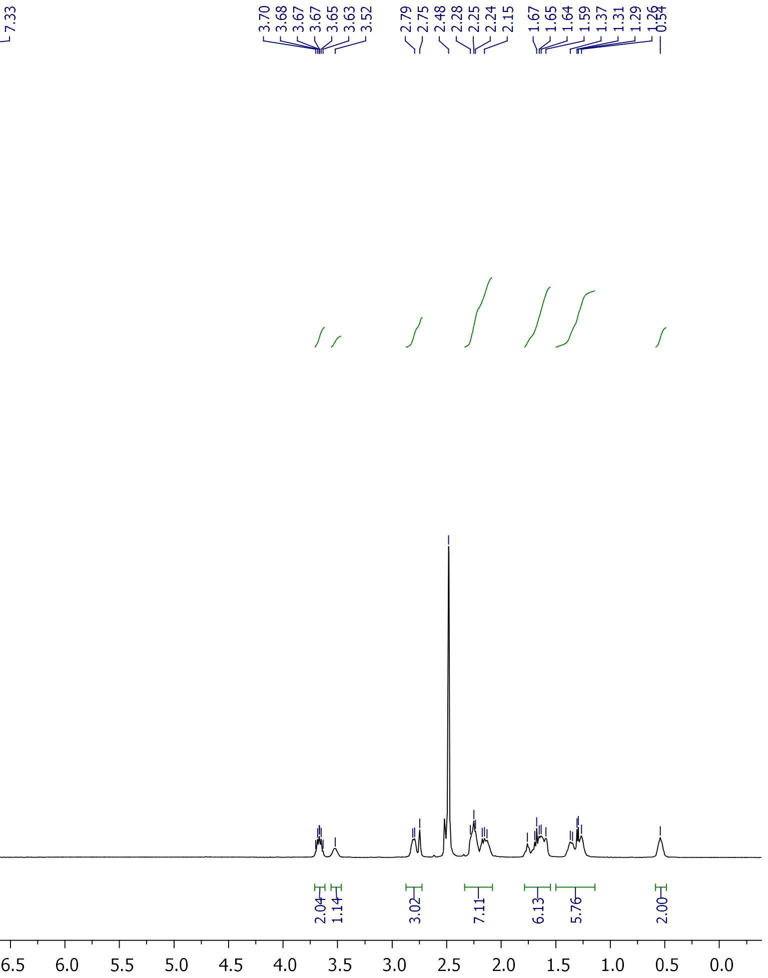

$^{19}F\{^{1}H\}$ NMR (376 MHz, DMSO-d_6)

R2837856_F19{H}

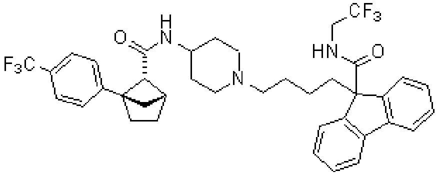

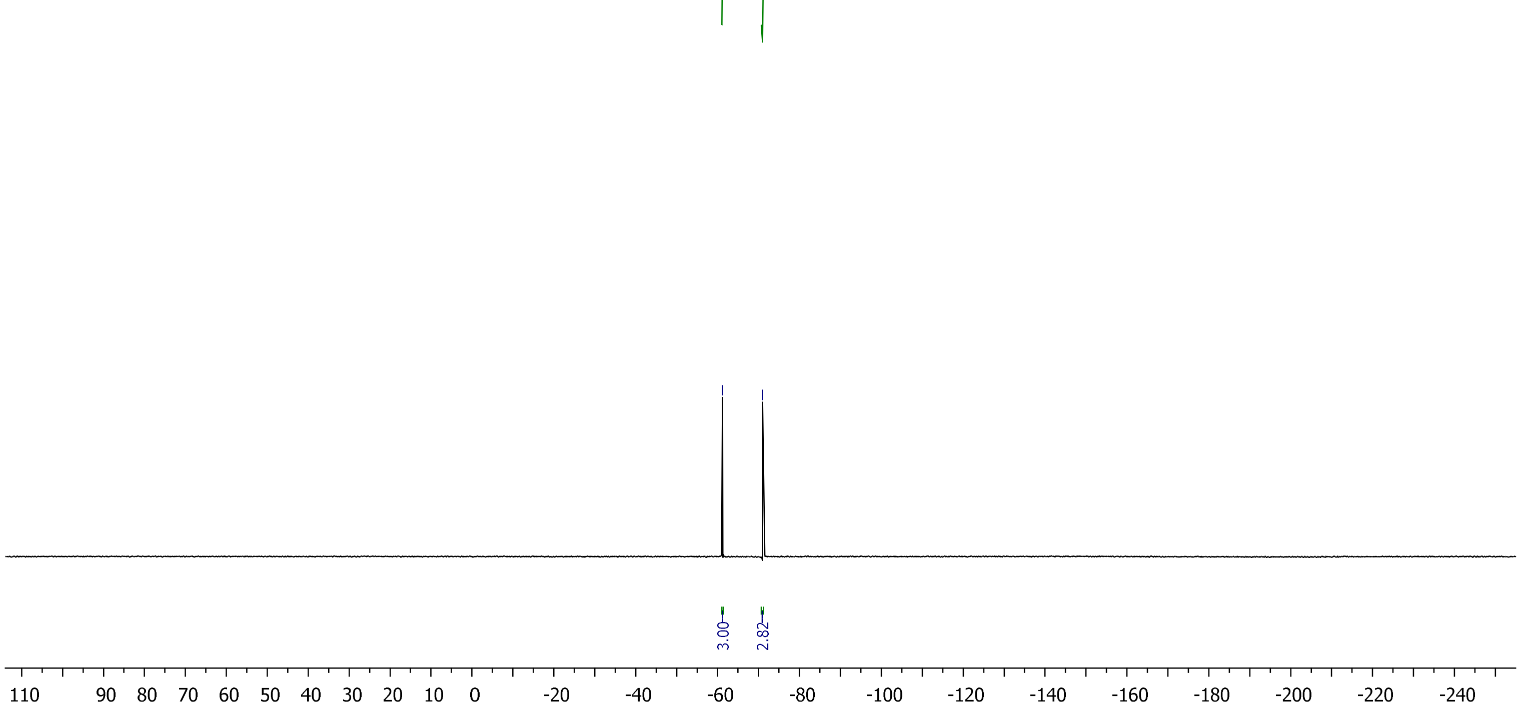

$^{13}C\{^{1}H\}$ NMR (151 MHz, DMSO-d_6)

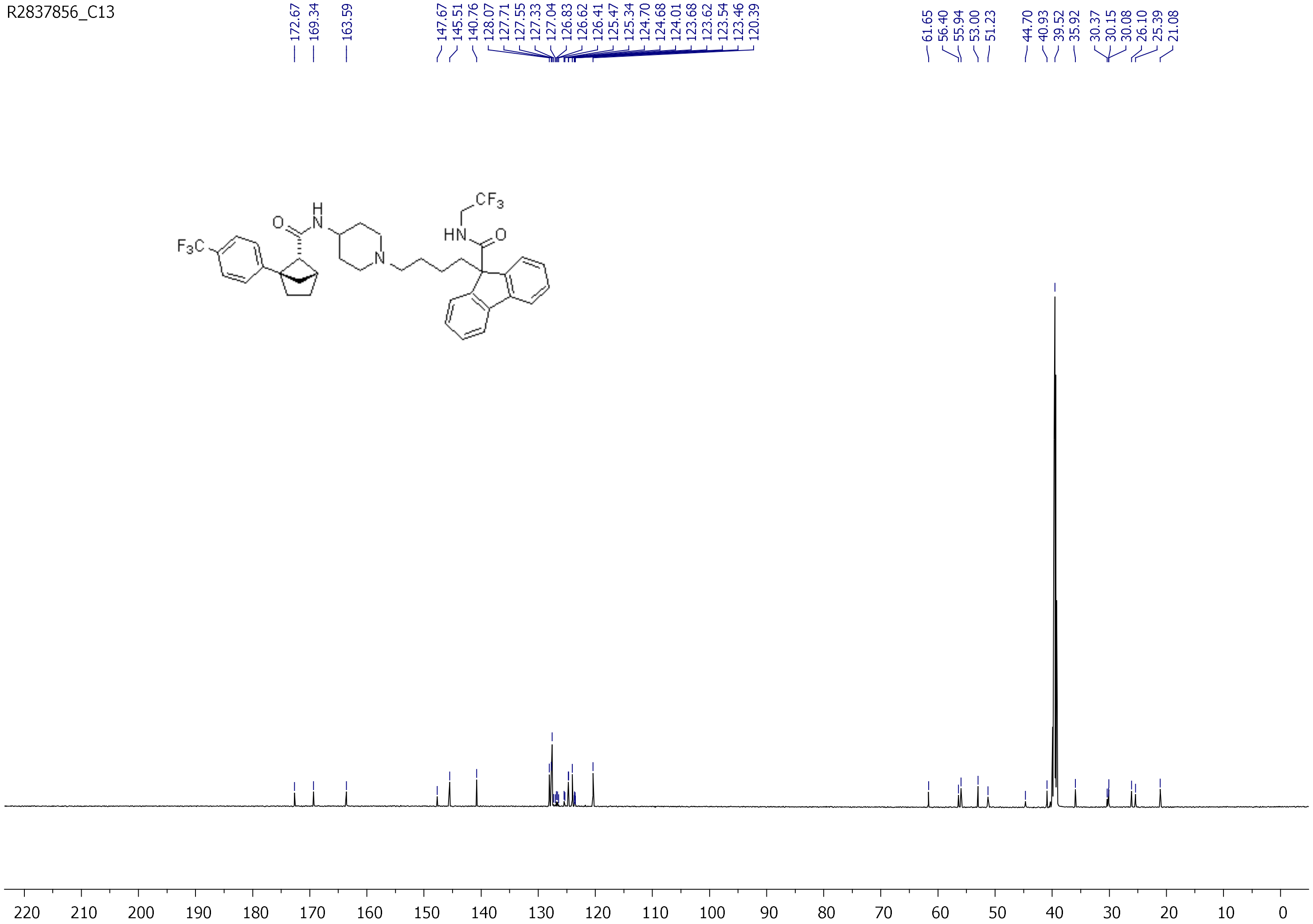

Compound (±)-35

^{1}H NMR (500 MHz, $CDCl_3$)

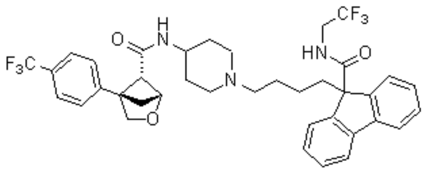

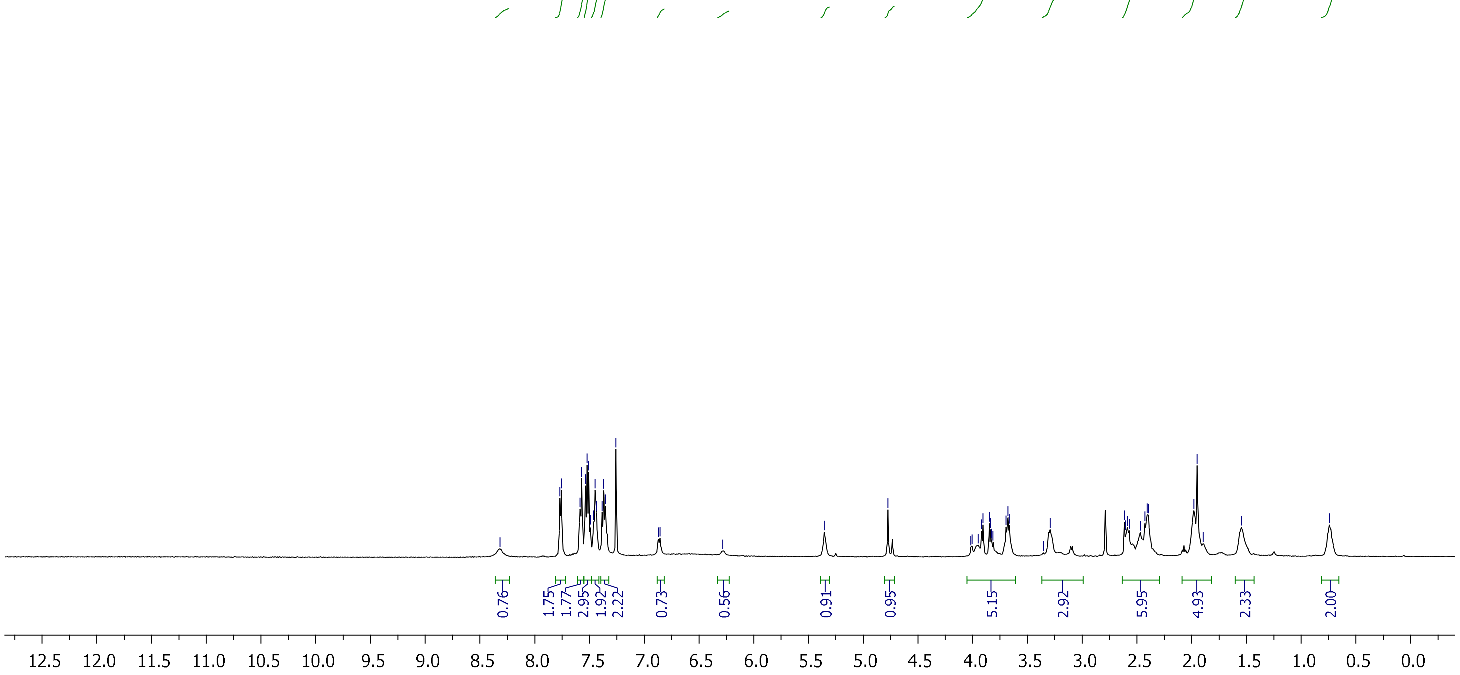

$^{13}C\{^{1}H\}$ NMR (126 MHz, $CDCl_3$)

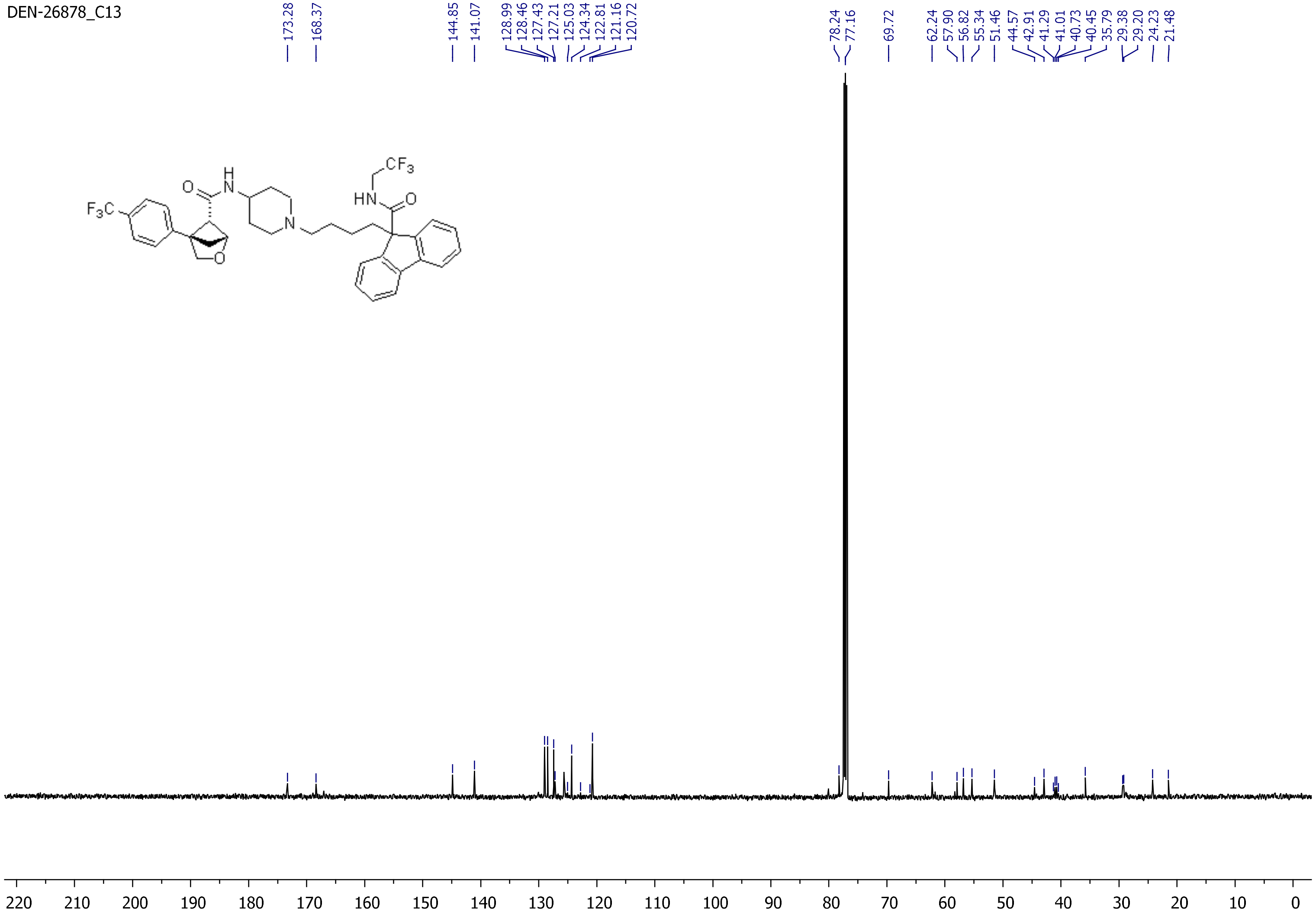

$^{19}F\{^{1}H\}$ NMR (376 MHz, $CDCl_3$)

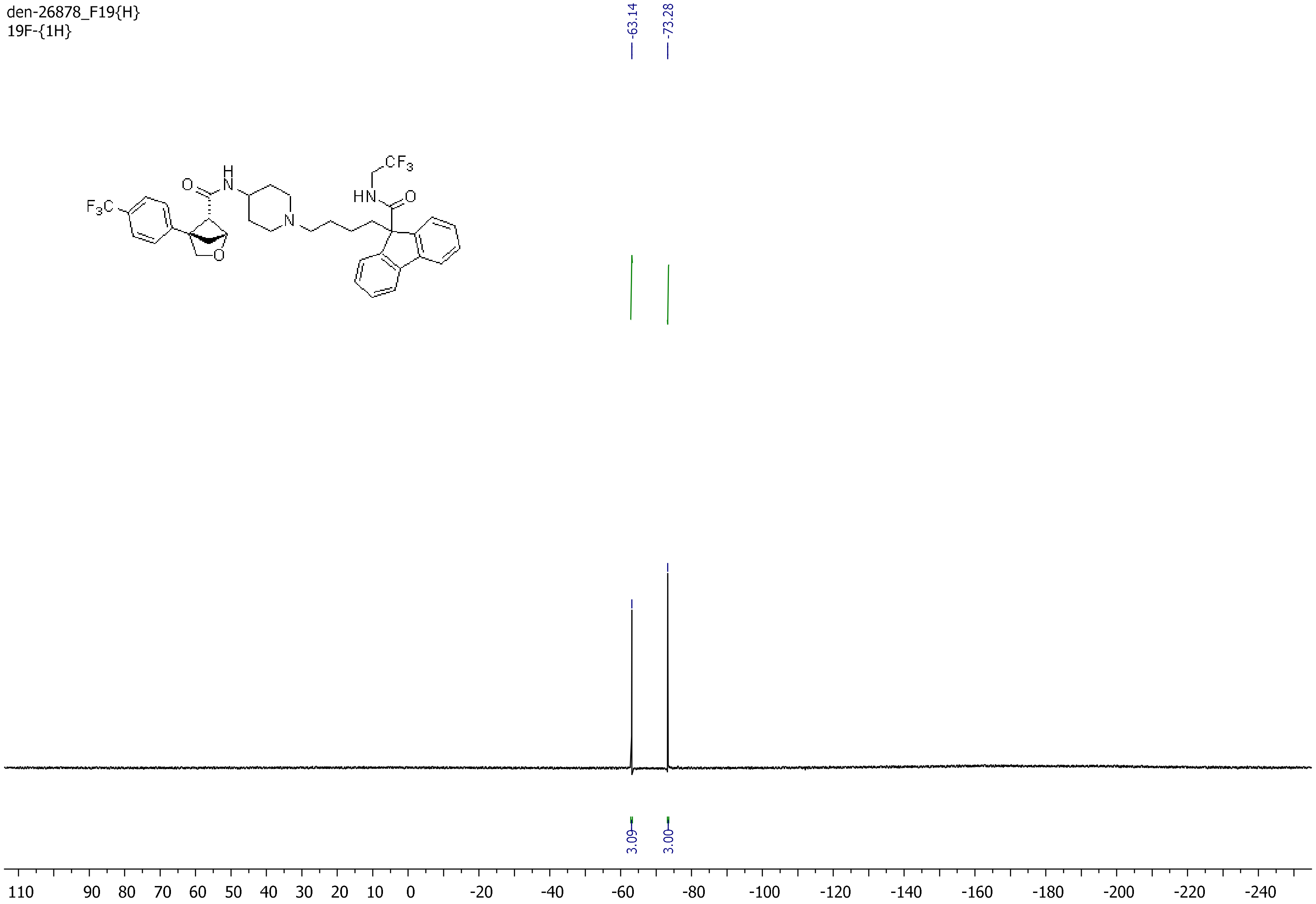

Crystallographic data (X-Ray)

Crystals of compounds **5b** and **9b** suitable for X-Ray diffraction studies were obtained by a low evaporation of a solution of *i*PrOH-toluene (10:1). Diffraction data were collected at room temperature on an Xcallibur-3 diffractometer with graphite-monochromated Mo Kα radiation (λ = 0.71073 Å) operating in the w-scans mode. The structure was solved by direct methods and refined by the full-matrix least-squares technique in the anisotropic approximation for non-hydrogen atoms using the SHELXTL program package. Crystallographic data for all structures in this paper have been deposited at Cambridge Crystallographic Data Centre. CCDC numbers: 2166325 (**5b**) and 2166326 (**9b**). Copies of the data can be obtained, free of charge, on application to CCDC, 12 Union Road, Cambridge CB21EZ, UK, (fax: +44-(0)1223-336033 or e-mail: deposit@ccdc.cam.ac.uk).

Compound **5b**

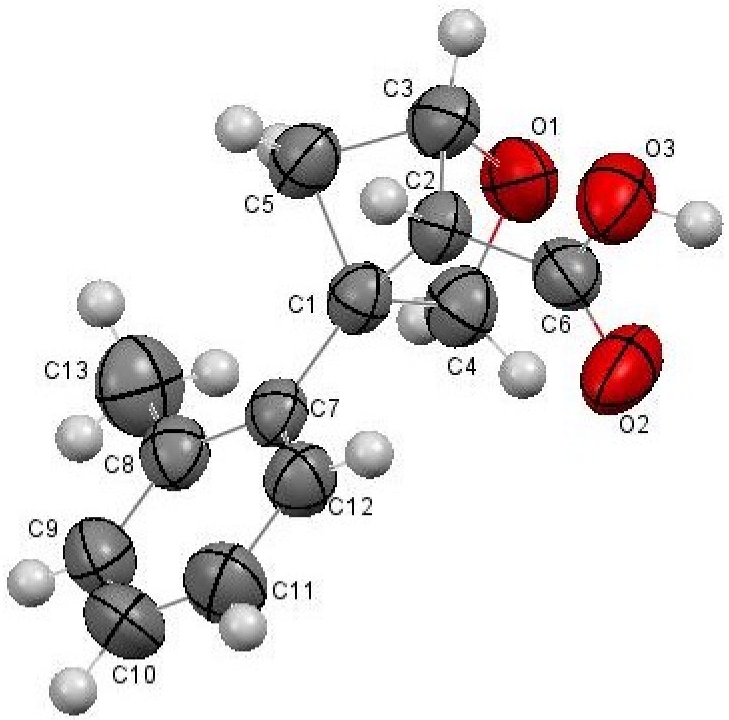

Figure S1. Molecular structure of **5b** according to X-Ray diffraction data. Thermal ellipsoids are shown at 50% probability level.

Crystal structure determination of 5b
data_t353

_audit_creation_method 'SHELXL-2016/6'

_shelx_SHELXL_version_number '2016/6'

_chemical_formula_moiety '$C_{13}H_{14}O_3$'

_chemical_formula_weight 218.24

_space_group_crystal_system monoclinic

_space_group_IT_number 14

_space_group_name_H-M_alt 'P 21/c'

_space_group_name_Hall '-P 2ybc'

_cell_length_a 7.7649(14)

_cell_length_b 5.6680(9)

_cell_length_c 25.802(4)

_cell_angle_alpha 90

_cell_angle_beta 98.022(14)

_cell_angle_gamma 90

_cell_volume 1124.5(3)

_cell_formula_units_Z 4

_cell_measurement_temperature 293(2)

_cell_measurement_reflns_used 535

_cell_measurement_theta_min 3.917

_cell_measurement_theta_max 19.612

_exptl_crystal_description stick

_exptl_crystal_colour colorless

_exptl_crystal_density_diffrn 1.289

_exptl_crystal_F_000 464

_exptl_crystal_size_max 0.200

_exptl_crystal_size_mid 0.050

_exptl_crystal_size_min 0.020

_exptl_absorpt_coefficient_mu 0.091

_shelx_estimated_absorpt_T_min 0.982

_shelx_estimated_absorpt_T_max 0.998

_exptl_absorpt_correction_type 'multi-scan'

_exptl_absorpt_correction_T_min 0.44329

_exptl_absorpt_correction_T_max 1.00000

_diffrn_ambient_temperature 293(2)

_diffrn_radiation_wavelength 0.71073

_diffrn_radiation_type MoK\a

_diffrn_source 'Enhance (Mo) X-ray Source'

_diffrn_measurement_device_type 'Xcalibur, Sapphire3'

_diffrn_measurement_method '\w scans'

_diffrn_detector_area_resol_mean 16.1827

_diffrn_reflns_number 7191

_diffrn_reflns_av_unetI/netI 0.0796

_diffrn_reflns_av_R_equivalents 0.0848

_diffrn_reflns_limit_h_min -9

_diffrn_reflns_limit_h_max 9

_diffrn_reflns_limit_k_min -6

_diffrn_reflns_limit_k_max 6

_diffrn_reflns_limit_l_min -30

_diffrn_reflns_limit_l_max 24

_diffrn_reflns_theta_min 3.189

_diffrn_reflns_theta_max 24.996

_diffrn_reflns_theta_full 24.996

_diffrn_measured_fraction_theta_max 0.998

_diffrn_measured_fraction_theta_full 0.998

_diffrn_reflns_Laue_measured_fraction_max 0.998

_diffrn_reflns_Laue_measured_fraction_full 0.998

_diffrn_reflns_point_group_measured_fraction_max 0.998

_diffrn_reflns_point_group_measured_fraction_full 0.998

_reflns_number_total 1985

_reflns_number_gt 1042

_reflns_threshold_expression 'I > 2\s(I)'

_reflns_Friedel_coverage 0.000

Compound **9b**

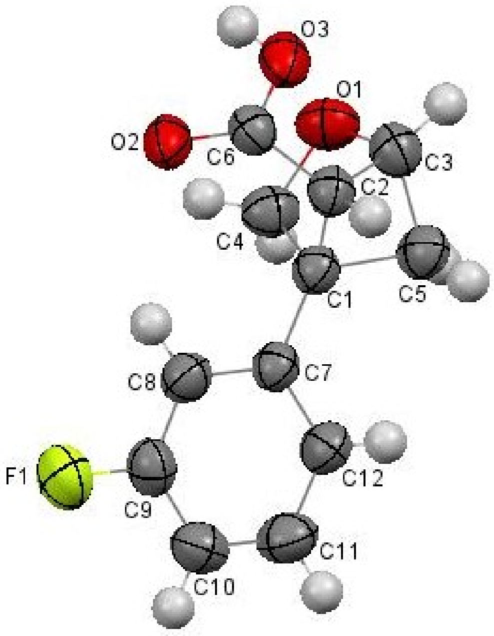

Figure S2. Molecular structure of **9b** according to X-Ray diffraction data. Thermal ellipsoids are shown at 50% probability level.

Crystal structure determination of 9b

data_tolm355

_audit_creation_method 'SHELXL-2016/6'

_shelx_SHELXL_version_number '2016/6'

_chemical_formula_moiety '$C_{12}H_{11}FO_3$'

_chemical_formula_weight 222.21

_space_group_crystal_system monoclinic

_space_group_IT_number 14

_space_group_name_H-M_alt 'P 21/n'

_space_group_name_Hall '-P 2yn'

_cell_length_a 7.6858(9)

_cell_length_b 15.4550(11)

_cell_length_c 9.1457(7)

_cell_angle_alpha 90

_cell_angle_beta 105.970(10)

_cell_angle_gamma 90

_cell_volume 1044.44(17)

_cell_formula_units_Z 4

_cell_measurement_temperature 293(2)

_cell_measurement_reflns_used 762

_cell_measurement_theta_min 3.045

_cell_measurement_theta_max 27.028

_exptl_crystal_description block

_exptl_crystal_colour colorless

_exptl_crystal_density_diffrn 1.413

_exptl_crystal_F_000 464

_exptl_crystal_size_max 0.200

_exptl_crystal_size_mid 0.150

_exptl_crystal_size_min 0.100

_exptl_absorpt_coefficient_mu 0.113

_shelx_estimated_absorpt_T_min 0.978

_shelx_estimated_absorpt_T_max 0.989

_exptl_absorpt_correction_type 'multi-scan'

_exptl_absorpt_correction_T_min 0.22376

_exptl_absorpt_correction_T_max 1.00000

_diffrn_ambient_temperature 293(2)

_diffrn_radiation_wavelength 0.71073

_diffrn_radiation_type MoK\a

_diffrn_source 'Enhance (Mo) X-ray Source'

_diffrn_measurement_device_type 'Xcalibur, Sapphire3'

_diffrn_measurement_method '\w scans'

_diffrn_detector_area_resol_mean 16.1827

_diffrn_reflns_number 6889

_diffrn_reflns_av_unetI/netI 0.0845

_diffrn_reflns_av_R_equivalents 0.1054

_diffrn_reflns_limit_h_min -9

_diffrn_reflns_limit_h_max 9

_diffrn_reflns_limit_k_min -18

_diffrn_reflns_limit_k_max 15

_diffrn_reflns_limit_l_min -10

_diffrn_reflns_limit_l_max 10

_diffrn_reflns_theta_min 3.056

_diffrn_reflns_theta_max 24.991

_diffrn_reflns_theta_full 24.991

_diffrn_measured_fraction_theta_max 0.999

_diffrn_measured_fraction_theta_full 0.999

_diffrn_reflns_Laue_measured_fraction_max 0.999

_diffrn_reflns_Laue_measured_fraction_full 0.999

_diffrn_reflns_point_group_measured_fraction_max 0.999

_diffrn_reflns_point_group_measured_fraction_full 0.999

_reflns_number_total 1832

_reflns_number_gt 1086

_reflns_threshold_expression 'I > 2\s(I)'

_reflns_Friedel_coverage 0.000

Analysis of Aqueous Solubility

Test articles (EN300-18202568 (Phthalylsulfathiazole), EN300-37274652 (33), EN300-37082352 (34), EN300-20331690 (Lopitamide), EN300-37082350 (35), EN300-264529 (Fluxapyroxad),[5, 6] EN300-7394812 (Boscalid),[7] EN300-37335003 (28), EN300-36480610 (29), EN300-7460805 (30), EN300-37274650 (32), and EN300-37152265 (31)) and reference compound (Ondansetron) were assessed for kinetic solubility in phosphate-buffered saline, pH 7.4.

Reagents and consumables

Phosphate buffered saline, pH 7.4 (Sigma-Aldrich, USA; Cat #P3813)
Acetonitrile Chromasolv, gradient grade, for HPLC, ≥99.9% (Sigma-Aldrich, USA; Cat #34851)
Ondansetron base powder (Enamine, Ukraine, Cat # EN300-117273)
DMSO (Sigma-Aldrich, USA; Cat # 34869)
Costar 96 Well Assay Blocks (Corning, USA; Cat # 3958)
MultiScreen HTS 96 Well Filter Plates (Millipore, Ireland; Cat # MSGVS2210)
UV-Star® 96 Well Microplate (Greiner Bio-One, Germany; Cat #655801)
Matrix Disposable pipette tips (ThermoScientific, USA; Cat ## 8041, 7622, 7321)
Flex-Tubes Microcentrifuge Tubes, 1.5ml (Eppendorf, Germany; Cat # 22364111)
Matrix Storage tubes, 1.4 ml (ThermoScientific, USA; Cat # 4247)

Equipment

Water purification system Millipore Milli-Q Gradient A10 (Millipore, France)
Thermomixer R Block, 1.5 mL (Eppendorf, Germany; Cat # 5355)
Matrix Multichannel Electronic Pipette 2-125 µL, 5-250 µL, 15-1250 µL (Thermo Scientific, USA; Cat ## 2011, 2012, 2004)
SpectraMax Plus Microplate Reader (Molecular Devices, USA; Product # 02196)
Multi-Well Plate Vacuum Manifold (Pall Corporation, USA; Product # 5014)
Vacuum pump (Millipore, USA; Model # XX5500000)

---

[5] EPA Registration Number 7969-308 (April 15, 2015; Fluxapyroxad).

[6] Commission Implementing Regulation (EU) No 589/2012 (July 4, 2012; Fluxapyroxad).

[7] EPA Registration Number 128008 (July 1, 2003; Boskalid).

Analytical System

The measurements were performed using SpectraMax Plus reader in UV-Vis mode. Acquisition and analysis of the data were performed using SoftMax Pro v.5.4 (Molecular Devices) and Excel 2010 data analysis software.

Methods

Kinetic solubility assay was performed according to the Enamine's aqueous solubility SOP. Briefly, using a 20 mM stock solution of the compound in 100% DMSO dilutions were prepared to a theoretical concentration of 400 μM in duplicates in phosphate-buffered saline pH 7.4 (138 mM NaCl, 2.7 mM KCl, 10 mM K-phosphate) with 2% final DMSO. The experimental compound dilutions in PBS were further allowed to equilibrate at 25 °C on a thermostatic shaker for two hours and then filtered through HTS filter plates using a vacuum manifold. The filtrates of test compounds were diluted 2-fold with acetonitrile with 2% DMSO before measuring.

In parallel, compound dilutions in acetonitrile/PBS (1:1) were prepared to theoretical concentrations of 0 μM (blank), 10 μM, 25 μM, 50 μM, 100 μM, and 200 μM with 2% final DMSO to generate calibration curves. Ondansetron was used as reference compound to control proper assay performance. 200 μl of each sample was transferred to 96-well plate and measured in 200-550 nm range with 5 nm step.

The concentrations of compounds in PBS filtrate are calculated using a dedicated Microsoft Excel calculation script. Proper absorbance wavelengths for calculations are selected for each compound manually based on absorbance maximums (absolute absorbance unit values for the minimum and maximum concentration points within 0 – 3 OD range). Each of the final datasets is additionally visually evaluated by the operator and goodness of fit (R2) is calculated for each calibration curve. The effective range of this assay is approximately 2-400 μM and the compounds returning values close to the upper limit of the range may have higher actual solubility (e.g. 5'-deoxy-5-fluorouridine). This method is not suitable for liquid (at 25 °C) substances (were not present among the tested compounds).

Two test articles (EN300-37335003 (28) and EN300-7460805 (30)) had low light absorbance (insufficient UV-Vis signal) and were detected using a HPLC-MS system.

Results

Table S1. The solubility data of the test and reference compounds The calibration curves are shown in the Appendix*.

ID	PBS solubility, pH 7.4, µM			SE
	**Incubation 1**	**Incubation 2**	**Mean**	
Ondansetron	120	116	**118****	1.2
EN300-18202568 (Phthalylsulfathiazole)	169	171	**170**	1.2
EN300-37274652 (33)	100	102	**101**	0.6
EN300-37082352 (34)	2	2	**2**	0.2
EN300-20331690 (Lopitamide)	3	3	**3**	0.1
EN300-37082350 (35)	2	4	**3**	0.8
EN300-264529 (Fluxapyroxad)	24	27	**25**	1.1
EN300-7394812 (Boscalid)	9	13	**11**	2.0
EN300-37274650 (32)	151	164	**158**	6.9
EN300-37152265 (31)	148	157	**152**	4.4
EN300-37335003 (28)	35	33	**34**	0.4
EN300-36480610 (29)	151	158	**155**	3.2
EN300-7460805 (30)	17	18	**17**	0.3

*Goodness of fit (R^2) in all titration curves as well as the variations between repeat measurements indicates high quality of the experimental data in the current batch of test articles.
**Ondansetron solubility data are consistent with previously obtained.

APPENDIX

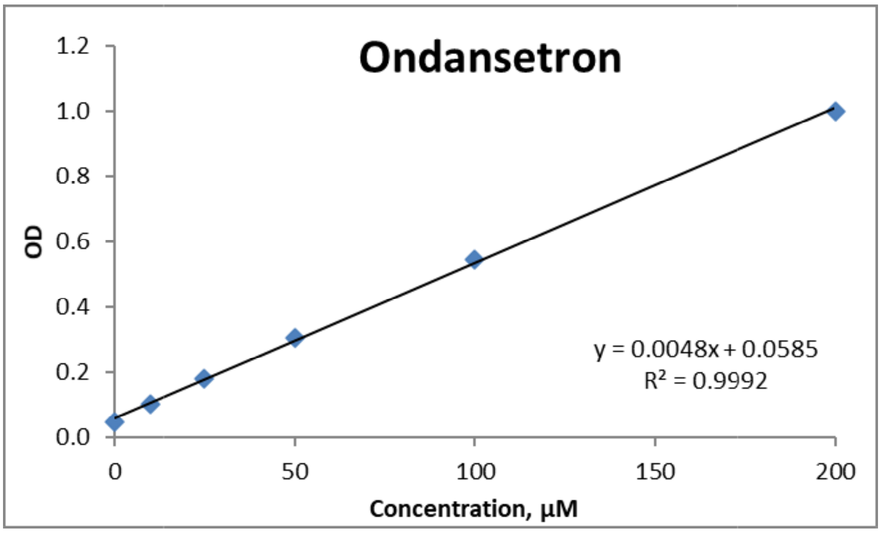

Figure S3. Calibration curve for **Ondansetron**

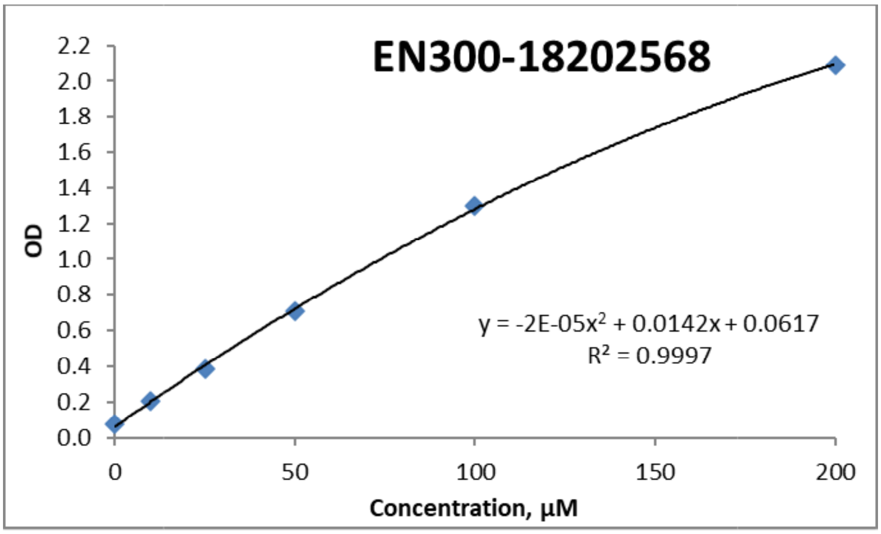

Figure S4. Calibration curve for **EN300-18202568 (Phthalylsulfathiazole)**

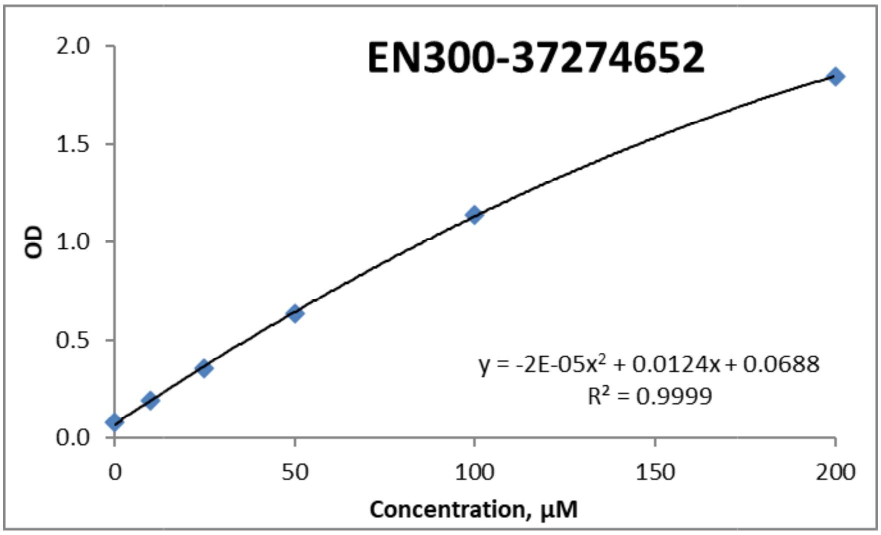

Figure S5. Calibration curve for **EN300-37274652 (33)**

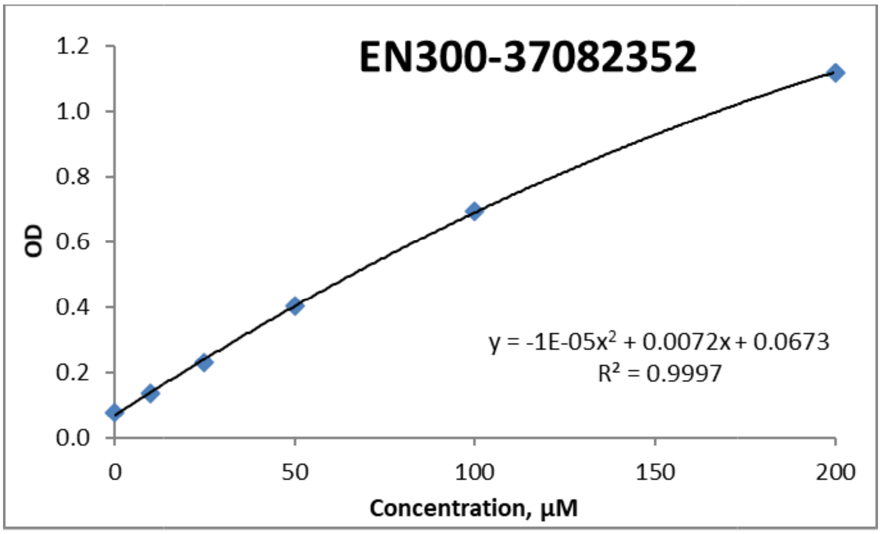

Figure S6. Calibration curve for **EN300-37082352 (34)**

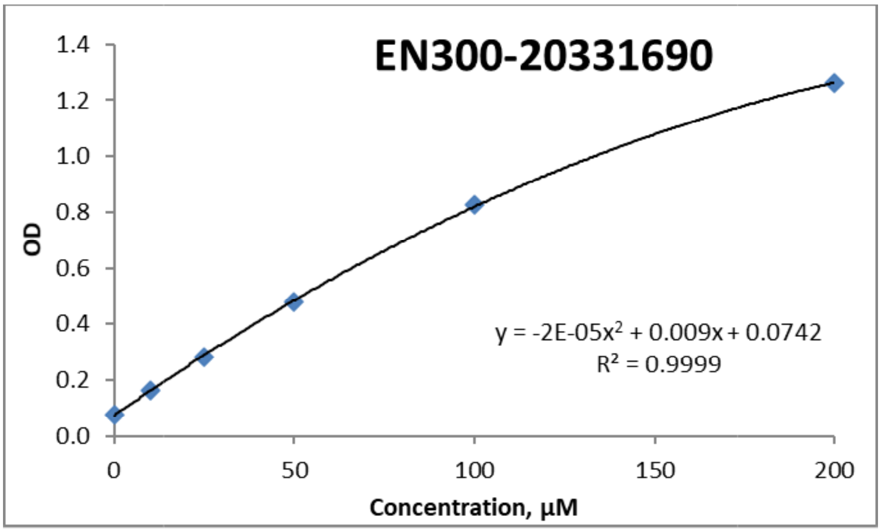

Figure S7. Calibration curve for **EN300-20331690 (Lopitamide)**

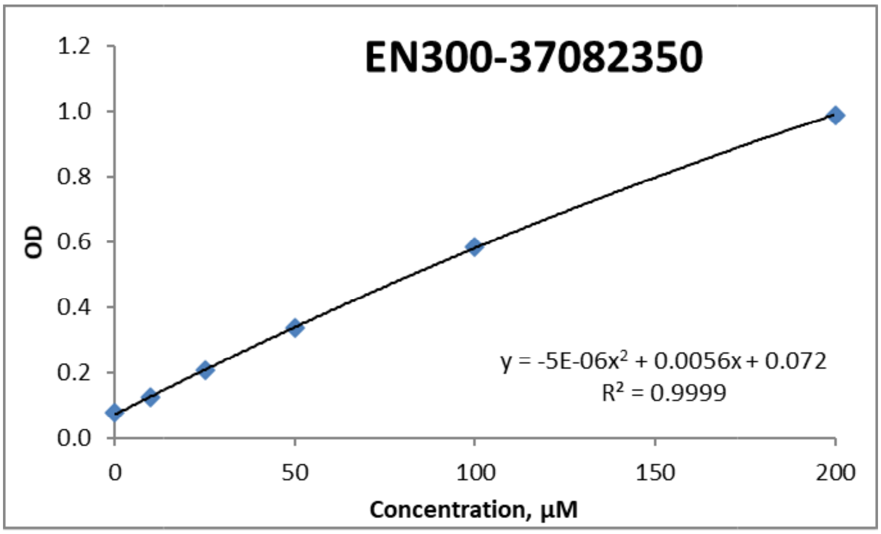

Figure S8. Calibration curve for **EN300-37082350 (35)**

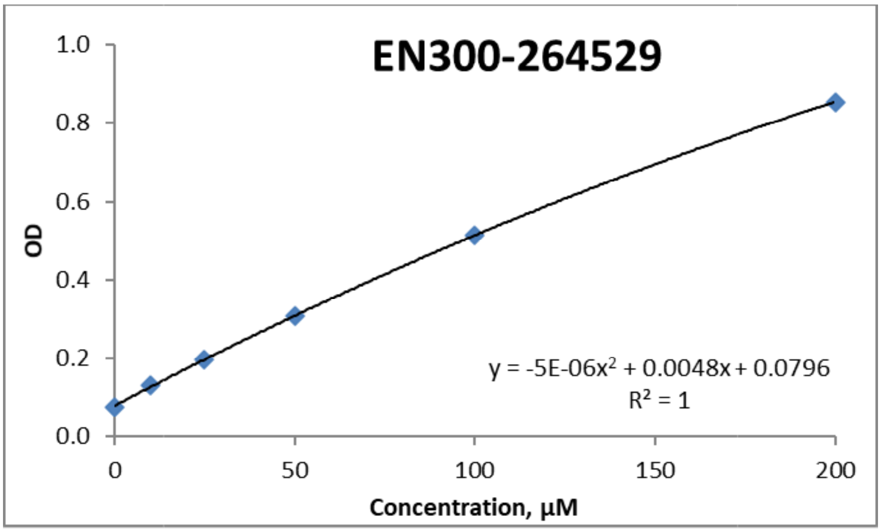

Figure S9. Calibration curve for **EN300-264529 (Fluxapyroxad)**

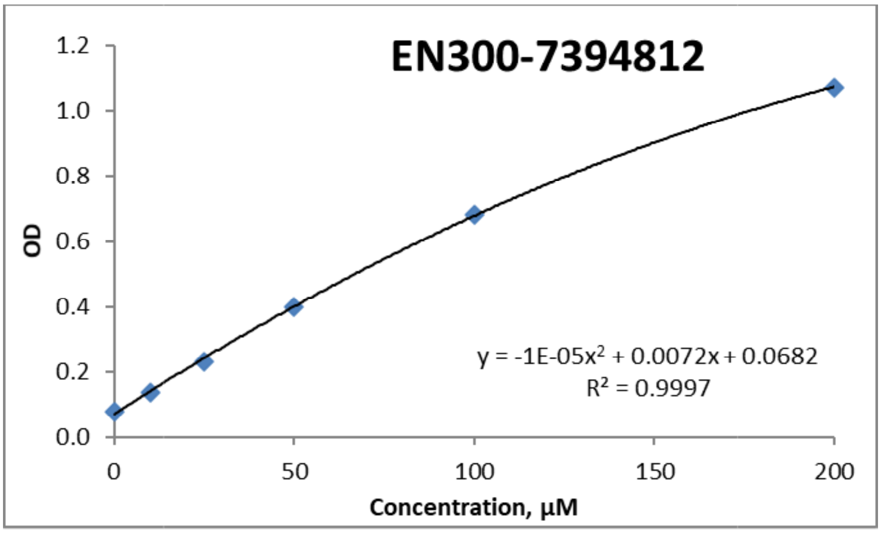

Figure S10. Calibration curve for **EN300-7394812 (Boscalid)**

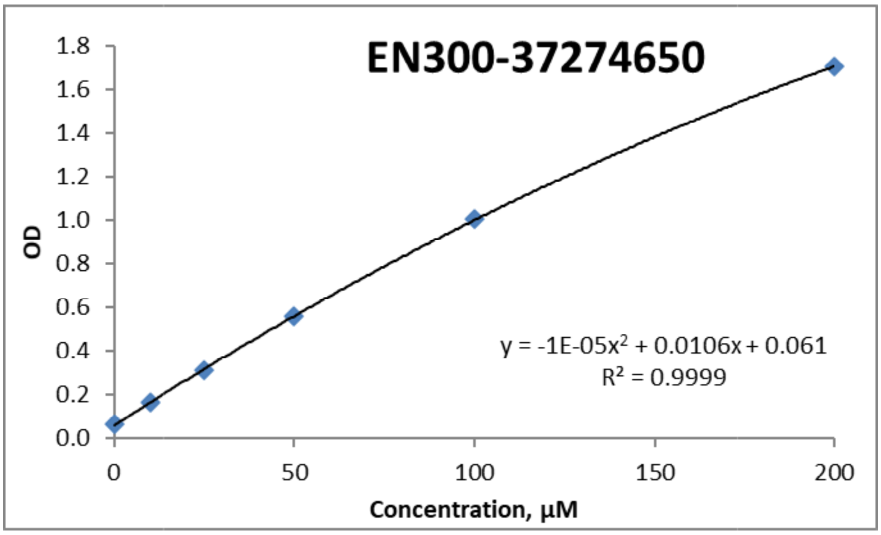

Figure S11. Calibration curve for **EN300-37274650 (32)**

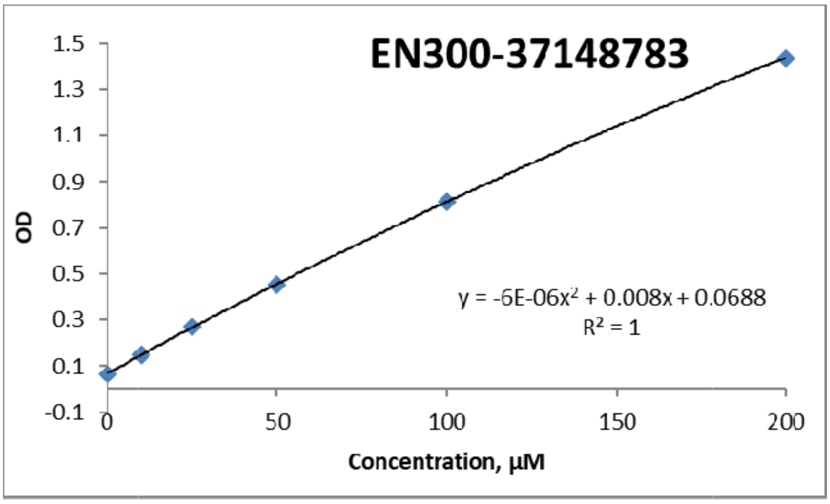

Figure S12. Calibration curve for **EN300-37152265 (31)**

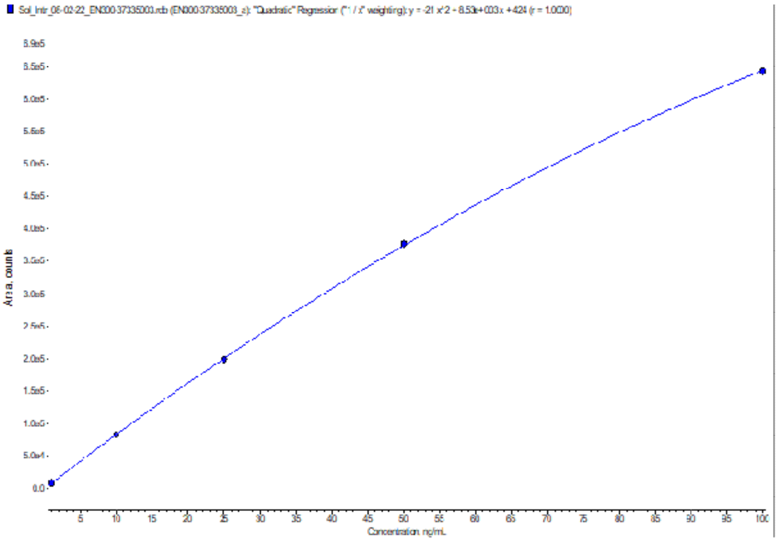

Figure S13. Calibration curve for **EN300-37335003 (28)**

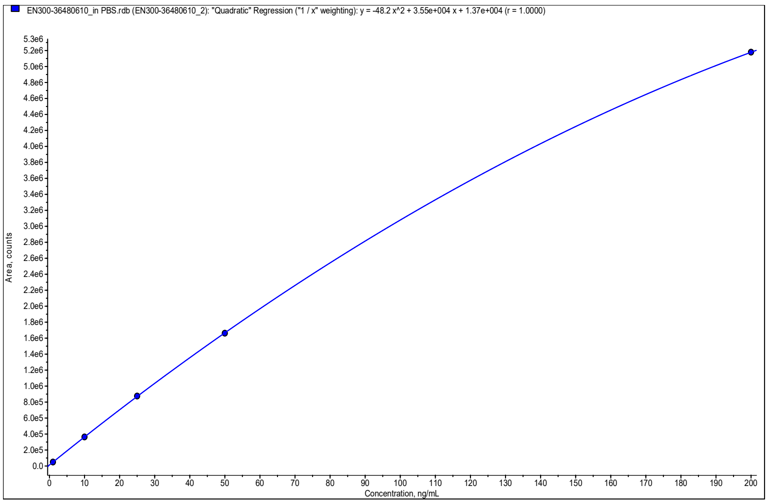

Figure S14. Calibration curve for **EN300-36480610 (29)**

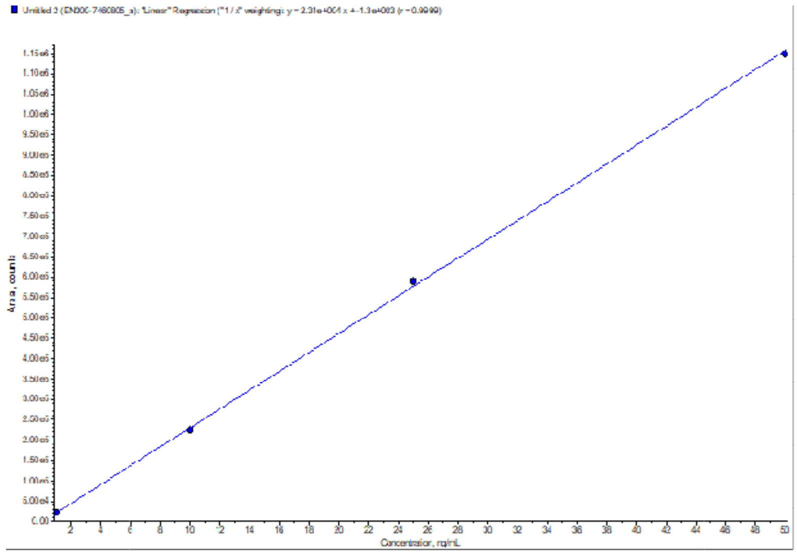

Figure S15. Calibration curve for **EN300-7460805 (30)**

Determination of Distribution Coefficient (LogD, pH 7.4)

Test articles EN300-18202568 (Phthalysulfathiazole), EN300-37274652 (33), EN300-37082352 (34), EN300-20331690 (Lopitamide), EN300-37082350 (35), EN300-264529 (Fluxapyroxad), EN300-7394812 (Boscalid), EN300-7460805 (30), EN300-37335003 (28), EN300366480610 (29), EN300-37274650 (32), EN300-37152265 (31) and reference compound (Mebendazole) in *n*-octanol – phosphate buffered saline (PBS), pH 7.4. Distribution coefficient (or LogD) is a logarithm of the ratio of drug concentrations in two immiscible solvents, typically pH-buffered water and *n*-octanol. It is a measure of hydrophobic/hydrophilic properties of a given molecule. The partition of test compounds is determined using a shake-flask method, which involves mixing of a certain amount of the solute of interest in defined volumes of *n*-octanol and an aqueous buffer of choice followed by equilibration of the mixture by incubation with efficient mixing. Then, the distribution of the compounds in each solvent was controlled using LC-MS/MS.

Reagents and consumables

DMSO Chromasolv Plus, HPLC grade, ≥99.7% (Sigma-Aldrich, USA; Cat #34869)
Acetonitrile Chromasolv, gradient grade, for HPLC, ≥99.9% (Sigma-Aldrich, USA; Cat #34851)
Formic acid for mass spectrometry, ~98% (Fluka, USA; Cat #94318)
Phosphate buffered saline, tablet (Sigma-Aldrich, USA; Cat # P4417)
Acetic acid (Enamine, Ukraine.)
1-Octanol ACS grade, ≥99% (Sigma-Aldrich, USA; Cat # 472328)
Mebendazole analytical standard, ≥ 98%, HPLC (Sigma-Aldrich, USA; Cat # M2523)
DMSO stock solutions of the test compounds 10mM
Phenomenex Luna® C18 HPLC column, 2.1 × 50 mm, 5 µm (Cat #5291-126)
1.1 mL microtubes in microracks, pipettor tips (Thermo Scientific, USA).
National Scientific MicroTube™ Rack (Thermo Fisher Scientific, USA; Cat # TN094612R)

Equipment

Gradient HPLC system (Shimadzu, Japan)
Triple quadrapole mass-detector API 3000 with TurboIonSpray Ion Source (AB Sciex, Canada)
VWR Membrane Nitrogen Generators N2-04-L1466, nitrogen purity 99%+ (VWR, USA)
MTR22 Multi Mix Rotator (UNICO, USA)
Laboratory Centrifuge, Sigma 4-15C, Qiagen (SIGMA GmbH, Germany)

Water purification system Millipore Milli-Q Gradient A10 (Millipore, France)

Multichannel Electronic Pipettes 0.5-12.5 µL, 2-125 µL, 5-250 µL, 15-1250 µL, Matrix (Thermo Scientific, USA; Cat ## 2009, 2001, 2002, 2004)

Analytical System

All measurements were performed using a Shimadzu Prominence HPLC system including a vacuum degasser, gradient pumps, a reverse phase column, a column oven and an autosampler. Mass spectrometric analysis was performed using an API 4000 QTRAP mass spectrometer from Applied Biosystems/MDS Sciex (AB Sciex) with Turbo V ion source and TurboIonspray interface. The TurboIonSpray ion source was used in both positive and negative ion modes. Acquisition and analysis of the data were performed using Analyst 1.6.3 software.

Methods

Incubations were carried out in Eppendorf-type polypropylene microtubes in triplicates. 5 µL aliquot of 10 mM DMSO stock of a test compound was added into the previously mutually saturated mixture containing 500 µL of PBS (pH 7.4) and 500 µL of octanol. The solution was allowed to mix in a rotator for 1 h at 30 rpm. Phase separation was assured by centrifugation for 2 min at 6000 rpm. The octanol phase was diluted 100-fold with 40% acetonitrile, and the aqueous phase (PBS buffer) was diluted 10-fold; for compounds Mebendazole, EN300-18202568 (Phthalysulfathiazole), EN300-37274652 (33), EN300-37082352 (34), EN300-20331690 (Lopitamide), EN300-37082350 (35), EN300-264529 (Fluxapyroxad), EN300-7394812 (Boscalid), EN300-7460805 (30), EN300-37335003 (28), EN300366480610 (29), EN300-37274650 (32), and EN300-37152265 (31), the aqueous phase was analyzed without dilution. The samples (both phases) were analyzed using a HPLC system coupled with a tandem mass spectrometer. Mebendazole was used as a reference compound.

Calculations of the partition ratios were carried out using the equation below.

$$D = \frac{d_o \cdot S_O}{d_p \cdot S_P}$$

where: S_O – peak area of the analyte in octanol phase

S_P – peak area of the analyte in PBS buffer

d_o – dilution coefficient for octanol phase

d_p – dilution coefficient for aqueous phase

Results

LogD data for the reference compound (Mebendazole) and test compound is provided in the table below.

Table S2. Experimental LogD, pH 7.4

Compound ID	Incubation	S_P	S_O	D	LogD, pH 7.4	
Mebendazole	1	1.15E+05	1.46E+06	1.27E+03	3.10	3.12
	2	1.21E+05	1.59E+06	1.32E+03	3.12	
	3	1.02E+05	1.35E+06	1.33E+03	3.12	
EN300-18202568 (**Phthalysulfathiazole)**	1	7.96E+06	1.66E+04	2.09E-01	-0.68	-1.04
	2	7.86E+06	7.07E+03	8.99E-02	-1.05	
	3	8.50E+06	3.54E+03	4.17E-02	-1.38	
EN300-37274652 **(33)**	1	1.34E+05	3.20E+02	2.39E-01	-0.62	-0.87
	2	1.16E+05	2.23E+01	1.93E-02	-1.72	
	3	1.21E+05	6.65E+02	5.50E-01	-0.26	
EN300-37082352 **(34)**	1	6.54E-02	2.31E+05	3.53E+08	8.55	6.78*
	2	6.22E+00	2.11E+05	3.40E+06	6.53	
	3	1.20E+02	2.06E+05	1.71E+05	5.23	
EN300-20331690 **(Lopitamide)**	1	4.09E-02	2.45E+05	5.98E+08	8.78	6.39*
	2	5.92E+01	2.03E+05	3.42E+05	5.54	
	3	2.49E+02	1.69E+05	6.79E+04	4.83	
EN300-37082350 **(35)**	1	6.05E+01	8.81E+04	1.46E+05	5.16	5.11*
	2	5.95E+01	9.61E+04	1.62E+05	5.21	
	3	1.01E+02	8.96E+04	8.88E+04	4.95	

Compound ID	Incuba-tion	S_P	S_O	D	LogD, pH 7.4	
EN300-264529 **(Fluxapyroxad)**	1	2.45E+03	8.27E+04	3.37E+03	3.53	3.51
	2	2.31E+03	6.90E+04	2.99E+03	3.48	
	3	2.56E+03	8.29E+04	3.24E+03	3.51	
EN300-7394812 **(Boscalid)**	1	1.25E+04	4.53E+05	3.62E+03	3.56	3.55
	2	1.53E+04	4.37E+05	2.86E+03	3.46	
	3	1.18E+04	4.81E+05	4.07E+03	3.61	
EN300-7460805 **(30)**	1	3.16E+04	1.54E+05	4.87E+02	2.69	2.66
	2	4.08E+04	1.29E+05	3.16E+02	2.50	
	3	3.53E+04	2.15E+05	6.11E+02	2.79	
EN300-37335003 **(28)**	1	7.42E+02	2.40E+05	3.24E+04	4.51	4.32
	2	1.23E+03	2.46E+05	2.00E+04	4.30	
	3	1.89E+03	2.50E+05	1.32E+04	4.12	
EN300366480610 (**29**)	1	3.72E+04	2.12E+05	5.70E+02	2.76	2.77
	2	3.41E+04	2.09E+05	6.13E+02	2.79	
	3	4.16E+04	2.40E+05	5.77E+02	2.76	
EN300-37274650 **(32)**	1	1.08E+07	8.77E+03	8.13E-02	-1.09	-1.39*
	2	1.13E+07	3.65E+03	3.23E-02	-1.49	
	3	1.14E+07	3.12E+03	2.75E-02	-1.56	
EN300-37152265 **(31)**	1	2.55E+04	7.77E+05	3.05E+03	3.48	3.50
	2	2.48E+04	7.84E+05	3.16E+03	3.50	
	3	2.62E+04	8.21E+05	3.13E+03	3.50	

**** - Reliable maesurable range is approximately - 1 to 4.5***

Assessment of Metabolic Stability in Human Liver Microsomes

The objective of this study was to determine metabolic stability of test articles (EN300-37274652 (33), EN300-37274650 (32), EN300-37152265 (31), EN300-20331690 (Lopitamide), EN300-37082352 (34), EN300-18202568 (Phthalylsulfathiazole), EN300-37082350 (35), EN300-264529 (Fluxapyroxad), EN300-7394812 (Boscalid), EN300-37335003 (28), EN300-36480610 (29) and EN300-7460805 (30)) and reference compounds in human liver microsomes at five time points over 40 minutes using HPLC-MS. Metabolic stability is defined as the percentage of parent compound lost over time in the presence of a metabolically active test system.

Reagents and consumables

DMSO (Sigma-Aldrich, 34869 - Chromasolv Plus, for HPLC, ≥99.7%)
Acetonitrile (Sigma-Aldrich, 34851 - Chromasolv Plus, for HPLC, ≥99.9%)
Methanol, for HPLC, ≥99.9% (Sigma-Aldrich, Cat #34860)
Ammonium acetate (Enamine, Ukraine, Cat# R59024)
Potassium phosphate monobasic (Helicon, Am-O781-0.5)
Potassium phosphate dibasic (Helicon, Am-O705-0.5)
Magnesium chloride hexahydrate (Helicon, Am-O288-0.1)
Human Liver Microsomes: pooled, mixed gender (XenoTech, H0630/lot #2010065)
Glucose-6-phosphate dehydrogenase from baker's yeast, type XV (Sigma-Aldrich, USA; G6378)
Glucose-6-phosphate sodium salt (Sigma-Aldrich, USA; G7879)
β-Nicotinamide adeninedinucleotide-2'-phosphate reduced, tetrasodium salt (Sigma Aldrich, USA; Cat #N1630)
Formic acid (Sigma-Aldrich, USA; 94318)
Niclosamide (Sigma Aldrich, USA; Cat #N3510)
Verapamil hydrochloride (Sigma Aldrich, USA; Cat #V4629)
(+,-) Propranolol hydrochloride (Sigma-Aldrich, USA; P0884)
Diclofenac, 96% purity (Enamine, # EN300-119509)
Phenomenex Luna® C18 HPLC column, 2.1x50 mm, 5 µm (Cat #5291-126)
1.1 ml microtubes in microracks, pipettor tips (Thermo Scientific).

Equipment

Gradient HPLC system (Shimadzu)

API 5000 mass spectrometer with Turbo V ion source (AB Sciex)
API 4000 QTRAP mass spectrometer with Turbo V ion source (AB Sciex)
Nitrogen generator N2-04-L1466, nitrogen purity 99%+ (Whatman)
Environmental Incubator Shaker G24; Digital Refrigerated Incubator/Shaker Innova 4330 (New Brunswick Scientific)
Water purification system Millipore Milli-Q Gradient A10 (Millipore, France)
Multichannel pipettors 5-250 µL, 2-125 µL, 15-1250 µL (Thermo Scientific)

Analytical System

All measurements were performed using a Shimadzu HPLC system including a vacuum degasser, gradient pumps, a reverse phase HPLC column, a column oven, and an autosampler. Mass spectrometric analysis was performed using an API 5000 mass spectrometer and/or a Mass spectrometric analysis was performed using an API 4000 QTRAP mass spectrometer with Turbo V ion source (AB Sciex). The TurboIonSpray ion source was used in both positive and negative ion modes. The data acquisition and system control was performed using Analyst 1.6.3 software from AB Sciex.

Methods

Microsomal incubations were carried out in 96-well plates in 5 aliquots of 30 µL each (one for each time point). Liver microsomal incubation medium comprised of phosphate buffer (100 mM, pH 7.4), $MgCl_2$ (3.3 mM), NADPH (3 mM), glucose-6-phosphate (5.3 mM), glucose-6-phosphate dehydrogenase (0.67 units/mL) with 0.42 mg of liver microsomal protein per ml. In the control reactions the NADPH-cofactor system was substituted with phosphate buffer. Test compounds (2 µM, final solvent concentration 1.6%) were incubated with microsomes at 37 °C, shaking at 100 rpm. Each reaction was performed in duplicates. Five time points over 40 minutes were analyzed. The reactions were stopped by adding 4 volumes of methanol containing internal standard to incubation aliquots, followed by protein sedimentation by centrifuging at 5500 rpm for 4 minutes. Each reaction was performed in duplicates. Supernatants were analyzed using the HPLC system coupled with tandem mass spectrometer.

The elimination constant (k_{el}), half-life ($t_{1/2}$) and intrinsic clearance (Cl_{int}) were determined in plot of ln(AUC) versus time, using linear regression analysis:[8]

$$k_{el} = -slope \qquad t_{\frac{1}{2}} = \frac{0.693}{k} \qquad Cl_{\text{int}} = \frac{0.693}{t_{1/2}} \times \frac{\mu l_{incubation}}{mg_{microsomes}}$$

[8] In order to indicate the quality of the linear regression analysis, the R (correlation coefficient) values are provided. In some cases, the last time point is excluded from the calculations to ensure acceptable logarithmic linearity of decay.

Results

Human microsomal stability data for reference and test compounds is provided in the tables below.

Table S3. Human microsomal stability (Batch #1)

Compound ID	Time, min	Peak Area Ratio		Peak Area Ratio, Mean of 2	% Remaining, Mean of 2	R	k_{el}, min^{-1}	$t_{1/2}$, min	Cl_{int}, µl/min/mg	% Remaining without cofactor, Mean of 2
		Inc. 1	Inc. 2							
1	2	3	4	5	6	7	8	9	10	11
Diclofenac human	0	3.15E+00	3.07E+00	3.11E+00	100	0.995	0.118	5.9	285	100
	7	1.50E+00	1.67E+00	1.59E+00	51	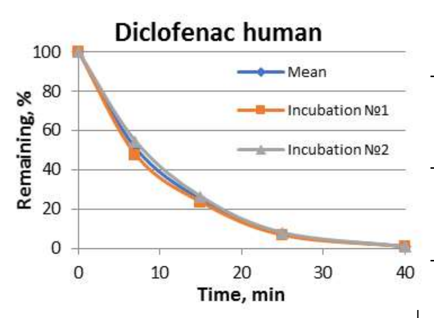				
	15	7.41E-01	8.05E-01	7.73E-01	25					
	25	2.13E-01	2.44E-01	2.29E-01	7					
	40	2.90E-02	2.60E-02	2.75E-02	1					90
Propranolol human	0	6.78E-01	6.54E-01	6.66E-01	100	0.959	0.007	95.7	17	100
	7	5.85E-01	6.07E-01	5.96E-01	89	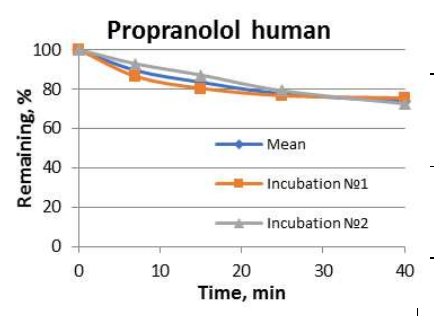				
	15	5.44E-01	5.70E-01	5.57E-01	84					
	25	5.19E-01	5.18E-01	5.19E-01	78					
	40	5.10E-01	4.75E-01	4.93E-01	74					84
**EN300-37082352 (34)** human	0	5.00E-02	5.52E-02	5.26E-02	100	0.998	0.065	10.6	157	100
	7	4.21E-02	3.83E-02	4.02E-02	76	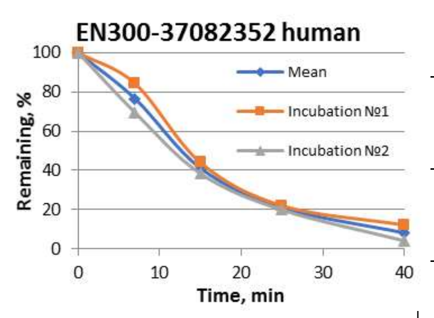				
	15	2.20E-02	2.12E-02	2.16E-02	41					
	25	1.10E-02	1.10E-02	1.10E-02	21					
	40	6.08E-03	2.29E-03	4.19E-03	8					91

1	2	3	4	5	6	7	8	9	10	11
**EN300-18202568** Phthalylsul-fathiazole human	0	3.01E-02	3.23E-02	3.12E-02	100	0.471	0.001*	959.7*	2*	100
	7	2.77E-02	3.31E-02	3.04E-02	97	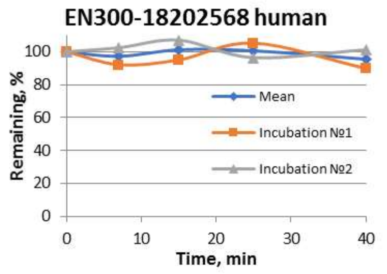				
	15	2.86E-02	3.46E-02	3.16E-02	101					
	25	3.17E-02	3.11E-02	3.14E-02	101					
	40	2.70E-02	3.27E-02	2.99E-02	96					124
**EN300-37082350 (35)** human	0	2.35E-02	1.56E-02	1.96E-02	100	0.954	0.036	19.2	87	100
	7	2.32E-02	1.65E-02	1.99E-02	102	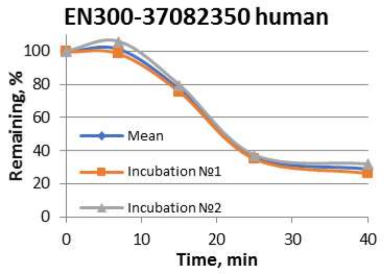				
	15	1.78E-02	1.24E-02	1.51E-02	77					
	25	8.26E-03	5.80E-03	7.03E-03	36					
	40	6.23E-03	4.97E-03	5.60E-03	29					84
**EN300-264529 Fluxapyro-xad** human	0	9.53E-03	1.11E-02	1.03E-02	100	0.972	0.012	59.0	28	100
	7	1.01E-02	9.65E-03	9.88E-03	96	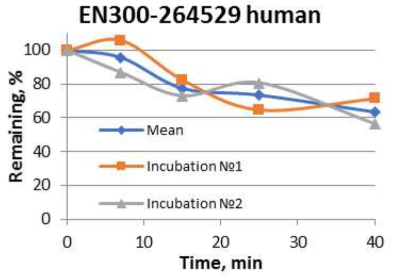				
	15	7.87E-03	8.10E-03	7.99E-03	77					
	25	6.19E-03	8.96E-03	7.58E-03	73					
	40	6.83E-03	6.25E-03	6.54E-03	63					79
**EN300-7394812 Boscalid** human	0	4.53E-02	4.46E-02	4.50E-02	100	0.994	0.011	63.8	26	100
	7	4.20E-02	4.35E-02	4.28E-02	95	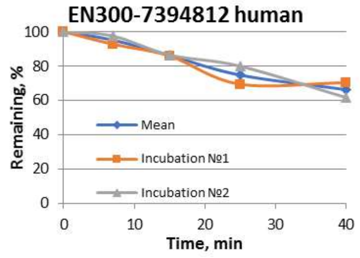				
	15	3.89E-02	3.86E-02	3.88E-02	86					
	25	3.14E-02	3.57E-02	3.36E-02	75					
	40	3.18E-02	2.76E-02	2.97E-02	66					59**

1	2	3	4	5	6	7	8	9	10	11
**EN300-37335003 (28)** human	0	1.25E-02	1.24E-02	1.25E-02	100	0.935	0.015	47.4	35	100
	7	1.04E-02	9.02E-03	9.71E-03	78	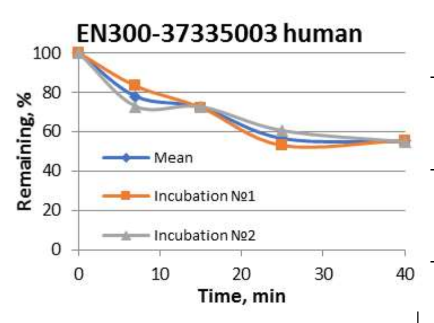				
	15	9.03E-03	9.00E-03	9.02E-03	72					
	25	6.63E-03	7.49E-03	7.06E-03	57					
	40	6.96E-03	6.77E-03	6.87E-03	55					99
**EN300-36480610 (29)** human	0	5.10E-01	5.69E-01	5.39E-01	100	0.957	0.009	73.4	23	100
	7	5.57E-01	5.86E-01	5.72E-01	106	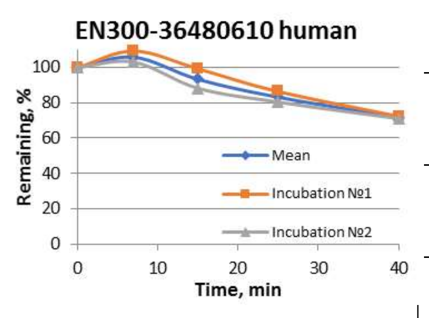				
	15	5.06E-01	5.02E-01	5.04E-01	93					
	25	4.41E-01	4.58E-01	4.50E-01	83					
	40	3.70E-01	4.05E-01	3.88E-01	72					89
**EN300-7460805 (30)** human	0	2.53E-02	2.54E-02	2.54E-02	100	0.956	0.005	138.5	12	100
	7	2.46E-02	2.30E-02	2.38E-02	94	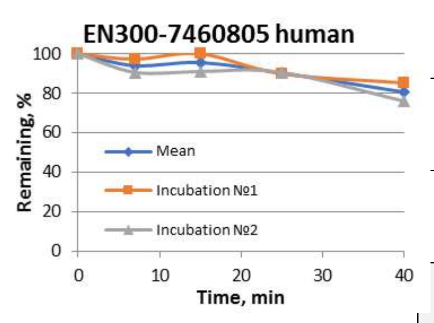				
	15	2.53E-02	2.31E-02	2.42E-02	95					
	25	2.27E-02	2.30E-02	2.29E-02	90					
	40	2.15E-02	1.93E-02	2.04E-02	80					71**

*Parameter should be considered as approximate due to the high stability of the compound.
**"No cofactor" control data indicates that the instability of compound is partially or completely not determined by CYP450 activity

Table S3 (*continued*). Human microsomal stability (Batch #2)

Compound ID	Time, min	Peak Area Ratio		Peak Area Ratio, Mean of 2	% Remaining, Mean of 2	R	k_{el}, min^{-1}	$t_{1/2}$, min	Cl_{int}, μl/min/mg	% Remaining without cofactor, Mean of 2
		Inc. 1	Inc. 2							
1	2	3	4	5	6	7	8	9	10	11
Diclofenac human	0	2.04E+00	1.80E+00	1.92E+00	100	0.996	0.115	6.0	276	100
	7	1.08E+00	9.78E-01	1.03E+00	54	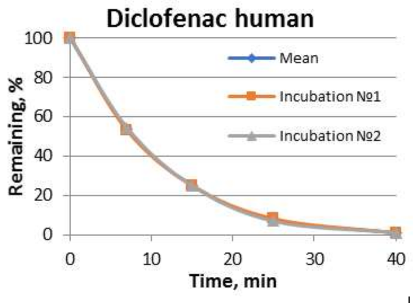				
	15	5.09E-01	4.48E-01	4.79E-01	25					
	25	1.66E-01	1.26E-01	1.46E-01	8					
	40	2.00E-02	1.98E-02	1.99E-02	1					146
Propranolol human	0	8.23E-02	6.81E-02	7.52E-02	100	0.951	0.007	98.1	17	100
	7	7.39E-02	6.08E-02	6.74E-02	90	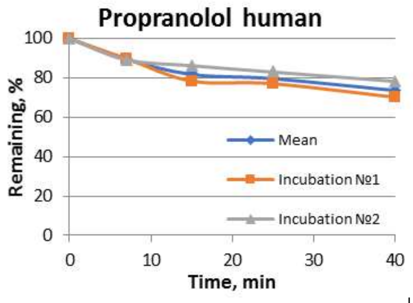				
	15	6.44E-02	5.88E-02	6.16E-02	82					
	25	6.33E-02	5.66E-02	6.00E-02	80					
	40	5.76E-02	5.34E-02	5.55E-02	74					93
**EN300-37274652 (33)** human	0	1.21E+00	1.22E+00	1.22E+00	100	NA	NA	1185.6*	1*	100
	7	1.31E+00	1.39E+00	1.35E+00	111	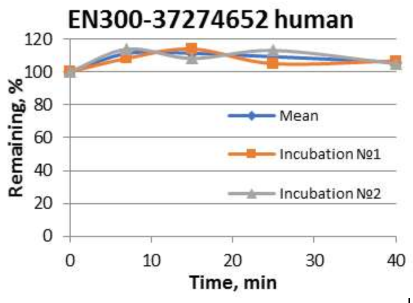				
	15	1.38E+00	1.32E+00	1.35E+00	111					
	25	1.27E+00	1.38E+00	1.33E+00	109					
	40	1.29E+00	1.28E+00	1.29E+00	106					92

1	2	3	4	5	6	7	8	9	10	11
**EN300-37274650 (32)** human	0	2.42E+00	2.33E+00	2.38E+00	100	NA	0.000*	4087.7*	0*	100
	7	2.23E+00	2.40E+00	2.32E+00	97	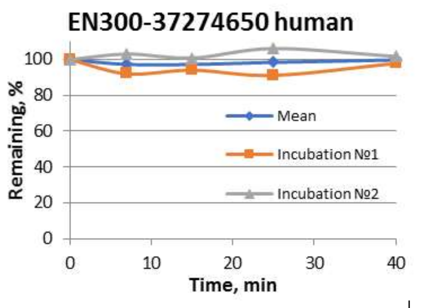				
	15	2.28E+00	2.35E+00	2.32E+00	97					
	25	2.21E+00	2.47E+00	2.34E+00	99					
	40	2.37E+00	2.37E+00	2.37E+00	100					89
**EN300-37152265 (31)** human	0	1.35E+00	1.17E+00	1.26E+00	100	NA	NA	485.1*	3*	100
	7	1.39E+00	1.30E+00	1.35E+00	107	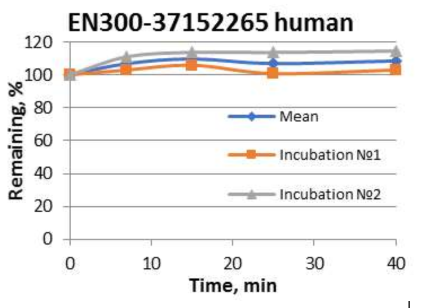				
	15	1.43E+00	1.33E+00	1.38E+00	110					
	25	1.36E+00	1.33E+00	1.35E+00	107					
	40	1.39E+00	1.34E+00	1.37E+00	108					85
**EN300-20331690 Lopitami-de** human	0	3.38E-02	3.94E-02	3.66E-02	100	0.928	0.023	30.4	55	100
	7	3.17E-02	3.94E-02	3.56E-02	97	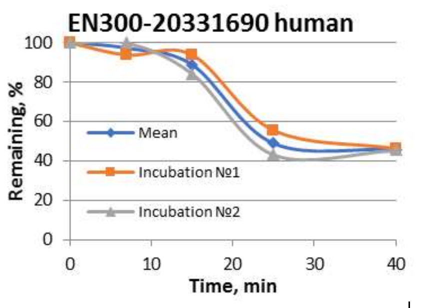				
	15	3.17E-02	3.32E-02	3.25E-02	89					
	25	1.88E-02	1.70E-02	1.79E-02	49					
	40	1.57E-02	1.79E-02	1.68E-02	46					88

*Parameter should be considered as approximate due to the high stability of the compound.

Interpretation of microsomal stability assay data

The test compounds can be classified in terms of their microsomal stability into low, medium and high clearance groups. The intrinsic clearance classification bands for human, rat, and human species are calculated according to the well stirred model equation:[1]

$$CL_{int} = \frac{CL_H}{fu \times (1 - E)}$$

where CL_H is a hepatic clearance (mL/min/kg), $CL_H = E \times Q_H$

Q_H = liver blood flow (mL/min/kg)[2]
E = extraction ratio, assumed at 0.3 for low clearance and at 0.7 for high clearance compounds
fu = fraction unbound in plasma, assumed at 1.

The CL_{int} classification values were calculated for mouse, rat, and human species using the literature data on liver weight[3] and microsomal protein concentration[3,4] and are represented in the following table.

Table S4. The intrinsic clearance groups for classification of test compounds

Classification group	Intrinsic clearance (µL/min/mg protein)		
	Mouse	Rat	Human
**Low clearance**	< 8.6	< 13	< 8.8
**High clearance**	> 48	> 72	> 48

[1]. J. B. Houston Utility of *in vitro* drug metabolism data in predicting *in vivo* metabolic clearance. Biochemical Pharmacology, **1994**, *47*, 1469-1479.

[2]. B. Davies and T. Morris. Physiological parameters in laboratory animals and humans. *Pharmaceutical Research, 1993,* **10,** 1093-1095.

[3]. Z. E. Barter *et al.* Scaling factors for the extrapolation of *in vivo* metabolic drug clearance from *in vitro* data: reaching a consensus on values of human microsomal protein and hepatocellularity per gram of liver. *Current Drug Metabolism,* **2007***, 8,* 33-45.

[4]. T. Iwatsubo *et al.* Prediction of species differences (rats, dogs, humans) in the *in vivo* metabolic clearance of YM796 by the liver from *in vitro* data. *Journal of Pharmacology and Experimental Therapeutics,* **1997***, 283*, 462-469.

Bioactivity

The agar well diffusion method is widely used to evaluate the antimicrobial activity of different substances (S. Magaldi, S. Mata-Essayag, C. Hartung de Capriles, et al. Well diffusion for antifungal susceptibility testing, *Int. J. Infect. Dis. 8*, **2004**, 39–45; C. Valgas, S. M. De Souza, E. F. A. Smânia, et al. Screening methods to determine antibacterial activity of natural products. *Braz. J. Microbiol. 38*, **2007**, 369–380). Similarly to the procedure used in a disk-diffusion method, the agar plate surface is inoculated by spreading a volume of the microbial inoculum over the entire agar surface. Then, a hole with a diameter of 6 to 8 mm is punched aseptically with a sterile cork borer or a tip, and a volume (20–100 mL) of the antimicrobial agent or extract solution at desired concentration is introduced into the well. Then, agar plates are incubated under suitable conditions depending upon the test microorganism. The antimicrobial agent diffuses in the agar medium and inhibits the growth of the microbial strain tested (M. Balouiri, M. Sadiki, S. K. Ibnsouda. Methods for in vitro evaluating antimicrobial activity. *J Pharm Anal.* **2016**, 71-79. doi:10.1016/j.jpha.2015.11.005).

Agar well diffusion method is a qualitative method used to measure the ability of compounds to inhibit microbial growth. This test is a quick and easy way to measure and compare levels of inhibitory activity.

Antifungal activity of the synthetic compounds using agar well diffusion

The compunds were tested towards plant pathogens *Fusarium oxysporum* Schltdl. and *F. verticillioides (Sacc.) Nirenberg* isolated from crops. Antifungal activity of the synthetic compounds was evaluated with using of an agar well diffusion method based on a disk diffusion assay for testing filamentous fungi (CLSI M51-A) [CLSI. Method for Antifungal Disk Diffusion Susceptibility Testing of Nondermatophyte Filamentous Fungi; Approved Guideline.CLSI document M51-A. Wayne, PA: Clinical and Laboratory Standards Institute; 2010. This method is standardized for *Altenaria*, *Aspergillus, Bipolaris*, *Fusarium*, order *Mucorales* etc.

Preparation of synthetic compounds for mycological assay

Solutions of fungicides and its analogs were diluted in dimethyl sulfoxide (DMSO) to produce the following concentrations: 0.0039, 0.0078, 0.0156, 0.03125, 0.0625, 0.125, 0.250, 0.5 and 1 µg/mL for each test component.

Antifungal well diffusion method

The fungal strains were cultured on potato dextrose agar slants (PDA, Eur. Pharm.) and incubated at 25 °C for 5 days. Sterile distilled water was used to cover and resuspend the colonies. Suspension of

conidia was adjusted to inoculums 5×10^6 conidia/mL. The suspension was further diluted in warm PDA 1:10 to obtain final working inoculums 5×10^5 conidia/mL.

Polypropylene cylinders (d, 5 mm) were placed on the surface and wells punched in the medium were used as reservoir of the test substances in different concentrations. 40 µl of solutions containing 0.0039-1.0 mg/mL of a test compound was added into the wells.

Each compound was tested in triplicate at different concentrations. Growth control was a well with 40 µl of DMSO, which was used for test components dilution.

Plates incubated at 25 °C for 72 h. The test compounds at known concentration into contact with an inoculated medium then exerts a growth-inhibiting effect then a clear zone (the zone of inhibition) appears around the test product. Diameter of a clear zone around the well is measured at the end of the incubation period in millimeters. If the fungal strain is susceptible to the antifungal agent, then a zone of inhibition appears on the agar plate. If it is resistant to the test compound, then no zone is evident.

The size of the zone of inhibition is usually related to the level of antifungal activity present in the compound – a larger zone of inhibition usually means that the antimicrobial is more potent.

The growth rate of *Fusarium oxysporum* and *F. verticillioides* for each well was determined visually and compared with the growth of control.

Results

As shown in the table the antifungal activity of the 6 compounds, - Fluxapyroxad, compound 28, compound 29, Boscalid, compound 30, compound 31, - were tested against plant pathogenic fungal strains. These substances demonstrated different level of antifungal activity at studied concentrations toward *Fusarium oxysporum* (Table S5, Figure S16). Inhibition zones of all compounds characterized by static growth of the fungus and changed morphology. Compounds 1, 2, 4 and 5 significantly reduced development of *F. oxysporum* at concentrations 0.0625-1.0 mg/mL and showed inhibition zones ranging from 24 to 44 mm. In our experiment the lowest inhibition concentration for compounds 29, Boscalid and 31 was 0.0039 mg/mL, for compound Fluxapyroxad, 28 and 30 it was below than 0.0039 mg/mL.

Strain *Fusarium verticillioides* showed suscaptibility to most studied compounds (table). Influence zones of all compounds had a static growth of the fungus and diminishing its mycelial growth that lost its density in comparison with control (Figure S17).

Compounds Fluxapyroxad and 28 significantly reduced development of *F. verticillioides* at all studied concentrations and showed inhibition zones ranging from 19 to 58 mm. In our experiment the lowest inhibition concentration for compounds 29 and Boscalid was 0.0078 mg/mL, for compound 30 it was below than 0.0039 mg/mL.

Compound 6 demonstrated gradually decreasing of its antifungal activity at studied concentrations against *F. oxysporum*. Fungus *F. verticillioides* was more resistant to this compound. Its fungicidal effect dramatically dropped at concentration below 0.25 mg/mL.

Thus, compounds studied demonstrated different antifungal activity. *In vitro* study showed higher level of antifungal activity of test compounds towards *F. verticillioides*.

Table S5. The antifungal activity of compounds studied

Concentration, mg/mL	Fungal test-culture											
	Fusarium oxysporum						*Fusarium verticillioides*					
	Test compound											
	**Fluxapyroxad**	(±)-**28**	(±)-**29**	**Boscalid**	(±)-**30**	(±)-**31**	**Fluxapyroxad**	(±)-**28**	(±)-**29**	**Boscalid**	(±)-**30**	(±)-**31**
	Diameter of inhibition zone, mm											
1	43±1.53 st	44±1.15 st	38±1.53 st	39±0.6 st	30±0.0 st	25±0.6 st	56±1.7 st	58±1.5 st	45±0.6 st	44±1.2 st	49±1.2 st	29±1.2 st
0.5	38±1.5 st	38±0.6 st	36±1.0 st	31±1.2 st	30±0.0 st	21±1.0 st	49±1.2 st	51±1.7 st	41±1.7 st	40±0.6 st	46±1.7 st	23±1.5 st
0.25	35±0.6 st	38±1.5 st	32±1.7 st	30±0.0 st	30±0.0 st	18±1.0 st	40±1.0 st	51±1.5 st	39±1.2 st	39±0.6 st	41±1.2 st	16±1.2 st
0.125	33±1.0 st	34±1.2 st	23±0.6 st	25±0.0 st	30±0.0 st	19±1.0 st	36±0.6 st	48±1.5 st	35±1.0 st	35±1.0 st	36±1.7 st	10±0.6 st
0.0625	30±0.6 st	30±0.6 st	18±0.6 st	24±1.0 st	25±0.0 st	18±0.6 st	34±1.2 st	47±1.0 st	31±1.2 st	30±0.6 st	31±1.0 st	0
0.03125	28±1.5 st	28±1.0 st	18±1.5 st	12±1.7 st	25±0.6 st	16±1.0 st	33±0.6 st	41±1.7 st	26±1.2 st	28±1.0 st	30±0.6 st	0
0.0156	22±1.7 st	26±1.0 st	14±1.2 st	11±1.5 st	20±1.5 st	14±1.0 st	30±1.5 st	41±1.2 st	15±0.6 st	24±1.5 st	25±0.6 st	0
0.0078	17±1.2 st	25±0.0 st	13±1.5 st	9±1.7 st	19±1.2 st	9±0.6 st	26±1.2 st	37±1.5 st	10±0.0 st	11±1.0 st	19±1.2 st	0
0.0039	14±1.2 st	20±0.0 st	9±1.2 st	9±0.6 st	16±1.2 st	8±0.6 st	19±0.6 st	35±1.5 st	0	0	11±1.2 st	0
Control DMSO	0						0					

Abbreviation: 0 – absence of antifungal activity; st – static growth, when fungal mycelium has secondary growth

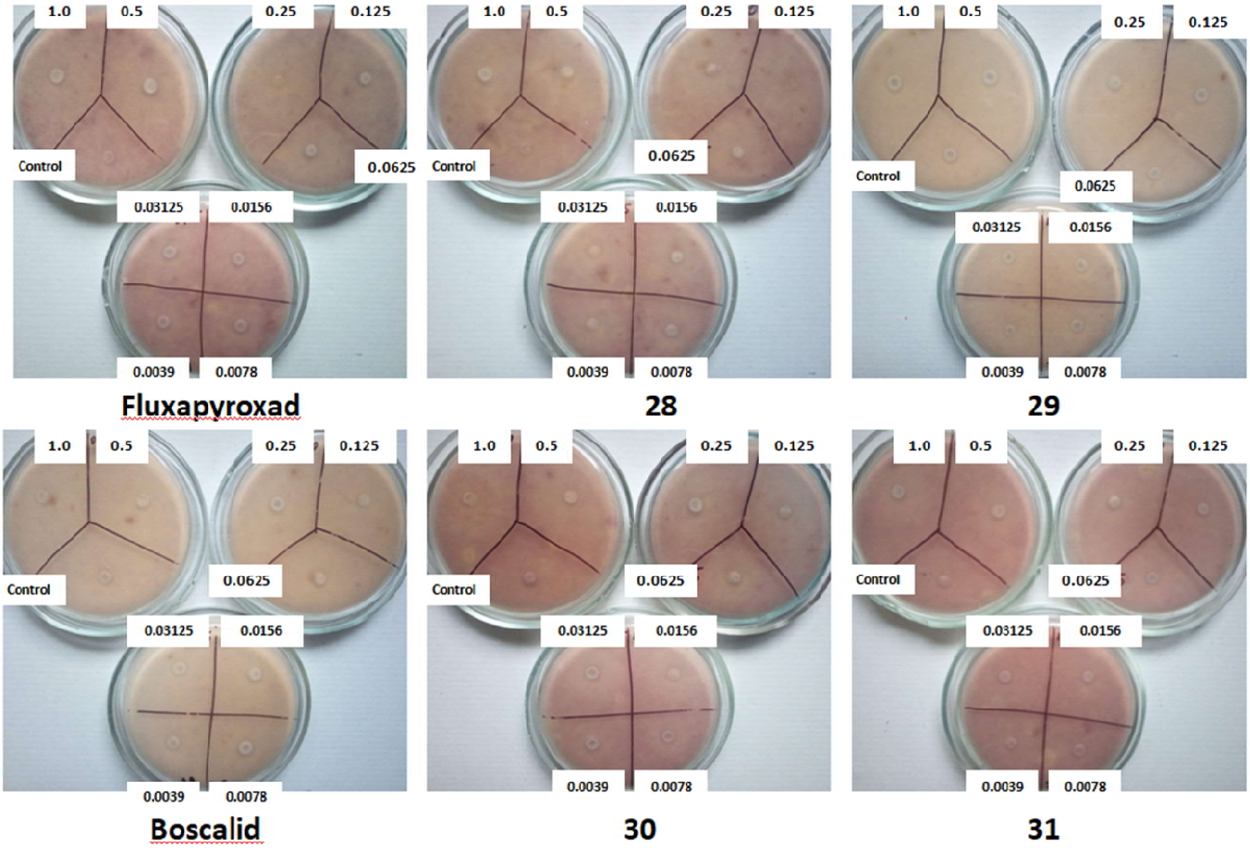

Figure S16. Activity of compounds toward *Fusarium oxysporum*, plates in 72 h (reverse side of Petri dish)

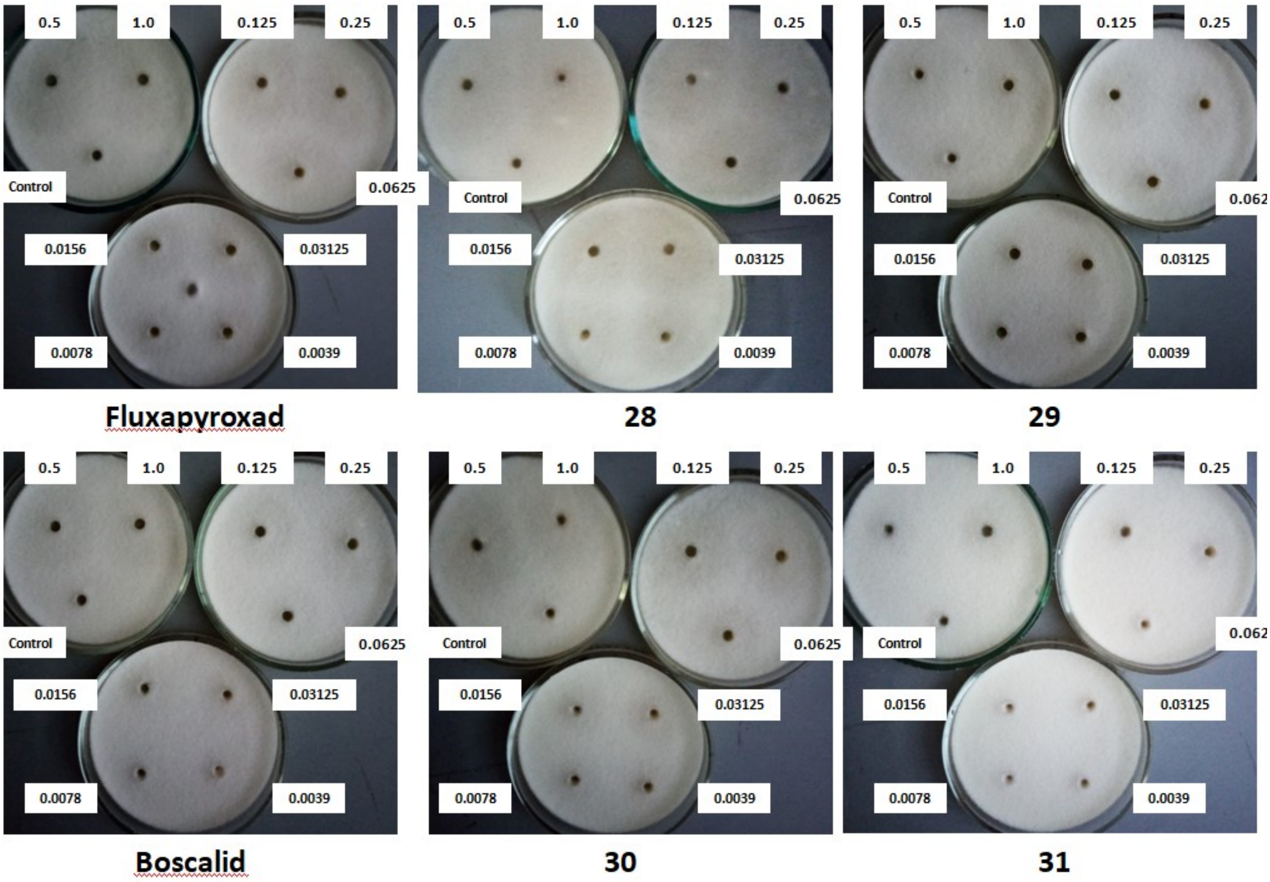

Figure S17. Activity of compounds toward *Fusarium verticillioides*, plates in 72 h (upside of Petri dish)

Antifungal susceptibility testing
(determination of MIC values)

The antifungal susceptibility tests were performed according to the Clinical and Laboratory Standards Institute (CLSI) M38-A2 protocol for mycelial fungi (1). Stock solutions were prepared by dissolving an appropriate amount of testing compounds in DMSO. Solutions of test compounds were added to the wells of a 96-well polystyrene microtiter plate (Colstar, USA) in the concentration range from 0.5 to 0.001 mg/mL. Mycelium growth was monitored photometrically at 490 nm by measuring the optical density of each well. Measurements were carried out two times: immediately after adding all components (RPMI 1640 broth, conidial suspension, test compound solutions) and after incubation plate at 26 °C for 48 h (2). MICs were the lowest testing compound concentrations that produced partial growth inhibition (50% of optical density relative to the control without testing compounds). The tests were performed in triplicate

Results:

Table S6. The MIC values of *Fluxapyroxad*, *Boscalid*, and their saturated analogues against *Fusarium oxysporum*, and *Fusarium verticillioides*

Antifungal compaunds	MIC, mg/mL	
	Fusarium oxysporum	*Fusarium verticillioides*
F**luxapyroxad**	-*	0.250
Analogue (±)-**28**	0.125	0.125
Analogue (±)-**29**	- **	0.250
**Boscalid**	0.250	- ***
Analogue (±)-**30**	0.031	0.031
Analogue (±)-**31**	0.250	- ****

** Fluxapyroxad maximally inhibited the growth of F. oxysporum by 38.0 ± 1.9 % at a concentration of 0.031 mg/mL.*
*** Analogue 29 maximally inhibited the growth of F. oxysporum by 35.7 ± 2.4 % at a concentration of 0.125 mg/mL.*
**** Boscalid maximally inhibited the growth of F. verticillioides by 39.2 ± 2,7 % at a concentration of 0.063 mg/mL.*
***** Analogue 31 maximally inhibited the growth of F. verticillioides by 36.3 ± 1,9 % at a concentration of 0.250 mg/mL.*

All tested substances only partially inhibited mycelium growth. This phenomenon has also been described for other fungicides (fluconazole, ketoconazole, and flucytosine) when tested by the microtitration method according to the CLSI or EUCAST protocols (3). Also, in this study, we observed the Eagle-like effect: instead of consistent growth across the wells, there is a distinct drop-off in growth and then a distinct regrowth further up the concentration gradient (4). It was because high concentrations of fungicides precipitated from a liquid medium.

The results of this study indicate that the saturated analogues of Fluxapyroxad, and Boscalid, **28** and **30** respectively, which contain bicyclo[2.1.1]hexane core showed more significant fungistatic activity against both testing fungal strains, than Fluxapyroxad, Boscalid, and their analogues **29**, **31** with 2-oxabicyclo[2.1.1]hexane core (Table S6). At the same time, analogues **29** and **31** had the same fungicidal activity as Fluxapyroxad, and Boscalid, respectively.

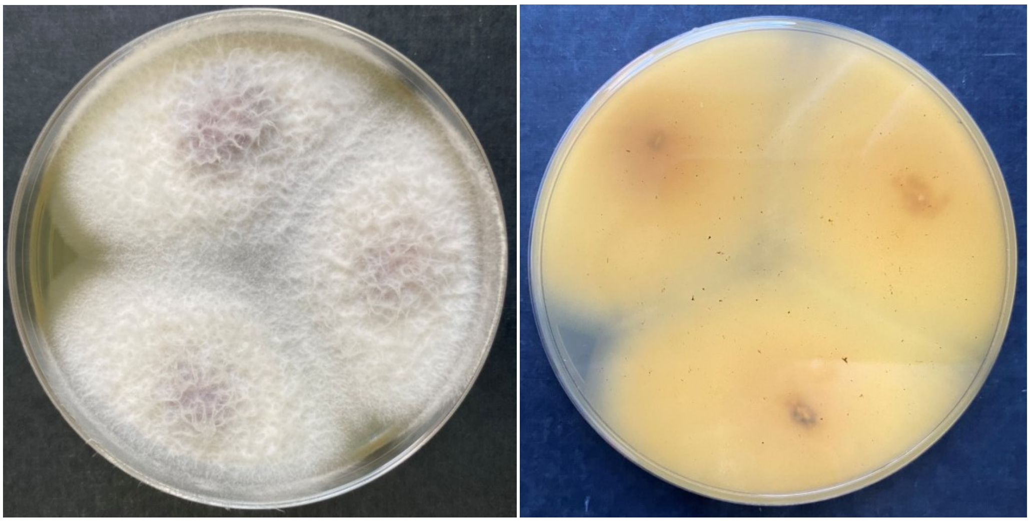

Figure S18. Colonies of *Fusarium verticilliodes* on PDA medium after 7 d at 25 °C, obverse (left) and reverse (right).

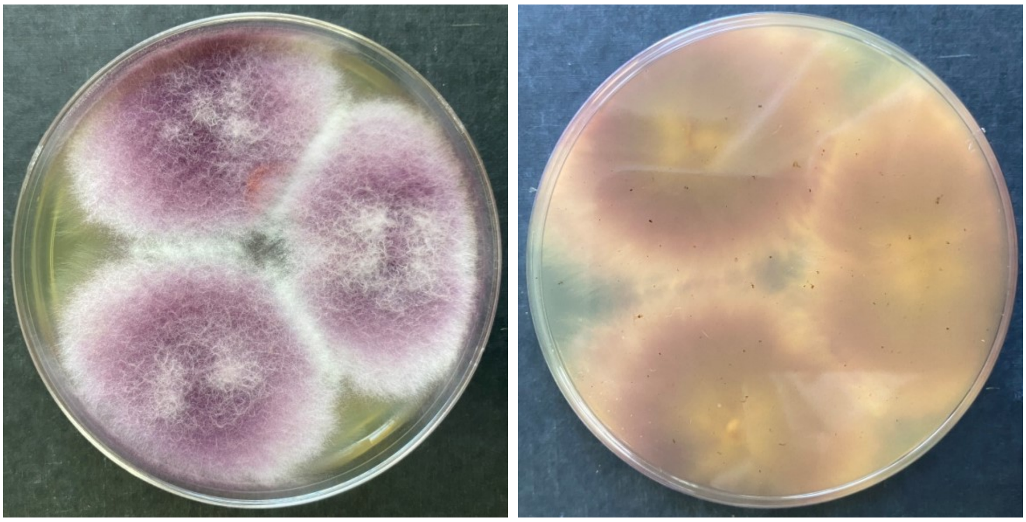

Figure S19. Colonies of *Fusarium oxysporum* on PDA medium after 7 d at 25 °C, obverse (left) and reverse (right).

References:

1. Clinical and Laboratory Standards Institute. 2008. Reference method for broth dilution antifungal susceptibility testing of filamentous fungi, 2nd ed Approved standard M38-A2 Clinical and Laboratory Standards Institute, Wayne, PA.

2. K. H. S. Kouassi, M. Bajji, Y. Brostaux, A. Zhiri, A. Samb, P. Lepoivre, M. H. Jijakli. Development and application of a microplate method to evaluate the efficacy of essential oils against Penicillium italicum Wehmer, Penicillium digitatum Sacc. and Colletotrichum musea (Berk. MA Curtis) Arx, three postharvest fungal pathogens of fruits. *Biotechnol. Agron. Soc. Environ*. **2012**. *16*, 325-336.

3. A. Espinel-Ingroff, D. P. B. Abreu, R. Almeida-Paes, R. S. N. Brilhante, A. Chakrabarti, A. Chowdhary, F. Hagen, S. Córdoba, G. M. Gonzalez, N. P. Govender, J. Guarro, E. M. Johnson, S. E. Kidd, S. A.Pereira, A. M. Rodrigues, S. Rozental, M. W. Szeszs, R. Ballesté Alaniz, A. Bonifaz, L. X. Bonfietti, L. P. Borba-Santos, J. Capilla, A. L. Colombo, M. Dolande, M. G. Isla, M. S. C. Melhem, A. C. Mesa-Arango, M. M. E. Oliveira, M. M. Panizo, Z. Pires de Camargo, R. M. Zancope-Oliveira, J. F. Meis, J. Turnidge. Multicenter, International Study of MIC/MEC Distributions for Definition of Epidemiological Cutoff Values for Sporothrix Species Identified by Molecular Methods. *Antimicrob Agents Chemother*. **2017**. *61*, e01057-17.

4. E. L. Berkow, S. R. Lockhart, L. Ostrosky-Zeichner. Antifungal Susceptibility Testing: Current Approaches. *Clin Microbiol Rev*. **2020**. *33*, e00069-19.
