# Supplementary material for: 2-Oxabicyclo[2.1.1]hexanes as saturated bioisosteres of the ortho-substituted phenyl ring
Source: Nat Chem. 2023 Jun 5;15(8):1155–63. doi: 10.1038/s41557-023-01222-0 (PMC10396955; doi:10.1038/s41557-023-01222-0)
Supplement: Supplementary file 2 — Reporting Summary [file 41557_2023_1222_MOESM2_ESM.pdf]

nature research

Corresponding author(s): Pavel K. Mykhailiuk

Last updated by author(s): Apr 25, 2023

Reporting Summary

Nature Research wishes to improve the reproducibility of the work that we publish. This form provides structure for consistency and transparency in reporting. For further information on Nature Research policies, see our Editorial Policies and the Editorial Policy Checklist.

Statistics

For all statistical analyses, confirm that the following items are present in the figure legend, table legend, main text, or Methods section.

n/a	Confirmed	
☒	☐	The exact sample size (n) for each experimental group/condition, given as a discrete number and unit of measurement
☒	☐	A statement on whether measurements were taken from distinct samples or whether the same sample was measured repeatedly
☒	☐	The statistical test(s) used AND whether they are one- or two-sided<br>*Only common tests should be described solely by name; describe more complex techniques in the Methods section.*
☒	☐	A description of all covariates tested
☒	☐	A description of any assumptions or corrections, such as tests of normality and adjustment for multiple comparisons
☒	☐	A full description of the statistical parameters including central tendency (e.g. means) or other basic estimates (e.g. regression coefficient) AND variation (e.g. standard deviation) or associated estimates of uncertainty (e.g. confidence intervals)
☒	☐	For null hypothesis testing, the test statistic (e.g. F, t, r) with confidence intervals, effect sizes, degrees of freedom and P value noted<br>*Give P values as exact values whenever suitable.*
☒	☐	For Bayesian analysis, information on the choice of priors and Markov chain Monte Carlo settings
☒	☐	For hierarchical and complex designs, identification of the appropriate level for tests and full reporting of outcomes
☒	☐	Estimates of effect sizes (e.g. Cohen's d, Pearson's r), indicating how they were calculated

Our web collection on statistics for biologists contains articles on many of the points above.

Software and code

Policy information about availability of computer code

Data collection	The irradiation experiments were performed using lamps Sylvania 368 Blacklight F25/T8/18/BL3368. Product purification was performed using HPLC AGILENT 1260 INFINITY (a column Chromatorex C18 SMB 100-5T, 100*19 mm, 5 microm) or PuriFlash XS420 Plus. The NMR data acquisition was performed using Varian UNITY III 400; Varian VNMRS 500; Bruker AVANCE DRX 500 and Bruker AVANCE III 400 spectrometers. HRMS data acquisition was performed using Agilent 6224 TOF LC/MS. Lipophilicity (clogP) was calculated with “Cxcalc” ChemAxon, version 22.5.0.
Data analysis	The NMR data analysis was performed using Mestrenova software (11.0.3-18688). The data acquisition and system control was performed using Analyst 1.6.3 software from AB Sciex.

For manuscripts utilizing custom algorithms or software that are central to the research but not yet described in published literature, software must be made available to editors and reviewers. We strongly encourage code deposition in a community repository (e.g. GitHub). See the Nature Research guidelines for submitting code & software for further information.

Data

Policy information about availability of data

All manuscripts must include a data availability statement. This statement should provide the following information, where applicable:

- Accession codes, unique identifiers, or web links for publicly available datasets
- A list of figures that have associated raw data
- A description of any restrictions on data availability

All data are available in the SI (Supporting Information)

Field-specific reporting

Please select the one below that is the best fit for your research. If you are not sure, read the appropriate sections before making your selection.

☒ Life sciences ☐ Behavioural & social sciences ☐ Ecological, evolutionary & environmental sciences

For a reference copy of the document with all sections, see nature.com/documents/nr-reporting-summary-flat.pdf

Life sciences study design

All studies must disclose on these points even when the disclosure is negative.

Sample size	All syntheses were performed on 100mg-20g scale. Analysis of reaction mixtures was performed with NMR and HRMS techniques. Typical sample size for analysis is 20 mg.
Data exclusions	no data were excluded from the analysis
Replication	All attempts at replication were successful. Experiments were performed minimal twice: on small scale first (20 mg), and on gram scale (5-20 g).
Randomization	not relevant in organic synthesis.
Blinding	In antifungal experiments, control tests (vehicle) were also performed.

Reporting for specific materials, systems and methods

We require information from authors about some types of materials, experimental systems and methods used in many studies. Here, indicate whether each material, system or method listed is relevant to your study. If you are not sure if a list item applies to your research, read the appropriate section before selecting a response.

Materials & experimental systems

n/a	Involved in the study	
☒	☐	Antibodies
☐	☒	Eukaryotic cell lines
☒	☐	Palaeontology and archaeology
☒	☐	Animals and other organisms
☒	☐	Human research participants
☒	☐	Clinical data
☒	☐	Dual use research of concern

Methods

n/a	Involved in the study	
☒	☐	ChIP-seq
☒	☐	Flow cytometry
☒	☐	MRI-based neuroimaging

Eukaryotic cell lines

Policy information about cell lines

Cell line source(s)	Single-spore isolates of Fusarium oxysporum Schltdl. and F. verticillioides (Sacc.) Nirenberg (formerly known as F. moniliforme J. Sheld.) were obtained from organic corn plants with rot symptoms and with no previous history of exposure to any fungicides.
Authentication	Identification of Fusarium species was performed according to specific morphological characterization of their sporulation structure. Sing-spore isolates were grown at 20°C in complete darkness for 14 days on synthetic nutrient-poor agar (SNA) to examine the production, type, and arrangement of microconidia and conidiogenous cells. Isolates were incubated at 25°C with a 12-h photoperiod for 14 days on carnation leaf agar (CLA) to examine the shape and size of macroconidia. Microconidia, macroconidia, and chlamydospores were measured based on 30 random selections.<br>Molecular genetic identification of isolates proved that they belonged to Fusarium genus. The verification was carried out by using method of polymerase chain reaction (PCR) with detection of amplification products in agarose gel. The DNA was extracted out by using guanidine-thiocyanate method with sorption on silicon oxide. Primers that flank part of ITS region of ribosomal DNA of Fusarium spp., 431 n.p. were used. After amplification in thermocycler 2720 (Applied Biosystems)with appropriate temperature mode its products were analyzed by separation in 1,5 % agarose gel.<br>https://www.researchgate.net/<br>publication/338875694_Interlaboratory_aprobation_of_primers_for_molecular_genetic_identification_of_Fusarium_link_fungus]
Mycoplasma contamination	Fusarium strains were not tested for mycoplasma contamination.

Commonly misidentified lines
(See ICLAC register)

Representatives of genus Fusarium are ubiquitous fungi and one of the most important economic plant pathogens causing significant crop losses and contamination of grain by their secondary metabolites (mycotoxins) on a global basis. Many Fusarium species are causative agents of wide range of plant diseases that affect many crops including major food cultures such as wheat, barley, corn, often with social and economic impact.
Fusarium disease is very difficult to control, bacause of its often development in the plant vascular system. Therefore, development of new bioactive compounds against Fusarium fungi is an important research in global scale.
